# Structural Determinants of μ‑Opioid Receptor Antagonism and Respiratory Liability in Phenylfentanyl Analogues

**DOI:** 10.1021/acschemneuro.5c00836

**Published:** 2026-04-30

**Authors:** Ennian Li, Balaji S. Kale, Abeje A. Silte, Rui Lyu, Neha Upadhyay, Logan T. Neel, Ahmed Reda, Huiqun Wang, William L. Dewey, Piyusha P. Pagare, Yan Zhang

**Affiliations:** † Department of Medicinal Chemistry, School of Pharmacy, 6889 Virginia Commonwealth University, 800 East Leigh Street, Richmond, Virginia 23298, United States; ‡ Department of Pharmacology and Toxicology, School of Medicine, Virginia Commonwealth University, 410 North 12th Street, Richmond, Virginia 23298, United States; § Center for Drug Discovery, Virginia Commonwealth University, 800 East Leigh Street, Richmond, Virginia 23298, United States; ∥ Institute for Drug and Alcohol Studies, Virginia Commonwealth University, 203 East Cary Street, Richmond, Virginia 23298, United States

**Keywords:** fentanyl, phenylfentanyl, isosteric modification, core ring extension, respiratory depression

## Abstract

The μ-opioid receptor (MOR) plays a key role in opioid addiction and fentanyl-induced respiratory depression. Phenylfentanyl, previously reported as a MOR antagonist, paradoxically induces respiratory depression in vivo, revealing a dissociation between receptor antagonism and respiratory liability within this scaffold. To interrogate structural determinants underlying this activity profile, phenylfentanyl was redesigned through systematic modification of the acyl chain, *N*-alkyl group, and core ring scaffold, including exploration of a 6,6-ring extension to probe the MOR binding space. Seventy-eight compounds were synthesized and evaluated. The 6,6-ring series exhibited markedly reduced MOR activity relative to the 6-membered core analogues, likely due to suboptimal pharmacophore spacing. Among these, compound **5** demonstrated solid MOR antagonist activity, effectively blocking morphine- and fentanyl-induced antinociception in vivo and showing greater potency than phenylfentanyl in vitro. Importantly, compound **5** did not induce respiratory depression at the tested dose, indicating a lack of intrinsic respiratory liability. These findings delineate structural features governing MOR antagonism and respiratory effects within a fentanyl-derived scaffold.

## Graphical abstract

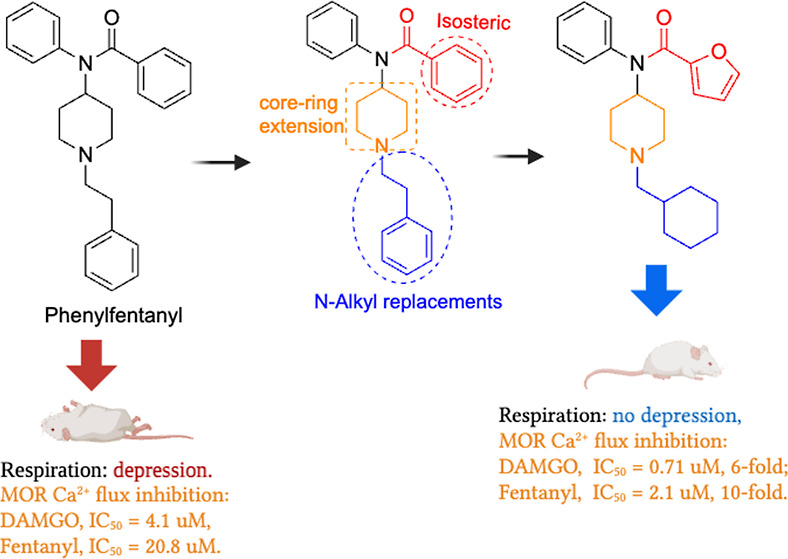

## Introduction

Opioid use disorder remains a pervasive public health issue in the United States and worldwide.^1^ Drug overdose cases have dramatically increased over the last two decades, with deaths increasing more than 500% between 1999 and 2022.^2^ Although there was a remarkable nearly 27% decrease in the predicted drug overdose deaths in 2024 compared to 2023 on average, overdose remains the leading cause of death for Americans ages 18 to 44.^3^ Two FDA-approved drugs for opioid overdose treatment, naloxone and nalmefene, however, both have limitations.^4–6^ For example, both drugs can induce severe opioid withdrawal symptoms. Furthermore, naloxone has a short half-life of about 1 h, which may lead to renarcotization once its effects wear off.^7^ What is more concerning is that naloxone has a relatively low potency to reverse the respiratory depression caused by fentanyl and its analogues, which has been perceived as the major cause for overdose deaths.^8,9^

As reported previously, phenylfentanyl, a fentanyl analogue, behaved as an MOR antagonist against fentanyl and morphine in vitro, while it also induced a respiratory depression effect similar to fentanyl, putatively through a second mechanism via inhibition of the adrenergic system.^10^ To address the limitations of phenylfentanyl, our group previously explored a core-ring expansion strategy along with isosteric substitutions of the acyl and *N*-alkyl groups of phenylfentanyl.^11^ This approach led to the identification of a lead compound, referred to here as **VZFN342**, featuring an 8-membered core ring, a 3-furanyl acyl substituent, and an *N*-allyl replacement of the *N*-ethyl phenyl group (Figure 1). **VZFN342** demonstrated promising opioid receptor antagonism and the ability to counteract fentanyl-induced respiratory depression in mice, providing key mechanistic insights into how core-ring modifications affect receptor binding and functional outcomes.

**Figure 1:**
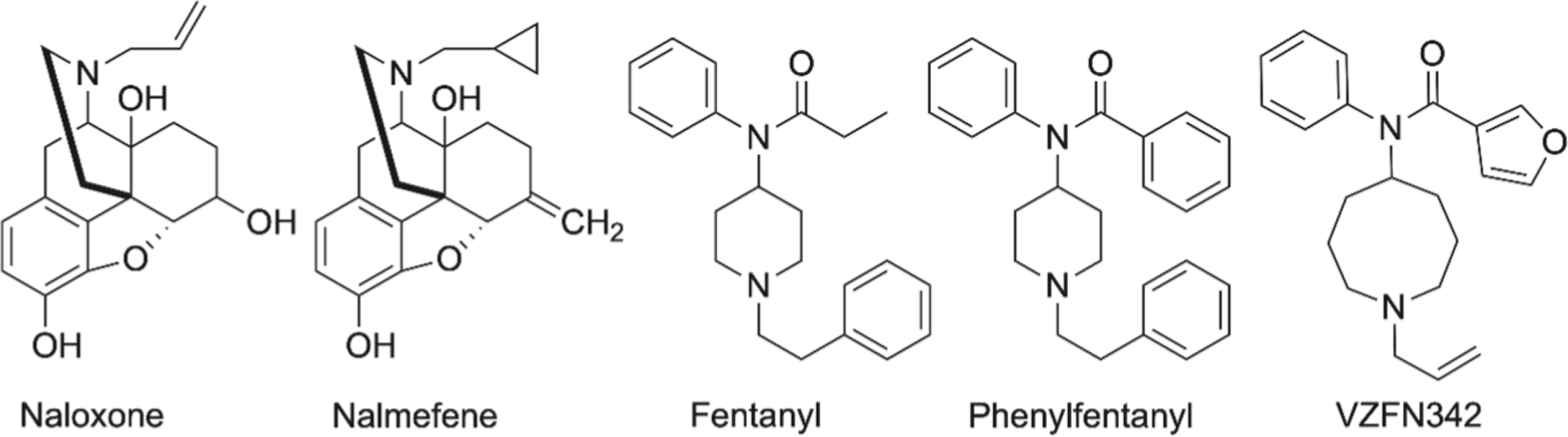
FDA-approved drugs for opioid overdose, fentanyl, phenylfentanyl, and its analog **VZFN342**.

Building on these insights, the present study sought to further define structural determinants governing μ-opioid receptor (MOR) antagonism and intrinsic respiratory liability within the phenylfentanyl scaffold. While outperforming clinical used antagonists will be our ultimate goal, our current objective is to interrogate how defined isosteric substitutions and core-ring modifications influence receptor engagement and functional outcomes in a fentanyl-derived framework. Two main approaches were pursued: (i) isosteric replacements of the phenyl group on the acyl side chain in combination with various *N*-alkyl substitutions and (ii) integration of these isosteric replacements with a 6,6-bicyclic core ring (3-azaspiro[5.5]­undecane scaffold) to investigate the impact of core-ring extension on opioid antagonist potency.

All synthesized compounds, representing a range of potencies to inform structure–activity relationships, were initially evaluated for opioid agonist and antagonist activity in single-dose and dose–response animal models. Calcium mobilization assays were subsequently conducted to confirm MOR pathway involvement of the antagonists, and molecular docking provided insights into potential interactions of these ligands within the inactive MOR binding site. The most potent compound was further characterized for drug-likeness and pharmacokinetic properties through in vitro ADME-Tox profiling. Central nervous system (CNS) exposure was additionally evaluated in vivo using a blood–brain barrier (BBB) penetration study. Selected analogues were assessed in vivo to determine whether structural modifications could dissociate MOR antagonism from intrinsic respiratory depression. Collectively, this approach provides further mechanistic insight into how targeted structural modifications within a fentanyl-derived scaffold influence antagonist function and respiratory liability.

## Rerults and Discussion

### Molecular Design and Synthesis

As showed in Figure 2, to optimize receptor interactions at the R1 position, the phenyl group was replaced with heteroaryl isosteres including furan, thiophene, and pyrrole. These five-membered heterocycles were selected for their similar sizes to the phenyl ring, distinct electronic properties, and potential to engage in additional noncovalent interactions compared to the phenyl ring. In parallel, to examine the impact of further extending the central scaffold in the MOR binding site, we modified the core piperidine ring by enlarging it to a bicyclic 6,6-system, even larger than the eight-member ring in compound **VZFN342**. This structural extension introduces additional conformational rigidity and spatial elongation, potentially probing receptor binding site accommodating capacity. Furthermore, at the R2 position, a series of side chains with varying steric bulkiness and hydrophobicity (i.e., allyl, cyclopropylmethyl, cyclobutylmethyl, cyclopentylmethyl, cyclohexylmethyl, and benzyl) were introduced to investigate their influence on the physicochemical properties and modulation of the MOR function.

**Figure 2:**
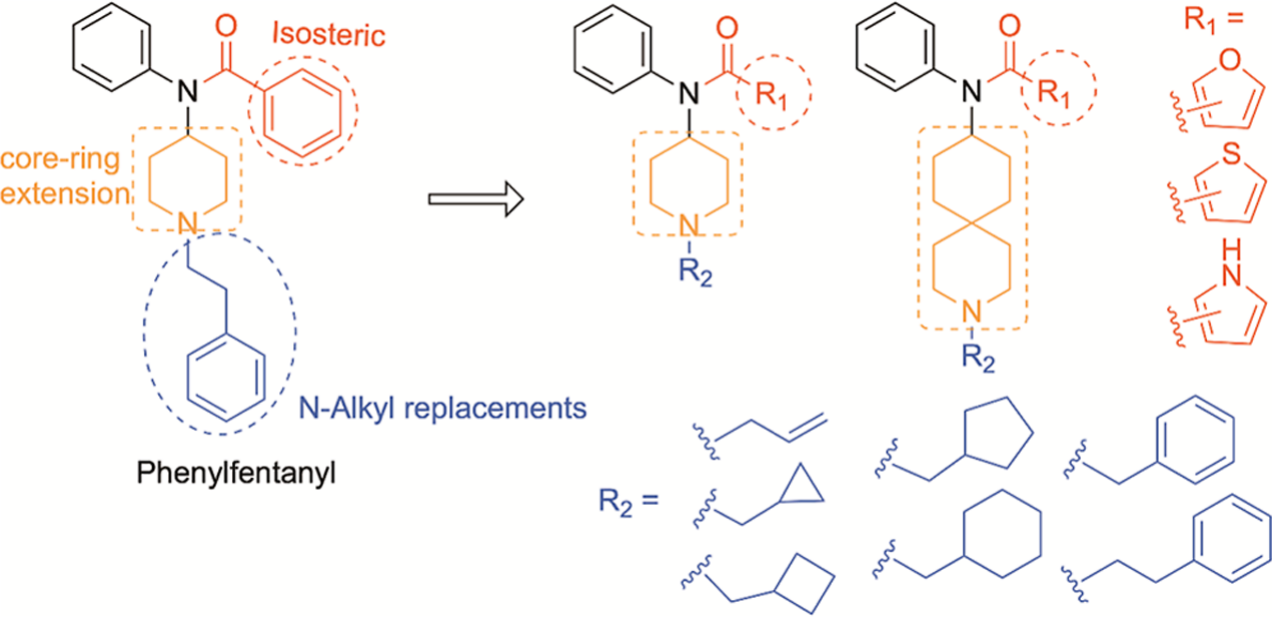
Molecular design for phenylfentanyl derivatives.

As depicted in Scheme 1, the starting material 1-Boc-4-piperidone (**a**) was coupled first with aniline to give the corresponding Schiff base, which was then reduced with sodium triacetoxyborohydride to yield the secondary amine **b**.^12^ Intermediate **b** was subsequently coupled with appropriate acyl chlorides in anhydrous DCM to obtain intermediates **c1**–**c6**.^13^ Boc protection was then removed using trifluoracetic acid, followed by coupling with various bulky *N*-alkyl side chains (phenylethyl was excluded in the 6-member ring series as being reported previously) under K_2_CO_3_ and acetonitrile.^10^ At this step, the reaction conditions varied in temperature: reactions with allyl, cyclopropylmethyl, and benzyl groups were carried out at room temperature while those with cyclobutylmethyl, cyclopentylmethyl, and cyclohexylmethyl were refluxed for 12 h. Purification by column chromatography was conducted to give the free bases as the products; then the free bases were converted to HCl salts (**1**–**36**). The 6,6-core ring series began with 3-Boc-9-oxo-3-azaspiro­[5,5]­undecane, following a similar procedure (phenylethyl was included in this series) to afford the final products as HCl salts (**37**–**78**). All synthesized compounds’ structures are shown in Table 1.

**Scheme 1:**
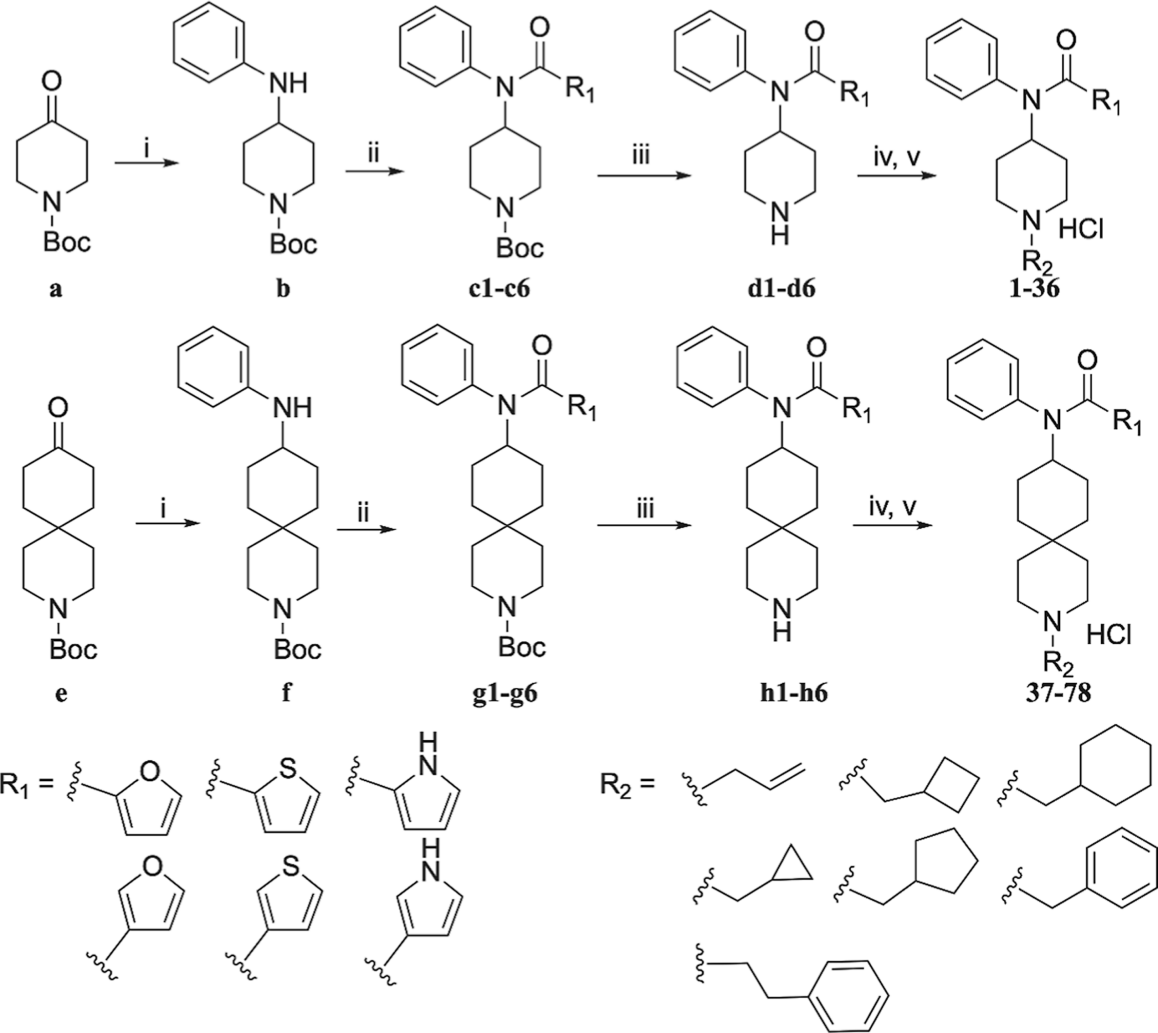
Synthesis of Target Compounds 1–78: (i) Aniline, AcOH, Na­(OAc)_3_BH, DCM, 0 °C-r.t: (ii) Acyl Chlorides, TEA, DCM, r.t: (iii) TFA, DCM, 0 °C-r.t. (iv) Alkyl Halides, K_2_CO_3_, ACN, Reflux: (v) 1.25 M HCl in MeOH, Diethyl Ether, 0 °C-r.t

**Table 1:**
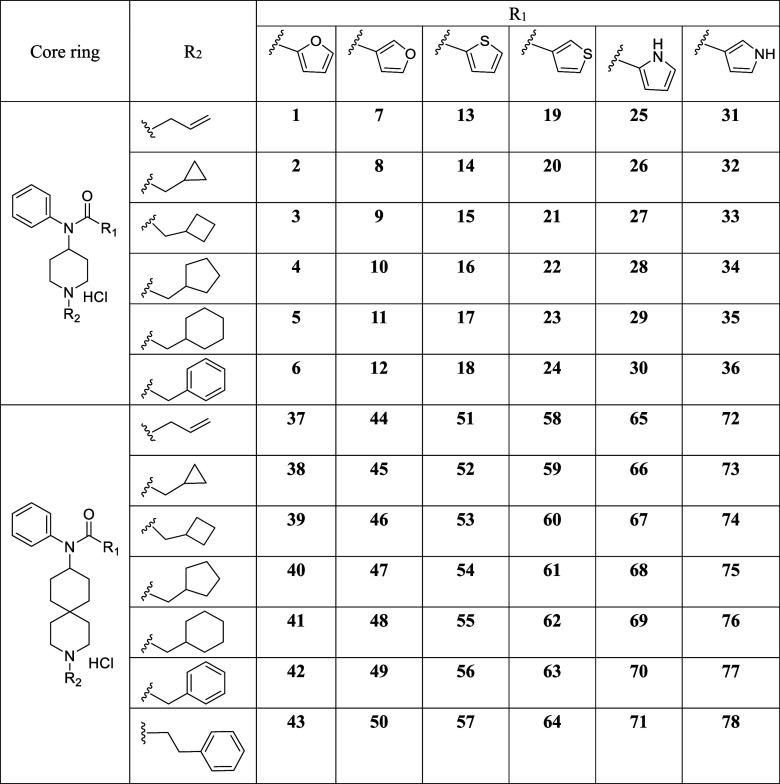
Structures of Synthesized Compound **1**–**78**

### Biological Studies

#### In Vivo Single-Dose Agonism Study

While in vitro assays are commonly used as an initial screening step in drug discovery, we employed a single-dose in vivo assay as a primary screening approach to rapidly assess the functional agonist activity of these phenylfentanyl derivatives. This method enables a rapid prioritization of compounds exhibiting agonistic or antagonistic properties, while concurrently allowing for the early detection of systemic side effects, toxicities, pharmacokinetic, or pharmacodynamic challenges that may be overlooked in simplified in vitro models. Follow-up in vitro studies will be conducted to elucidate the underlying cellular and molecular mechanisms once promising and desired in vivo function is observed. This approach was further necessitated by a nationwide shortage of radiolabeled ligands and the accelerated timeline required for this project. We have also recognized the safety and welfare of the animal subjects and have optimized our protocols in order to minimize the number of animal subjects involved in our studies.

Briefly, compounds that showed less than 35% maximum possible effect (MPE) in this initial screen would be selected for further evaluation as potential antagonists against morphine.^10,14^ As shown in Figure 3, 29 out of 36 6-membered core ring series compounds and all 6,6-core ring compounds did not exhibit significant antinociceptive effects, warranting single-dose antagonism testing to assess their potential opioid antagonistic activity. To be noted, during the in vivo evaluation, compounds **25**, **26**, **27**, and **28** produced acute toxicity, resulting in animal death within approximately 5 min after subcutaneous administration (s.c.) (10 mg/kg). As a result, no further pharmacological testing was conducted for these compounds.

**Figure 3:**
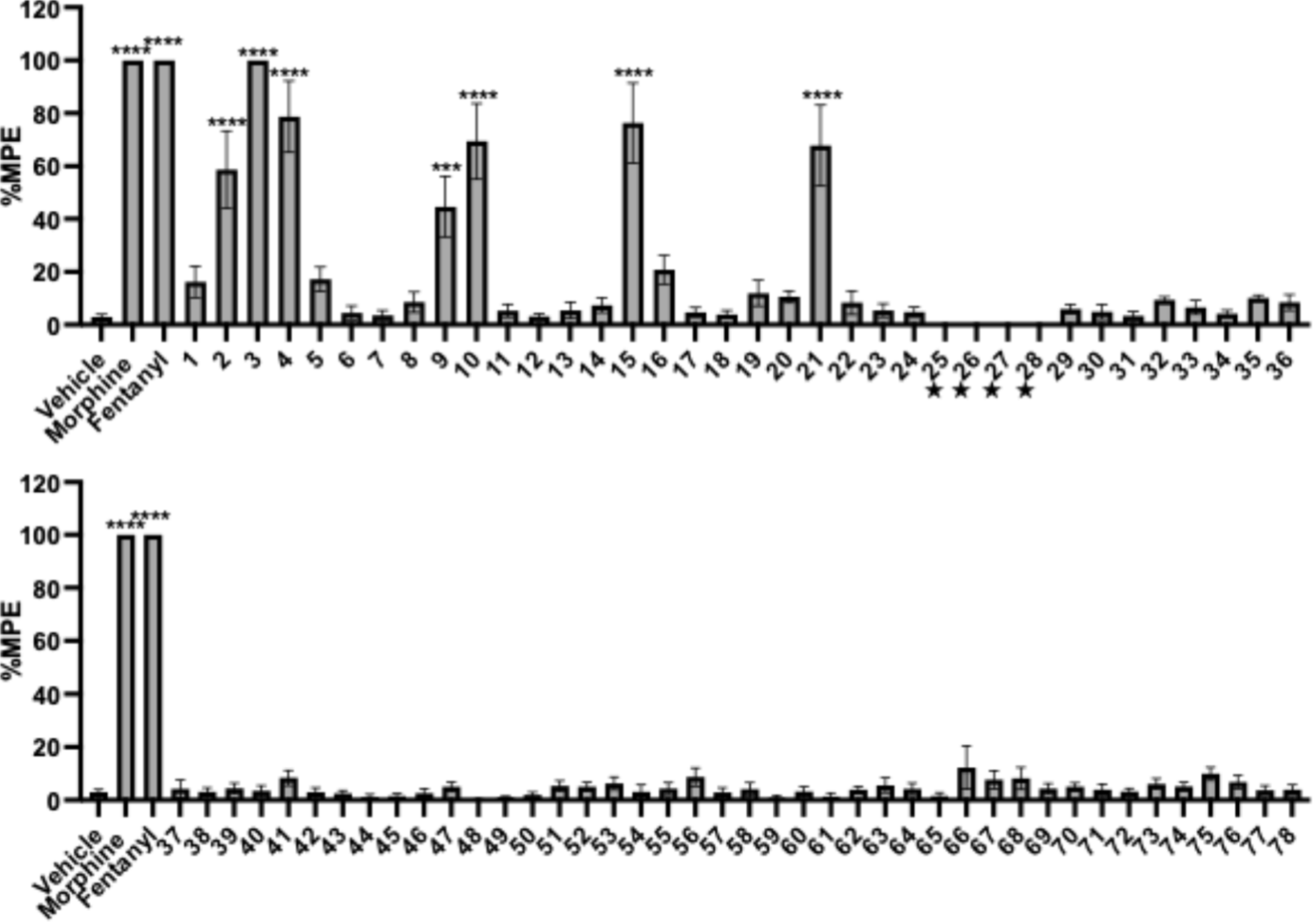
Warm-water tail immersion assay results of derivatives as agonists at a single dose of 10 mg/kg (s.c.). Saline was used as the negative control while morphine and fentanyl were used as positive controls. Data are presented as mean values ± SD ***P < 0.0005, ****P < 0.0001, compared to vehicle (s.c.).

#### In Vivo Single Dose Blockade of Morphine-Induced Antinociception

To further validate the antagonistic activity, we conducted an in vivo single dose assay against morphine following a previously reported protocol.^10^ Selected compounds (10 mg/kg) were coadministered with morphine (10 mg/kg). Compounds that reduced morphine-induced MPE by ≥ 60% were considered to exhibit antagonist activity and were selected for further evaluation. As shown in Figure 4, compounds **5**, **6**, **29**, **34**, and **36** demonstrated clear antagonist activity against morphine and were selected for further testing against fentanyl. Additionally, compounds **42**, **48**, **52**, **59**, **60, 63**, **65**, **67**, **72**, **73**, and **75** exhibited full or moderate inhibition effect in the morphine assay and were also advanced for evaluation of their inhibitory activity against fentanyl.

**Figure 4:**
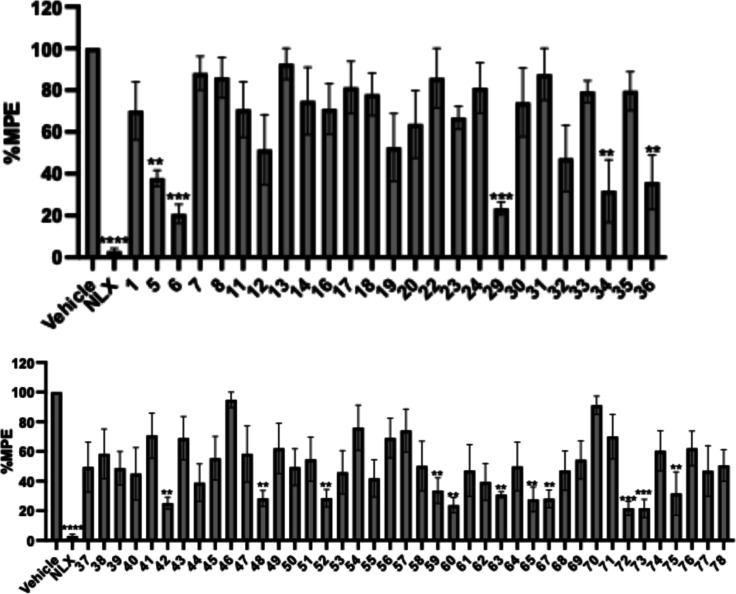
Warm-water tail immersion assay results of derivatives as antagonists at a single dose of 10 mg/kg (s.c.) in the presence of morphine (10 mg/kg, s.c.). Saline was used as the control and data are presented as mean values ± SD **P < 0.01, ***P < 0.0005, ****P < 0.0001, compared to vehicle (s.c.).

#### In Vivo Single Dose Blockade of Fentanyl-Induced Antinociception

Since the primary goal of this study was to identify antagonists effectively counteracting against fentanyl, compounds that showed antagonistic activity in the single dose morphine assay were subsequently tested in a fentanyl challenge. This progression enabled the prioritization of candidates with potential efficacy against fentanyl-induced effects while using morphine as an initial screening model due to its well-characterized pharmacological profile. Based on this rationale, selected compounds were evaluated in an in vivo single dose assay to access their ability to antagonize fentanyl, following protocol as previously reported.^10^ As shown in Figure 5, compound **5**, featuring a 2-furan acyl moiety coupled with a methyl cyclohexane alkyl chain, reduced fentanyl-induced effects to 40% MPE, suggesting effective opioid antagonism. Similarly, compound **34**, bearing a 3-pyrrole acyl group and a methyl cyclopentane side chain, showed moderate antagonism, reducing fentanyl MPE to approximately 60%. Among the 6,6-ring analogs, compound **42** (2-thiophene acyl paired with cyclobutylmethyl side chain) and compound **63** (3-thiophene acyl paired with a benzyl group) exhibited a comparable blocking effect to compound **34**. The moderate potency of compounds **42** and **63** suggests that scaffold extension to a 6,6-core ring system may retain opioid antagonist-like effects in certain cases. However, the reduced in vivo potency observed for most 6,6-ring derivatives may result from multiple factors, including suboptimal MOR binding affinity, or inadequate exposure at the tested dose, rendering these compounds less effective at the tested dose.

**Figure 5:**
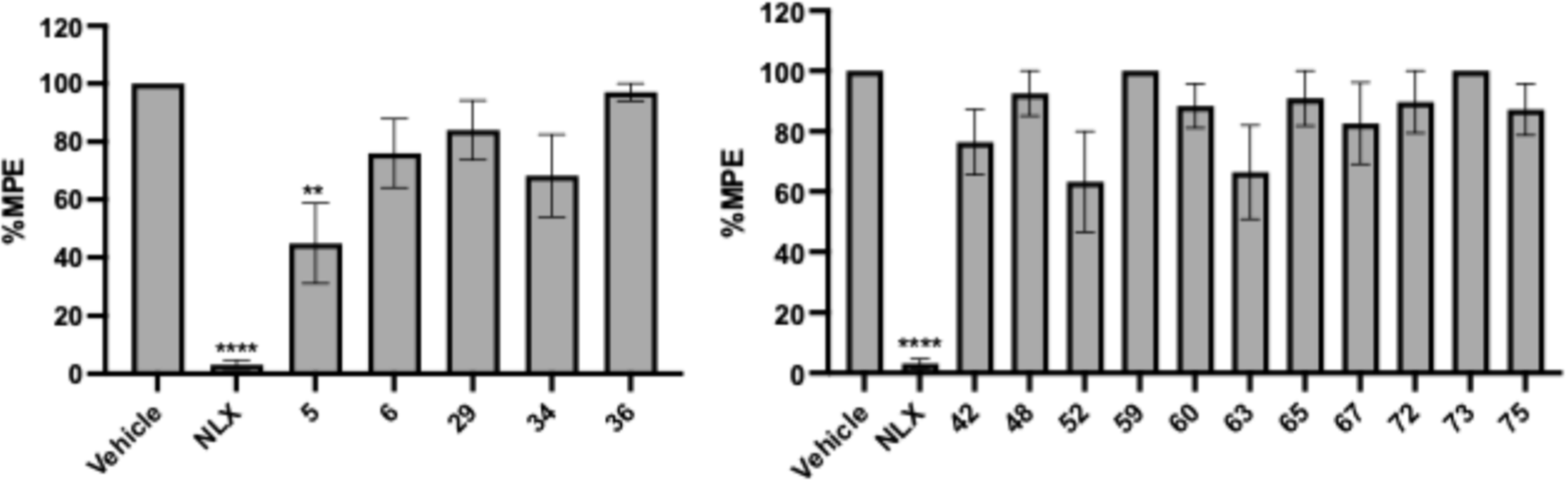
Warm-water tail immersion assay results of derivatives as antagonists at a single dose of 10 mg/kg (s.c.) in the presence of fentanyl (0.1 mg/kg, s.c.). Saline was used as the control and data are presented as mean values ± SD **P < 0.01, ****P < 0.0001, compared to vehicle (s.c.).

#### In Vivo Dose Response Antagonism Study

To further verify the opioid antagonist activity of compound **5**, dose response assays were conducted against both morphine and fentanyl.^10^ As shown in Figure 6, compound **5** exhibited moderate inhibitory effects, with AD_50_ values of 8.49 mg/kg for morphine and 10.70 mg/kg for fentanyl. While its potency is modest, the compound demonstrated a clear dose-dependent antagonism. As previously reported, phenylfentanyl was identified as an antagonist in vitro but showed only limited in vivo activity, exhibiting moderate antagonism of morphine and no significant effect counteracts the antinociceptive effects of fentanyl. In contrast, compound **5** represents a notable improvement from phenylfentanyl, demonstrating more effective antagonism in vivo. These results suggest that specific modifications on the acyl and alkyl side chains may significantly shift the functional outcome, supporting further development of this scaffold as a potential opioid antagonist.

**Figure 6:**
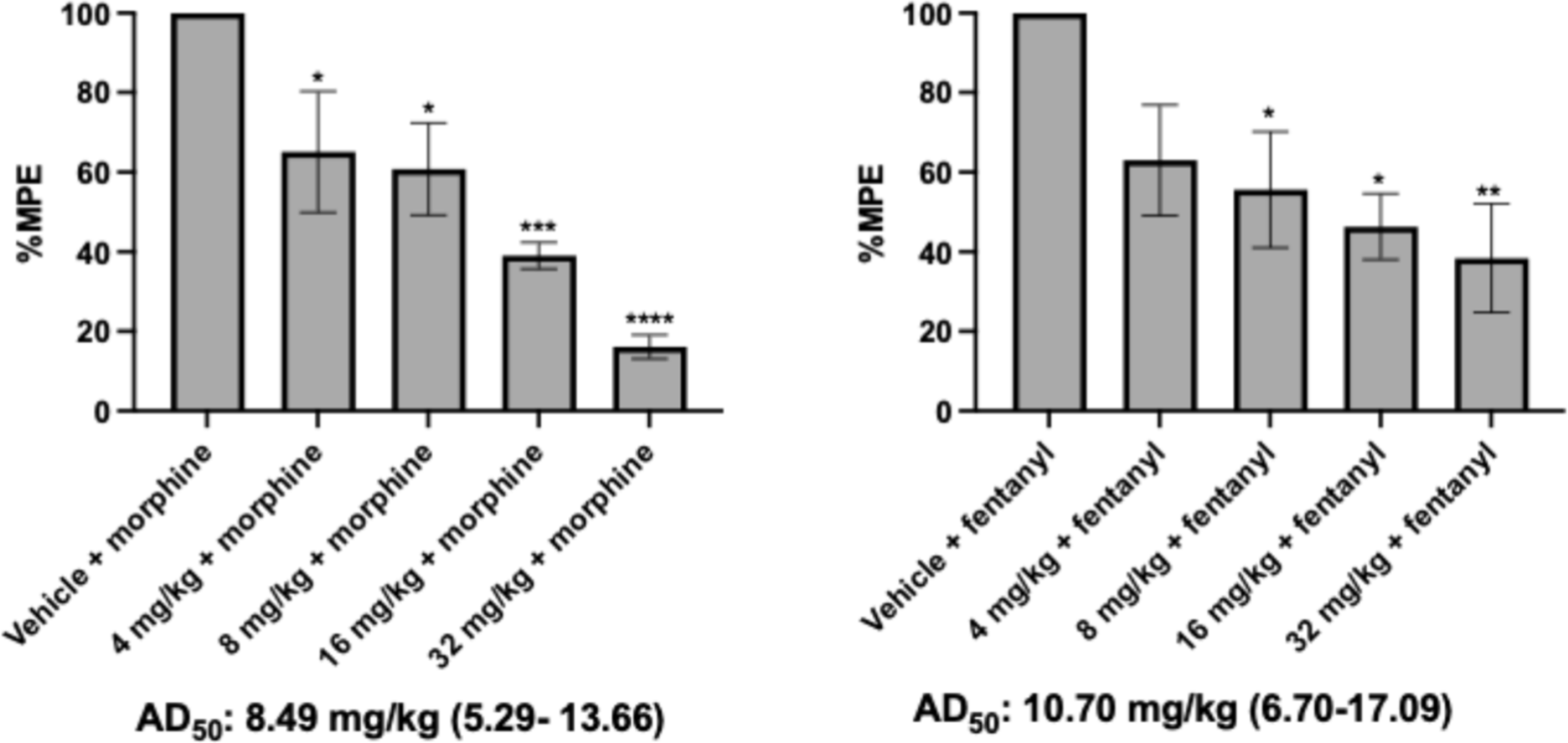
Dose response study of the most potent compound **5**, against (A) 10 mg/kg morphine (s.c.) and (B) 0.1 mg/kg fentanyl (s.c.). Data are presented as mean values ± SD *P < 0.05, **P < 0.01, ***P < 0.0005, ****P < 0.0001, compared to vehicle (s.c.).

#### In Vitro Calcium Flux Assay

To further elucidate the underlying mechanism of action of compound **5**, calcium mobilization assays were performed in MOR-expressing cells.^15^ As shown in Figure S1A, compound **5** did not show agonist activity, in contrast to the MOR agonist DAMGO (ED_50_ = 89.18 ± 10.18 nM). Meanwhile, as in Figure S1B,C, compound **5** functioned as a MOR antagonist, causing a rightward shift in the dose–response curve of both DAMGO and fentanyl. As summarized in Table 2, compound **5** exhibited ca. 6-fold and 10-fold greater antagonist potency than phenylfentanyl against DAMGO and fentanyl, respectively. In comparison with naltrexone (NTX), compound **5** was ca. 30-fold less potent than in antagonizing a DAMGO-induced response and ca. 300-fold less potent in antagonizing a fentanyl-induced response. Despite its lower potency relative to NTX, compound **5** demonstrates clear MOR antagonism and effective engagement of MOR signaling pathways.

**Table 2:** In Vitro Calcium Mobilization Study of Compound **5** against DAMGO and Fentanyl^a^

compds	NTX	phenylfentanyl^b^	compd. **5**	NTX/**5**	**5**/Ph
IC_50_, DAMGO (μM)	0.021 ± 0.015	4.1 ± 0.6	0.71 ± 0.19	33.8	5.8
IC_50_, fentanyl (μM)	0.007 ± 0.001	20.4 ± 1.3	2.08 ± 0.26	297	9.8

a Assays were performed in triplicate against DAMGO (500 nM) and fentanyl (250 nM).

b Data cited from ref 10 NTX—Naltrexone. Ph—Phenylfentanyl.

#### Molecular Modeling Studies

Molecular docking studies of compound **5** with the MOR were performed to rationalize the biological findings and provide mechanistic insights into the binding interactions at the MOR. Compound **5** was initially docked into the inactive MOR structure (PDB ID: 4DKL),^16^ with docking solutions evaluated and ranked using the CHEM-PLP scoring function, which considers both geometric complementarity and chemical interactions between the ligand and the receptor. The docking results demonstrated that phenylfentanyl and compound **5** exhibited similar binding modes with the MOR (Figures 7 and S3). The aniline ring and amide groups of both ligands were positioned within the orthosteric binding site of the receptor where they established critical hydrophobic interactions with residues Y148, W293, I296, H297, I322, and Y326 (Figure 7 and Table S1). These interactions have been identified as essential for MOR ligand binding at the orthosteric site through site-directed mutagenesis studies.^17–21^ Both ligands also maintained the characteristic ionic interaction through formation of a salt bridge between their protonated tertiary amine groups and the carboxylate group of D147. This electrostatic interaction represents a fundamental binding requirement for opioid receptor ligands and is crucial for maintaining the compounds in their bioactive conformations.^16,17^ The phenylethyl moiety of phenylfentanyl and the cyclohexyl methyl moiety of compounds **5** oriented upward the extracellular ends of transmembrane domain 2 (TM2), extracellular loop 1 (ECL1), and TM3. This binding orientation positioned both ligands to interact with key residues Q124, W133, V143, and I144 located at one of the subdomains of the allosteric site of the MOR (Figure S3).^21,22^ Notably, the phenylethyl group of phenylfentanyl extended more deeply into this hydrophobic pocket, facilitating π–π stacking and strong hydrophobic interactions with the protein (Figure 7A). In contrast, the cyclohexylmethyl substituent of compound **5**, while conformationally more flexible, did not penetrate as deeply into this pocket, resulting in weaker hydrophobic contacts within this binding domain (Figure 7B). These structural differences are consistent with altered interaction patterns within the receptor and provide a structural basis for the observed functional divergence between phenylfentanyl and compound **5** for future molecular design.

**Figure 7:**
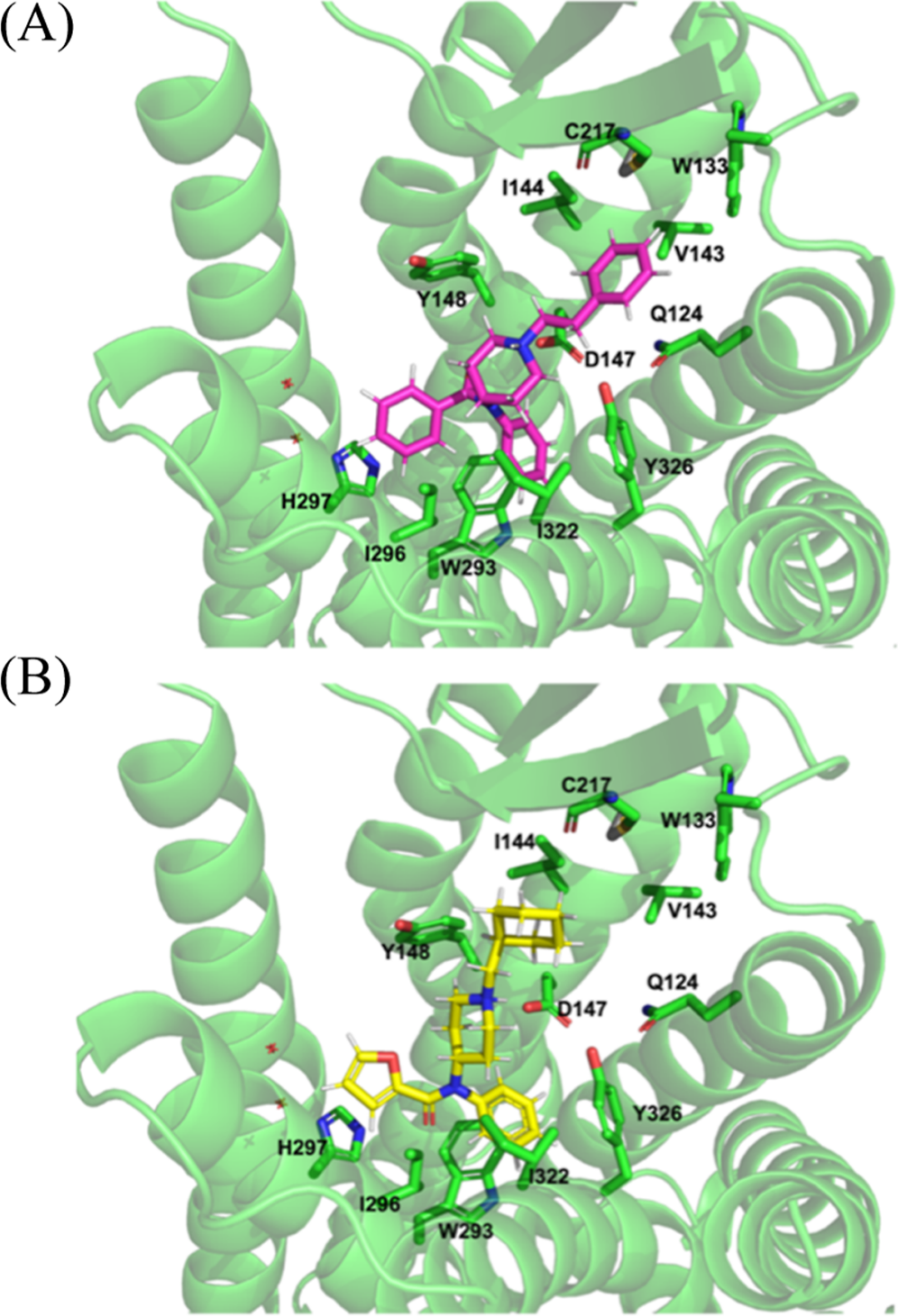
Binding pose of (A) phenylfentanyl and (B) compound **5** with the inactive MOR (PDB; 4DKL) from a docking study. MOR is shown as a cartoon. Phenylfentanyl (magenta sticks), compound **5** (yellow), and key amino acids (green sticks) are shown as stick models.

#### Metabolic Stability and Permeability Studies

To further characterize the drug-like properties of compound **5**, its metabolic stability and permeability were evaluated. Metabolic stability was evaluated in human liver S9 fractions following a standard protocol.^23^ To ensure enzymatic competence of the human and CD-1 mouse liver S9 fractions, four positive control compounds were included: clozapine (CYP1A2 and CYP3A4),^24^ diclofenac (CYP2C9),^25^ estrone (UGT enzymes and breast cancer resistance protein (BCRP) transporter),^26^ terfenadine (CYP3A4 and hERG inhibition).^27^ As showed in Table 3, compound **5** showed moderate metabolic stability in human liver S9 (*t*_1/2_ = 47.2 min; Clint = 14.8 μL/min/mg), while a comparably faster metabolism was observed in mouse liver S9 (*t*_1/2_ = 30.1 min; Clint = 23.5 μL/min/mg). Compared to the standard control compounds, compound **5** demonstrated moderate clearance. Notably, a species difference in metabolism (mouse vs human) should be taken into consideration for future development consideration.

**Table 3:** In Vitro Metabolism Profile of Compound **5**

compd	source	*t*_1/2_ (min)	clint (μL/min/mg)
**5**	human liver S9	47.2	14.8
clozapine		98.1	7.1
diclofenac		30.9	22.5
estrone		61.4	11.3
terfenadine		77.0	9.0
**5**	mouse liver S9 (CD-1)	30.1	23.5
clozapine		45.4	15.3
diclofenac		81.2	8.5
estrone		22.3	31.3
terfenadine		38.6	18.1

The permeability and efflux potential of compound **5** were assessed in Caco-2 bidirectional transport assays to evaluate its interaction with common efflux transporters, *P*-glycoprotein (*P*-gp) and BCRP.^28^ As shown in Table 4, the efflux ratios of compound **5** were 1.1 in both the *P*-gp and BCRP assays, indicating minimal susceptibility to transporter-mediated efflux. When coincubated with transporter-specific inhibitors (verapamil^29^ for *P*-gp and KO143 ^30^ for BCRP), the efflux ratios of compound **5** decreased slightly to 0.7 and 0.5, respectively. This suggests that compound **5** may not be a potent substrate of either *P*-gp or BCRP. For comparison, the known efflux substrates colchicine (*P*-gp) and estrone sulfate (BCRP) exhibited high efflux ratios (67.4 and 33.6, respectively), which were significantly reduced in the presence of inhibitors, confirming appropriate assay performance. Overall, these results suggested a favorable ADME profile for compound **5**.

**Table 4:** In Vitro Absorption Profile of Compound **5** on Caco-2 Cells

compd	substrate	efflux ratio	efflux ratio + inhibitor^a^
**5**	*P*-gp	1.1	0.7
colchicine		67.4	7.1
**5**	BCRP	1.1	0.5
estrone sulfate		33.6	2.6

a Verapamil used as a *P*-gp inhibitor, KO143 used as a BCRP inhibitor.

#### Time-Dependent BBB Penetration Study

An in vivo time-dependent BBB penetration study was performed to assess the CNS exposure of compound **5**.^31,32^ Following subcutaneous administration at 10 mg/kg, plasma and brain samples were collected at selected time points and analyzed by LC–MS/MS. As shown in Table 5, compound **5** was rapidly detected in plasma within 5 min and reached its maximal concentration at 10 min. Brain concentrations were measurable at 5 min and remained relatively stable over the 60 min study period (203, 406, 324, and 304 ng/g at 5, 10, 30, and 60 min, respectively). Although plasma levels declined after 10 min, brain concentrations were maintained, resulting in a progressive increase in the brain-to-plasma ratio, which peaked at 30 min (B/P = 2.64). Despite measurable brain exposure, the absolute CNS concentrations of compound **5** were substantially lower than those observed for the positive control naltrexone across all time points, indicating a comparatively limited central exposure.

**Table 5:** Time-Dependent BBB Penetration of Compound **5** (10 mg/kg s.c.) in Mice (*n* = 3, Mean ± SD)

compd	time (min)	brain (ng/g)	plasma (ng/mL)	brain/plasma ratio	
**5**	5	203 ± 81	188 ± 18	1.08	
	10	406 ± 45	316 ± 3	1.29	
	30	324 ± 23	123 ± 9	2.64	
	60	304 ± 92	156 ± 51	1.95	
NTX	5	1885 ± 1512	1406 ± 952	1.33	
	10	3533 ± 1150	1627 ± 286	2.17	
	30	3653 ± 1177	1018 ± 132	3.59	
	60	1353 ± 204	488 ± 121	2.77	

#### In Vivo Respiratory Depression Study

A leading factor contributing to fatalities in opioid overdoses is respiratory depression. The whole-body plethysmography (WBP) has routinely been used to evaluate opioid-induced respiratory depression.^33^ The main outcome measurements present as minute volume, which is the product of respiratory rate and tidal volume.

In our study, the respiration was recorded using WBP chambers supplied with an air mixture containing 5% CO_2_. A 20 min baseline respiration period was recorded prior to any compound administration. After the acclimatization period, 0.3 mg/kg fentanyl was administered subcutaneously, and respiration was recorded for 20 min. Following this, vehicle, or 10 mg/kg compound **5** was administered subcutaneously, and respiration was recorded for a period of 35 min. As shown in Figure 8, administration of compound **5** alone did not induce marked respiratory depression at the tested dose. While minor reductions in respiratory rate and tidal volume were observed relative to saline, the overall decrease in minute volume was modest and substantially less pronounced than that produced by fentanyl or phenylfentanyl, indicating a lack of intrinsic respiratory liability.

**Figure 8:**
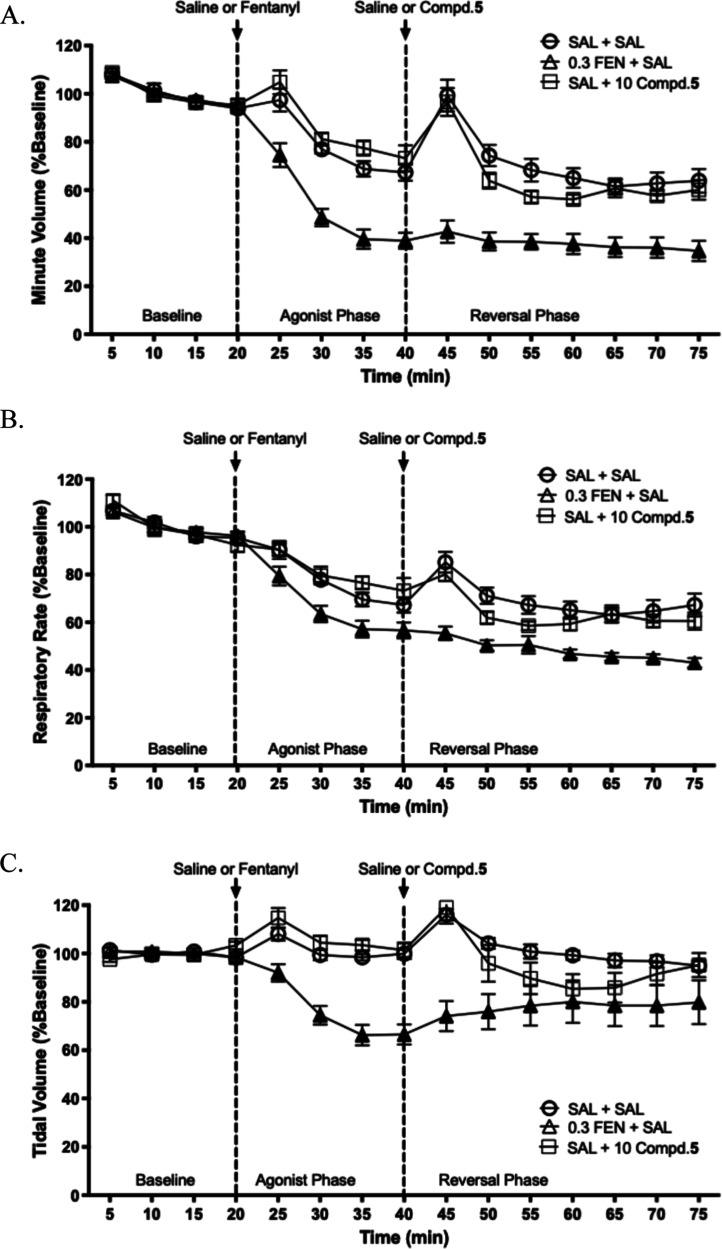
Effects of compd. **5** on ventilation in mice. (A) Minute volume; (B) respiratory rate; and (C) tidal volume. Error bars represent the standard error of normalized mean values within individual 5 min bins. Open symbols indicate no significant difference compared to the saline-treated controls (SAL + SAL) at individual time points (*p* > 0.05, one-way analysis of variance (ANOVA)). Closed symbols indicate a significant difference compared to the saline-treated controls (SAL + SAL) at individual time points (*p* ≤ 0.05, one-way ANOVA).

To evaluate whether compound **5** could reverse opioid-induced respiratory depression, an exploratory fentanyl challenge study was also conducted. Following administration of fentanyl (0.3 mg/kg, s.c.), compound **5** did not significantly reverse fentanyl-induced respiratory depression in vivo (Figure S4). This outcome is consistent with the reduced CNS exposure of compound 5 relative to naltrexone observed in the BBB penetration study. Together, these findings indicate that although compound **5** lacks sufficient central activity to reverse fentanyl-induced respiratory depression, it does not itself induce respiratory depression, supporting a favorable respiratory safety profile.

## Conclusion

In this study, a series of fentanyl-derived analogues featuring modifications to the core scaffold were designed to explore structure–activity relationships at the MOR and to identify antagonists with improved safety characteristics. While extension to a 6,6-core ring did not yield potent MOR antagonists, likely due to suboptimal spacing between key pharmacophoric elements, retention of the 6-membered core scaffold enabled identification of compound **5** as a lead structure. Incorporation of a 2-furanyl acyl substituent and a cyclohexylmethyl alkyl group proved favorable for MOR antagonism while differentiating its pharmacological profile from fentanyl and phenylfentanyl.

Compound **5** consistently demonstrated antagonist activity across multiple in vitro and in vivo assays, including single-dose and dose-dependent antagonism of morphine- and fentanyl-induced effects. In vitro calcium mobilization assays further confirmed MOR antagonism without detectable agonist activity and showed greater potency than phenylfentanyl in inhibiting fentanyl-induced signaling. Pharmacokinetic profiling revealed moderate metabolic stability and low efflux potential. Although compound **5** was detectable in the brain following systemic administration, its absolute CNS exposure was substantially lower than that of the reference antagonist naltrexone. Consistent with this exposure profile, compound **5** did not reverse fentanyl-induced respiratory depression in vivo. However, compound **5** did not itself induce marked respiratory depression at the tested dose. This dissociation between MOR antagonism and intrinsic respiratory liability distinguishes compound **5** from phenylfentanyl and highlights the feasibility of structurally tuning fentanyl-derived scaffolds to reduce respiratory risk while preserving antagonist function. Notably, while the previously reported 8-membered analogue demonstrated relatively stronger in vivo antagonist potency and was capable of reversing fentanyl-induced respiratory depression, compound **5** retains the original 6-membered fentanyl core yet lacks intrinsic respiratory depression while maintaining reasonably in vivo antagonist activity with even higher in vitro potency. This observation suggests that respiratory liability within the phenylfentanyl chemotype can be mitigated through targeted side-chain modifications even without extensive scaffold reorganization. Together, these findings indicate that core scaffold geometry, acyl substituent identity, and *N*-alkyl substitution represent key structural determinants governing MOR antagonist activity and respiratory liability within this phenylfentanyl-derived chemotype. Consistent with these observations, molecular docking studies suggest that tailored acyl and *N*-alkyl substitutions can modulate interactions within the inactive MOR binding pocket, providing a structural framework for future molecular design efforts. Collectively, these findings support the concept that fentanyl-based scaffolds can be rationally modified to decouple antagonist activity from respiratory depression, offering mechanistic guidance for the development of safer MOR antagonists.

## Materials and Methods

### Chemistry

#### General Experimental Procedures

All nonaqueous reactions were performed under a nitrogen atmosphere in predried glassware. All other solvents and reagents were purchased from Sigma-Aldrich, Alfa Aesar, Bepharm Scientific, and Fisher Scientific and were used as received without further purification. Analytical thin-layer chromatography (TLC) analyses were carried out on Analtech Uniplate F254 plates, and flash column chromatography (FCC) was performed over silica gel (230–400 mesh, Merck). The ^1^H (400 MHz) and ^13^C (100 MHz) nuclear magnetic resonance (NMR) spectra were recorded on a Bruker Ultrashield 400 Plus spectrometer, and chemical shifts were expressed in parts per million. The high-resolution mass spectra were obtained on an Applied BioSystems 3200 Q trap with a turbo V source for TurbolonSpray. Analytical reversed-phase high-performance liquid chromatography (HPLC) was performed on a Waters Arc HPLC system using an XBridge C_18_ 3.5 μm (4.6 × 50 mm). All analyses were conducted at ambient temperature with a flow rate of 0.8 or 1.5 mL/min. The mobile phase is acetonitrile/water (80:20 or 70:30) with 0.1% trifluoroacetic acid (TFA). The UV detector was set up at 210 nm. Compound purities were calculated as the percentage peak area of the analyzed compound, and retention times (*R*_t_) were presented in minutes. The purity of all newly synthesized compounds was identified as ≥95%.

#### General Procedure for the Synthesis of Phenylfentanyl Derivatives

The synthesis procedures for compounds **a** to **1**–**36** and **f** to **37**–**78** are analogous; therefore, only the synthesis of **a** to compounds **1**–**36** is descripted here as a representative example.

To a solution of **a** in anhydrous DCM, acetic acid was added, followed by aniline at 0 °C under a nitrogen atmosphere. The reaction mixture was stirred for 5 min, after which sodium triacetoxyborohydride was added slowly. The mixture was stirred at room temperature for an additional 16 h and then quenched by the addition of methanol. The organic layer was washed sequentially with water, saturated sodium bicarbonate (NaHCO_3_), and brine, dried over anhydrous sodium sulfate (Na_2_SO_4_), and purified by silica gel FCC using hexanes/ethyl acetate (10:1, v/v) to yield intermediate **b**.

Intermediate **b** was dissolved in anhydrous DCM and cooled to 0 °C. Triethylamine was added, followed by slow addition of an appropriate acyl chloride. The reaction mixture was stirred overnight at room temperature. The organic layer was washed with water and brine, dried over Na_2_SO_4_, and purified by FCC using hexanes/ethyl acetate (5:1, v/v) to afford intermediate **c**.

Intermediate **c** was dissolved in DCM, and trifluoroacetic acid (TFA) was added slowly at 0 °C. The mixture was stirred overnight and evaporated under reduced pressure to yield intermediate **d**, which was used in the next step without further purification.

Intermediate **d** was dissolved in anhydrous acetonitrile, followed by the addition of potassium carbonate (K_2_CO_3_) and an appropriate alkyl bromide. The reaction mixture was refluxed, and the progress was monitored by TLC. Upon completion, the mixture was cooled to room temperature, and the organic layer was washed with water and brine, dried over Na_2_SO_4_, and purified by FCC using DCM/MeOH/NH_3_·H_2_O (20:1:0.1, v/v/v) as the eluent to afford the target compounds. The resulting free bases were immediately converted to their HCl salt forms by treatment with HCl in methanol to obtain the final compounds.

#### *tert*-Butyl 4-(phenylamino)­piperidine-1-carboxylate (**b**)

The title compound was prepared following the general procedure as a white powder in 91% yield. ^1^H NMR (400 MHz, Chloroform-*d*): δ 7.26–7.07 (m, 2H), 6.72 (tt, *J* = 7.4, 1.1 Hz, 1H), 6.70–6.52 (m, 2H), 4.07 (d, *J* = 13.5 Hz, 2H), 3.51 (d, *J* = 17.7 Hz, 1H), 3.48–3.35 (m, 1H), 2.95 (td, *J* = 13.8, 11.2, 2.8 Hz, 2H), 2.11–1.98 (m, 2H), 1.50 (s, 9H), 1.33 (s, 2H). ^13^C NMR (100 MHz, Chloroform-*d*): δ 155.04, 147.31, 129.30, 117.06, 113.18, 79.27, 52.01, 34.21, 30.81, 29.44, 29.10. HRMS (ESI) *m*/*z*: [M + H]^+^ calcd for C_16_H_25_N_2_O_2_, 277.1916; found, 277.1897.

#### *tert*-Butyl 4-(*N*-phenylfuran-2-carboxamido)­piperidine-1-carboxylate **(c1)**, White Powder, Yield 91%

^1^H NMR (400 MHz, Chloroform-*d*): δ 7.43–7.30 (m, 3H), 7.26 (dd, *J* = 1.7, 0.8 Hz, 1H), 7.07 (dd, *J* = 7.7, 1.9 Hz, 2H), 6.07 (dd, *J* = 3.6, 1.8 Hz, 1H), 5.36 (d, *J* = 3.6 Hz, 1H), 4.84 (tt, *J* = 12.2, 3.8 Hz, 1H), 4.09 (s, 2H), 2.78 (t, *J* = 12.7 Hz, 2H), 1.88–1.72 (m, 2H), 1.33 (s, 9H), 1.25 (dd, *J* = 12.4, 4.5 Hz, 2H). ^13^C NMR (100 MHz, Chloroform-*d*): δ 158.86, 154.58, 147.05, 144.42, 138.47, 130.80, 129.33, 128.93, 116.12, 110.93, 79.62, 53.29, 30.26, 28.38. HRMS (ESI) *m*/*z*: [M + H]^+^ calcd for C_21_H_27_N_2_O_4_, 371.1971; found, 371.1951.

#### *tert*-Butyl 4-(*N*-phenylfuran-3-carboxamido)­piperidine-1-carboxylate **(c2)**, White Powder, Yield 90.6%

^1^H NMR (400 MHz, Chloroform-*d*): δ 7.35 (d, *J* = 7.0 Hz, 3H), 7.18–6.93 (m, 3H), 6.45 (s, 1H), 6.08 (s, 1H), 4.94–4.72 (m, 1H), 4.23–3.90 (m, 2H), 2.76 (s, 2H), 1.79 (d, *J* = 12.2 Hz, 2H), 1.32 (s, 12H). ^13^C NMR (100 MHz, Chloroform-*d*): δ 162.78, 154.59, 145.26, 141.86, 138.62, 131.00, 129.47, 129.11, 122.34, 111.34, 79.61, 53.24, 30.34, 28.37.

#### *tert*-Butyl 4-(*N*-phenylthiophene-2-carboxamido)­piperidine-1-carboxylate **(c3)**, White Powder, Yield 92%

^1^H NMR (400 MHz, Chloroform-*d*): δ 7.43–7.29 (m, 3H), 7.24–7.19 (m, 1H), 7.15–7.05 (m, 2H), 6.68 (dd, *J* = 5.0, 3.8 Hz, 1H), 6.53 (dd, *J* = 3.8, 1.2 Hz, 1H), 4.94–4.76 (m, 1H), 4.08 (dd, 2H), 2.77 (t, *J* = 13.0 Hz, 2H), 1.90–1.74 (dt, 2H), 1.35–1.24 (m, 11H). ^13^C NMR (100 MHz, Chloroform-*d*): δ 155.48, 147.12, 146.45, 129.32, 129.29, 118.51, 117.00, 115.11, 113.34, 79.34, 51.81, 47.36, 32.48, 31.35, 28.62, 24.75, 23.81.

#### *tert*-Butyl 4-(*N*-phenylthiophene-3-carboxamido)­piperidine-1-carboxylate **(c4)**, White Powder, Yield 64%

^1^H NMR (400 MHz, DMSO-*d*_6_): δ 7.36 (td, *J* = 4.6, 2.6 Hz, 3H), 7.28 (dd, *J* = 5.1, 3.0 Hz, 1H), 7.23–7.08 (m, 3H), 6.80 (dd, *J* = 5.0, 1.3 Hz, 1H), 4.69 (tt, *J* = 12.1, 3.8 Hz, 1H), 4.08–3.85 (m, 2H), 2.82 (s, 2H), 1.96–1.76 (m, 2H), 1.33 (s, 9H), 1.21 (td, *J* = 12.5, 4.6 Hz, 2H). ^13^C NMR (100 MHz, Chloroform-*d*): δ 155.48, 147.12, 146.45, 129.32, 129.29, 118.51, 117.00, 115.11, 113.34, 79.34, 51.81, 47.36, 47.08, 32.48, 31.35, 28.62, 24.75, 23.81. HRMS (ESI) *m*/*z*: [M + H]^+^ calcd for C_21_H_27_N_2_O_3_S, 387.1742; found, 387.1754.

#### *tert*-Butyl 4-(*N*-phenyl-1*H*-pyrrole-2-carboxamido)­piperidine-1-carboxylate **(c5)**, White Powder, Yield 74%

^1^H NMR (400 MHz, Chloroform-*d*): δ 9.49 (s, 1H), 7.40 (tdd, *J* = 9.0, 6.9, 2.0 Hz, 3H), 7.16–7.04 (m, 2H), 6.73 (td, *J* = 2.7, 1.3 Hz, 1H), 5.80 (dt, *J* = 3.8, 2.7 Hz, 1H), 4.85 (tt, *J* = 12.1, 3.8 Hz, 1H), 4.52 (ddd, *J* = 4.0, 2.4, 1.3 Hz, 1H), 4.08 (s, 2H), 2.77 (t, *J* = 12.9 Hz, 2H), 1.78 (ddd, *J* = 12.1, 4.6, 2.4 Hz, 2H), 1.34–1.24 (s, 11H). ^13^C NMR (100 MHz, Chloroform-*d*): δ 160.87, 154.59, 138.60, 131.19, 129.47, 129.07, 125.32, 120.82, 113.35, 109.83, 79.60, 53.10, 45.70, 30.39, 28.38, 8.58.

#### *tert*-Butyl 4-(*N*-phenyl-1*H*-pyrrole-3-carboxamido)­piperidine-1-carboxylate **(c6)**, White Powder in 44% Yield

^1^H NMR (400 MHz, Chloroform-*d*): δ 7.32 (q, *J* = 4.1, 1.9 Hz, 3H), 7.16–6.99 (m, 2H), 6.36 (q, *J* = 2.5 Hz, 1H), 6.27 (dt, *J* = 3.4, 1.8 Hz, 1H), 5.66 (td, *J* = 2.8, 1.5 Hz, 1H), 4.87 (tt, *J* = 12.1, 3.7 Hz, 1H), 4.06 (d, *J* = 11.9 Hz, 2H), 2.83–2.61 (m, 2H), 1.87–1.67 (m, 3H), 1.32 (s, 9H), 1.27–1.08 (m, 2H). ^13^C NMR (100 MHz, DMSO-*d*_6_): δ 170.81, 160.52, 154.35, 139.89, 131.47, 129.68, 129.02, 125.67, 121.58, 112.93, 108.96, 78.81, 60.22, 55.20, 34.90, 30.58, 28.55, 25.88, 21.23, 14.55. HRMS (ESI) *m*/*z*: [M + H]^+^ calcd for C_21_H_28_N_3_O_3_, 370.2131; found, 370.2091.

#### *tert*-Butyl 9-(phenylamino)-3-azaspiro[5.5]­undecane-3-carboxylate (**f**), White Powder, Yield 96%

^1^H NMR (400 MHz, DMSO-*d*_6_): δ 7.12–6.97 (m, 2H), 6.59–6.50 (m, 2H), 6.51–6.38 (m, 1H), 3.29 (dd, *J* = 8.5, 4.0 Hz, 4H), 1.82–1.60 (m, 4H), 1.41 (dd, *J* = 6.2, 3.9 Hz, 11H), 1.39 (s, 10H), 1.34–1.13 (m, 6H). ^13^C NMR (100 MHz, DMSO-*d*_6_): δ 154.45, 148.51, 129.34, 115.61, 112.80, 78.82, 51.21, 38.94, 34.10, 32.49, 30.95, 28.59, 27.64. HRMS (ESI) *m*/*z*: [M + H]^+^ calcd for C_21_H_33_N_2_O_2_, 345.2542; found, 345.2576.

#### *tert*-Butyl 9-(*N*-phenylfuran-2-carboxamido)-3-azaspiro­[5.5]­undecane-3-carboxylate **(g1)**, White Powder, Yield 89%

^1^H NMR (400 MHz, Chloroform-*d*): δ 7.49–7.37 (m, 3H), 7.31 (d, *J* = 1.7 Hz, 1H), 7.23–7.10 (m, 2H), 3.39–3.30 (m, 2H), 3.29–3.20 (m, 2H), 1.81–1.68 (m, 4H), 1.42 (s, 9H), 1.41–1.22 (m, 8H). ^13^C NMR (100 MHz, Chloroform-*d*): δ 158.79, 154.96, 147.37, 144.19, 139.22, 130.60, 129.22, 128.67, 115.78, 110.83, 79.21, 55.85, 39.89, 34.94, 30.99, 30.45, 28.46, 25.71. HRMS (ESI) *m*/*z*: [M + H]^+^ calcd for C_26_H_35_N_2_O_4_, 439.2597; found, 439.2597.

#### *tert*-Butyl 9-(*N*-phenylfuran-3-carboxamido)-3-azaspiro­[5.5]­undecane-3-carboxylate **(g2)**, White Powder, Yield 71%

^1^H NMR (400 MHz, Chloroform-*d*): δ 7.52–7.39 (m, 3H), 7.22–7.16 (m, 2H), 7.14 (p, *J* = 1.6 Hz, 1H), 6.60–6.46 (m, 1H), 6.18 (dd, *J* = 1.9, 0.8 Hz, 1H), 4.70 (tt, *J* = 11.9, 3.7 Hz, 1H), 3.40–3.31 (m, 2H), 3.31–3.21 (m, 2H), 1.83–1.69 (m, 4H), 1.44 (s, 9H), 1.40–1.23 (m, 8H). ^13^C NMR (100 MHz, Chloroform-*d*): δ 162.68, 154.96, 145.12, 141.75, 139.35, 130.82, 129.36, 128.87, 122.62, 111.37, 79.22, 55.77, 39.87, 34.98, 30.99, 30.46, 28.46, 25.80. HRMS (ESI) *m*/*z*: [M + H]^+^ calcd for C_26_H_35_N_2_O_4_, 439.2597; found, 439.2596.

#### *tert*-Butyl 9-(*N*-phenylthiophene-2-carboxamido)-3-azaspiro­[5.5]­undecane-3-carboxylate **(g3)**, White Powder, Yield 57%

^1^H NMR (400 MHz, Chloroform-*d*): δ 7.49–7.42 (m, 3H), 7.30–7.26 (m, 1H), 7.24–7.20 (m, 2H), 6.76 (dd, *J* = 5.1, 3.8 Hz, 1H), 6.58 (dd, *J* = 3.9, 1.2 Hz, 1H), 4.69 (m, 1H), 3.40–3.32 (m, 2H), 3.30–3.23 (m, 2H), 1.83–1.71 (m, 4H), 1.45 (s, 10H), 1.43–1.34 (m, 2H), 1.30 (m, 6H). ^13^C NMR (100 MHz, Chloroform-*d*): δ 161.97, 154.95, 139.33, 139.24, 131.81, 131.05, 130.62, 129.40, 128.93, 126.59, 56.64, 35.00, 31.00, 30.48, 28.46, 25.75. HRMS (ESI) *m*/*z*: [M + H]^+^ calcd for C_26_H_35_N_2_O_3_S, 455.2368; found, 455.2346.

#### *tert*-Butyl 9-(*N*-phenylthiophene-3-carboxamido)-3-azaspiro­[5.5]­undecane-3-carboxylate (**g4**), White Powder, Yield 59%

^1^H NMR (400 MHz, Chloroform-*d*): δ 7.27 (dt, *J* = 4.3, 1.4 Hz, 3H), 7.08–7.00 (m, 2H), 6.95–6.86 (m, 2H), 6.82 (dd, *J* = 4.9, 1.4 Hz, 1H), 4.59 (m, 1H), 3.33–3.24 (m, 2H), 3.23–3.14 (m, 2H), 1.68 (td, *J* = 12.1, 11.2, 3.5 Hz, 4H), 1.40 (d, *J* = 2.9 Hz, 1H), 1.36 (s, 10H), 1.32–1.18 (m, 6H). ^13^C NMR (100 MHz, Chloroform-*d*): δ 164.36, 154.97, 140.19, 137.58, 130.43, 129.18, 129.11, 128.77, 128.17, 123.99, 79.22, 56.46, 39.88, 35.06, 31.03, 30.50, 28.46, 25.88. HRMS (ESI) *m*/*z*: [M + H]^+^ calcd for C_26_H_35_N_2_O_3_S, 455.2368; found, 455.2347.

#### *tert*-Butyl-9-(*N*-phenyl-1*H*-pyrrole-2-carboxamido)-3-azaspiro­[5.5]­undecane-3-carboxylate **(g5)**, White Powder, Yield 55%

^1^H NMR (400 MHz, DMSO-*d*_6_): δ 11.34 (s, 1H), 7.48 (dd, *J* = 4.9, 2.0 Hz, 3H), 7.24 (dd, *J* = 6.7, 2.9 Hz, 2H), 6.73 (td, *J* = 2.6, 1.4 Hz, 1H), 5.72 (q, *J* = 2.8 Hz, 1H), 4.60–4.49 (m, 1H), 4.45 (s, 1H), 3.21 (dt, *J* = 36.6, 5.5 Hz, 4H), 1.76–1.54 (m, 4H), 1.36 (s, 9H), 1.29–1.06 (m, 8H). ^13^C NMR (100 MHz, DMSO-*d*_6_): δ 170.80, 160.52, 154.35, 139.89, 131.47, 129.68, 129.02, 125.66, 121.58, 112.92, 108.96, 78.80, 60.22, 55.20, 34.90, 30.58, 28.55, 25.88, 21.23, 14.55. HRMS (ESI) *m*/*z*: [M + H]^+^ calcd for C_26_H_36_N_3_O_3_, 438.2757; found, 438.2744.

#### *tert*-Butyl-9-(*N*-phenyl-1*H*-pyrrole-3-carboxamido)-3-azaspiro­[5.5]­undecane-3-carboxylate **(g6)**, White Powder Yield 65%

^1^H NMR (400 MHz, Chloroform-*d*): δ 8.60 (s, 1H), 7.32 (dd, *J* = 5.0, 1.9 Hz, 3H), 7.16–7.03 (m, 2H), 6.41–6.32 (m, 1H), 6.27 (m, 1H), 5.68–5.57 (m, 1H), 4.63 (m, 1H), 3.31–3.21 (m, 2H), 3.21–3.11 (m, 2H), 1.65 (td, *J* = 11.3, 10.7, 3.8 Hz, 4H), 1.36–1.17 (m, 17H). ^13^C NMR (100 MHz, Chloroform-*d*): δ 164.93, 155.02, 140.40, 131.03, 129.05, 128.21, 122.87, 119.94, 117.24, 110.47, 79.25, 55.62, 39.90, 35.06, 31.00, 30.47, 28.46, 25.93. HRMS (ESI) *m*/*z*: [M + H]^+^ calcd for C_26_H_36_N_3_O_3_, 438.2757; found, 438.2744.

#### *N*-(1-Allylpiperidin-4-yl)-*N*-phenylfuran-2-carboxamide Hydrochloride (**1**)

The title compound was prepared following the general procedure as a white powder in 94% yield. ^1^H NMR (400 MHz, DMSO-*d*_6_): δ 10.45 (s, 1H), 7.65 (d, *J* = 1.7 Hz, 1H), 7.50 (dd, *J* = 5.0, 1.8 Hz, 3H), 7.33–7.24 (m, 2H), 6.32 (dd, *J* = 3.5, 1.8 Hz, 1H), 6.01–5.86 (m, 1H), 5.52–5.39 (m, 3H), 4.80 (m, 1H), 3.63 (dd, *J* = 7.4, 3.5 Hz, 2H), 3.45–3.36 (m, 2H), 3.09 (q, *J* = 11.6 Hz, 2H), 2.04 (d, *J* = 13.2 Hz, 2H), 1.74 (qd, *J* = 13.2, 3.9 Hz, 2H). ^13^C NMR (100 MHz, DMSO-*d*_6_): δ 158.46, 146.96, 145.65, 138.30, 131.18, 129.93, 129.54, 127.94, 125.09, 116.33, 111.71, 58.23, 50.89, 50.63, 27.42. HRMS (ESI) *m*/*z*: [M + H]^+^ calcd for C_19_H_23_N_2_O_2_, 311.1760; found, 311.1754. HPLC: purity = 100%; *R*_t_ = 3.235 min.

#### *N*-(1-(Cyclopropylmethyl)­piperidin-4-yl)-*N*-phenylfuran-2-carboxamide Hydrochloride **(2)**

The title compound was prepared following the general procedure as a white powder in 82% yield. ^1^H NMR (400 MHz, DMSO-*d*_6_): δ 10.42 (s, 1H), 7.65 (d, *J* = 1.8 Hz, 1H), 7.55–7.46 (m, 3H), 7.29 (dd, *J* = 6.6, 2.9 Hz, 2H), 6.33 (dd, *J* = 3.6, 1.7 Hz, 1H), 5.43 (d, *J* = 3.6 Hz, 1H), 4.82 (m, 1H), 3.53 (d, *J* = 12.5 Hz, 2H), 3.14 (m, 2H), 2.88 (dd, *J* = 7.2, 5.1 Hz, 2H), 2.09–1.98 (m, 2H), 1.81 (qd, *J* = 13.2, 3.8 Hz, 2H), 1.15–1.02 (m, 1H), 0.65–0.54 (m, 2H), 0.36 (d, *J* = 5.2 Hz, 2H). ^13^C NMR (100 MHz, DMSO-*d*_6_): δ 158.47, 146.96, 145.64, 138.29, 131.18, 129.92, 129.53, 116.31, 111.71, 60.36, 50.90, 50.79, 27.36, 5.78, 4.59. HRMS (ESI) *m*/*z*: [M + H]^+^ calcd for C_20_H_25_N_2_O_2_, 325.1916; found, 325.1911. HPLC: purity = 100%; *R*_t_ = 4.102 min.

#### *N*-(1-(Cyclobutylmethyl)­piperidin-4-yl)-*N*-phenylfuran-2-carboxamide Hydrochloride **(3)**

The title compound was prepared following the general procedure as a white powder in 83% yield. ^1^H NMR (400 MHz, DMSO-*d*_6_): δ 9.97 (s, 1H), 7.64 (d, *J* = 1.7 Hz, 1H), 7.56–7.45 (m, 3H), 7.29 (dd, *J* = 6.6, 2.9 Hz, 2H), 6.32 (dd, *J* = 3.6, 1.7 Hz, 1H), 5.44 (d, *J* = 3.6 Hz, 1H), 4.80 (m, 1H), 3.40–3.33 (m, 2H), 3.11 (td, *J* = 11.2, 9.8, 2.7 Hz, 2H), 3.01 (dd, *J* = 7.0, 5.1 Hz, 2H), 2.72 (p, *J* = 7.5 Hz, 1H), 2.03 (m, 4H), 1.90–1.82 (m, 1H), 1.76 (tt, *J* = 10.9, 5.4 Hz, 5H). ^13^C NMR (100 MHz, DMSO-*d*_6_): δ 158.45, 146.97, 145.64, 138.31, 131.23, 129.91, 129.52, 116.31, 111.70, 61.08, 51.26, 50.52, 30.41, 27.36, 27.16, 18.50. HRMS (ESI) *m*/*z*: [M + H]^+^ calcd for C_21_H_27_N_2_O_2_, 339.2073; found, 339.2082. HPLC: purity = 100%; *R*_t_ = 3.705 min.

#### *N*-(1-(Cyclopentylmethyl)­piperidin-4-yl)-*N*-phenylfuran-2-carboxamide Hydrochloride **(4)**

The title compound was prepared following the general procedure as a white powder in 84% yield. ^1^H NMR (400 MHz, DMSO-*d*_6_): δ 9.59 (s, 1H), 7.65 (d, *J* = 1.7 Hz, 1H), 7.50 (q, *J* = 3.0 Hz, 3H), 7.34–7.24 (m, 2H), 6.32 (dd, *J* = 3.6, 1.7 Hz, 1H), 5.43 (d, *J* = 3.5 Hz, 1H), 4.82 (tt, *J* = 12.2, 3.8 Hz, 1H), 3.49 (d, *J* = 12.1 Hz, 2H), 3.12 (dt, *J* = 12.8, 9.9 Hz, 2H), 2.97 (dd, *J* = 7.2, 5.4 Hz, 2H), 2.20 (m, *J* = 7.7 Hz, 1H), 2.00 (d, *J* = 12.0 Hz, 2H), 1.80 (qt, *J* = 11.0, 4.7 Hz, 4H), 1.63–1.41 (m, 4H), 1.25–1.13 (m, 2H). ^13^C NMR (100 MHz, DMSO-*d*_6_): δ 158.46, 146.97, 145.64, 138.31, 131.29, 129.92, 129.52, 116.31, 111.70, 61.41, 51.79, 50.56, 34.86, 31.22, 27.26, 25.08. HRMS (ESI) *m*/*z*: [M + H]^+^ calcd for C_22_H_29_N_2_O_2_, 353.2229; found, 353.2220. HPLC: purity = 100%; *R*_t_ = 2.427 min.

#### *N*-(1-(Cyclohexylmethyl)­piperidin-4-yl)-*N*-phenylfuran-2-carboxamide Hydrochloride **(5)**

The title compound was prepared following the general procedure as a white powder in 55% yield. ^1^H NMR (400 MHz, DMSO-*d*_6_): δ 9.17 (s, 1H), 7.64 (d, *J* = 2.1 Hz, 1H), 7.54–7.46 (m, 3H), 7.34–7.26 (m, 2H), 6.32 (dd, *J* = 3.6, 1.8 Hz, 1H), 5.45 (d, *J* = 3.5 Hz, 1H), 4.82 (m, 1H), 3.53–3.43 (m, 2H), 3.11 (dt, *J* = 13.6, 10.8 Hz, 2H), 2.83 (t, *J* = 6.0 Hz, 2H), 2.00 (d, *J* = 13.4 Hz, 2H), 1.88–1.54 (m, 8H), 1.28–1.05 (m, 3H), 0.98–0.83 (m, 2H). ^13^C NMR (100 MHz, DMSO-*d*_6_): δ 158.48, 146.94, 145.66, 138.29, 131.32, 129.93, 129.54, 116.35, 111.72, 62.38, 52.11, 50.48, 32.58, 30.74, 27.19, 25.88, 25.33. HRMS (ESI) *m*/*z*: [M + H]^+^ calcd for C_23_H_31_N_2_O_2_, 367.2386; found, 367.2395. HPLC: purity = 98.79%; *R*_t_ = 3.532 min.

#### *N*-(1-Benzylpiperidin-4-yl)-*N*-phenylfuran-2-carboxamide Hydrochloride **(6)**

The title compound was prepared following the general procedure as a white powder in 95% yield. ^1^H NMR (400 MHz, DMSO-*d*_6_): δ 10.43 (s, 1H), 7.64 (d, *J* = 1.7 Hz, 1H), 7.56 (dd, *J* = 6.7, 2.9 Hz, 2H), 7.48 (dd, *J* = 5.0, 1.9 Hz, 3H), 7.46–7.40 (m, 3H), 7.30–7.20 (m, 2H), 6.31 (dd, *J* = 3.5, 1.7 Hz, 1H), 5.44 (d, *J* = 3.6 Hz, 1H), 4.79 (tt, *J* = 12.2, 3.8 Hz, 1H), 4.22 (d, *J* = 5.3 Hz, 2H), 3.31–3.27 (m, 1H), 3.15 (dt, *J* = 12.9, 9.9 Hz, 2H), 2.01 (d, *J* = 13.3 Hz, 2H), 1.77 (qd, *J* = 13.2, 3.9 Hz, 2H). ^13^C NMR (100 MHz, DMSO-*d*_6_): δ 158.49, 146.91, 145.66, 138.25, 131.75, 131.18, 130.21, 129.96, 129.92, 129.52, 129.23, 116.36, 111.71, 59.43, 51.14, 50.66, 27.22. HRMS (ESI) *m*/*z*: [M + H]^+^ calcd for C_23_H_25_N_2_O_2_, 361.1916; found, 361.1925. HPLC: purity = 100%; *R*_t_ = 2.213 min.

#### *N*-(1-Allylpiperidin-4-yl)-*N*-phenylfuran-3-carboxamide Hydrochloride **(7)**

The title compound was prepared following the general procedure as a white powder in 54% yield. ^1^H NMR (400 MHz, DMSO-*d*_6_): δ 10.38 (s, 1H), 7.58–7.44 (m, 4H), 7.34–7.24 (m, 2H), 6.78 (s, 1H), 6.00 (d, *J* = 1.9 Hz, 1H), 5.97–5.85 (m, 1H), 5.53–5.41 (m, 2H), 4.82 (m, 1H), 3.64 (dd, *J* = 7.0, 4.9 Hz, 2H), 3.41 (s, 1H), 3.16–3.02 (m, 2H), 2.09–1.99 (m, 2H), 1.72 (qd, *J* = 13.2, 4.0 Hz, 2H). ^13^C NMR (100 MHz, DMSO-*d*_6_): δ 162.44, 145.48, 143.31, 138.40, 131.45, 130.00, 129.63, 127.92, 125.14, 122.57, 111.24, 58.23, 50.95, 50.42, 27.50. HRMS (ESI) *m*/*z*: [M + H]^+^ calcd for C_19_H_23_N_2_O_2_, 311.1760; found, 311.1758. HPLC: purity = 96.21%; *R*_t_ = 2.292 min.

#### *N*-(1-(Cyclopropylmethyl)­piperidin-4-yl)-*N*-phenylfuran-3-carboxamide Hydrochloride **(8)**

The title compound was prepared following the general procedure as a white powder in 100% yield. ^1^H NMR (400 MHz, DMSO-*d*_6_): δ 10.36 (s, 1H), 7.56–7.42 (m, 4H), 7.34–7.23 (m, 2H), 6.77 (s, 1H), 6.01 (d, *J* = 1.9 Hz, 1H), 4.82 (tt, *J* = 12.2, 3.7 Hz, 1H), 3.63–3.44 (m, 2H), 3.21–3.03 (m, 2H), 2.88 (dd, *J* = 7.2, 5.1 Hz, 2H), 2.12–1.93 (m, 2H), 1.80 (qd, *J* = 13.2, 4.0 Hz, 2H), 1.09 (m, 1H), 0.64–0.52 (m, 2H), 0.35 (dt, *J* = 6.3, 4.4 Hz, 2H). ^13^C NMR (100 MHz, DMSO-*d*_6_): δ 162.43, 145.47, 143.31, 138.41, 131.45, 129.98, 129.61, 122.60, 111.25, 60.37, 50.94, 50.59, 27.43, 5.79, 4.59. HRMS (ESI) *m*/*z*: [M + H]^+^ calcd for C_20_H_25_N_2_O_2_, 325.1916; found, 325.1917. HPLC: purity = 99.88%; *R*_t_ = 2.907 min.

#### *N*-(1-(Cyclobutylmethyl)­piperidin-4-yl)-*N*-phenylfuran-3-carboxamide Hydrochloride **(9)**

The title compound was prepared following the general procedure as a white powder in 95% yield. ^1^H NMR (400 MHz, DMSO-*d*_6_): δ 9.97 (s, 1H), 7.57–7.42 (m, 4H), 7.34–7.23 (m, 2H), 6.77 (s, 1H), 6.00 (d, *J* = 1.9 Hz, 1H), 4.81 (m, 1H), 3.36 (s, 1H), 3.10 (td, *J* = 11.1, 9.9, 2.8 Hz, 2H), 3.01 (dd, *J* = 7.0, 5.1 Hz, 2H), 2.72 (p, *J* = 7.4 Hz, 1H), 2.13–1.94 (m, 4H), 1.92–1.65 (m, 6H). ^13^C NMR (100 MHz, DMSO-*d*_6_): δ 162.44, 145.48, 143.31, 138.39, 131.48, 130.00, 129.62, 122.58, 111.24, 61.08, 51.31, 50.32, 30.41, 27.44, 27.13, 18.49. HRMS (ESI) *m*/*z*: [M + H]^+^ calcd for C_21_H_27_N_2_O_2_, 339.2073; found, 339.2080. HPLC: purity = 100%; *R*_t_ = 2.117 min.

#### *N*-(1-(Cyclopentylmethyl)­piperidin-4-yl)-*N*-phenylfuran-3-carboxamide Hydrochloride **(10)**

The title compound was prepared following the general procedure as a white powder in 74% yield. ^1^H NMR (400 MHz, DMSO-*d*_6_): δ 9.60 (s, 1H), 7.55–7.44 (m, 4H), 7.33–7.23 (m, 2H), 6.77 (s, 1H), 6.00 (d, *J* = 2.0 Hz, 1H), 4.83 (tt, *J* = 12.3, 3.9 Hz, 1H), 3.49 (d, *J* = 12.2 Hz, 2H), 3.11 (dt, *J* = 12.9, 9.9 Hz, 2H), 2.97 (dd, *J* = 7.1, 5.4 Hz, 2H), 2.20 (p, *J* = 7.7 Hz, 1H), 2.00 (d, *J* = 11.3 Hz, 2H), 1.87–1.71 (m, 4H), 1.64–1.42 (m, 4H), 1.25–1.12 (m, 2H). ^13^C NMR (100 MHz, DMSO-*d*_6_): δ 162.47, 145.48, 143.31, 138.39, 131.53, 130.01, 129.63, 122.57, 111.25, 61.43, 51.85, 50.35, 34.86, 31.15, 27.34, 25.07. HRMS (ESI) *m*/*z*: [M + H]^+^ calcd for C_22_H_29_N_2_O_2_, 353.2229; found, 353.2216. HPLC: purity = 100%; *R*_t_ = 2.845 min.

#### *N*-(1-(Cyclohexylmethyl)­piperidin-4-yl)-*N*-phenylfuran-3-carboxamide Hydrochloride **(11)**

The title compound was prepared following the general procedure as a white powder in 91% yield. ^1^H NMR (400 MHz, DMSO-*d*_6_): δ 9.04 (s, 1H), 7.55–7.44 (m, 4H), 7.32–7.24 (m, 2H), 6.78 (s, 1H), 6.00 (d, *J* = 1.9 Hz, 1H), 4.83 (m, 1H), 3.49 (d, *J* = 12.1 Hz, 2H), 3.10 (dt, *J* = 13.6, 10.8 Hz, 2H), 2.83 (t, *J* = 6.0 Hz, 2H), 2.00 (d, *J* = 12.6 Hz, 2H), 1.85–1.54 (m, 9H), 1.26–1.06 (m, 3H), 0.89 (q, *J* = 11.9 Hz, 2H). ^13^C NMR (100 MHz, DMSO-*d*_6_): δ 162.45, 145.48, 143.31, 138.39, 131.58, 130.00, 129.62, 122.56, 111.25, 62.37, 52.14, 50.26, 32.58, 30.72, 27.27, 25.88, 25.33. HRMS (ESI) *m*/*z*: [M + H]^+^ calcd for C_23_H_31_N_2_O_2_, 367.2386; found, 367.2380. HPLC: purity = 100%; *R*_t_ = 4.202 min.

#### *N*-(1-Benzylpiperidin-4-yl)-*N*-phenylfuran-3-carboxamide Hydrochloride **(12)**

The title compound was prepared following the general procedure as a white powder in 100% yield. ^1^H NMR (400 MHz, DMSO-*d*_6_): δ 10.38 (s, 1H), 7.59–7.51 (m, 2H), 7.51–7.45 (m, 4H), 7.43 (m, *J* = 3.5 Hz, 3H), 7.30–7.20 (m, 2H), 6.77 (s, 1H), 5.99 (d, *J* = 1.9 Hz, 1H), 4.81 (m, 1H), 4.22 (d, *J* = 5.3 Hz, 2H), 3.34–3.25 (m, 2H), 3.21–3.08 (m, 2H), 2.07–1.95 (m, 2H), 1.76 (qd, *J* = 13.2, 4.0 Hz, 2H). ^13^C NMR (100 MHz, DMSO-*d*_6_): δ 162.43, 145.48, 143.31, 138.36, 131.75, 131.44, 130.26, 129.98, 129.94, 129.60, 129.22, 122.55, 111.23, 59.38, 51.13, 50.48, 27.29. HRMS (ESI) *m*/*z*: [M + H]^+^ calcd for C_23_H_25_N_2_O_2_, 361.1916; found, 361.1912. HPLC: purity = 100%; *R*_t_ = 2.640 min.

#### *N*-(1-Allylpiperidin-4-yl)-*N*-phenylthiophene-2-carboxamide Hydrochloride **(13)**

The title compound was prepared following the general procedure as a white solid in 88% yield. ^1^H NMR (400 MHz, DMSO-*d*_6_): δ 9.84 (s, 1H), 7.62 (dd, *J* = 5.0, 1.2 Hz, 1H), 7.60–7.41 (m, 3H), 7.34 (dd, *J* = 6.6, 2.9 Hz, 2H), 6.84 (dd, *J* = 5.1, 3.8 Hz, 1H), 6.44 (d, *J* = 3.8 Hz, 1H), 5.89 (d, *J* = 6.4 Hz, 1H), 5.54–5.40 (m, 2H), 4.94–4.72 (m, 1H), 3.65 (t, *J* = 6.1 Hz, 2H), 3.42 (d, *J* = 12.2 Hz, 2H), 3.10 (d, *J* = 11.7 Hz, 2H), 2.09 (d, *J* = 13.3 Hz, 2H), 1.68 (d, *J* = 13.2 Hz, 2H). ^13^C NMR (100 MHz, DMSO-*d*_6_): δ 161.61, 138.85, 138.32, 132.30, 132.03, 131.64, 130.09, 129.85, 128.03, 127.47, 125.02, 58.21, 51.21, 50.87, 27.45. HRMS (ESI) *m*/*z*: [M + H]^+^ calcd for C_19_H_23_N_2_OS, 327.1531; found, 327.1525. HPLC: purity = 98.65%; *R*_t_ = 3.913 min.

#### *N*-(1-(Cyclopropylmethyl)­piperidin-4-yl)-*N*-phenylthiophene-2-carboxamide Carboxamide Hydrochloride **(14)**

The title compound was prepared following the general procedure as a white solid in 94% yield. ^1^H NMR (400 MHz, DMSO-*d*_6_): δ 7.62 (dd, *J* = 5.0, 1.2 Hz, 1H), 7.60–7.43 (m, 3H), 7.35 (q, *J* = 3.9, 3.1 Hz, 2H), 6.84 (dd, *J* = 5.1, 3.8 Hz, 1H), 6.45 (d, *J* = 3.8 Hz, 1H), 4.84 (tt, *J* = 12.4, 3.9 Hz, 1H), 3.55 (d, *J* = 12.2 Hz, 2H), 3.13 (q, *J* = 11.7 Hz, 2H), 2.90 (t, *J* = 6.1 Hz, 2H), 2.08 (d, *J* = 13.4 Hz, 2H), 1.72 (d, *J* = 15.0 Hz, 2H), 1.12–0.96 (m, 1H), 0.68–0.55 (m, 2H), 0.42–0.28 (m, 2H). ^13^C NMR (100 MHz, DMSO-*d*_6_): δ 161.61, 138.85, 138.32, 132.30, 132.03, 131.64, 130.09, 129.85, 128.03, 127.47, 125.02, 58.21, 51.21, 50.87, 27.45. HRMS (ESI) *m*/*z*: [M + H]^+^ calcd for C_20_H_25_N_2_OS, 341.1688; found, 341.1682. HPLC: purity = 100%; *R*_t_ = 2.122 min.

#### *N*-(1-(Cyclobutylmethyl)­piperidin-4-yl)-*N*-phenylthiophene-2-carboxamide Hydrochloride **(15)**

The title compound was prepared following the general procedure as a white solid in 92% yield. ^1^H NMR (400 MHz, DMSO-*d*_6_): δ 9.28 (s, 1H), 7.61 (dd, *J* = 5.0, 1.2 Hz, 1H), 7.52 (dd, *J* = 5.0, 1.9 Hz, 3H), 7.40–7.27 (m, 2H), 6.84 (dd, *J* = 5.1, 3.8 Hz, 1H), 6.44 (d, *J* = 3.6 Hz, 1H), 4.90–4.72 (m, 1H), 3.38 (d, *J* = 11.9 Hz, 2H), 3.21–2.93 (m, 4H), 2.67 (s, 1H), 2.12–1.98 (m, 4H), 1.95–1.58 (m, 6H). ^13^C NMR (100 MHz, DMSO-*d*_6_): δ 161.59, 138.86, 138.32, 132.29, 132.03, 131.69, 130.09, 129.84, 127.46, 61.05, 51.26, 51.08, 30.42, 27.39, 27.18, 18.51. HRMS (ESI) *m*/*z*: [M + H]^+^ calcd for C_21_H_27_N_2_OS, 355.1844; found, 355.1838. HPLC: purity = 100%; *R*_t_ = 2.945 min.

#### *N*-(1-(Cyclopentylmethyl)­piperidin-4-yl)-*N*-phenylthiophene-2-carboxamide Hydrochloride **(16)**

The title compound was prepared following the general procedure as a white solid in 86% yield. ^1^H NMR (400 MHz, DMSO-*d*_6_): δ 9.13 (s, 1H), 7.62 (dd, *J* = 5.0, 1.3 Hz, 1H), 7.53 (dd, *J* = 4.9, 2.0 Hz, 3H), 7.35 (dd, *J* = 6.5, 3.0 Hz, 2H), 6.84 (dd, *J* = 5.1, 3.8 Hz, 1H), 6.44 (dd, *J* = 3.9, 1.2 Hz, 1H), 4.85 (tt, *J* = 12.3, 3.8 Hz, 1H), 3.51 (d, *J* = 12.2 Hz, 2H), 3.20–3.05 (m, 2H), 2.98 (dd, *J* = 7.2, 5.4 Hz, 2H), 2.27–2.12 (m, 1H), 2.04 (d, *J* = 13.5 Hz, 2H), 1.78 (td, *J* = 10.9, 9.9, 4.8 Hz, 4H), 1.64–1.43 (m, 4H), 1.16 (d, *J* = 13.1 Hz, 2H). ^13^C NMR (100 MHz, DMSO-*d*_6_): δ 161.61, 138.86, 138.33, 132.30, 132.03, 131.75, 130.09, 129.84, 127.47, 61.39, 51.79, 51.12, 34.86, 31.23, 27.30, 25.09. HRMS (ESI) *m*/*z*: [M + H]^+^ calcd for C_22_H_29_N_2_OS, 369.2001; found, 369.1995. HPLC: purity = 97.10%; *R*_t_ = 4.068 min.

#### *N*-(1-(Cyclohexylmethyl)­piperidin-4-yl)-*N*-phenylthiophene-2-carboxamide Hydrochloride **(17)**

The title compound was prepared following the general procedure as a white solid in 91% yield. ^1^H NMR (400 MHz, DMSO-*d*_6_): δ 9.13 (s, 1H), 7.62 (dd, *J* = 5.1, 1.2 Hz, 1H), 7.57–7.45 (m, 3H), 7.40–7.27 (m, 2H), 6.84 (dd, *J* = 5.0, 3.8 Hz, 1H), 6.43 (dd, *J* = 3.9, 1.2 Hz, 1H), 4.84 (tt, *J* = 12.2, 3.8 Hz, 1H), 3.49 (d, *J* = 12.2 Hz, 2H), 3.10 (dt, *J* = 13.8, 10.9 Hz, 2H), 2.84 (t, *J* = 6.0 Hz, 2H), 2.03 (d, *J* = 13.4 Hz, 2H), 1.87–1.54 (m, 8H), 1.28–1.05 (m, 3H), 0.98–0.82 (m, 2H). ^13^C NMR (100 MHz, DMSO-*d*_6_): δ 161.63, 138.84, 138.30, 132.30, 132.05, 131.75, 130.08, 129.84, 127.47, 62.35, 52.06, 51.11, 32.60, 30.89, 27.22, 25.90, 25.37. HRMS (ESI) *m*/*z*: [M + H]^+^ calcd for C_23_H_31_N_2_OS, 383.2157; found, 383.2151. HPLC: purity = 100%; *R*_t_ = 2.025 min.

#### *N*-(1-Benzylpiperidin-4-yl)-*N*-phenylthiophene-2-carboxamide Hydrochloride **(18)**

The title compound was prepared following the general procedure as a white solid in 90% yield. ^1^H NMR (400 MHz, DMSO-*d*_6_): δ 10.06 (s, 1H), 7.61 (dd, *J* = 5.1, 1.2 Hz, 1H), 7.58–7.35 (m, 8H), 7.34–7.20 (m, 2H), 6.83 (dd, *J* = 5.1, 3.8 Hz, 1H), 6.42 (dd, *J* = 3.9, 1.3 Hz, 1H), 4.82 (tt, *J* = 12.2, 3.8 Hz, 1H), 4.23 (d, *J* = 5.3 Hz, 2H), 3.40–3.33 (m, 2H), 3.16 (td, *J* = 13.6, 13.0, 6.7 Hz, 2H), 2.05 (d, *J* = 13.2 Hz, 2H), 1.73 (q, *J* = 12.8, 12.0 Hz, 2H). ^13^C NMR (100 MHz, DMSO-*d*_6_): δ 161.60, 138.83, 138.29, 132.30, 132.03, 131.77, 131.64, 130.31, 130.07, 129.90, 129.82, 129.20, 127.47, 59.33, 51.25, 51.08, 27.25. HRMS (ESI) *m*/*z*: [M + H]^+^ calcd for C_23_H_25_N_2_OS, 377.1688; found, 377.1681. HPLC: purity = 99.85%; *R*_t_ = 3.775 min.

#### *N*-(1-Allylpiperidin-4-yl)-*N*-phenylthiophene-3-carboxamide Hydrochloride **(19)**

The title compound was prepared following the general procedure as a white solid in 54% yield. ^1^H NMR (400 MHz, DMSO-*d*_6_): δ 10.35 (s, 1H), 7.38 (q, *J* = 4.1 Hz, 3H), 7.29 (dd, *J* = 5.1, 3.0 Hz, 1H), 7.25–7.14 (m, 3H), 6.80 (dd, *J* = 5.1, 1.3 Hz, 1H), 5.93 (td, *J* = 10.3, 5.4 Hz, 1H), 5.57–5.39 (m, 2H), 4.80 (m, 1H), 3.73–3.59 (m, 2H), 3.46–3.38 (m, 2H), 3.19–2.97 (m, 2H), 2.06 (d, *J* = 13.4 Hz, 2H), 1.76 (dd, *J* = 12.6, 3.8 Hz, 2H). ^13^C NMR (100 MHz, DMSO-*d*_6_): δ 164.56, 139.12, 137.57, 131.16, 129.67, 129.65, 128.87, 128.46, 127.99, 125.76, 125.06, 58.23, 50.98, 27.58. HRMS (ESI) *m*/*z*: [M + H]^+^ calcd for C_19_H_23_N_2_OS, 327.1531; found, 327.1541. HPLC: purity = 99.36%; *R*_t_ = 3.275 min.

#### *N*-(1-(Cyclopropylmethyl)­piperidin-4-yl)-*N*-phenylthiophene-3-carboxamide Hydrochloride **(20)**

The title compound was prepared following the general procedure as a white solid in 59% yield. ^1^H NMR (400 MHz, DMSO-*d*_6_): δ 9.94 (s, 1H), 7.39 (d, *J* = 6.6 Hz, 3H), 7.30 (dd, *J* = 5.1, 3.0 Hz, 1H), 7.26–7.11 (m, 3H), 6.81 (d, *J* = 5.1 Hz, 1H), 4.80 (m, *J* = 12.4, 7.7, 3.8 Hz, 1H), 3.54 (d, *J* = 12.1 Hz, 2H), 3.12 (dt, *J* = 13.1, 10.5 Hz, 2H), 2.89 (dd, *J* = 7.3, 5.1 Hz, 2H), 2.06 (d, *J* = 13.5 Hz, 2H), 1.90–1.72 (m, 2H), 1.14–0.99 (m, 1H), 0.59 (dt, *J* = 8.5, 3.2 Hz, 2H), 0.41–0.27 (m, 2H). ^13^C NMR (100 MHz, DMSO-*d*_6_): δ 164.56, 139.12, 137.59, 131.18, 129.68, 129.64, 128.87, 128.48, 125.76, 60.40, 51.10, 51.02, 27.53, 5.80, 4.60. HRMS (ESI) *m*/*z*: [M + H]^+^ calcd for C_20_H_25_N_2_OS, 341.1688; found, 341.1697. HPLC: purity = 99.25%; *R*_t_ = 4.152 min.

#### *N*-(1-(Cyclobutylmethyl)­piperidin-4-yl)-*N*-phenylthiophene-3-carboxamide Hydrochloride **(21)**

The title compound was prepared following the general procedure as a white solid in 67% yield. ^1^H NMR (400 MHz, DMSO-*d*_6_): δ 10.10 (s, 1H), 7.46–7.34 (m, 3H), 7.29 (dd, *J* = 5.1, 3.0 Hz, 1H), 7.26–7.04 (m, 3H), 4.78 (m, 1H), 3.39–3.28 (m, 2H), 3.05 (dd, *J* = 28.0, 8.5, 4.0 Hz, 4H), 2.83–2.67 (m, 1H), 2.02 (dt, *J* = 15.9, 5.9 Hz, 4H), 1.92–1.66 (m, 6H). ^13^C NMR (100 MHz, DMSO-*d*_6_): δ 164.53, 139.10, 137.58, 131.18, 129.67, 129.63, 128.85, 128.48, 125.74, 61.05, 51.31, 50.91, 30.43, 27.52, 27.20, 18.52. HRMS (ESI) *m*/*z*: [M + H]^+^ calcd for C_21_H_27_N_2_OS, 355.1844; found, 355.1842. HPLC: purity = 100%; *R*_t_ = 2.615 min.

#### *N*-(1-(Cyclopentylmethyl)­piperidin-4-yl)-*N*-phenylthiophene-3-carboxamide Hydrochloride **(22)**

The title compound was prepared following the general procedure as a white solid in 65% yield. ^1^H NMR (400 MHz, DMSO-*d*_6_): δ 9.49 (s, 1H), 7.46–7.33 (m, 3H), 7.30 (dd, *J* = 5.1, 3.0 Hz, 1H), 7.27–7.06 (m, 3H), 6.80 (dd, *J* = 5.2, 1.3 Hz, 1H), 4.80 (td, *J* = 10.2, 8.4, 6.1 Hz, 1H), 3.50 (d, *J* = 12.1 Hz, 2H), 3.19–3.02 (m, 2H), 2.97 (dd, *J* = 7.1, 5.4 Hz, 2H), 2.27–2.11 (m, 1H), 2.02 (d, *J* = 13.5 Hz, 2H), 1.91–1.70 (m, 4H), 1.64–1.42 (m, 4H), 1.27–1.13 (m, 2H). ^13^C NMR (100 MHz, DMSO-*d*_6_): δ 164.57, 139.10, 137.58, 131.26, 129.67, 129.64, 128.87, 128.47, 125.75, 61.41, 51.87, 50.91, 34.87, 31.22, 27.43, 25.08. HRMS (ESI) *m*/*z*: [M + H]^+^ calcd for C_22_H_29_N_2_OS, 369.2001; found, 369.2008. HPLC: purity = 96.26%; *R*_t_ = 3.585 min.

#### *N*-(1-(Cyclohexylmethyl)­piperidin-4-yl)-*N*-phenylthiophene-3-carboxamide Hydrochloride **(23)**

The title compound was prepared following the general procedure as a white solid in 66% yield. ^1^H NMR (400 MHz, DMSO-*d*_6_): δ 9.67 (s, 1H), 7.45–7.32 (m, 3H), 7.30 (dd, *J* = 5.1, 3.0 Hz, 1H), 7.26–7.17 (m, 3H), 6.80 (d, *J* = 5.2 Hz, 1H), 4.81 (m, *J* = 12.3, 3.9 Hz, 1H), 3.49 (s, 2H), 3.10 (dt, *J* = 12.8, 9.8 Hz, 2H), 2.83 (t, *J* = 5.9 Hz, 2H), 2.00 (dd, *J* = 13.2, 3.7 Hz, 2H), 1.95–1.82 (m, 2H), 1.73 (m, 3H), 1.69–1.53 (m, 3H), 1.28–1.02 (m, 3H), 0.98–0.81 (m, 2H). ^13^C NMR (100 MHz, DMSO-*d*_6_): δ 164.55, 139.08, 137.60, 131.23, 129.64, 129.61, 128.83, 128.48, 125.74, 62.36, 52.08, 50.96, 32.63, 31.00, 27.34, 25.91, 25.41. HRMS (ESI) *m*/*z*: [M + H]^+^ calcd for C_23_H_31_N_2_OS, 383.2157; found, 383.2158. HPLC: purity = 100%; *R*_t_ = 1.885 min.

#### *N*-(1-Benzylpiperidin-4-yl)-*N*-phenylthiophene-3-carboxamide Hydrochloride **(24)**

The title compound was prepared following the general procedure as a white solid in 68% yield. ^1^H NMR (400 MHz, DMSO-*d*_6_): δ 10.28 (s, 1H), 7.54 (dd, *J* = 6.4, 3.0 Hz, 2H), 7.44 (m, *J* = 3.4, 2.9 Hz, 3H), 7.41–7.34 (m, 3H), 7.29 (dd, *J* = 5.1, 3.0 Hz, 1H), 7.19 (m, *J* = 7.1, 2.1 Hz, 3H), 4.79 (tt, *J* = 12.3, 3.8 Hz, 1H), 4.23 (d, *J* = 5.3 Hz, 2H), 3.34 (s, 2H), 3.15 (d, *J* = 14.1 Hz, 2H), 2.03 (d, *J* = 13.2 Hz, 2H), 1.78 (dd, *J* = 13.0, 3.8 Hz, 2H).^13^C NMR (100 MHz, DMSO-*d*_6_): δ 171.60, 153.19, 143.60, 142.85, 142.77, 135.56, 132.83, 132.49, 130.70, 130.51, 122.27, 121.82, 119.42, 119.31, 119.12, 110.58, 109.91, 109.66, 52.28, 47.30, 42.89, 42.54, 33.52, 12.16. HRMS (ESI) *m*/*z*: [M + H]^+^ calcd for C_23_H_25_N_2_OS, 377.1688; found, 377.1687. HPLC: purity = 100%; *R*_t_ = 3.357 min.

#### *N*-(1-Allylpiperidin-4-yl)-*N*-phenyl-1*H*-pyrrole-2-carboxamide Hydrochloride **(25)**

The title compound was prepared following the general procedure as a white solid in 78% yield. ^1^H NMR (400 MHz, DMSO-*d*_6_): δ 11.42 (s, 1H), 9.45 (d, *J* = 71.8 Hz, 1H), 7.53 (m, *J* = 3.6, 3.2 Hz, 3H), 7.29 (dd, *J* = 6.8, 3.0 Hz, 2H), 6.77 (td, *J* = 2.8, 1.4 Hz, 1H), 5.87 (s, 1H), 5.75 (q, *J* = 2.7 Hz, 1H), 5.57–5.38 (m, 2H), 4.99–4.75 (m, 1H), 4.51 (s, 1H), 3.66 (t, *J* = 6.1 Hz, 2H), 3.43 (d, *J* = 12.3 Hz, 2H), 3.11 (q, *J* = 12.0 Hz, 2H), 2.05 (d, *J* = 13.5 Hz, 2H), 1.65 (s, 2H). ^13^C NMR (100 MHz, DMSO-*d*_6_): δ 160.83, 138.93, 131.65, 129.90, 129.42, 125.18, 122.08, 113.38, 109.15, 58.23, 50.99, 27.62. HRMS (ESI) *m*/*z*: [M + H]^+^ calcd for C_19_H_24_N_3_O, 310.1919; found, 310.1913. HPLC: purity = 98.41%; *R*_t_ = 1.510 min.

#### *N*-(1-(Cyclopropylmethyl)­piperidin-4-yl)-*N*-phenyl-1*H*-pyrrole-2-carboxamide Hydrochloride **(26)**

The title compound was prepared following the general procedure as a white solid in 83% yield. ^1^H NMR (400 MHz, DMSO-*d*_6_): δ 11.43 (s, 1H), 9.52 (s, 1H), 7.53 (dd, *J* = 4.9, 1.8 Hz, 3H), 7.38–7.18 (m, 2H), 6.77 (td, *J* = 2.7, 1.4 Hz, 1H), 5.75 (dt, *J* = 3.8, 2.4 Hz, 1H), 4.87 (m, 1H), 4.59–4.44 (m, 1H), 3.55 (d, *J* = 12.2 Hz, 2H), 3.50–3.32 (m, 2H), 2.90 (dd, *J* = 7.3, 5.1 Hz, 2H), 2.04 (d, *J* = 13.4 Hz, 2H), 1.73 (dd, *J* = 14.4, 10.8 Hz, 2H), 1.15–0.98 (m, 1H), 0.68–0.54 (m, 2H), 0.39–0.29 (m, 2H). ^13^C NMR (100 MHz, DMSO-*d*_6_): δ 160.84, 138.95, 131.70, 129.90, 129.40, 125.20, 122.07, 113.37, 109.15, 51.05, 27.57, 5.81, 4.58. HRMS (ESI) *m*/*z*: [M + H]^+^ calcd for C_20_H_26_N_3_O, 324.2076; found, 324.2085. HPLC: purity = 99.16%; *R*_t_ = 1.737 min.

#### *N*-(1-(Cyclobutylmethyl)­piperidin-4-yl)-*N*-phenyl-1*H*-pyrrole-2-carboxamide Hydrochloride **(27)**

The title compound was prepared following the general procedure as a white solid in 84% yield. ^1^H NMR (400 MHz, DMSO-*d*_6_): δ 11.42 (s, 1H), 9.09 (s, 1H), 7.53 (m, *J* = 3.6, 3.2 Hz, 3H), 7.36–7.18 (m, 2H), 6.76 (td, *J* = 2.8, 1.4 Hz, 1H), 5.74 (dt, *J* = 3.8, 2.4 Hz, 1H), 4.86 (t, *J* = 12.2 Hz, 1H), 4.51 (d, *J* = 3.5 Hz, 1H), 3.38 (d, *J* = 11.8 Hz, 4H), 3.14–3.08 (m, 1H), 3.04 (dd, *J* = 7.1, 5.2 Hz, 2H), 2.65 (d, *J* = 9.1 Hz, 1H), 2.08–1.99 (m, 3H), 1.92–1.58 (m, 6H).). ^13^C NMR (100 MHz, DMSO-*d*_6_): δ159.74, 137.85, 130.66, 128.82, 128.32, 124.09, 120.98, 112.29, 108.06, 60.04, 50.35, 48.86, 47.97, 29.35, 26.48, 26.01, 17.42, 0.50. HRMS (ESI) *m*/*z*: [M + H]^+^ calcd for C_21_H_28_N_3_O, 338.2232; found, 338.2227. HPLC: purity = 98.59%; *R*_t_ = 2.345 min.

#### *N*-(1-(Cyclopentylmethyl)­piperidin-4-yl)-*N*-phenyl-1*H*-pyrrole-2-carboxamide Hydrochloride **(28)**

The title compound was prepared following the general procedure as a white solid in 88% yield. ^1^H NMR (400 MHz, DMSO-*d*_6_): δ 11.40 (s, 1H), 8.94 (s, 1H), 7.62–7.41 (m, 3H), 7.37–7.22 (m, 2H), 6.77 (td, *J* = 2.7, 1.4 Hz, 1H), 5.75 (dt, *J* = 3.8, 2.5 Hz, 1H), 5.01–4.79 (m, 1H), 4.58–4.40 (m, 1H), 3.51 (d, *J* = 12.2 Hz, 2H), 3.20–3.06 (m, 2H), 2.98 (dd, *J* = 7.2, 5.3 Hz, 2H), 2.24–2.12 (m, 1H), 2.01 (d, *J* = 13.5 Hz, 2H), 1.83–1.65 (m, 4H), 1.65–1.43 (m, 4H), 1.17 (dd, *J* = 13.2, 6.9 Hz, 2H). ^13^C NMR (100 MHz, DMSO-*d*_6_): δ 160.84, 138.94, 131.80, 129.91, 129.41, 125.19, 122.07, 113.37, 109.15, 61.43, 51.92, 50.01, 34.87, 31.15, 27.46, 25.09. HRMS (ESI) *m*/*z*: [M + H]^+^ calcd for C_23_H_30_N_3_O, 352.2389; found, 352.2381. HPLC: purity = 100%; *R*_t_ = 3.132 min.

#### *N*-(1-(Cyclohexylmethyl)­piperidin-4-yl)-*N*-phenyl-1*H*-pyrrole-2-carboxamide Hydrochloride **(29)**

The title compound was prepared following the general procedure as a white solid in 80% yield. ^1^H NMR (400 MHz, DMSO-*d*_6_): δ 11.42 (s, 1H), 9.15 (s, 1H), 7.57–7.43 (m, 3H), 7.29 (dd, *J* = 6.5, 3.0 Hz, 2H), 6.77 (td, *J* = 2.8, 1.4 Hz, 1H), 5.74 (dt, *J* = 3.9, 2.4 Hz, 1H), 4.87 (m, 1H), 4.49 (m, 1H), 3.48 (d, *J* = 12.5 Hz, 2H), 3.11 (dt, *J* = 13.7, 10.8 Hz, 2H), 2.83 (t, *J* = 6.0 Hz, 2H), 1.98 (d, *J* = 13.4 Hz, 2H), 1.84–1.55 (m, 8H), 1.29–1.08 (m, 3H), 0.97–0.83 (m, 2H). ^13^C NMR (100 MHz, DMSO-*d*_6_): δ 160.83, 138.93, 131.74, 129.87, 129.38, 125.20, 122.06, 113.37, 109.14, 65.39, 62.34, 55.40, 52.10, 50.16, 32.63, 30.99, 27.38, 25.91, 25.40, 15.64. HRMS (ESI) *m*/*z*: [M + H]^+^ calcd for C_23_H_32_N_3_O, 366.2545; found, 366.2539. HPLC: purity = 97.35%; *R*_t_ = 4.743 min.

#### *N*-(1-Benzylpiperidin-4-yl)-*N*-phenyl-1*H*-pyrrole-2-carboxamide Hydrochloride **(30)**

The title compound was prepared following the general procedure as a white solid in 79% yield. ^1^H NMR (400 MHz, DMSO-*d*_6_): δ 11.42 (s, 1H), 9.42 (s, 1H), 7.59–7.39 (m, 7H), 7.38–7.11 (m, 3H), 6.76 (d, *J* = 3.3 Hz, 1H), 5.81–5.69 (m, 1H), 4.87 (s, 1H), 4.50 (s, 1H), 4.24 (d, *J* = 5.3 Hz, 2H), 3.18 (d, *J* = 11.0 Hz, 3H), 2.03 (d, *J* = 13.4 Hz, 2H), 1.65 (s, 2H). ^13^C NMR (100 MHz, DMSO-*d*_6_): δ 160.83, 138.90, 131.74, 131.68, 130.29, 129.95, 129.89, 129.39, 129.23, 125.15, 122.08, 113.37, 109.15, 59.41, 55.39, 51.23, 50.16, 27.42. HRMS (ESI) *m*/*z*: [M + H]^+^ calcd for C_23_H_26_N_3_O, 360.2076; found, 360.2070. HPLC: purity = 98.71%; *R*_t_ = 2.830 min.

#### *N*-(1-Allylpiperidin-4-yl)-*N*-phenyl-1*H*-pyrrole-3-carboxamide Hydrochloride **(31)**

The title compound was prepared following the general procedure as a white solid in 33% yield.^1^H NMR (400 MHz, DMSO-*d*_6_): δ 10.90 (s, 1H), 9.22 (s, 1H), 7.49 (dd, *J* = 5.0, 1.9 Hz, 3H), 7.24 (dd, *J* = 6.6, 3.0 Hz, 2H), 6.48 (q, *J* = 2.4 Hz, 1H), 6.07 (dt, *J* = 3.2, 1.8 Hz, 1H), 5.88–5.79 (m, 1H), 5.61 (q, *J* = 2.4 Hz, 1H), 5.52–5.42 (m, 2H), 4.92–4.83 (m, 1H), 3.66 (t, *J* = 6.0 Hz, 2H), 3.42 (d, *J* = 12.2 Hz, 2H), 3.10 (q, *J* = 11.6 Hz, 2H), 2.04 (d, *J* = 13.5 Hz, 2H), 1.62 (q, *J* = 12.8 Hz, 2H). ^13^C NMR (100 MHz, DMSO-*d*_6_): δ 170.08, 146.92, 143.65, 134.04, 129.45, 127.24, 126.85, 126.59, 123.97, 116.20, 111.54, 51.71, 46.85, 42.65, 33.55, 24.39, 12.11. HRMS (ESI) *m*/*z*: [M + H]^+^ calcd for C_19_H_24_N_3_O, 310.1919; found, 310.1907. HPLC: purity = 98.85%; *R*_t_ = 2.980 min.

#### *N*-(1-(Cyclopropylmethyl)­piperidin-4-yl)-*N*-phenyl-1*H*-pyrrole-3-carboxamide Hydrochloride **(32)**

The title compound was prepared following the general procedure as a white solid in 36% yield. ^1^H NMR (400 MHz, DMSO-*d*_6_): δ 10.91 (s, 1H), 9.28 (s, 1H), 7.57–7.42 (m, 3H), 7.32–7.13 (m, 2H), 6.54–6.40 (m, 1H), 6.07 (dt, *J* = 3.3, 1.8 Hz, 1H), 5.62 (td, *J* = 2.7, 1.5 Hz, 1H), 4.85 (t, *J* = 3.7 Hz, 1H), 3.54 (d, *J* = 12.0 Hz, 2H), 3.18–3.06 (m, 2H), 2.91 (s, 2H), 2.03 (d, *J* = 13.5 Hz, 2H), 1.68 (dd, *J* = 12.8, 3.7 Hz, 2H), 1.02 (td, *J* = 7.7, 3.9 Hz, 1H), 0.67–0.52 (m, 2H), 0.33 (dd, *J* = 4.7, 1.7 Hz, 2H). ^13^C NMR (100 MHz, DMSO-*d*_6_): δ 170.11, 142.28, 133.91, 129.47, 128.08, 127.20, 126.80, 124.04, 121.68, 118.59, 116.28, 111.55, 110.11, 47.37, 46.88, 42.67, 28.28, 16.13, 12.30. HRMS (ESI) *m*/*z*: [M + H]^+^ calcd for C_20_H_26_N_3_O, 324.2076; found, 324.2060. HPLC: purity = 99.12%; *R*_t_ = 1.322 min.

#### *N*-(1-(Cyclobutylmethyl)­piperidin-4-yl)-*N*-phenyl-1*H*-pyrrole-3-carboxamide Hydrochloride **(33)**

The title compound was prepared following the general procedure as a white solid in 31% yield. ^1^H NMR (400 MHz, DMSO-*d*_6_): δ 10.90 (s, 1H), 9.17 (s, 1H), 7.49 (dd, *J* = 5.0, 1.8 Hz, 3H), 7.24–7.15 (m, 2H), 6.48 (q, *J* = 2.4 Hz, 1H), 6.06 (dt, *J* = 3.2, 1.8 Hz, 1H), 5.62 (td, *J* = 2.7, 1.6 Hz, 1H), 4.87–4.80 (m, 1H), 3.47–3.36 (m, 3H), 3.17–3.10 (m, 1H), 3.05–3.02 (m, 2H), 2.69–2.62 (t, *J* = 6.0 Hz, 1H), 2.08–2.08 (m, *J* = 12.5 Hz, 4H), 1.89–1.83 (t, *J* = 6.0 Hz, 1H), 1.77–1.72 (m, 3H), 1.69–1.62 (m, 2H). ^13^C NMR (100 MHz, DMSO-*d*_6_): δ 160.56, 139.90, 131.41, 129.72, 129.06, 125.62, 121.64, 112.96, 108.98, 65.38, 60.71, 55.11, 47.85, 47.77, 36.41, 30.60, 29.79, 29.47, 28.12, 27.39, 27.21, 25.95, 25.81, 18.56, 15.64. HRMS (ESI) *m*/*z*: [M + H]^+^ calcd for C_21_H_28_N_3_O, 338.2232; found, 338.2240. HPLC: purity = 96.85%; *R*_t_ = 1.343 min.

#### *N*-(1-(Cyclopentylmethyl)­piperidin-4-yl)-*N*-phenyl-1*H*-pyrrole-3-carboxamide Hydrochloride **(34)**

The title compound was prepared following the general procedure as a white solid in 36% yield. ^1^H NMR (400 MHz, DMSO-*d*_6_): δ 10.92 (s, 1H), 9.00 (s, 1H), 7.57–7.43 (m, 3H), 7.32–7.17 (m, 2H), 6.48 (d, *J* = 2.5 Hz, 1H), 6.11–5.98 (m, 1H), 5.62 (q, *J* = 1.1 Hz, 1H), 4.85 (m, 1H), 3.50 (d, *J* = 12.0 Hz, 2H), 3.10 (d, *J* = 11.9 Hz, 2H), 2.98 (dd, *J* = 7.2, 5.3 Hz, 2H), 2.18 (m, 1H), 1.99 (d, *J* = 13.4 Hz, 2H), 1.83–1.69 (m, 4H), 1.60–1.44 (m, 4H), 1.18 (q, *J* = 9.1, 8.2 Hz, 2H).^13^C NMR (100 MHz, DMSO-*d*_6_): δ 170.11, 142.28, 133.91, 129.47, 128.08, 127.20, 126.80, 124.04, 121.68, 118.59, 116.28, 111.55, 110.11, 47.37, 46.88, 42.67, 28.28, 16.13, 12.30. HRMS (ESI) *m*/*z*: [M + H]^+^ calcd for C_23_H_30_N_3_O, 352.2389; found, 352.2385. HPLC: purity = 97.27%; *R*_t_ = 1.723 min.

#### *N*-(1-(Cyclohexylmethyl)­piperidin-4-yl)-*N*-phenyl-1*H*-pyrrole-3-carboxamide Hydrochloride **(35)**

The title compound was prepared following the general procedure as a white solid in 32% yield. ^1^H NMR (400 MHz, DMSO-*d*_6_): δ 10.90 (s, 1H), 8.67–8.66 (b, 1H), 7.50–7.46 (m, 3H), 7.25–7.23 (m, 2H), 6.48–6.47 (d, *J* = 2.5 Hz, 1H), 6.06–6.05 (t, 1H), 5.61–5.60 (t, *J* = 1.1 Hz, 1H), 4.88–4.82 (m, 1H), 3.53–3.50 (d, *J* = 12.0 Hz, 3H), 3.16–3.05 (d, *J* = 11.9 Hz, 2H), 2.85–2.82 (t, *J* = 7.2, 5.3 Hz, 2H), 1.99–1.96 (dd, *J* = 13.4 Hz, 2H), 1.72–1.63 (m, 8H), 1.22–1.19 (m, 2H), 0.90–0.87 (q, *J* = 9.1, 8.2 Hz, 2H).^13^C NMR (100 MHz, DMSO-*d*_6_): δ 170.11, 142.28, 133.91, 129.47, 128.08, 127.20, 126.80, 124.04, 121.68, 118.59, 116.28, 111.55, 110.11, 47.37, 46.88, 42.67, 28.28, 16.13, 12.30. HRMS (ESI) *m*/*z*: [M + H]^+^ calcd for C_23_H_32_N_3_O, 366.2545; found, 366.2537. HPLC: purity = 96.99%; *R*_t_ = 2.458 min.

#### *N*-(1-Benzylpiperidin-4-yl)-*N*-phenyl-1*H*-pyrrole-3-carboxamide Hydrochloride **(36)**

The title compound was prepared following the general procedure as a white solid in 20% yield. ^1^H NMR (400 MHz, DMSO-*d*_6_): δ 10.91 (s, 1H), 9.62 (s, 1H), 7.46 (dd, *J* = 5.6, 2.6 Hz, 8H), 6.47 (q, *J* = 2.4 Hz, 1H), 6.05 (dd, *J* = 3.1, 1.6 Hz, 1H), 5.61 (dd, *J* = 2.7, 1.5 Hz, 1H), 4.89–4.78 (m, 1H), 4.23 (d, *J* = 5.3 Hz, 2H), 3.42–3.28 (m, 3H), 3.15 (d, *J* = 12.1 Hz, 2H), 2.00 (d, *J* = 13.4 Hz, 2H), 1.65 (d, *J* = 12.7 Hz, 2H). ^13^C NMR (100 MHz, DMSO-*d*_6_): δ 170.11, 142.28, 133.91, 129.47, 128.08, 127.20, 126.80, 124.04, 121.68, 118.59, 116.28, 111.55, 110.11, 47.37, 46.88, 42.67, 28.28, 16.13, 12.30. HRMS (ESI) *m*/*z*: [M + H]^+^ calcd for C_23_H_26_N_3_O, 360.2076; found, 360.2061. HPLC: purity = 97.27%; *R*_t_ = 1.723 min.

#### *N*-(3-Allyl-3-azaspiro­[5.5]­undecan-9-yl)-*N*-phenylfuran-2-carboxamide Hydrochloride **(37)**

The title compound was prepared following the general procedure as a white solid in 68% yield. ^1^H NMR (400 MHz, DMSO-*d*_6_): δ 10.40 (s, 1H), 7.62 (d, *J* = 1.7 Hz, 1H), 7.54–7.40 (m, 3H), 7.35–7.19 (m, 2H), 6.30 (dd, *J* = 3.6, 1.7 Hz, 1H), 5.95 (m, *J* = 16.9, 9.8, 7.0 Hz, 1H), 5.63–5.33 (m, 3H), 4.48 (dt, *J* = 12.0, 3.9 Hz, 1H), 3.65 (dd, *J* = 7.1, 5.1 Hz, 2H), 3.23–3.07 (m, 2H), 3.00–2.78 (m, 2H), 2.13 (dd, *J* = 13.7, 3.1 Hz, 1H), 1.70 (dd, *J* = 15.9, 11.7 Hz, 4H), 1.51–1.06 (m, 7H). ^13^C NMR (100 MHz, DMSO-*d*_6_): δ 158.22, 147.41, 145.29, 139.32, 130.93, 129.74, 129.14, 128.16, 124.92, 115.80, 111.56, 57.92, 55.67, 47.39, 38.82, 36.41, 29.72, 29.58, 28.13, 25.77, 25.61. HRMS (ESI) *m*/*z*: [M + H]^+^ calcd for C_24_H_31_N_2_O_2_, 379.2386; found, 379.2388. HPLC: purity = 100%; *R*_t_ = 2.680 min.

#### *N*-(3-(Cyclopropylmethyl)-3-azaspiro­[5.5]­undecan-9-yl)-*N*-phenylfuran-2-carboxamide Hydrochloride **(38)**

The title compound was prepared following the general procedure as a white solid in 73% yield. ^1^H NMR (400 MHz, DMSO-*d*_6_): δ 10.09 (s, 1H), 7.62 (d, *J* = 1.7 Hz, 1H), 7.53–7.42 (m, 3H), 7.32–7.22 (m, 2H), 6.30 (dd, *J* = 3.5, 1.8 Hz, 1H), 5.41 (d, *J* = 3.6 Hz, 1H), 4.47 (tt, *J* = 11.9, 3.8 Hz, 1H), 3.27 (t, *J* = 16.1 Hz, 2H), 2.90 (m, 4H), 2.14 (dt, *J* = 13.6, 3.1 Hz, 1H), 1.62–1.75 (m, 4H), 1.50–1.23 (m, 6H), 1.18–1.02 (m, 2H), 0.65–0.54 (m, 2H), 0.34 (dt, *J* = 6.1, 4.2 Hz, 2H). ^13^C NMR (100 MHz, DMSO-*d*_6_): δ 145.28, 130.93, 129.73, 129.13, 115.79, 111.54, 60.00, 55.64, 47.44, 40.24, 39.61, 38.88, 36.37, 28.07, 25.77, 25.62, 5.74, 4.62, 4.49. HRMS (ESI) *m*/*z*: [M + H]^+^ calcd for C_25_H_33_N_2_O_2_, 393.2542; found, 393.2551. HPLC: purity = 99.71%; *R*_t_ = 2.723 min.

#### *N*-(3-(Cyclobutylmethyl)-3-azaspiro­[5.5]­undecan-9-yl)-*N*-phenylfuran-2-carboxamide Hydrochloride **(39)**

The title compound was prepared following the general procedure as a white solid in 76% yield. ^1^H NMR (400 MHz, DMSO-*d*_6_): δ 10.22 (s, 1H), 7.62 (d, *J* = 1.7 Hz, 1H), 7.47 (m, 3H), 7.26 (dd, *J* = 6.5, 3.0 Hz, 2H), 6.30 (dd, *J* = 3.5, 1.7 Hz, 1H), 5.41 (d, *J* = 3.4 Hz, 1H), 4.47 (m, 1H), 3.16–2.96 (m, 4H), 2.96–2.68 (m, 3H), 2.20–2.10 (m, 1H), 2.05 (m, 2H), 1.92–1.64 (m, 7H), 1.60 (dd, *J* = 14.5, 2.9 Hz, 1H), 1.50–1.19 (m, 6H), 1.17–1.04 (m, 1H). ^13^C NMR (100 MHz, DMSO-*d*_6_): δ 158.23, 147.43, 145.27, 139.34, 130.92, 129.73, 129.13, 115.79, 111.54, 60.69, 55.69, 47.73 (d, *J* = 8.0 Hz), 38.89, 36.32, 30.60, 29.73, 29.46, 28.05, 27.44, 27.27, 25.71 (d, *J* = 13.8 Hz), 18.56. HRMS (ESI) *m*/*z*: [M + H]^+^ calcd for C_26_H_35_N_2_O_2_, 407.2699; found, 407.2701. HPLC: purity = 99.16%; *R*_t_ = 2.805 min.

#### *N*-(3-(Cyclopentylmethyl)-3-azaspiro­[5.5]­undecan-9-yl)-*N*-phenylfuran-2-carboxamide Hydrochloride **(40)**

The title compound was prepared following the general procedure as a white solid in 71% yield. ^1^H NMR (400 MHz, DMSO-*d*_6_): δ 9.72 (s, 1H), 7.62 (s, 1H), 7.50–7.44 (m, 3H), 7.26 (dd, *J* = 6.5, 3.0 Hz, 2H), 6.30 (d, *J* = 4.4 Hz, 1H), 5.41 (d, *J* = 3.5 Hz, 1H), 4.47 (tt, *J* = 12.0, 3.9 Hz, 1H), 3.22 (dd, *J* = 24.0, 12.6 Hz, 2H), 3.04–2.78 (m, 4H), 2.18 (m, 2H), 1.85–1.76 (m, 3H), 1.76–1.68 (m, 1H), 1.68 (d, *J* = 10.5 Hz, 1H), 1.64–1.05 (m, 11H). ^13^C NMR (100 MHz, DMSO-*d*_6_): δ 158.23, 147.44, 145.27, 139.35, 130.93, 129.73, 129.13, 115.80, 111.54, 60.94, 55.68, 48.35, 48.23, 36.25, 34.89, 31.37, 29.88, 29.49, 27.99, 25.79, 25.62, 25.09, 25.05. HRMS (ESI) *m*/*z*: [M + H]^+^ calcd for C_27_H_37_N_2_O_2_, 421.2855; found, 421.2857. HPLC: purity = 100%; *R*_t_ = 2.872 min.

#### *N*-(3-(Cyclohexylmethyl)-3-azaspiro­[5.5]­undecan-9-yl)-*N*-phenylfuran-2-carboxamide Hydrochloride **(41)**

The title compound was prepared following the general procedure as a white solid in 69% yield. ^1^H NMR (400 MHz, DMSO-*d*_6_): δ 9.56–9.24 (m, 1H), 7.62 (s, 1H), 7.53–7.37 (m, 3H), 7.26 (dd, *J* = 6.3, 3.0 Hz, 2H), 6.30 (d, *J* = 3.7 Hz, 1H), 5.41 (s, 1H), 4.60–4.37 (m, 1H), 3.20 (dd, *J* = 25.9, 12.7 Hz, 2H), 2.87 (dt, *J* = 23.5, 8.2 Hz, 4H), 2.14 (d, *J* = 13.3 Hz, 1H), 1.67 (m, 10H), 1.46–1.11 (m, 9H), 1.10–0.80 (m, 3H). ^13^C NMR (100 MHz, DMSO-*d*_6_): δ 147.45, 145.28, 139.36, 130.93, 129.72, 129.12, 115.81, 111.55, 61.94, 55.66, 48.68, 48.49, 36.20, 32.54, 30.98, 29.49, 27.94, 25.90, 25.76, 25.58, 25.40. HRMS (ESI) *m*/*z*: [M + H]^+^ calcd for C_28_H_39_N_2_O_2_, 435.3012; found, 435.3022. HPLC: purity = 99.62%; *R*_t_ = 2.987 min.

#### *N*-(3-Benzyl-3-azaspiro­[5.5]­undecan-9-yl)-*N*-phenylfuran-2-carboxamide Hydrochloride **(42)**

The title compound was prepared following the general procedure as a white solid in 80% yield. ^1^H NMR (400 MHz, DMSO-*d*_6_): δ 10.27 (s, 1H), 7.61 (d, *J* = 1.6 Hz, 1H), 7.59–7.50 (m, 2H), 7.44 (m, 6H), 7.32–7.18 (m, 2H), 6.30 (dd, *J* = 3.5, 1.8 Hz, 1H), 5.40 (d, *J* = 3.5 Hz, 1H), 4.46 (m, 1H), 4.23 (d, *J* = 5.4 Hz, 2H), 3.13–2.82 (m, 4H), 2.13 (dd, *J* = 13.4, 3.1 Hz, 1H), 1.69 (m, 4H), 1.50–1.19 (m, 6H), 1.18–1.06 (m, 1H). ^13^C NMR (100 MHz, DMSO-*d*_6_): δ 171.60, 153.19, 143.60, 142.85, 142.77, 135.56, 132.83, 132.49, 130.70, 130.51, 122.27, 121.82, 119.42, 119.31, 119.12, 110.58, 109.91, 109.66, 52.28, 47.30, 42.89, 42.54, 33.52, 12.16. HRMS (ESI) *m*/*z*: [M + H]^+^ calcd for C_28_H_33_N_2_O_2_, 429.2542; found, 429.2518. HPLC: purity = 99.29%; *R*_t_ = 2.803 min.

#### *N*-(3-phenethyl-3-azaspiro­[5.5]­undecan-9-yl)-*N*-phenylfuran-2-carboxamide Hydrochloride **(43)**

The title compound was prepared following the general procedure as a white solid in 78% yield. ^1^H NMR (400 MHz, DMSO-*d*_6_): δ 10.23 (s, 1H), 7.62 (d, *J* = 1.7 Hz, 1H), 7.47 (q, *J* = 2.8 Hz, 3H), 7.41–7.19 (m, 7H), 6.30 (dd, *J* = 3.5, 1.8 Hz, 1H), 5.42 (d, *J* = 3.5 Hz, 1H), 4.47 (m, 1H), 3.21 (td, *J* = 13.8, 12.8, 7.8 Hz, 2H), 3.07–2.85 (m, 4H), 2.15 (dd, *J* = 13.8, 3.2 Hz, 1H), 1.79–1.61 (m, 4H), 1.54–1.12 (m, 7H). ^13^C NMR (100 MHz, DMSO-*d*_6_): δ 162.04, 145.15, 143.13, 139.45, 137.71, 131.21, 129.82, 129.41, 129.26, 129.12, 129.10, 127.23, 123.01, 111.30, 65.38, 56.59, 55.48, 47.82, 47.78, 38.84, 36.43, 29.90, 29.73, 29.61, 28.17, 25.83, 25.70, 15.64. HRMS (ESI) *m*/*z*: [M + H]^+^ calcd for C_29_H_35_N_2_O_2_, 443.2699; found, 443.2704. HPLC: purity = 99.76%; *R*_t_ = 2.893 min.

#### *N*-(9-Allylspiro­[5.5]­undecan-3-yl)-*N*-phenylfuran-3-carboxamide Hydrochloride **(44)**

The title compound was prepared following the general procedure as a white solid in 76% yield. ^1^H NMR (400 MHz, DMSO-*d*_6_): δ 10.42 (s, 1H), 7.52–7.37 (m, 4H), 7.30–7.18 (m, 2H), 6.72 (s, 1H), 6.05–5.87 (m, 2H), 5.50–5.38 (m, 2H), 4.57–4.34 (m, 1H), 3.65 (t, *J* = 6.1 Hz, 2H), 3.17–3.04 (m, 2H), 2.89 (m, 2H), 2.17–2.07 (m, 1H), 1.78–1.59 (m, 4H), 1.48–1.22 (m, 6H), 1.21–1.09 (m, 1H). ^13^C NMR (100 MHz, DMSO-*d*_6_): δ 162.04, 145.14, 143.13, 139.43, 131.21, 129.82, 129.25, 128.16, 124.91, 123.00, 111.30, 57.92, 55.47, 49.05, 47.39, 38.86, 36.41, 29.74, 29.58, 28.13, 25.86, 25.70. HRMS (ESI) *m*/*z*: [M + H]^+^ calcd for C_24_H_31_N_2_O_2_, 379.2386; found, 379.2397. HPLC: purity = 97.41%; *R*_t_ = 2.722 min.

#### *N*-(9-(Cyclopropylmethyl)­spiro­[5.5]­undecan-3-yl)-*N*-phenylfuran-3-carboxamide Hydrochloride **(45)**

The title compound was prepared following the general procedure as a white solid in 78% yield. ^1^H NMR (400 MHz, DMSO-*d*_6_): δ 9.80 (s, 1H), 7.54–7.40 (m, 4H), 7.33–7.19 (m, 2H), 6.72 (s, 1H), 5.98 (d, *J* = 1.5 Hz, 1H), 4.58–4.39 (m, 1H), 3.30 (dd, *J* = 31.7, 16.3 Hz, 2H), 3.01–2.77 (m, 4H), 2.13 (d, *J* = 13.2 Hz, 1H), 1.77–1.58 (m, 4H), 1.51–1.40 (m, 2H), 1.31 (dt, *J* = 26.2, 13.2 Hz, 4H), 1.24–1.12 (m, 1H), 1.05 (dt, *J* = 7.8, 3.8 Hz, 1H), 0.66–0.53 (m, 2H), 0.39–0.27 (m, 2H). ^13^C NMR (100 MHz, DMSO-*d*_6_): δ 162.04, 145.15, 143.14, 139.42, 131.23, 129.83, 129.26, 123.00, 111.30, 65.38, 60.07, 55.43, 49.06, 47.52, 36.43, 29.74, 29.63, 28.15, 25.88, 25.71, 15.64, 5.77, 4.61, 4.50. HRMS (ESI) *m*/*z*: [M + H]^+^ calcd for C_25_H_33_N_2_O_2_, 393.2542; found, 393.2553. HPLC: purity = 99.62%; *R*_t_ = 2.775 min.

#### *N*-(9-(Cyclobutylmethyl)­spiro­[5.5]­undecan-3-yl)-*N*-phenylfuran-3-carboxamide Hydrochloride **(46)**

The title compound was prepared following the general procedure as a white solid in 93% yield. ^1^H NMR (400 MHz, DMSO-*d*_6_): δ 10.24 (s, 1H), 7.42–7.33 (m, 2H), 7.21–7.11 (m, 2H), 7.11–6.99 (m, 2H), 6.66 (d, *J* = 8.1 Hz, 1H), 6.58 (t, *J* = 7.5 Hz, 1H), 4,83 (br s, 1H), 4.07 (d, *J* = 5.3 Hz, 2H), 3.65 (s, 2H), 3.17 (d, *J* = 5.1 Hz, 6H), 0.98 (s, 6H). ^13^C NMR (100 MHz, DMSO-*d*_6_): δ 169.87, 160.36, 132.83, 131.45, 131.37, 127.24, 126.79, 123.97, 116.32, 115.47, 115.26, 111.55, 55.35, 49.06, 46.90, 42.02. HRMS (ESI) *m*/*z*: [M + H]^+^ calcd for C_26_H_35_N_2_O_2_, 407.2699; found, 407.2700. HPLC: purity = 100%; *R*_t_ = 2.852 min.

#### *N*-(9-(Cyclopentylmethyl)­spiro­[5.5]­undecan-3-yl)-*N*-phenylfuran-3-carboxamide Hydrochloride **(47)**

The title compound was prepared following the general procedure as a white solid in 84% yield. ^1^H NMR (400 MHz, DMSO-*d*_6_): δ 9.86 (s, 1H), 7.61 (dd, *J* = 8.1, 1.3 Hz, 1H), 7.35–7.24 (m, 1H), 7.07 (m, 2H), 6.98 (dd, *J* = 7.8, 1.6 Hz, 1H), 6.85 (m, 1H), 6.52 (dd, *J* = 8.1, 1.3 Hz, 1H), 6.42 (m, 1H), 5.08 (s, 1H), 3.51 (s, 2H), 3.17 (t, *J* = 6.3 Hz, 2H), 2.84 (t, *J* = 6.2 Hz, 2H), 2.74 (q, *J* = 7.2 Hz, 4H), 0.90 (t, *J* = 7.2 Hz, 6H). ^13^C NMR (100 MHz, DMSO-*d*_6_): δ 169.73, 143.43, 142.13, 135.70, 131.65, 131.55, 128.58, 128.05, 126.99, 126.43, 125.26, 124.44, 123.77, 119.02, 116.81, 111.59, 110.87, 50.58, 47.00, 42.24, 38.97, 9.83. HRMS (ESI) *m*/*z*: [M + H]^+^ calcd for C_27_H_37_N_2_O_2_, 421.2855; found, 421.2845. HPLC: purity = 100%; *R*_t_ = 2.935 min.

#### *N*-(9-(Cyclohexylmethyl)­spiro­[5.5]­undecan-3-yl)-*N*-phenylfuran-3-carboxamide Hydrochloride **(48)**

The title compound was prepared following the general procedure as a white solid in 94% yield. ^1^H NMR (400 MHz, DMSO-*d*_6_): δ 9.53 (s, 1H), 7.68 (d, *J* = 8.1 Hz, 2H), 7.58 (d, *J* = 8.0 Hz, 2H), 7.10–7.00 (m, 2H), 6.66 (dd, *J* = 8.1, 1.3 Hz, 1H), 6.58 (td, *J* = 7.5, 1.3 Hz, 1H), 4.88 (s, 1H), 3.77 (s, 2H), 3.10 (t, *J* = 6.6 Hz, 2H), 2.69–2.61 (m, 2H), 2.53 (d, *J* = 7.3 Hz, 4H), 0.94 (t, *J* = 7.1 Hz, 6H). ^13^C NMR (100 MHz, DMSO-*d*_6_): δ 169.21, 143.55, 141.56, 130.46, 127.86, 127.55, 127.37, 126.92, 126.24, 125.55, 125.51, 125.48, 125.44, 123.79, 123.54, 116.25, 111.55, 51.52, 46.81, 42.65, 11.75. HRMS (ESI) *m*/*z*: [M + H]^+^ calcd for C_28_H_39_N_2_O_2_, 435.3012; found, 435.3014. HPLC: purity = 100%; *R*_t_ = 3.047 min.

#### *N*-(9-Benzylspiro­[5.5]­undecan-3-yl)-*N*-phenylfuran-3-carboxamide Hydrochloride **(49)**

The title compound was prepared following the general procedure as a white solid in 90% yield. ^1^H NMR (400 MHz, DMSO-*d*_6_): δ 10.38 (s, 1H), 7.80 (d, *J* = 8.2 Hz, 2H), 7.54 (d, *J* = 8.1 Hz, 2H), 7.05 (t, *J* = 7.8 Hz, 2H), 6.66 (dd, *J* = 7.8 Hz, 2H), 4.78 (s, 1H), 3.76 (s, 2H), 3.34 (d, *J* = 4.4 Hz, 2H), 3.04 (d, *J* = 5.9 Hz, 2H), 2.59–2.52 (m, 4H), 0.92 (t, *J* = 7.1 Hz, 6H). ^13^C NMR (100 MHz, DMSO-*d*_6_): δ 171.60, 153.19, 143.60, 142.85, 142.77, 135.56, 132.83, 132.49, 130.70, 130.51, 122.27, 121.82, 119.42, 119.31, 119.12, 110.58, 109.91, 109.66, 52.28, 47.30, 42.89, 42.54, 33.52, 12.16. HRMS (ESI) *m*/*z*: [M + H]^+^ calcd for C_28_H_33_N_2_O_2_, 429.2542; found, 429.2531. HPLC: purity = 99.82%; *R*_t_ = 2.867 min.

#### *N*-(9-Phenethylspiro­[5.5]­undecan-3-yl)-*N*-phenylfuran-3-carboxamide Hydrochloride **(50)**

The title compound was prepared following the general procedure as a white solid in 74% yield. ^1^H NMR (400 MHz, DMSO-*d*_6_): δ 10.55 (s, 1H), 7.57–7.38 (m, 4H), 7.38–7.29 (m, 2H), 7.26 (td, *J* = 7.9, 6.9, 3.9 Hz, 5H), 6.73 (s, 1H), 5.99 (d, *J* = 2.0 Hz, 1H), 4.53–4.40 (m, 1H), 3.40–3.15 (m, 4H), 3.09–2.85 (m, 4H), 2.16 (dd, *J* = 13.4, 3.2 Hz, 1H), 1.84–1.58 (m, 4H), 1.51–1.22 (m, 6H), 1.22–1.11 (m, 1H). ^13^C NMR (100 MHz, DMSO-*d*_6_): δ 162.04, 145.15, 143.13, 139.45, 137.71, 131.21, 129.82, 129.41, 129.26, 129.12, 129.10, 127.23, 123.01, 111.30, 65.38, 56.59, 55.48, 47.82, 47.78, 38.84, 36.43, 29.90, 29.73, 29.61, 28.17, 25.83, 25.70, 15.64. HRMS (ESI) *m*/*z*: [M + H]^+^ calcd for C_29_H_35_N_2_O_2_, 443.2699; found, 443.2698. HPLC: purity = 100%; *R*_t_ = 2.938 min.

#### *N*-(3-Allyl-3-azaspiro­[5.5]­undecan-9-yl)-*N*-phenylthiophene-2-carboxamide Hydrochloride **(51)**

The title compound was prepared following the general procedure as a white powder in 77% yield. ^1^H NMR (400 MHz, DMSO-*d*_6_): δ 10.17 (s, 1H), 7.58 (dd, *J* = 5.0, 1.2 Hz, 1H), 7.48 (m, *J* = 4.2, 1.9 Hz, 3H), 7.35–7.26 (m, 2H), 6.81 (dd, *J* = 5.1, 3.8 Hz, 1H), 6.36 (dd, *J* = 3.8, 1.2 Hz, 1H), 5.94 (m, 1H), 5.52–5.37 (m, 2H), 4.54–4.43 (m, 1H), 3.66 (t, *J* = 6.1 Hz, 2H), 3.16 (dd, *J* = 19.2, 13.5 Hz, 2H), 3.00–2.78 (m, 2H), 2.13 (d, *J* = 13.4 Hz, 1H), 1.70 (dt, *J* = 20.7, 14.3 Hz, 4H), 1.46 (dd, *J* = 15.4, 11.7 Hz, 2H), 1.40–1.21 (m, 4H), 1.18–1.09 (m, 1H). ^13^C NMR (100 MHz, DMSO-*d*_6_): δ 161.27, 139.53, 139.38, 131.84, 131.57, 131.36, 129.93, 129.48, 128.11, 127.37, 125.01, 57.93, 56.31, 47.45, 38.83, 36.45, 29.75, 29.58, 28.18, 25.79, 25.61. HRMS (ESI) *m*/*z*: [M + H]^+^ calcd for C_24_H_31_N_2_OS, 395.2157; found, 395.2150. HPLC: purity = 99.55%; *R*_t_ = 2.832 min.

#### *N*-(3-(Cyclopropylmethyl)-3-azaspiro­[5.5]­undecan-9-yl)-*N*-phenylthiophene-2-carboxamide Hydrochloride **(52)**

The title compound was prepared following the general procedure as a white powder in 91% yield. ^1^H NMR (400 MHz, DMSO-*d*_6_): δ 10.05 (s, 1H), 7.58 (dd, *J* = 5.0, 1.2 Hz, 1H), 7.52–7.44 (m, 3H), 7.34–7.25 (m, 2H), 6.82 (dd, *J* = 5.0, 3.8 Hz, 1H), 6.36 (dd, *J* = 3.9, 1.2 Hz, 1H), 4.49 (m, *J* = 11.8, 3.7 Hz, 1H), 3.31–3.21 (m, 2H), 3.02–2.80 (m, 4H), 2.14 (dd, *J* = 13.7, 3.2 Hz, 1H), 1.81–1.59 (m, 4H), 1.52–1.21 (m, 6H), 1.20–0.99 (m, 2H), 0.64–0.54 (m, 2H), 0.34 (dt, *J* = 6.0, 4.3 Hz, 2H). ^13^C NMR (100 MHz, DMSO-*d*_6_): δ 161.27, 139.53, 139.36, 131.84, 131.57, 131.38, 129.94, 129.49, 127.37, 60.03, 56.30, 47.49, 47.45, 38.91, 36.38, 29.74, 29.65, 28.10, 25.80, 25.64, 5.76, 4.63, 4.51. HRMS (ESI) *m*/*z*: [M + H]^+^ calcd for C_25_H_33_N_2_OS, 409.2314; found, 409.2323. HPLC: purity = 100%; *R*_t_ = 2.890 min.

#### *N*-(3-(Cyclobutylmethyl)-3-azaspiro­[5.5]­undecan-9-yl)-*N*-phenylthiophene-2-carboxamide Hydrochloride **(53)**

The title compound was prepared following the general procedure as a white powder in 86% yield. ^1^H NMR (400 MHz, DMSO-*d*_6_): δ 9.93 (s, 1H), 7.58 (dd, *J* = 5.1, 1.2 Hz, 1H), 7.48 (dd, *J* = 5.0, 2.0 Hz, 3H), 7.32–7.26 (m, 2H), 6.81 (dd, *J* = 5.1, 3.8 Hz, 1H), 6.36 (dd, *J* = 3.8, 1.2 Hz, 1H), 4.47 (m, 1H), 3.16–2.99 (m, 4H), 2.97–2.66 (m, 3H), 2.14 (dd, *J* = 13.4, 3.1 Hz, 1H), 2.10–1.99 (m, 2H), 1.92–1.56 (m, 8H), 1.49–1.21 (m, 6H), 1.18–1.06 (m, 1H). ^13^C NMR (100 MHz, DMSO-*d*_6_): δ 161.26, 139.53, 139.36, 131.84, 131.56, 131.38, 129.93, 129.49, 127.37, 60.70, 56.29, 47.81, 47.74, 36.36, 30.61, 29.73, 29.46, 28.10, 27.41, 27.24, 25.78, 25.63, 18.56. HRMS (ESI) *m*/*z*: [M + H]^+^ calcd for C_26_H_35_N_2_OS, 423.2470; found, 423.2468. HPLC: purity = 99.21%; *R*_t_ = 2.978 min.

#### *N*-(3-(Cyclopentylmethyl)-3-azaspiro­[5.5]­undecan-9-yl)-*N*-phenylthiophene-2-carboxamide Hydrochloride **(54)**

The title compound was prepared following the general procedure as a white powder in 87% yield. ^1^H NMR (400 MHz, DMSO-*d*_6_): δ 9.84 (s, 1H), 7.58 (dd, *J* = 5.0, 1.2 Hz, 1H), 7.54–7.43 (m, 3H), 7.34–7.25 (m, 2H), 6.81 (dd, *J* = 5.1, 3.8 Hz, 1H), 6.36 (dd, *J* = 3.9, 1.2 Hz, 1H), 4.49 (m, 1H), 3.29–3.18 (m, 2H), 3.03–2.79 (m, 4H), 2.27–2.10 (m, 2H), 1.86–1.67 (m, 5H), 1.59 (m, 3H), 1.52–1.07 (m, 11H). ^13^C NMR (100 MHz, DMSO-*d*_6_): δ 161.27, 139.53, 139.37, 131.83, 131.57, 131.38, 129.93, 129.49, 127.36, 60.89, 56.31, 48.26, 48.16, 38.77, 36.19, 34.89, 31.45, 29.90, 29.50, 27.92, 25.79, 25.63, 25.10, 25.06. HRMS (ESI) *m*/*z*: [M + H]^+^ calcd for C_27_H_37_N_2_OS, 437.2627; found, 437.2635. HPLC: purity = 100%; *R*_t_ = 3.105 min.

#### *N*-(3-(Cyclohexylmethyl)-3-azaspiro­[5.5]­undecan-9-yl)-*N*-phenylthiophene-2-carboxamide Hydrochloride **(55)**

The title compound was prepared following the general procedure as a white powder in 84% yield. ^1^H NMR (400 MHz, DMSO-*d*_6_): δ 9.47 (s, 1H), 7.58 (dd, *J* = 5.0, 1.2 Hz, 1H), 7.49 (dd, *J* = 5.1, 2.0 Hz, 3H), 7.34–7.24 (m, 2H), 6.81 (dd, *J* = 5.0, 3.8 Hz, 1H), 6.36 (dd, *J* = 3.8, 1.2 Hz, 1H), 4.49 (m, 1H), 3.27–3.17 (m, 2H), 2.99–2.78 (m, 4H), 2.14 (dd, *J* = 13.5, 3.1 Hz, 1H), 1.84–1.54 (m, 10H), 1.50–1.05 (m, 10H), 0.92 (td, *J* = 14.5, 13.5, 7.2 Hz, 2H). ^13^C NMR (100 MHz, DMSO-*d*_6_): δ 161.25, 139.53, 139.36, 131.84, 131.57, 131.38, 129.92, 129.48, 127.36, 61.90, 56.26, 48.57, 48.40, 38.70, 36.11, 32.55, 31.11, 29.93, 29.52, 27.85, 25.92, 25.77, 25.60, 25.46, 25.44. HRMS (ESI) *m*/*z*: [M + H]^+^ calcd for C_28_H_39_N_2_OS, 451.2783; found, 451.2787. HPLC: purity = 100%; *R*_t_ = 3.215 min.

#### *N*-(3-Benzyl-3-azaspiro­[5.5]­undecan-9-yl)-*N*-phenylthiophene-2-carboxamide Hydrochloride **(56)**

The title compound was prepared following the general procedure as a white powder in 65% yield. ^1^H NMR (400 MHz, DMSO-*d*_6_): δ 9.83 (s, 1H), 7.58 (dd, *J* = 5.0, 1.2 Hz, 1H), 7.55–7.38 (m, 8H), 7.32–7.24 (m, 2H), 6.81 (dd, *J* = 5.1, 3.8 Hz, 1H), 6.35 (dd, *J* = 3.8, 1.2 Hz, 1H), 4.48 (t, *J* = 12.1 Hz, 1H), 4.24 (d, *J* = 5.4 Hz, 2H), 3.11–2.88 (m, 4H), 2.18–2.09 (m, 1H), 1.68 (m, 4H), 1.43 (d, *J* = 14.6 Hz, 2H), 1.29 (m, 4H), 1.14 (t, *J* = 12.0 Hz, 1H). ^13^C NMR (100 MHz, DMSO-*d*_6_): δ 161.26, 139.52, 139.34, 131.85, 131.76, 131.56, 131.37, 130.37, 129.93, 129.47, 129.24, 127.37, 56.25, 47.73, 29.77, 29.62, 25.81. HRMS (ESI) *m*/*z*: [M + H]^+^ calcd for C_28_H_33_N_2_OS, 445.2314; found, 445.2310. HPLC: purity = 100%; *R*_t_ = 2.997 min.

#### *N*-(3-Phenethyl-3-azaspiro­[5.5]­undecan-9-yl)-*N*-phenylthiophene-2-carboxamide Hydrochloride **(57)**

The title compound was prepared following the general procedure as a white powder in 69% yield. ^1^H NMR (400 MHz, DMSO-*d*_6_): δ 10.09 (s, 1H), 7.58 (dd, *J* = 5.1, 1.2 Hz, 1H), 7.49 (dd, *J* = 5.0, 2.0 Hz, 3H), 7.37–7.28 (m, 4H), 7.25 (td, *J* = 6.3, 1.6 Hz, 3H), 6.82 (dd, *J* = 5.0, 3.8 Hz, 1H), 6.37 (dd, *J* = 3.9, 1.2 Hz, 1H), 4.49 (m, 1H), 3.33–3.18 (m, 4H), 3.05–2.89 (m, 4H), 2.16 (dd, *J* = 13.4, 3.3 Hz, 1H), 1.70 (m, 4H), 1.46 (dt, *J* = 13.3, 3.7 Hz, 2H), 1.41–1.26 (m, 4H), 1.14 (td, *J* = 13.4, 3.2 Hz, 1H). ^13^C NMR (100 MHz, DMSO-*d*_6_): δ 161.27, 139.53, 139.39, 137.61, 131.85, 131.58, 131.37, 129.94, 129.49, 129.14, 129.11, 127.37, 127.27, 56.59, 56.30, 47.93, 38.80, 36.48, 29.96, 29.74, 29.61, 28.25, 25.76, 25.61. HRMS (ESI) *m*/*z*: [M + H]^+^ calcd for C_29_H_35_N_2_OS, 459.2470; found, 459.2474. HPLC: purity = 100%; *R*_t_ = 3.082 min.

#### *N*-(3-Allyl-3-azaspiro­[5.5]­undecan-9-yl)-*N*-phenylthiophene-3-carboxamide Hydrochloride **(58)**

The title compound was prepared following the general procedure as a white powder in 69% yield. ^1^H NMR (400 MHz, DMSO-*d*_6_): δ 10.81 (s, 1H), 7.41–7.32 (m, 3H), 7.28 (dd, *J* = 5.1, 3.0 Hz, 1H), 7.22–7.12 (m, 3H), 6.82–6.75 (m, 1H), 6.06–5.92 (m, 1H), 5.49–5.43 (m, 1H), 5.42 (s, 1H), 4.53–4.38 (m, 1H), 3.65 (t, *J* = 6.1 Hz, 2H), 3.55 (s, 3H), 3.15–3.05 (m, 2H), 2.90 (m, 2H), 2.21–2.09 (m, 1H), 1.72 (m, 4H), 1.50–1.23 (m, 6H), 1.13 (dd, *J* = 14.6, 3.3 Hz, 1H). ^13^C NMR (100 MHz, DMSO-*d*_6_): δ 164.25, 140.18, 138.18, 130.86, 129.47, 129.10, 128.48, 128.26, 125.54, 124.76, 65.38, 57.90, 56.16, 49.05, 47.31, 38.94, 36.35, 29.82, 29.62, 28.07, 25.93, 25.78, 15.64. HRMS (ESI) *m*/*z*: [M + H]^+^ calcd for C_24_H_31_N_2_OS, 395.2157; found, 395.2160. HPLC: purity = 99.78%; *R*_t_ = 2.780 min.

#### *N*-(3-(Cyclopropylmethyl)-3-azaspiro­[5.5]­undecan-9-yl)-*N*-phenylthiophene-3-carboxamide Hydrochloride **(59)**

The title compound was prepared following the general procedure as a white powder in 82% yield. ^1^H NMR (400 MHz, DMSO-*d*_6_): δ 10.42 (s, 1H), 7.43–7.32 (m, 3H), 7.28 (dd, *J* = 5.1, 2.9 Hz, 1H), 7.18 (m, 3H), 6.78 (d, *J* = 5.1 Hz, 1H), 4.45 (dt, *J* = 12.9, 6.9 Hz, 1H), 3.27 (t, *J* = 14.2 Hz, 2H), 2.90 (m, 4H), 2.22–2.10 (m, 1H), 1.85–1.61 (m, 4H), 1.52–1.23 (m, 6H), 1.09 (m, 2H), 0.66–0.55 (m, 2H), 0.36 (t, *J* = 4.8 Hz, 2H). ^13^C NMR (100 MHz, DMSO-*d*_6_): δ 164.25, 140.17, 138.18, 130.89, 129.48, 129.10, 128.48, 125.54, 65.38, 59.95, 56.13, 49.05, 47.40, 47.36, 39.01, 36.34, 29.82, 29.69, 28.05, 25.94, 25.80, 15.64, 5.74, 4.65, 4.53. HRMS (ESI) *m*/*z*: [M + H]^+^ calcd for C_25_H_33_N_2_OS, 409.2314; found, 409.2322. HPLC: purity = 99.55%; *R*_t_ = 2.833 min.

#### *N*-(3-(Cyclobutylmethyl)-3-azaspiro­[5.5]­undecan-9-yl)-*N*-phenylthiophene-3-carboxamide Hydrochloride **(60)**

The title compound was prepared following the general procedure as a white powder in 90% yield. ^1^H NMR (400 MHz, DMSO-*d*_6_): δ 9.95 (s, 1H), 7.40–7.32 (m, 3H), 7.28 (dd, *J* = 5.1, 3.0 Hz, 1H), 7.18 (m, *J* = 7.9, 6.0, 2.5 Hz, 3H), 6.78 (dd, *J* = 5.0, 1.3 Hz, 1H), 4.43 (d, *J* = 12.3 Hz, 1H), 3.16–2.98 (m, 4H), 2.96–2.79 (m, 2H), 2.72 (m, 1H), 2.20–2.10 (m, 1H), 2.10–1.98 (m, 2H), 1.89–1.59 (m, 8H), 1.47–1.22 (m, 6H), 1.17–1.04 (m, 1H). ^13^C NMR (100 MHz, DMSO-*d*_6_): δ 140.16, 138.16, 130.90, 129.48, 129.12, 128.48, 125.55, 60.70, 56.09, 47.81, 47.75, 38.98, 36.37, 30.61, 29.80, 29.48, 28.12, 27.40, 27.24, 25.93, 25.78, 18.56. HRMS (ESI) *m*/*z*: [M + H]^+^ calcd for C_26_H_35_N_2_OS, 423.2470; found, 423.2454. HPLC: purity = 99.91%; *R*_t_ = 2.928 min.

#### *N*-(3-(Cyclopentylmethyl)-3-azaspiro­[5.5]­undecan-9-yl)-*N*-phenylthiophene-3-carboxamide Hydrochloride **(61)**

The title compound was prepared following the general procedure as a white powder in 94% yield. ^1^H NMR (400 MHz, DMSO-*d*_6_): δ 9.63 (s, 1H), 7.40–7.32 (m, 3H), 7.28 (dd, *J* = 5.0, 3.0 Hz, 1H), 7.18 (m, 3H), 6.78 (dd, *J* = 5.1, 1.3 Hz, 1H), 4.44 (d, *J* = 12.2 Hz, 1H), 3.29–3.15 (m, 2H), 3.02–2.82 (m, 4H), 2.28–2.10 (m, 2H), 1.84–1.67 (m, 5H), 1.66–1.54 (m, 3H), 1.53–1.29 (m, 8H), 1.27–1.09 (m, 3H). ^13^C NMR (100 MHz, DMSO-*d*_6_): δ 164.25, 140.17, 138.17, 130.90, 129.47, 129.12, 128.48, 125.55, 60.91, 56.10, 48.31, 48.21, 38.84, 36.24, 34.89, 31.40, 29.96, 29.52, 27.98, 25.94, 25.78, 25.10, 25.06. HRMS (ESI) *m*/*z*: [M + H]^+^ calcd for C_27_H_37_N_2_OS, 437.2627; found, 437.2633. HPLC: purity = 100%; *R*_t_ = 3.020 min.

#### *N*-(3-(Cyclohexylmethyl)-3-azaspiro­[5.5]­undecan-9-yl)-*N*-phenylthiophene-3-carboxamide Hydrochloride **(62)**

The title compound was prepared following the general procedure as a white powder in 74% yield. ^1^H NMR (400 MHz, DMSO-*d*_6_): δ 9.50 (s, 1H), 7.35 (m, 3H), 7.28 (dd, *J* = 5.1, 3.0 Hz, 1H), 7.18 (m, 3H), 6.78 (dd, *J* = 5.1, 1.3 Hz, 1H), 4.53–4.38 (m, 1H), 3.20 (dd, *J* = 23.4, 12.7 Hz, 2H), 3.00–2.91 (m, 1H), 2.89–2.78 (m, 3H), 2.14 (dd, *J* = 13.6, 3.1 Hz, 1H), 1.80–1.55 (m, 10H), 1.52–1.30 (m, 6H), 1.25–1.04 (m, 4H), 1.00–0.84 (m, 2H). ^13^C NMR (100 MHz, DMSO-*d*_6_): δ 164.24, 140.17, 138.17, 130.90, 129.47, 129.12, 128.48, 125.55, 61.91, 56.07, 48.57, 48.42, 38.77, 36.12, 32.56, 31.11, 30.01, 29.54, 27.87, 25.92, 25.75, 25.46, 25.44. HRMS (ESI) *m*/*z*: [M + H]^+^ calcd for C_28_H_39_N_2_OS, 451.2783; found, 451.2797. HPLC: purity = 100%; *R*_t_ = 3.147 min.

#### *N*-(3-Benzyl-3-azaspiro­[5.5]­undecan-9-yl)-*N*-phenylthiophene-3-carboxamide Hydrochloride **(63)**

The title compound was prepared following the general procedure as a white powder in 82% yield. ^1^H NMR (400 MHz, DMSO-*d*_6_): δ 10.32 (s, 1H), 7.56 (dd, *J* = 6.6, 3.0 Hz, 2H), 7.47–7.40 (m, 3H), 7.38–7.32 (m, 3H), 7.27 (dd, *J* = 5.1, 2.9 Hz, 1H), 7.22–7.13 (m, 3H), 6.77 (dd, *J* = 5.1, 1.3 Hz, 1H), 4.43 (d, *J* = 12.1 Hz, 1H), 4.24 (d, *J* = 5.4 Hz, 2H), 3.13–2.83 (m, 4H), 2.14 (d, *J* = 13.5 Hz, 1H), 1.70 (q, *J* = 14.7, 14.3 Hz, 4H), 1.36 (m, 6H), 1.20–1.06 (m, 1H). ^13^C NMR (100 MHz, DMSO-*d*_6_): δ 164.23, 140.14, 138.16, 131.80, 130.88, 130.44, 129.84, 129.46, 129.17, 129.11, 128.48, 125.54, 58.97, 56.06, 47.62, 38.90, 36.23, 29.85, 29.66, 27.97, 25.95, 25.77. HRMS (ESI) *m*/*z*: [M + H]^+^ calcd for C_29_H_35_N_2_OS, 445.2314; found, 445.2289. HPLC: purity = 99.11%; *R*_t_ = 2.948 min.

#### *N*-(3-Phenethyl-3-azaspiro­[5.5]­undecan-9-yl)-*N*-phenylthiophene-3-carboxamide Hydrochloride **(64)**

The title compound was prepared following the general procedure as a white powder in 53% yield. ^1^H NMR (400 MHz, DMSO-*d*_6_): δ 9.88 (s, 1H), 7.41–7.31 (m, 5H), 7.30–7.22 (m, 4H), 7.19 (m, 3H), 6.78 (dd, *J* = 5.0, 1.3 Hz, 1H), 4.47 (d, *J* = 11.9 Hz, 1H), 3.29–3.18 (m, 2H), 3.08–2.86 (m, 4H), 2.15 (d, *J* = 13.6 Hz, 1H), 1.81–1.62 (m, 4H), 1.38 (m, 6H), 1.16 (d, *J* = 13.5 Hz, 1H). ^13^C NMR (100 MHz, DMSO-*d*_6_): δ 138.17, 137.56, 130.90, 129.48, 129.15, 129.12, 128.49, 127.29, 125.56, 56.59, 56.11, 29.99, 29.62, 25.90, 25.75. HRMS (ESI) *m*/*z*: [M + H]^+^ calcd for C_29_H_35_N_2_OS, 459.2470; found, 459.2450. HPLC: purity = 99.09%; *R*_t_ = 3.030 min.

#### *N*-(9-Allylspiro­[5.5]­undecan-3-yl)-*N*-phenyl-1*H*-pyrrole-2-carboxamide Hydrochloride **(65)**

The title compound was prepared following the general procedure as a white solid in 67% yield. ^1^H NMR (400 MHz, DMSO-*d*_6_): δ 11.34 (s, 1H), 10.36 (s, 1H), 7.54–7.40 (m, 3H), 7.31–7.18 (m, 2H), 6.73 (td, *J* = 2.7, 1.4 Hz, 1H), 6.05–5.86 (m, 1H), 5.72 (dt, *J* = 3.8, 2.4 Hz, 1H), 5.46 (s, 1H), 5.43 (d, *J* = 3.2 Hz, 1H), 4.52 (t, *J* = 12.0 Hz, 1H), 4.45 (s, 1H), 3.65 (d, *J* = 12.2 Hz, 2H), 3.23–3.07 (m, 2H), 3.00–2.76 (m, 2H), 2.13 (d, *J* = 13.3 Hz, 1H), 1.68 (m, 4H), 1.44 (d, *J* = 13.5 Hz, 2H), 1.29 (d, *J* = 57.1 Hz, 4H), 1.14 (dd, *J* = 13.7, 3.2 Hz, 1H). ^13^C NMR (100 MHz, DMSO-*d*_6_): δ 160.57, 139.93, 131.41, 129.72, 129.05, 128.15, 125.63, 124.93, 121.64, 112.96, 108.98, 65.38, 57.93, 55.17, 47.40, 38.93, 36.44, 29.80, 29.60, 28.14, 25.95, 25.79, 15.49. HRMS (ESI) *m*/*z*: [M + H]^+^ calcd for C_24_H_32_N_3_O, 378.2545; found, 378.2547. HPLC: purity = 98.42%; *R*_t_ = 2.715 min.

#### *N*-(9-(Cyclopropylmethyl)­spiro­[5.5]­undecan-3-yl)-*N*-phenyl-1*H*-pyrrole-2-carboxamide Hydrochloride **(66)**

The title compound was prepared following the general procedure as a white solid in 92% yield. ^1^H NMR (400 MHz, DMSO-*d*_6_): δ 11.34 (s, 1H), 9.93 (s, 1H), 7.49 (dd, *J* = 5.0, 1.9 Hz, 3H), 7.31–7.17 (m, 2H), 6.74 (td, *J* = 2.7, 1.4 Hz, 1H), 5.72 (dt, *J* = 3.8, 2.5 Hz, 1H), 4.54 (tt, *J* = 12.0, 3.9 Hz, 1H), 4.45 (s, 1H), 3.28 (t, *J* = 15.3 Hz, 2H), 3.02–2.79 (m, 4H), 2.13 (dd, *J* = 13.4, 3.1 Hz, 1H), 1.80–1.58 (m, 4H), 1.44 (dt, *J* = 14.2, 3.1 Hz, 2H), 1.31 (m, 4H), 1.19–1.11 (m, 1H), 1.05 (dd, *J* = 9.0, 4.5 Hz, 1H), 0.65–0.53 (m, 2H), 0.40–0.28 (m, 2H). ^13^C NMR (100 MHz, DMSO-*d*_6_): δ 160.56, 139.91, 131.44, 129.73, 129.06, 125.62, 121.65, 112.97, 108.99, 65.38, 60.05, 55.12, 47.52, 47.47, 38.99, 36.43, 29.79, 29.65, 28.12, 25.96, 25.81, 15.64, 5.76, 4.63, 4.50. HRMS (ESI) *m*/*z*: [M + H]^+^ calcd for C_25_H_34_N_3_O, 392.2702; found, 392.2704. HPLC: purity = 97.22%; *R*_t_ = 2.763 min.

#### *N*-(9-(Cyclobutylmethyl)­spiro­[5.5]­undecan-3-yl)-*N*-phenyl-1*H*-pyrrole-2-carboxamide Hydrochloride **(67)**

The title compound was prepared following the general procedure as a white solid in 95% yield. ^1^H NMR (400 MHz, DMSO-*d*_6_): δ 11.34 (s, 1H), 9.81 (s, 1H), 7.49 (d, *J* = 2.2 Hz, 3H), 7.25 (dd, *J* = 6.3, 3.2 Hz, 2H), 6.73 (d, *J* = 1.5 Hz, 1H), 5.72 (dt, *J* = 3.8, 2.4 Hz, 1H), 4.61–4.49 (m, 1H), 4.45 (s, 1H), 3.16–2.98 (m, 4H), 2.96–2.76 (m, 2H), 2.71 (m, 1H), 2.13 (dd, *J* = 13.3, 3.1 Hz, 1H), 2.10–1.97 (m, 2H), 1.86 (m, 1H), 1.82–1.70 (m, 4H), 1.70–1.55 (m, 3H), 1.42 (m, 2H), 1.37–1.18 (m, 4H), 1.17–1.10 (m, 1H). ^13^C NMR (100 MHz, DMSO-*d*_6_): δ 160.56, 139.91, 131.44, 129.72, 129.06, 125.62, 121.64, 112.97, 108.99, 65.38, 60.72, 55.11, 47.86, 47.77, 38.99, 36.41, 30.60, 29.79, 29.47, 28.13, 27.39, 27.21, 25.95, 25.81, 18.56, 15.64. HRMS (ESI) *m*/*z*: [M + H]^+^ calcd for C_26_H_36_N_3_O, 406.2858; found, 406.2865. HPLC: purity = 98.22%; *R*_t_ = 2.853 min.

#### *N*-(9-(Cyclopentylmethyl)­spiro­[5.5]­undecan-3-yl)-*N*-phenyl-1*H*-pyrrole-2-carboxamide Hydrochloride **(68)**

The title compound was prepared following the general procedure as a white solid in 80% yield. ^1^H NMR (400 MHz, DMSO-*d*_6_): δ 11.35 (s, 1H), 9.48 (s, 1H), 7.49 (dd, *J* = 5.0, 1.9 Hz, 3H), 7.34–7.17 (m, 2H), 6.74 (d, *J* = 1.4 Hz, 1H), 5.78–5.67 (m, 1H), 4.53 (m, 1H), 4.45 (s, 1H), 3.22 (dd, *J* = 24.2, 12.5 Hz, 2H), 2.98 (q, *J* = 3.8, 2.0 Hz, 3H), 2.94–2.77 (m, 2H), 2.26–2.07 (m, 2H), 1.85–1.64 (m, 5H), 1.59 (m, 3H), 1.50 (m, 2H), 1.46–1.32 (m, 4H), 1.32–1.11 (m, 5H). ^13^C NMR (100 MHz, DMSO-*d*_6_): δ 160.56, 139.91, 131.44, 129.72, 129.06, 125.62, 121.65, 112.97, 108.99, 60.93, 55.11, 48.33, 48.25, 38.86, 36.29, 34.88, 31.36, 29.93, 29.50, 28.00, 25.96, 25.80, 25.09, 25.05. HRMS (ESI) *m*/*z*: [M + H]^+^ calcd for C_27_H_38_N_3_O, 420.3015; found, 420.3006. HPLC: purity = 99.10%; *R*_t_ = 2.935 min.

#### *N*-(9-(Cyclohexylmethyl)­spiro­[5.5]­undecan-3-yl)-*N*-phenyl-1*H*-pyrrole-2-carboxamide Hydrochloride **(69)**

The title compound was prepared following the general procedure as a white solid in 78% yield. ^1^H NMR (400 MHz, DMSO-*d*_6_): δ 11.34 (d, *J* = 3.4 Hz, 1H), 9.46 (s, 1H), 7.49 (dd, *J* = 5.0, 1.9 Hz, 3H), 7.33–7.19 (m, 2H), 6.74 (td, *J* = 2.8, 1.4 Hz, 1H), 5.72 (dt, *J* = 3.8, 2.5 Hz, 1H), 4.53 (m, 1H), 4.45 (m, 1H), 3.20 (dd, *J* = 25.1, 12.5 Hz, 2H), 2.93 (m, 1H), 2.84 (t, *J* = 5.8 Hz, 3H), 2.14 (dd, *J* = 13.5, 3.1 Hz, 1H), 1.80–1.52 (m, 10H), 1.51–1.30 (m, 5H), 1.29–1.05 (m, 6H), 1.00–0.84 (m, 2H). ^13^C NMR (100 MHz, DMSO-*d*_6_): δ 160.55, 139.91, 131.44, 129.71, 129.06, 125.62, 121.65, 112.97, 108.99, 61.91, 55.09, 48.57, 48.43, 38.78, 36.15, 32.55, 31.10, 29.99, 29.52, 27.84, 25.92, 25.77, 25.46, 25.44. HRMS (ESI) *m*/*z*: [M + H]^+^ calcd for C_28_H_40_N_3_O, 434.3171; found, 434.3171. HPLC: purity = 97.04%; *R*_t_ = 3.057 min.

#### *N*-(9-Benzylspiro­[5.5]­undecan-3-yl)-*N*-phenyl-1*H*-pyrrole-2-carboxamide Hydrochloride **(70)**

The title compound was prepared following the general procedure as a white solid in 84% yield. ^1^H NMR (400 MHz, DMSO-*d*_6_): δ 11.33 (s, 1H), 9.99 (s, 1H), 7.56–7.50 (m, 2H), 7.50–7.40 (m, 6H), 7.25 (dd, *J* = 6.4, 3.2 Hz, 2H), 6.73 (d, *J* = 1.4 Hz, 1H), 5.72 (dt, *J* = 3.9, 2.5 Hz, 1H), 4.58–4.47 (m, 1H), 4.44 (s, 1H), 4.24 (d, *J* = 5.5 Hz, 2H), 3.14–2.84 (m, 5H), 2.13 (d, *J* = 13.2 Hz, 1H), 1.77–1.58 (m, 4H), 1.43 (d, *J* = 14.1 Hz, 2H), 1.38–1.20 (m, 4H), 1.19–1.11 (m, 1H). ^13^C NMR (100 MHz, DMSO-*d*_6_): δ 160.55, 139.88, 131.78, 131.43, 130.38, 129.90, 129.71, 129.21, 129.04, 125.61, 121.64, 112.97, 108.99, 59.05, 55.07, 47.68, 38.91, 36.30, 29.83, 29.63, 28.01, 25.98, 25.79. HRMS (ESI) *m*/*z*: [M + H]^+^ calcd for C_28_H_34_N_3_O, 428.2702; found, 428.2708. HPLC: purity = 97.61%; *R*_t_ = 2.865 min.

#### *N*-(9-Phenethylspiro­[5.5]­undecan-3-yl)-*N*-phenyl-1*H*-pyrrole-2-carboxamide Hydrochloride **(71)**

The title compound was prepared following the general procedure as a white solid in 60% yield. ^1^H NMR (400 MHz, DMSO-*d*_6_): δ 11.35 (q, *J* = 2.8 Hz, 1H), 10.13 (s, 1H), 7.49 (dd, *J* = 4.9, 2.0 Hz, 3H), 7.39–7.30 (m, 2H), 7.25 (ddd, *J* = 8.0, 5.9, 2.1 Hz, 5H), 6.74 (td, *J* = 2.8, 1.4 Hz, 1H), 5.72 (q, *J* = 2.6 Hz, 1H), 4.53 (m, 1H), 4.45 (d, *J* = 3.5 Hz, 1H), 3.37–3.18 (m, 4H), 3.08–2.85 (m, 4H), 2.22–2.07 (m, 1H), 1.80–1.60 (m, 4H), 1.54–1.41 (m, 2H), 1.41–1.19 (m, 4H), 1.19–1.08 (m, 1H). ^13^C NMR (100 MHz, DMSO-*d*_6_): δ 160.57, 139.94, 137.62, 131.43, 129.72, 129.14, 129.11, 129.06, 127.27, 125.63, 121.65, 112.98, 108.99, 56.60, 55.14, 47.90, 38.89, 36.50, 29.95, 29.79, 29.61, 28.23, 25.92, 25.78. HRMS (ESI) *m*/*z*: [M + H]^+^ calcd for C_29_H_36_N_3_O, 442.2858; found, 442.2861. HPLC: purity = 98.34%; *R*_t_ = 2.935 min.

#### *N*-(3-Allyl-3-azaspiro­[5.5]­undecan-9-yl)-*N*-phenyl-1*H*-pyrrole-3-carboxamide Hydrochloride **(72)**

The title compound was prepared following the general procedure as a white solid in 49% yield. ^1^H NMR (400 MHz, DMSO-*d*_6_): δ 10.79 (d, *J* = 89.3 Hz, 2H), 7.59–7.36 (m, 3H), 7.33–7.16 (m, 2H), 6.46 (q, *J* = 2.4 Hz, 1H), 6.00 (s, 1H), 5.67–5.53 (m, 1H), 5.53–5.36 (m, 2H), 4.63–4.41 (m, 1H), 3.57 (d, *J* = 45.8 Hz, 4H), 2.99–2.77 (m, 2H), 2.18–2.06 (m, 1H), 1.83–1.56 (m, 4H), 1.32 (m, 6H), 1.10 (t, *J* = 14.2 Hz, 1H). ^13^C NMR (100 MHz, DMSO-*d*_6_): δ 164.18, 140.77, 131.46, 131.22, 129.87, 129.51, 128.59, 128.18, 124.87, 122.31, 119.61, 117.64, 110.29, 57.92, 55.11, 49.05, 47.39, 39.06, 36.46, 29.90, 29.61, 28.14, 26.07, 25.90. HRMS (ESI) *m*/*z*: [M + H]^+^ calcd for C_24_H_32_N_3_O, 378.2545; found, 378.2549. HPLC: purity = 95.14%; *R*_t_ = 2.575 min.

#### *N*-(3-(Cyclopropylmethyl)-3-azaspiro­[5.5]­undecan-9-yl)-*N*-phenyl-1*H*-pyrrole-3-carboxamide Hydrochloride **(73)**

The title compound was prepared following the general procedure as a white solid in 58% yield. ^1^H NMR (400 MHz, DMSO-*d*_6_): δ 10.98–10.82 (m, 1H), 10.17 (d, *J* = 48.9 Hz, 1H), 7.53–7.38 (m, 3H), 7.20 (dd, *J* = 6.6, 2.9 Hz, 2H), 6.46 (q, *J* = 2.4 Hz, 1H), 6.06–5.89 (m, 1H), 5.60 (q, *J* = 2.3 Hz, 1H), 4.51 (ddd, *J* = 11.9, 8.1, 3.7 Hz, 1H), 3.27 (t, *J* = 14.7 Hz, 2H), 2.99–2.82 (m, 4H), 2.20–2.08 (m, 1H), 1.69 (td, *J* = 24.6, 18.4, 8.9 Hz, 4H), 1.45–1.23 (m, 6H), 1.16–1.04 (m, 2H), 0.59 (d, *J* = 7.5 Hz, 2H), 0.35 (t, *J* = 4.9 Hz, 2H). ^13^C NMR (100 MHz, DMSO-*d*_6_): δ 164.18, 140.76, 131.48, 129.52, 128.60, 122.31, 119.60, 117.64, 110.30, 60.01, 55.08, 49.05, 47.48, 47.43, 39.12, 36.44, 29.89, 29.68, 28.11, 26.08, 25.92, 5.75, 4.63, 4.51. HRMS (ESI) *m*/*z*: [M + H]^+^ calcd for C_25_H_34_N_3_O_2_, 392.2702; found, 392.2684. HPLC: purity = 99.50%; *R*_t_ = 2.593 min.

#### *N*-(3-(Cyclobutylmethyl)-3-azaspiro­[5.5]­undecan-9-yl)-*N*-phenyl-1*H*-pyrrole-3-carboxamide Hydrochloride **(74)**

The title compound was prepared following the general procedure as a white solid in 51% yield. ^1^H NMR (400 MHz, DMSO-*d*_6_): δ 10.91 (s, 1H), 10.17 (s, 1H), 7.53–7.40 (m, 3H), 7.20 (dt, *J* = 5.9, 3.4 Hz, 2H), 6.46 (q, *J* = 2.4 Hz, 1H), 6.00 (q, *J* = 2.2 Hz, 1H), 5.59 (q, *J* = 2.3 Hz, 1H), 4.51–4.49 (m, 1H), 3.12–3.00 (m, 4H), 2.92–2.71 (m, 3H), 2.18–2.10 (m, 1H), 2.05 (m, 2H), 1.87–1.58 (m, 9H), 1.40–1.20 (m, 6H), 1.10 (dd, *J* = 13.7, 3.4 Hz, 1H). ^13^C NMR (100 MHz, DMSO-*d*_6_): δ 164.18, 140.75, 131.47, 129.52, 128.60, 122.30, 119.59, 117.64, 110.29, 60.69, 55.08, 49.05, 47.80, 47.70, 39.11, 36.39, 30.60, 29.89, 29.49, 28.08, 27.44, 27.27, 26.06, 25.92, 18.56. HRMS (ESI) *m*/*z*: [M + H]^+^ calcd for C_26_H_36_N_3_O_2_, 406.2858; found, 406.2842. HPLC: purity = 99.40%; *R*_t_ = 2.647 min.

#### *N*-(3-(Cyclopentylmethyl)-3-azaspiro­[5.5]­undecan-9-yl)-*N*-phenyl-1*H*-pyrrole-3-carboxamide Hydrochloride **(75)**

The title compound was prepared following the general procedure as a white solid in 46% yield. ^1^H NMR (400 MHz, DMSO-*d*_6_): δ 10.90 (s, 1H), 9.76 (s, 1H), 7.51–7.39 (m, 3H), 7.20 (dd, *J* = 6.6, 3.0 Hz, 2H), 6.46 (q, *J* = 2.4 Hz, 1H), 6.01 (t, *J* = 2.3 Hz, 1H), 5.59 (q, *J* = 2.4 Hz, 1H), 4.50 (s, 1H), 4.06 (s, 4H), 3.24 (d, *J* = 12.2 Hz, 1H), 3.05–2.78 (m, 4H), 2.29–2.08 (m, 2H), 1.78 (m, 3H), 1.68–1.57 (m, 4H), 1.52–1.46 (m, 2H), 1.44–1.35 (m, 3H), 1.27–1.18 (m, 3H), 1.12 (dd, *J* = 13.6, 3.4 Hz, 1H). ^13^C NMR (100 MHz, DMSO-*d*_6_): δ 164.19, 140.76, 131.47, 129.51, 128.60, 122.31, 119.60, 117.63, 110.29, 60.92, 55.08, 48.30, 48.22, 38.97, 36.29, 34.89, 31.42, 30.05, 29.53, 27.97, 26.07, 25.91, 25.10, 25.05. HRMS (ESI) *m*/*z*: [M + H]^+^ calcd for C_27_H_38_N_3_O_2_, 420.3015; found, 420.3009. HPLC: purity = 97.85%; *R*_t_ = 2.700 min.

#### *N*-(3-(Cyclohexylmethyl)-3-azaspiro­[5.5]­undecan-9-yl)-*N*-phenyl-1*H*-pyrrole-3-carboxamide Hydrochloride **(76)**

The title compound was prepared following the general procedure as a white solid in 45% yield. ^1^H NMR (400 MHz, DMSO-*d*_6_): δ 10.90 (s, 1H), 9.64–9.46 (m, 1H), 7.49–7.39 (m, 3H), 7.20 (dd, *J* = 6.6, 3.0 Hz, 2H), 6.46 (q, *J* = 2.4 Hz, 1H), 6.00 (d, *J* = 2.9 Hz, 1H), 5.59 (q, *J* = 2.3 Hz, 1H), 4.51 (m, 1H), 3.20 (dd, *J* = 24.1, 12.5 Hz, 2H), 2.94–2.79 (m, 4H), 2.17–2.08 (m, 1H), 1.81–1.56 (m, 11H), 1.47–1.33 (m, 4H), 1.24–1.09 (m, 5H), 0.93 (t, *J* = 12.4 Hz, 2H).^13^C NMR (100 MHz, DMSO-*d*_6_): δ 164.17, 140.76, 131.48, 129.51, 128.60, 122.31, 119.60, 117.64, 110.29, 61.91, 55.04, 48.56, 48.43, 38.89, 36.16, 32.56, 31.13, 30.10, 29.55, 27.85, 26.05, 25.92, 25.45, 15.63. HRMS (ESI) *m*/*z*: [M + H]^+^ calcd for C_28_H_40_N_3_O, 434.3171; found, 434.3177. HPLC: purity = 99.16%; *R*_t_ = 2.778 min.

#### *N*-(3-Benzyl-3-azaspiro­[5.5]­undecan-9-yl)-*N*-phenyl-1*H*-pyrrole-3-carboxamide Hydrochloride **(77)**

The title compound was prepared following the general procedure as a white solid in 38% yield. ^1^H NMR (400 MHz, DMSO-*d*_6_): δ 10.91 (s, 1H), 10.55 (s, 1H), 7.59 (dt, *J* = 7.0, 3.3 Hz, 2H), 7.42 (q, *J* = 3.1 Hz, 6H), 7.19 (dd, *J* = 6.7, 2.8 Hz, 2H), 6.45 (t, *J* = 2.4 Hz, 1H), 6.00 (q, *J* = 2.1 Hz, 1H), 5.59 (q, *J* = 2.3 Hz, 1H), 4.57–4.42 (m, 1H), 4.23 (d, *J* = 5.5 Hz, 2H), 3.11–2.84 (m, 4H), 2.19–2.07 (m, 1H), 1.82–1.58 (m, 4H), 1.45–1.19 (m, 6H), 1.12 (d, *J* = 8.9 Hz, 1H).^13^C NMR (100 MHz, DMSO-*d*_6_): δ 164.17, 140.72, 131.83, 131.45, 130.47, 129.79, 129.50, 129.13, 128.58, 122.30, 119.59, 117.63, 110.29, 65.38, 58.91, 55.05, 47.54, 39.03, 36.24, 29.93, 29.67, 27.92, 26.08, 25.90, 15.63. HRMS (ESI) *m*/*z*: [M + H]^+^ calcd for C_28_H_34_N_3_O, 428.2702; found, 428.2702. HPLC: purity = 98.00%; *R*_t_ = 2.648 min.

#### *N*-(3-Phenethyl-3-azaspiro­[5.5]­undecan-9-yl)-*N*-phenyl-1*H*-pyrrole-3-carboxamide Hydrochloride **(78)**

The title compound was prepared following the general procedure as a white solid in 43% yield. ^1^H NMR (400 MHz, DMSO-*d*_6_): δ 11.16–10.50 (m, 2H), 7.44 (d, *J* = 4.7 Hz, 3H), 7.37–7.01 (m, 7H), 6.46 (s, 1H), 6.01 (s, 1H), 5.60 (s, 1H), 4.50 (s, 1H), 3.42–3.13 (m, 4H), 3.11–2.82 (m, 4H), 2.15 (d, *J* = 13.2 Hz, 1H), 1.70 (m, 4H), 1.52–1.02 (m, 7H).^13^C NMR (100 MHz, DMSO-*d*_6_): δ 164.19, 140.78, 137.77, 131.46, 129.51, 129.11, 129.09, 128.59, 127.21, 122.30, 119.60, 117.63, 110.29, 56.59, 55.12, 47.78, 39.04, 36.47, 29.87, 29.65, 28.17, 26.03, 25.90. HRMS (ESI) *m*/*z*: [M + H]^+^ calcd for C_29_H_36_N_3_O, 442.2858; found, 442.2856. HPLC: purity = 97.21%; *R*_t_ = 2.702 min.

#### Biological Evaluations Drugs

Morphine (morphine sulfate pentahydrate salt) was purchased from Mallinckrodt (St. Louis, MO) or provided by the National Institute of Drug Abuse. All drugs and tested compounds were dissolved in pyrogen-free isotonic saline (Baxter Healthcare, Deerfield, IL) or sterile-filtered distilled/deionized water. All other reagents were purchased from either Sigma-Aldrich or Thermo Fisher.

#### Animals

Male Swiss–Webster mice (23–35 g, 7–8 weeks, Harlan Laboratories, Indianapolis, IN) were housed five to a cage in animal care quarters maintained at 22 °C on a 12 h light/dark cycle with food and water available ad libitum. All procedures were approved by the Institutional Animal Care and Use Committee (IACUC, Animal Welfare Assurance Number D16-00180) at Virginia Commonwealth University Medical Center and were conducted in accordance with the recommendations of the International Association for the Study of Pain (IASP).

#### Warm Water Immersion Assay

6–8 week 25–35 g male Swiss Webster mice were housed in cages (five maximal per cage) in animal care quarters and maintained at 22 ± 2 °C on a 12 h light–dark cycle. Food (standard chow) and water were available ad libitum. The mice were brought to the laboratory (22 ± 2 °C, 12 h light–dark cycle) and allowed 18 h to recover from the transport. The tail-flick test was performed using a water bath with the temperature maintained at 56 ± 0.1 °C. Each mouse was gently wrapped in a cloth with only the tail exposed. Baseline latency was measured before s.c. injection of the compounds. The distal one-third of the tail was immersed perpendicularly in water, and the mouse rapidly flicked his tail from the bath at the first sign of discomfort. The duration of time the tail remained in the water bath was counted as the baseline latency. Untreated mice with baseline latency reaction times ranging from 2 to 4 s were used. Test latency was obtained 20 min later after the agonist injection. A 10 s maximum cutoff latency was used to prevent any tissue damage. Antinociception was quantified as the percentage of maximal possible effect (% MPE), which was calculated as % MPE = [(test latency – control latency)/(10 – control latency)] × 100. The % MPE value was calculated for each mouse using six mice per compound. If the compound was evaluated for its antagonizing effects against morphine or fentanyl, the compound was s.c. injected 5 min prior to the agonist administration.

#### Calcium Mobilization Assay

mMOR-CHO cells were cultured with DMEM/F-12 supplemented with 10% FBS at 37 °C and 5% CO_2_. The cells were transfected with Gqi5 cDNA using lipofectamine 2000 medium OPTI according to the manufacturer’s recommended procedure. Then, the cells were incubated for 4 h before being plated to a clear bottom, black 96-well assay plate at 15,000 cells/well in cell growth media. Cells were ready for calcium mobilization assay after 16–20 h incubation. 50 μL of loading buffer was added to each well in the assay plate followed by 1 h incubation. The positive control and varying concentrations of the testing compound were added to a source plate (for antagonist measurement, 20 μL of the testing compound was then added to each well and incubated for another 15 min). Before the measurement, the loading buffer was decanted, and 80 μL/well of the washing buffer was added to the 96-well plate. Subsequently, the assay plates were read on a FlexStation3 microplate reader at 494/516 ex/em. The changes in fluorescence were monitored, and peak height values were obtained using SoftMaxPro software (Molecular Devices). Nonlinear regression curves and IC_50_ values were generated using Graph Pad Prism 8.0. All concentrations were tested in triplicate, and all experiments were repeated at least three times.

#### Measurement of Respiration

6–8 weeks 25–35 g male Swiss Webster mice were housed in cages (five maximal per cage) in animal care quarters and were maintained at 22 ± 2 °C on a reversed 12 h dark–light cycle. All experiments were conducted in the dark (active) phase. Respiration was measured using WBP chambers (EMKA Technologies, France) in freely moving mice. The chambers were supplied with an air mixture containing 5% CO_2_. A 10 min baseline respiration period was recorded prior to any administration. The rate and depth of respiration were recorded and averaged over 1 to 5 min periods. Tidal volume was calculated from the raw inspiration data and expiration data. Minute volume was then calculated as rate × tidal volume. The first compound was administered s.c., and respiration was recorded for 5 min. Then, respiration was recorded for a period of 30 min after the second injection.

### ADMET

#### In Vitro Metabolism

*Intrinsic Clearance* (microsomes, S9, cryopreserved hepatocytes, recombinant CYP, recombinant UGT).

Metabolic stability, expressed as percent of the parent compound remaining, was calculated by comparing the peak area of the compound at the time point relative to that at time-0. The half-life (*T*_1/2_) was estimated from the slope of the initial linear range of the logarithmic curve of compound remaining (%) vs time, assuming the first-order kinetics. The apparent intrinsic clearance (CLint, in μL/min/pmol, μL/min/mg or μL/min/Mcell) was calculated according to the following formula (eq 1)­

1 $${CL}_{\mathrm{int}}=\frac{0.693}{T_{1/2}\left(mg\;protein/\mu L\;\text{or}\;million\;cells/\mu L\;\text{or}\;pmol\;CYP\;isoyme/\mu L\right)}$$

#### Permeability

The apparent permeability coefficient (*P*_app_) of the test compound was calculated as follows (eq 2)­

2 $$P_{app}\left(cm/s\right)=\frac{V_{R\;}\times C_{R,end}}{\Delta t}\times \frac{1}{A\times \left(C_{D,mid}\right)-C_{R,mid}}$$

where *V*_R_ is the volume of the receiver chamber. *C*_R,end_ is the concentration of the test compound in the receiver chamber at the end time point, Δ*t* is the incubation time, and *A* is the surface area of the cell monolayer. *C*_D,mid_ is the calculated midpoint concentration of the test compound in the donor side, which is the mean value of the donor concentration at time 0 min and the donor concentration at the end time point. *C*_R,mid_ is the midpoint concentration of the test compound in the receiver side, which is one-half of the receiver concentration at the end time point. Concentrations of the test compound were expressed as peak areas of the test compound.

Recovery of the test compound from the permeability assay. The recovery of the test compound was calculated as follows (eq 3)­

3 $$Recovery\;\left(\%\right)=\frac{V_{D}\times C_{D,end}+V_{R}\times C_{R,end}}{V_{D}\times C_{D0}}\times 100$$

where *V*_D_ and *V*_R_ are the volumes of the donor and receiver chambers, respectively. *C*_D,end_ is the concentration of the test compound in the donor sample at the end time point. *C*_R,end_ is the concentration of the test compound in the receiver sample at the end time point. *C*_D0_ is the concentration of the test compound in the donor sample at time zero. Concentrations of the test compound are expressed as peak areas of the test compound.

Fluorescein assessment for Permeability assays. Fluorescein was used as the cell monolayer integrity marker. Fluorescein permeability assessment (in the A–B direction at pH 7.4 on both sides) was performed after the permeability assay for the test compound. The cell monolayer that had a fluorescein permeability of less than 1.5 × 10^–6^ cm/s for Caco-2 and MDR1-MDCKII cells and 2.5 × 10^–6^ cm/s for MDCKII cells was considered intact, and the permeability result of the test compound from the intact cell monolayer is reported.

#### Efflux Transporter Substrate Assessment

The ER was calculated as follows (eq 4)­

4 $$ER=\frac{P_{app}\left(B-A\right)}{P_{app}\left(A-B\right)}$$

where *P*_app_(*B–A*) is the apparent permeability coefficient in the *B* to *A* direction, and *P*_app_(*A–B*) is the apparent permeability coefficient in the *A* to *B* direction. A compound is considered as a substrate of an efflux transporter if the ER ≥2; and the transporter selective inhibitor inhibits the ER more than 50%.

#### In Vivo BBB Penetration Studies

Following our previously reported protocol,^31,32^ Swiss Webster mice (three mice each time point) were given compound **5** (10 mg/kg, s.c.). At 5-, 10-, 30-, and 60 min time points post administration, the mice were decapitated, and whole brain and blood samples were collected. Brain samples were washed with saline to ensure removal of any blood on the isolated brains. They were then immersed in 300 μL of saline. Blood samples were centrifuged for 10 min at 15,000*g* at 4 °C following which plasma was collected. Brain and plasma samples were stored at −80 °C until further analysis.

##### UPLC-MS/MS Analysis

The identification and quantification of compound **5** in mouse plasma and brain were performed using a modification of a previously described method with naloxone-*d*_5_ as the internal standard (ISTD).^34^ Prior to extraction, brain tissues were homogenized with deionized water at a 1:3 (w/w) ratio using an Omni Bead Ruptor (Omni International Inc., Kennesaw, GA). Each analytical run included seven-point calibration curves (10–1000 ng/mL or ng/g) for compound 5, quality control samples at 30, 300, and 750 ng/mL or ng/g, as well as negative and blank controls, all prepared in plasma or brain homogenate. An ISTD containing 10 ng of naloxone-*d*_5_ in methanol was added to 100 μL of plasma or 400 μL of brain homogenate for each calibrator, control, and specimen. After mixing, 100 μL of 5 M ammonium hydroxide and 2 mL of a 25:75 methylene chloride-diethyl ether mixture was added. Samples were vortexed for 2 min and centrifuged at 3000 rpm for 5 min. The organic layer was evaporated under nitrogen and reconstituted with 100 μL of the mobile phase before LC–MS/MS analysis. Chromatography was performed on a Sciex ExionLC 2.0+ system coupled to a Sciex 6500 QTRAP with an IonDrive Turbo V source (Sciex, Ontario, Canada), using a Zorbax Eclipse column (4.6 × 75 mm, 3.5 μm; Agilent, USA) and an isocratic mobile phase of 10 mM ammonium formate: methanol (50:50, v/v) at 0.6 mL/min. Source conditions included a temperature of 600 °C, curtain gas at 30 mL/min, ion spray voltage of 5000 V, and ion source gases 1 and 2 at 50 and 30 mL/min, respectively. Data were acquired in positive-ion mode using multiple reaction monitoring with the following transitions (*m*/*z*), and collision energy (eV) in parentheses: compd. **5**, 367 > 180 (28) and 367 > 98 (33); naloxone-*d*_5_, 333 > 212 (45), 333 > 315 (25), and 333 > 273. Total run time was 4 min. Quantification was performed using linear regression of analyte-to-ISTD peak area ratios from the calibration curves.

##### Statistical Analysis

One-way ANOVA followed by the posthoc Dunnett test was performed to assess significance using Prosm 6.0 software (GraphPad Software, San Diago, CA).

### Molecular Modeling Study

Molecular docking studies were performed to elucidate the binding mode of phenyl fentanyl and compound 5 with the inactive mu-opioid receptor (MOR) crystal structure, to provide deeper understandings of their mechanism of action. The compounds were initially sketched using Sybyl X2.1, with Gasteiger–Hückel charges assigned, followed by energy minimization (100,000 iterations) to a gradient of 0.05 kcal/(mol·Å) using the Tripos Force Field. The X-ray crystal structure of the antagonist-bound MOR (PDB ID: 4DKL)^16^ was retrieved from the Protein Data Bank (http://www.rcsb.org). To prepare the receptor for docking, hydrogen atoms were added, water molecules and bound ligands were removed, and missing residues in the intracellular loop 3 (ICL-3) region were modeled using Sybyl 8.0 (Tripos, MO, USA). Molecular docking studies were performed using the genetic algorithm-based docking program GOLD 2020.^35^ Consistent with known opioid ligands, the carboxylate group of D147 (Ballesteros-Weinstein numbering)^36^ in the orthosteric site formed an ionic interaction with the protonated nitrogen atom of the ligand’s amino group. The binding site was defined as atoms within 10 Å of the γ-carbon atom of D147, with a distance constraint applied between the quaternary amine of the ligand and the carboxylate group of D147. The docking results were analyzed based on the highest CHEM-PLP scores, with molecular interactions visualized and analyzed using the PyMOL Molecular Graphics System.

## Supplementary Material



## Notes

Ca^2+^ mobilization assays for Compound **5**; molecular docking for phenylfentanyl and compound **5** in inactive MOR; effects of compound 5 on fentanyl-induced respiratory depression; NMR and HRMS spectra for intermediates and compounds **1**–**78**; and HPLC data and chromatograms (PDF)

## ABBERVIATIONS

BBB: blood–brain barrier
BCRP: breast cancer resistance protein
CNS: central nervous system
DAMGO: [d-Ala^2^, *N*-MePhe^4^, ECL, extracellular loop; Gly­(ol)]- enkephalin
DOR: δ opioid receptor
HPLC: high-performance liquid chromatography
HRMS: high resolution mass spectroscopy
KOR: κ opioid receptor
MOR: μ opioid receptor
NTX: naltrexone
OUD: opioid use disorder
% MPE: percentage maximum possible effect
*P*-gp: *P*-glycoprotein
SAR: structure*–*activity relationship
TM: transmembrane
WBP: whole-body plethysmography
