# Supplementary material for: Structural Determinants of μ‑Opioid Receptor Antagonism and Respiratory Liability in Phenylfentanyl Analogues
Source: ACS Chem Neurosci. 2026 Apr 30;17(10):1888–908. doi: 10.1021/acschemneuro.5c00836 (PMC13195661; doi:10.1021/acschemneuro.5c00836)
Supplement: Supplementary file 1 [file cn5c00836_si_001.pdf]

Structural determinants of μ-opioid receptor antagonism and respiratory liability in phenylfentanyl analogues

Ennian Li[a], Balaji S. Kale[a], Abeje A. Silte[a], Rui Lyu[a], Neha Upadhyay[a], Logan T. Neel[a], Ahmed Reda[a], Huiqun Wang[a], William L. Dewey[b], Piyusha P. Pagare[a,c], Yan Zhang[a,b,c,d,#]

[a] Department of Medicinal Chemistry, School of Pharmacy, Virginia Commonwealth University, 800 East Leigh Street, Richmond, Virginia 23298, United States

[b] Department of Pharmacology and Toxicology, School of Medicine, Virginia Commonwealth University, 410 North 12th Street, Richmond, Virginia 23298, United States

[c] Center for Drug Discovery, Virginia Commonwealth University, 800 East Leigh Street, Richmond, Virginia 23298, United States

[d] Institute for Drug and Alcohol Studies, Virginia Commonwealth University, 203 East Cary Street, Richmond, Virginia 23298, United States

[#] Corresponding author information: **Yan Zhang**; orcid.org/0000-0001-8934-7016

Phone: +1(804)828-0021; Email: yzhang2@vcu.edu

1. Calcium mobilization assays for compound **5** 3
2. Molecular docking for phenylfentanyl and compd. **5** in inactive MOR. 3
3. Effects of compd. **5** on fentanyl-induced respiratory depression.......... 5
4. NMR and HRMS spectrum for intermediates **b**, **c1-c6**, **f**, **g1-g6**, and compounds **1-78**. 7
5. HPLC data and spectrum. 167

1. Calcium mobilization assays for compound **5**

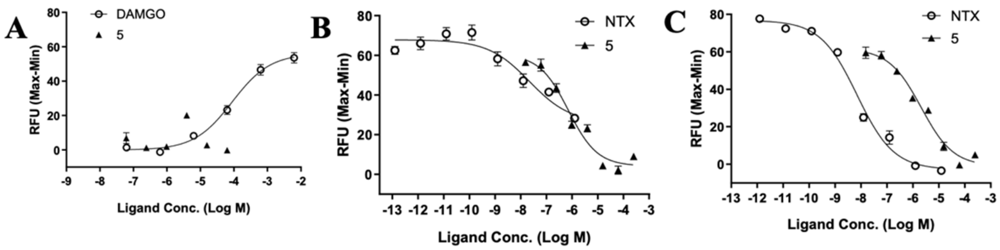

Figure S1. Calcium mobilization assays for compound **5**. (A) Dose response agonism activity of **5**, with DAMGO as positive control; (B) Dose response antagonism against DAMGO, with naltrexone as control; (C) Dose response antagonism against fentanyl, with naltrexone as control. Assay was performed in triplicates against DAMGO (500 nM), Fentanyl (250 nM). NTX, Naltrexone.

2. Molecular docking for phenylfentanyl and compd. **5** in inactive MOR.

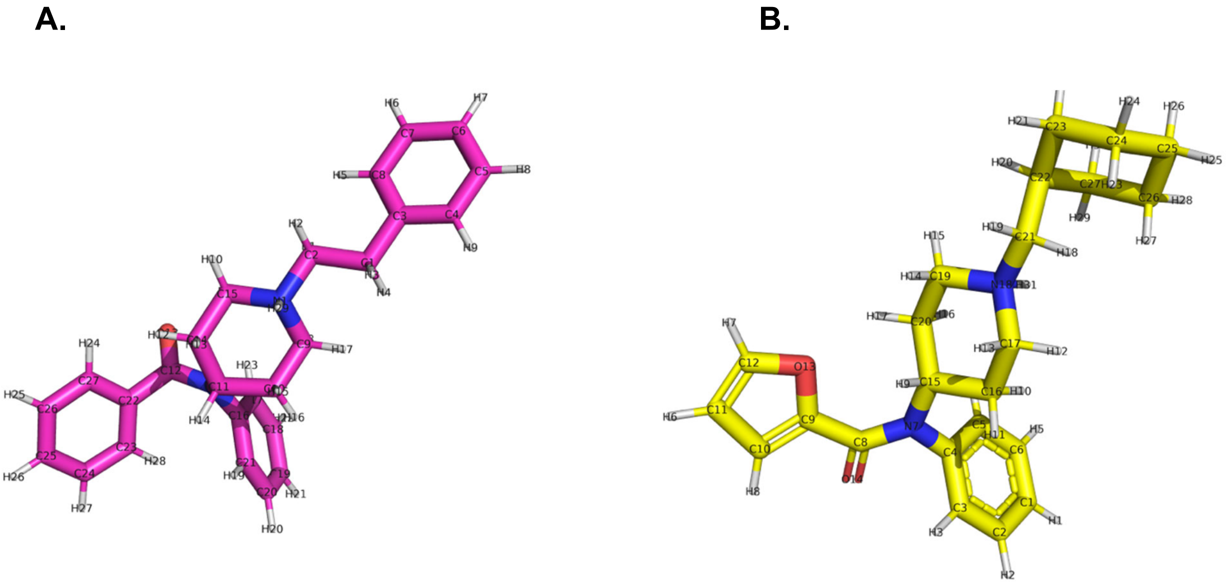

Figure S2. The three-dimension chemical structure of A. Phenyl-fentanyl and B. compound **5** with atom notation after molecular docking.

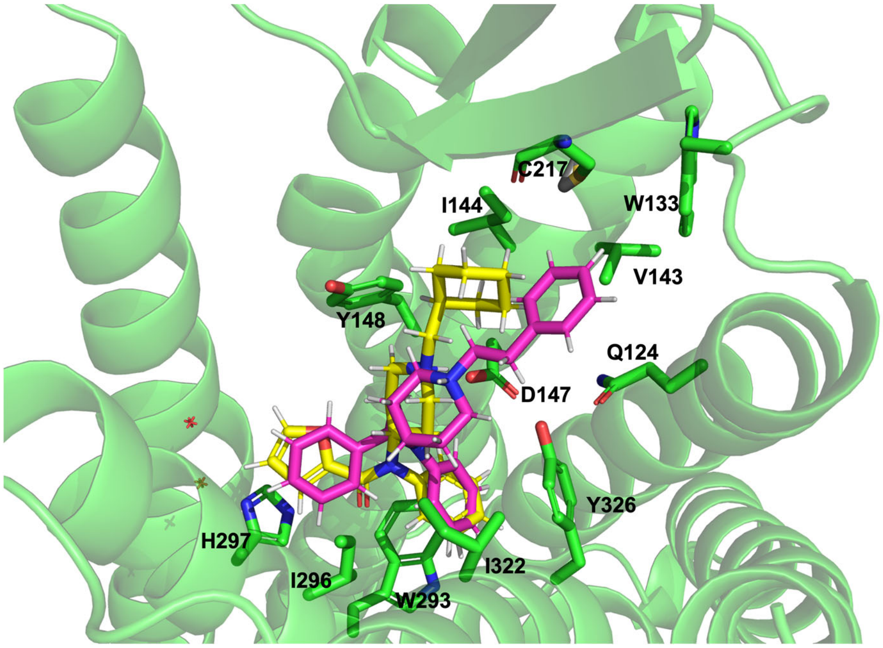

Figure S3. Alignment of the binding pose of compound Phenyl fentanyl and compound 5 at the inactive MOR (4DKL). The MOR is shown as cartoons. Phenyl fentanyl, compound 5, and key amino acid residues are shown on the sticks. Carbon atoms: phenyl fentanyl (magenta) compound 5 (yellow); key amino residues for (green); oxygen atoms (red); nitrogen atoms (blue)

Table S1. Measured Shortest Distances between Atoms on Critical Amino Acid Residues and Atoms on the Ligand with MOR after docking

Ligand	Atom of ligand	Atom of residue	Distance after docking (Å)
Phenyl fentanyl	C06	CZ3@W133	4.2
	C04	CD@Q124	3.8
	C06	CB2@C217	4.2
	C06	CG1@V143	4.0
	C07	CG1@I144	3.6
	N01	OD2@D147	3.8
	C15	CE1@Y148	3.7
	C18	CH2@W293	3.6
	C25	CE1@H297	4.1
	C21	CD1@I322	3.6
	C09	CE1@Y326	3.7
Compound 5	C25	CZ3@W133	7.1
	C26	CD@Q124	5.2
	C25	CB2@C217	5.5
	C25	CG1@V143	5.3
	C25	CG1@I144	3.4
	N18	OD2@D147	2.8

	C19	OH@Y148	4.2
	C06	CH2@W293	3.5
	C10	CE1@H297	3.8
	C03	CD1@I322	4.3
	C17	OH@Y326	5.0

3. Effects of compd. **5** on fentanyl-induced respiratory depression

A.

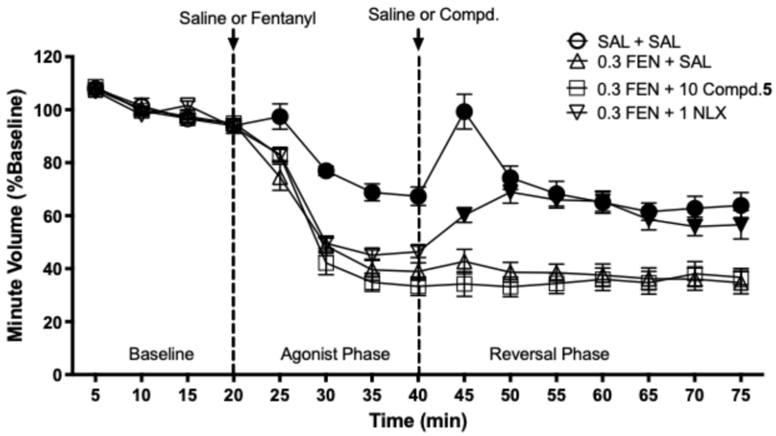

B.

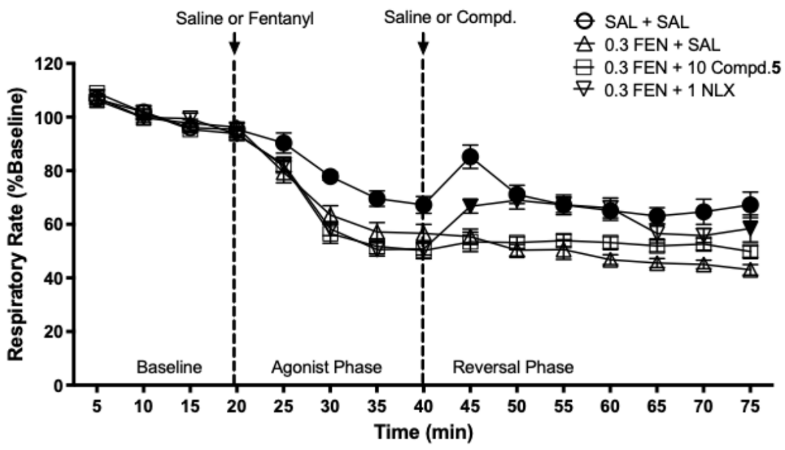

C.

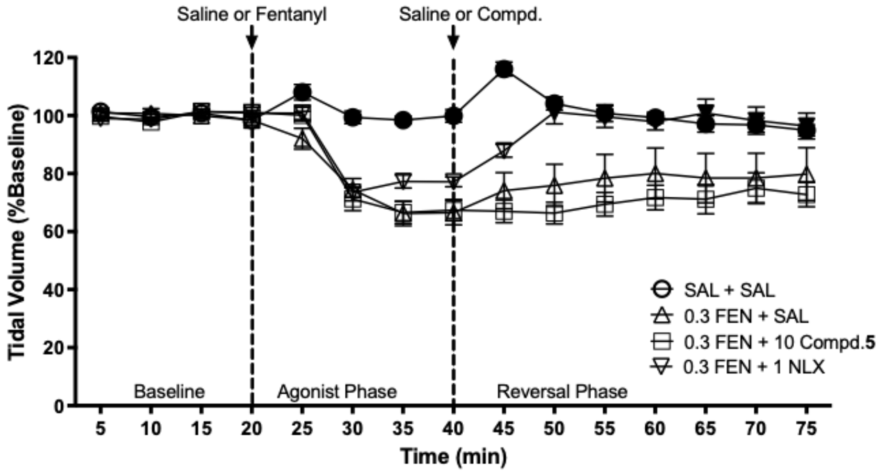

Figure S4. Effects of compd. **5** on fentanyl induced respiratory depression in mice. **(A)** Minute volume; **(B)** Respiratory rate and **(C)** Tidal volume. Error bars represent the standard error of normalized mean values within individual 5 min bins. Closed symbols indicate significant differences compared to the fentanyl (0.3 FEN + SAL)-treated controls at individual timepoints ($p \leq 0.05$) via one-way ANOVA.

4. NMR and HRMS spectrum for intermediates **b**, **c1-c6**, **f**, **g1-g6**, and compounds **1-78**.

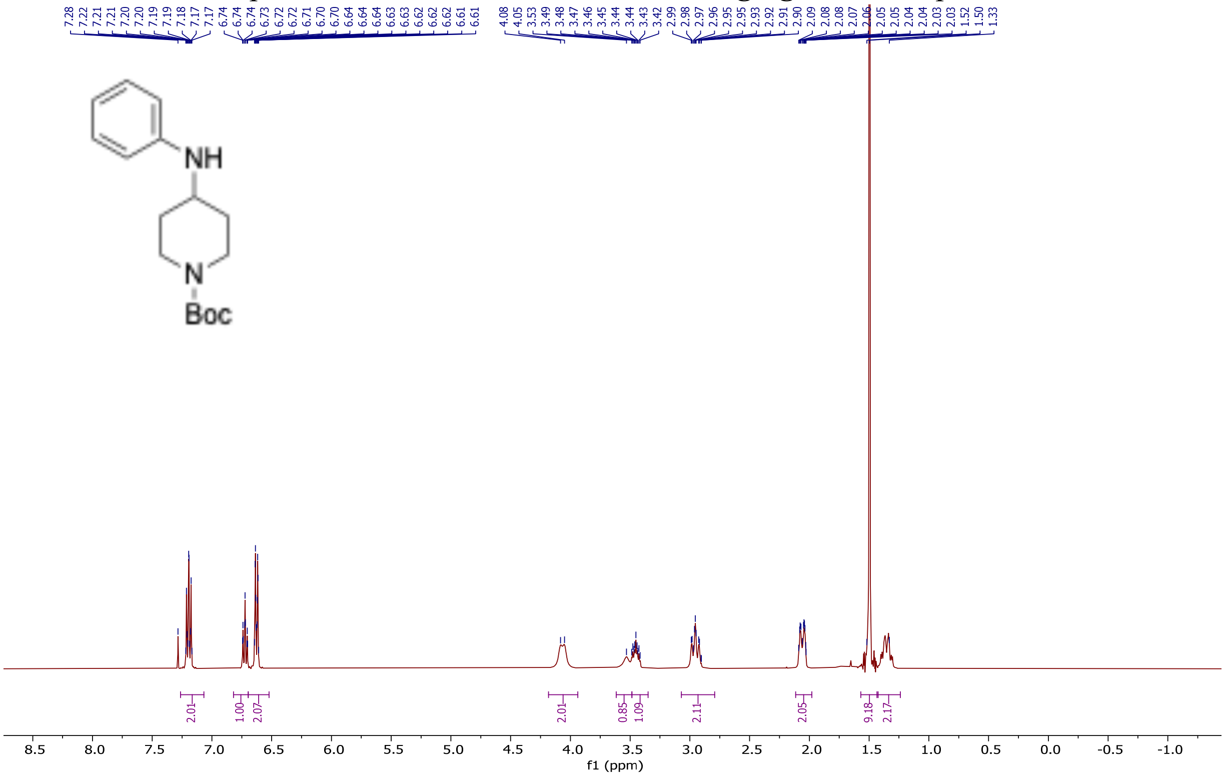

^{1}H NMR (400 MHz, $CDCl_3$) spectrum for compound **b**.

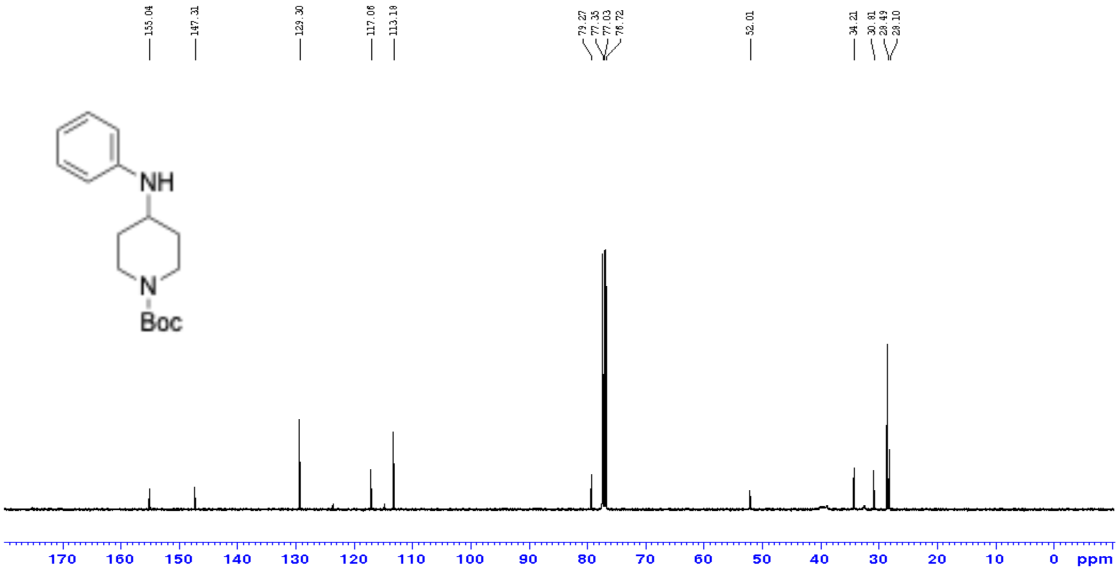

^{13}C NMR (100 MHz, $CDCl_3$) spectrum for compound **b**.

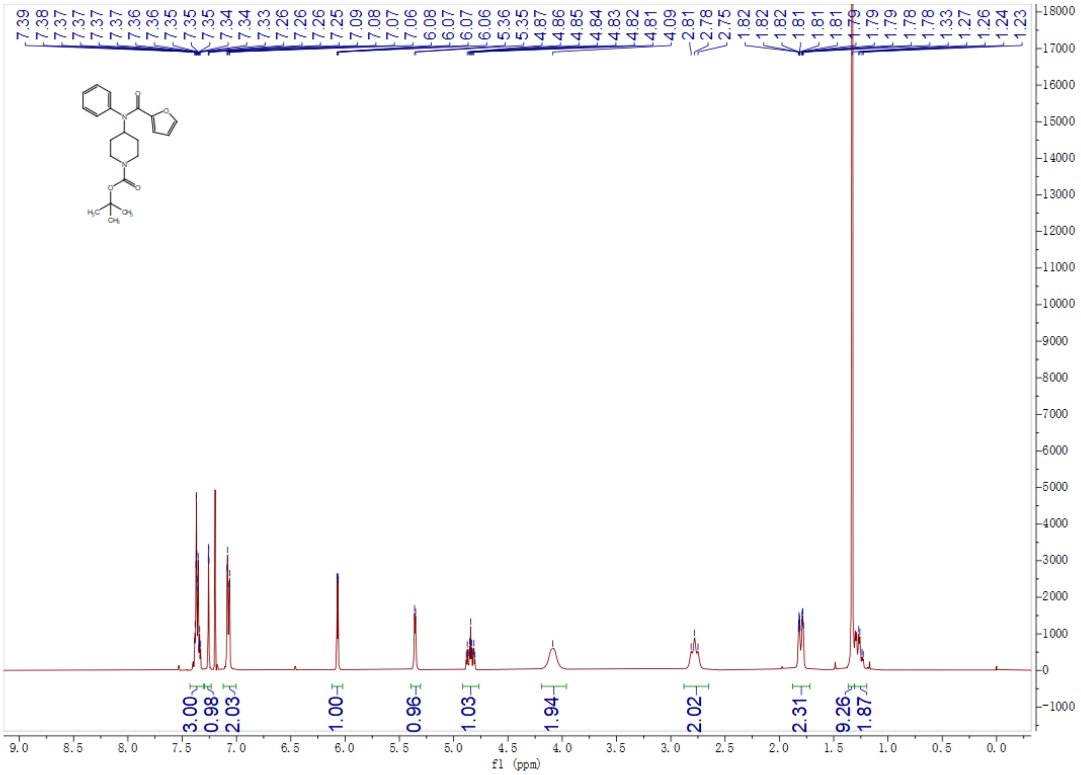

^{1}H NMR (400 MHz, CDCl3) spectrum for compound **c1**.

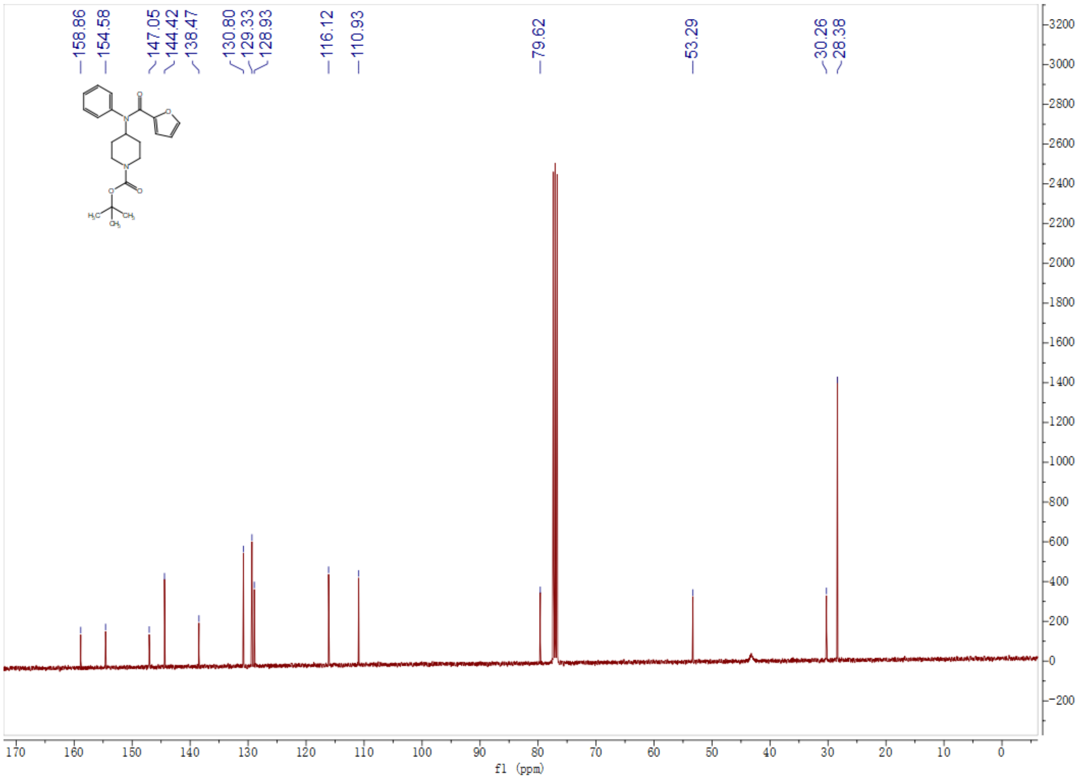

^{13}C NMR (100 MHz, CDCl3) spectrum for compound **c1**.

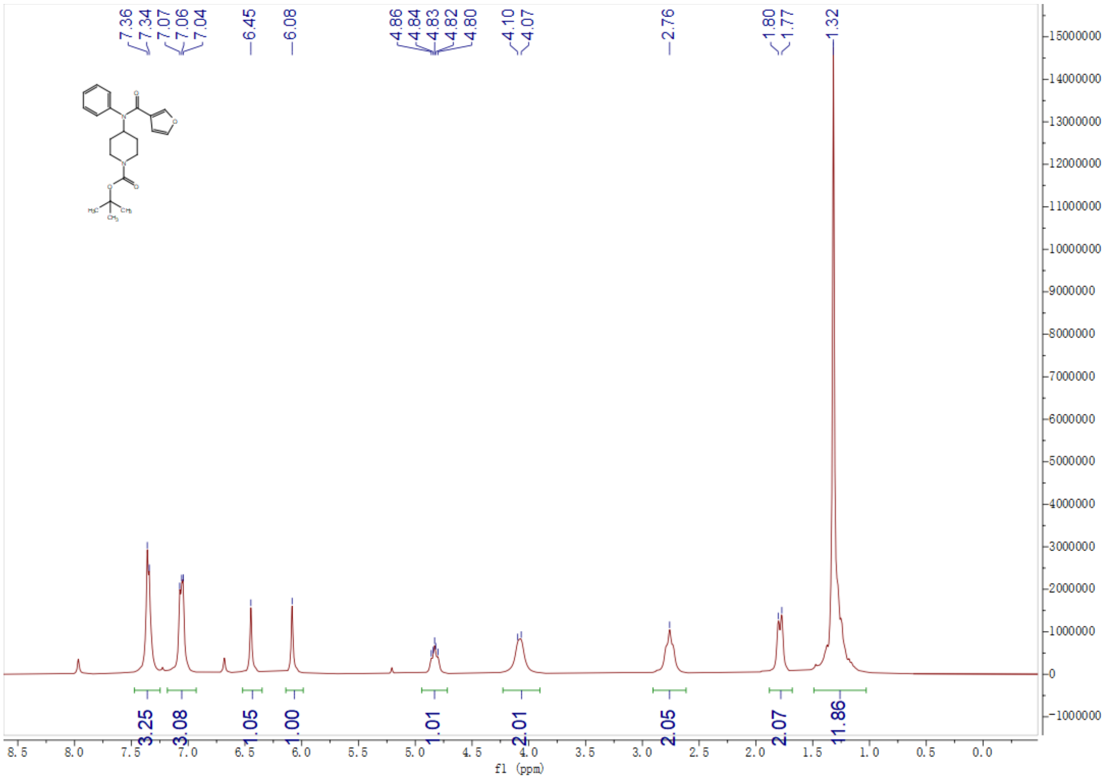

^{1}H NMR (400 MHz, CDCl3) spectrum for compound **c2**.

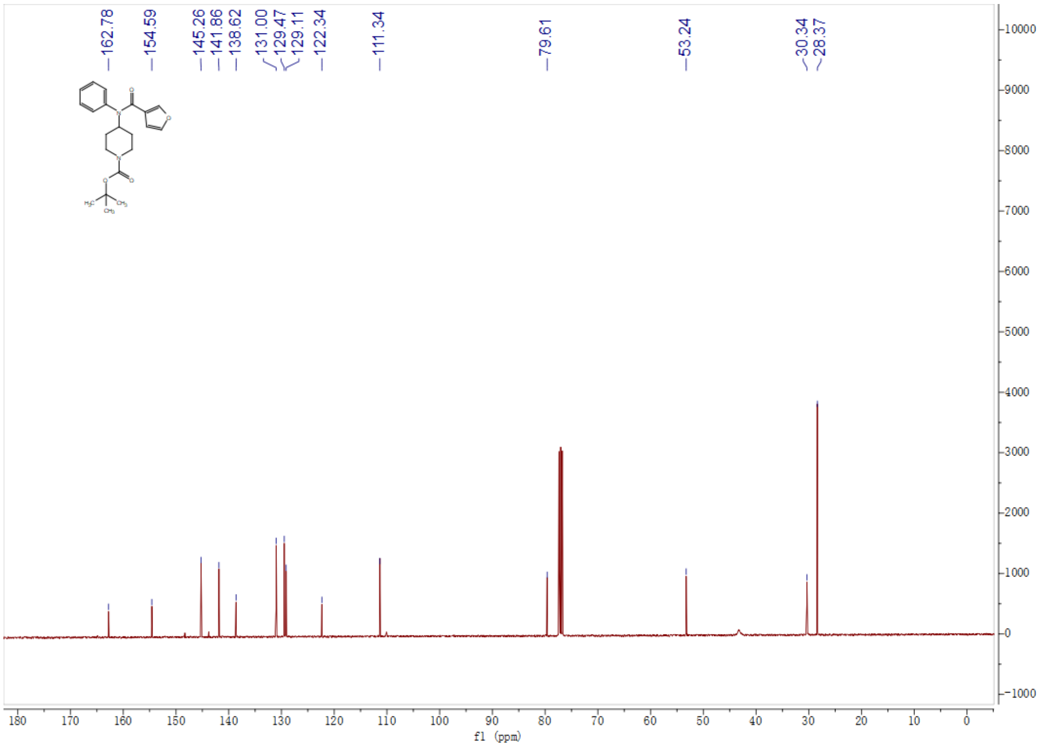

^{13}C NMR (100 MHz, CDCl3) spectrum for compound **c2**.

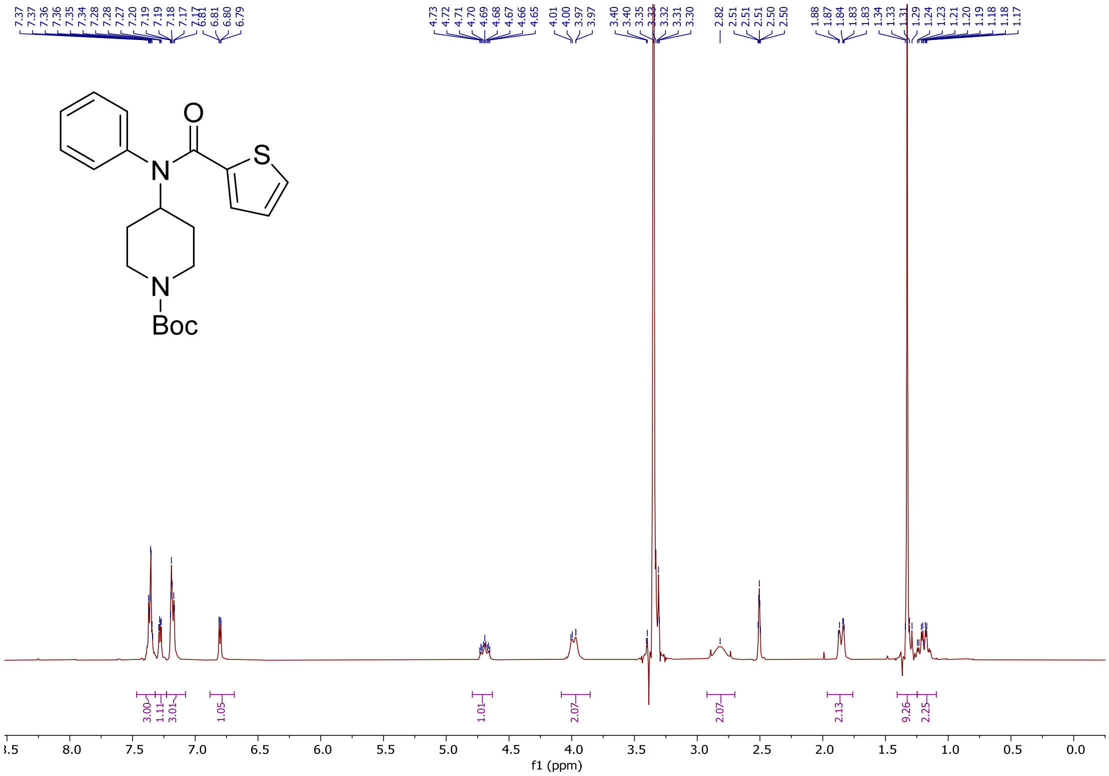

^{1}H NMR (400 MHz, $CDCl_3$) spectrum for compound **c3.**

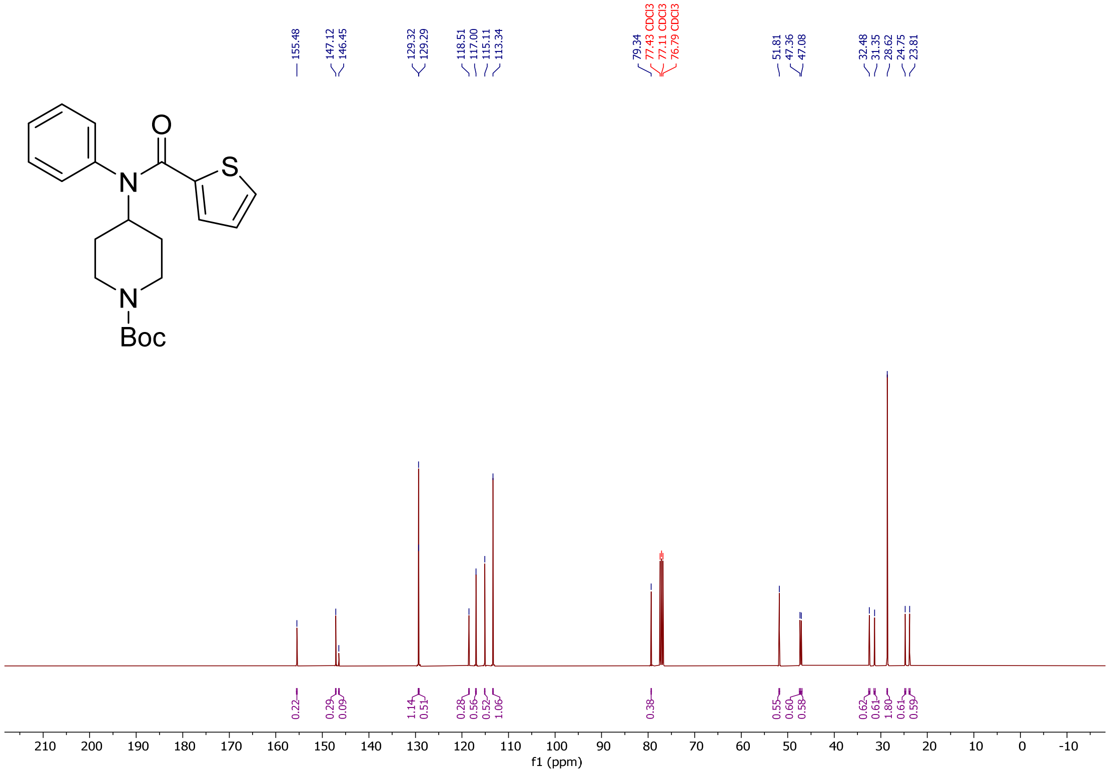

^{13}C NMR (100 MHz, $CDCl_3$) spectrum for compound **c3**.

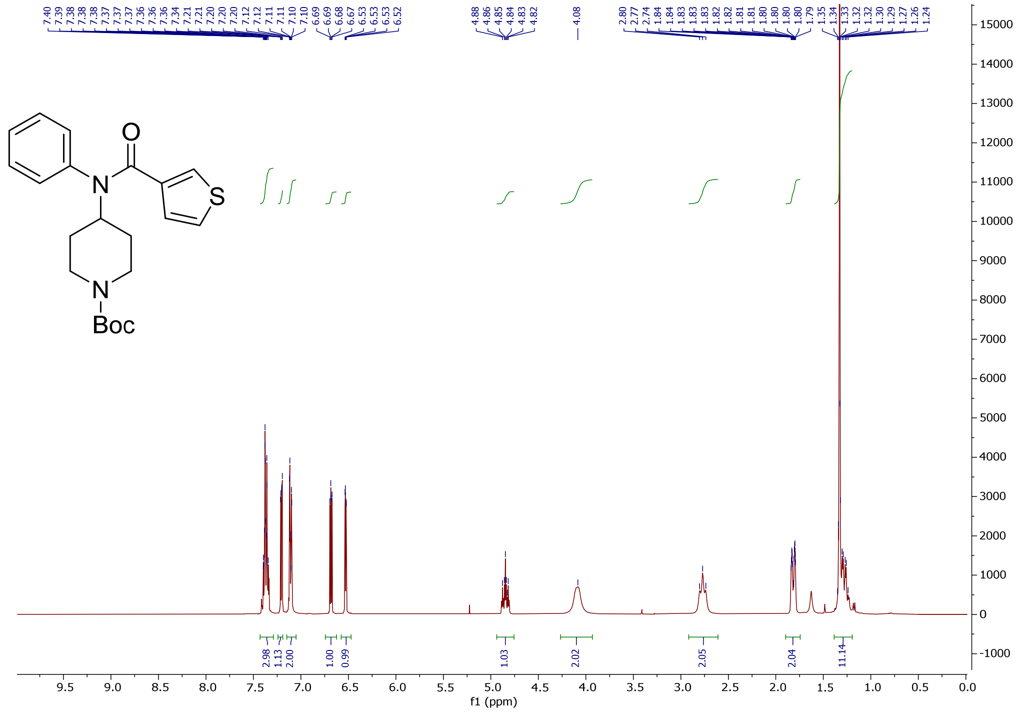

^{1}H NMR (400 MHz, $CDCl_3$) spectrum for compound **c4.**

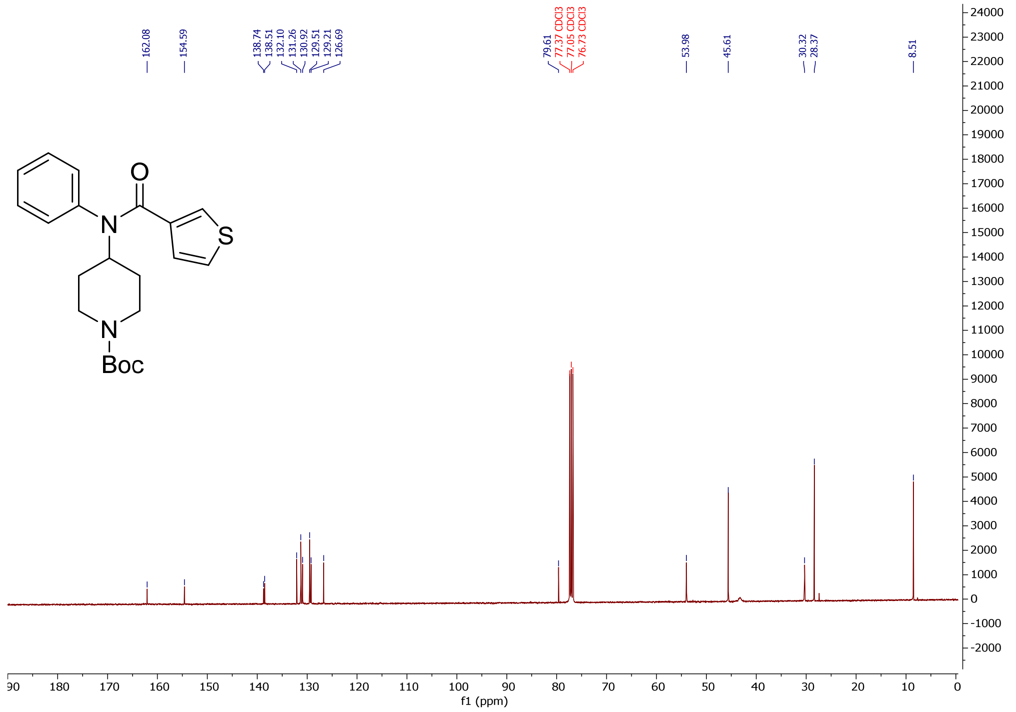

^{13}C NMR (100 MHz, $CDCl_3$) spectrum for compound **c4**.

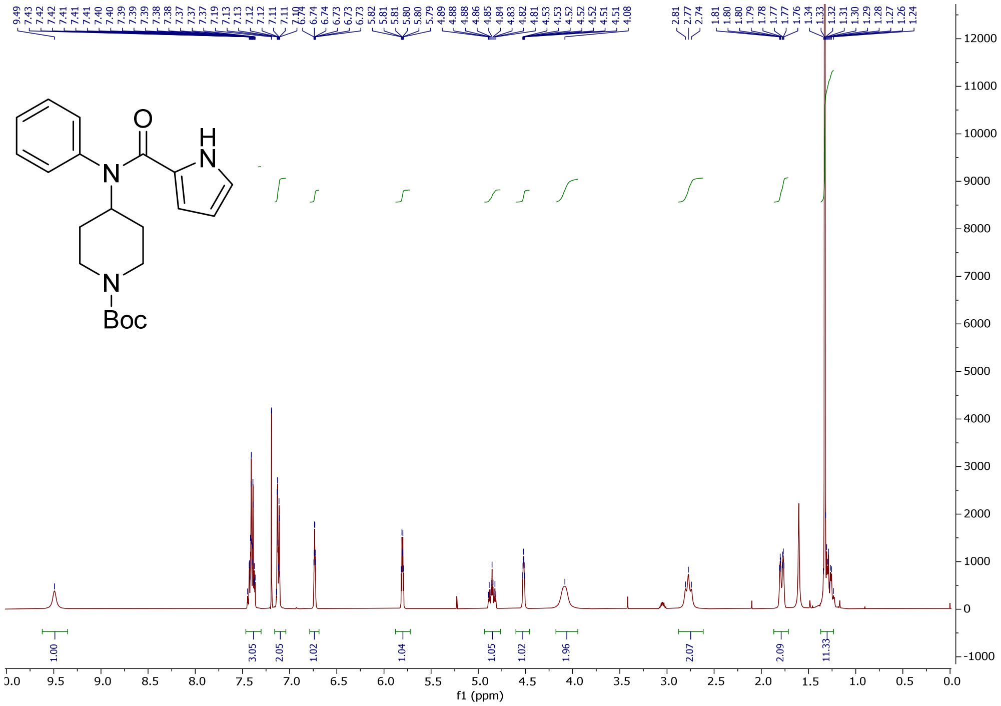

^{1}H NMR (400 MHz, $CDCl_3$) spectrum for compound **c5.**

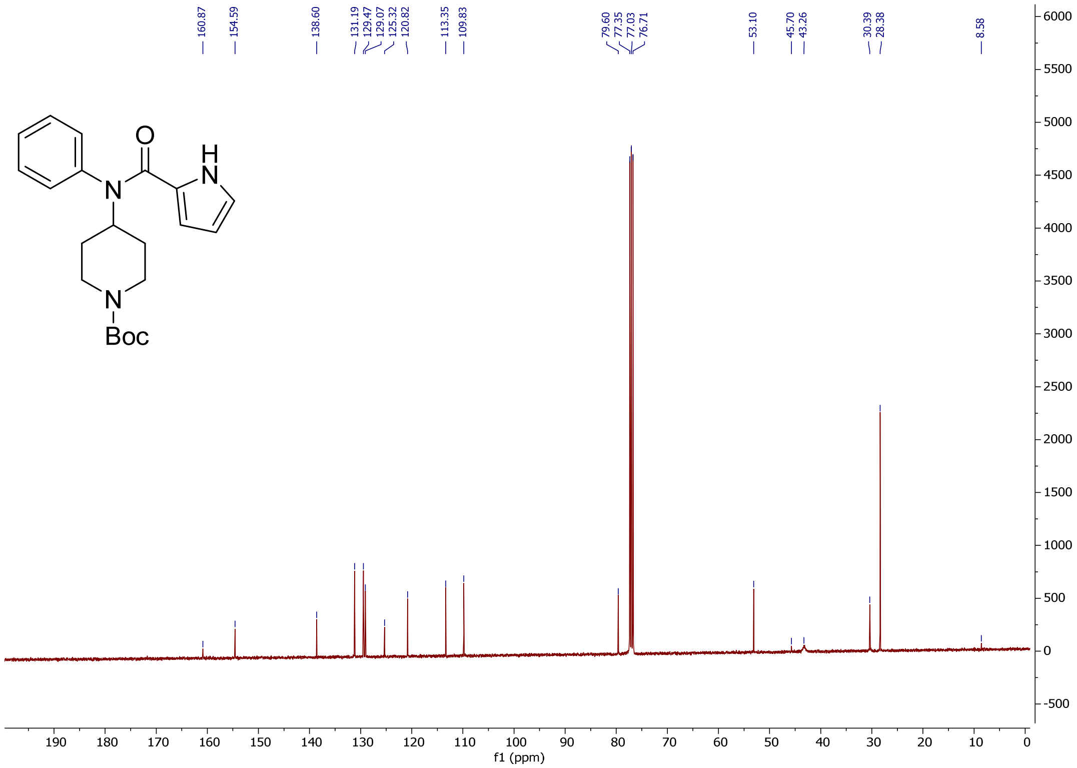

^{13}C NMR (100 MHz, $CDCl_3$) spectrum for compound **c5**.

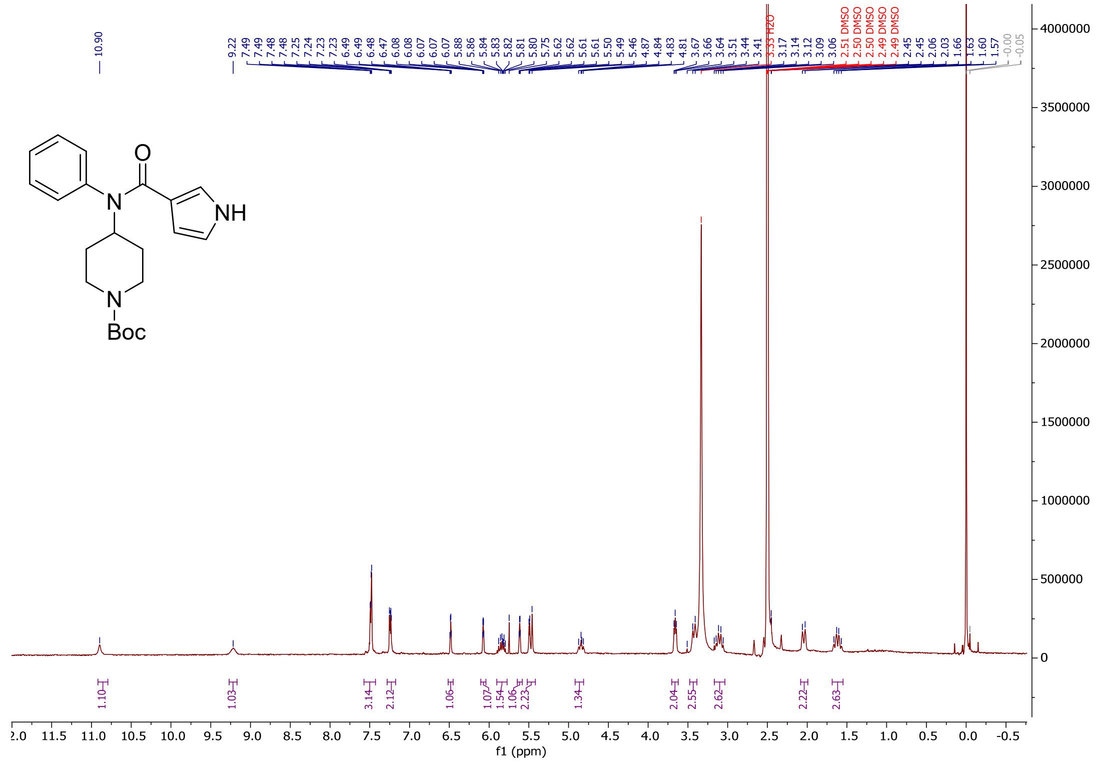

^{1}H NMR (400 MHz, DMSO-d_6) spectrum for compound **c6**

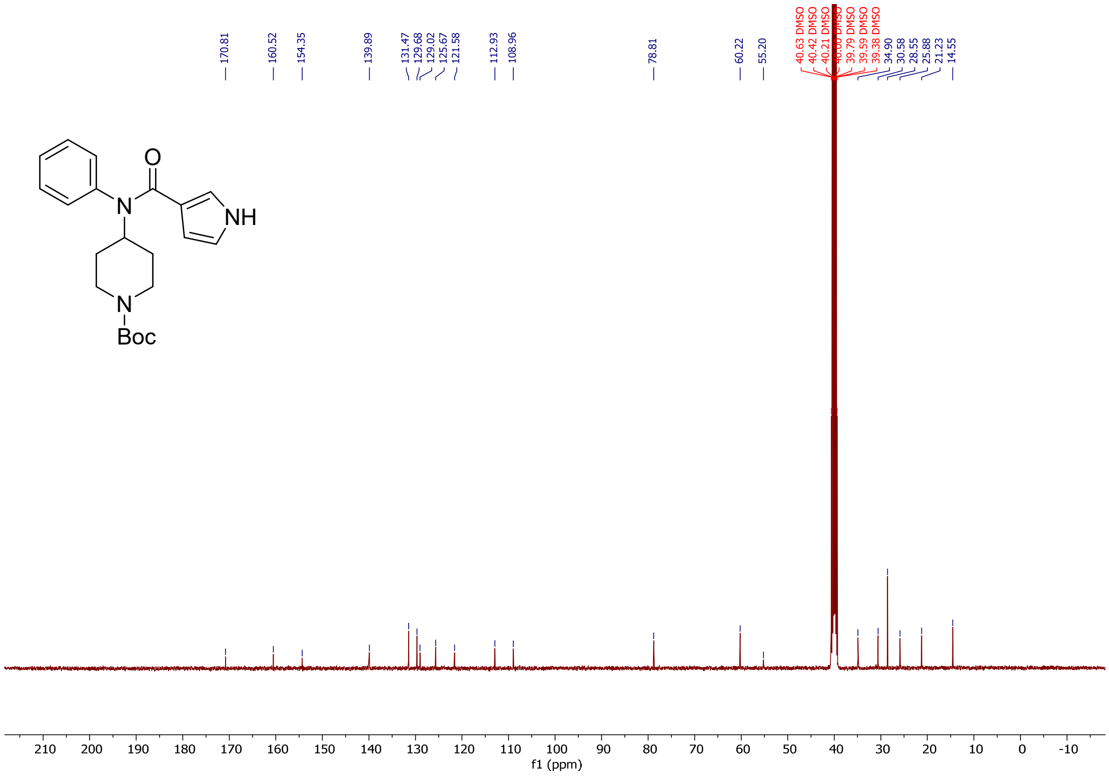

^{13}C NMR (100 MHz, DMSO-d_6) spectrum for compound **c6**.

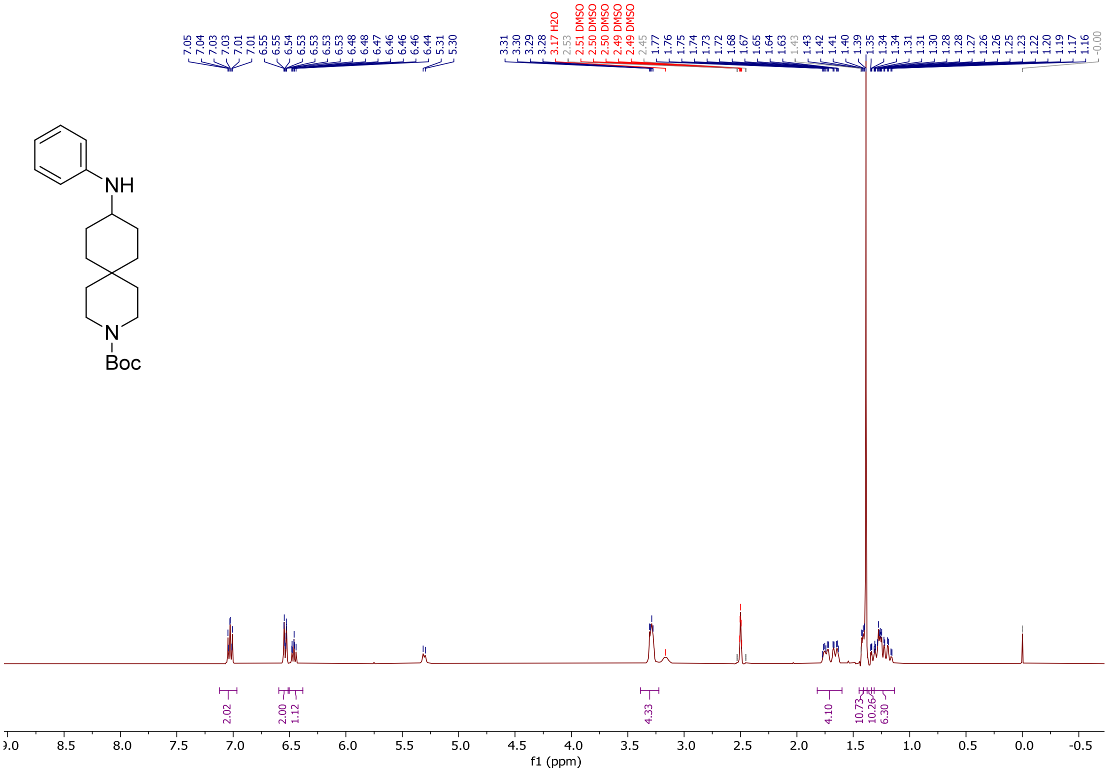

^{1}H NMR (400 MHz, DMSO-d_6) spectrum for compound **f.**

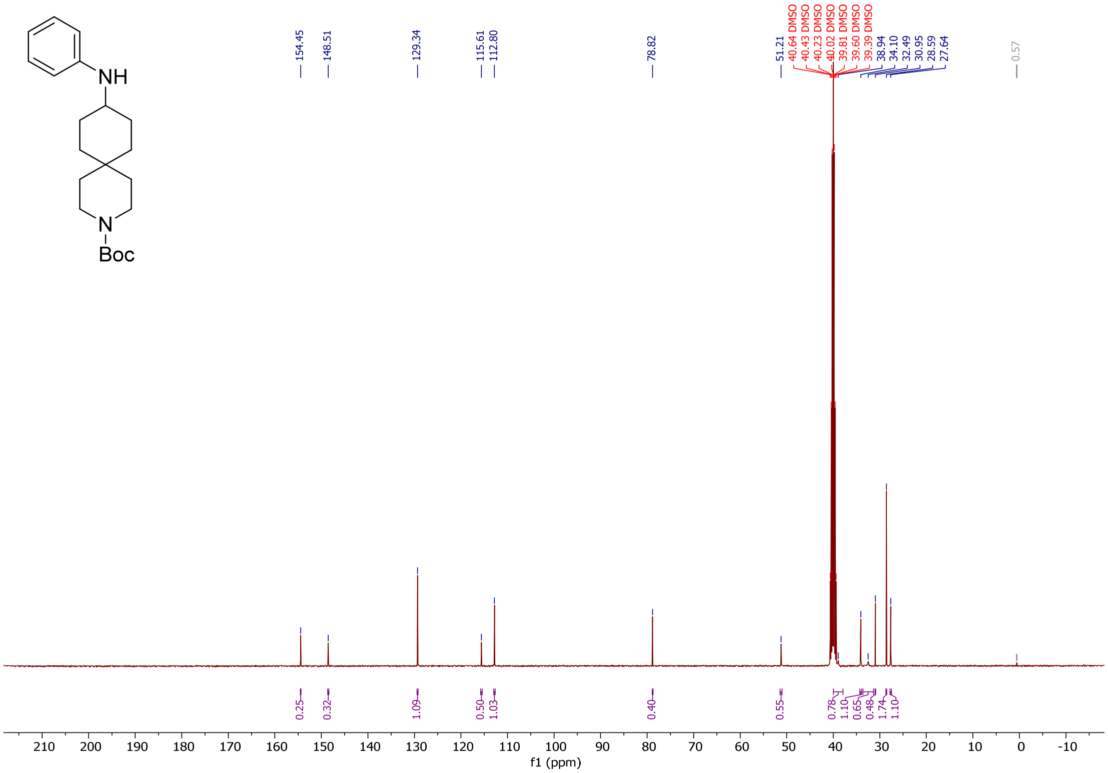

^{13}C NMR (100 MHz, DMSO-d_6) spectrum for compound **f**.

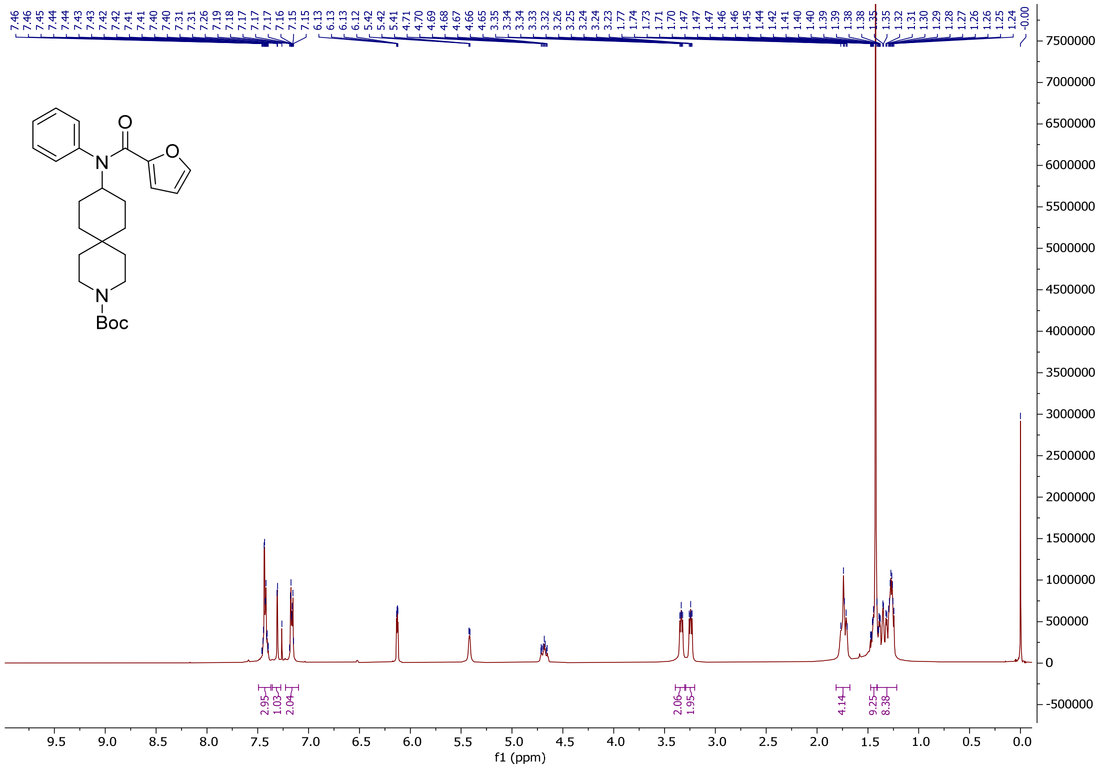

^{1}H NMR (400 MHz, $CDCl_3$) spectrum for compound **g1**.

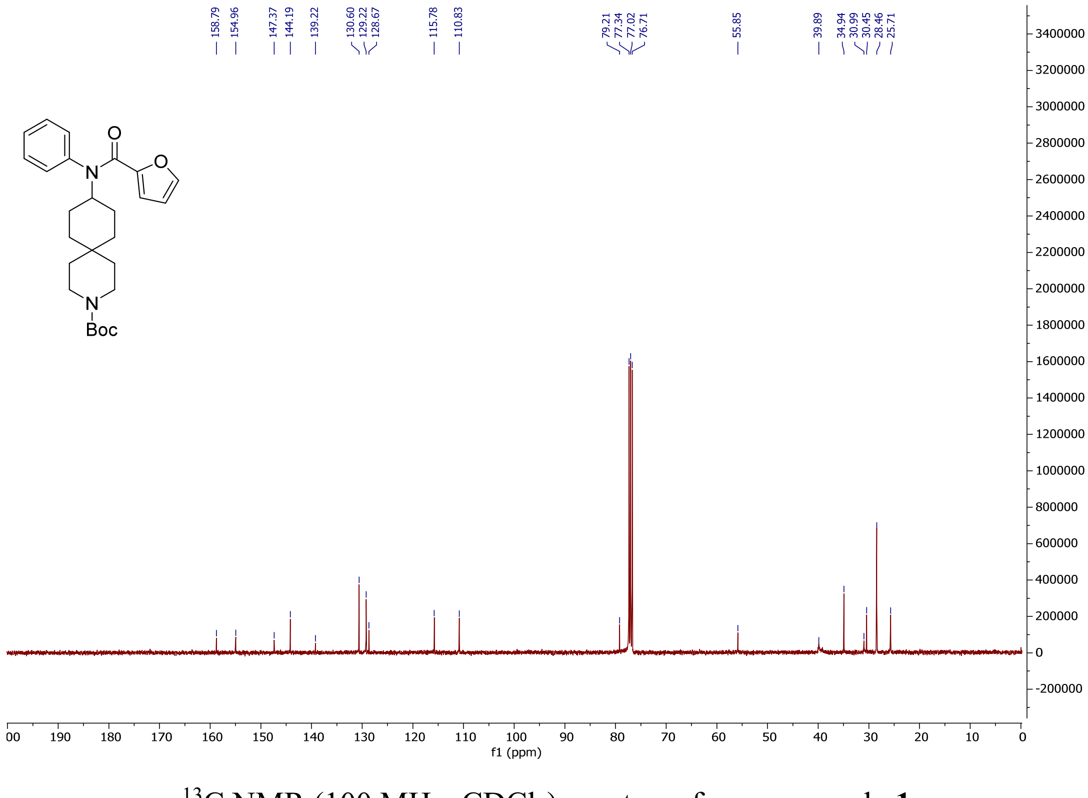

^{13}C NMR (100 MHz, $CDCl_3$) spectrum for compound **g1**.

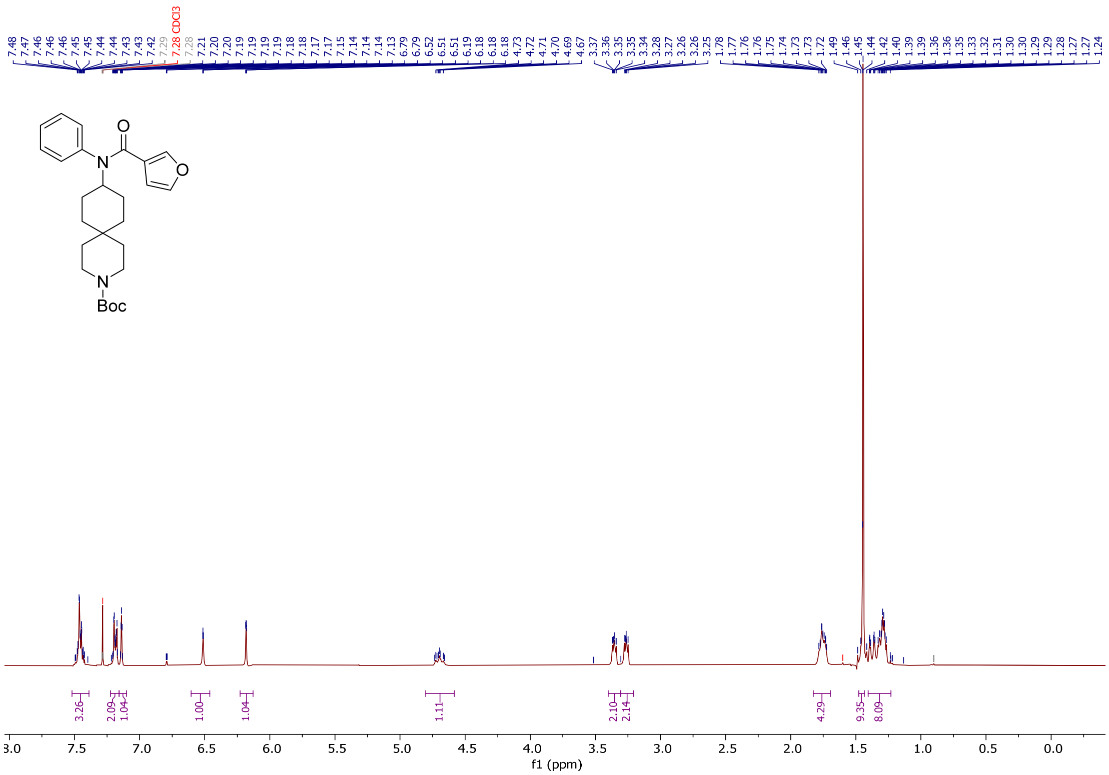

^{1}H NMR (400 MHz, $CDCl_3$) spectrum for compound **g2**.

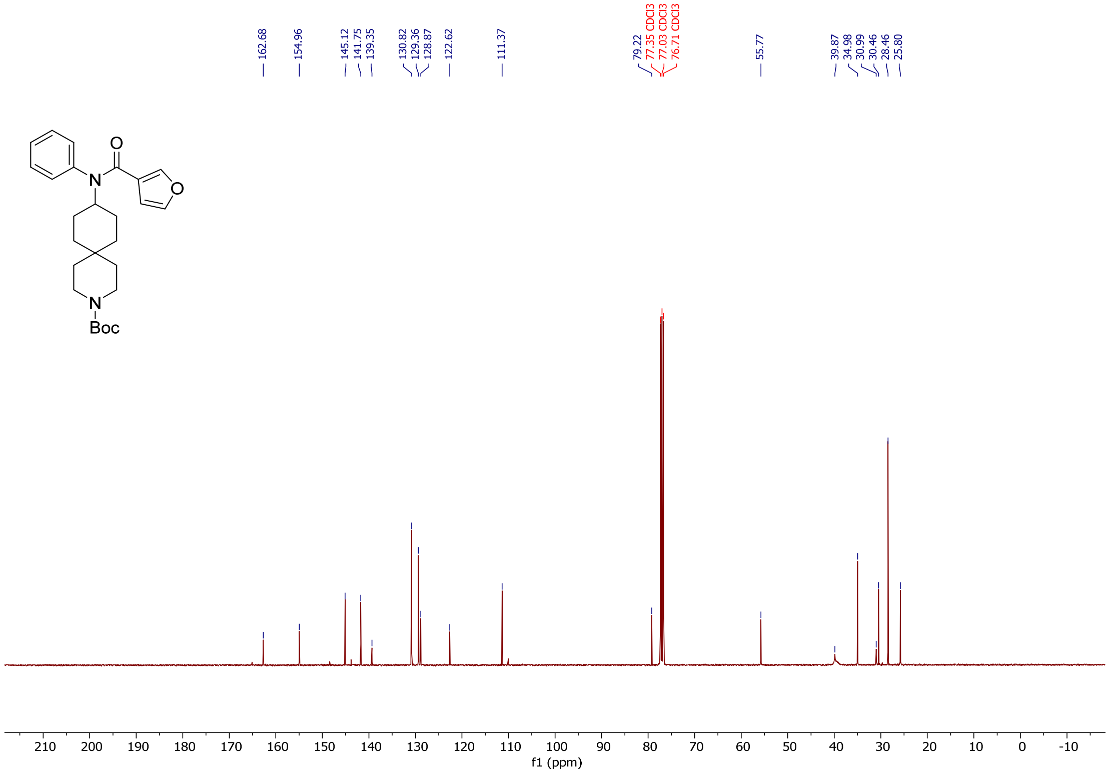

^{13}C NMR (100 MHz, $CDCl_3$) spectrum for compound **g2**.

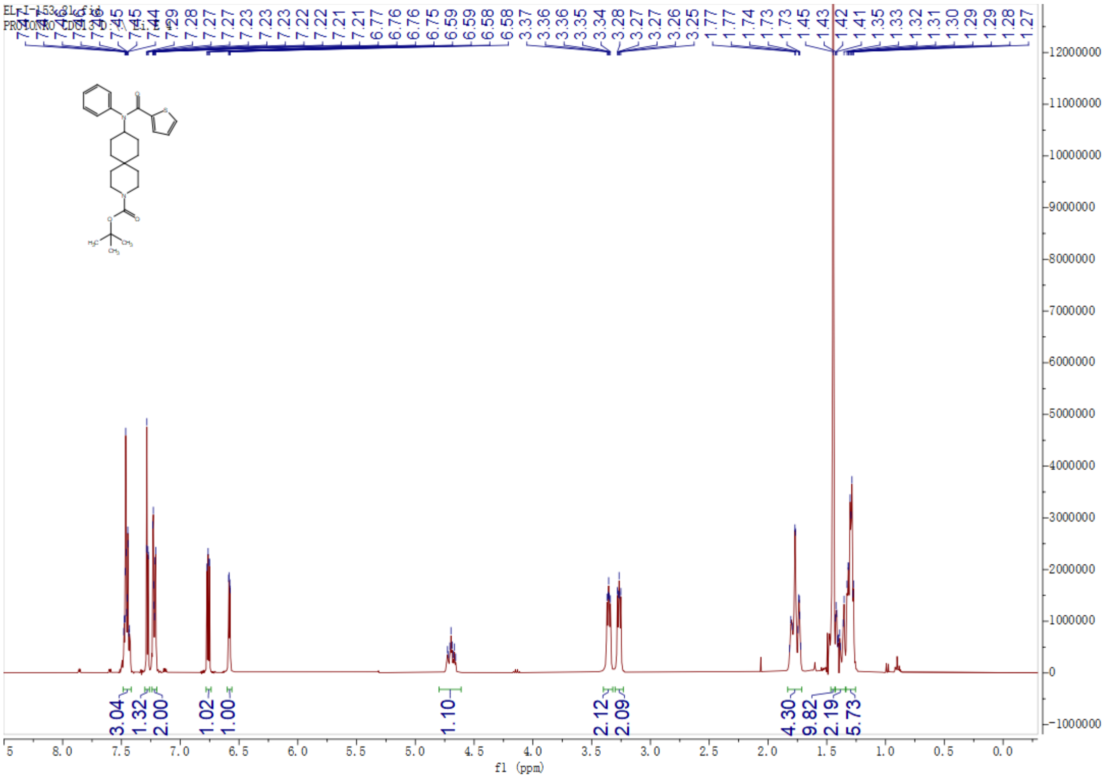

^{1}H NMR (400 MHz, $CDCl_3$) spectrum for compound **g3**.

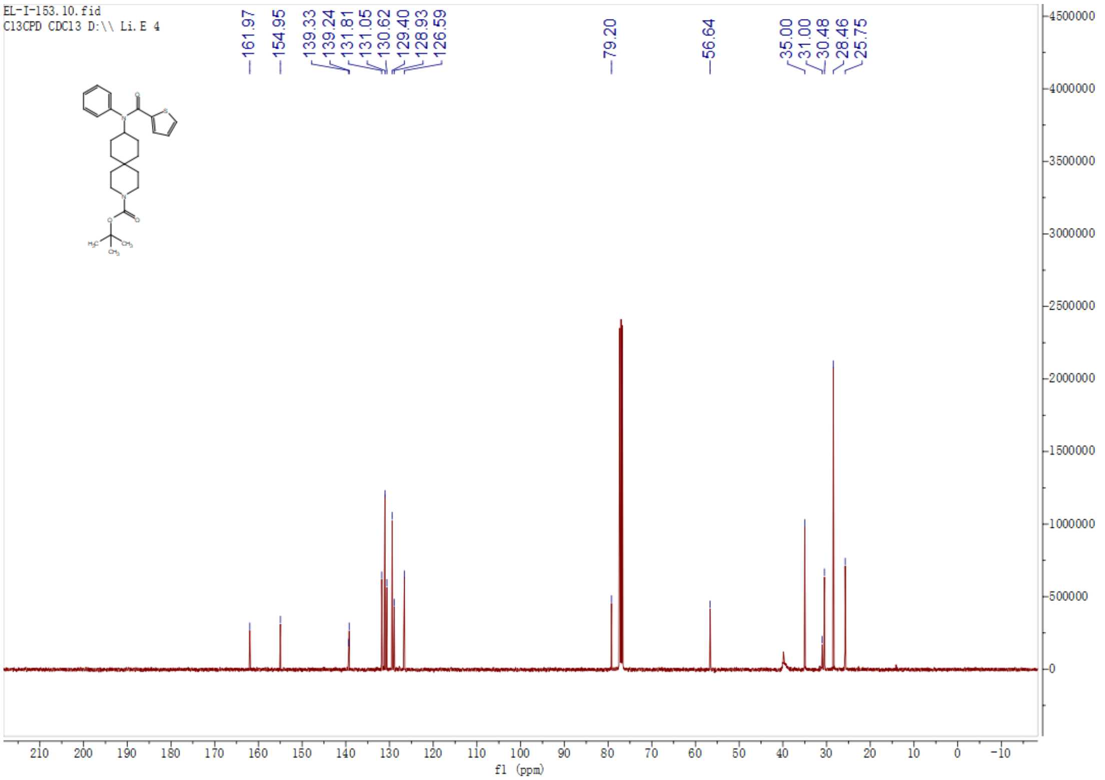

^{13}C NMR (100 MHz, $CDCl_3$) spectrum for compound **g3**.

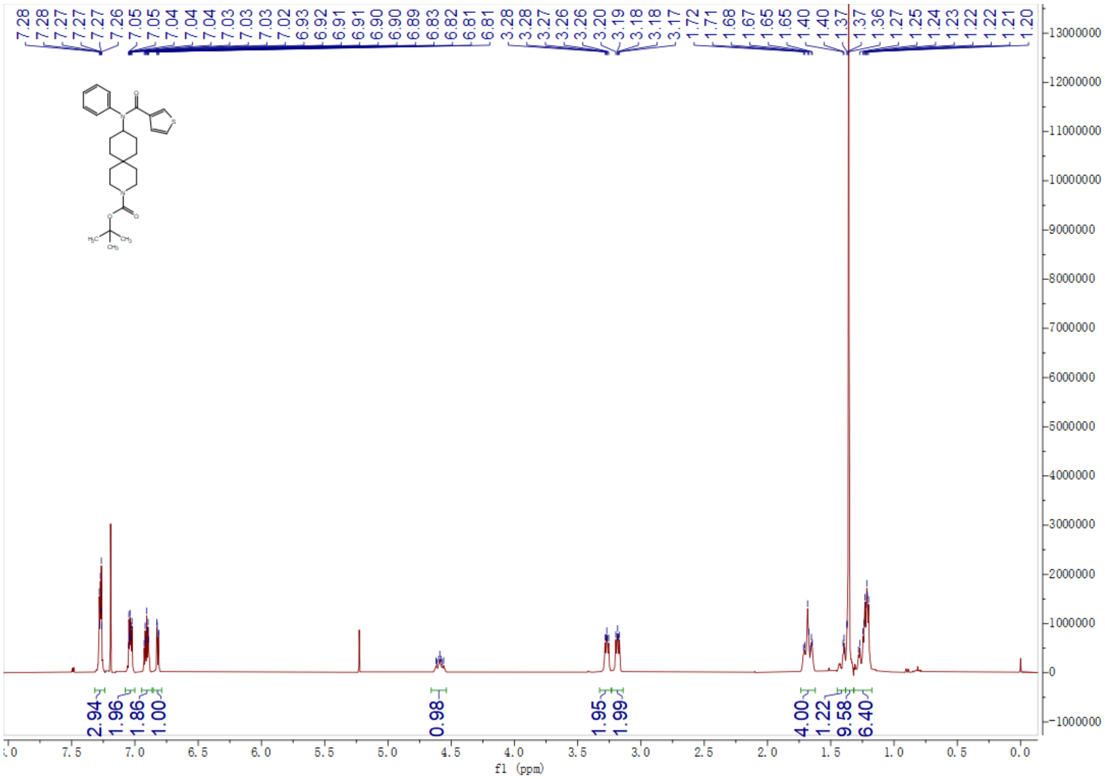

^{1}H NMR (400 MHz, $CDCl_3$) spectrum for compound **g4**.

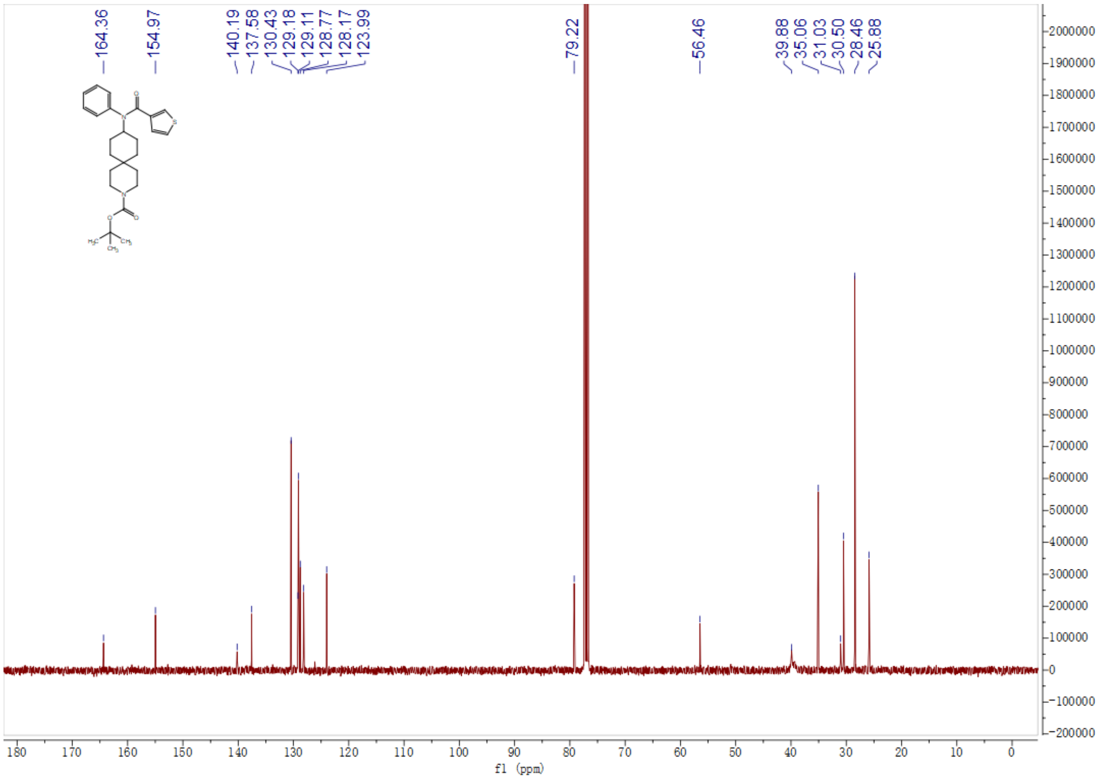

^{13}C NMR (100 MHz, $CDCl_3$) spectrum for compound **g4**.

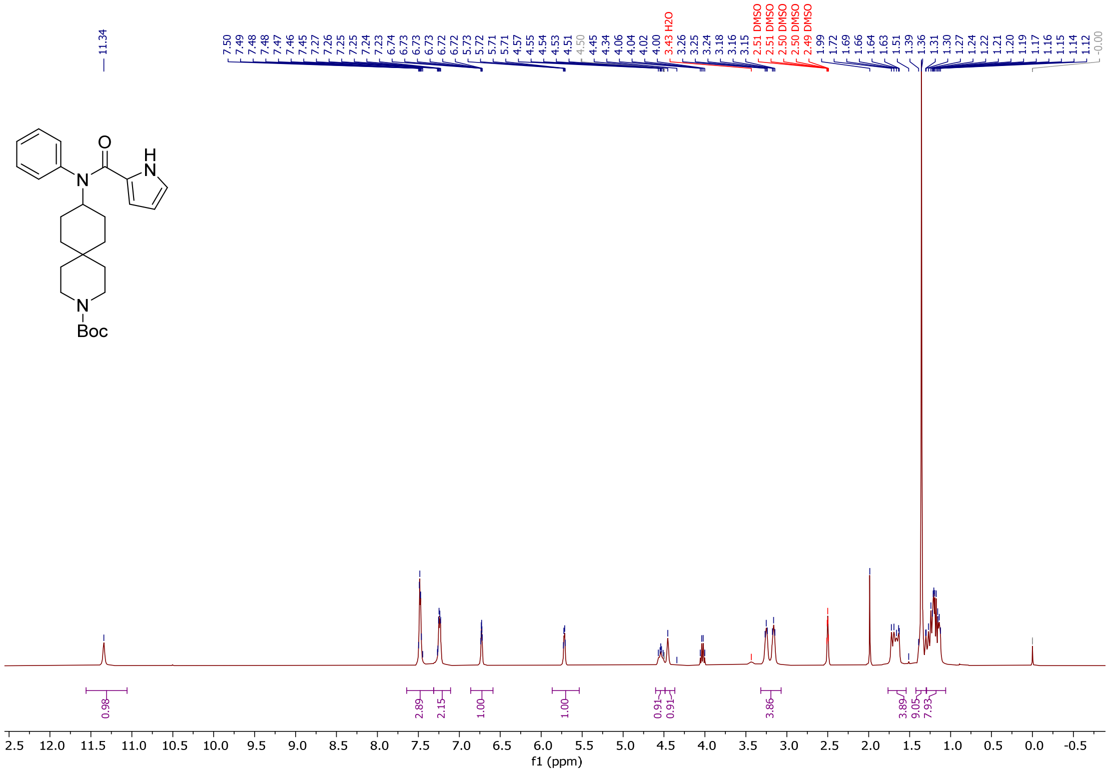

^{1}H NMR (400 MHz, DMSO-d_6) spectrum for compound **g5.**

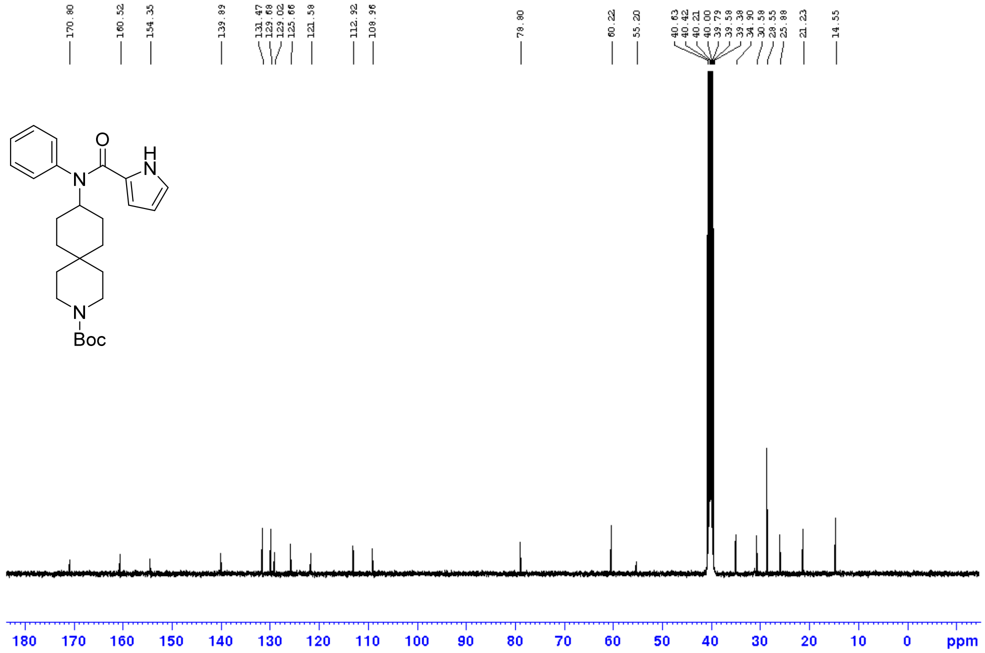

^{13}C NMR (100 MHz, DMSO-d_6) spectrum for compound **g5**.

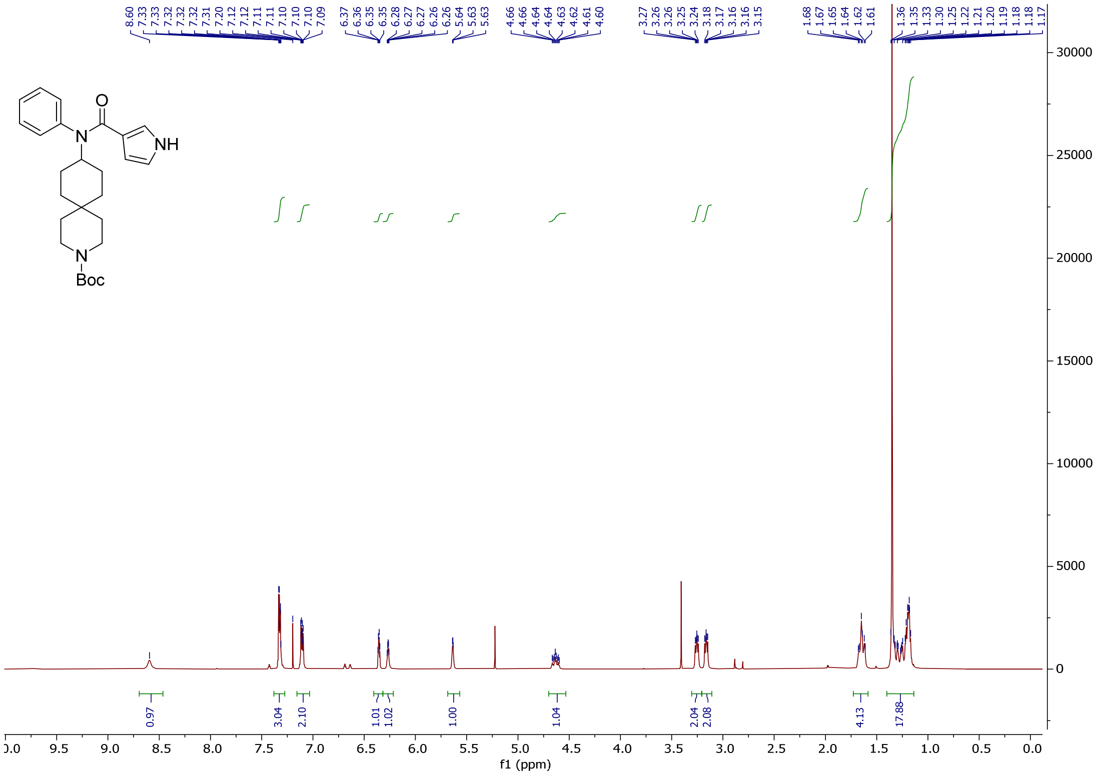

^{1}H NMR (400 MHz, $CDCl_3$) spectrum for compound **g6**.

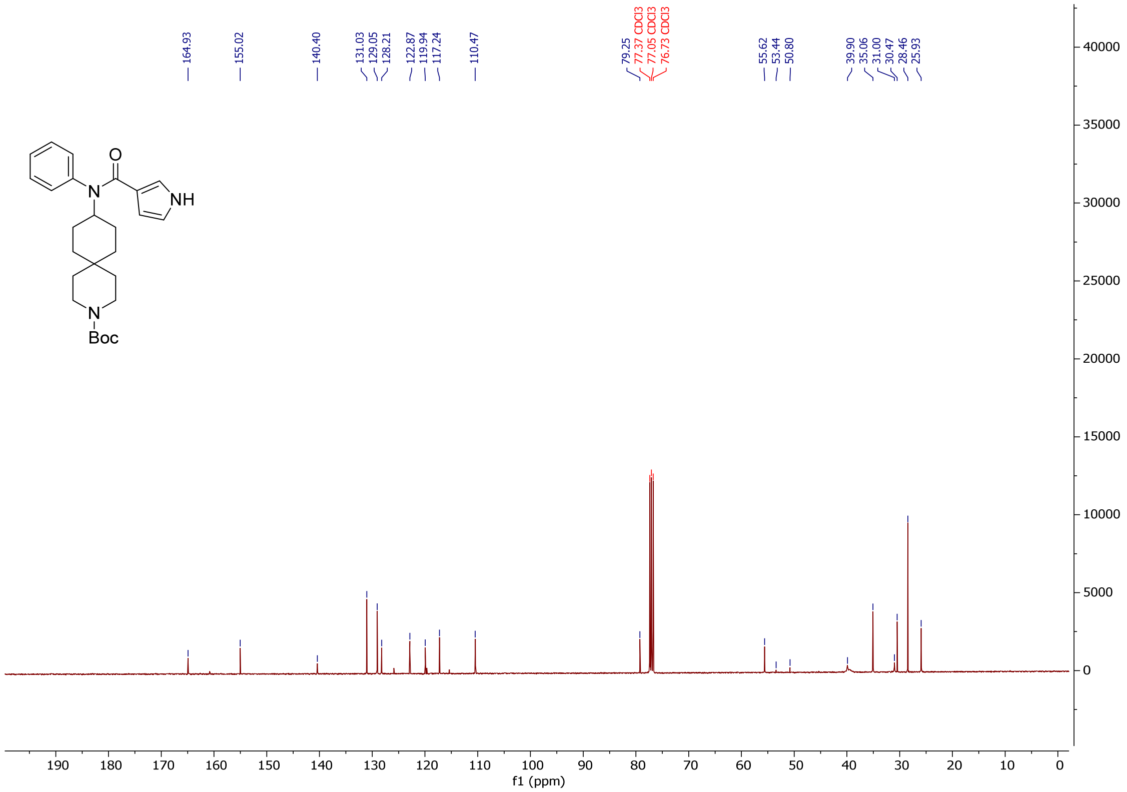

^{13}C NMR (100 Hz, $CDCl_3$) spectrum for compound **g6**.

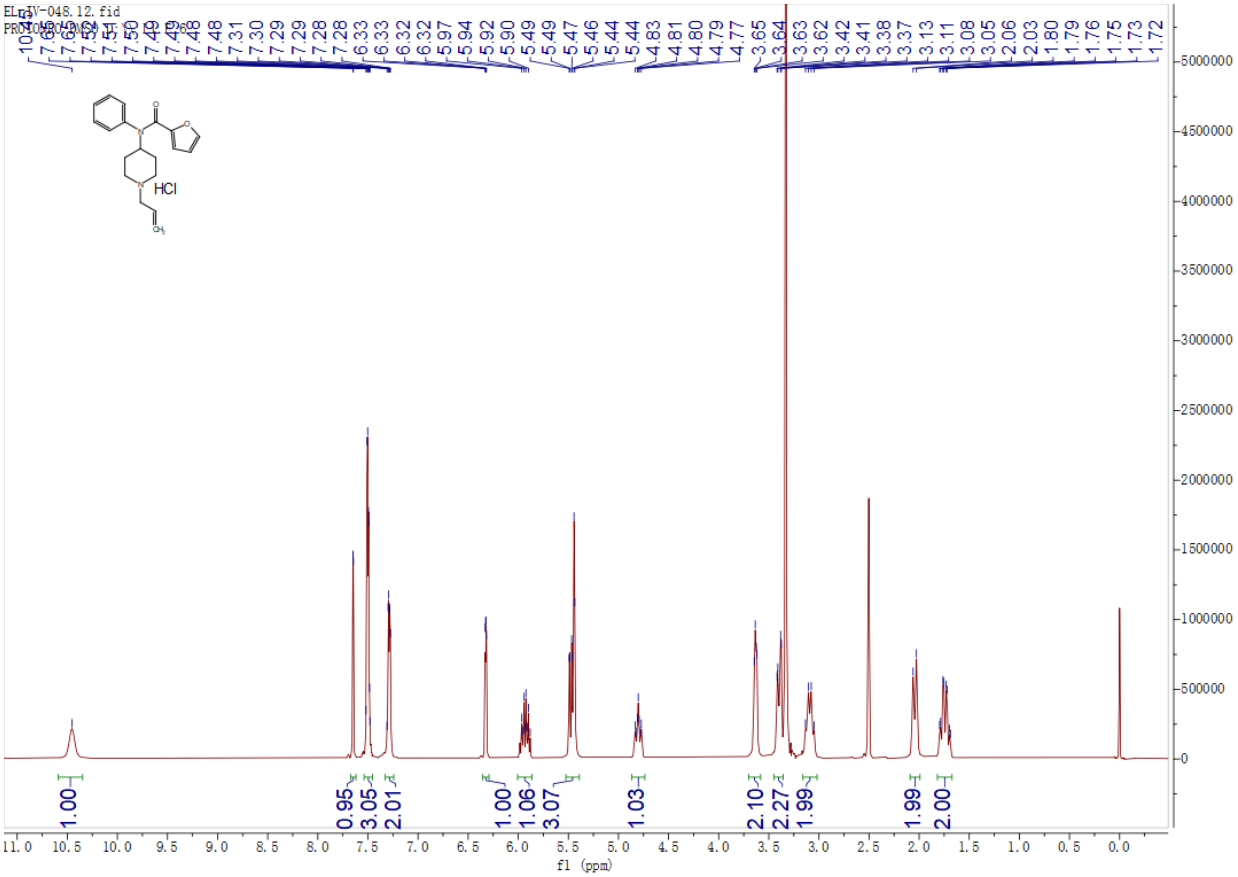

^{1}H NMR (400 MHz, DMSO-d_6) spectrum for compound **1**.

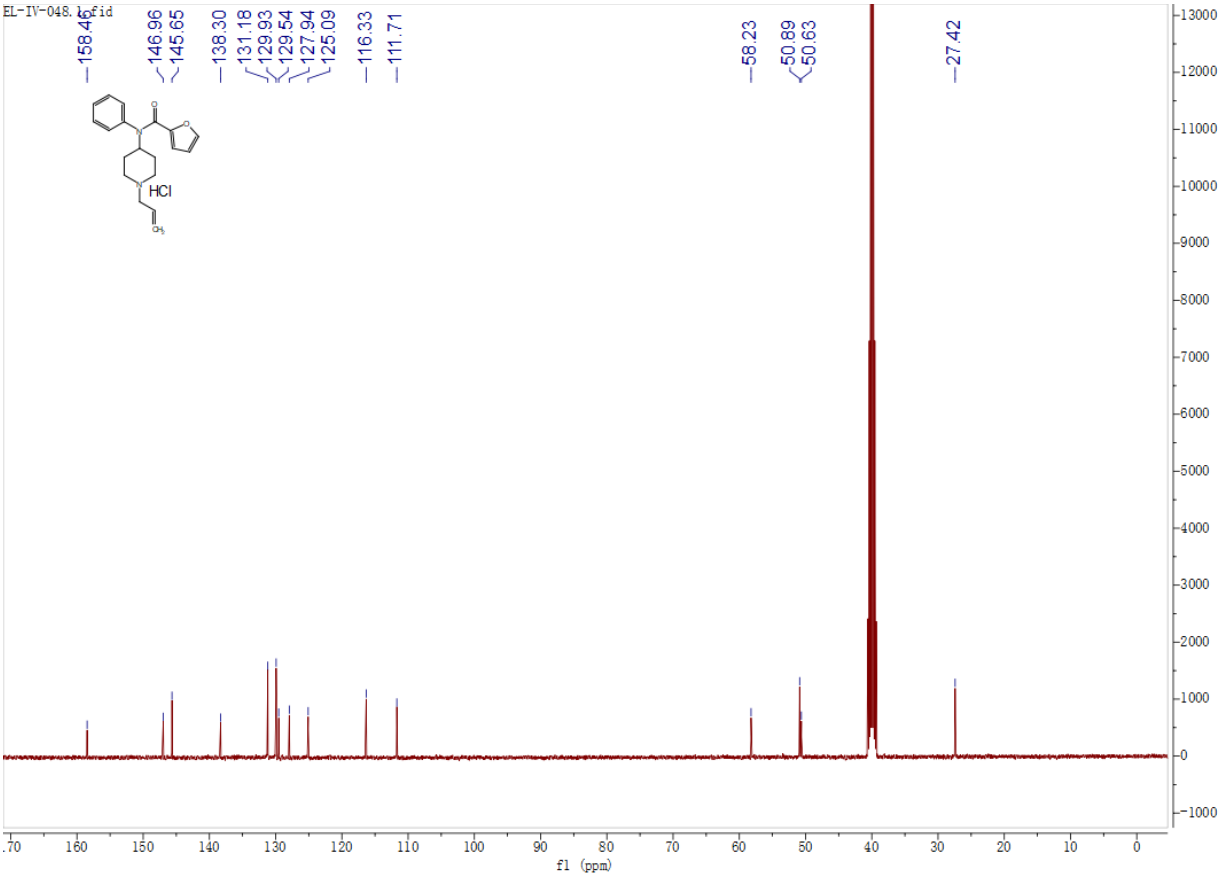

^{13}C NMR (100 MHz, DMSO-d_6) spectrum for compound **1**.

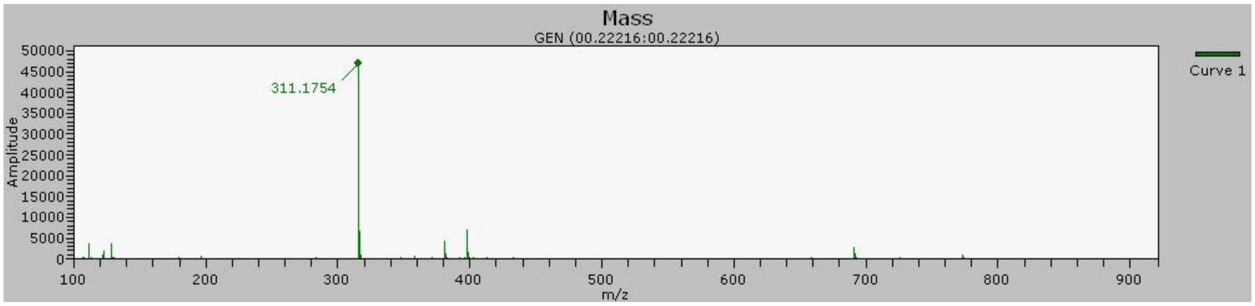

HRMS spectrum for compound **1**.

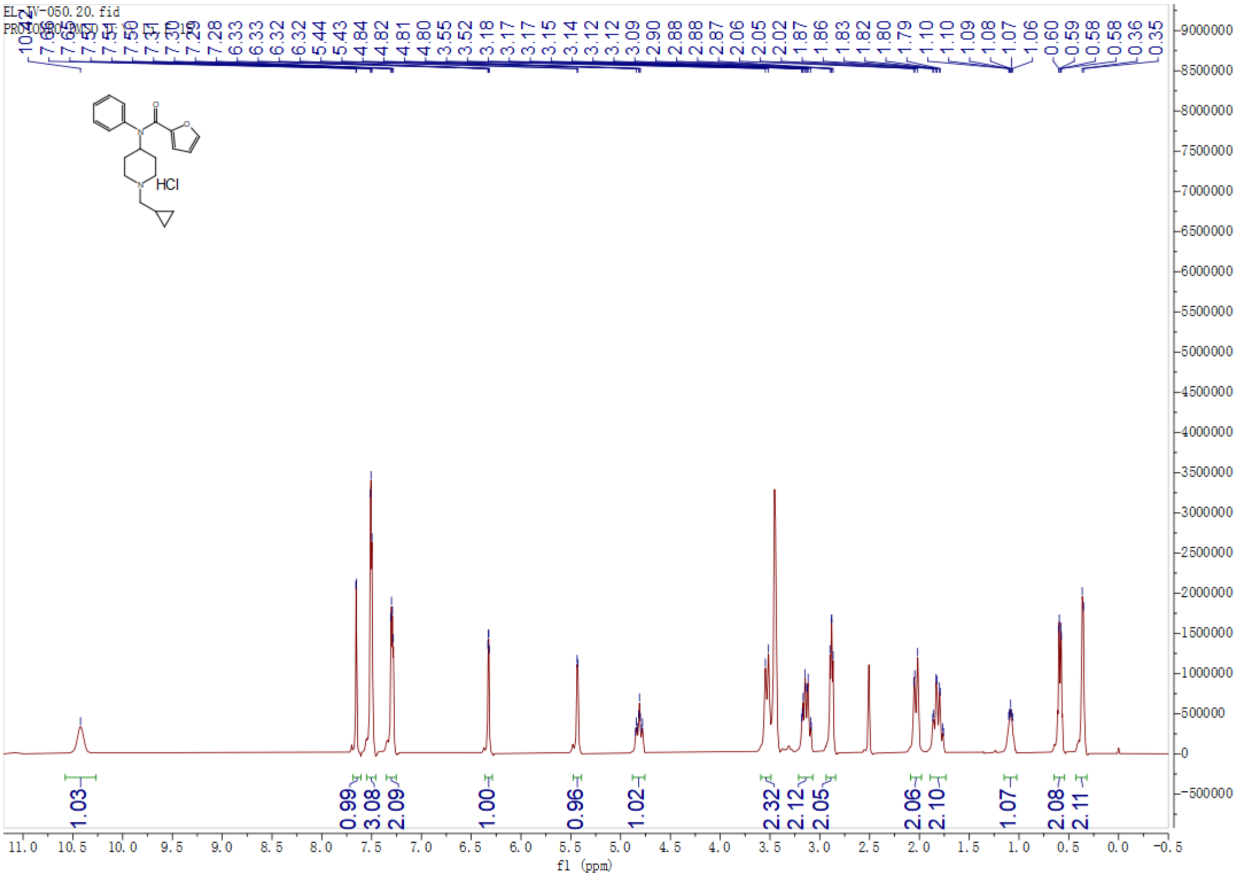

^{1}H NMR (400 MHz, DMSO-d_6) spectrum for compound **2**.

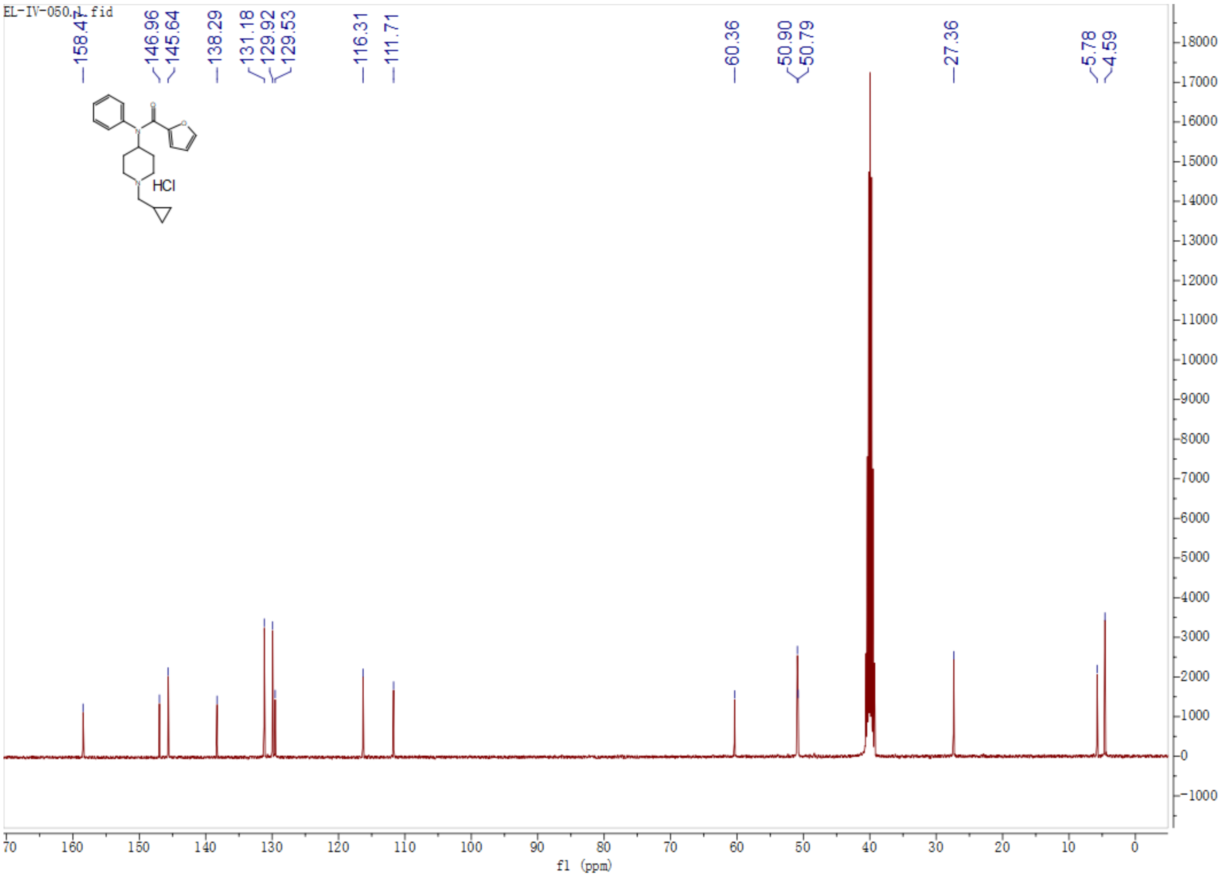

^{13}C NMR (100 MHz, DMSO-d_6) spectrum for compound **2**.

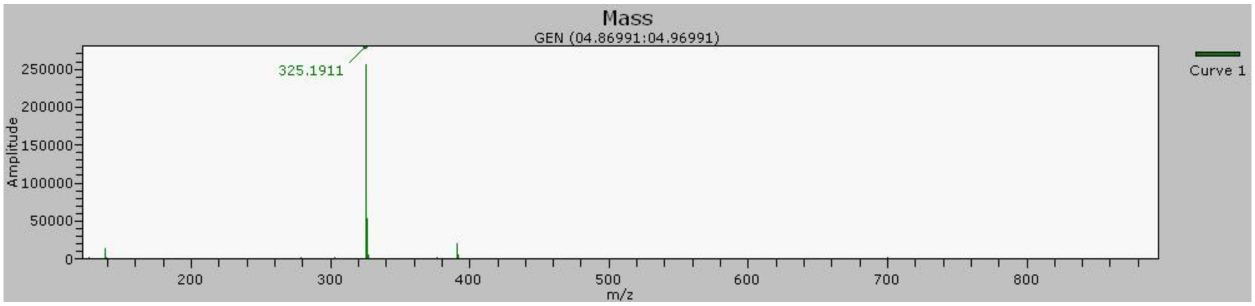

HRMS spectrum for compound **2**.

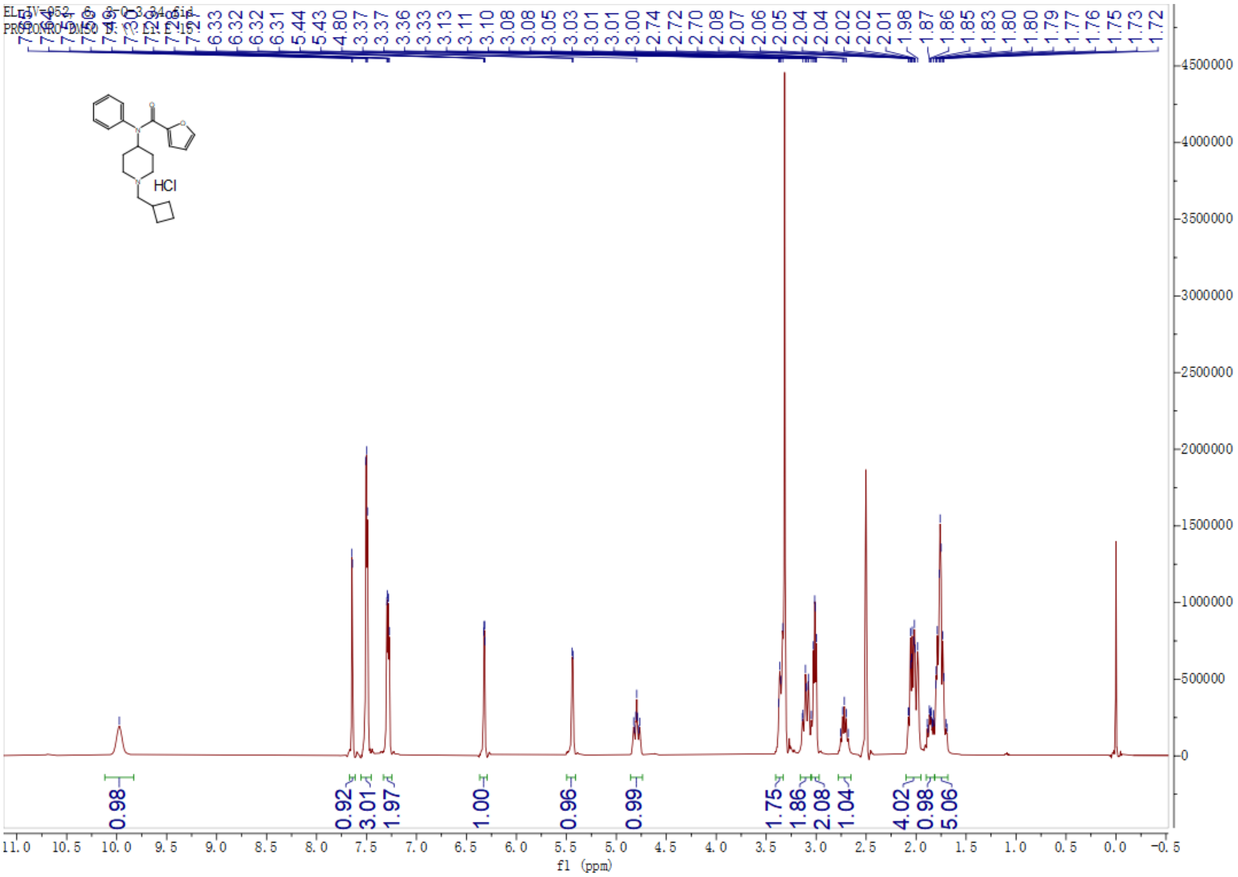

^{1}H NMR (400 MHz, DMSO-d_6) spectrum for compound **3**.

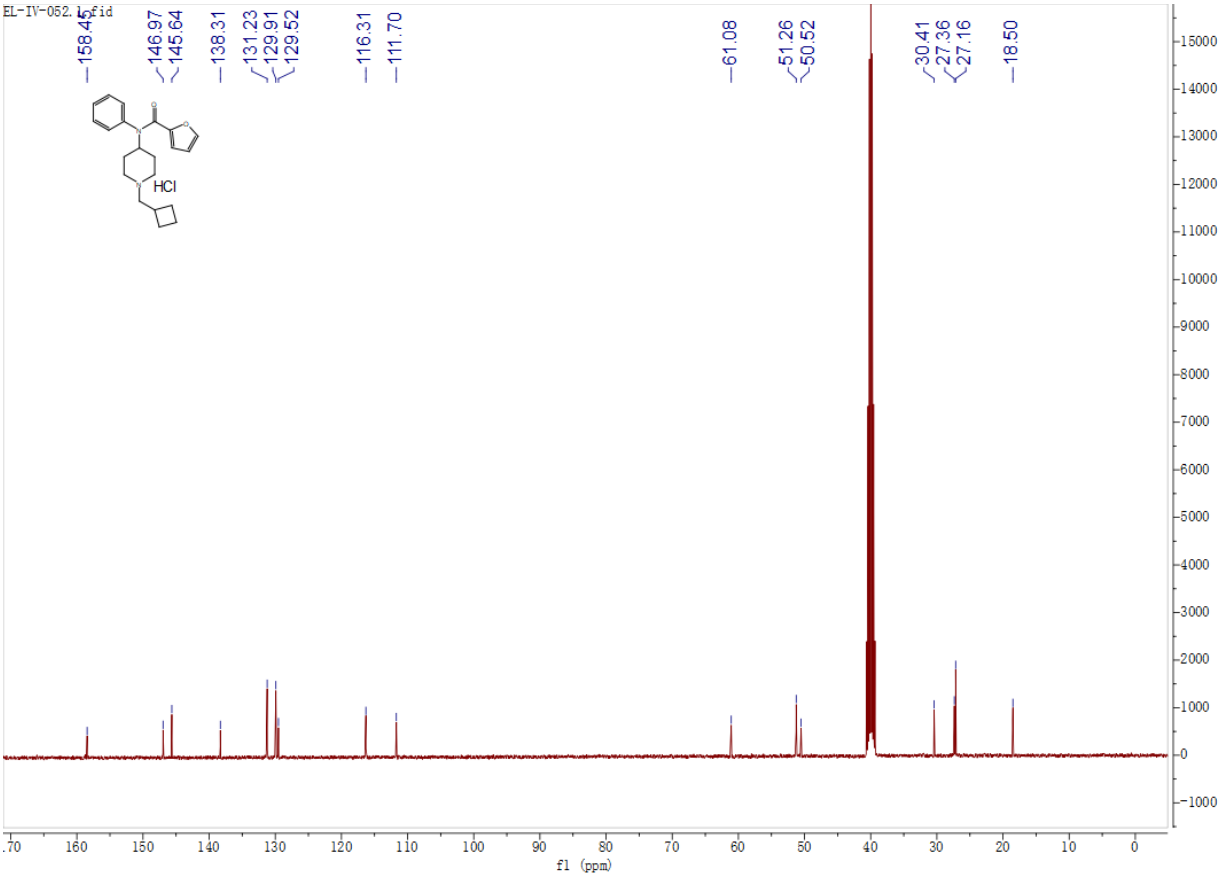

^{13}C NMR (100 MHz, DMSO-d_6) spectrum for compound **3**.

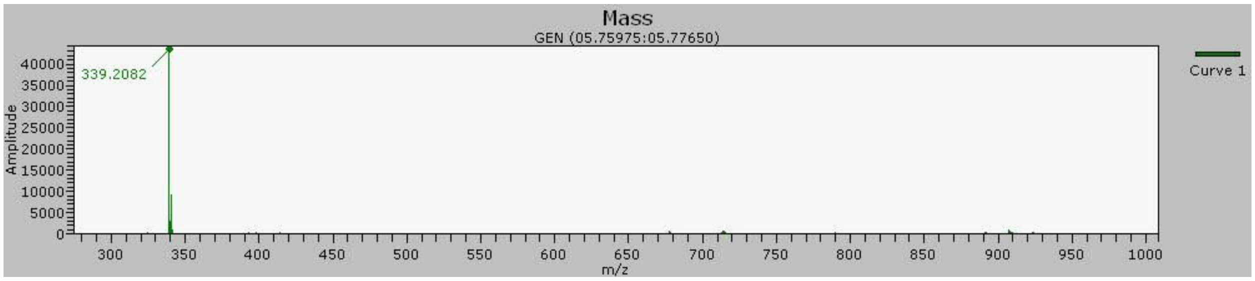

HRMS spectrum for compound **3**.

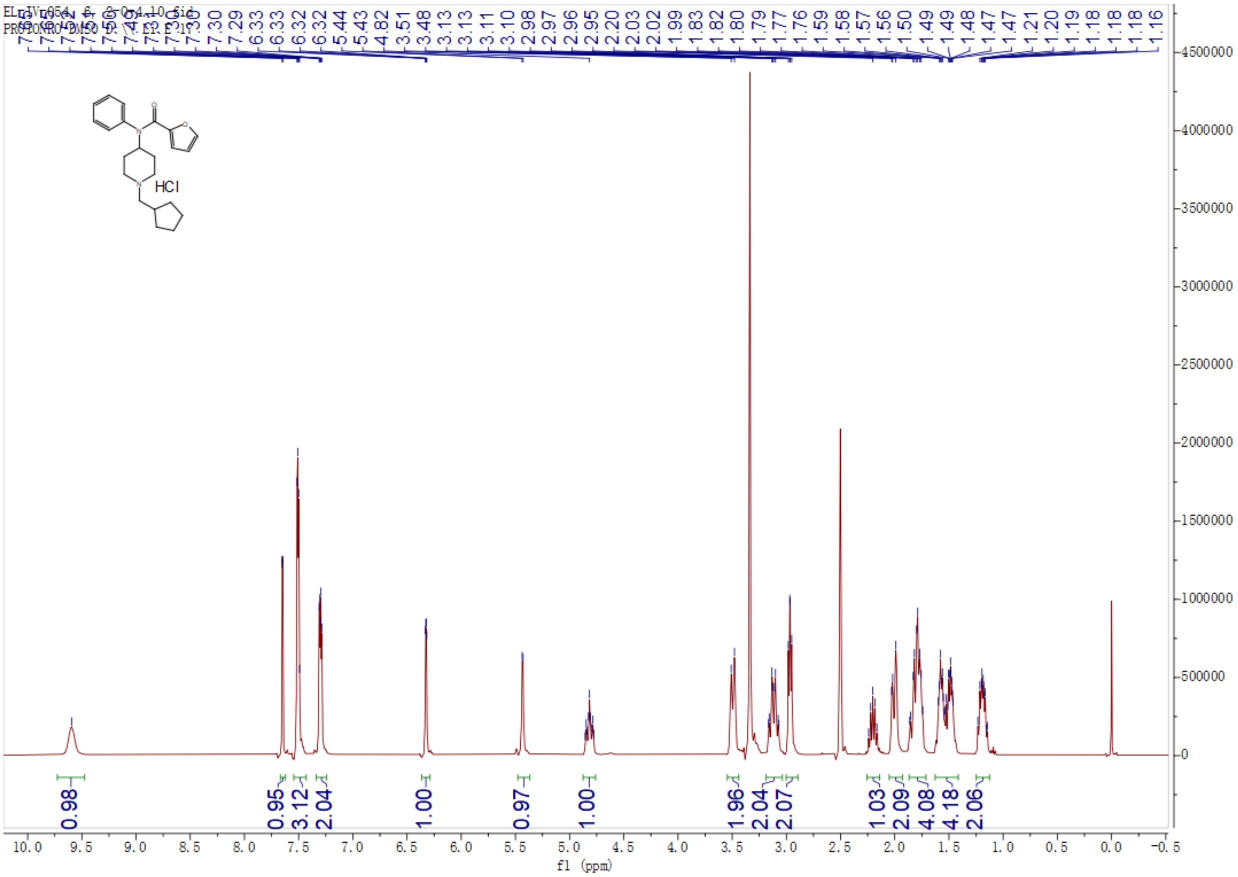

^{1}H NMR (400 MHz, DMSO-d_6) spectrum for compound **4**.

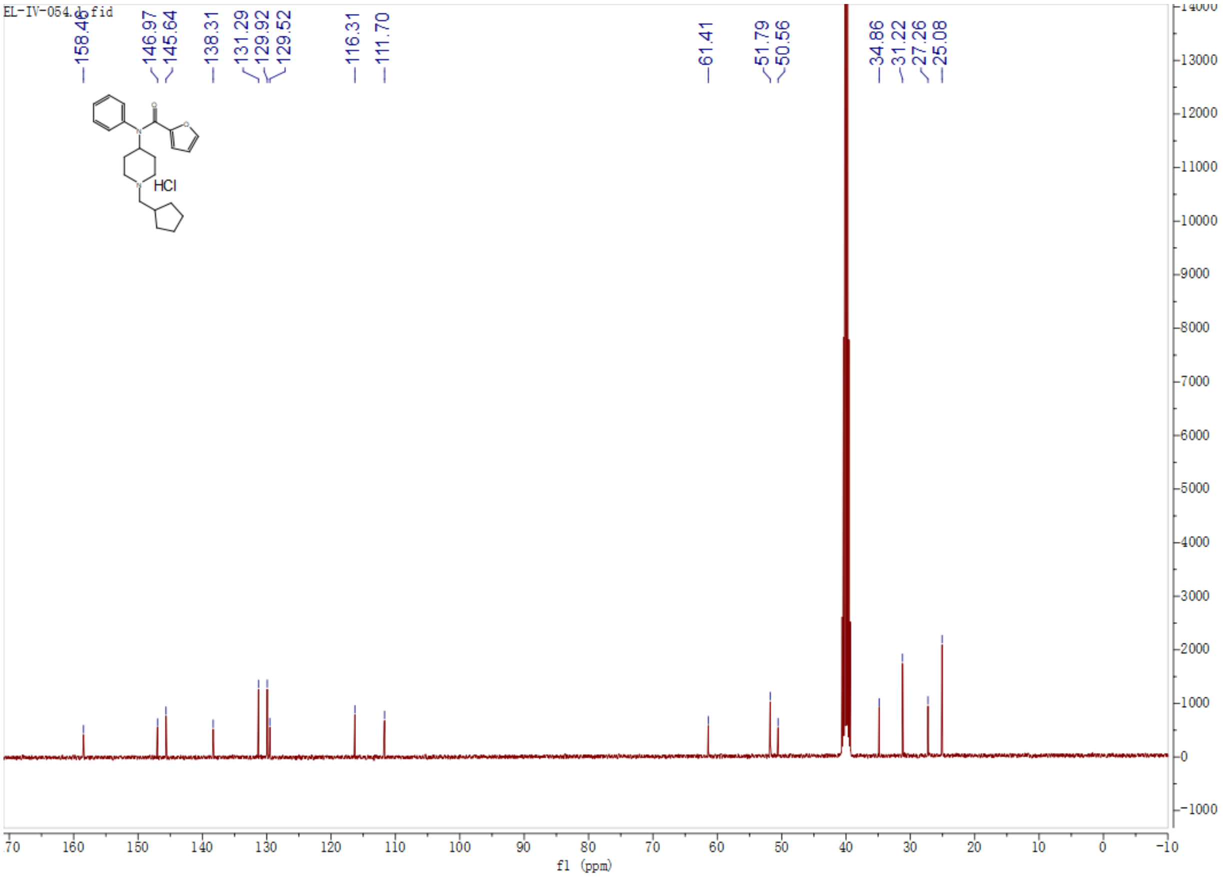

^{13}C NMR (100 MHz, DMSO-d_6) spectrum for compound **4**.

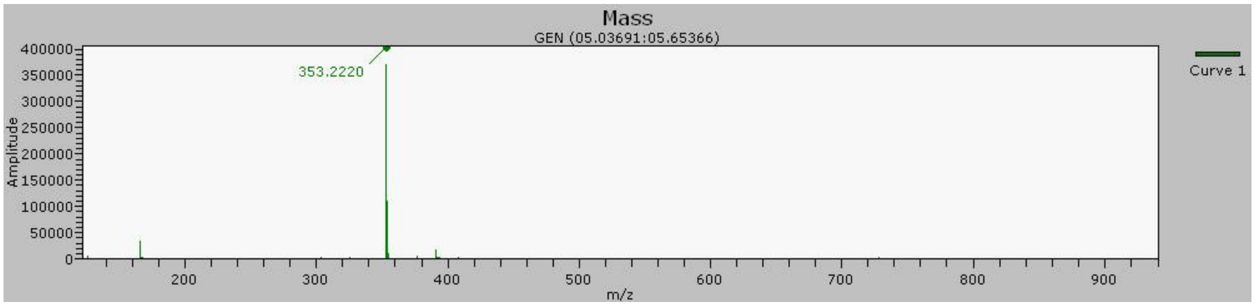

HRMS spectrum for compound **4**.

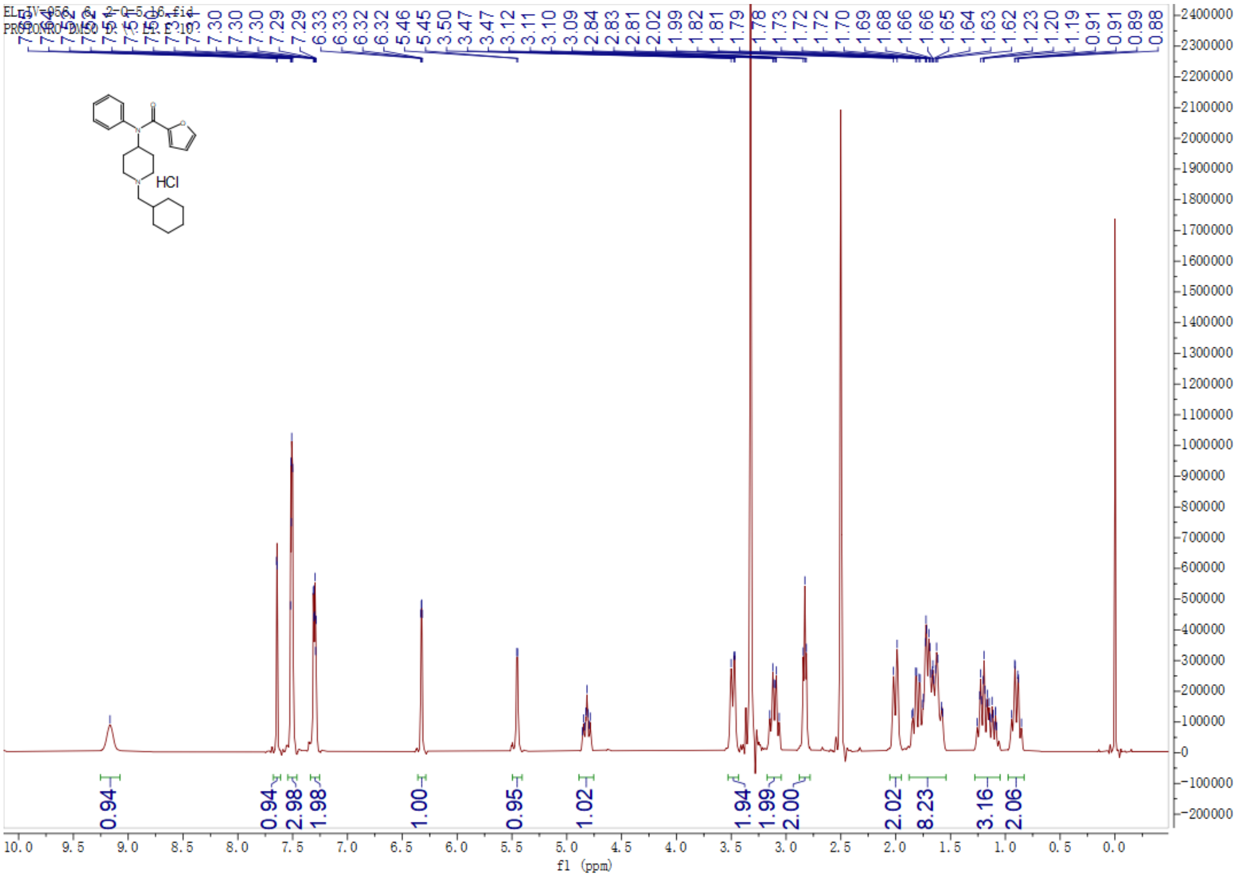

^{1}H NMR (400 MHz, DMSO-d_6) spectrum for compound **5**.

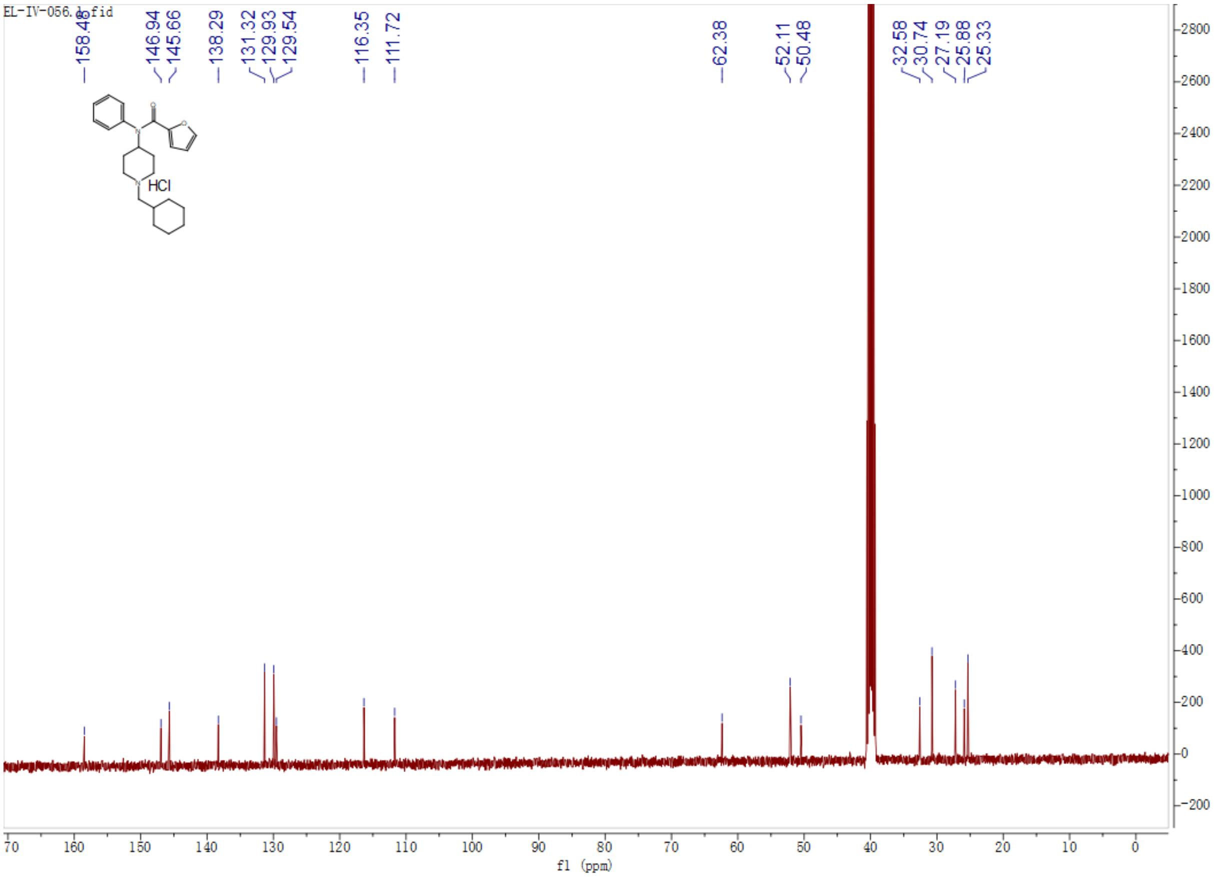

^{13}C NMR (100 MHz, DMSO-d_6) spectrum for compound **5**.

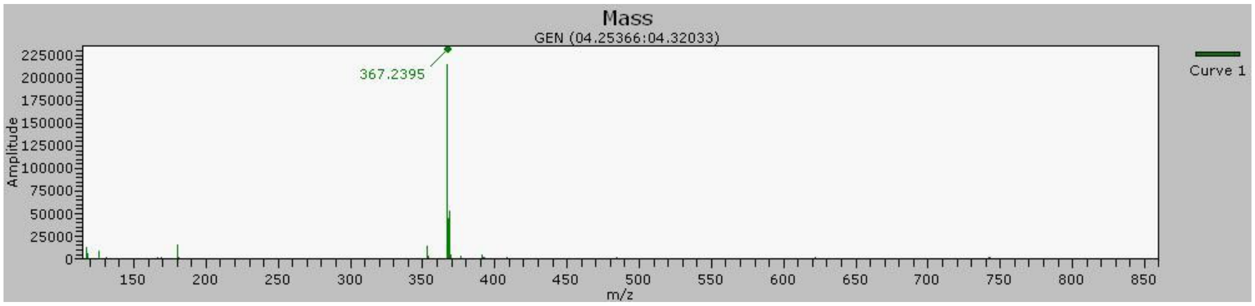

HRMS spectrum for compound **5**.

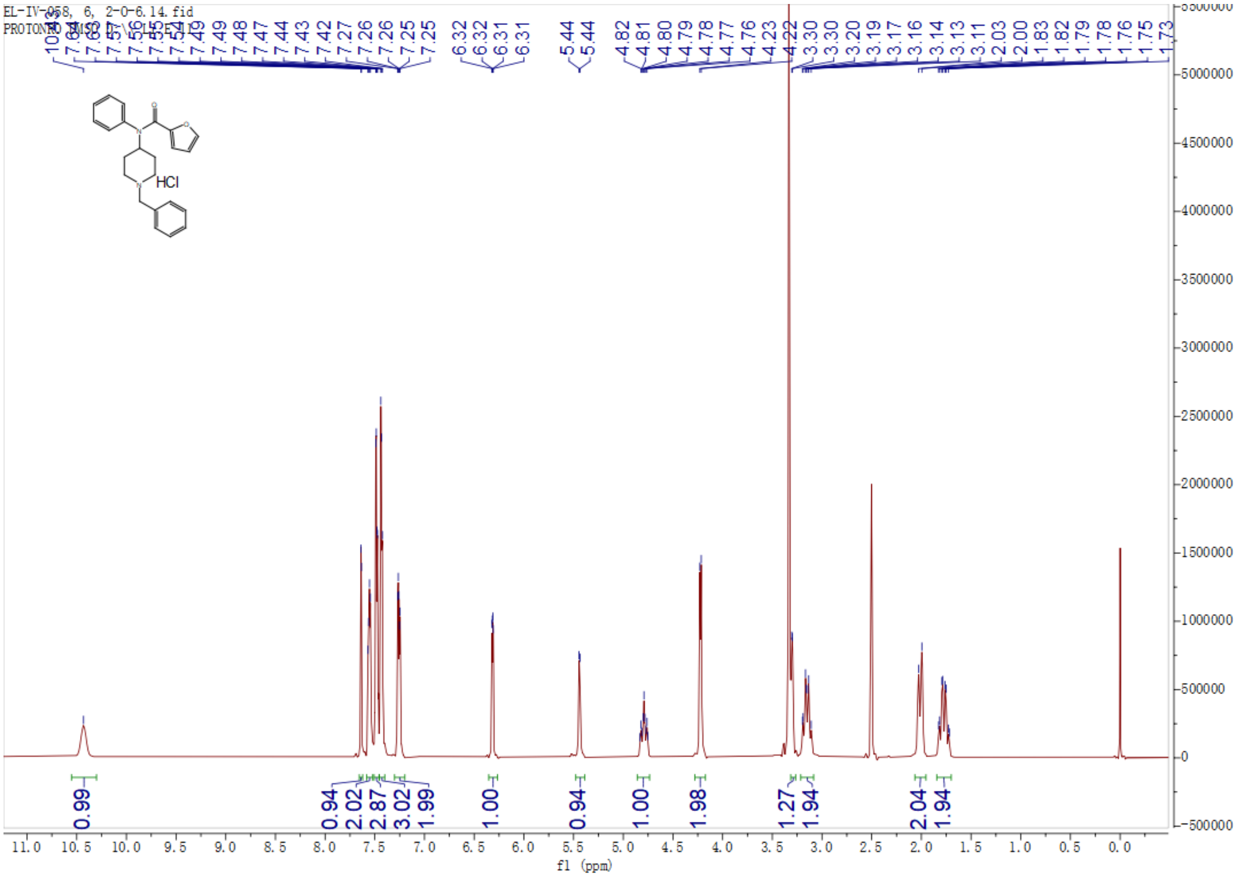

^{1}H NMR (400 MHz, DMSO-d_6) spectrum for compound **6**.

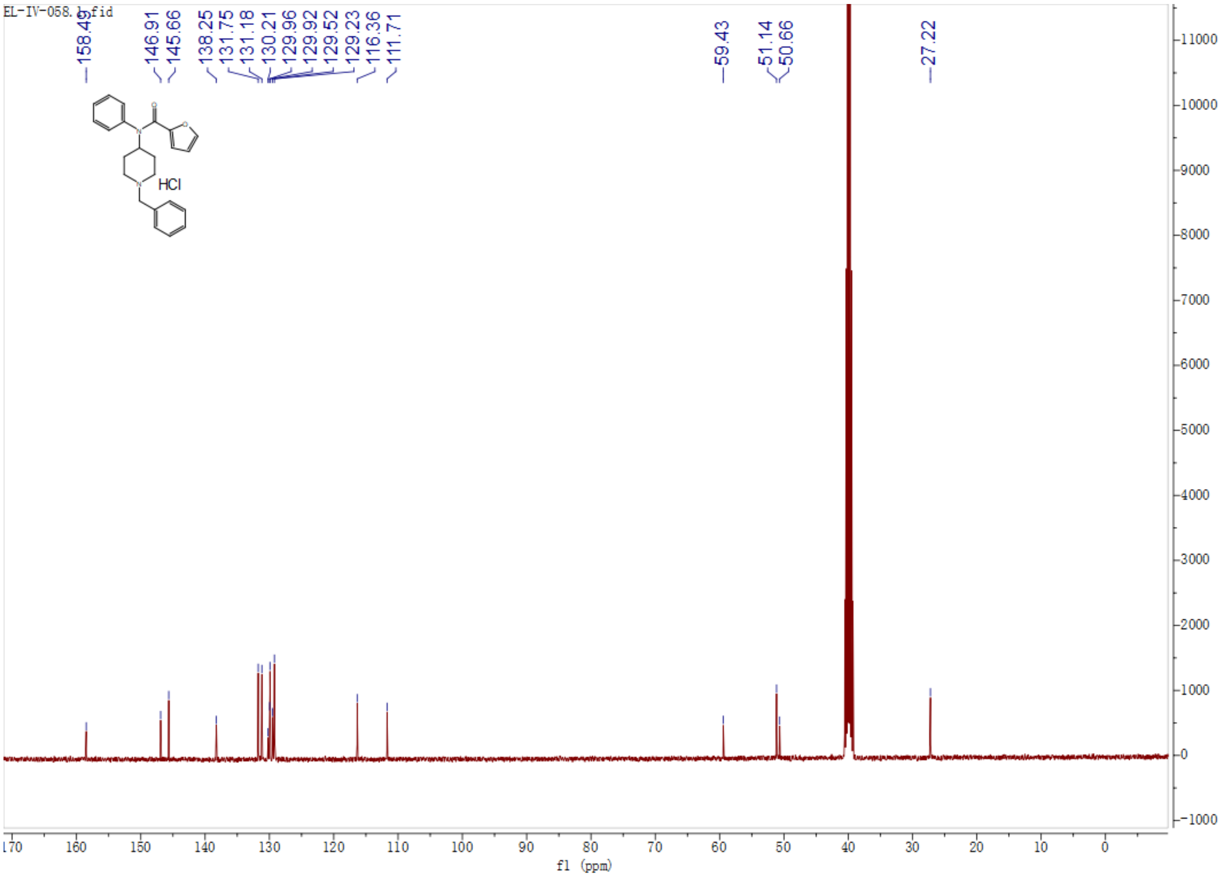

^{13}C NMR (100 MHz, DMSO-d_6) spectrum for compound **6**.

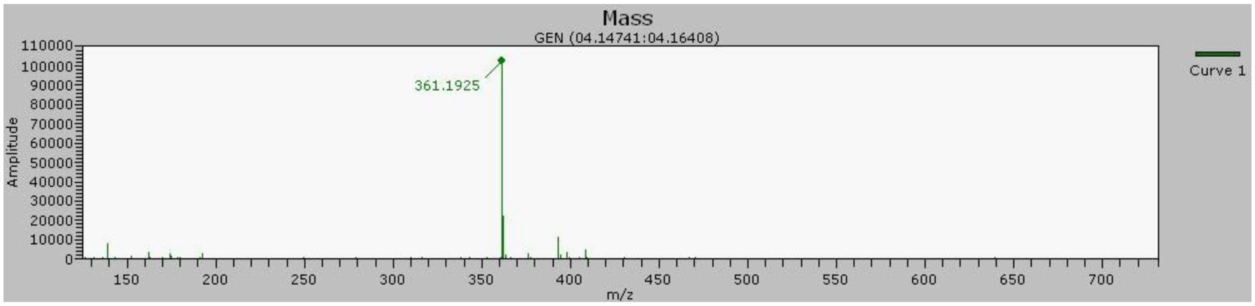

HRMS spectrum for compound **6**.

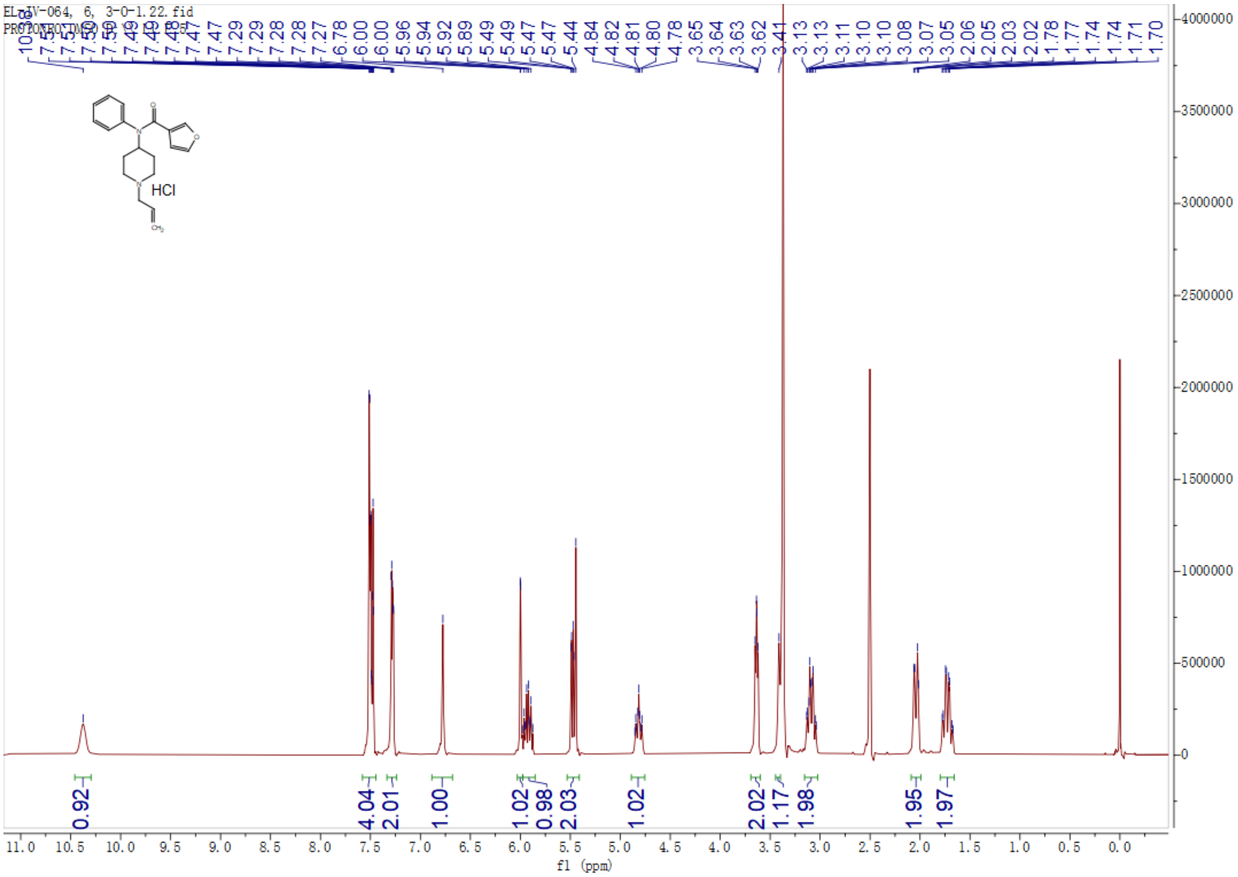

^{1}H NMR (400 MHz, DMSO-d_6) spectrum for compound **7**.

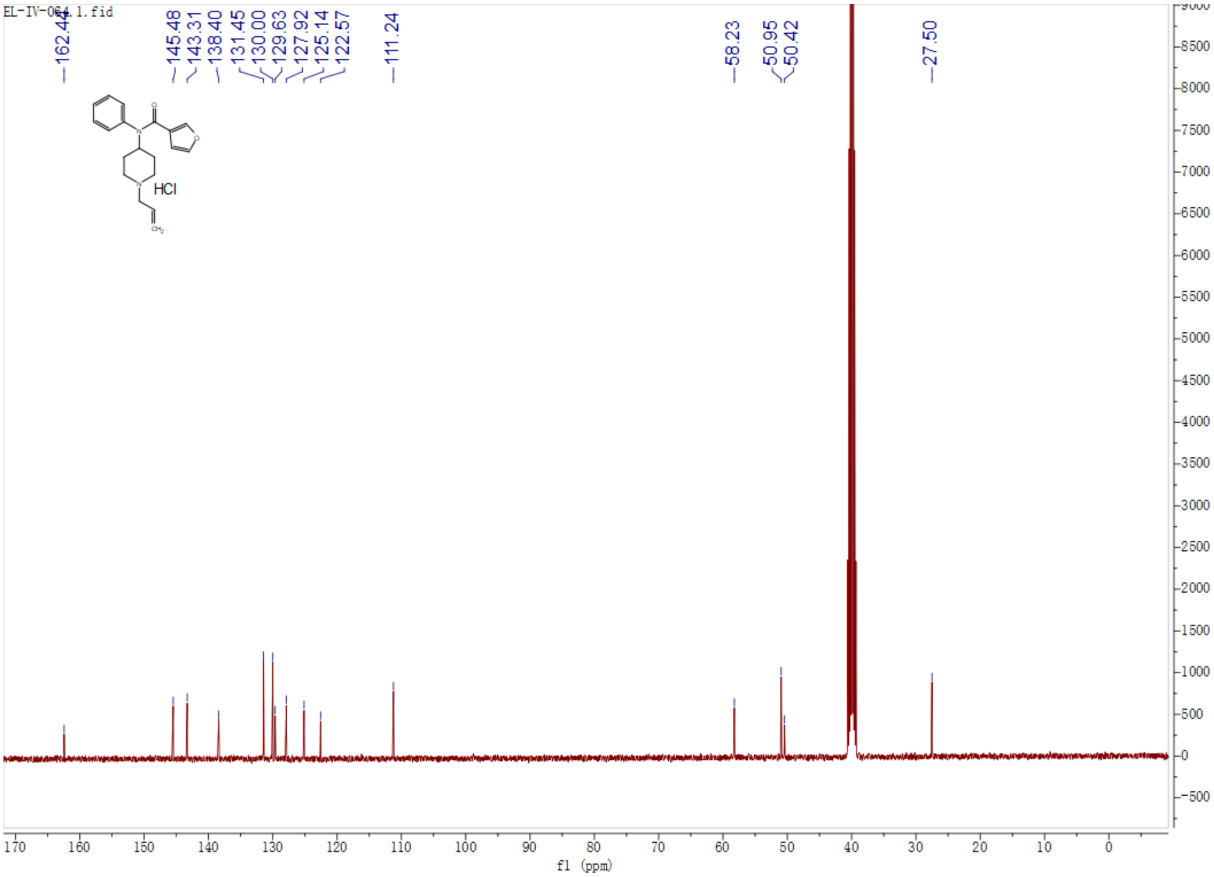

^{13}C NMR (100 MHz, DMSO-d_6) spectrum for compound **7**.

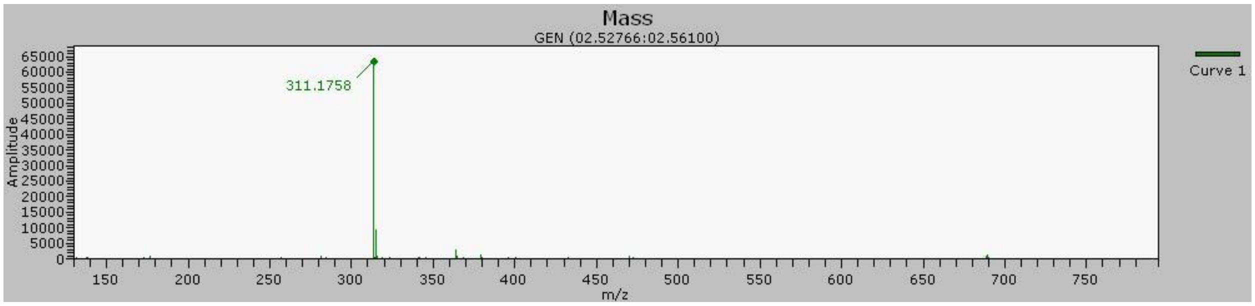

HRMS spectrum for compound **7**.

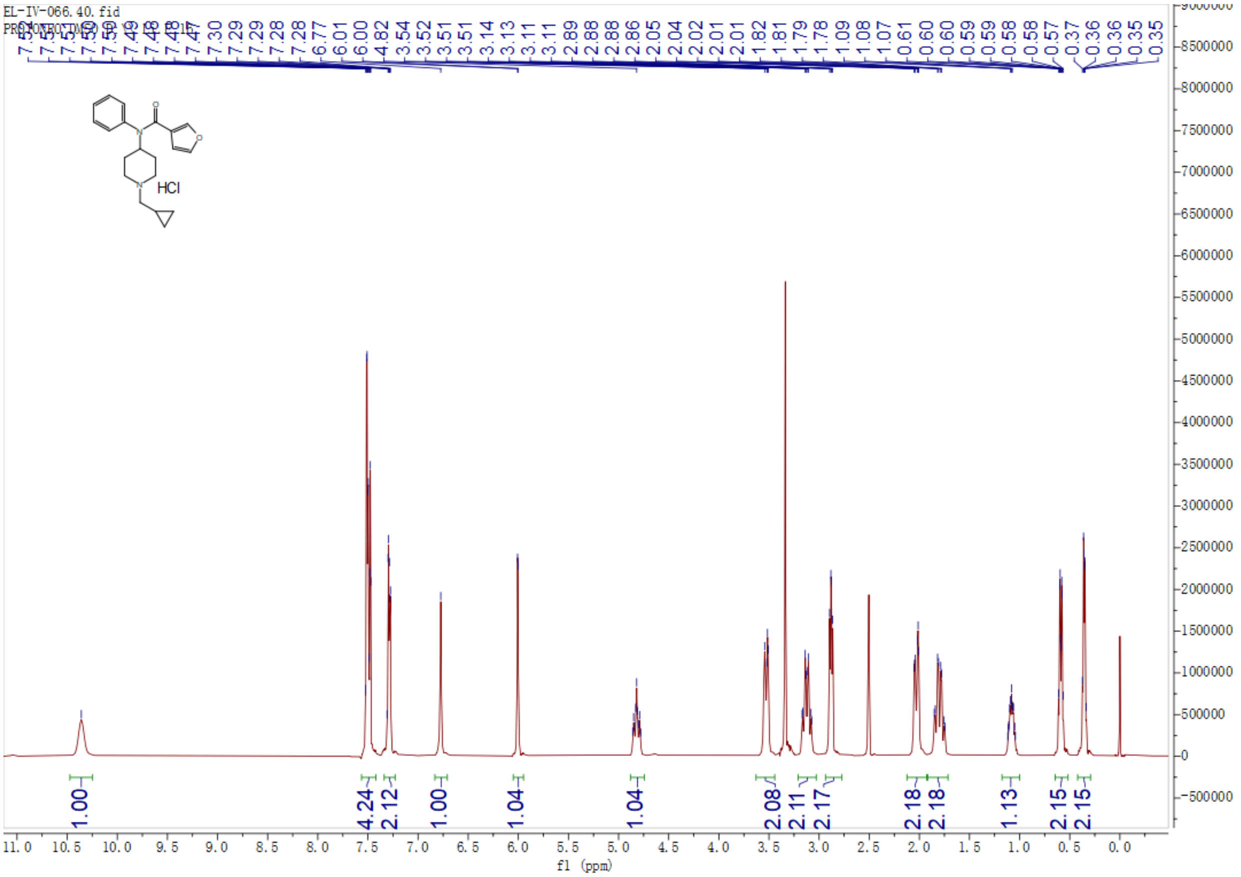

^{1}H NMR (400 MHz, DMSO-d_6) spectrum for compound **8**.

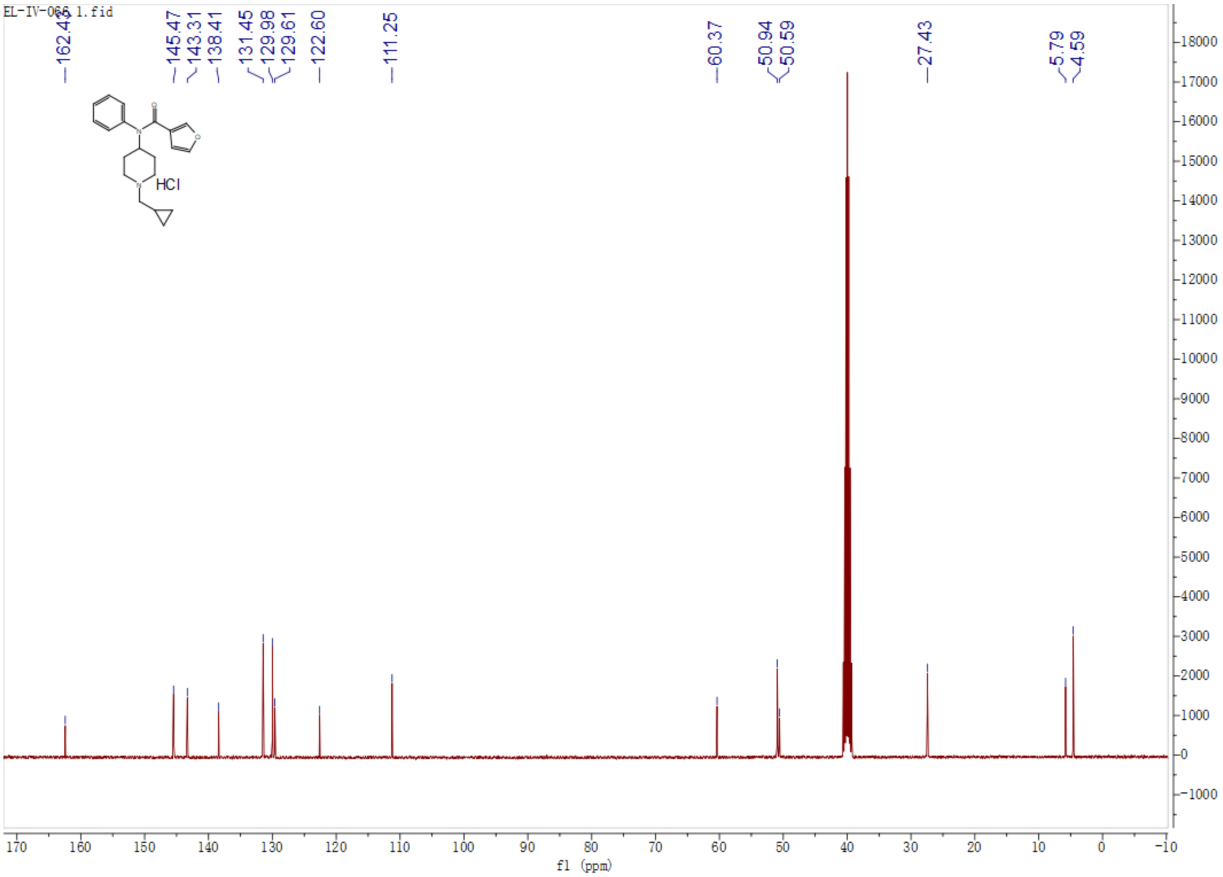

^{13}C NMR (100 MHz, DMSO-d_6) spectrum for compound **8**.

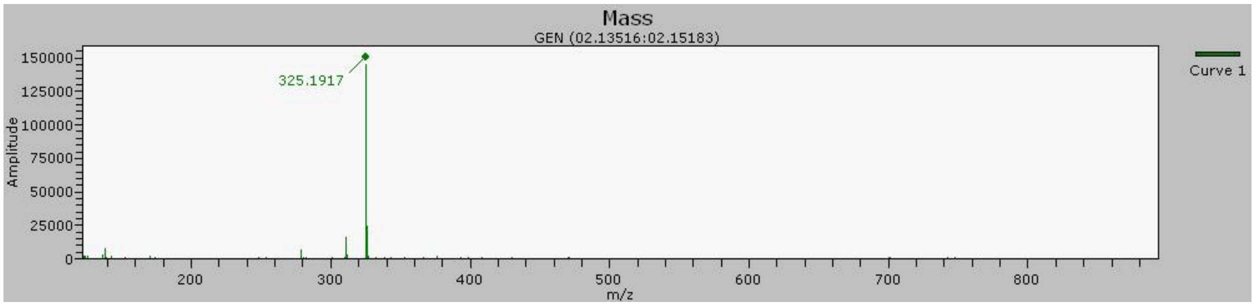

HRMS spectrum for compound **8**.

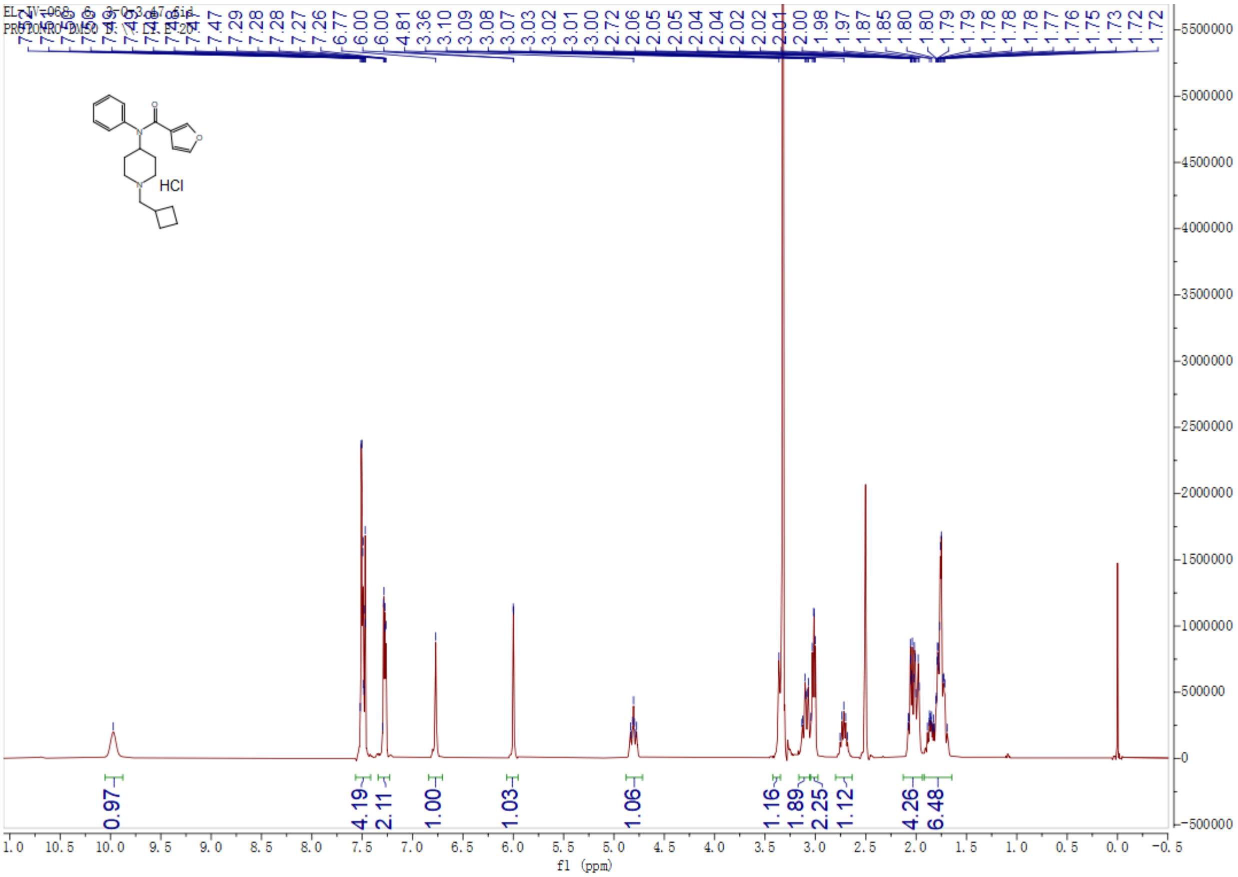

^{1}H NMR (400 MHz, DMSO-d_6) spectrum for compound **9**.

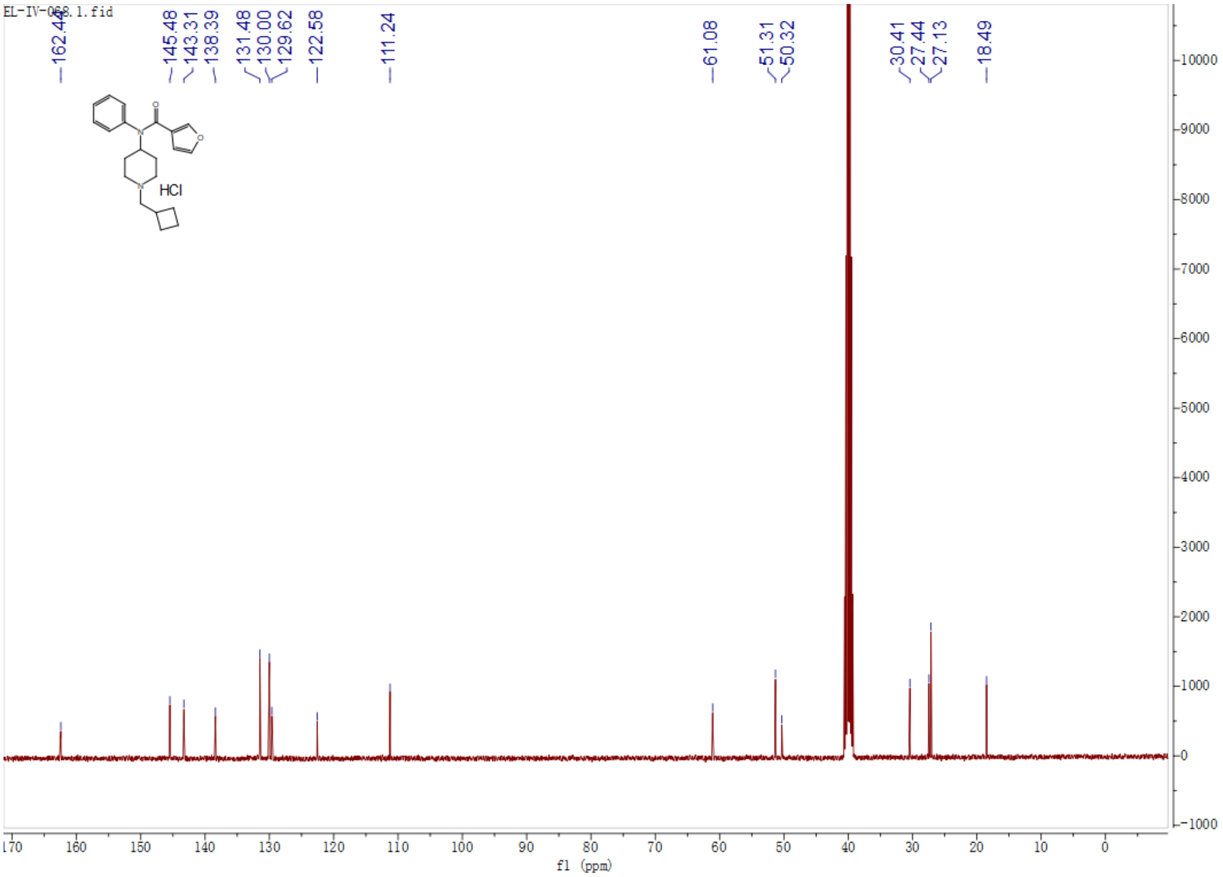

^{13}C NMR (100 MHz, DMSO-d_6) spectrum for compound **9**.

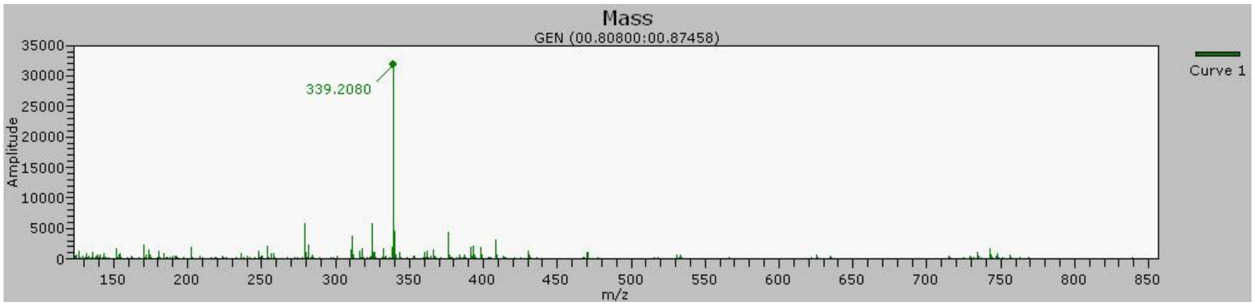

HRMS spectrum for compound **9**.

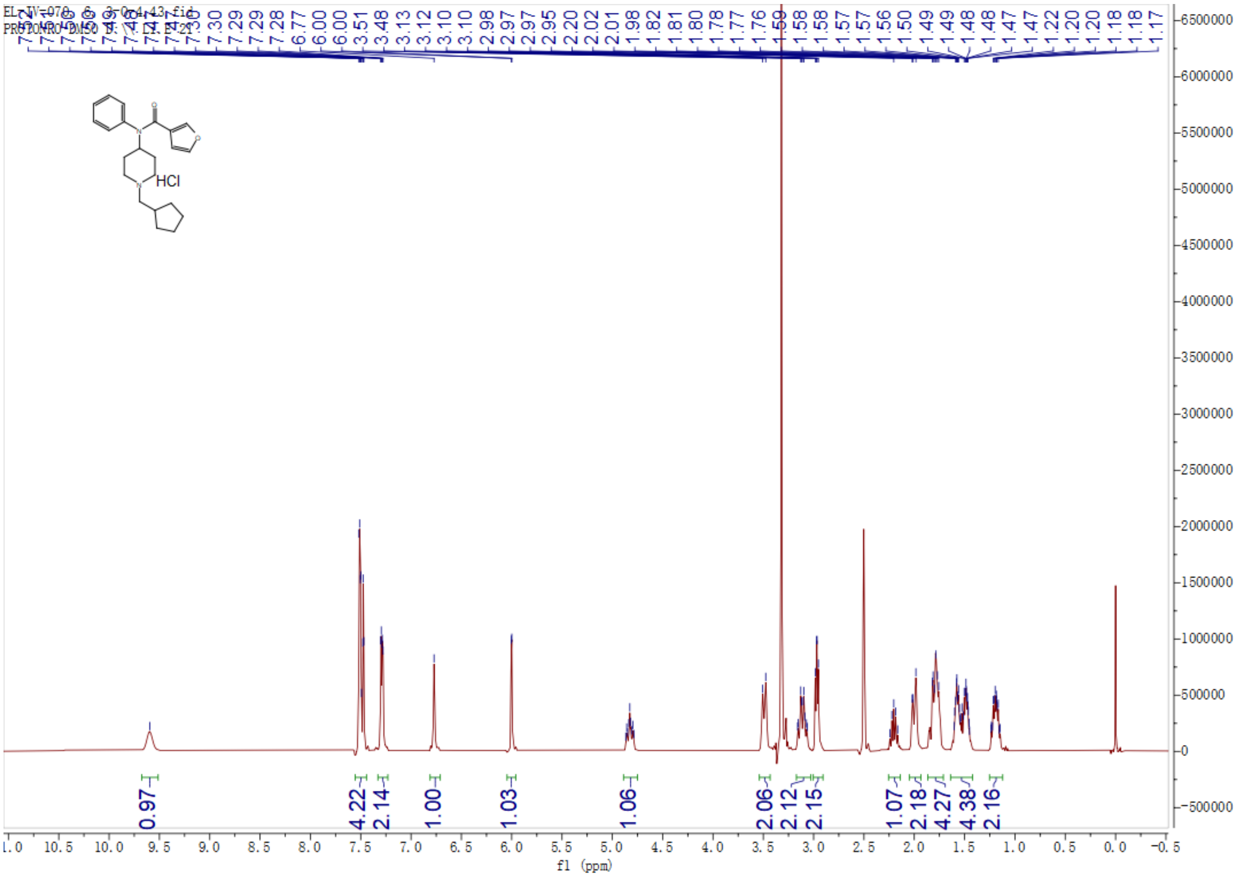

^{1}H NMR (400 MHz, DMSO-d_6) spectrum for compound **10**.

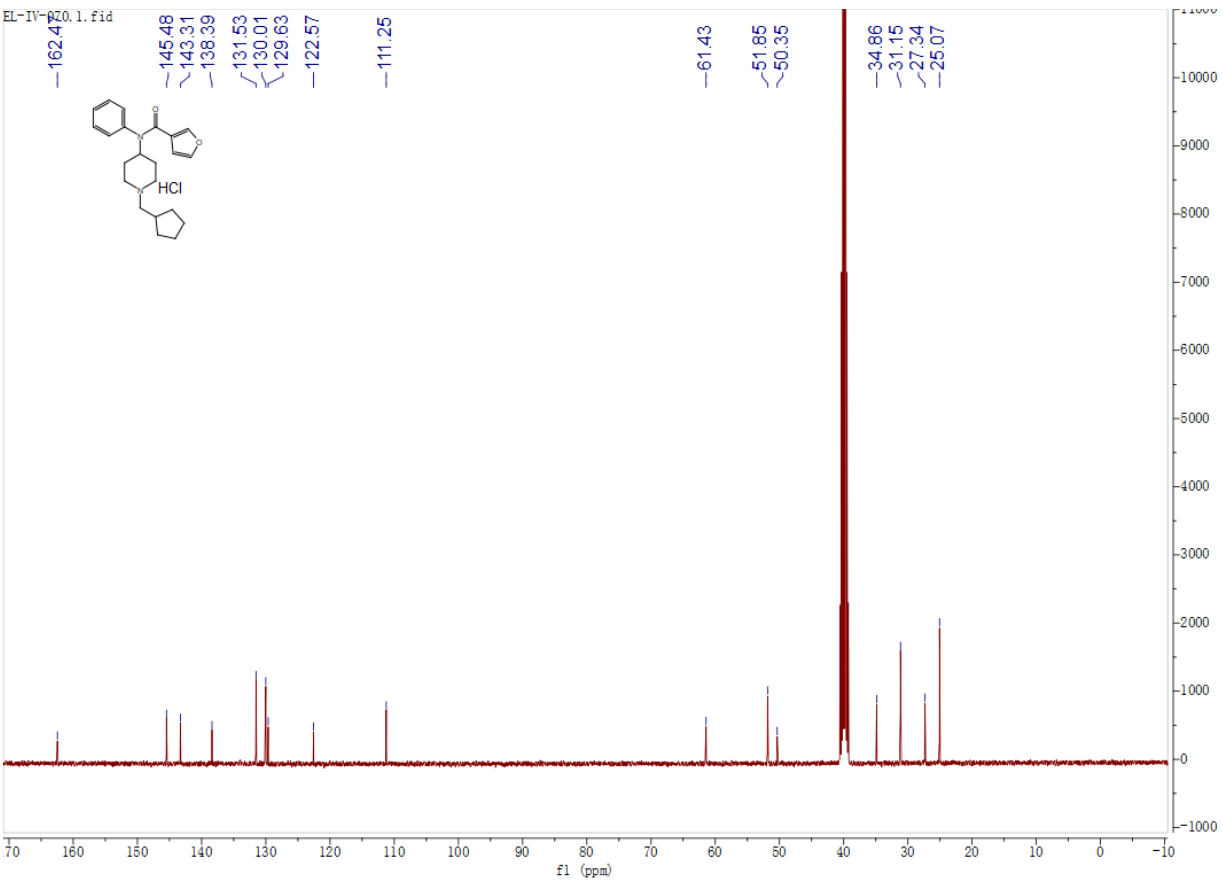

^{13}C NMR (100 MHz, DMSO-d_6) spectrum for compound **10**.

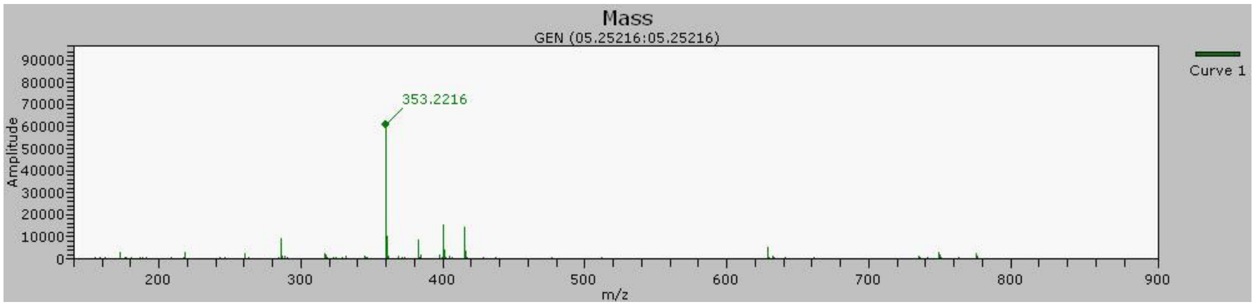

HRMS spectrum for compound **10**.

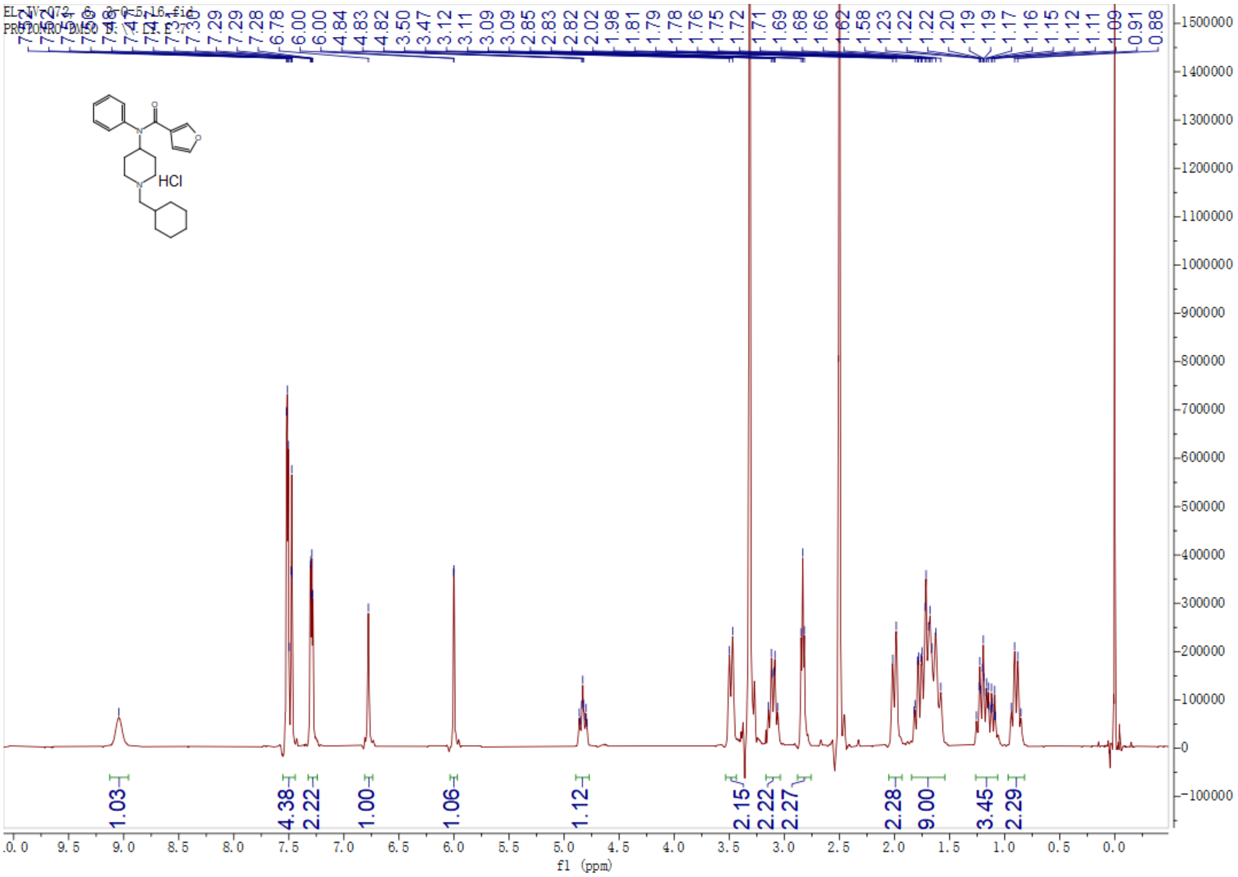

^{1}H NMR (400 MHz, DMSO-d_6) spectrum for compound **11**.

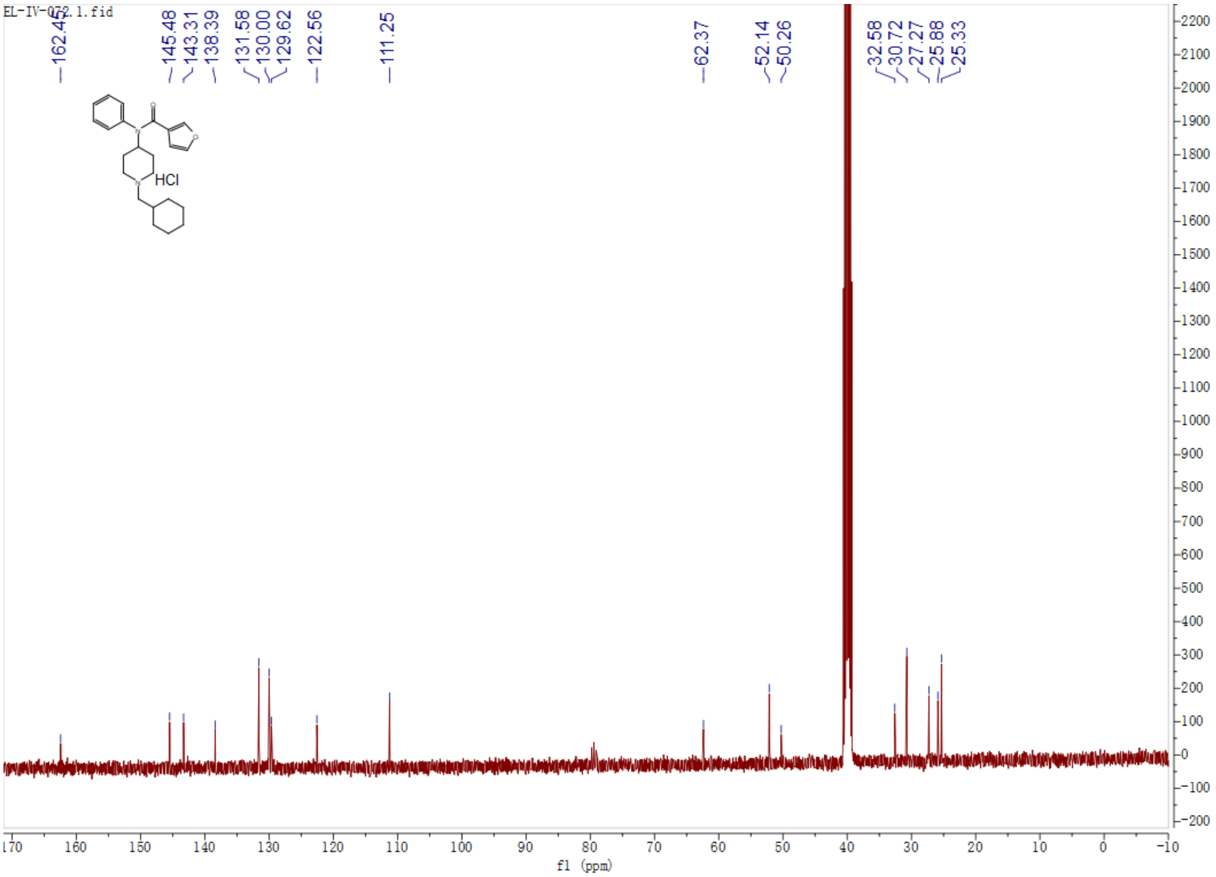

^{13}C NMR (100 MHz, DMSO-d_6) spectrum for compound **11**.

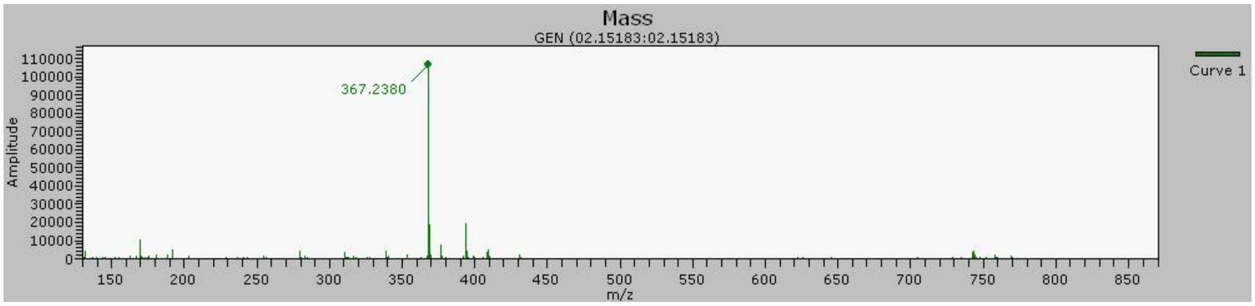

HRMS spectrum for compound **11**.

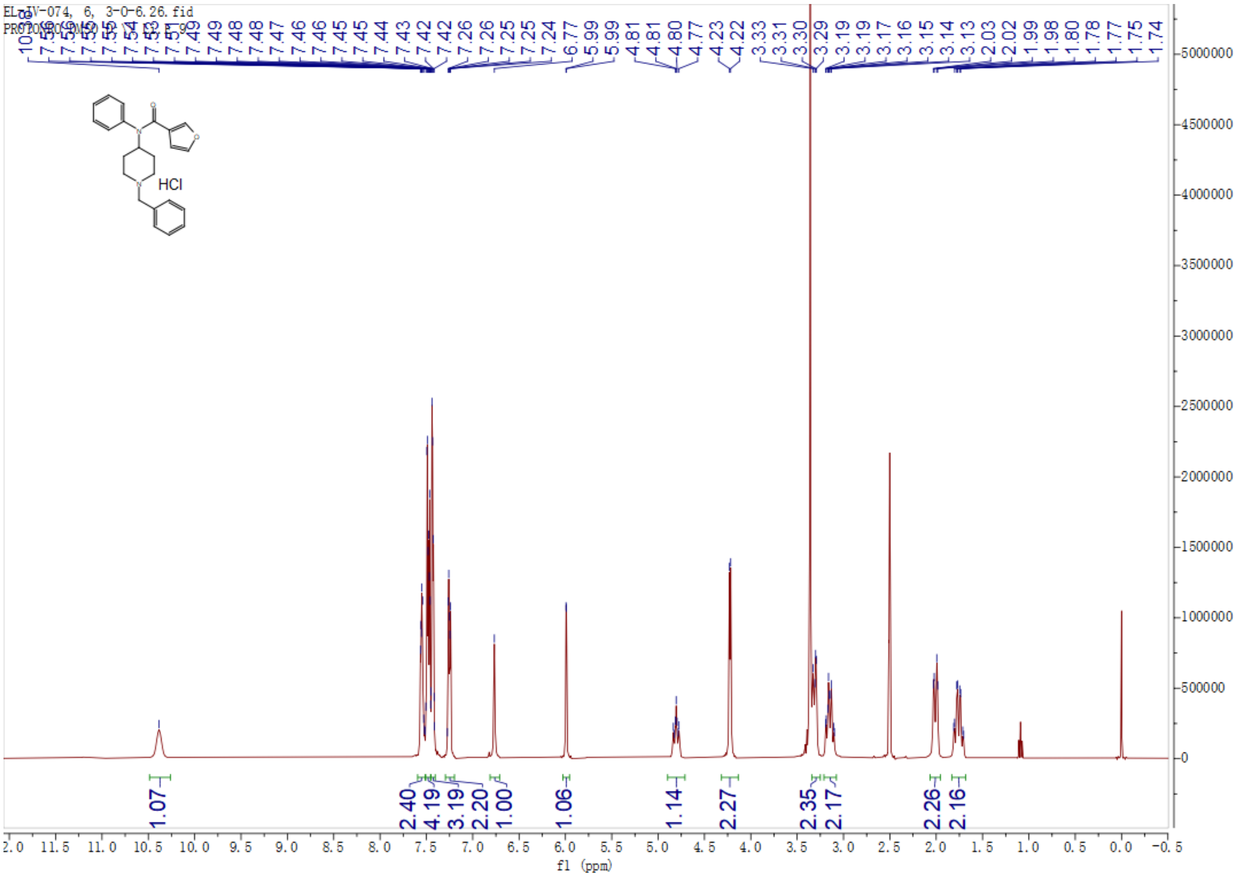

^{1}H NMR (400 MHz, DMSO-d_6) spectrum for compound **12**.

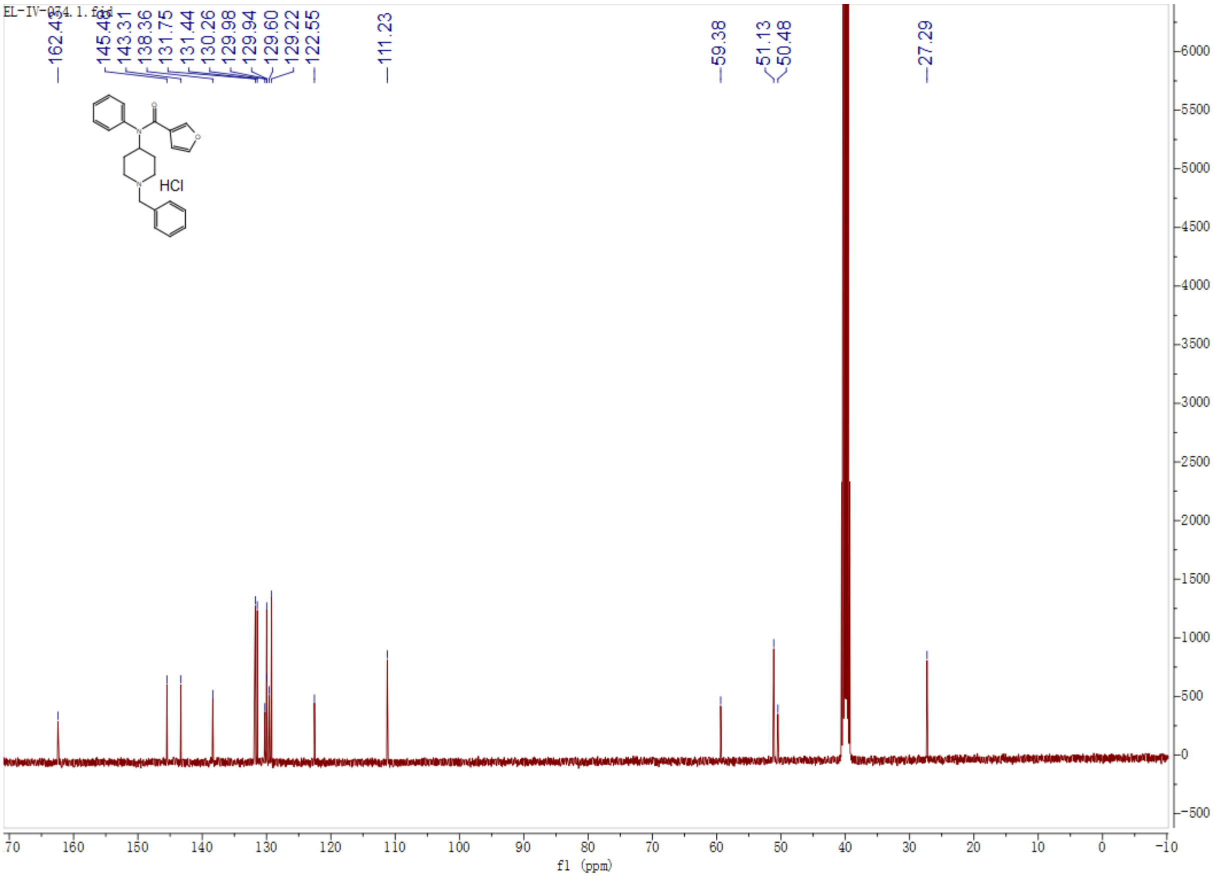

^{13}C NMR (100 MHz, DMSO-d_6) spectrum for compound **12**.

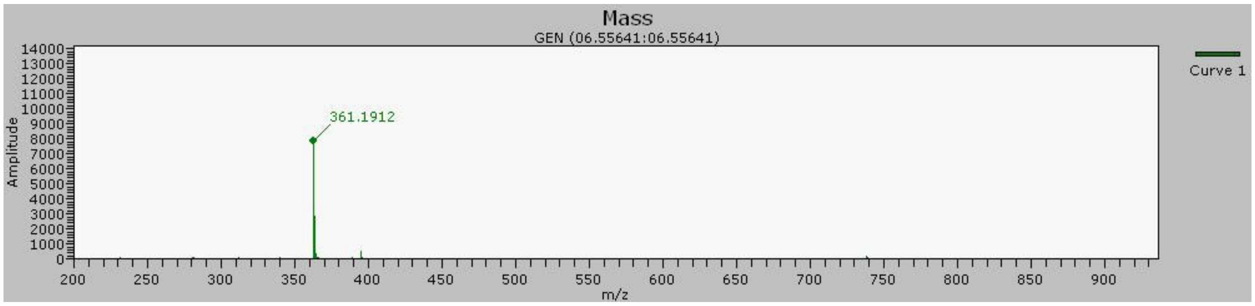

HRMS spectrum for compound **12**.

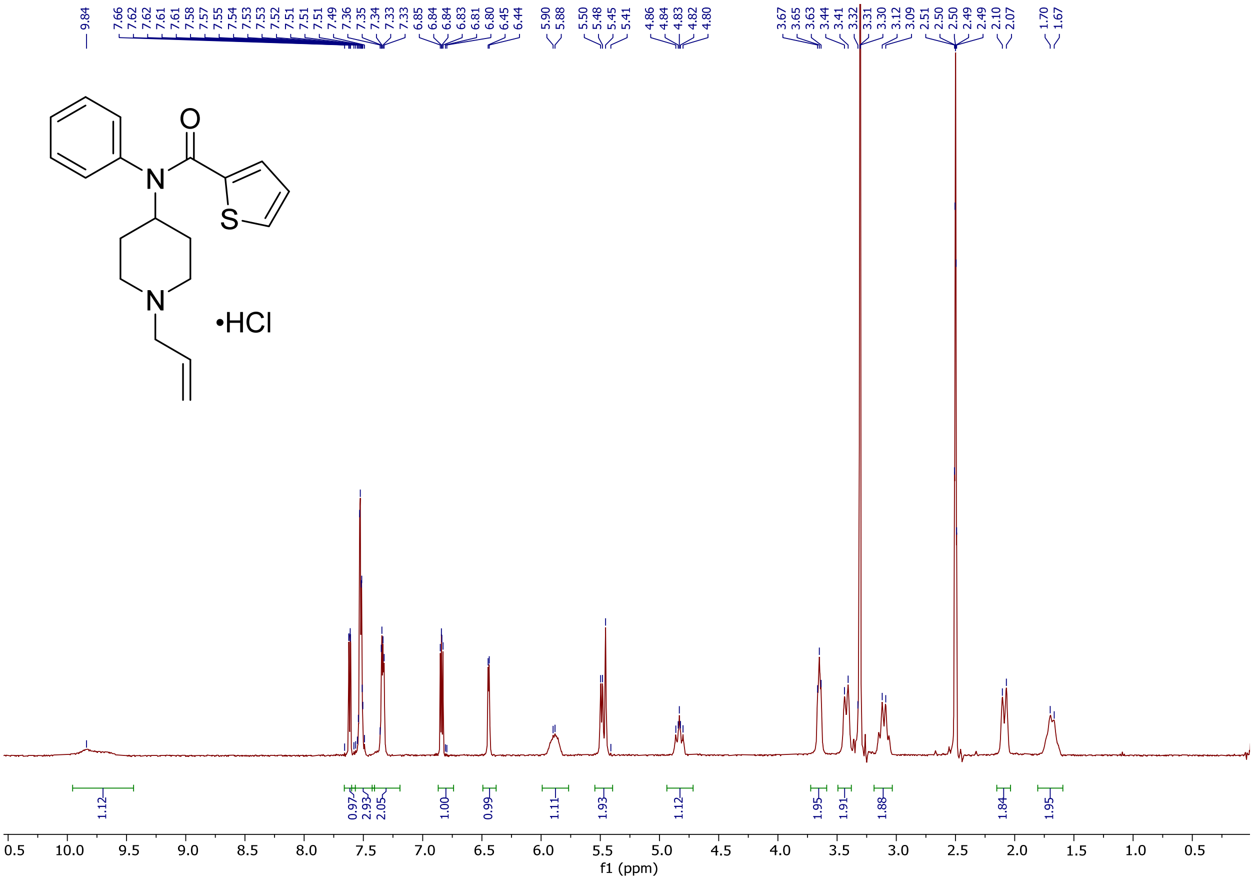

^{1}H NMR (400 MHz, DMSO-d_6) spectrum for compound **13**.

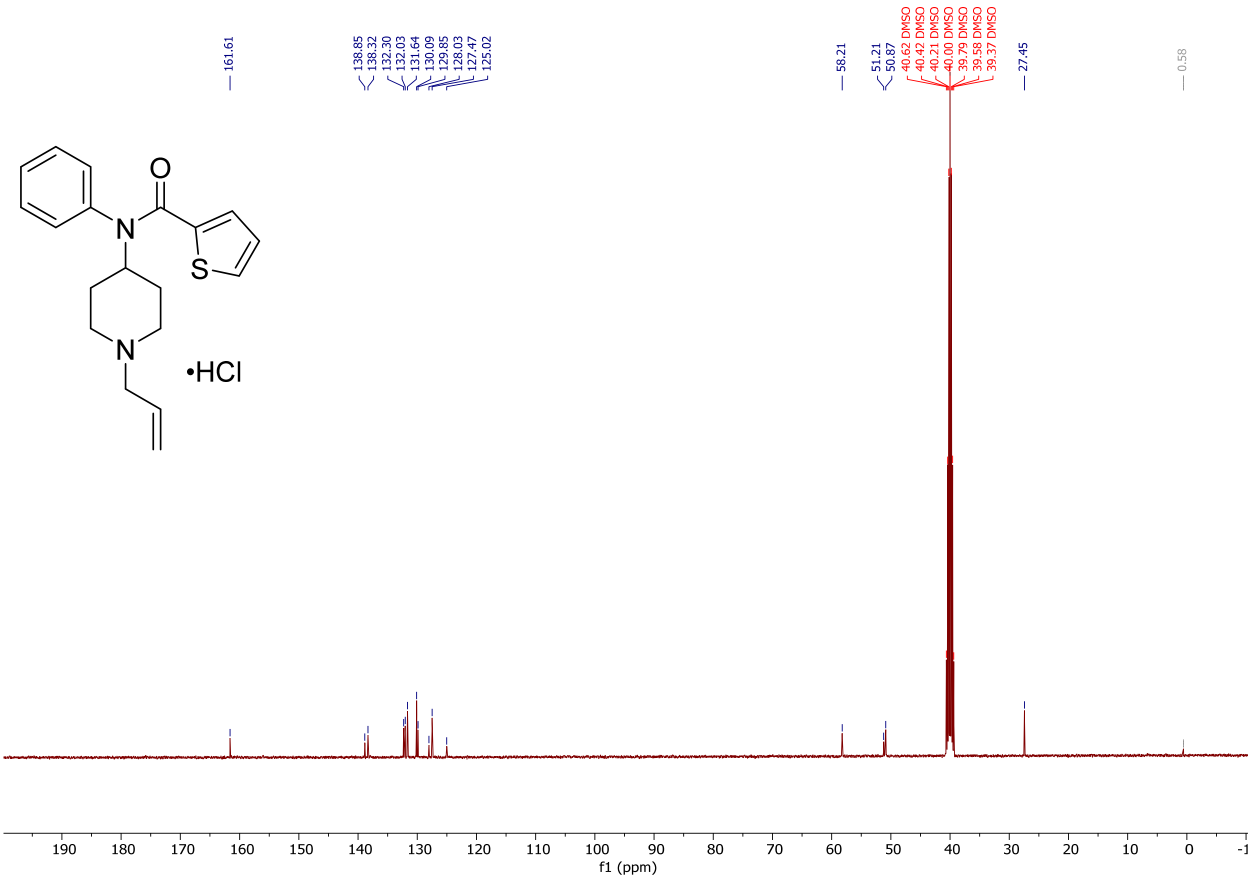

^{13}C NMR (100 MHz, DMSO-d_6) spectrum for compound **13**.

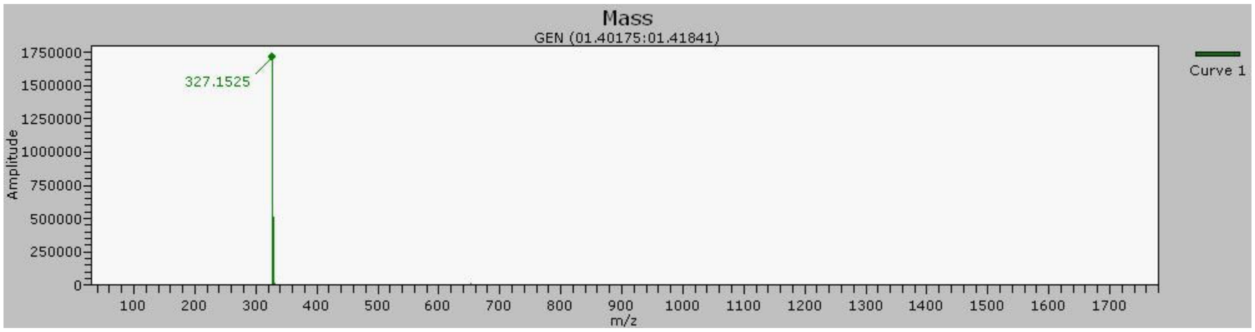

HRMS spectrum for compound **13**.

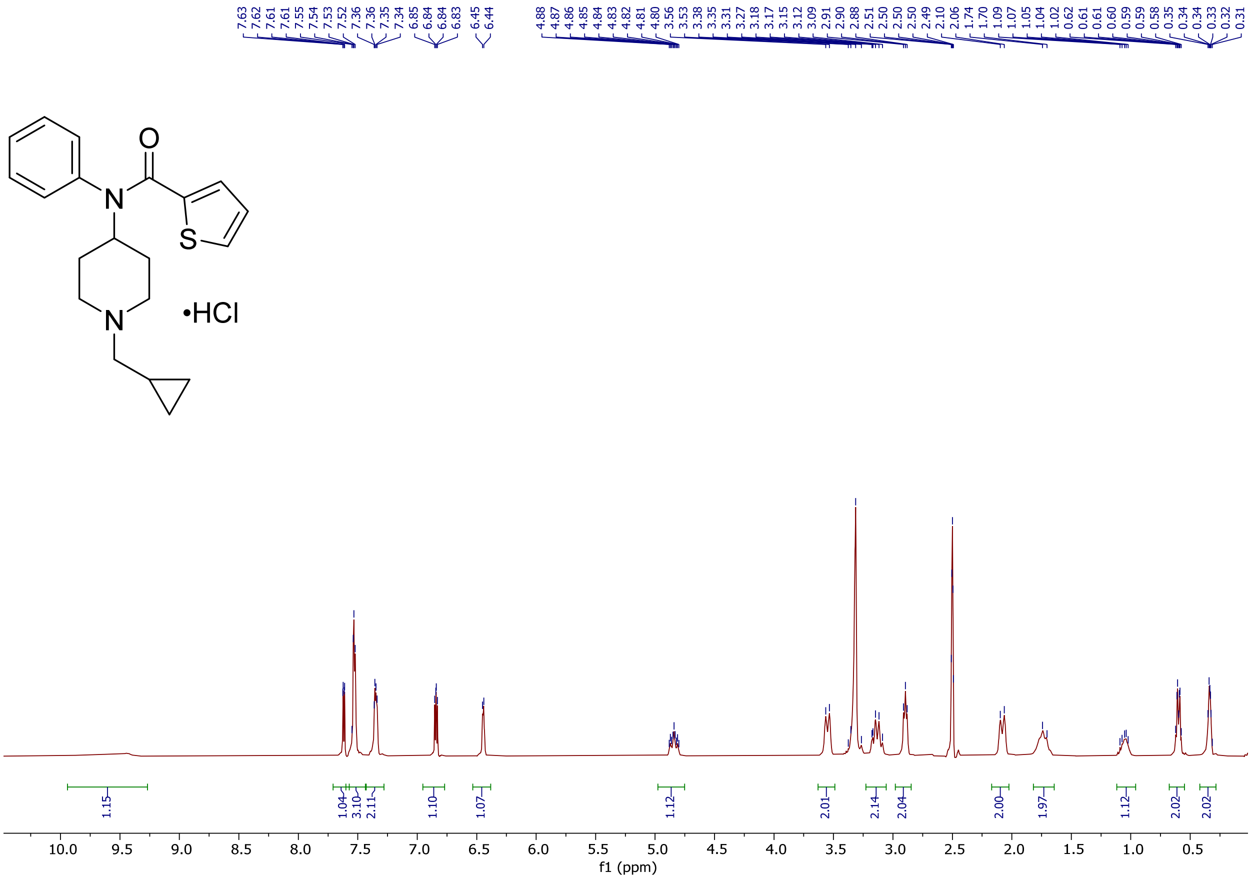

^{1}H NMR (400 MHz, DMSO-d_6) spectrum for compound **14**.

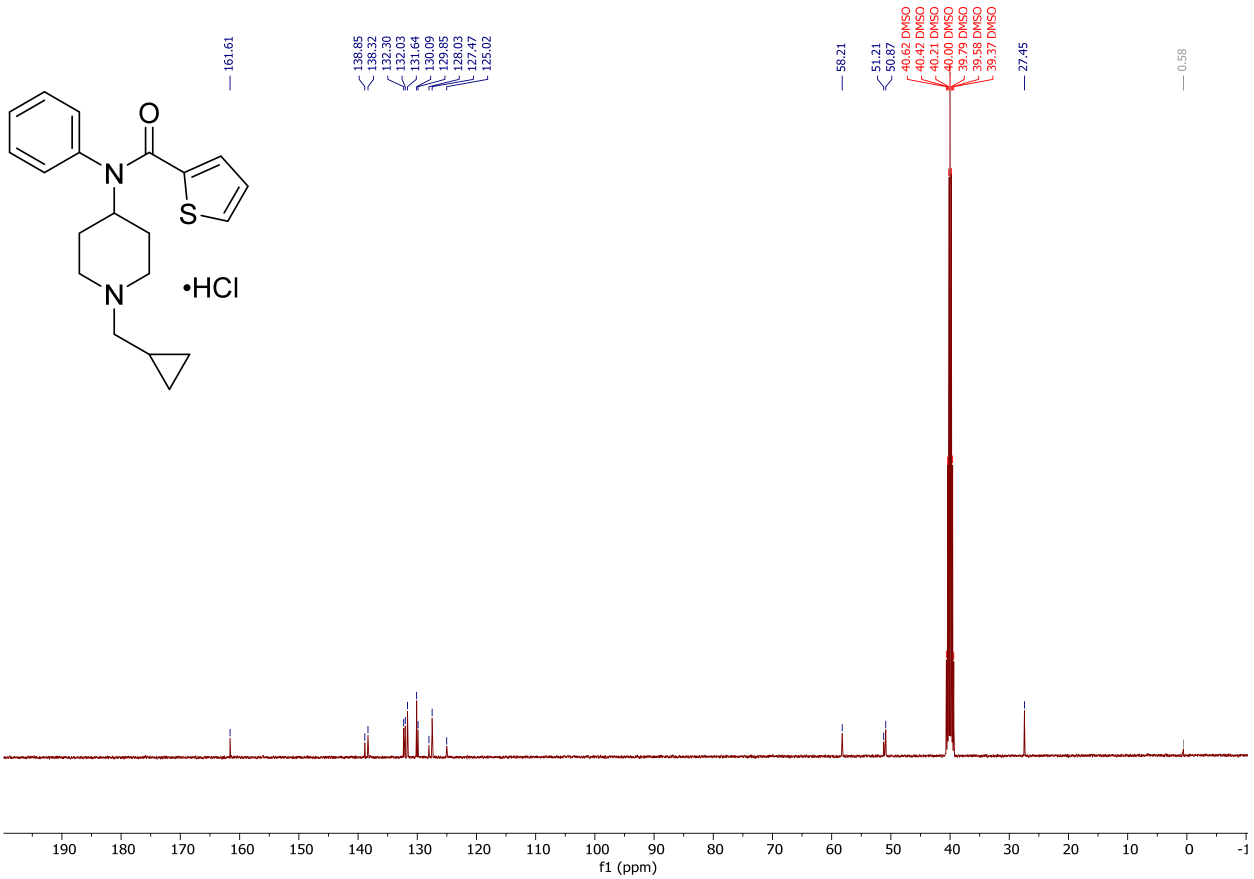

^{13}C NMR (100 MHz, DMSO-d_6) spectrum for compound **14**.

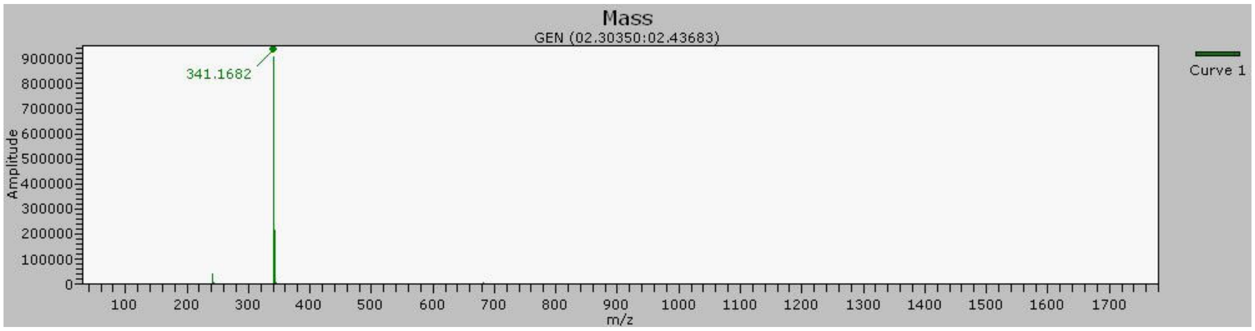

HRMS spectrum for compound **14**.

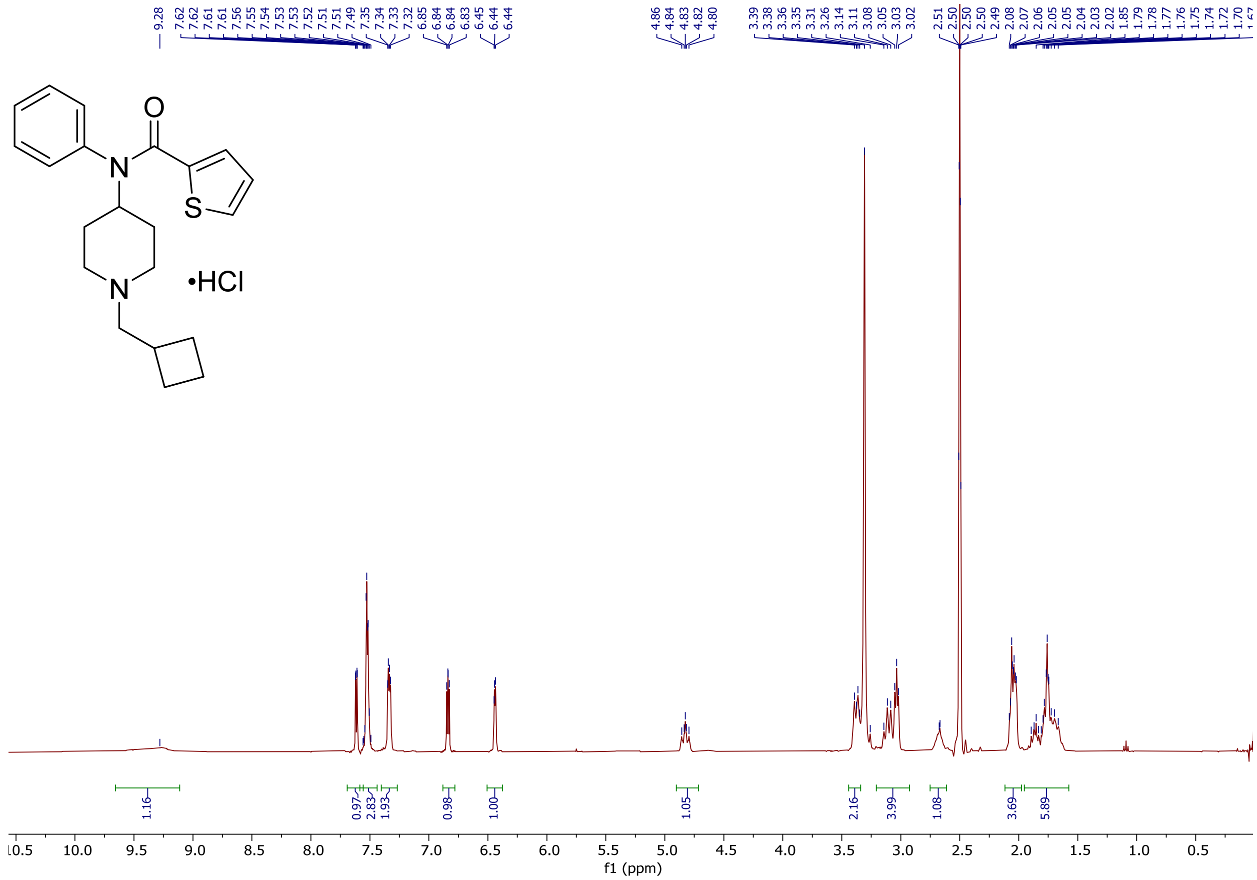

^{1}H NMR (400 MHz, DMSO-d_6) spectrum for compound **15**.

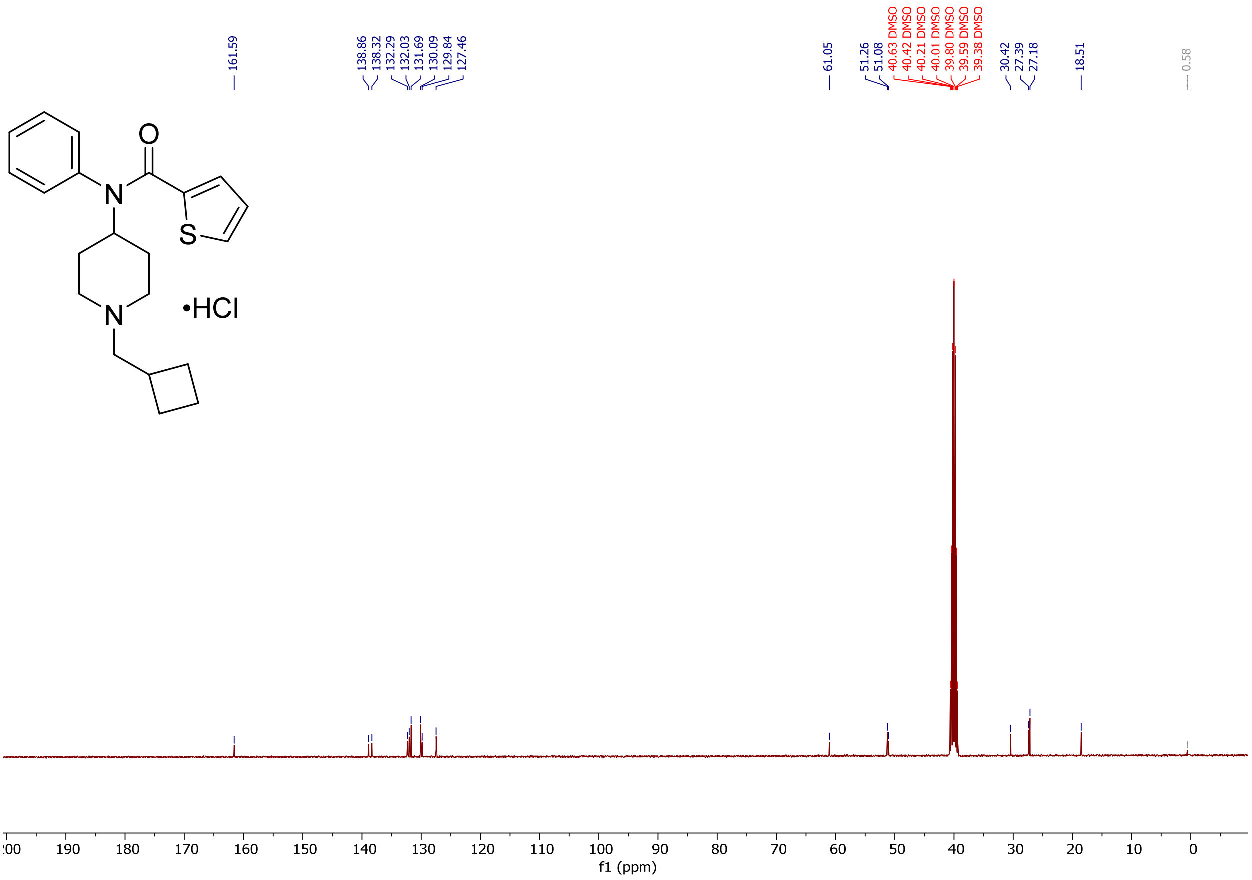

^{13}C NMR (100 MHz, DMSO-d_6) spectrum for compound **15**.

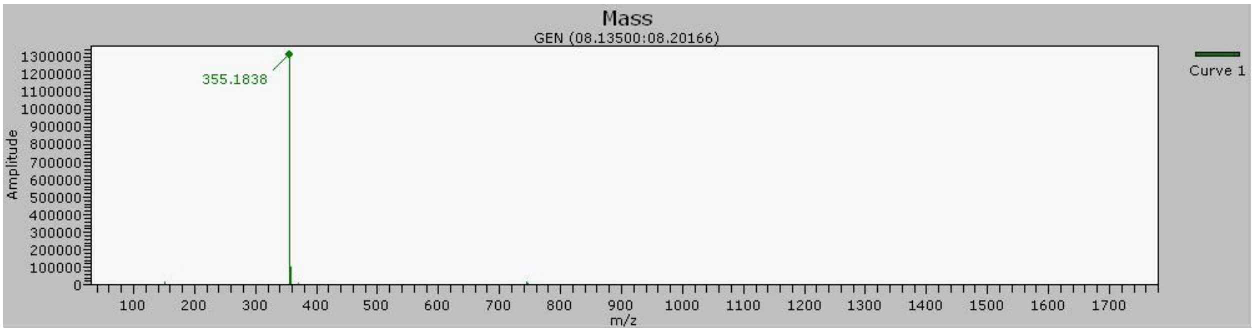

HRMS spectrum for compound **15**.

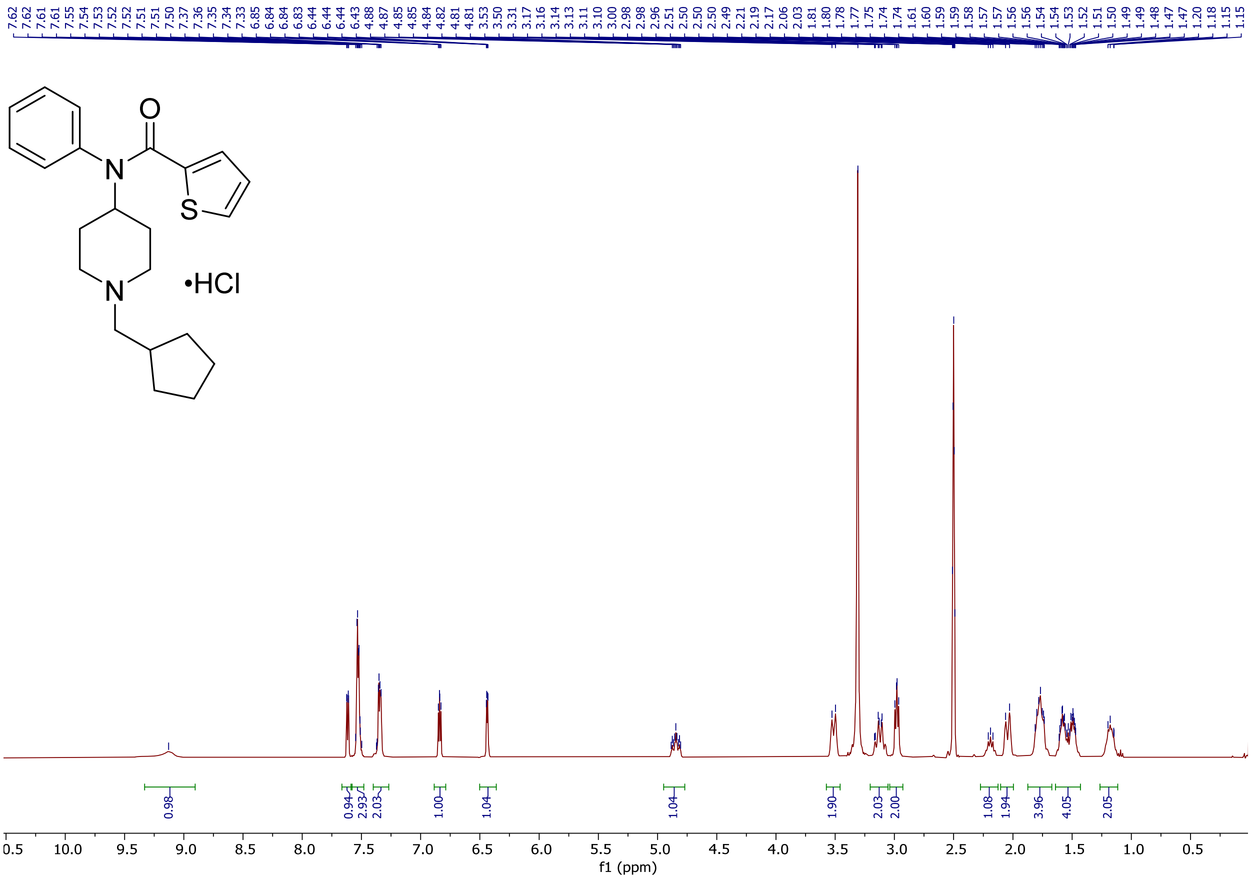

^{1}H NMR (400 MHz, DMSO-d_6) spectrum for compound **16**.

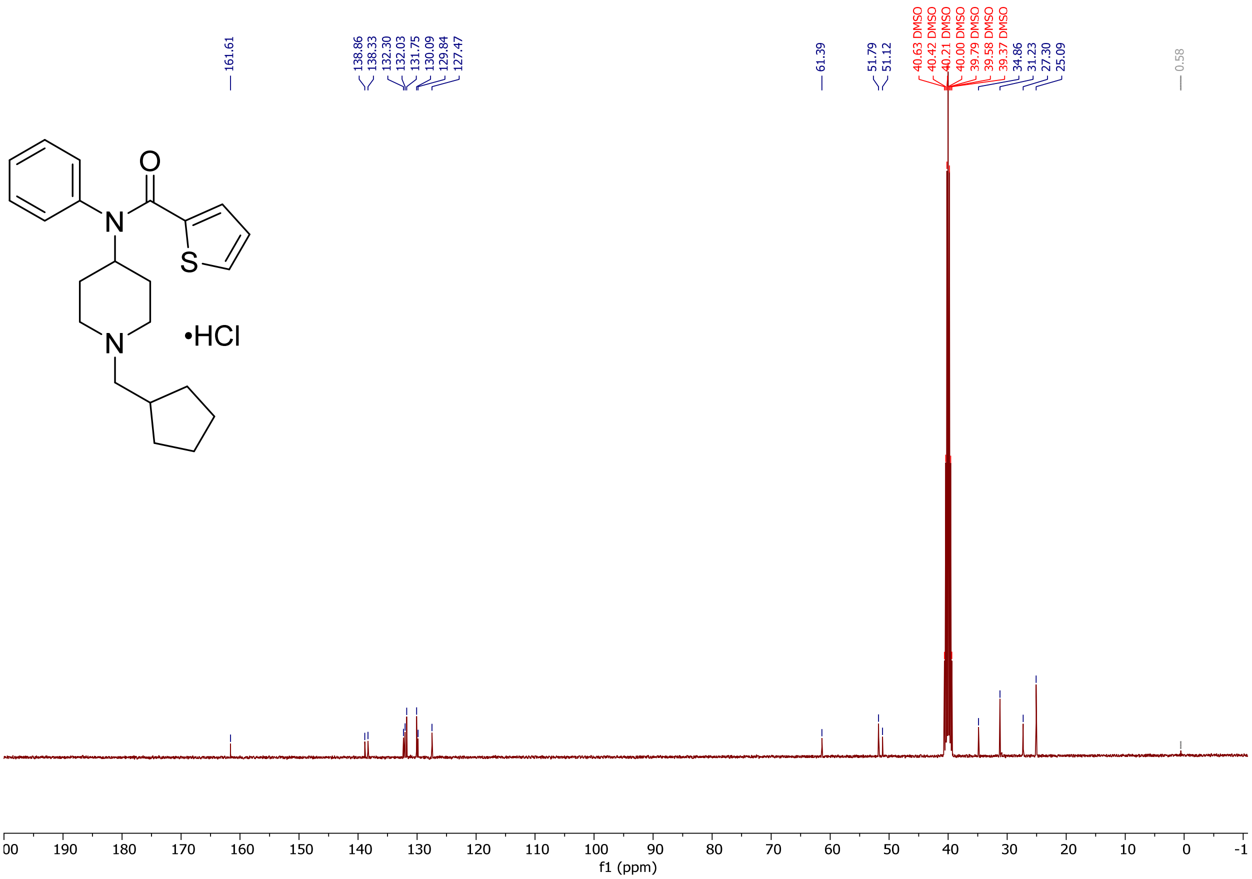

^{13}C NMR (100 MHz, DMSO-d_6) spectrum for compound **16**.

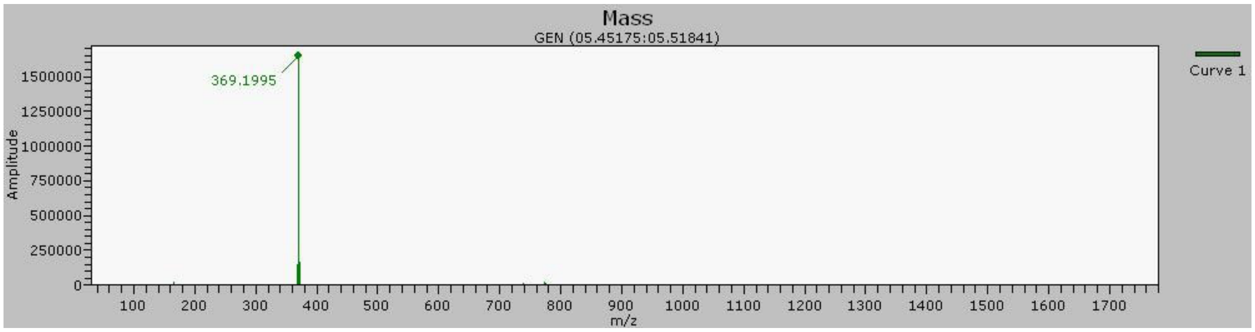

HRMS spectrum for compound **16**.

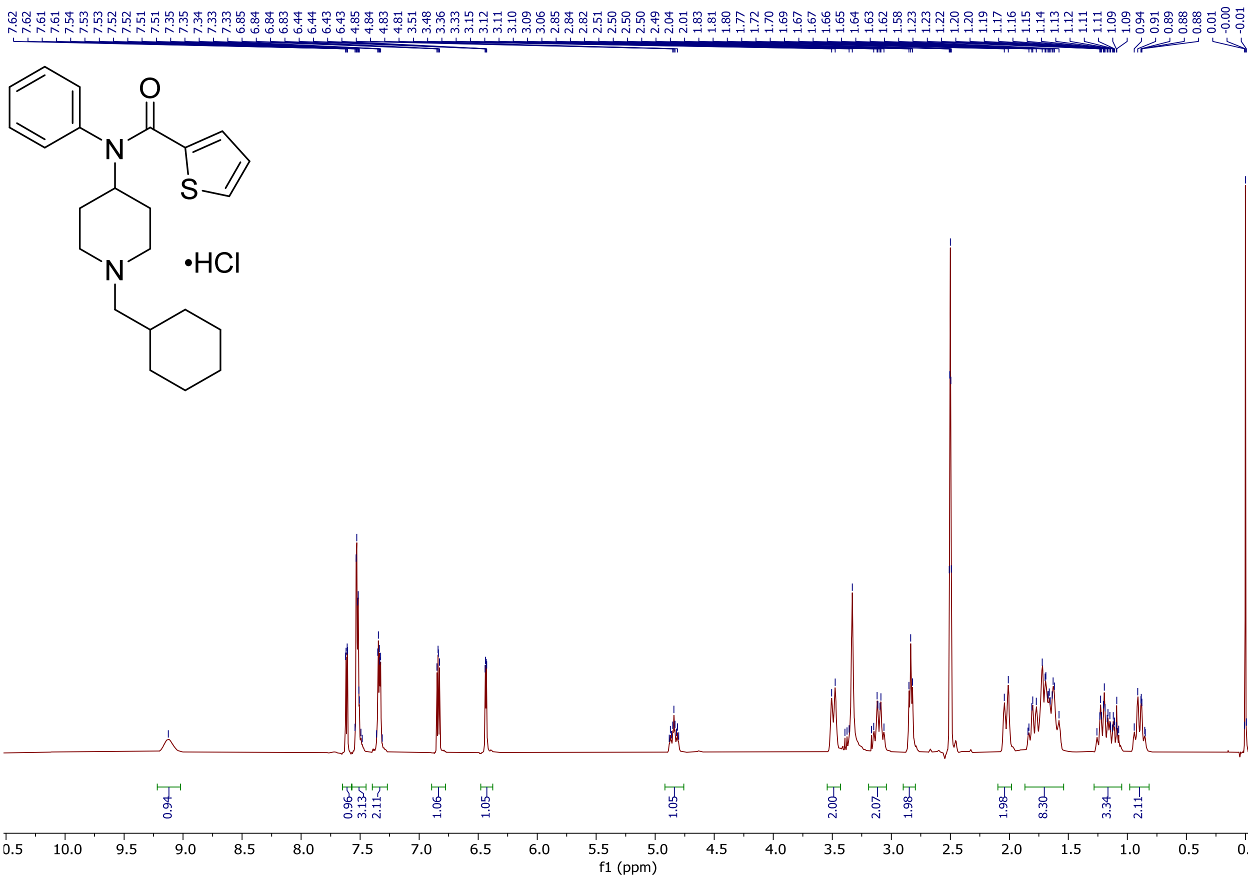

^{1}H NMR (400 MHz, DMSO-d_6) spectrum for compound **17**.

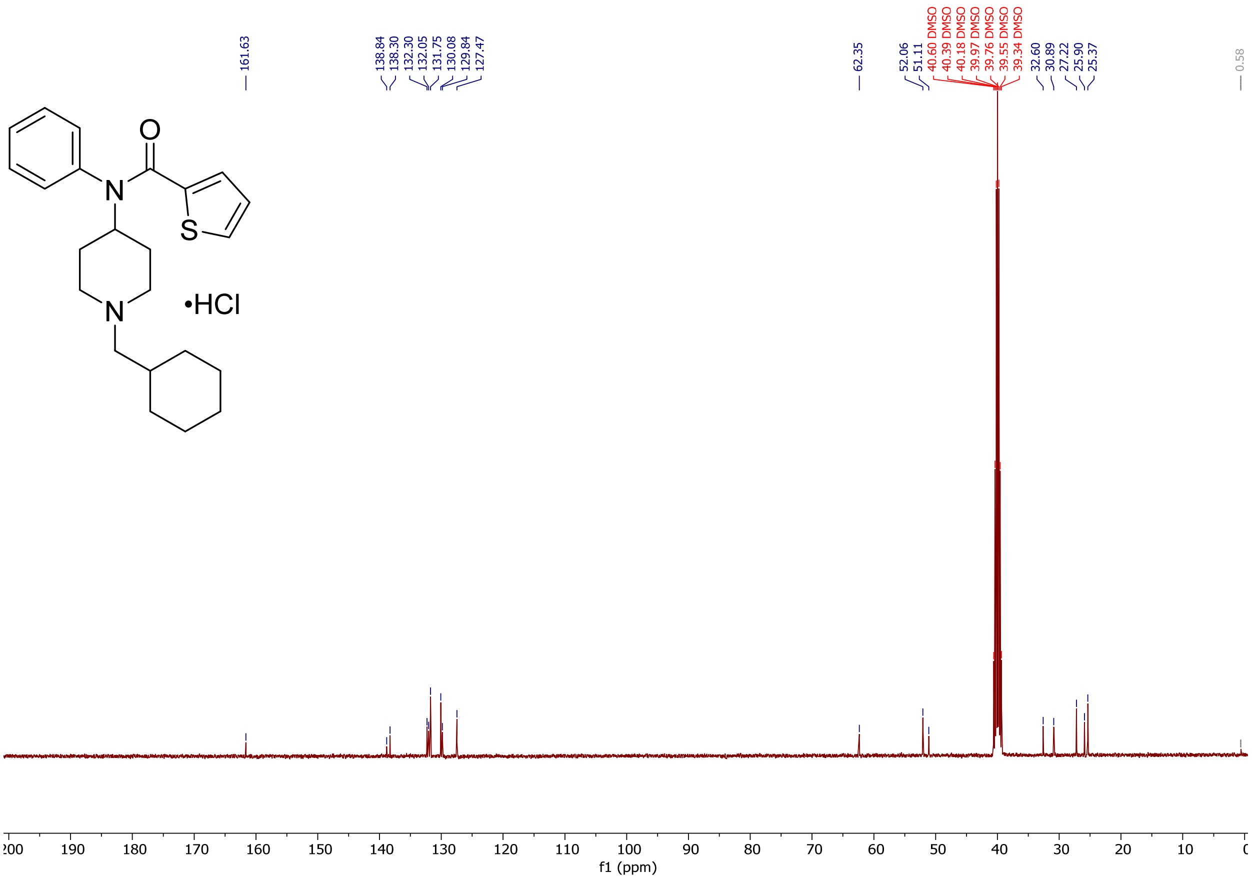

^{13}C NMR (100 MHz, DMSO-d_6) spectrum for compound **17**.

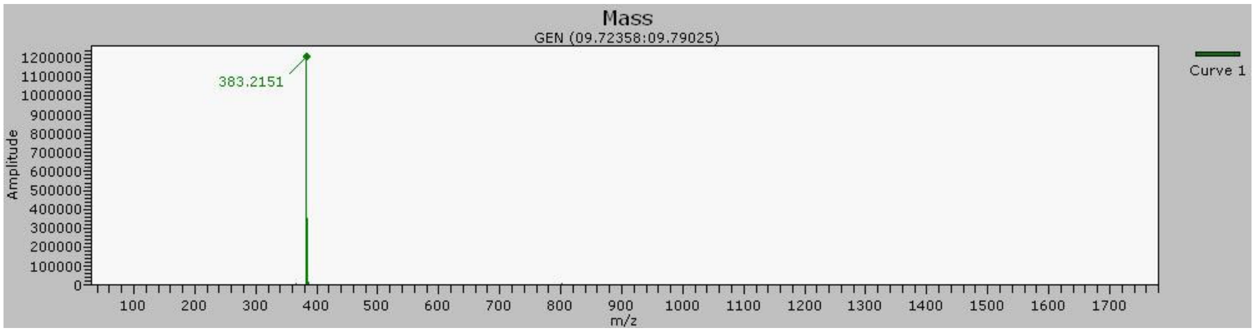

HRMS spectrum for compound **17**.

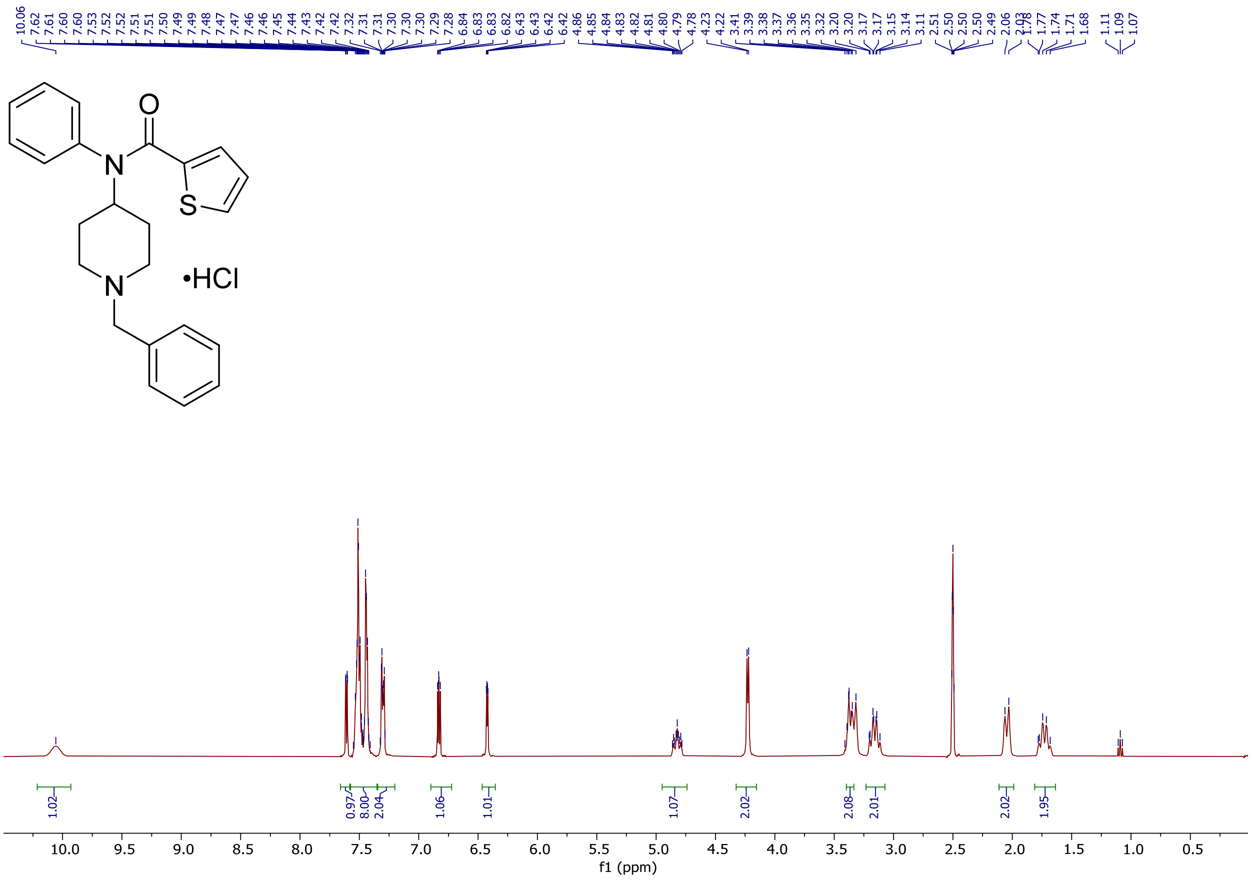

^{1}H NMR (400 MHz, DMSO-d_6) spectrum for compound **18**.

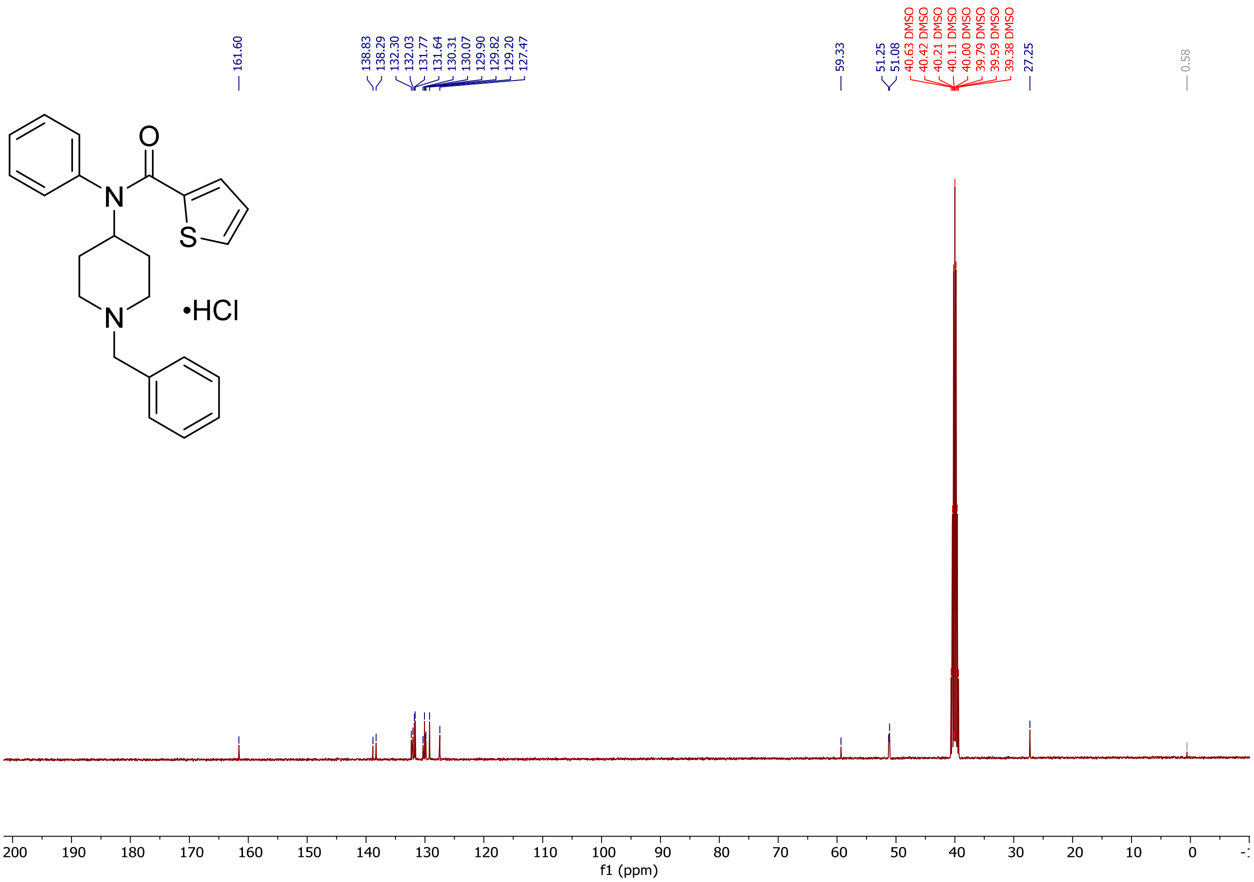

^{13}C NMR (100 MHz, DMSO-d_6) spectrum for compound **18**.

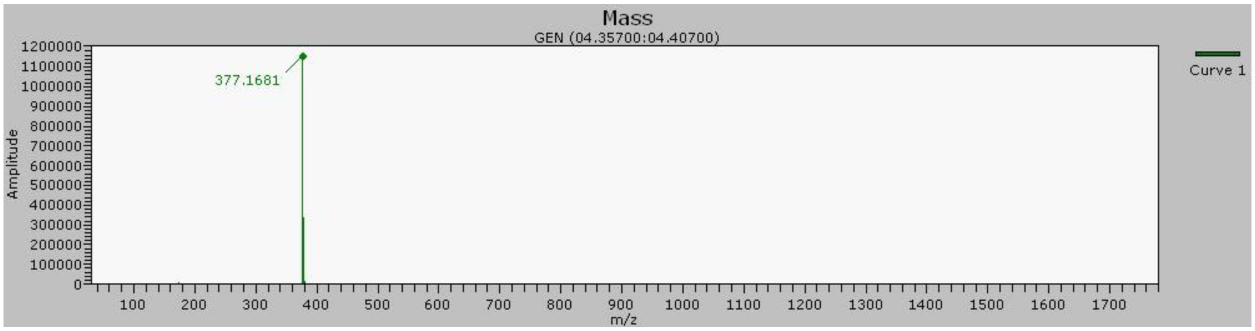

HRMS spectrum for compound **18**.

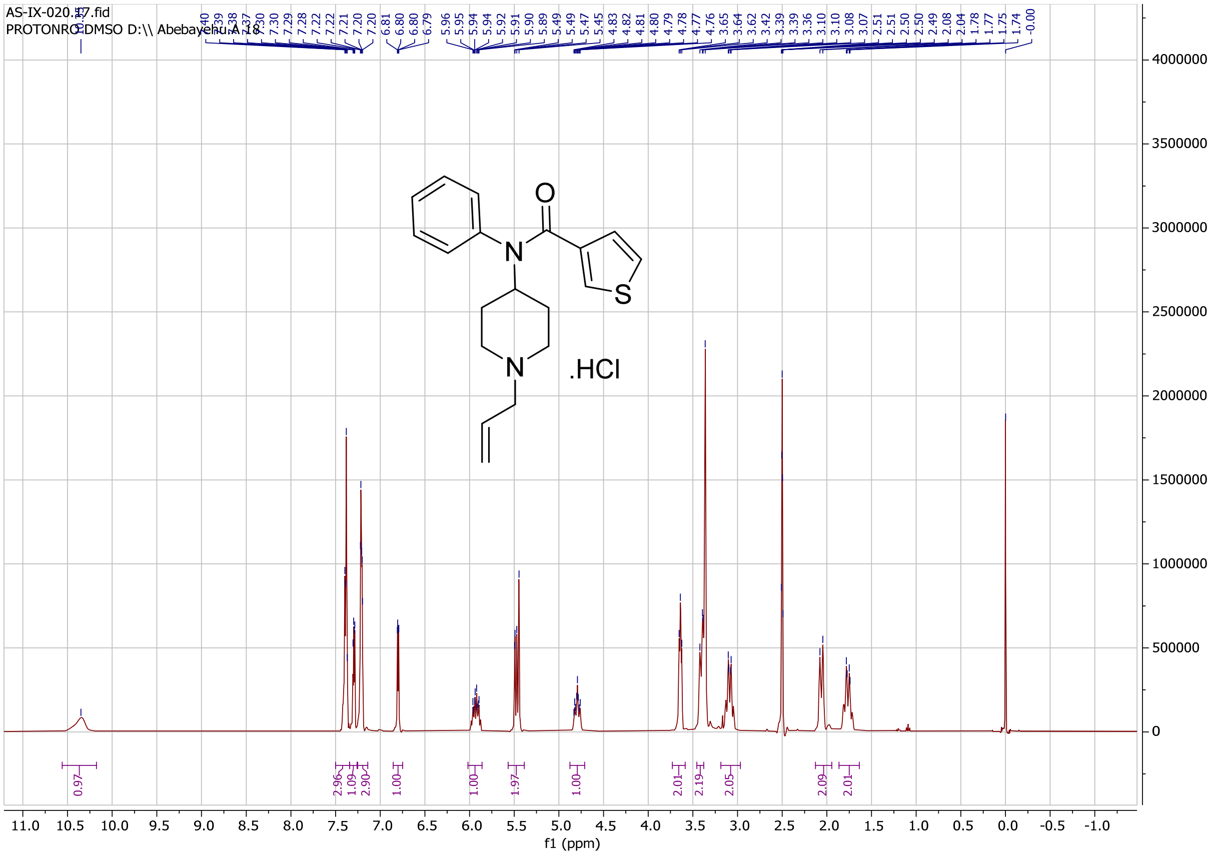

^{1}H NMR (400 Hz, DMSO-d_6) spectrum for compound **19.**

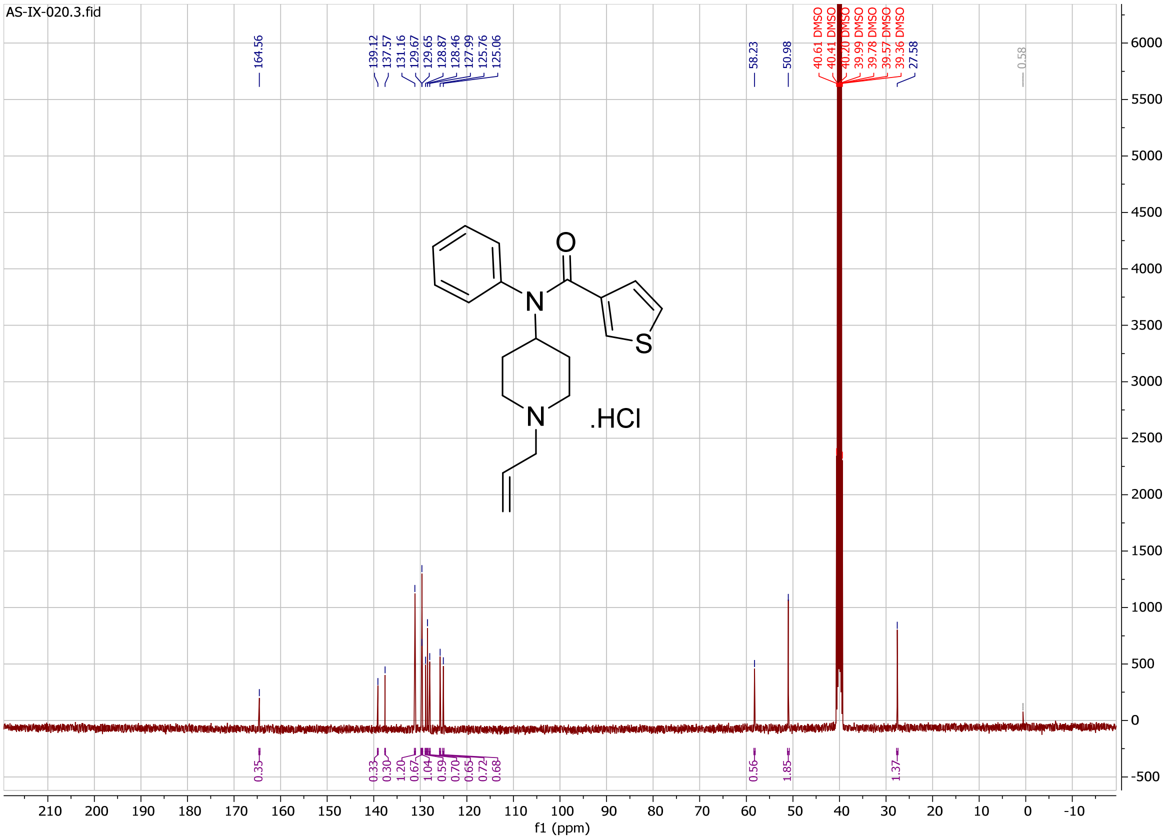

^{13}C NMR (100 Hz, DMSO-d_6) spectrum for compound **19**.

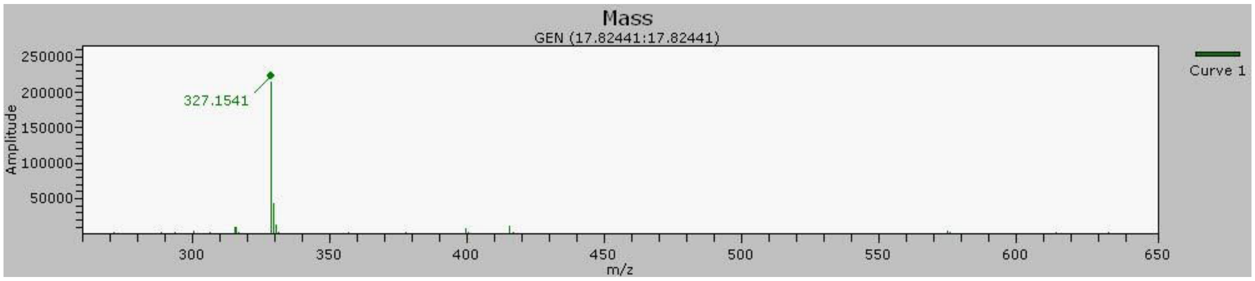

HRMS spectrum for compound **19**.

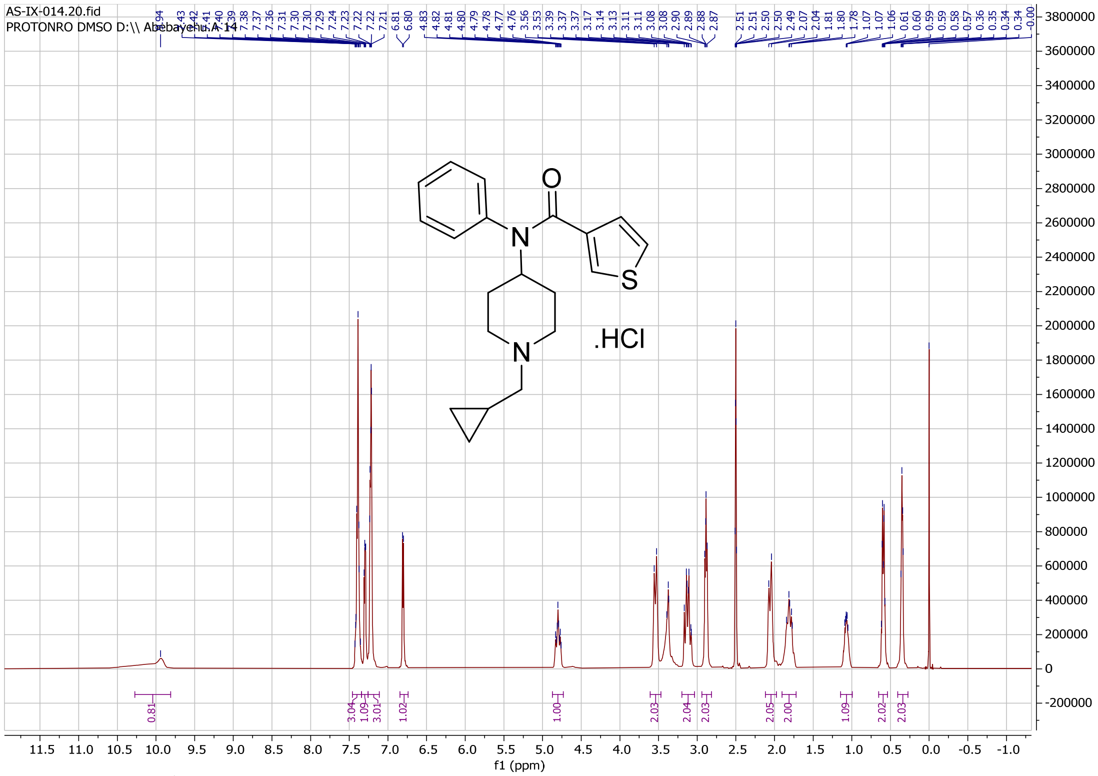

^{1}H NMR (400 MHz, DMSO-d_6) spectrum for compound **20**.

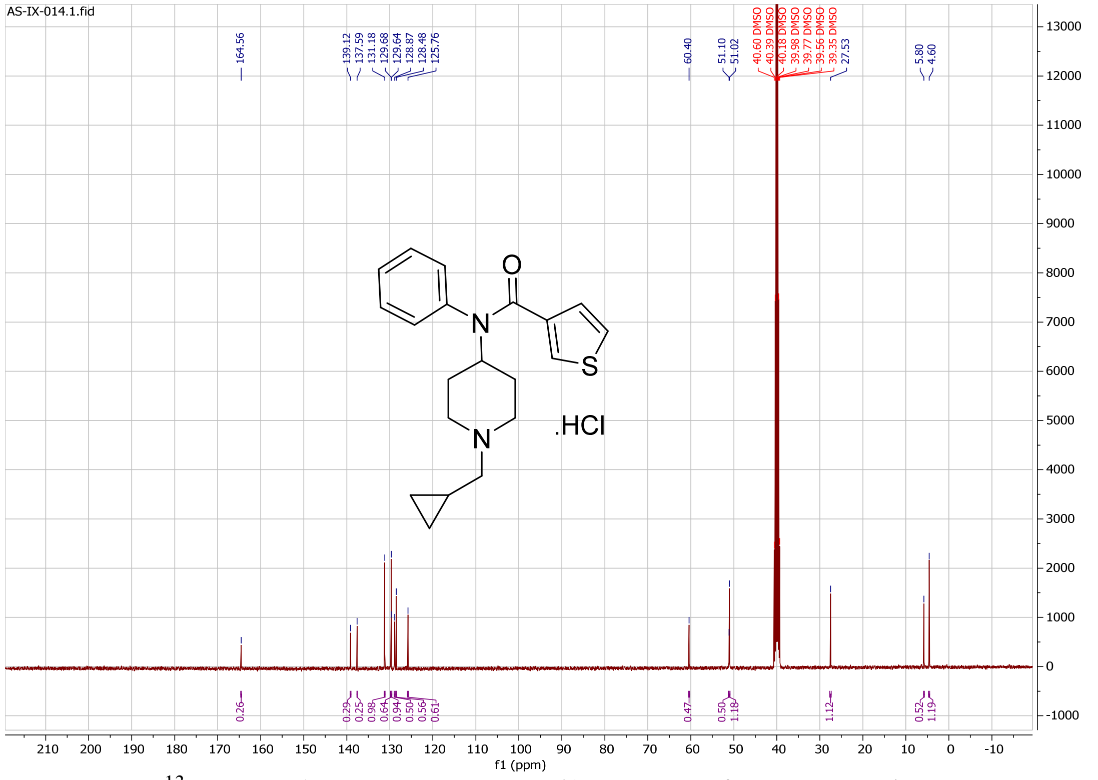

^{13}C NMR (100 MHz, DMSO-d_6) spectrum for compound **20**.

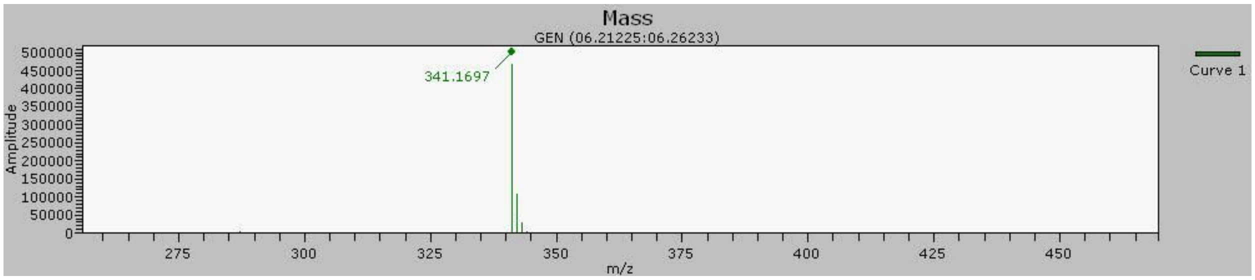

HRMS spectrum for compound **20**.

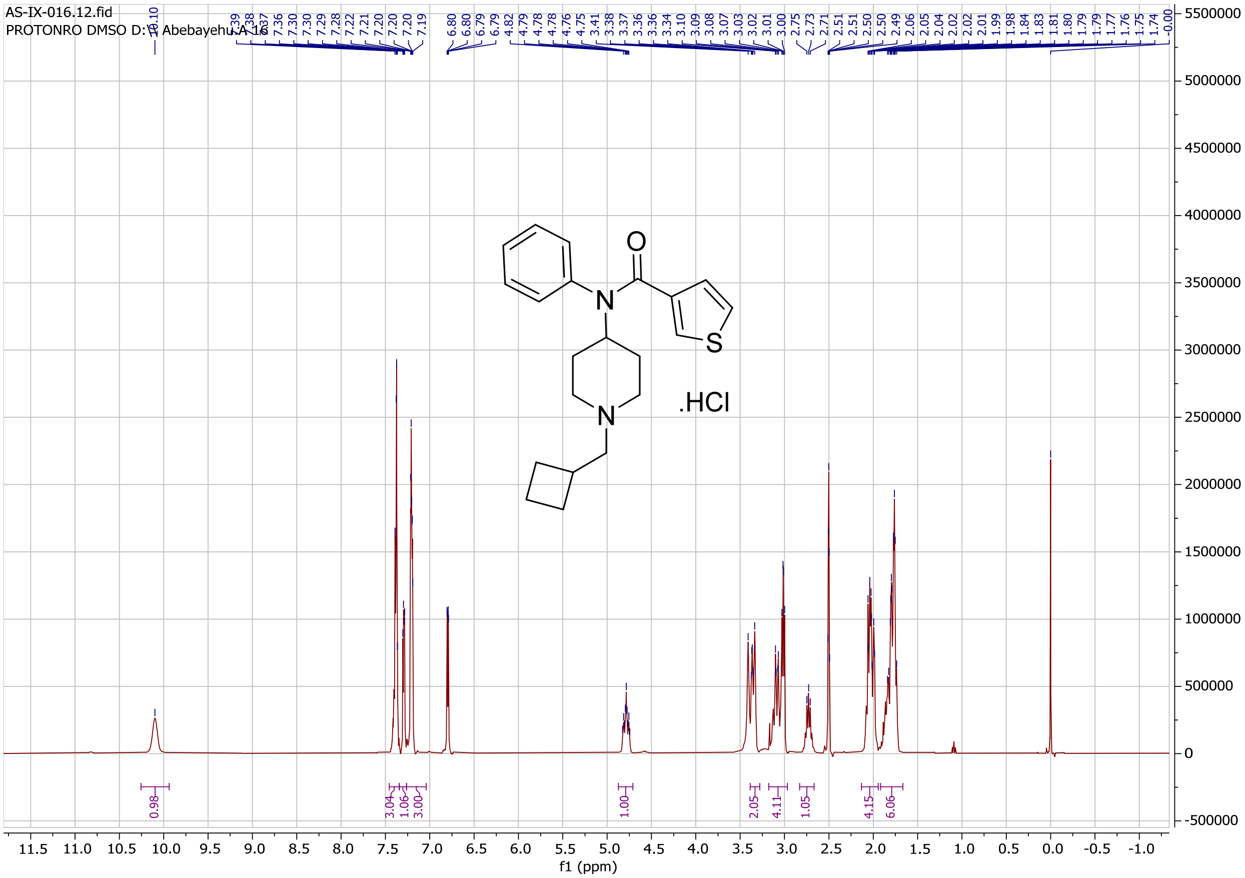

^{1}H NMR (400 MHz, DMSO-d_6) spectrum for compound **21**.

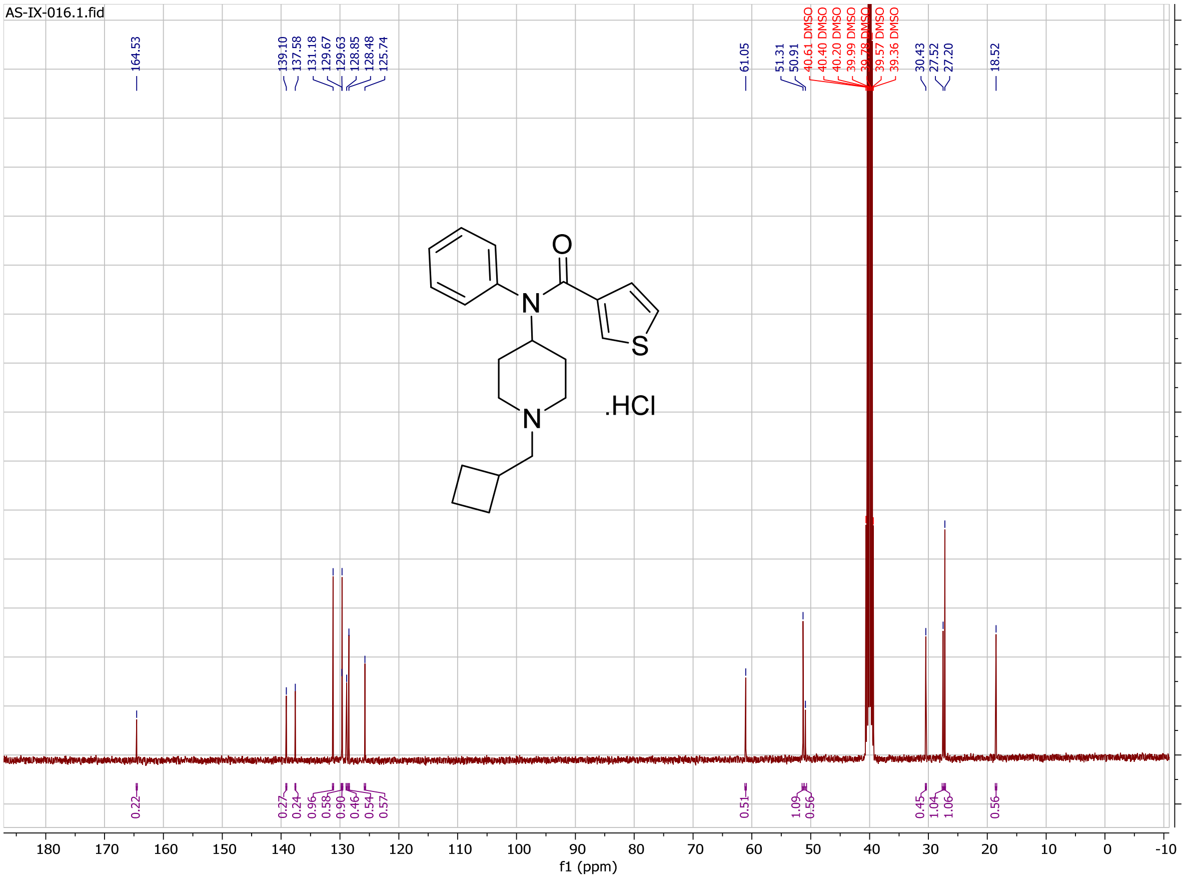

^{13}C NMR (100 MHz, DMSO-d_6) spectrum for compound **21**.

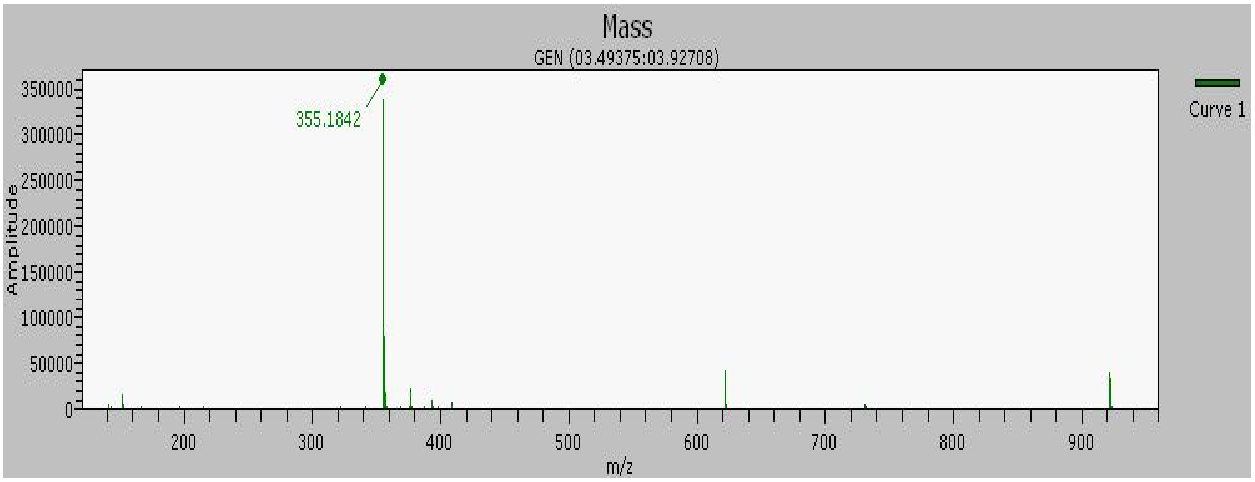

HRMS spectrum for compound **21**.

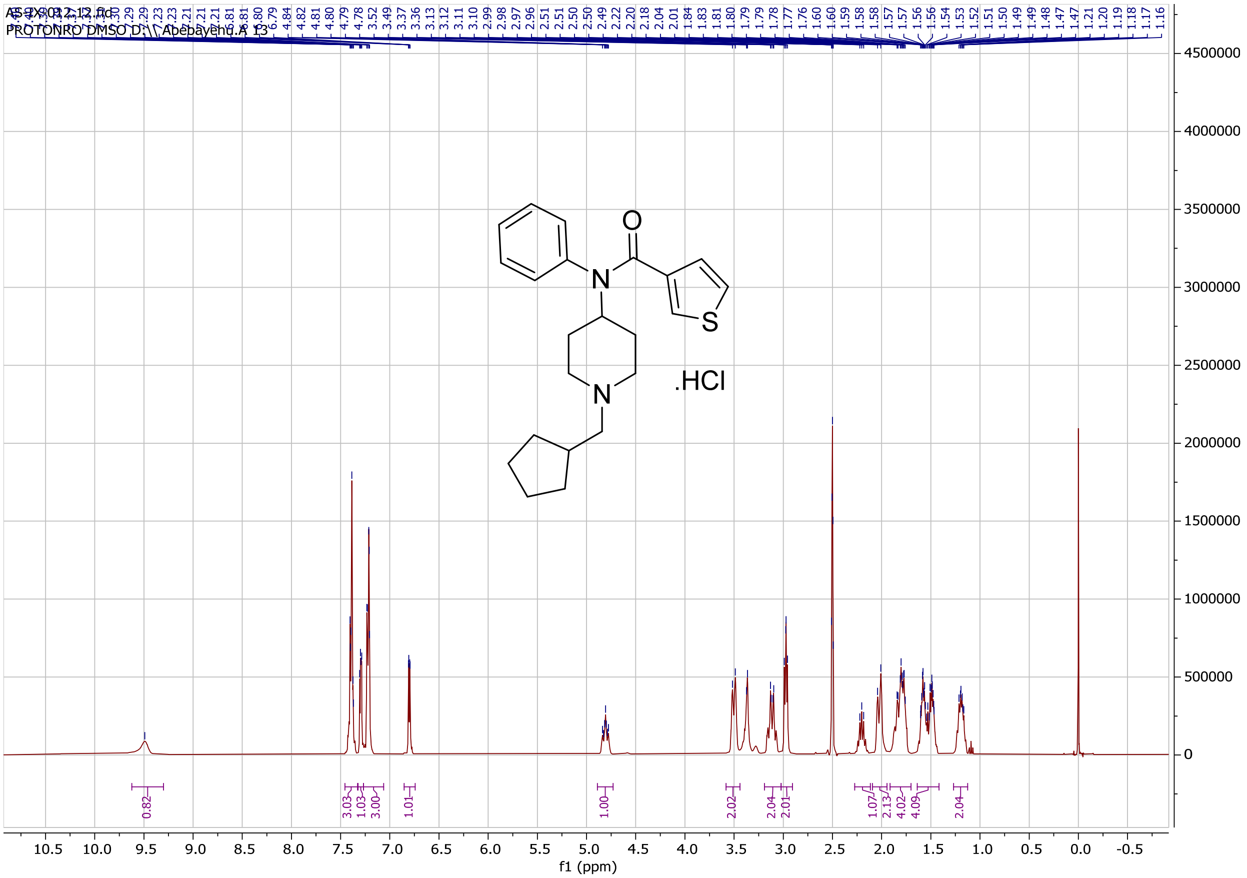

^{1}H NMR (400 MHz, DMSO-d_6) spectrum for compound **22**.

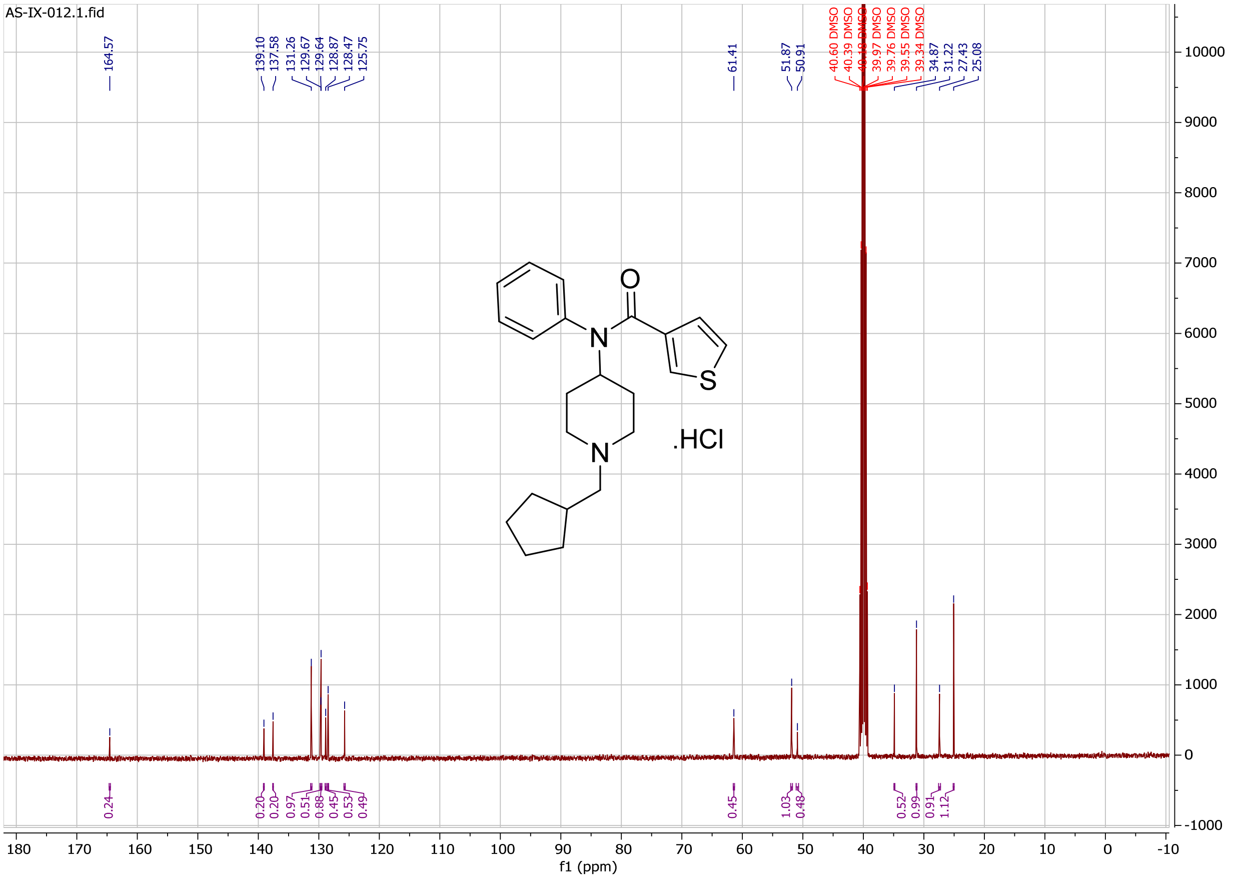

^{13}C NMR (100 MHz, DMSO-d_6) spectrum for compound **22**.

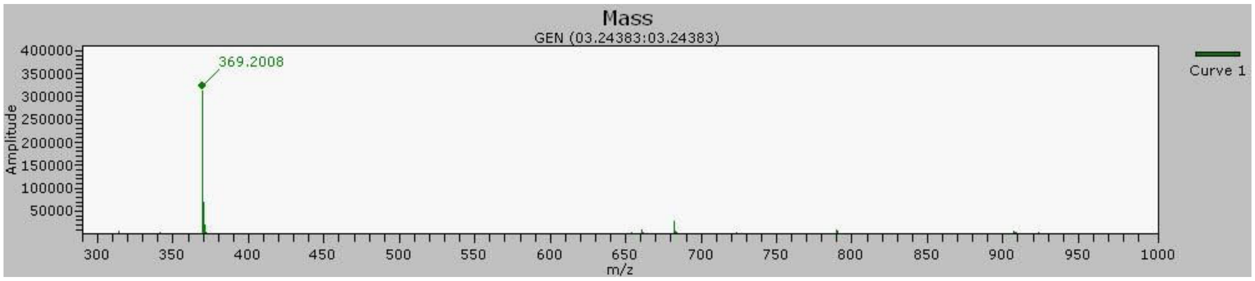

HRMS spectrum for compound **22**.

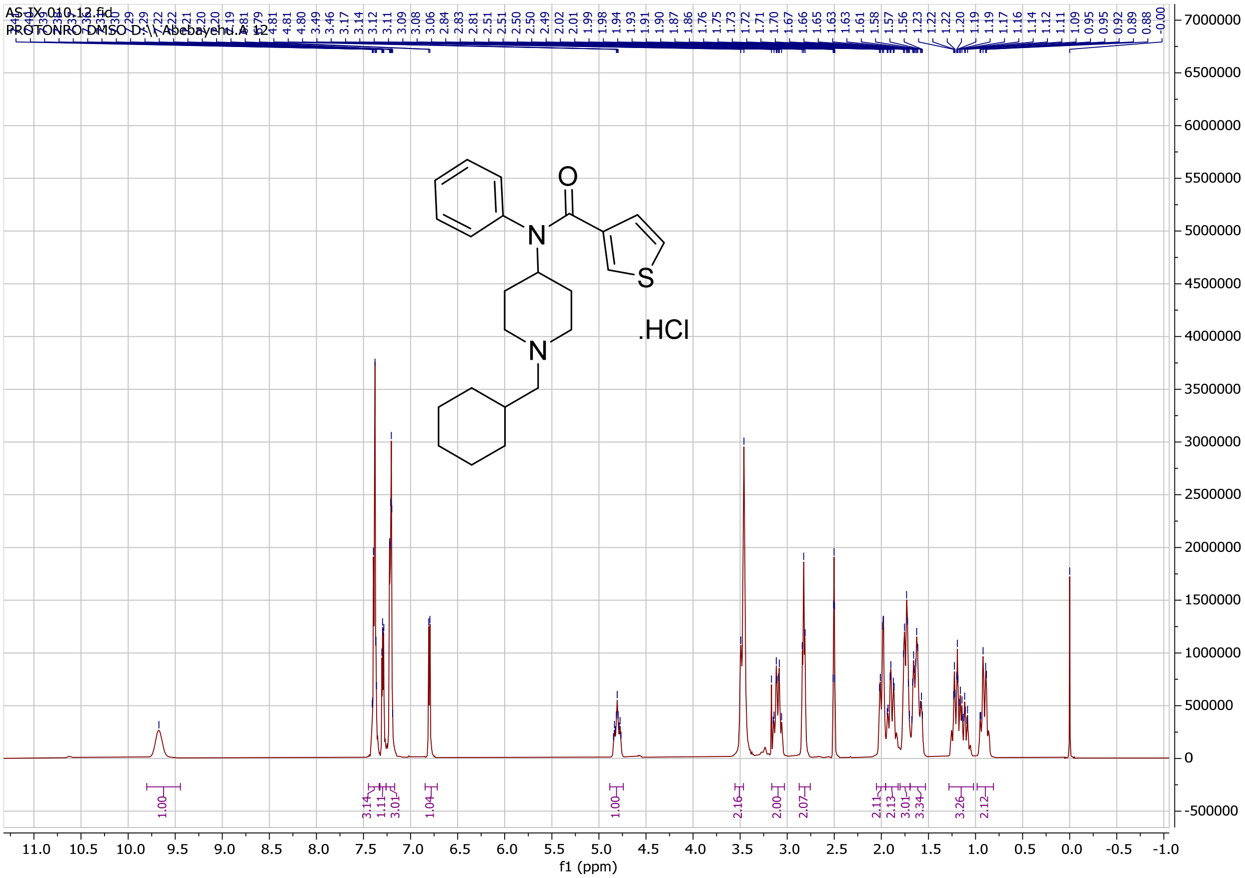

^{1}H NMR (400 MHz, DMSO-d_6) spectrum for compound **23**.

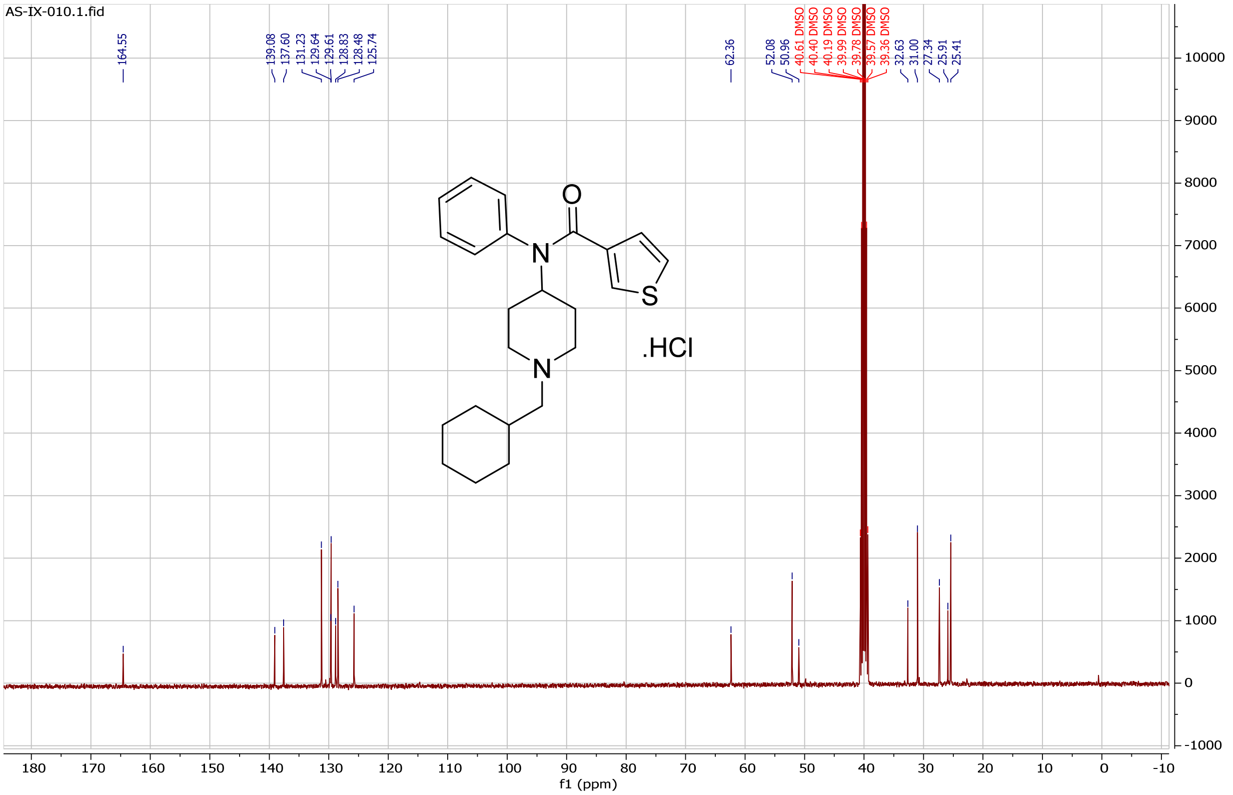

^{13}C NMR (100 MHz, DMSO-d_6) spectrum for compound **23**.

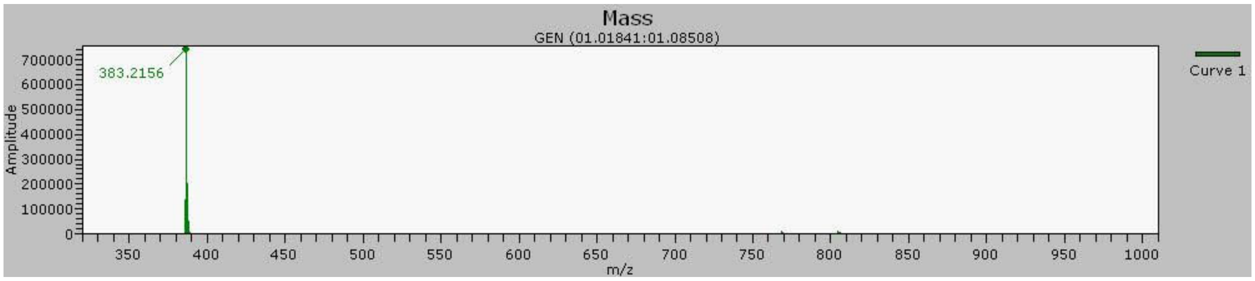

HRMS spectrum for compound **23**.

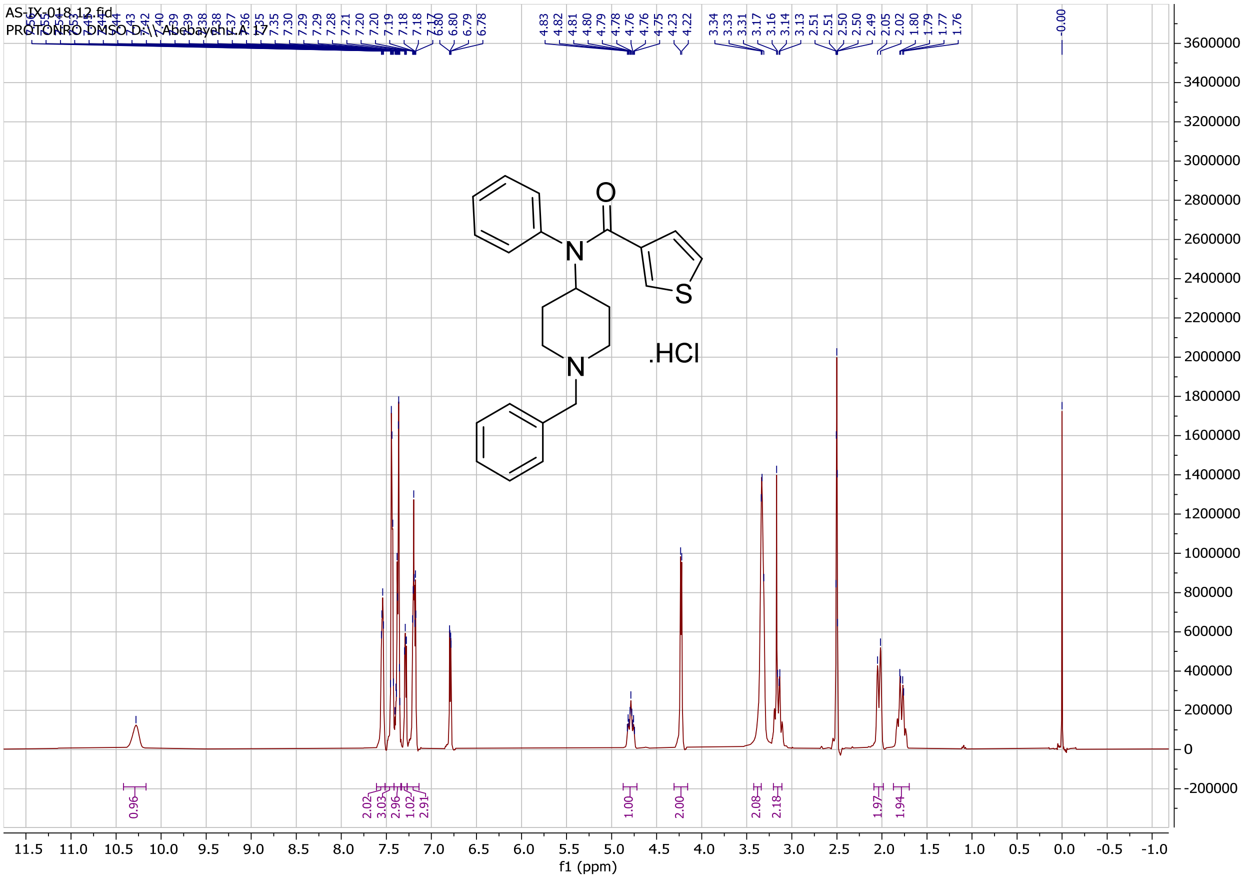

^{1}H NMR (400 MHz, DMSO-d_6) spectrum for compound **24**.

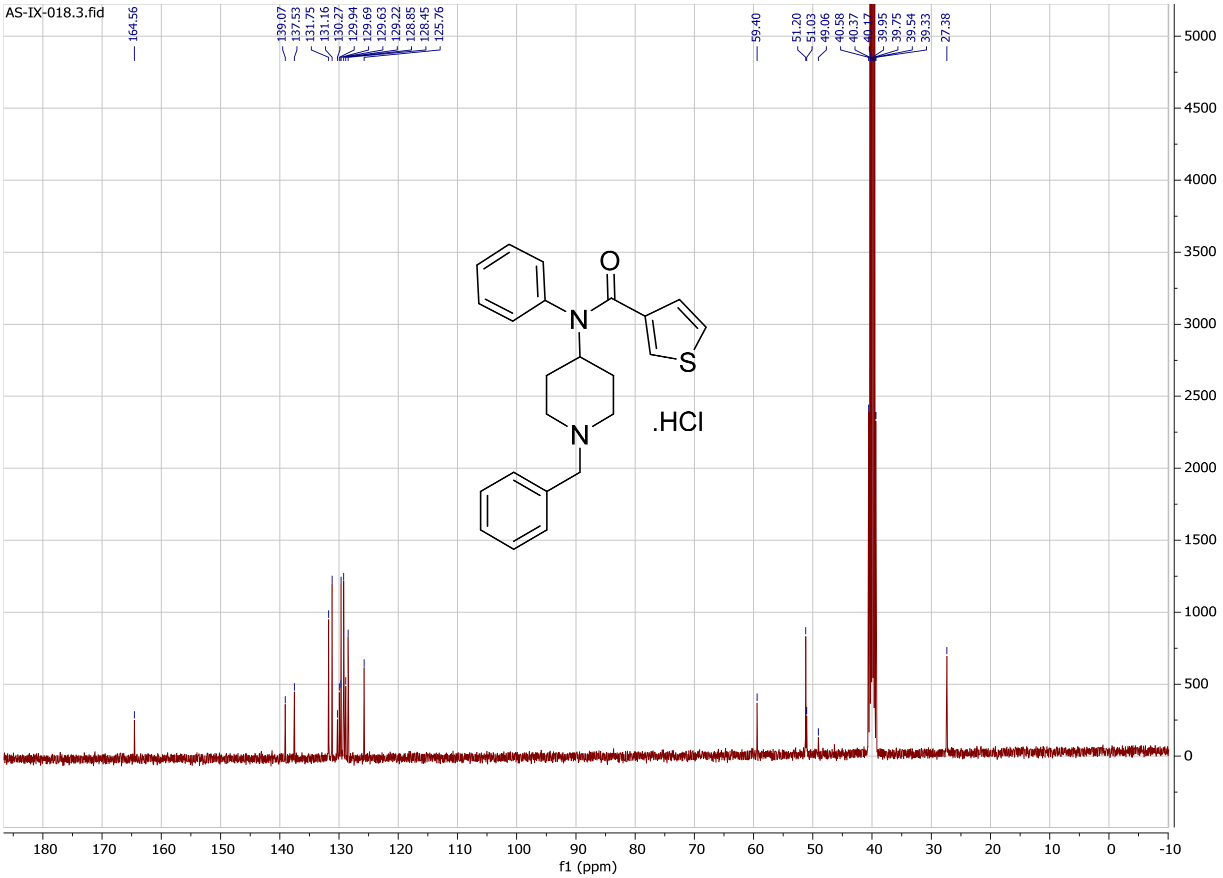

^{13}C NMR (100 MHz, DMSO-d_6) spectrum for compound **24**.

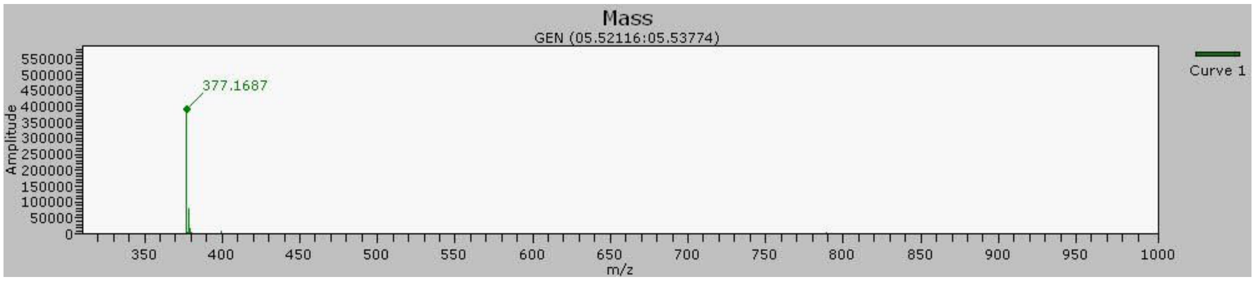

HRMS spectrum for compound **24**.

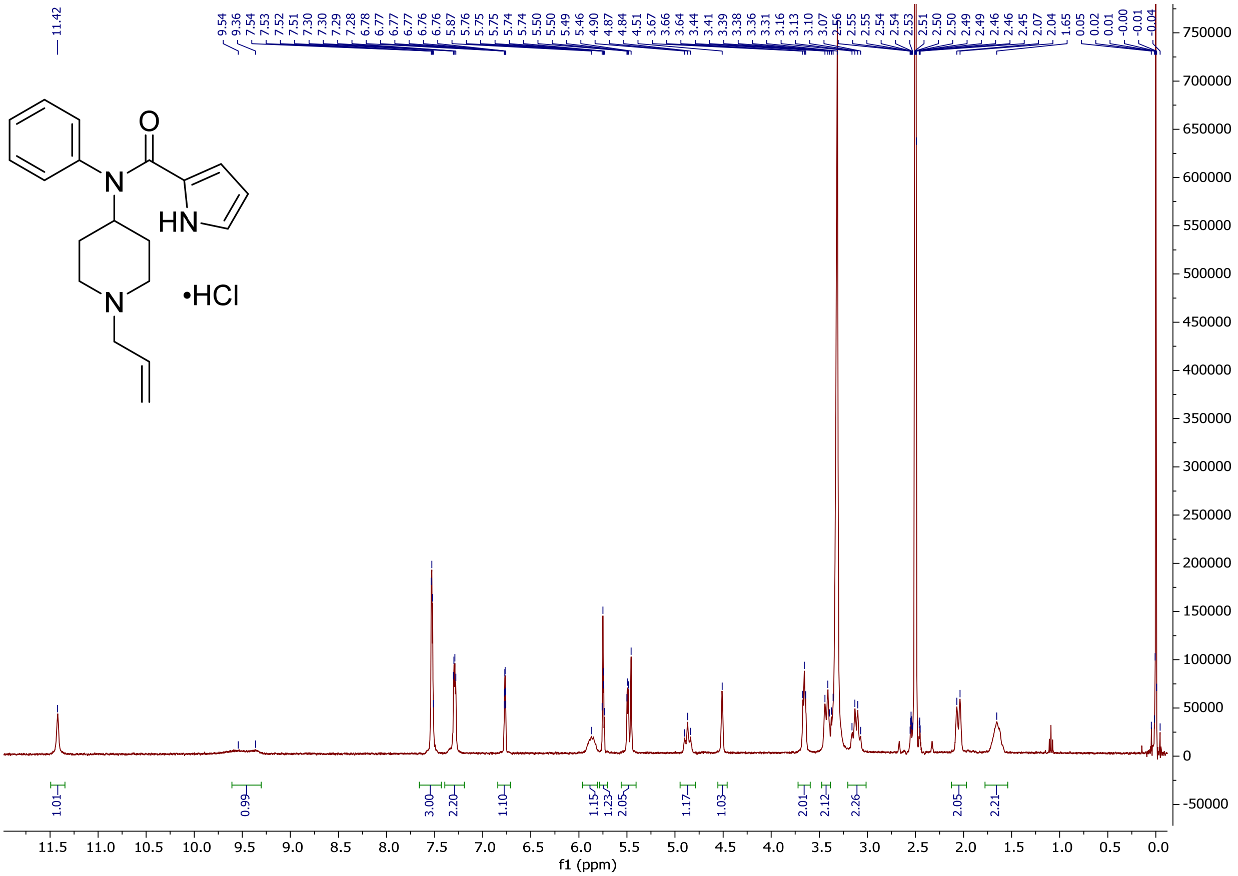

^{1}H NMR (400 MHz, DMSO-d_6) spectrum for compound **25**.

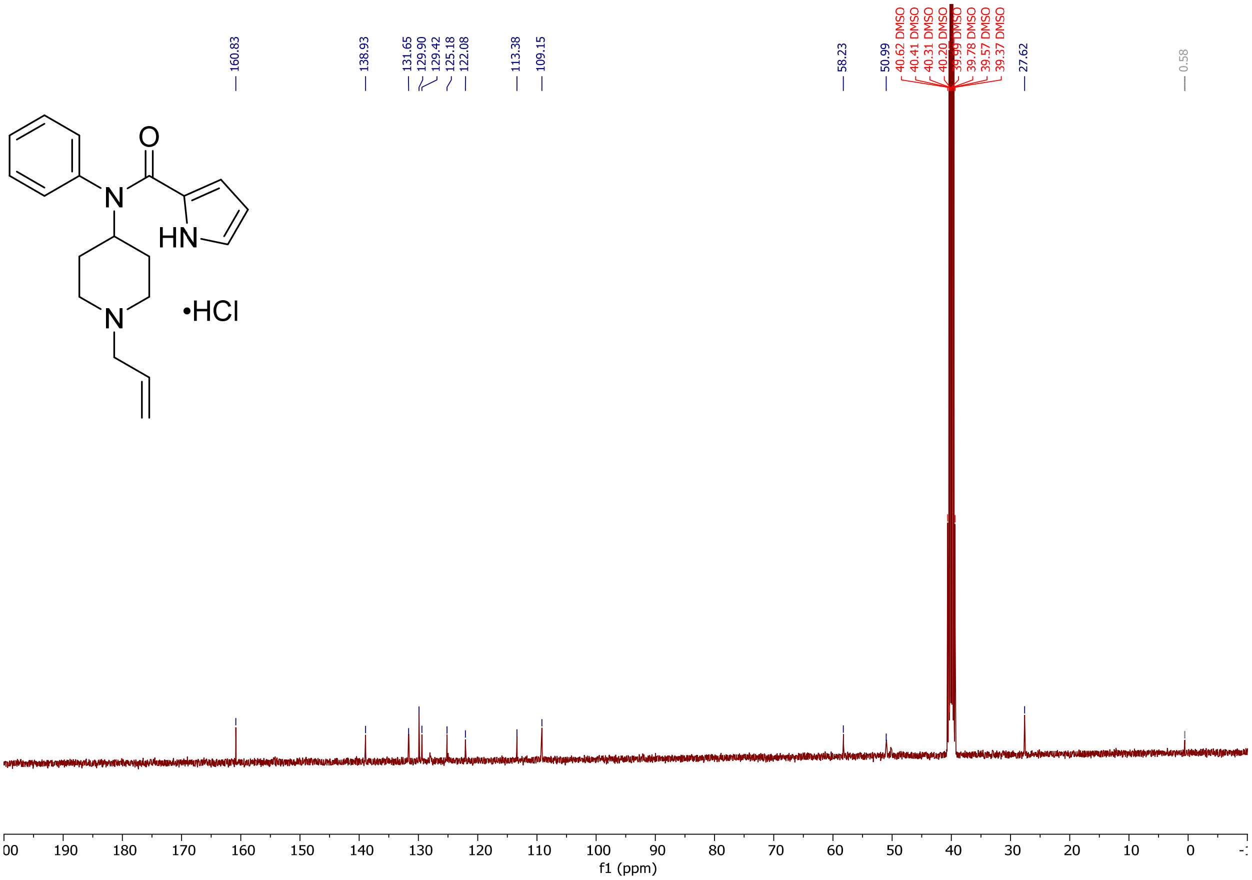

^{13}C NMR (100 MHz, DMSO-d_6) spectrum for compound **25**.

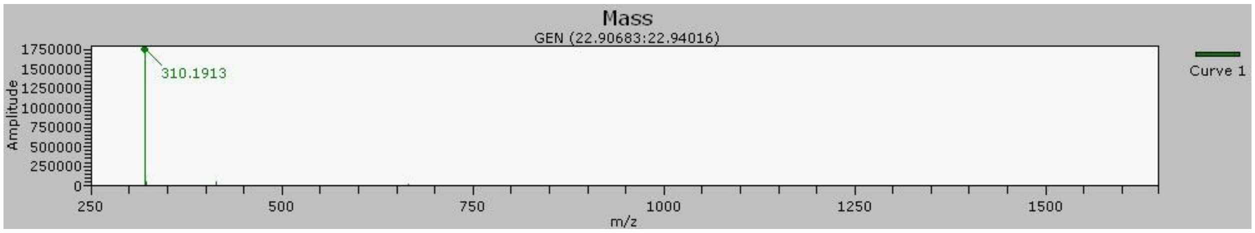

HRMS spectrum for compound **25**.

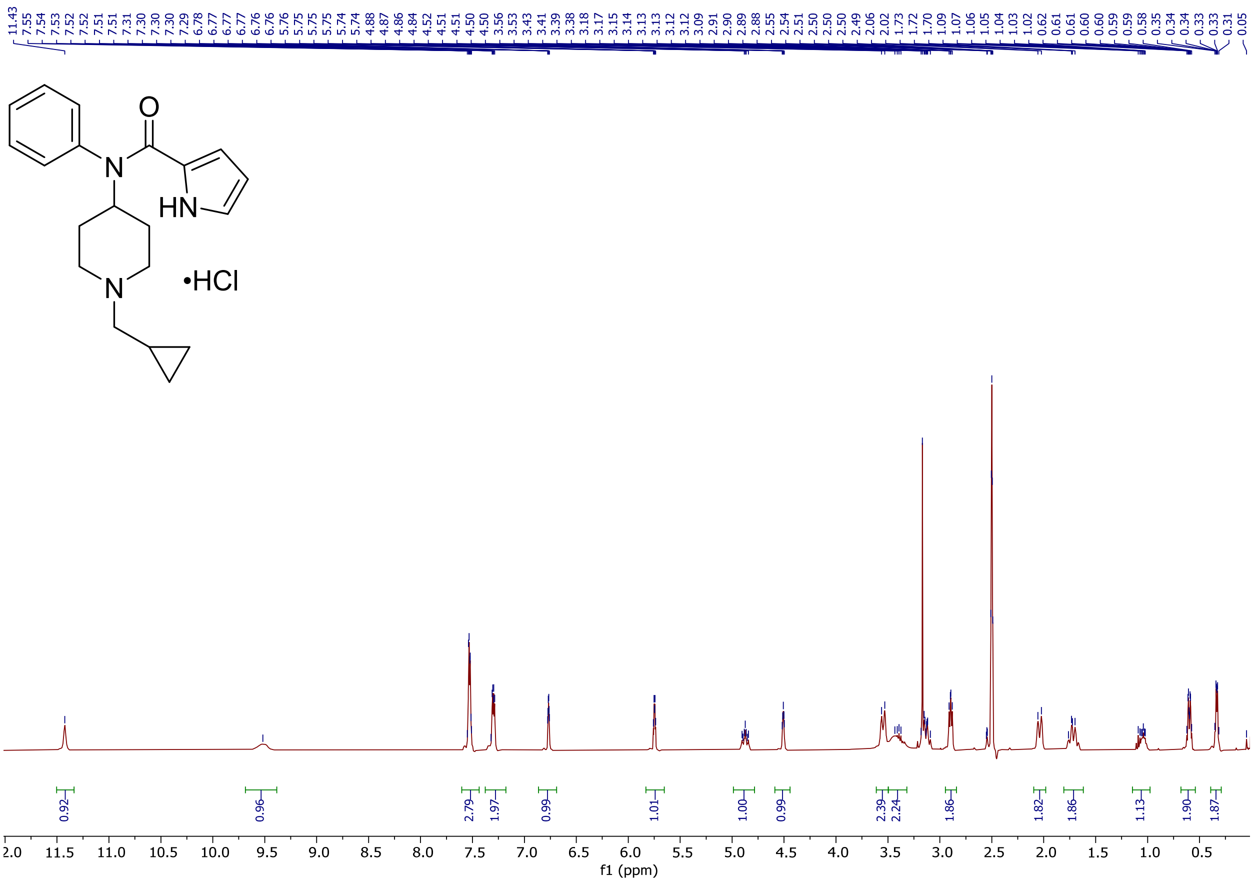

^{1}H NMR (400 MHz, DMSO-d_6) spectrum for compound **26**.

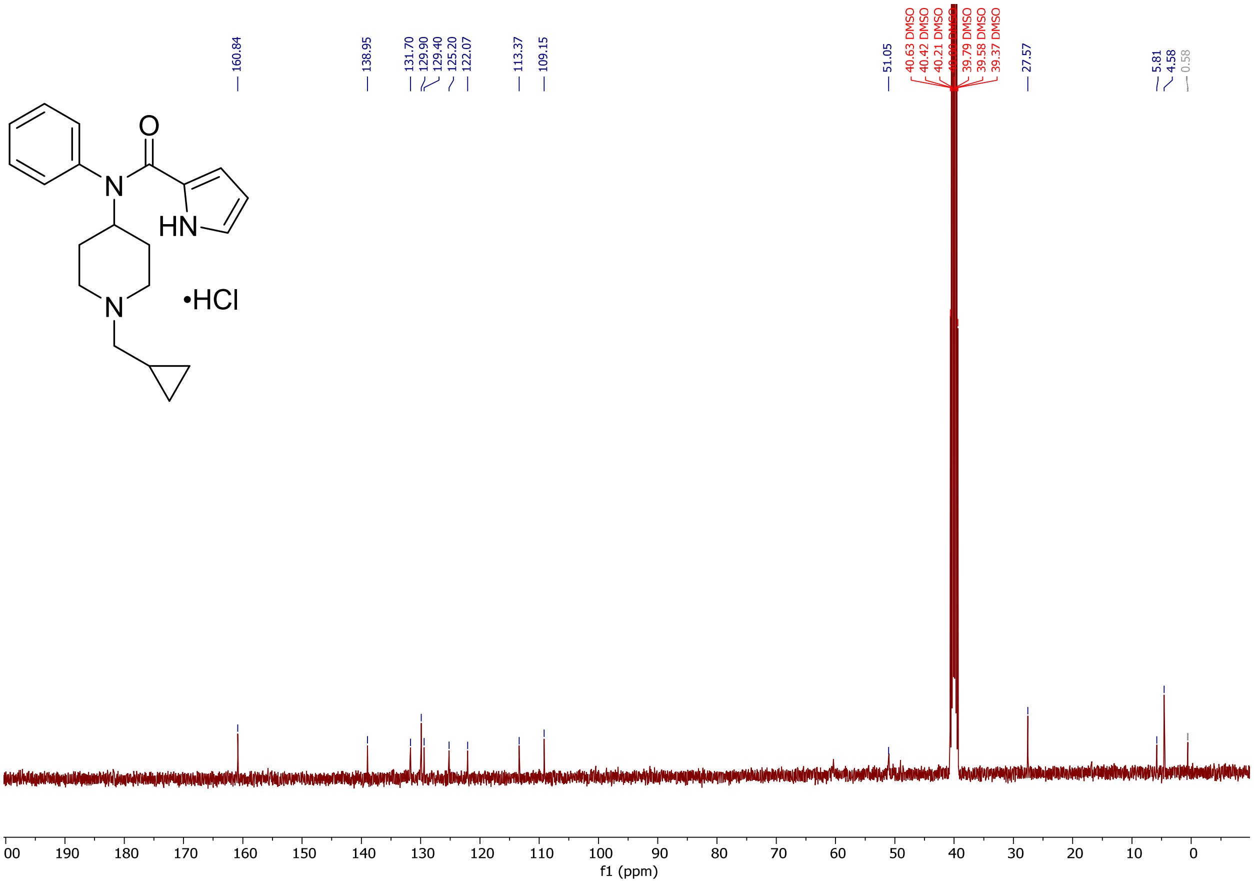

^{13}C NMR (100 MHz, DMSO-*d*6) spectrum for compound **26**.

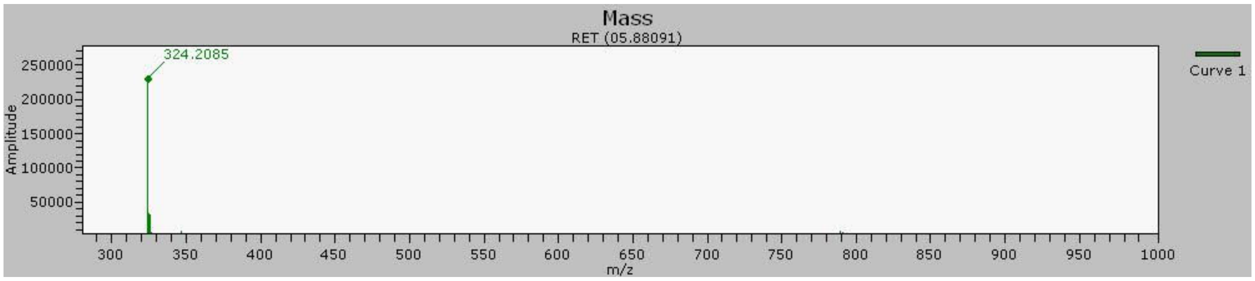

HRMS spectrum for compound **26**.

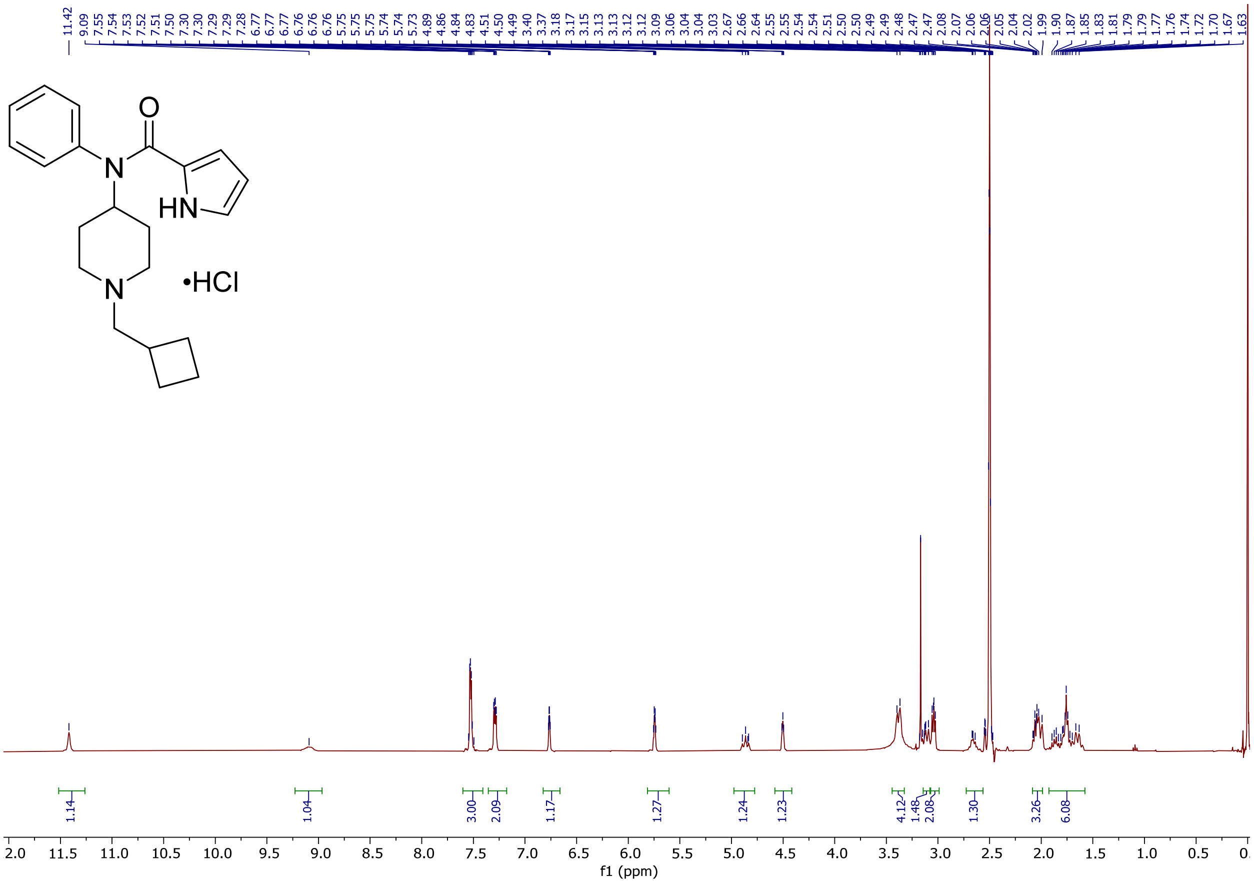

^{1}H NMR (400 MHz, DMSO-d_6) spectrum for compound **27**.

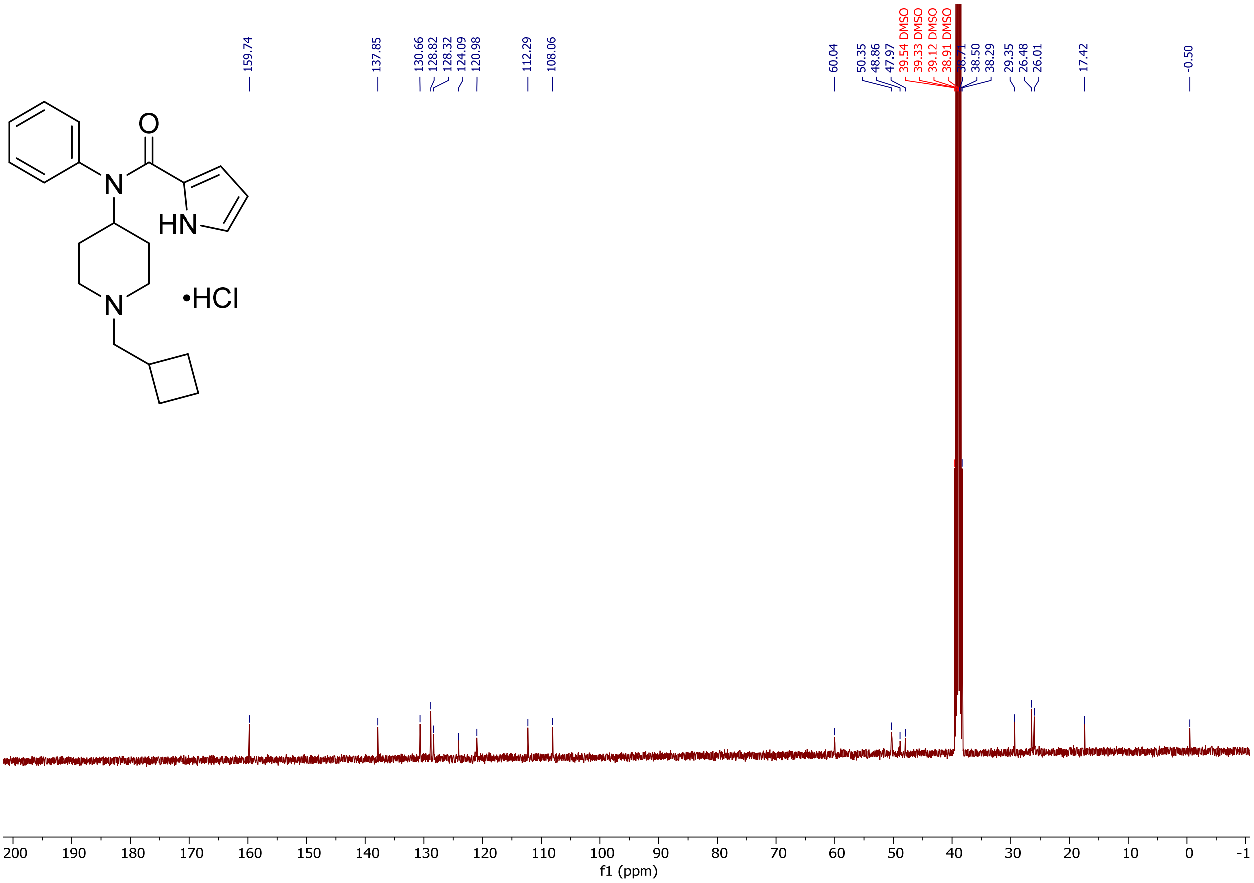

^{13}C NMR (100 MHz, DMSO-d_6) spectrum for compound **27**.

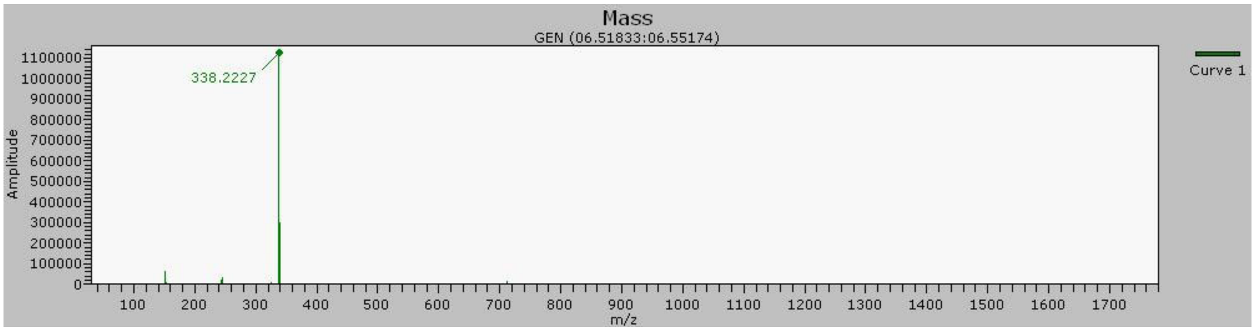

HRMS spectrum for compound **27**.

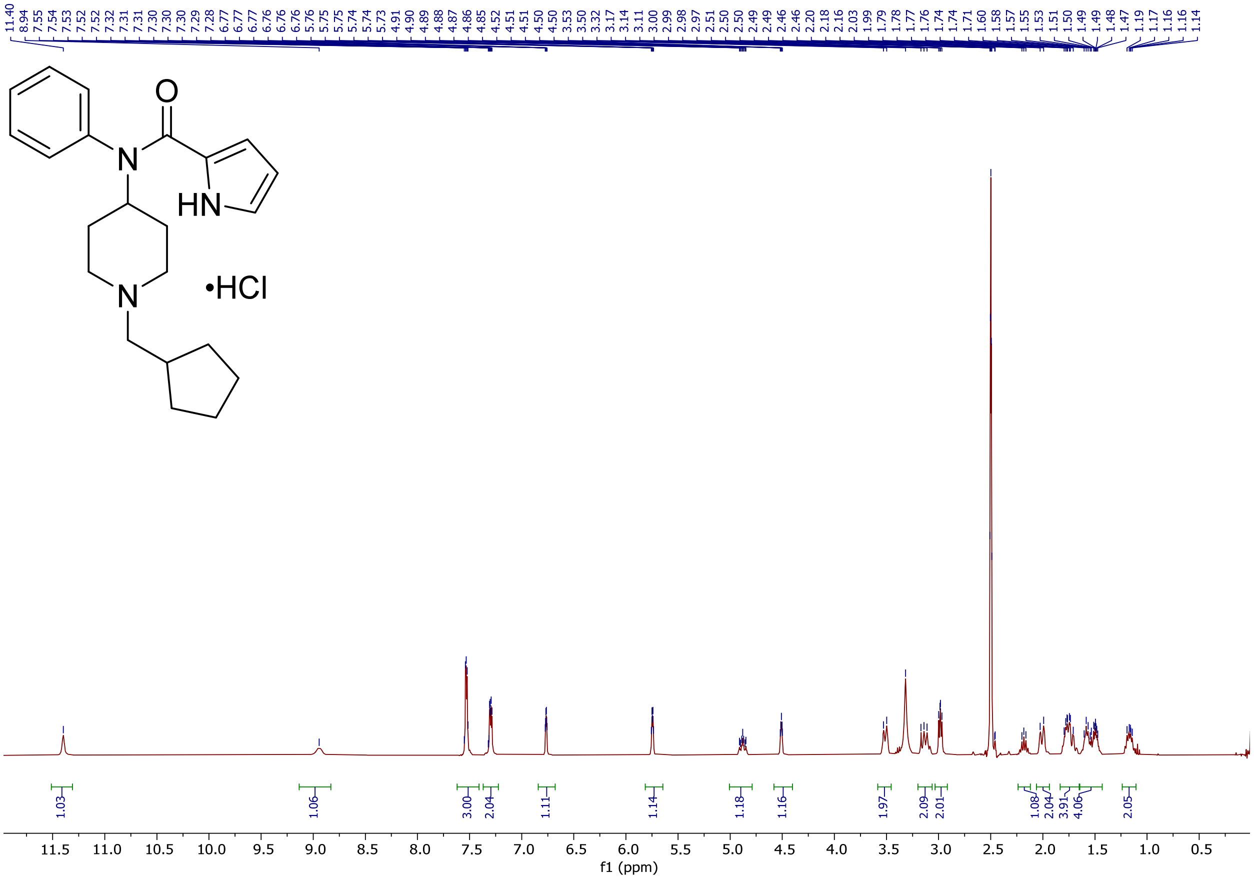

^{1}H NMR (400 MHz, DMSO-d_6) spectrum for compound **28**.

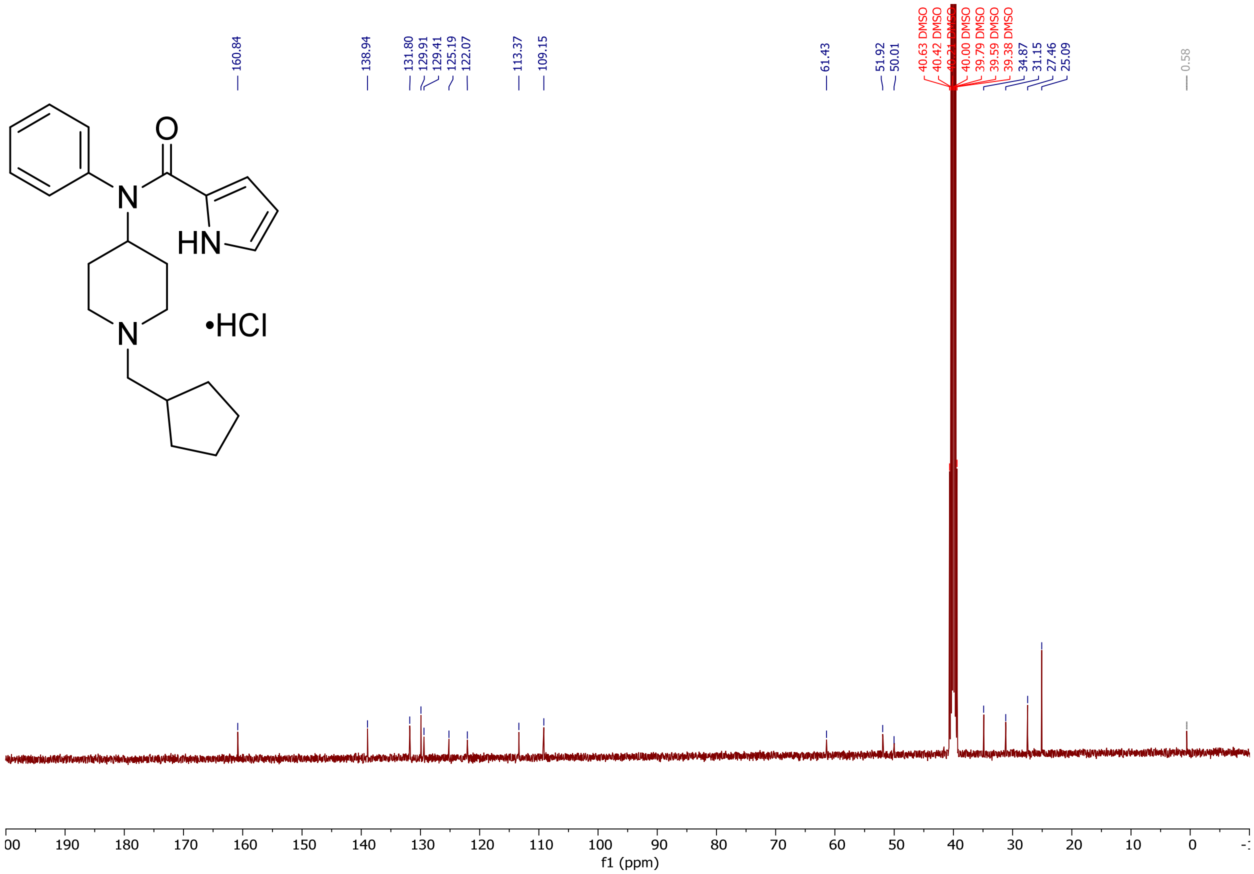

^{13}C NMR (100 MHz, DMSO-d_6) spectrum for compound **28**.

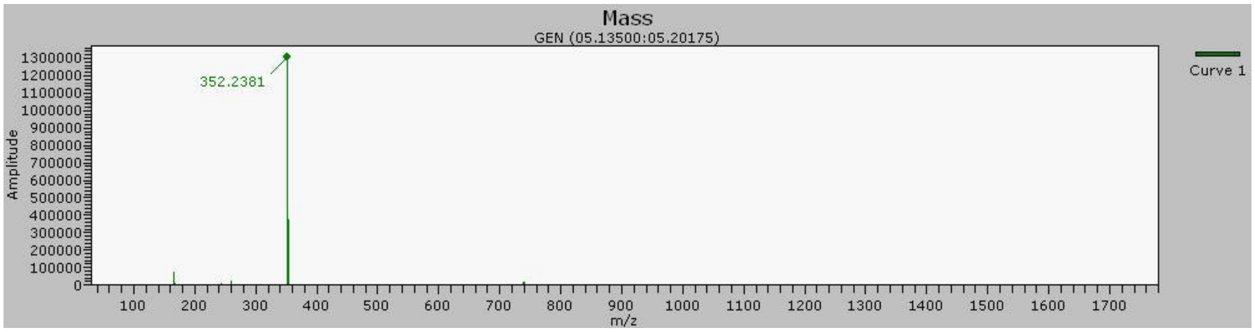

HRMS spectrum for compound **28**.

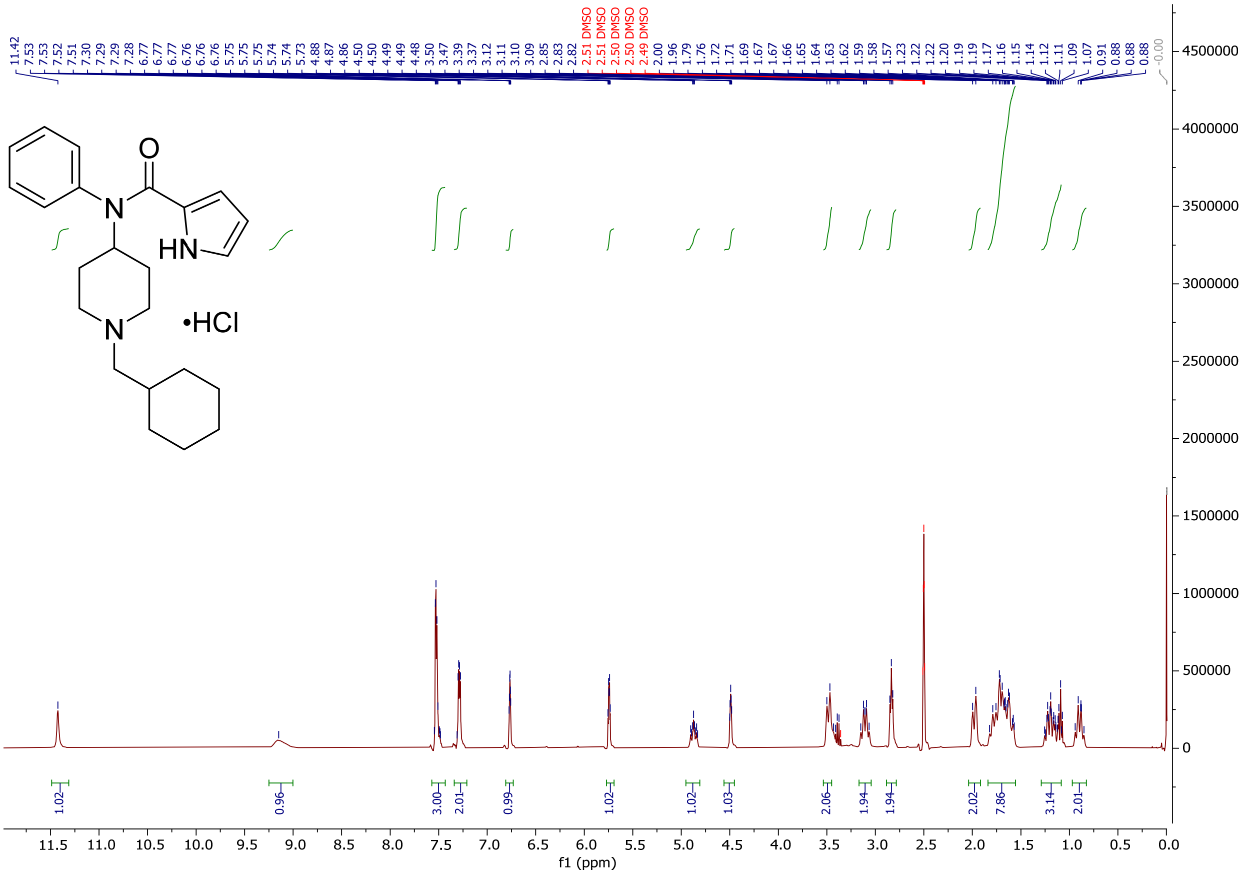

^{1}H NMR (400 MHz, DMSO-d_6) spectrum for compound **29**.

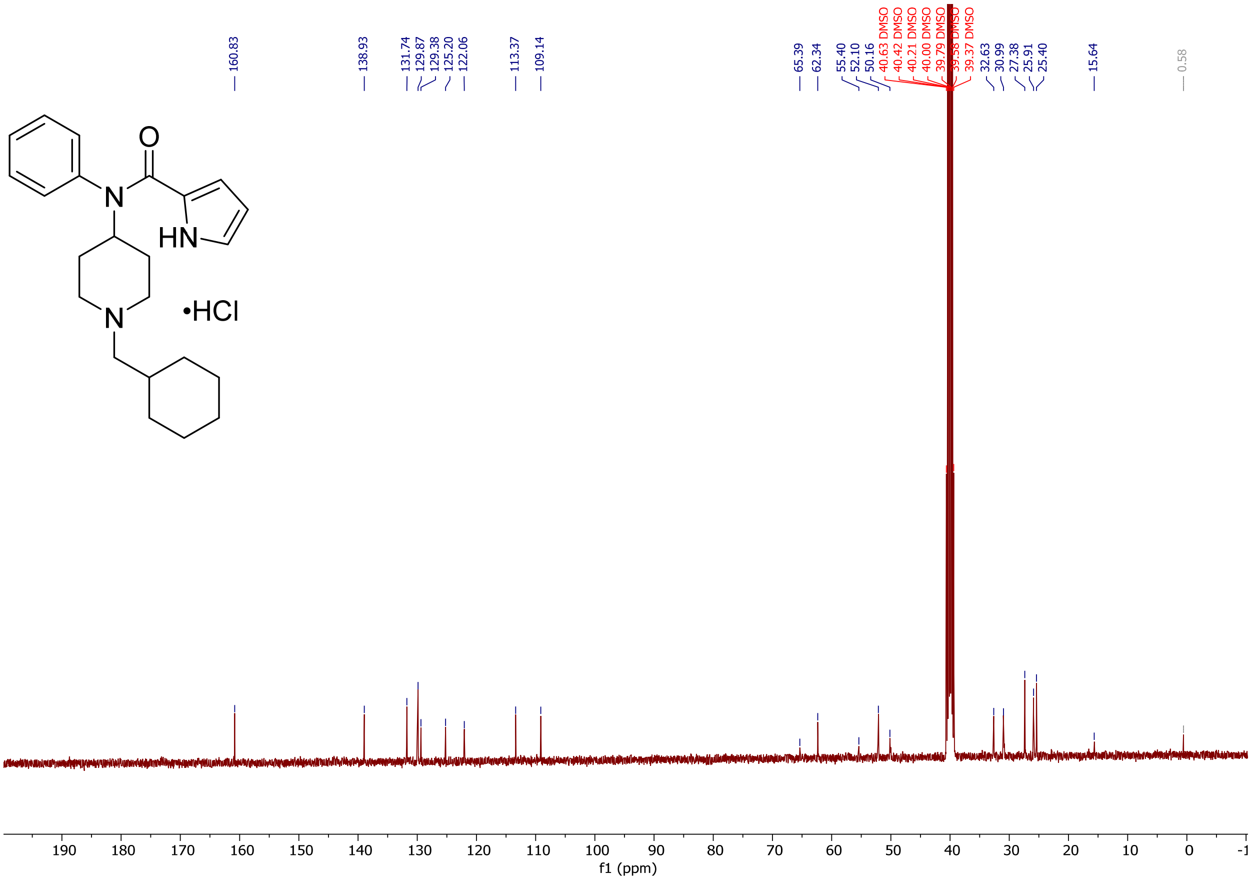

^{13}C NMR (100 MHz, DMSO-d_6) spectrum for compound **29**.

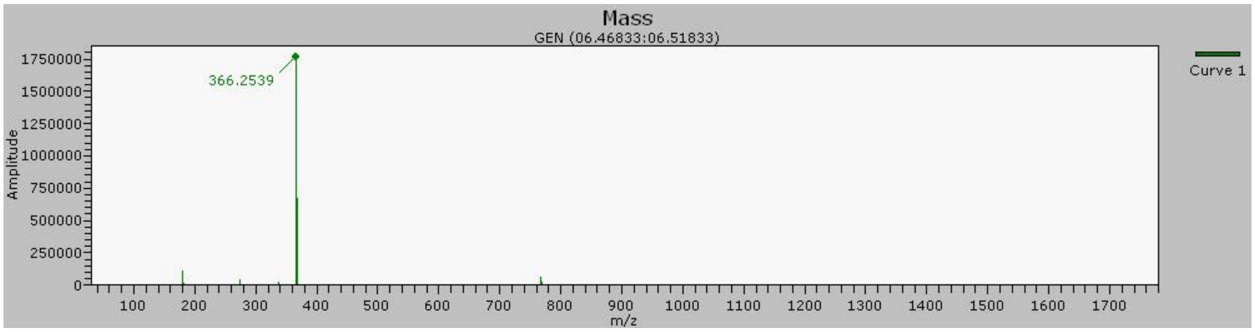

HRMS spectrum for compound **29**.

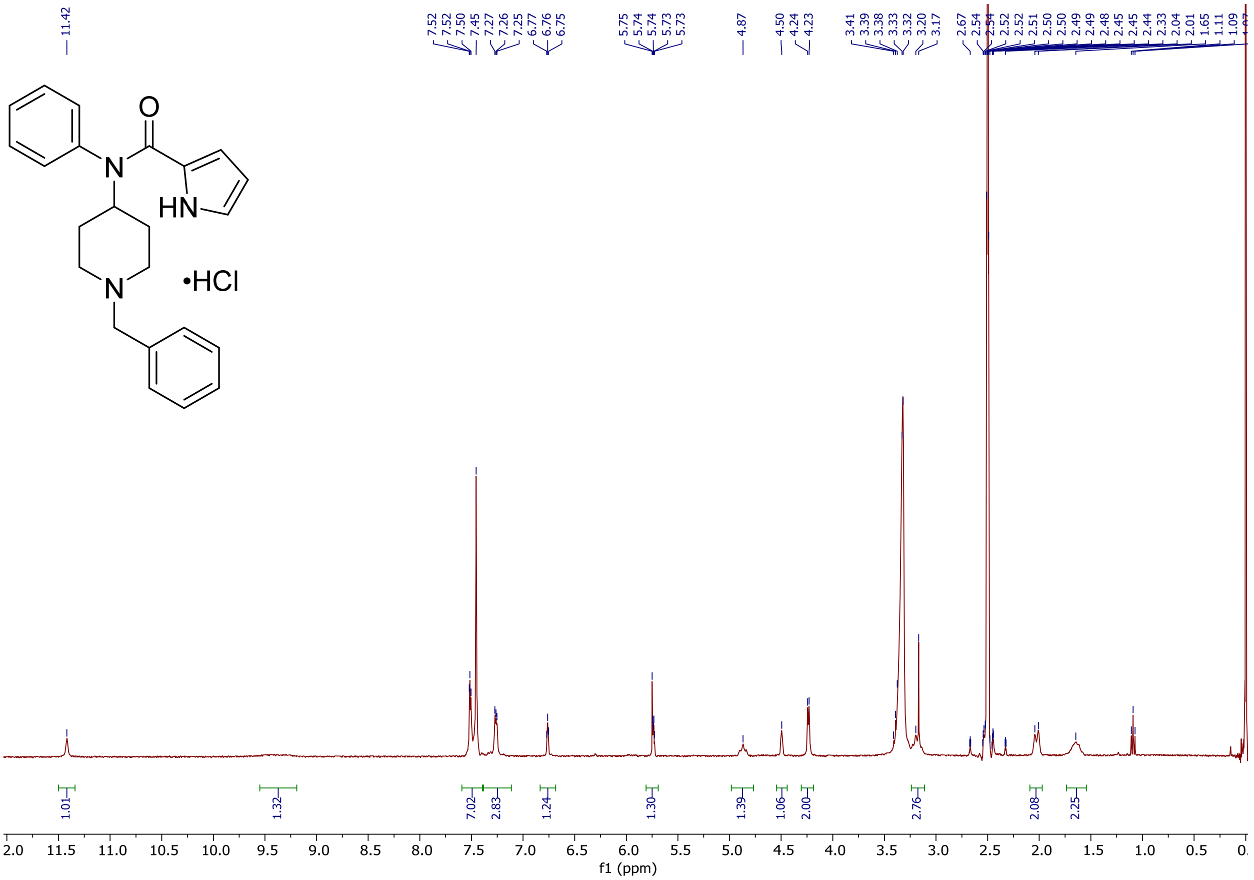

^{1}H NMR (400 MHz, DMSO-d_6) spectrum for compound **30**.

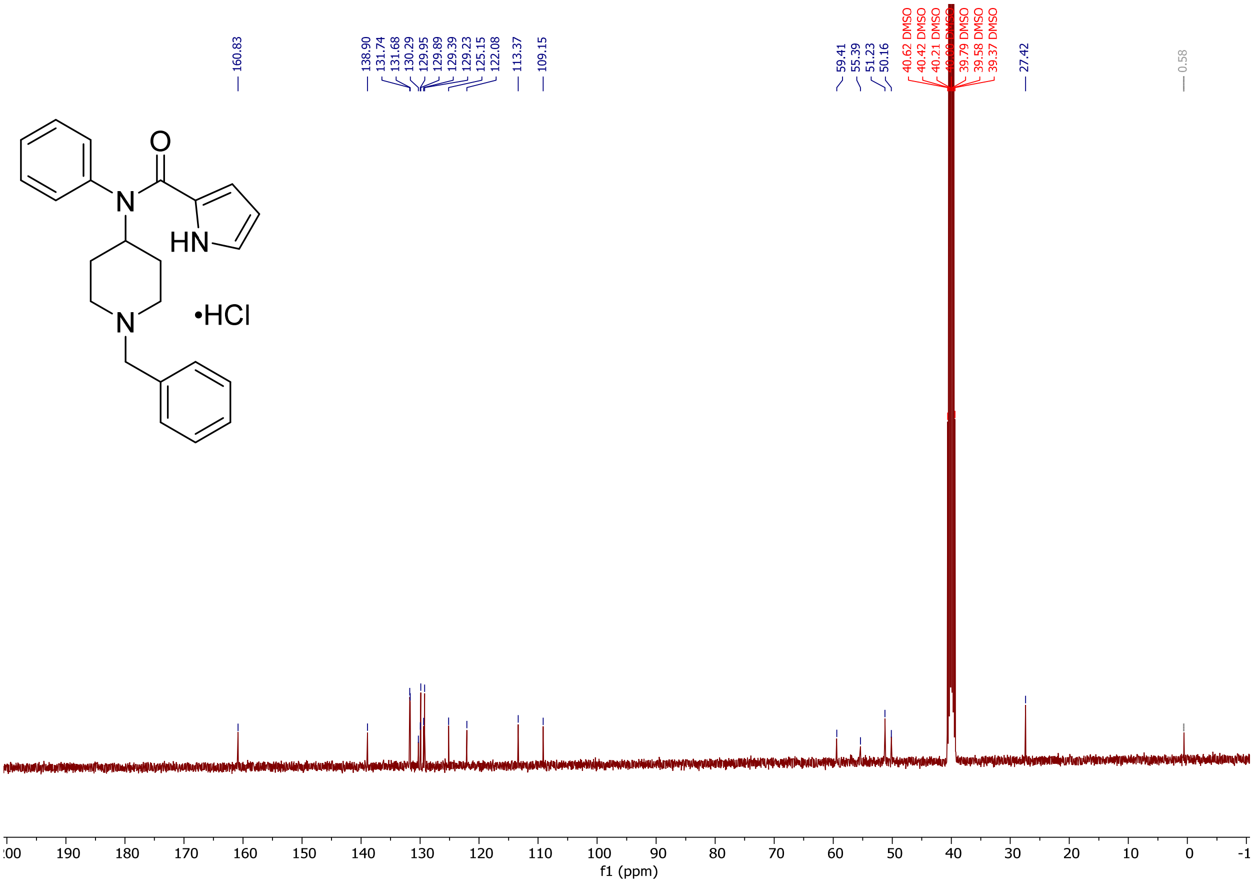

^{13}C NMR (100 MHz, DMSO-d_6) for compound **30**.

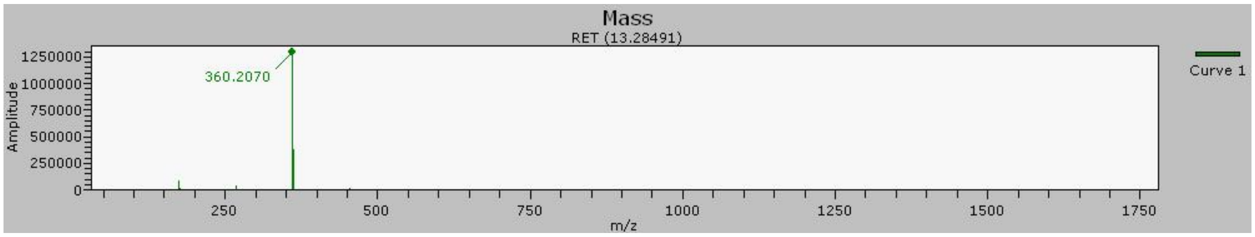

HRMS spectrum for compound **30**.

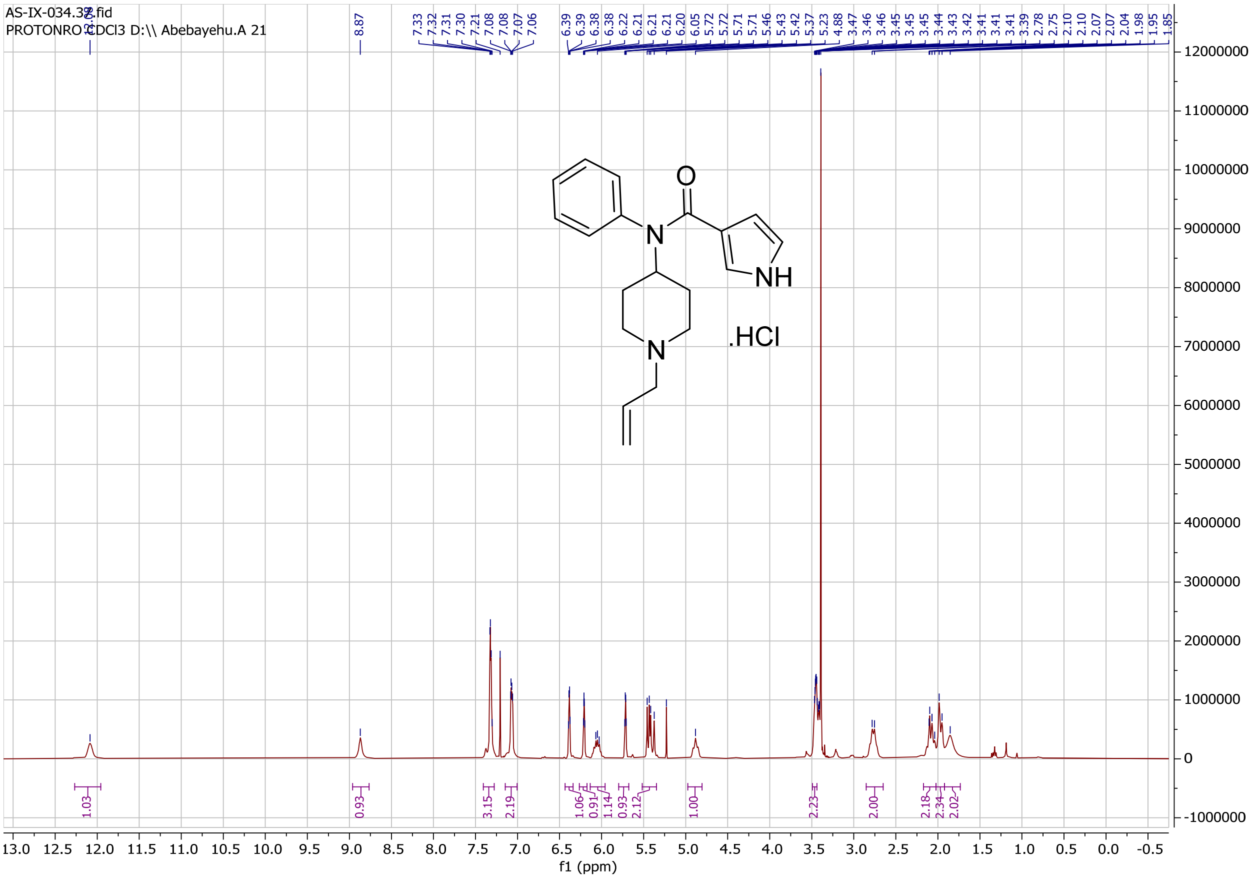

^{1}H NMR (400 MHz, DMSO-d_6) spectrum for compound **31**.

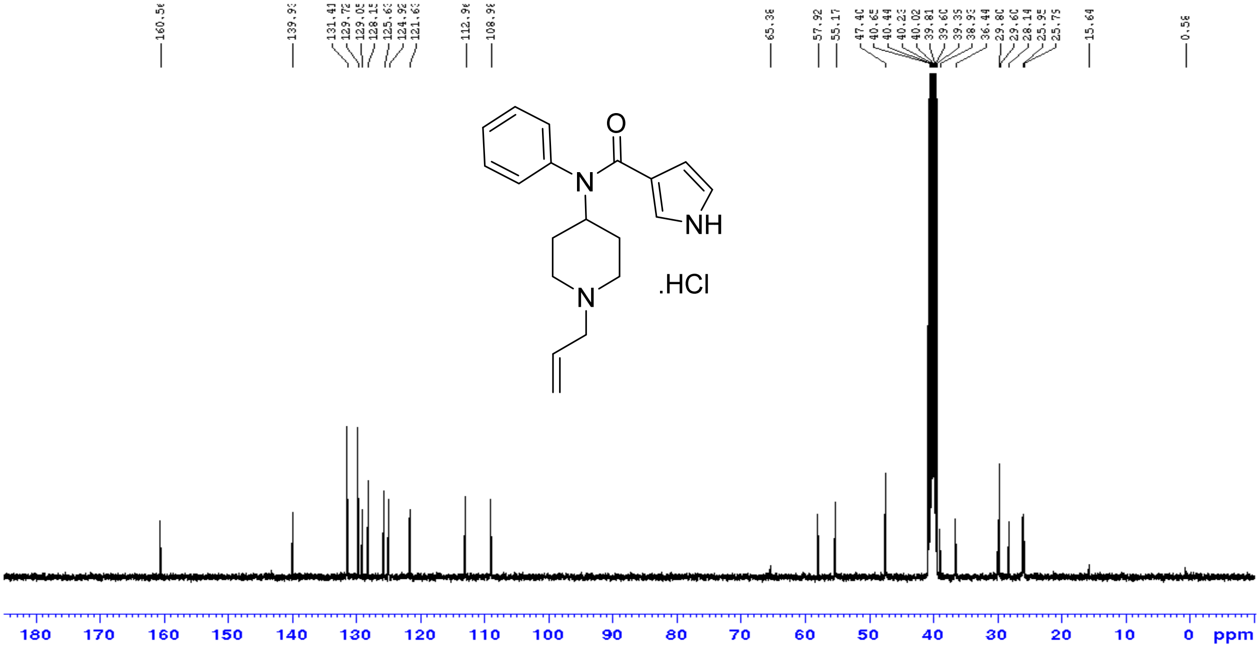

^{13}C NMR (100 MHz, DMSO-d_6) spectrum for compound **31**.

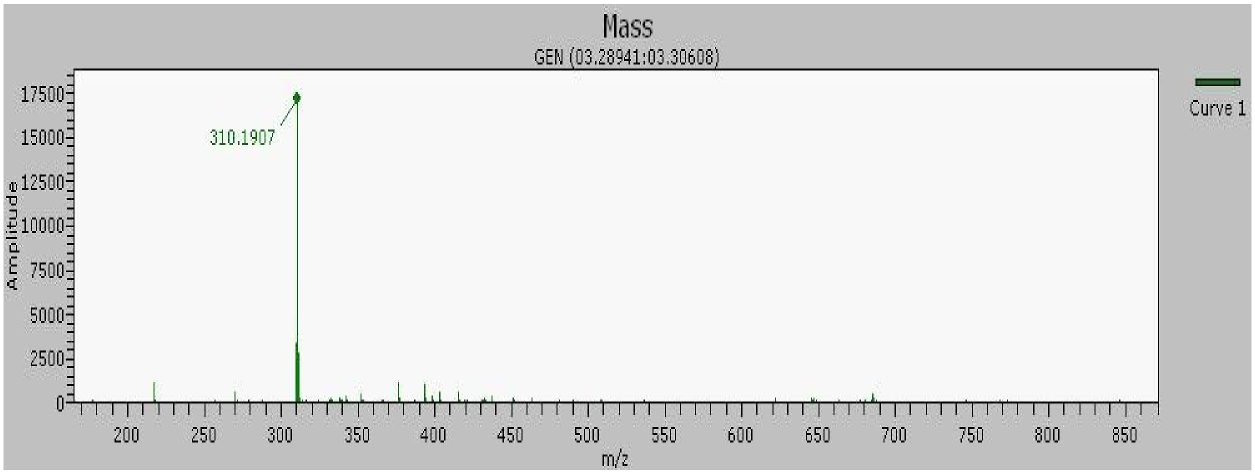

HRMS spectrum for compound **31**.

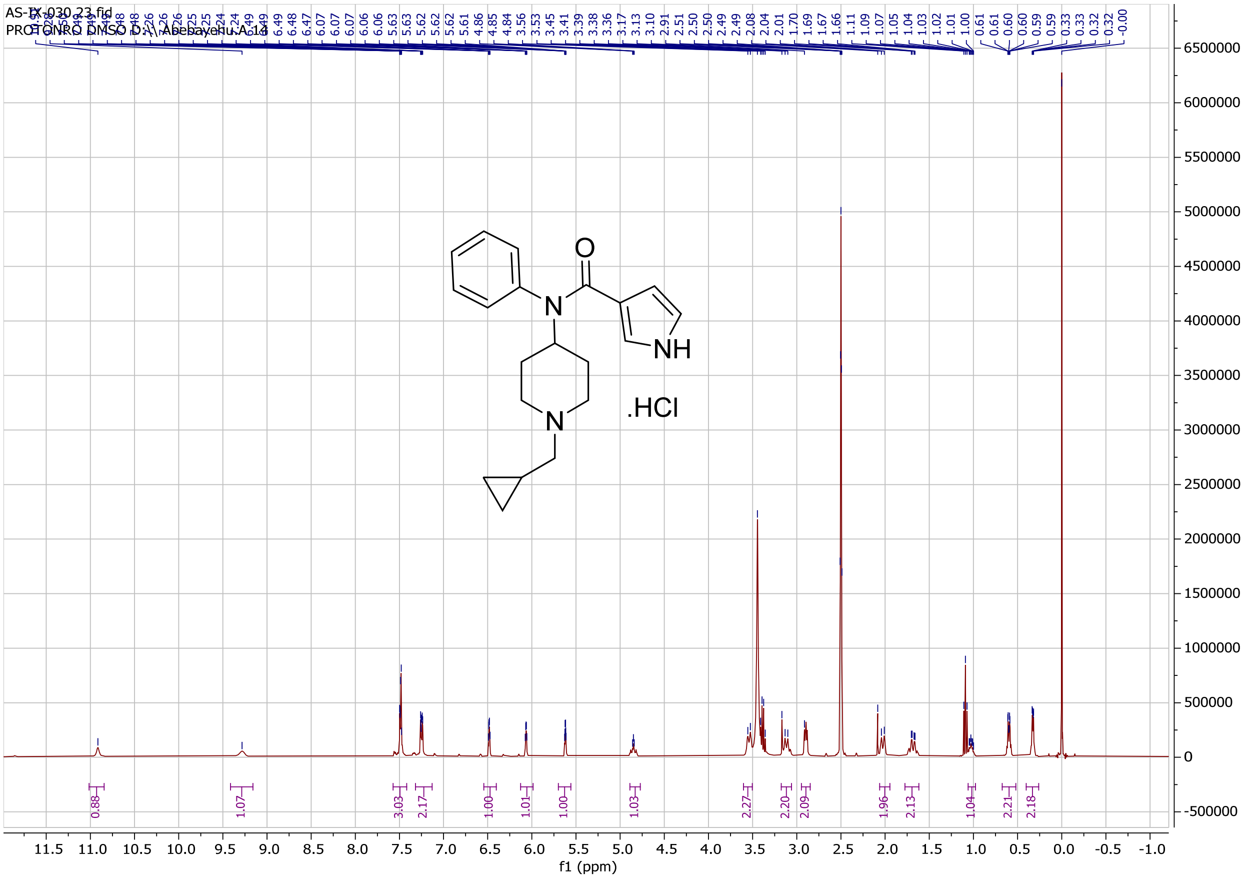

^{1}H NMR (400 MHz, DMSO-d_6) spectrum for compound **32**.

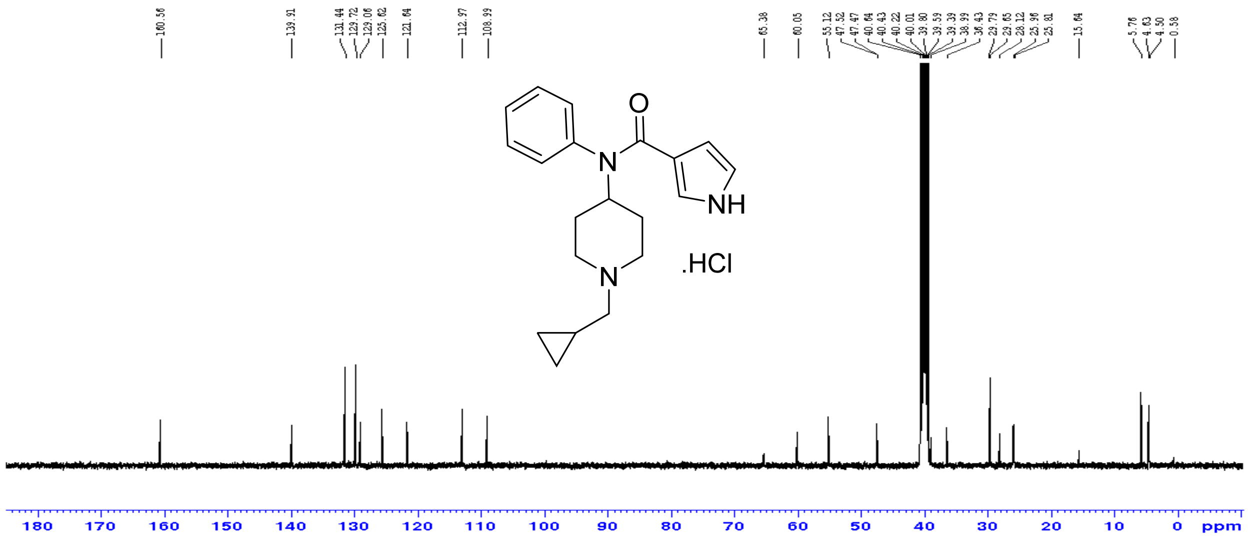

^{13}C NMR (100 MHz, DMSO-d_6) spectrum for compound **32**.

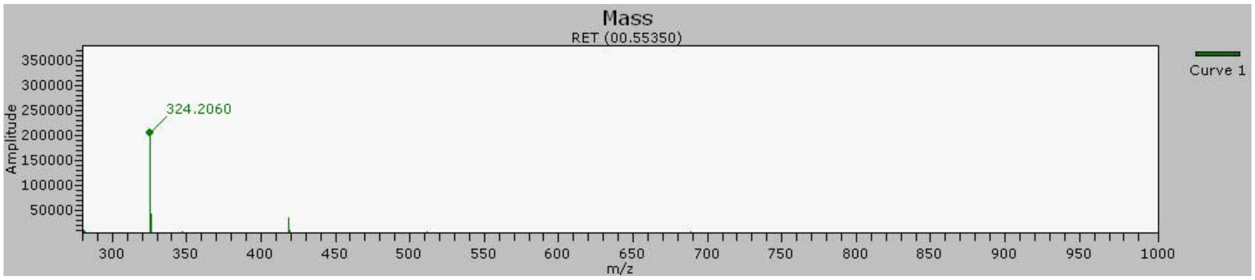

HRMS spectrum for compound **32**.

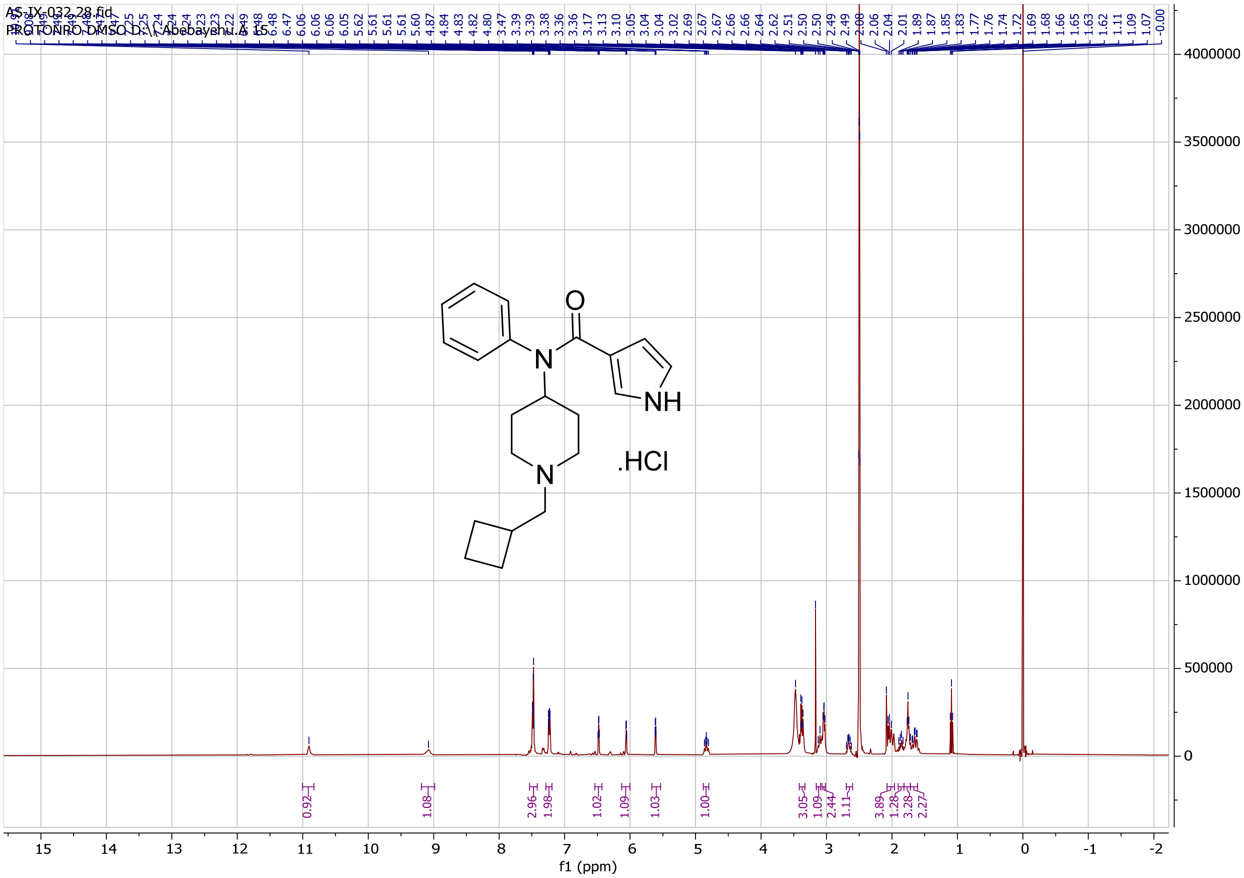

^{1}H NMR (400 MHz, DMSO-d_6) spectrum for compound **33**.

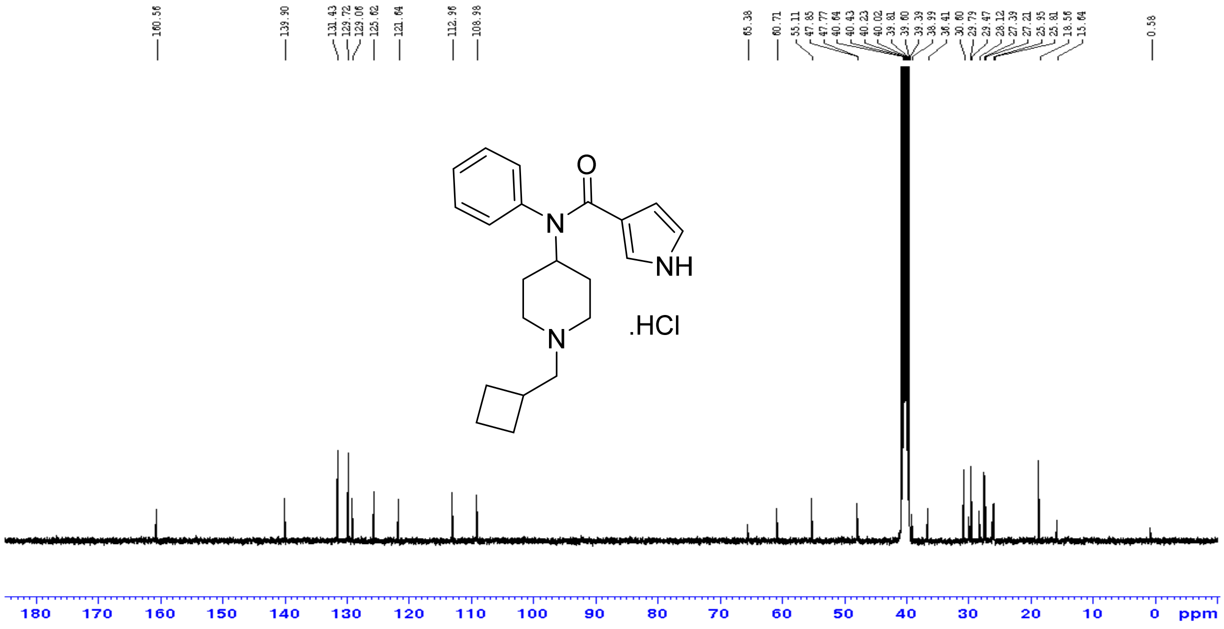

^{13}C NMR (100 MHz, DMSO-d_6) spectrum for compound **33**.

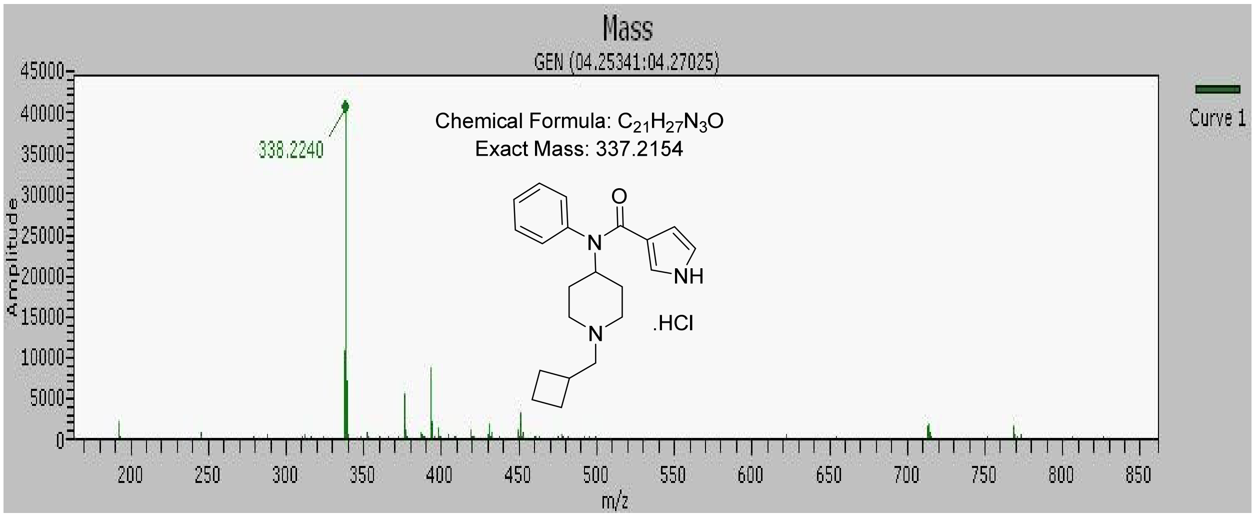

HRMS spectrum for compound **33**.

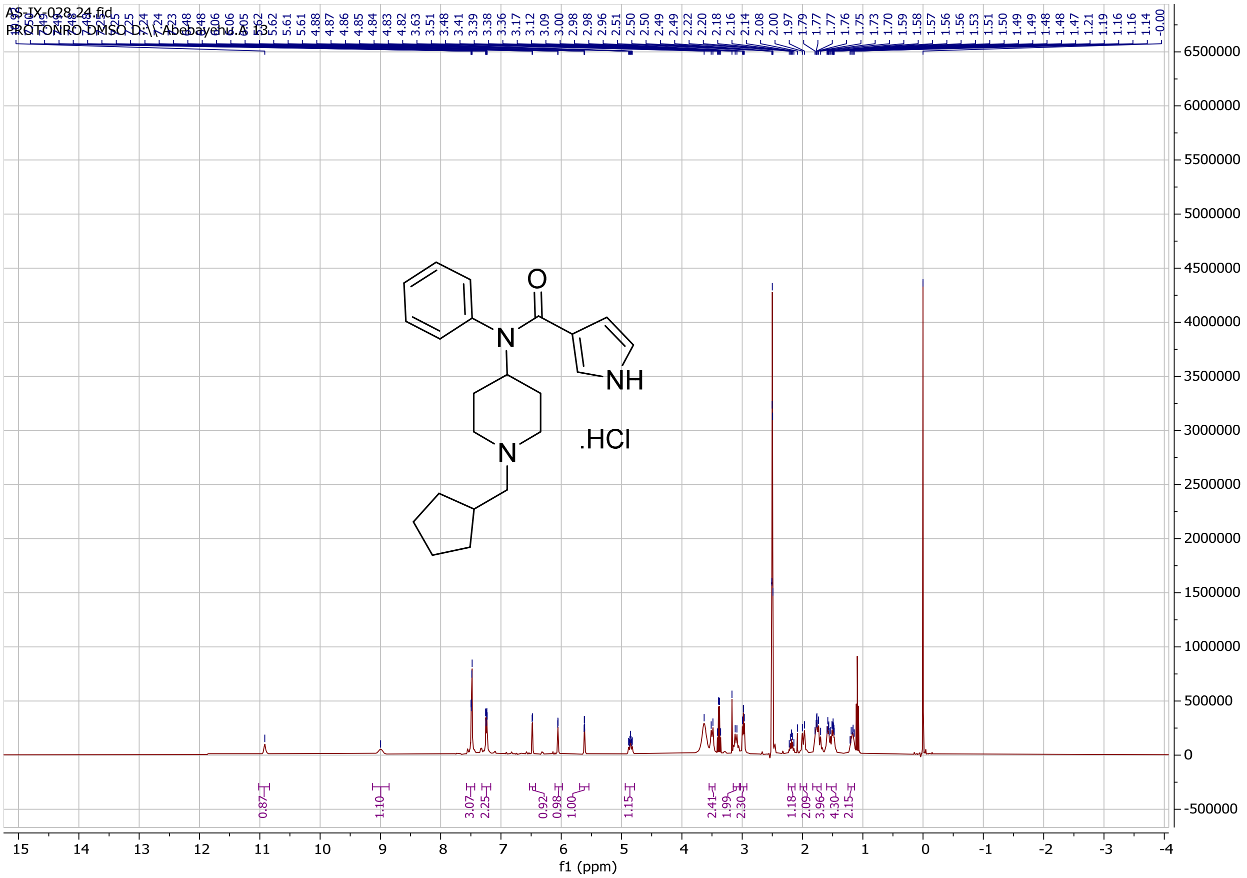

^{1}H NMR (400 MHz, DMSO-d_6) spectrum for compound **34**.

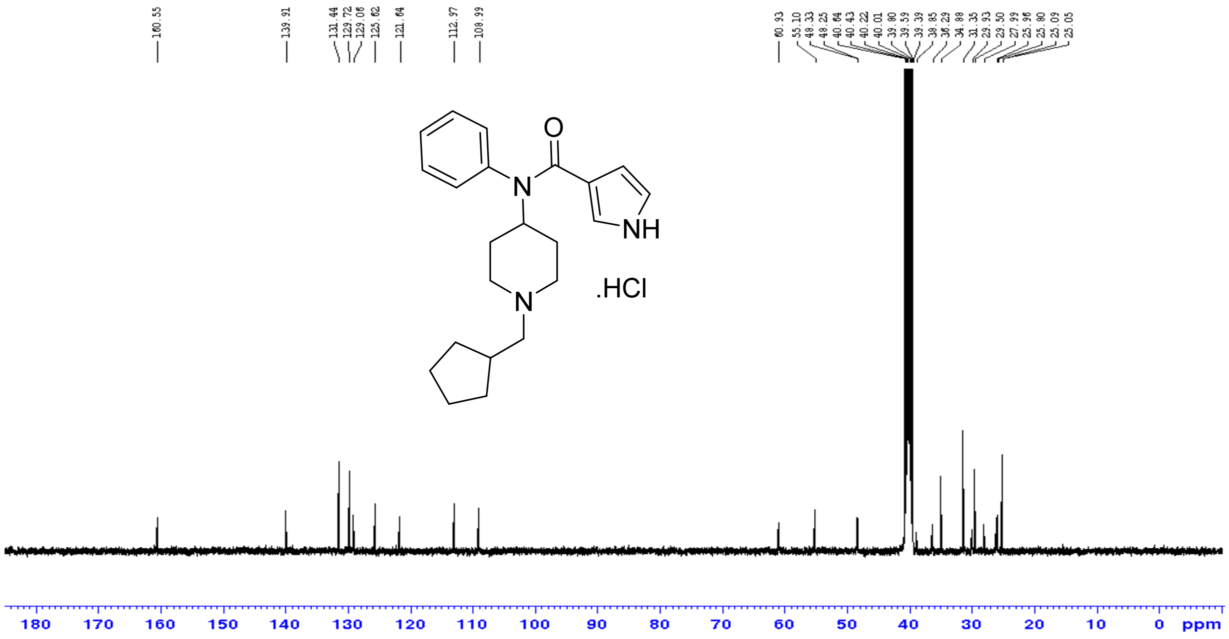

^{13}C NMR (100 MHz, DMSO-d_6) spectrum for compound **34**.

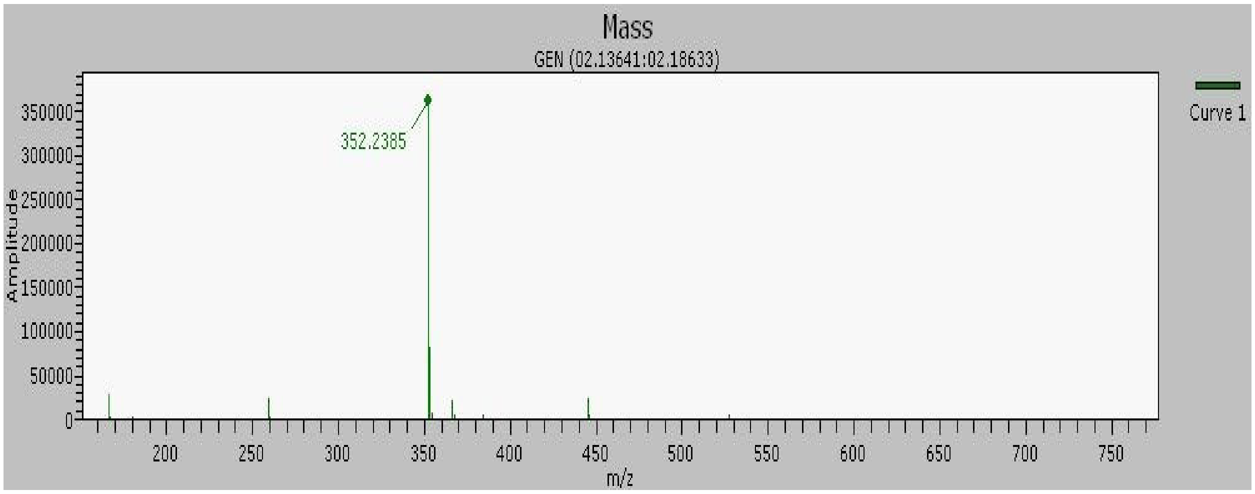

HRMS spectrum for compound **34**.

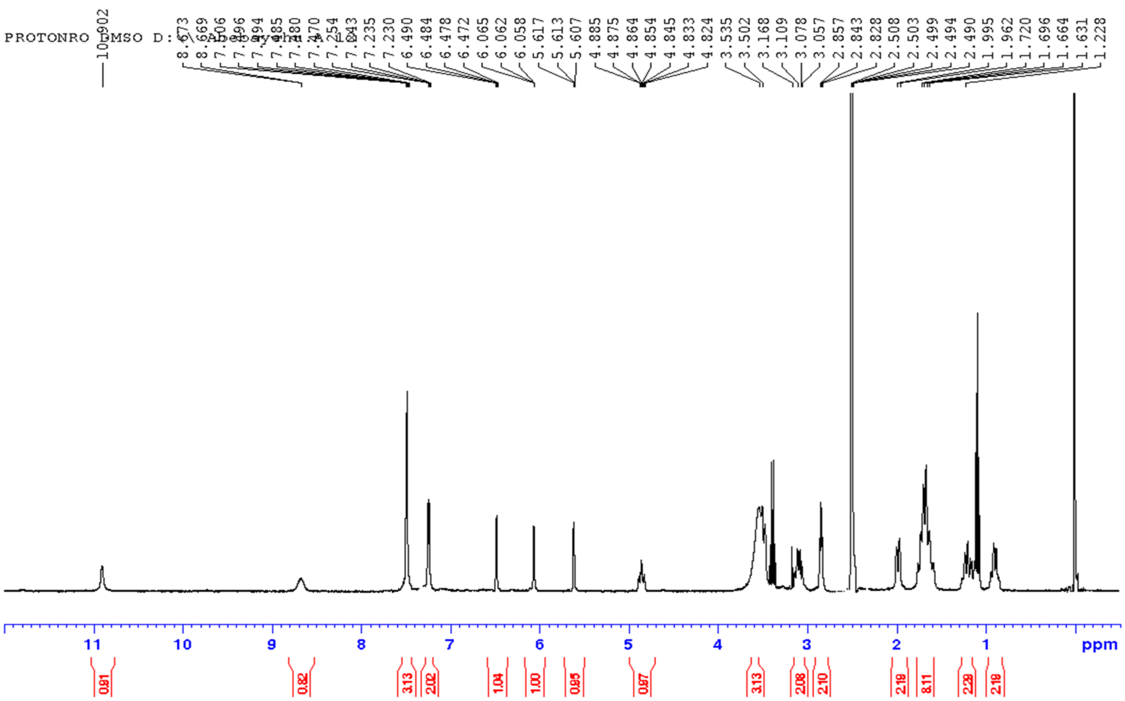

^{1}H NMR (400 MHz, DMSO-d_6) spectrum for compound **35**.

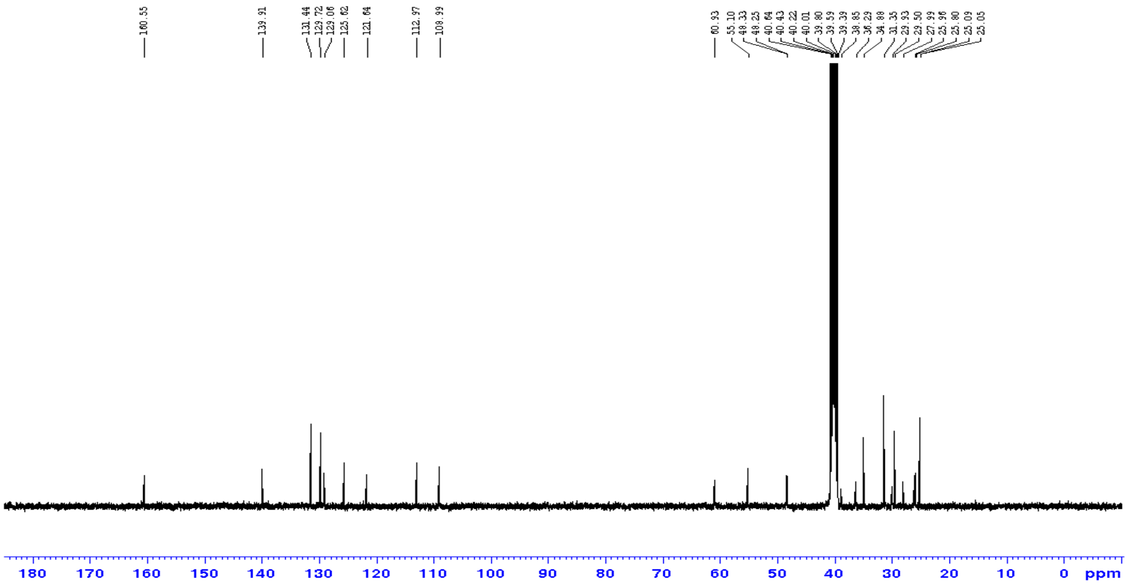

^{13}C NMR (100 MHz, DMSO-d_6) spectrum for compound **35**.

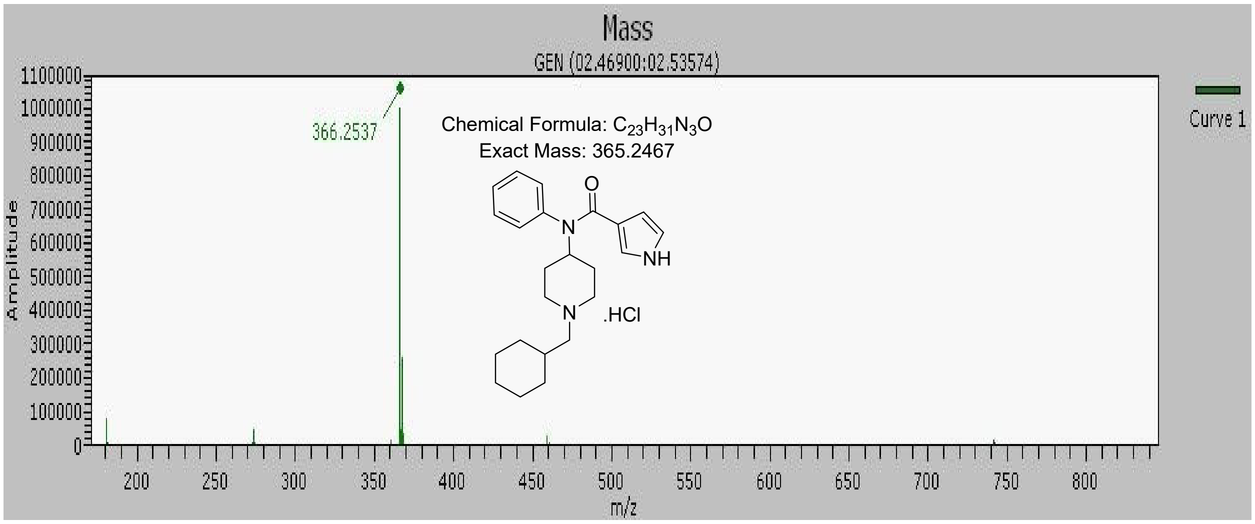

HRMS spectrum for compound **35**.

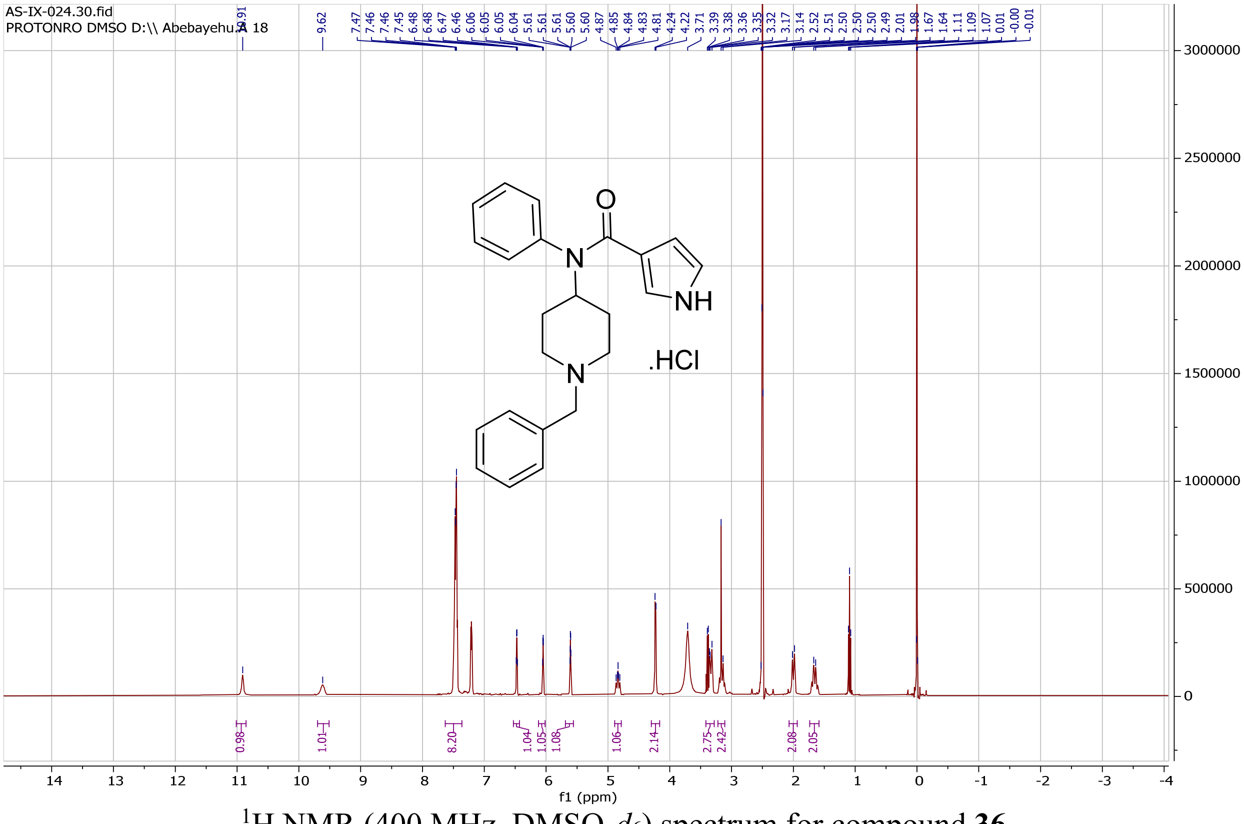

^{1}H NMR (400 MHz, DMSO-d_6) spectrum for compound **36**.

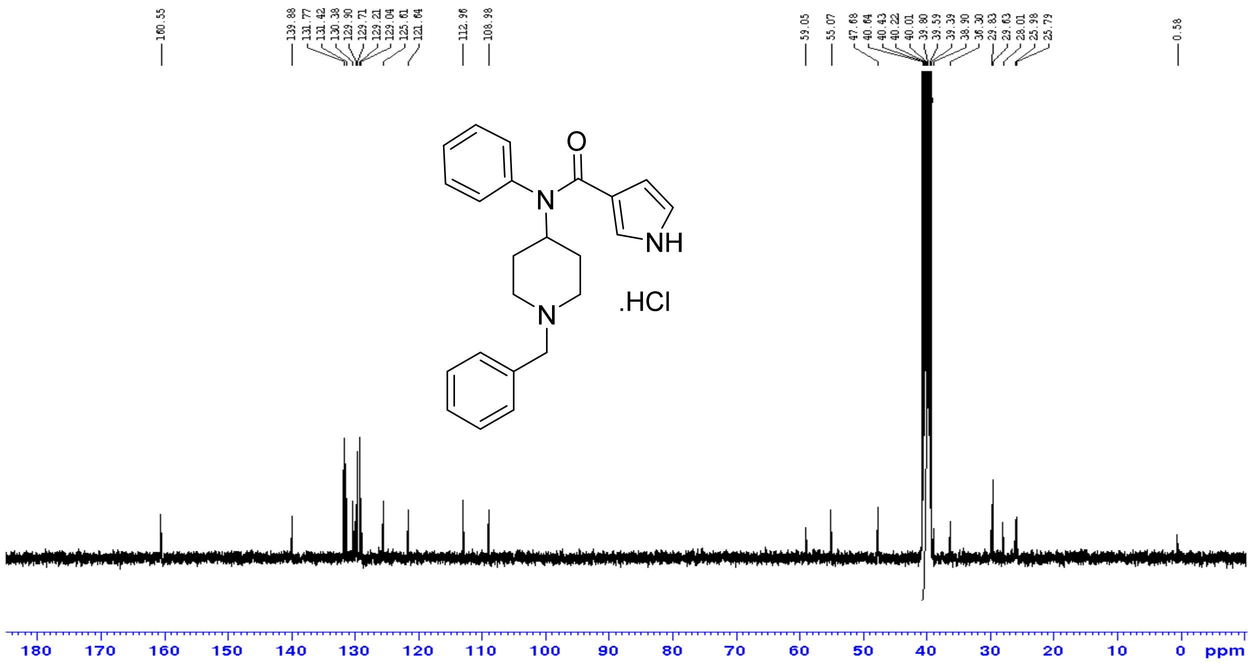

^{13}C NMR (100 MHz, DMSO-d_6) spectrum for compound **36**.

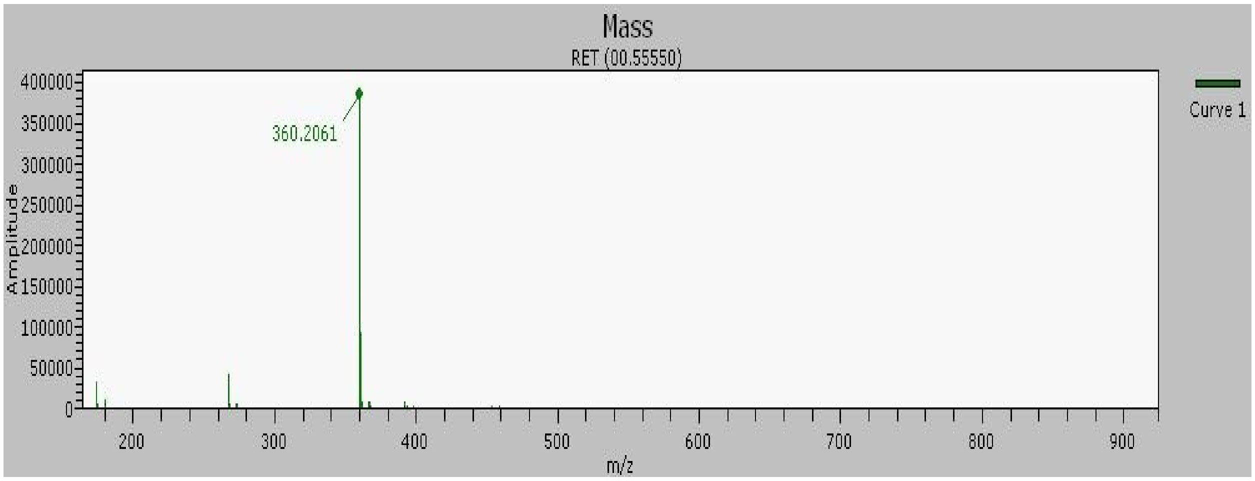

HRMS spectrum for compound **36**.

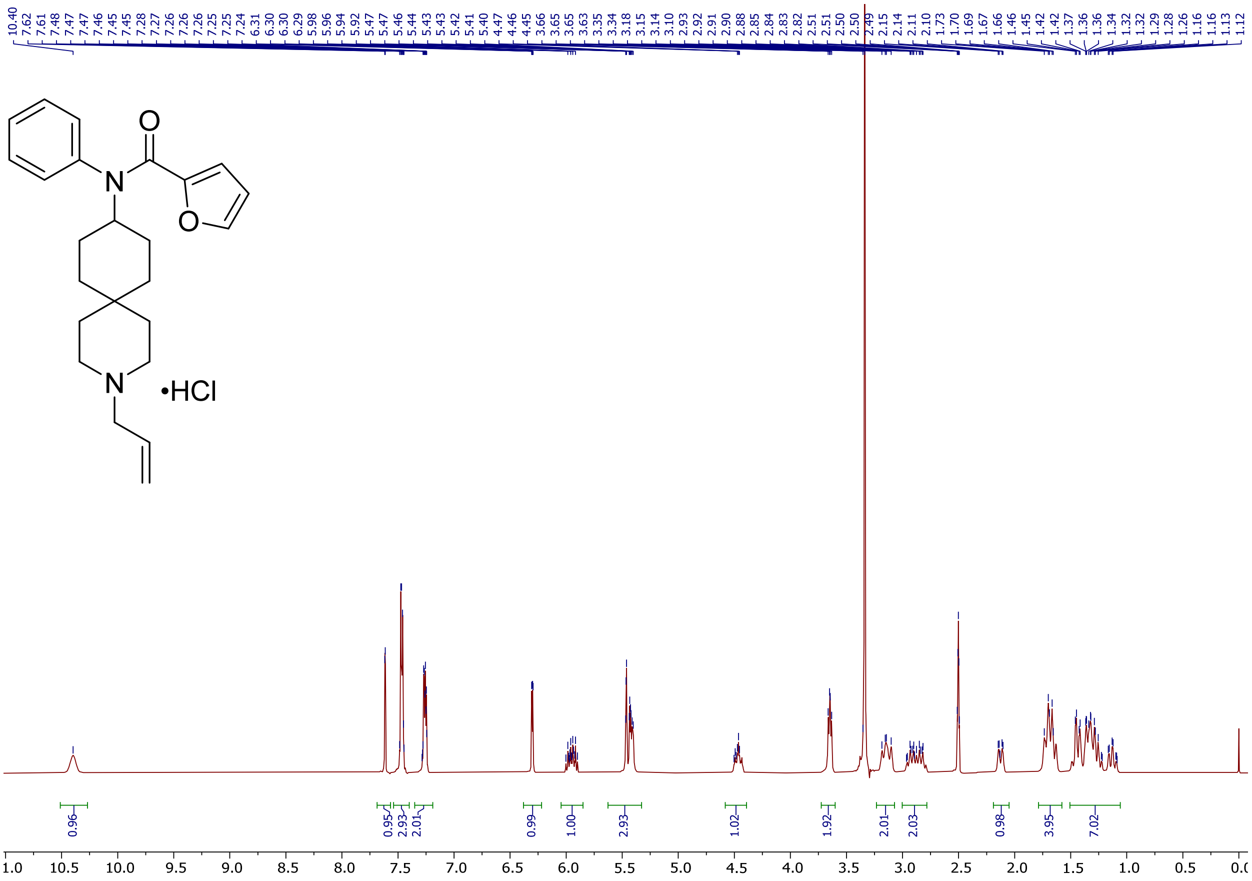

^{1}H NMR (400 MHz, DMSO-d_6) spectrum for compound **37**.

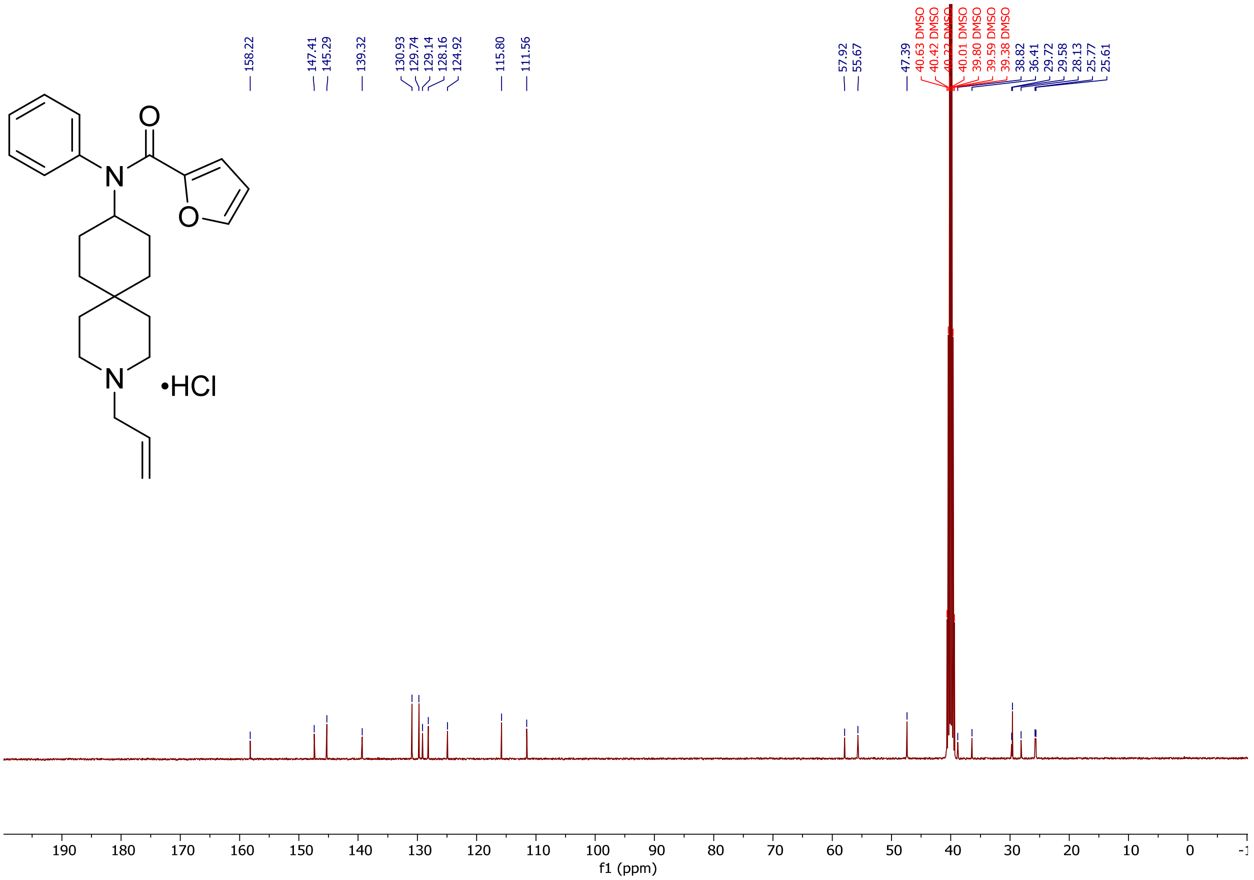

^{13}C NMR (100 MHz, DMSO-d_6) spectrum for compound **37**.

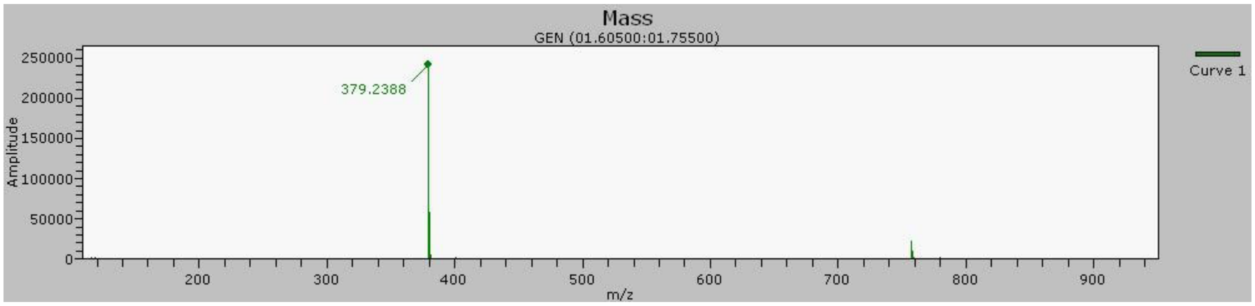

HRMS spectrum for compound **37**.

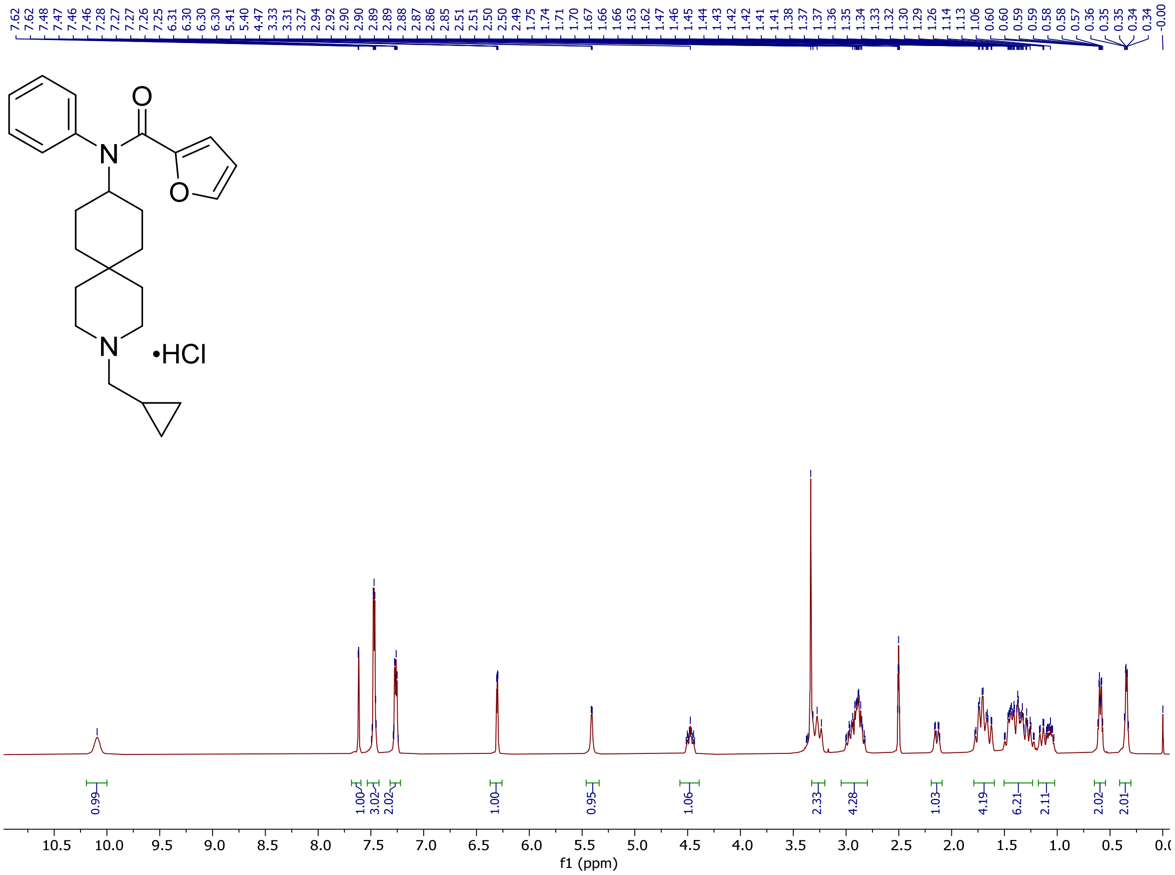

^{1}H NMR (400 MHz, DMSO-d_6) spectrum for compound **38**.

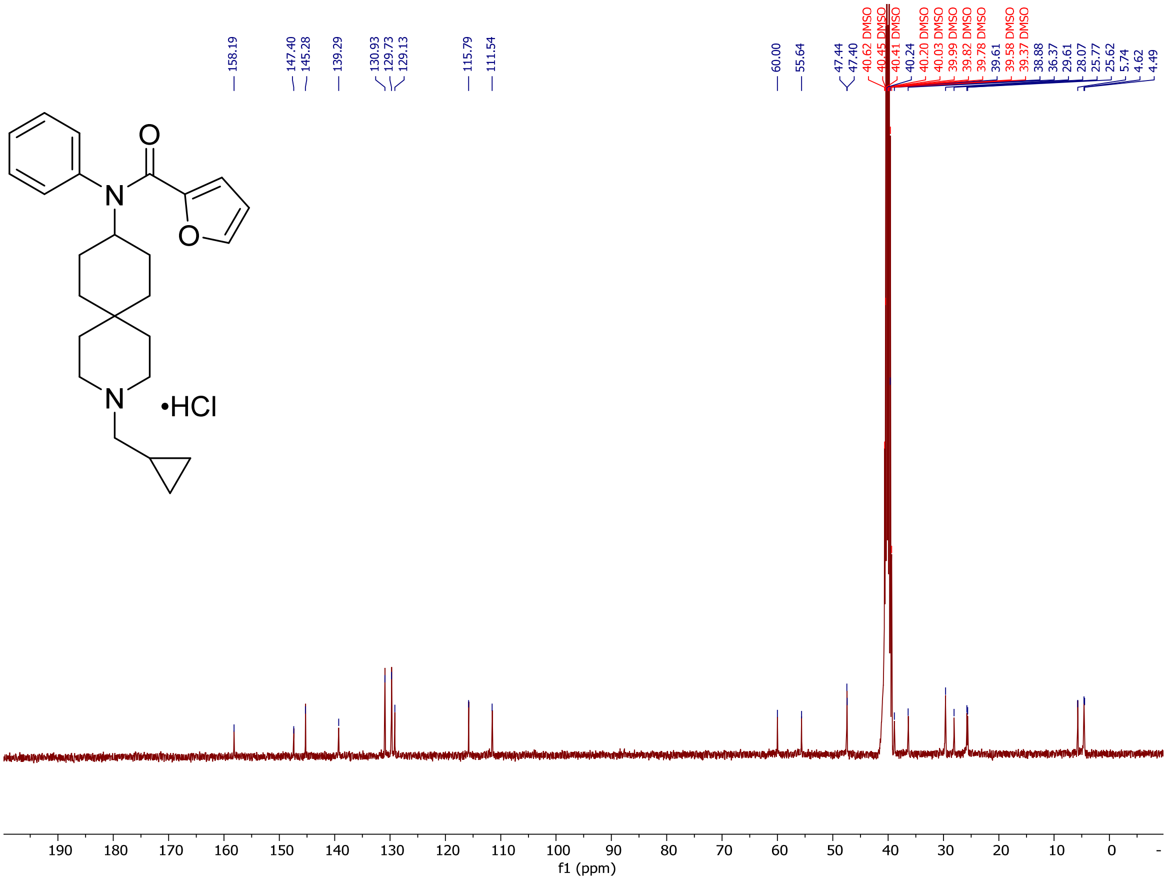

^{13}C NMR (100 MHz, DMSO-d_6) spectrum for compound **38**.

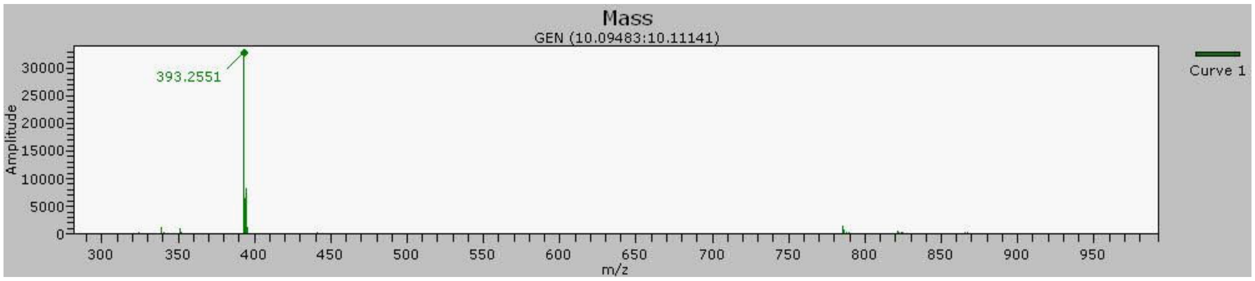

HRMS spectrum for compound **38**.

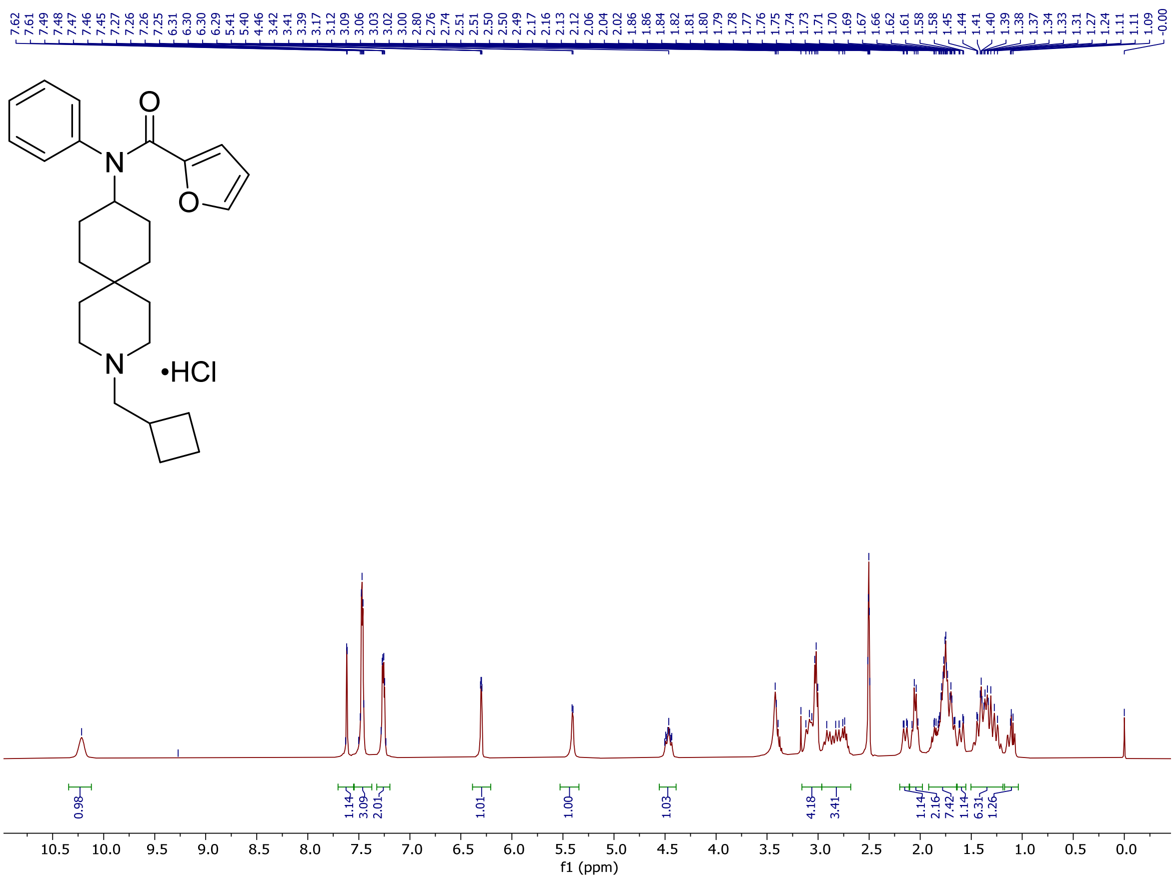

^{1}H NMR (400 MHz, DMSO-d_6) spectrum for compound **39**.

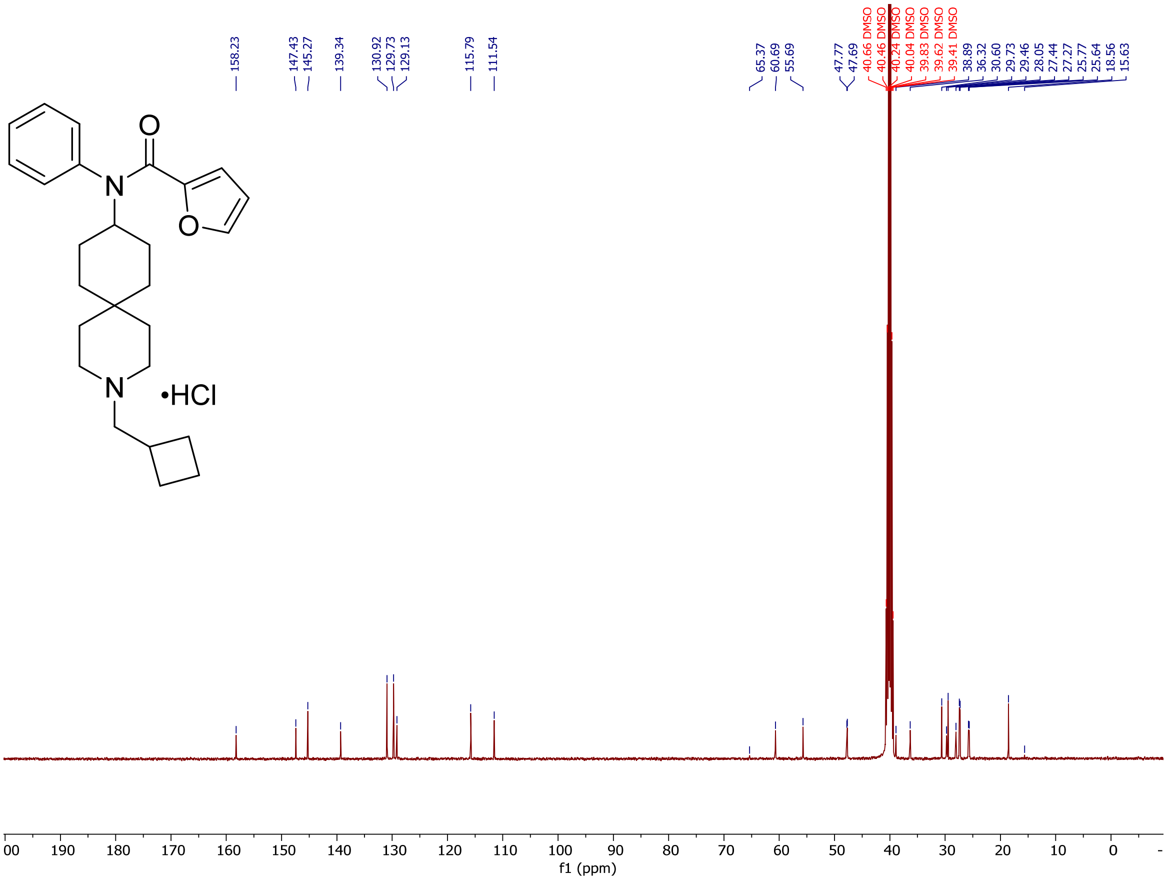

^{13}C NMR (100 MHz, DMSO-d_6) spectrum for compound **39**.

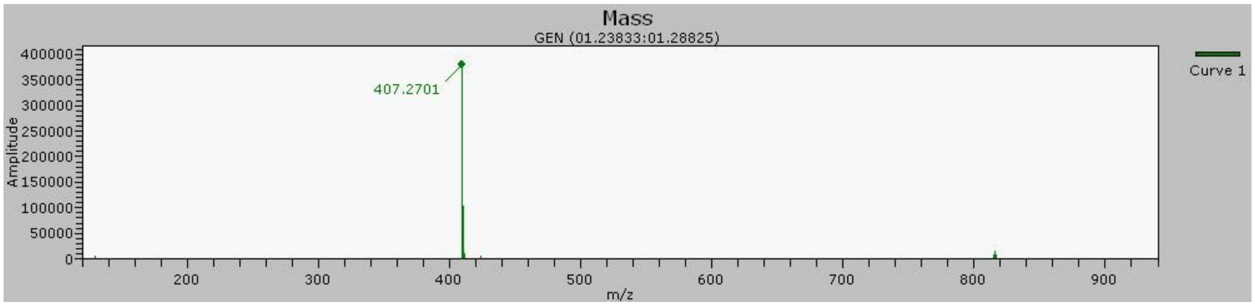

HRMS spectrum for compound **39**.

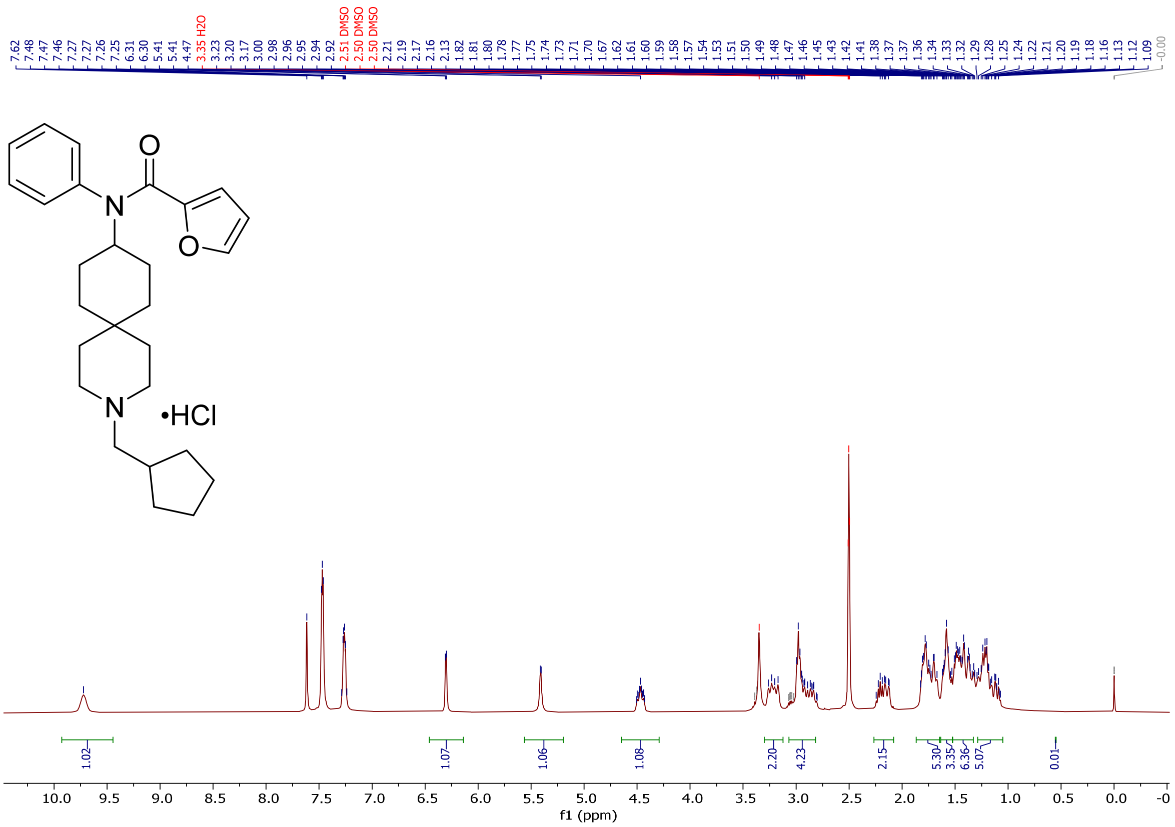

^{1}H NMR (400 MHz, DMSO-d_6) spectrum for compound **40**.

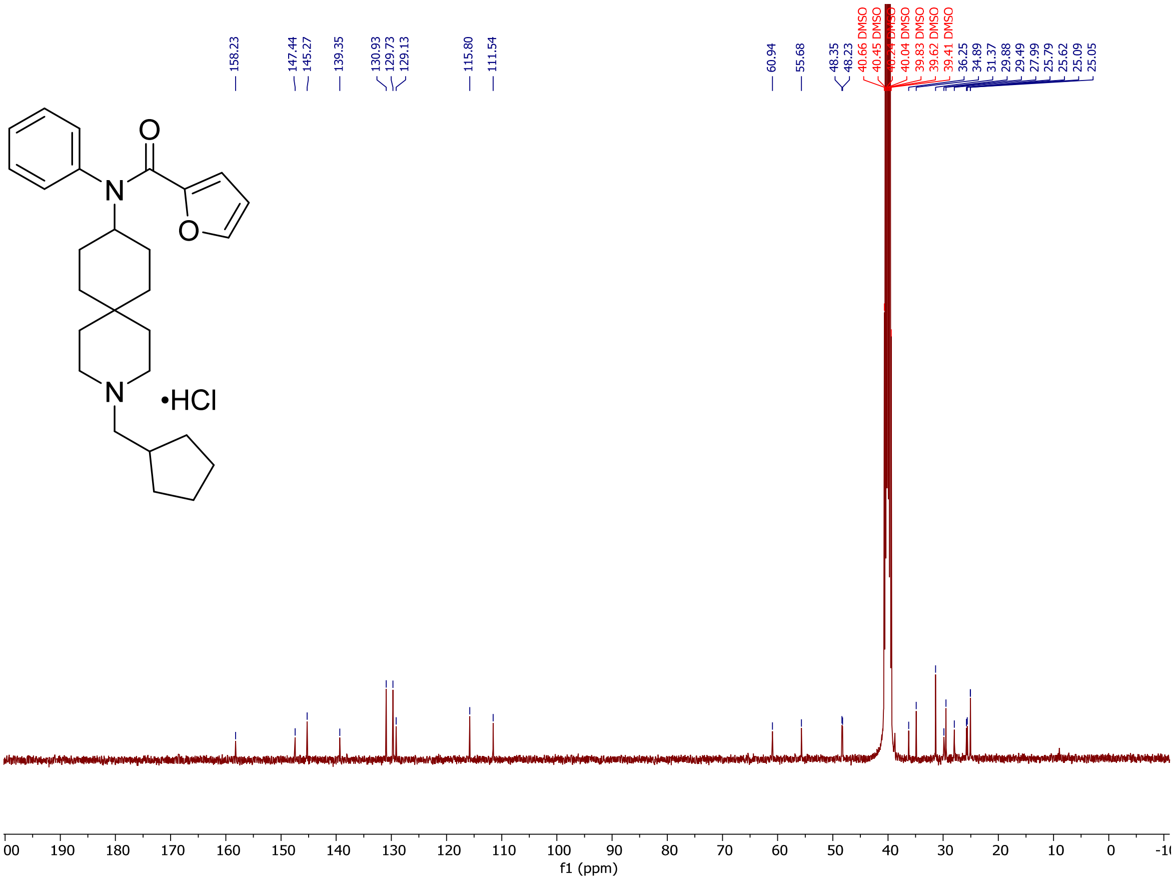

^{13}C NMR (100 MHz, DMSO-d_6) spectrum for compound **40**.

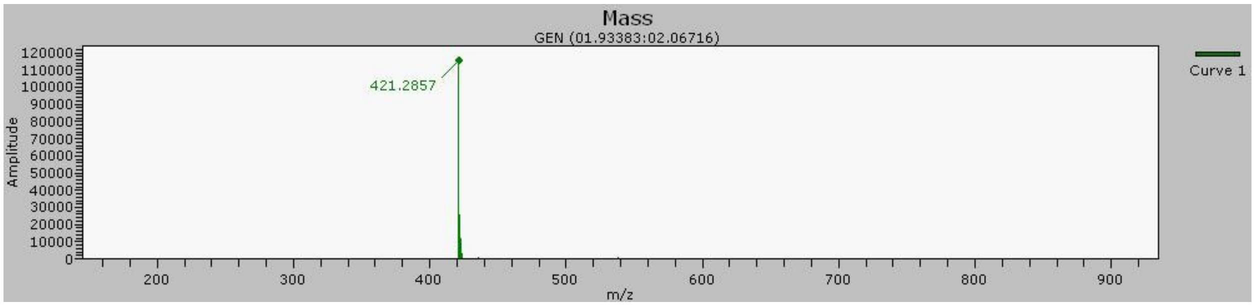

HRMS spectrum for compound **40**.

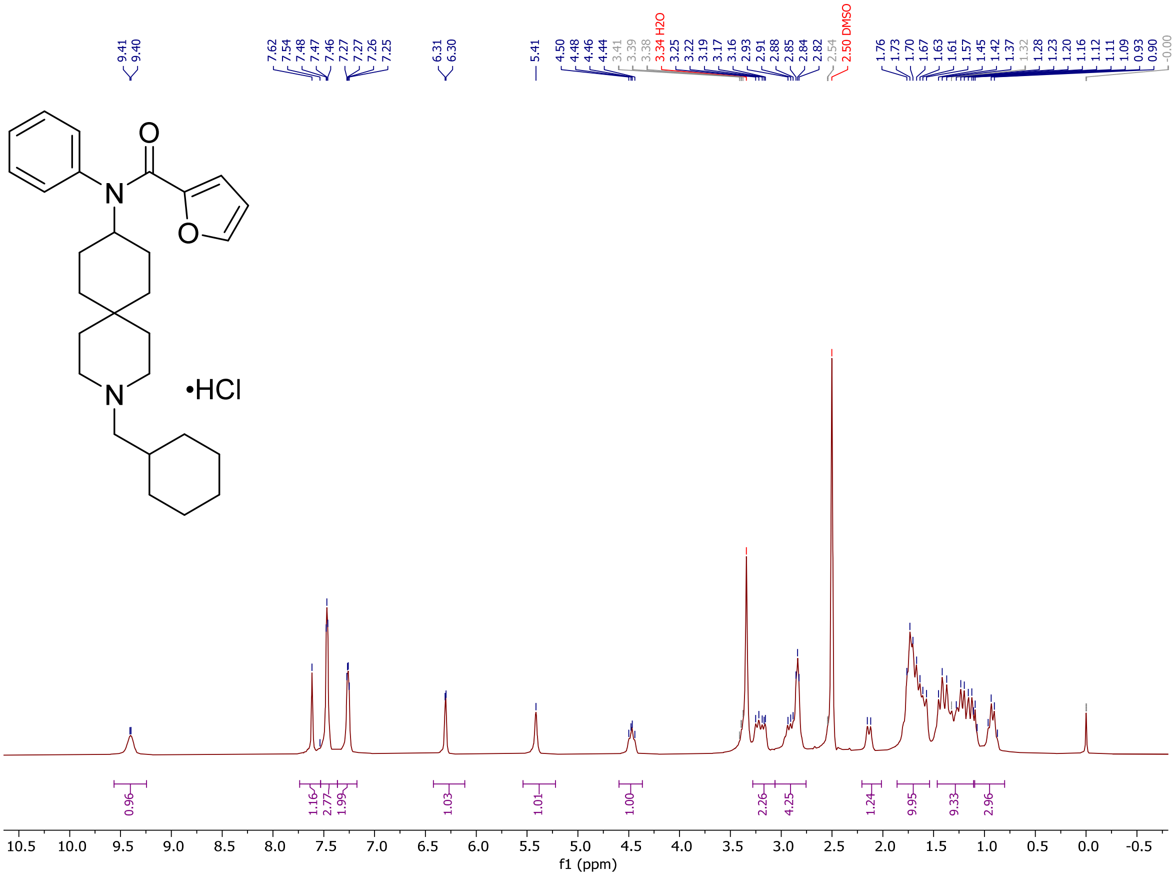

^{1}H NMR (400 MHz, DMSO-d_6) spectrum for compound **41**.

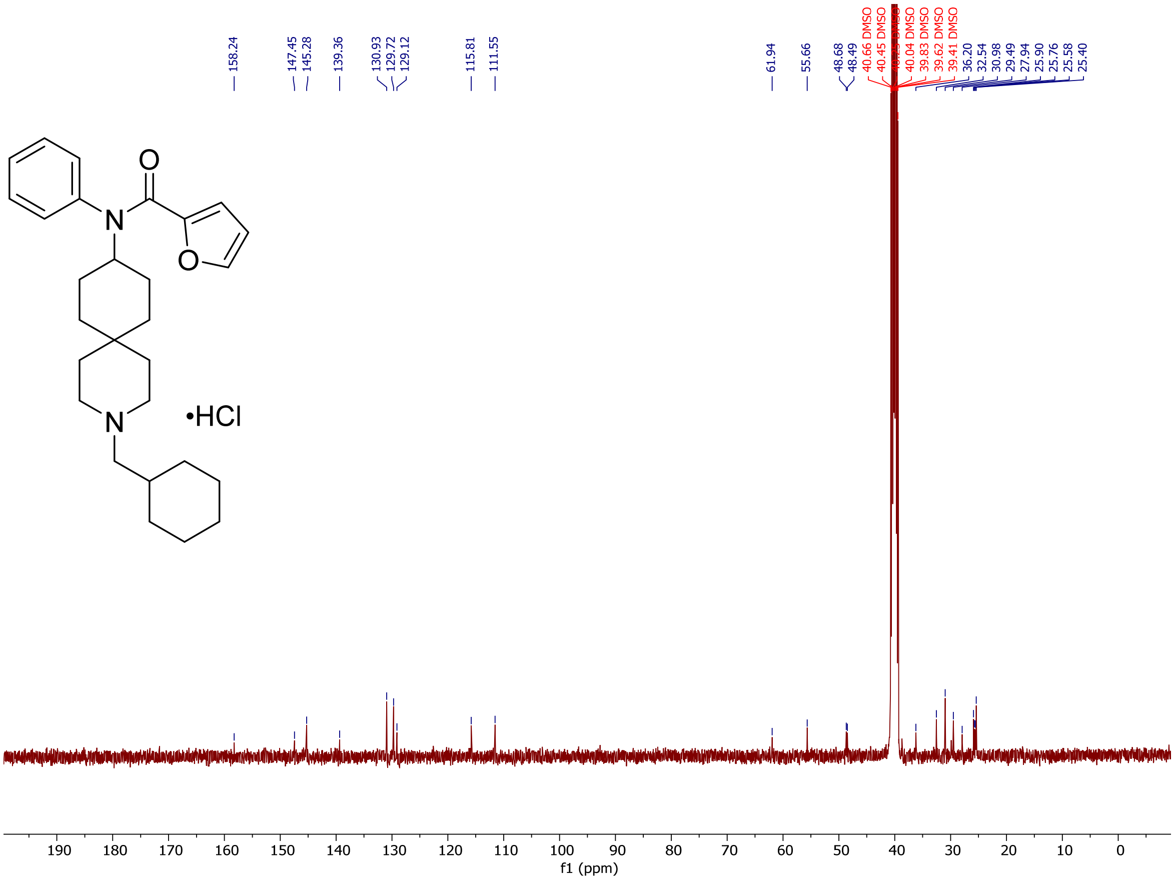

^{13}C NMR (100 MHz, DMSO-d_6) spectrum for compound **41**.

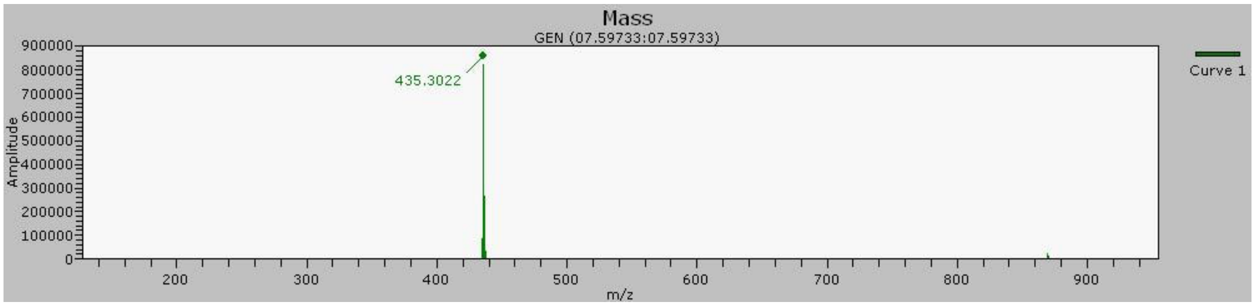

HRMS spectrum for compound **41**.

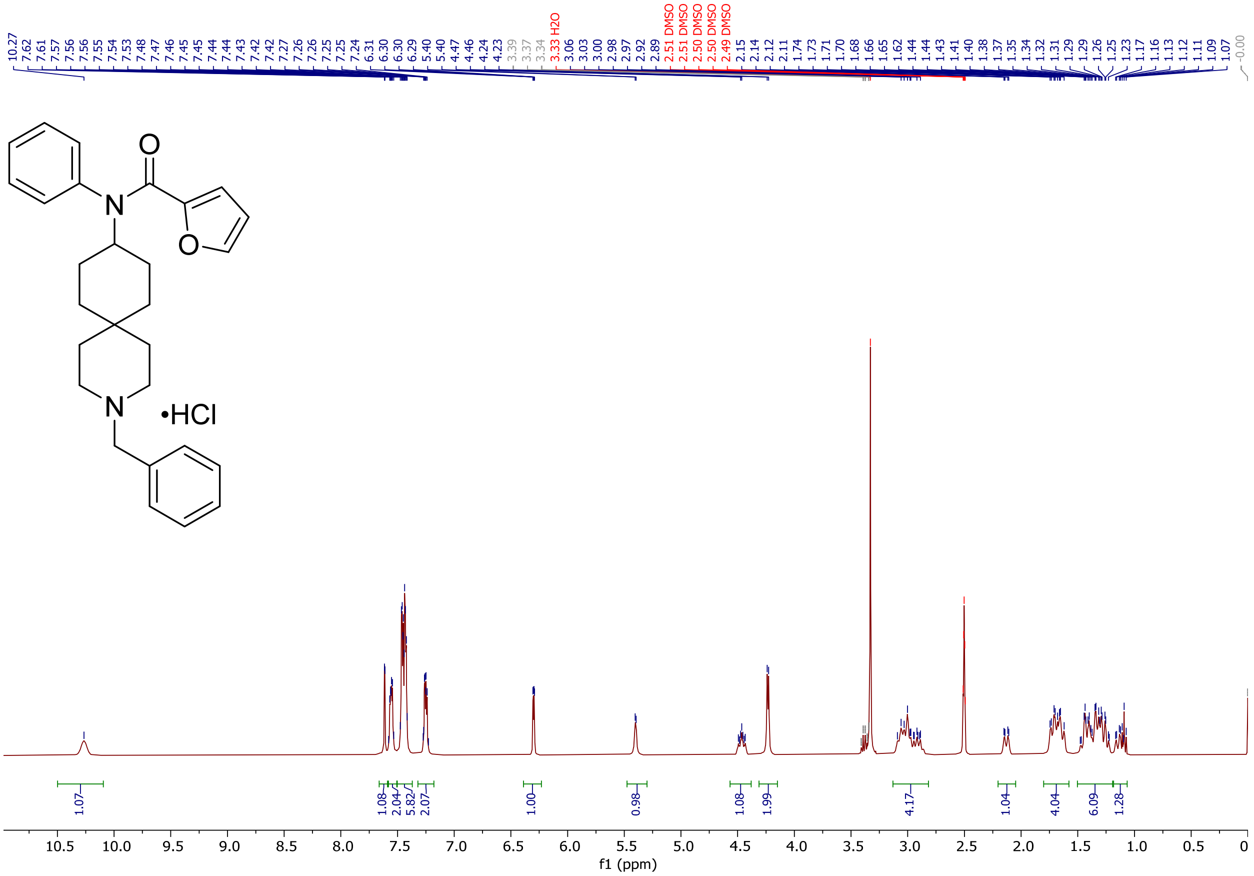

^{1}H NMR (400 MHz, DMSO-d_6) spectrum for compound **42**.

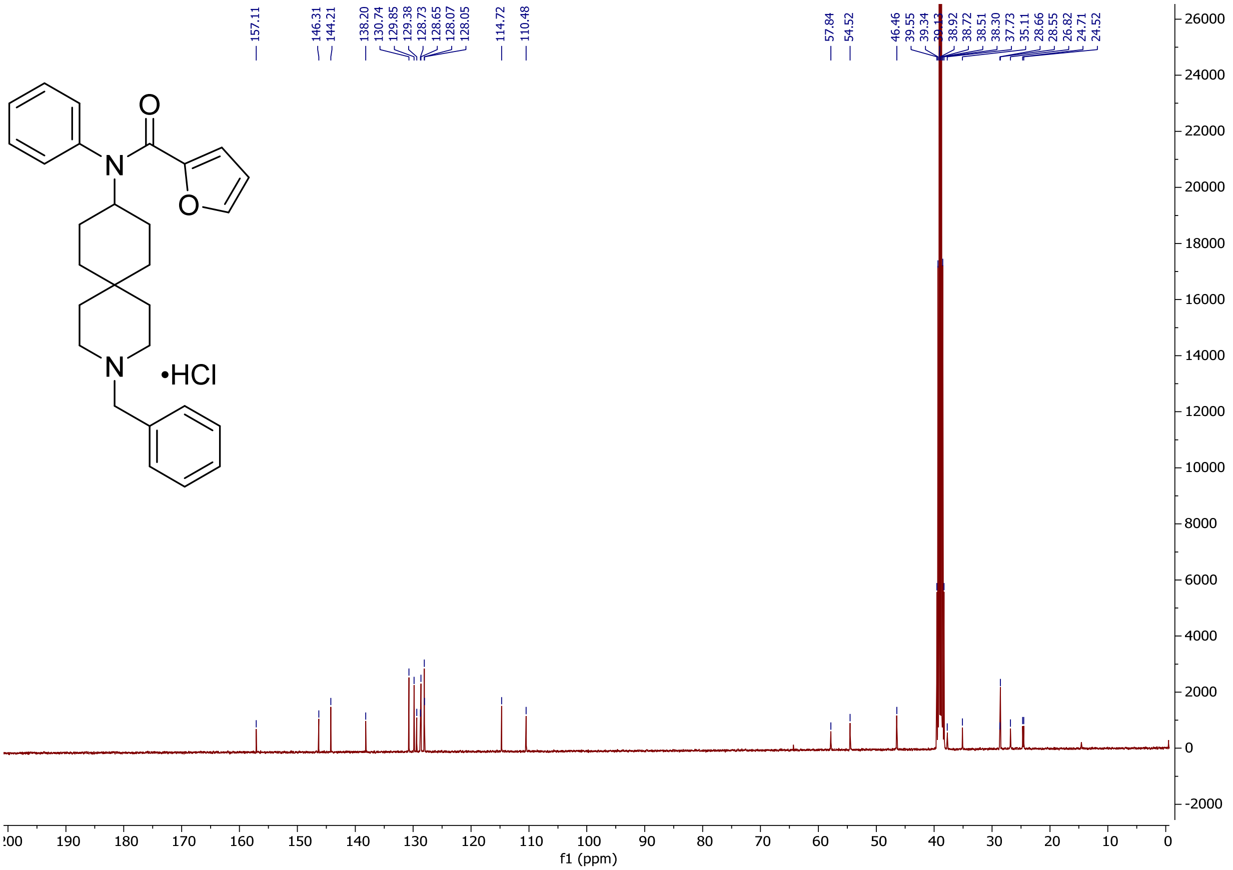

^{13}C NMR (100 MHz, DMSO-d_6) spectrum for compound **42**.

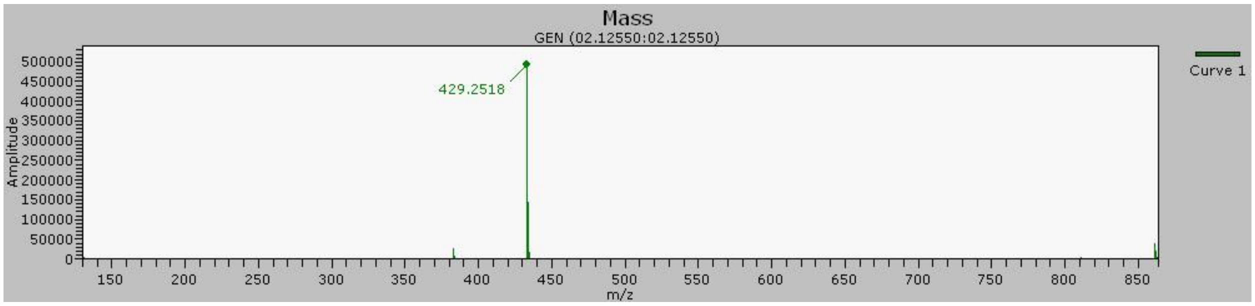

HRMS spectrum for compound **42**.

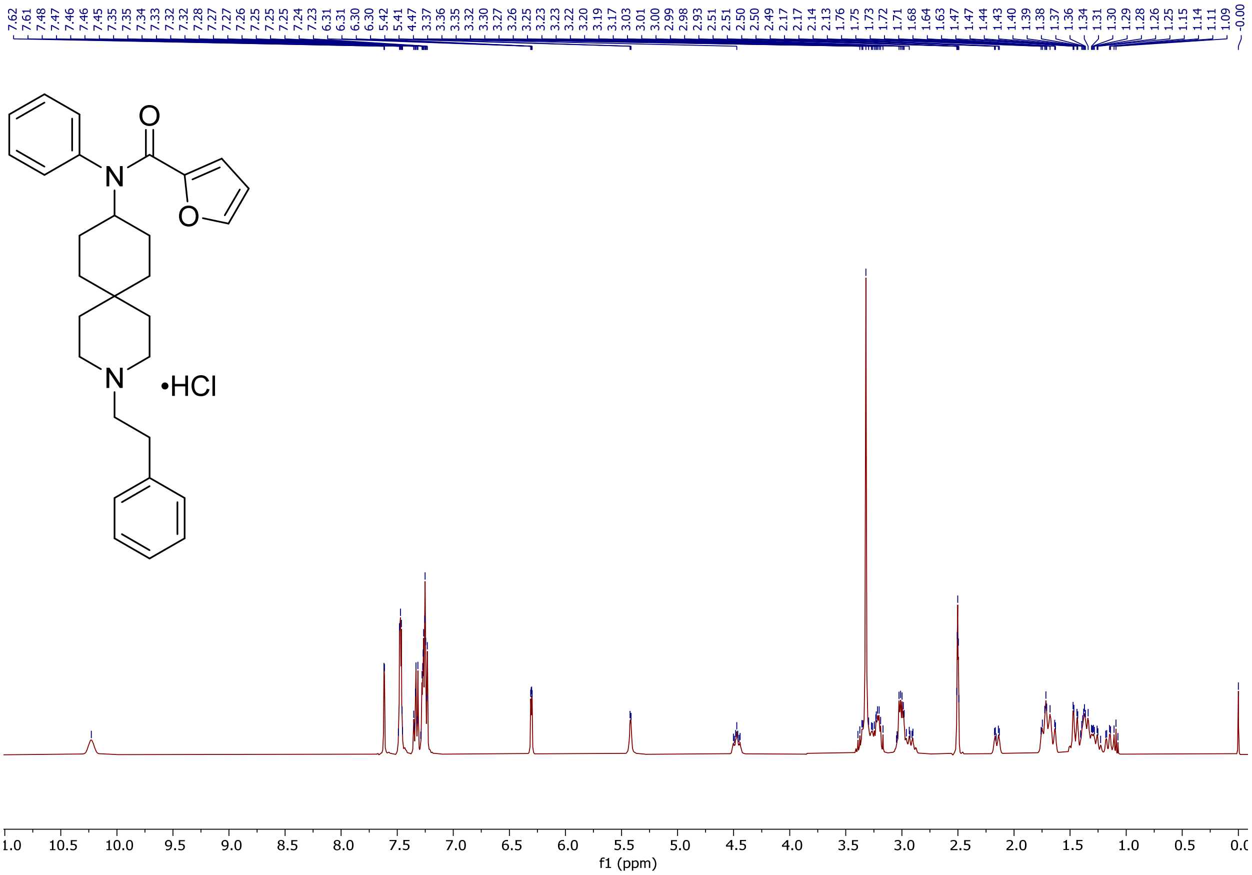

^{1}H NMR (400 MHz, DMSO-d_6) spectrum for compound **43**.

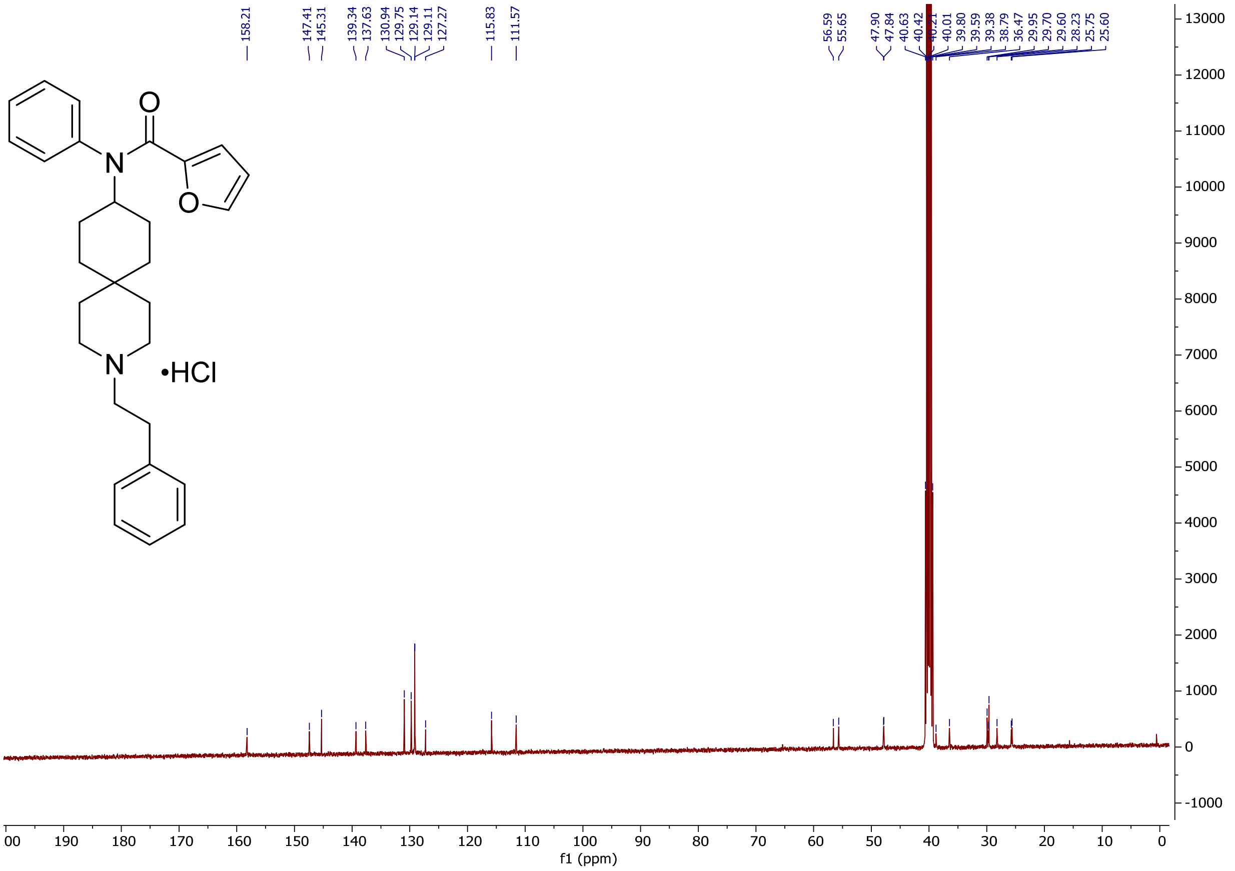

^{13}C NMR (100 MHz, DMSO-d_6) spectrum for compound **43**.

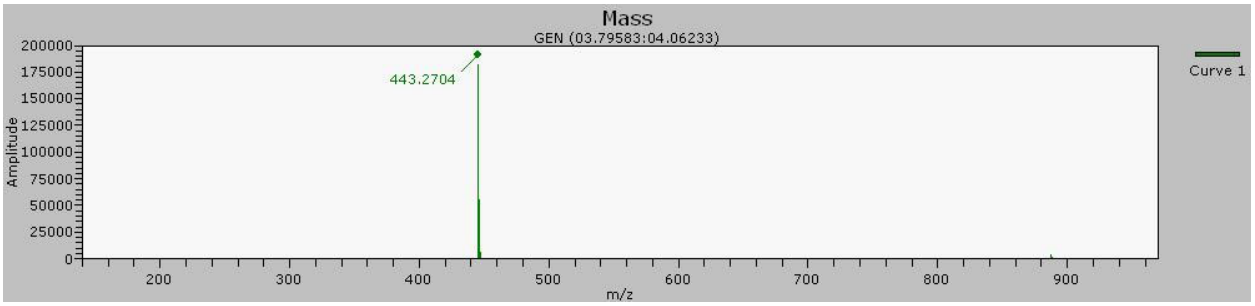

HRMS spectrum for compound **43**.

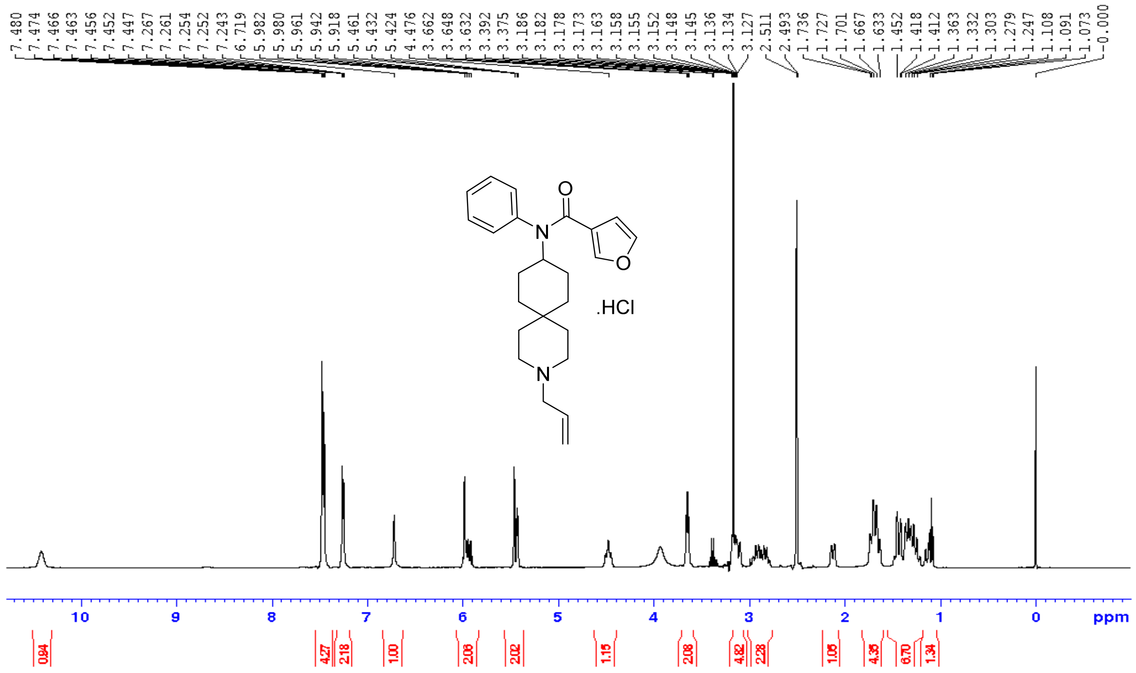

^{1}H NMR (400 MHz, DMSO-*d*6) spectrum for compound **44**.

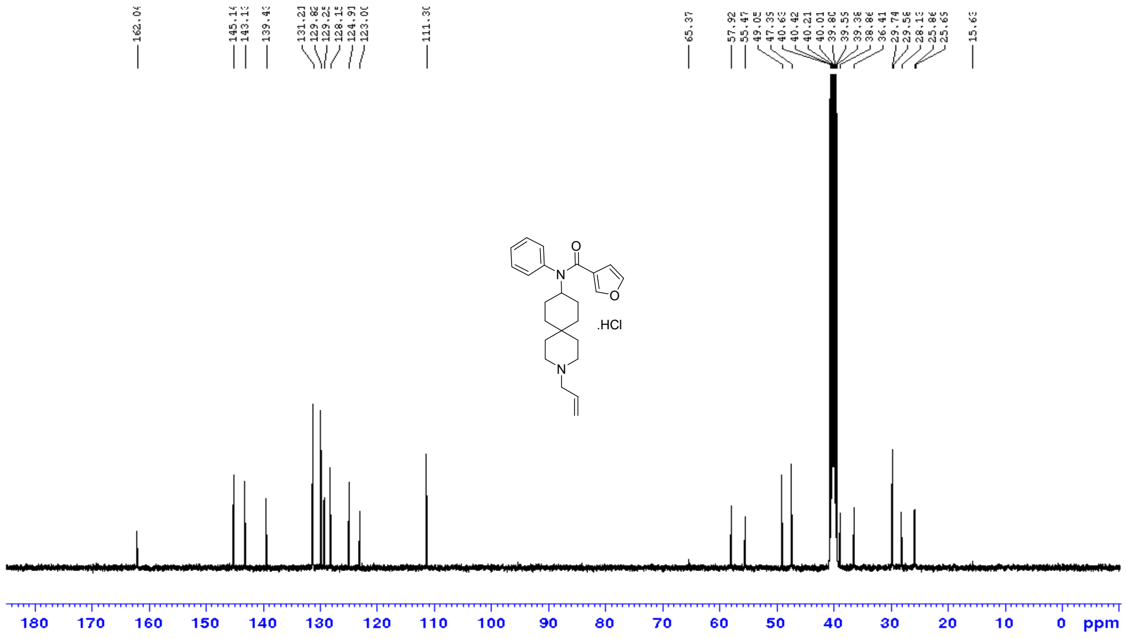

^{13}C NMR (100 MHz, DMSO-*d*6) spectrum for compound **44**.

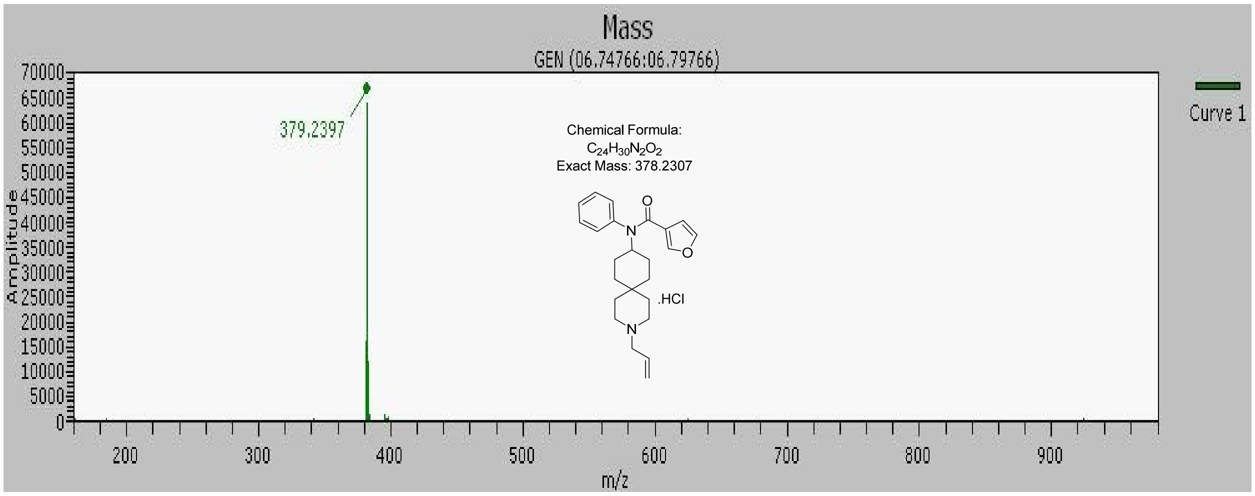

HRMS spectrum for compound **44**.

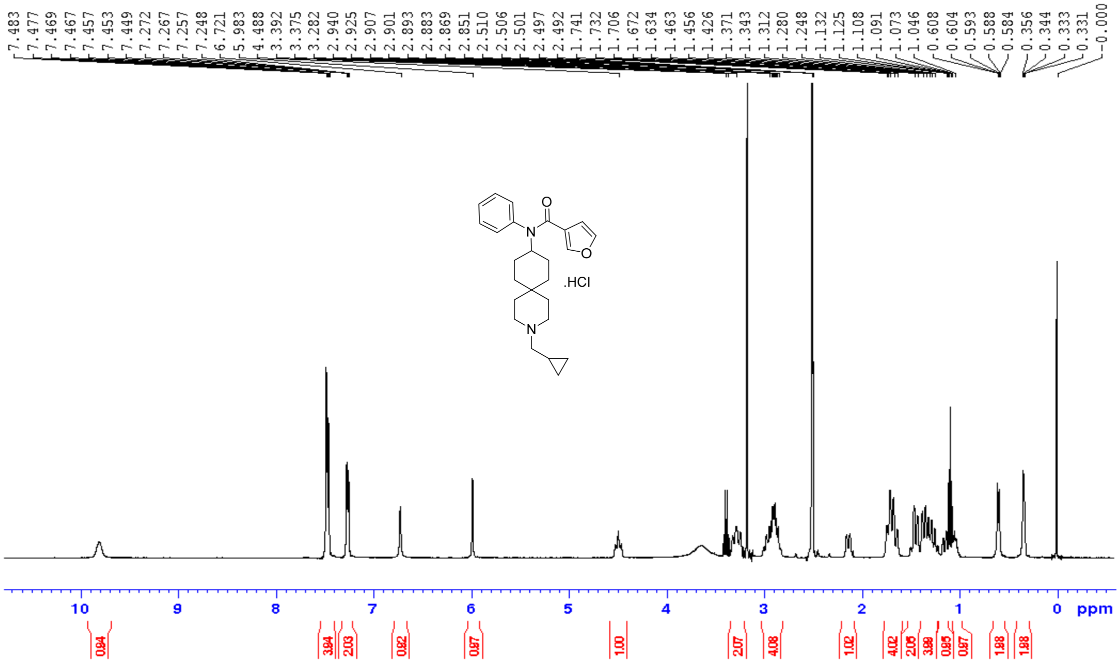

^{1}H NMR (400 MHz, DMSO-d_6) spectrum for compound **45**.

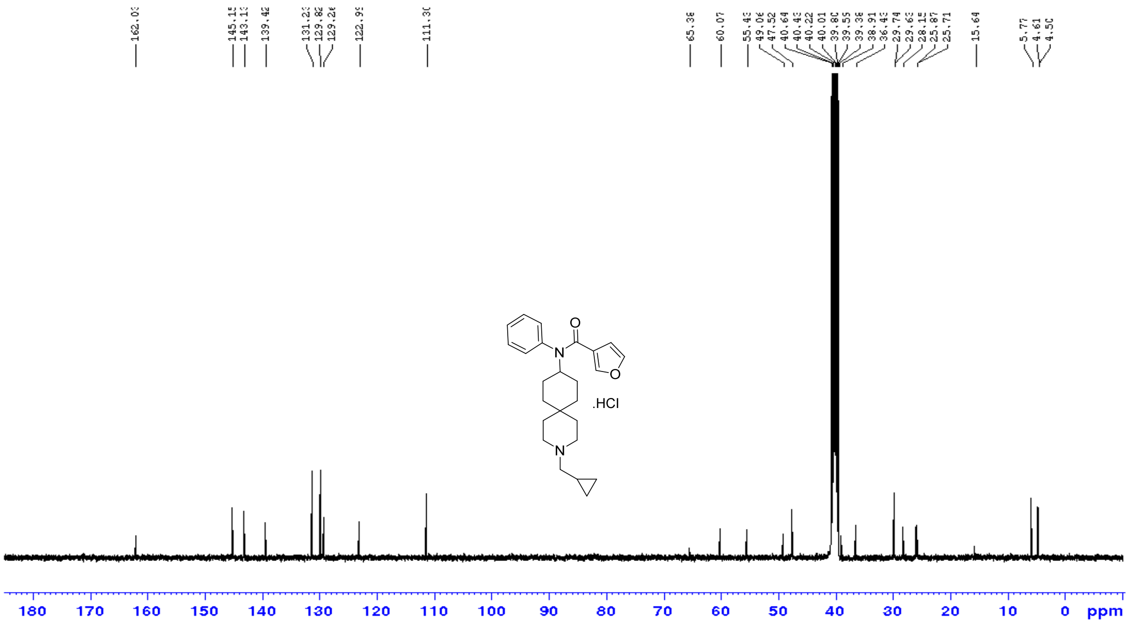

^{13}C NMR (100 MHz, DMSO-d_6) spectrum for compound **45**.

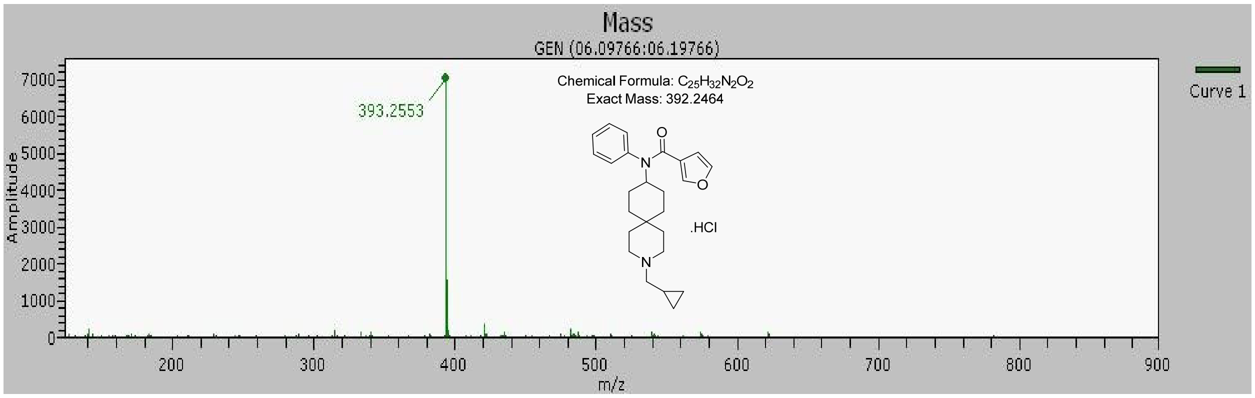

HRMS spectrum for compound **45**.

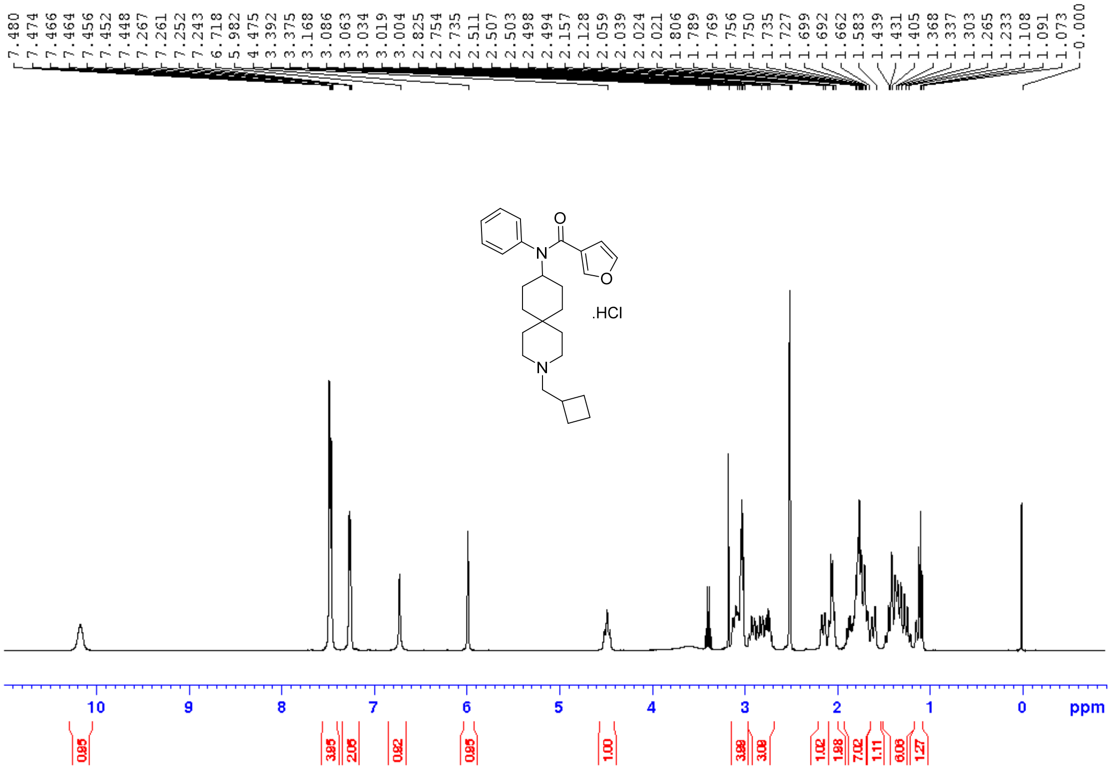

^{1}H NMR (400 MHz, DMSO-d_6) spectrum for compound **46**.

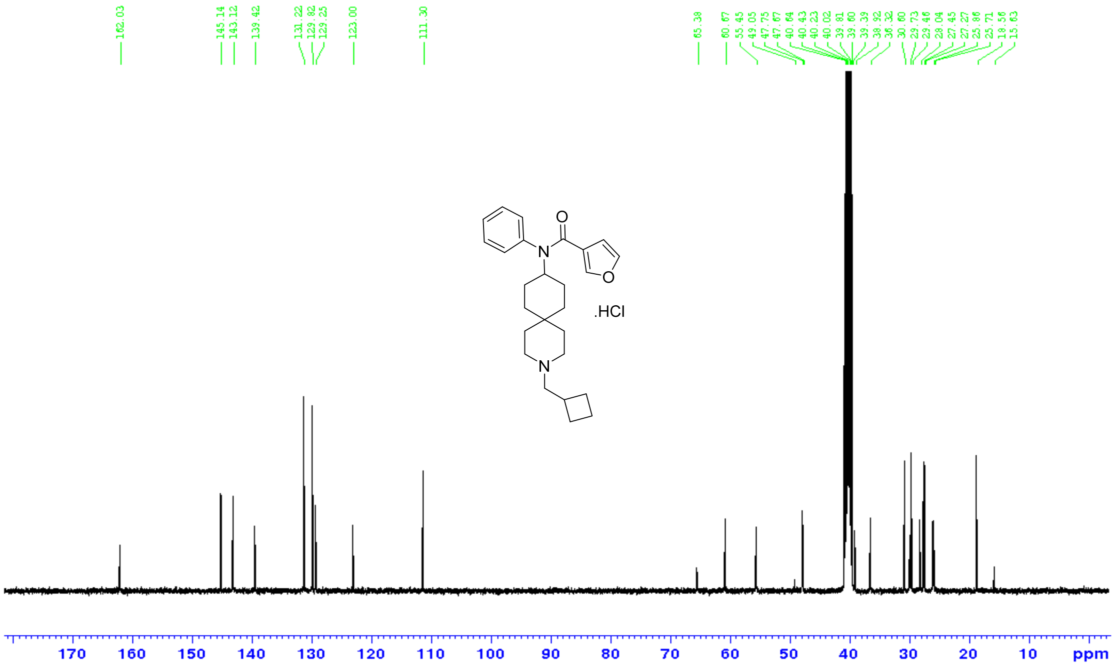

^{13}C NMR (100 MHz, DMSO-d_6) spectrum for compound **46**.

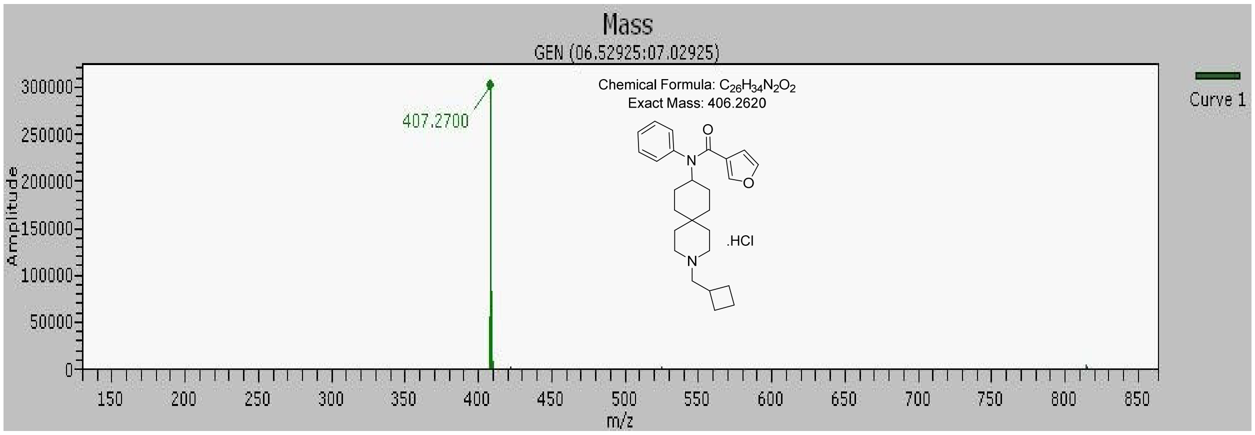

HRMS spectrum for compound **46**.

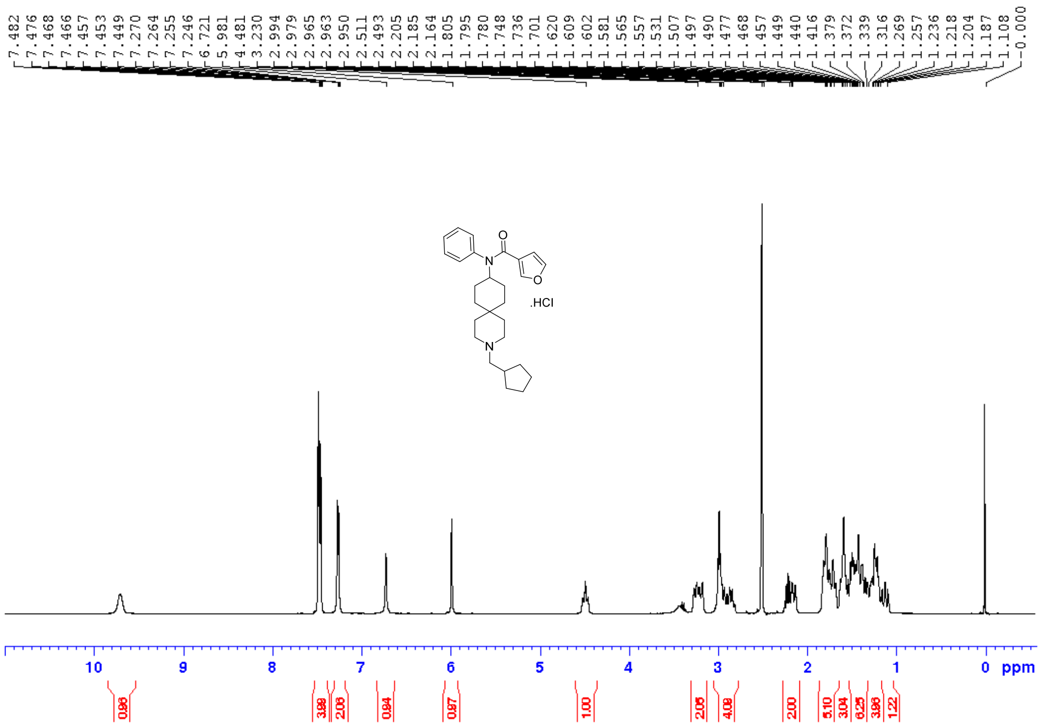

^{1}H NMR (400 MHz, DMSO-d_6) spectrum for compound **47**.

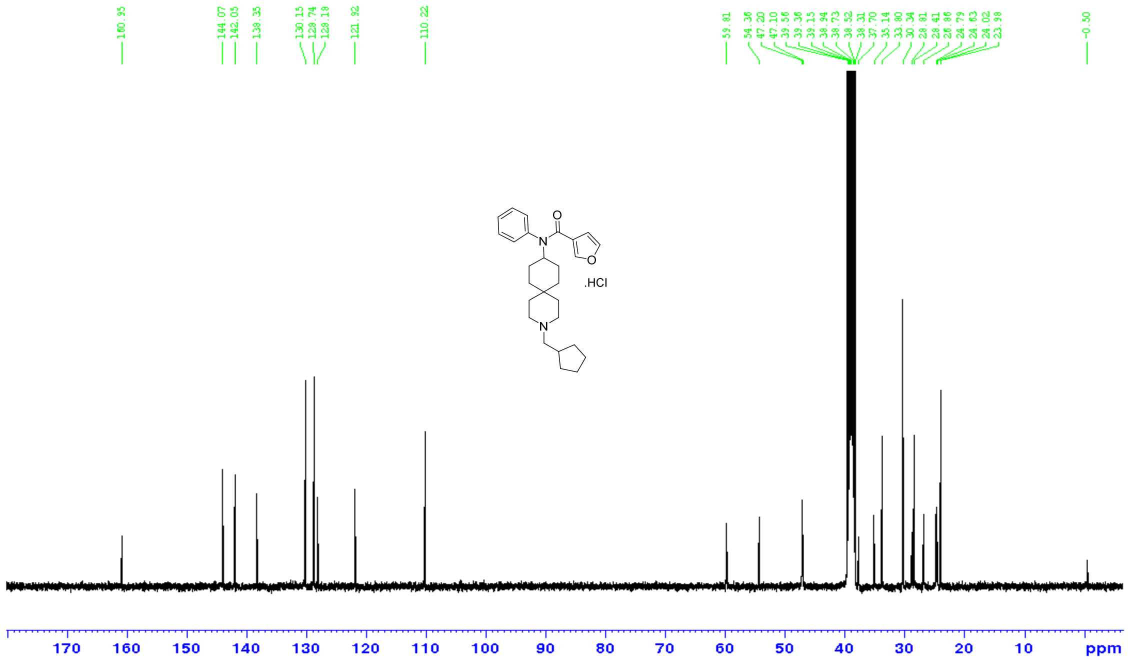

^{13}C NMR (100 MHz, DMSO-d_6) spectrum for compound **47**.

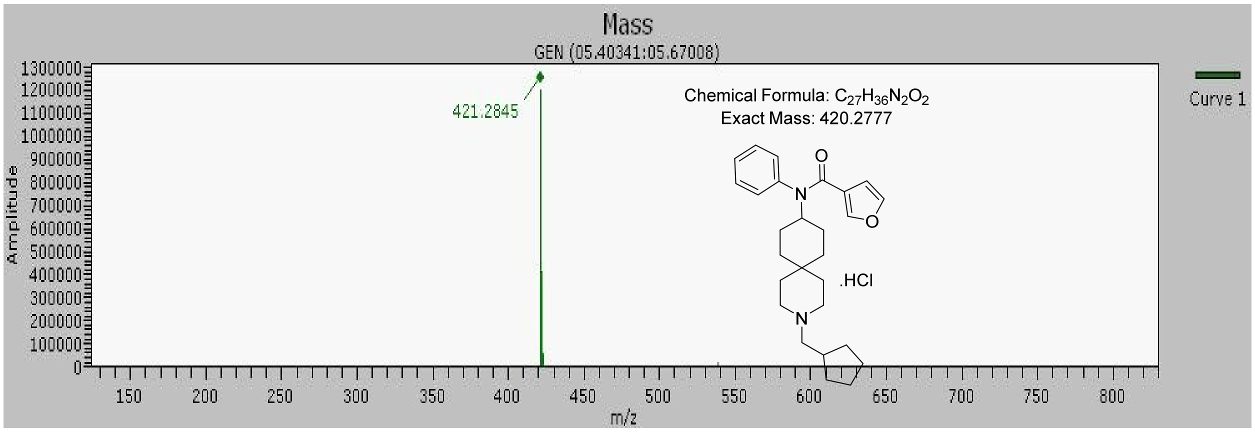

HRMS spectrum for compound **47**.

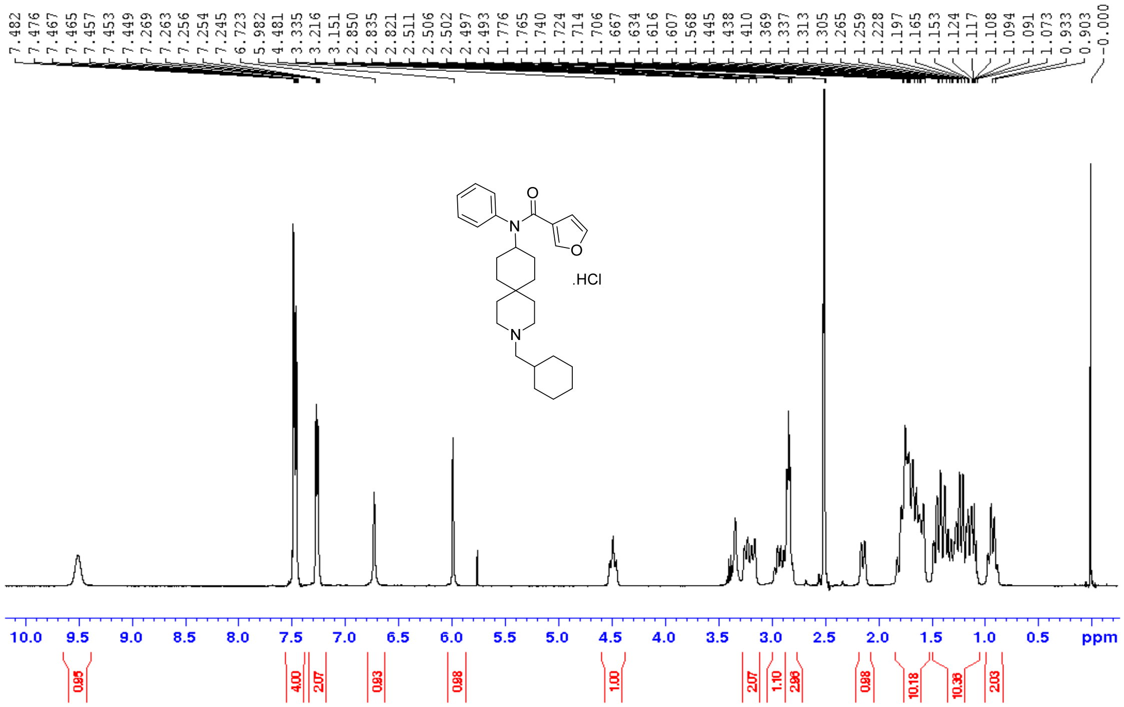

^{1}H NMR (400 MHz, DMSO-d_6) spectrum for compound **48**.

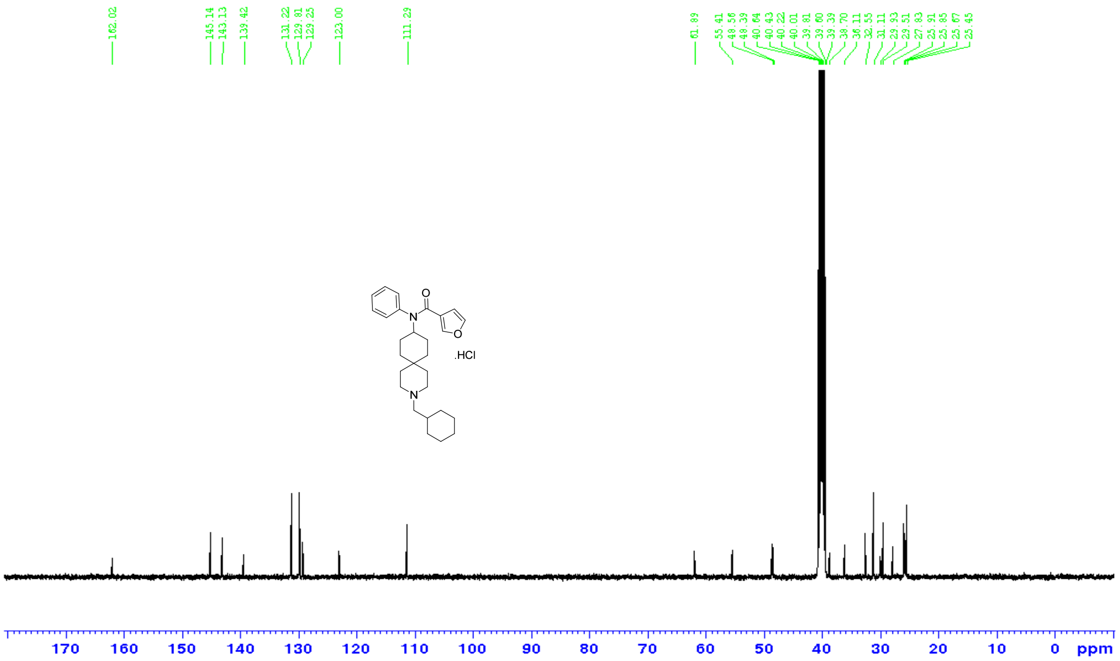

^{13}C NMR (100 MHz, DMSO-*d*$_6$) spectrum for compound **48**.

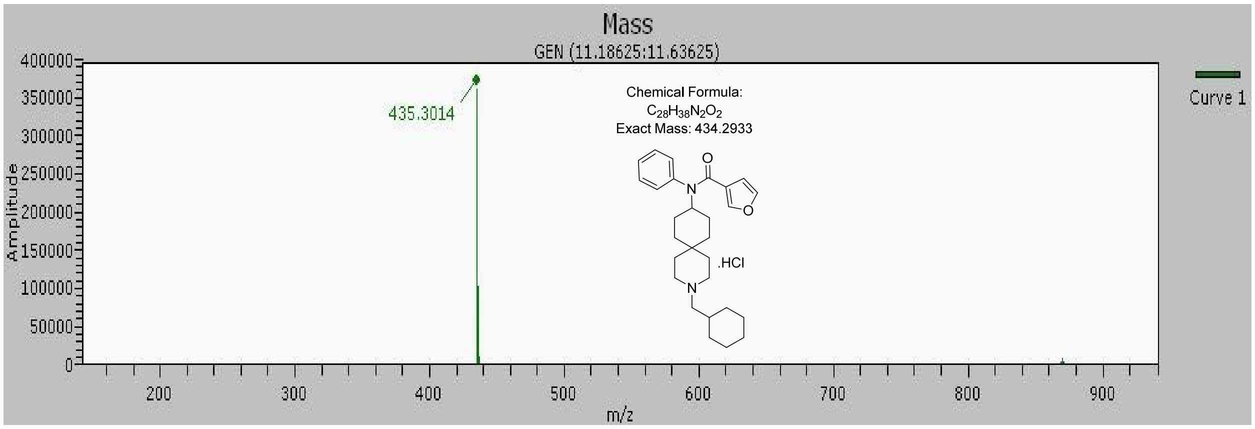

HRMS spectrum for compound **48**.

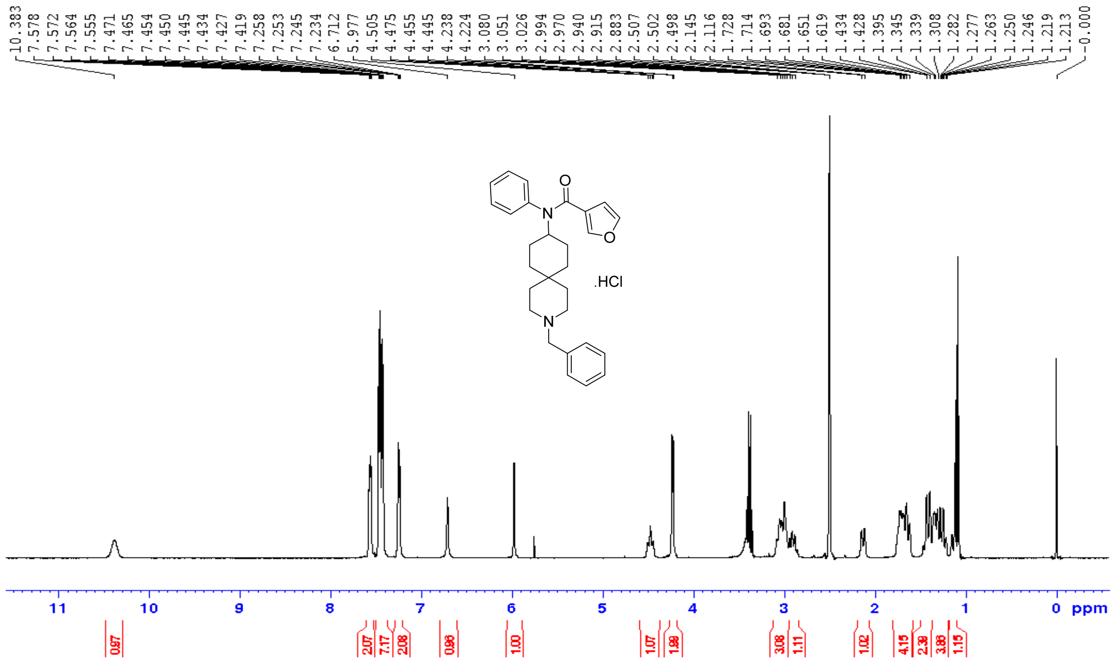

^{1}H NMR (400 MHz, DMSO-*d*6) spectrum for compound **49**.

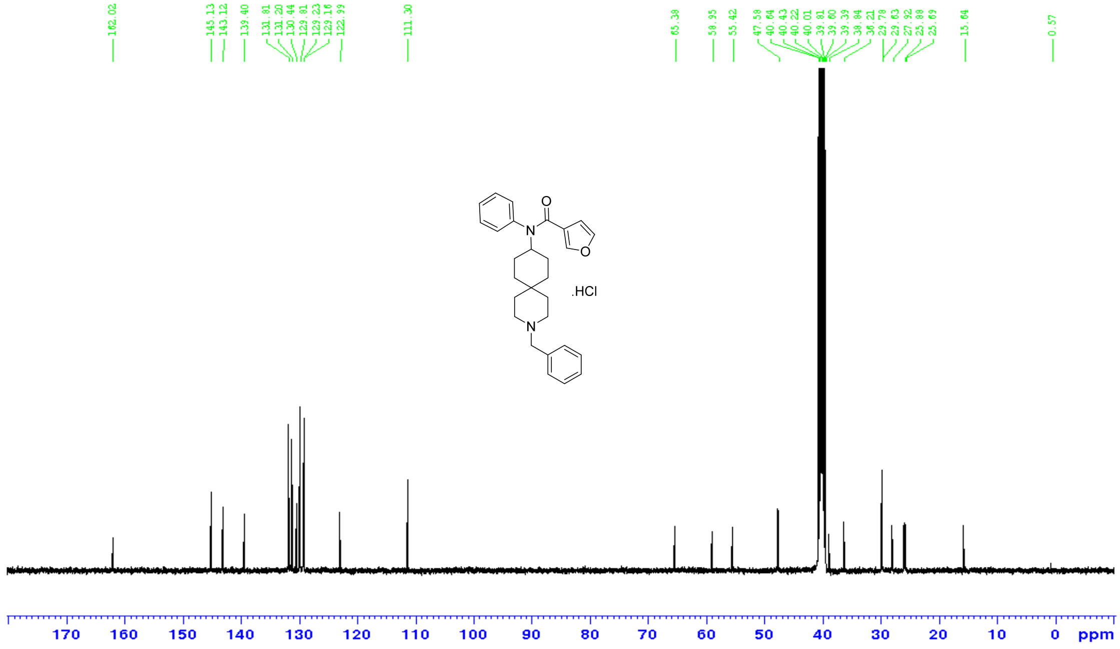

^{13}C NMR (100 MHz, DMSO-*d*6) spectrum for compound **49**.

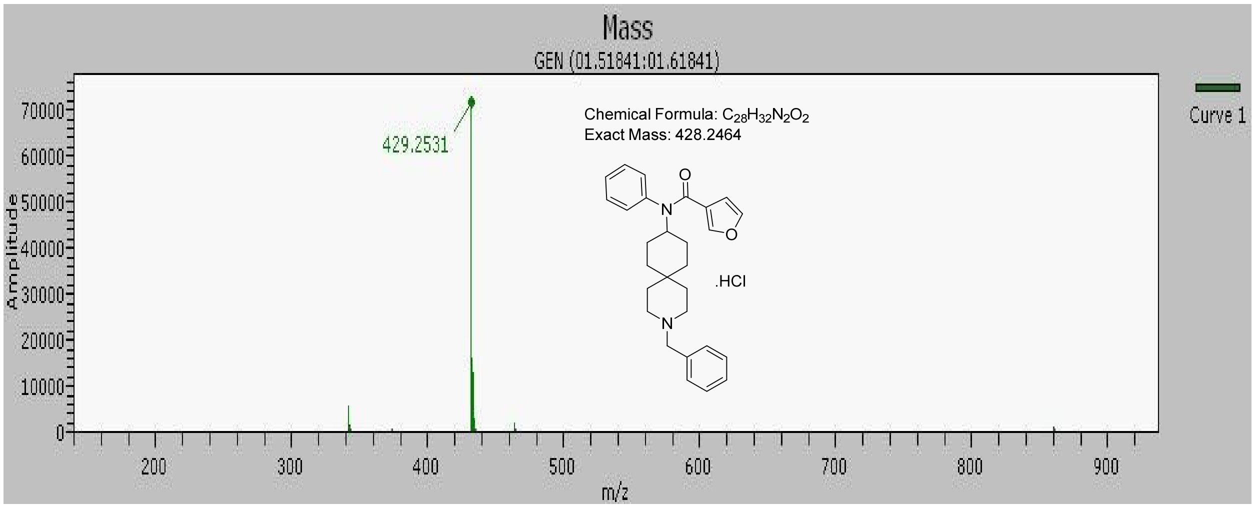

HRMS spectrum for compound **49**.

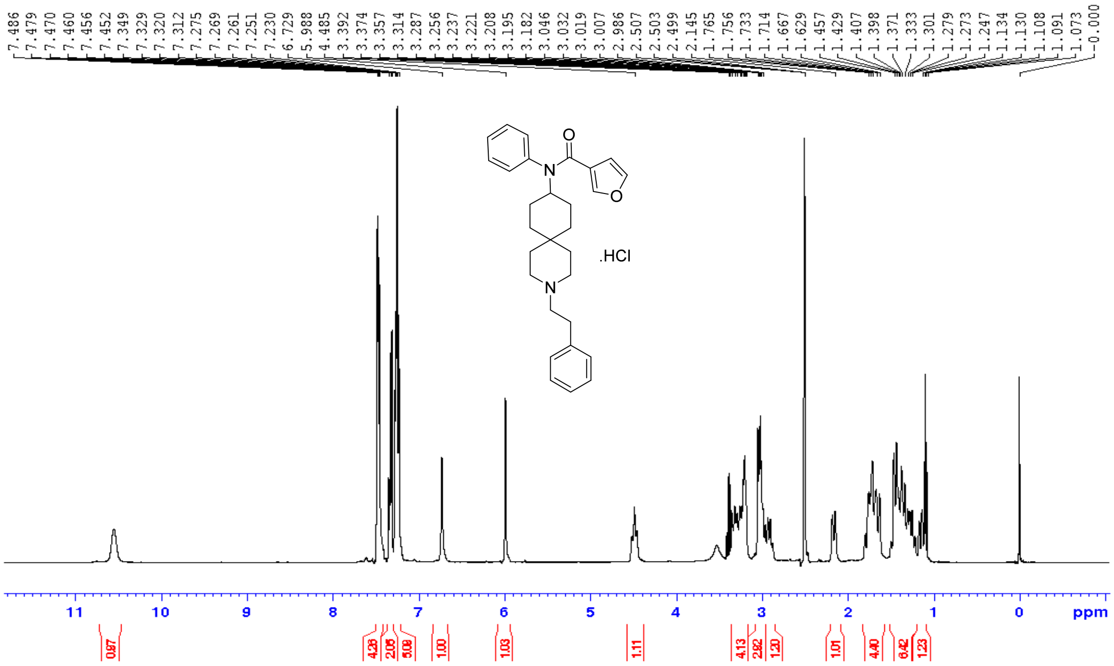

^{1}H NMR (400 MHz, DMSO-d_6) spectrum for compound **50**.

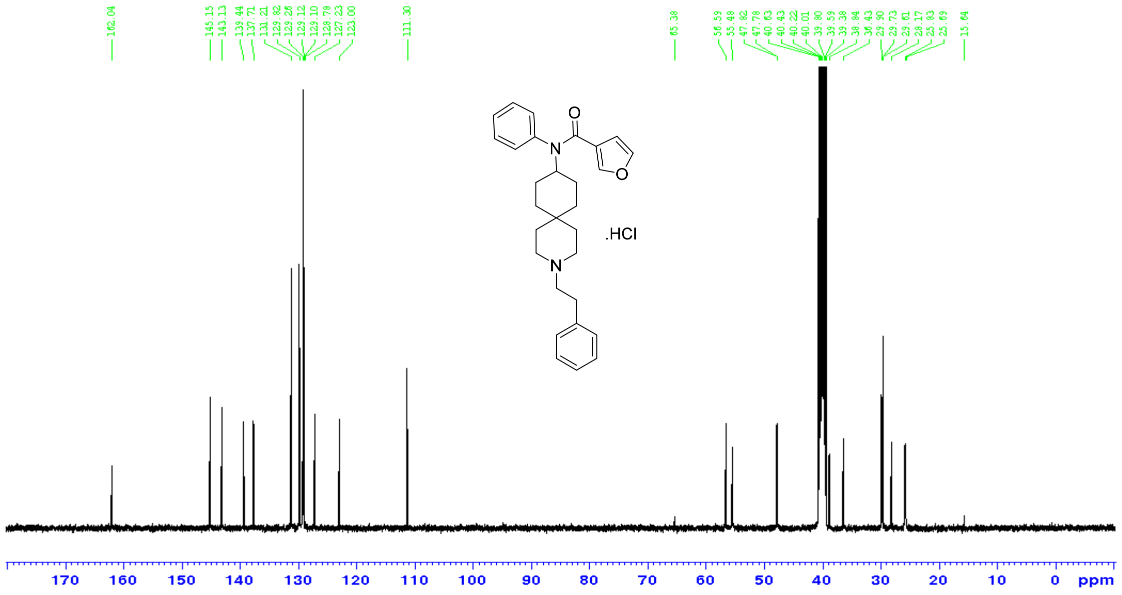

^{13}C NMR (100 MHz, DMSO-d_6) spectrum for compound **50**.

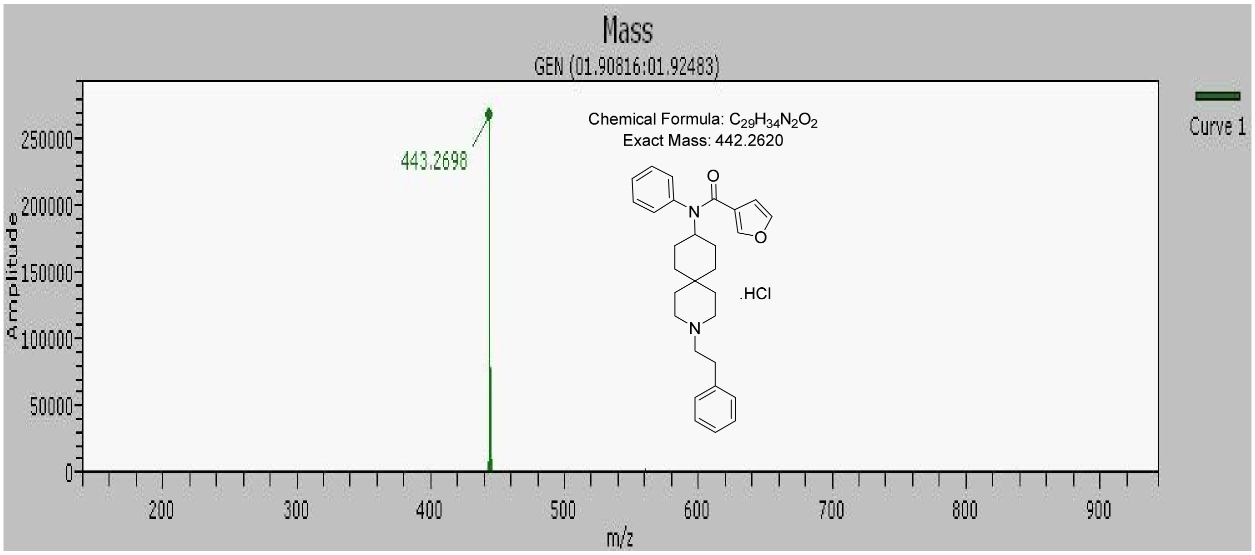

HRMS spectrum for compound **50**.

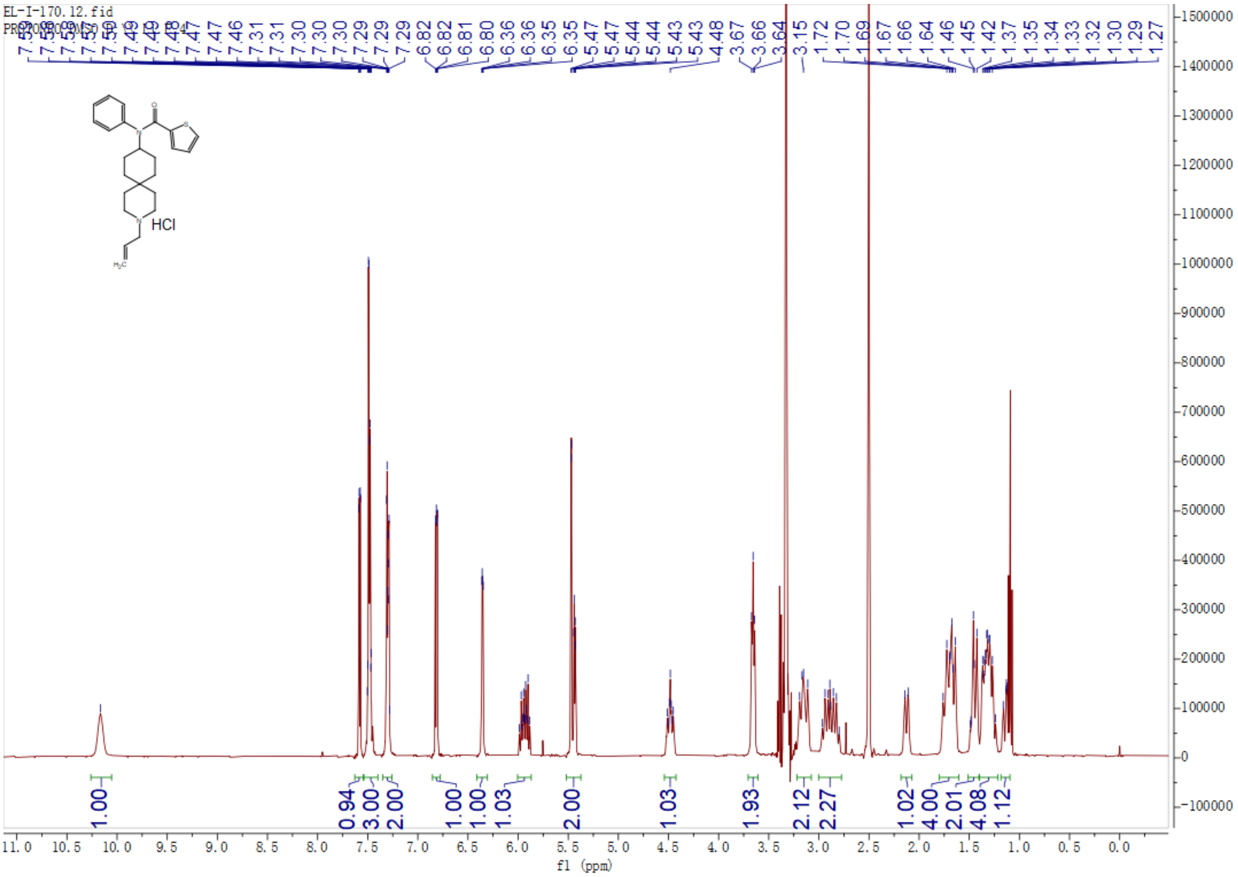

^{1}H NMR (400 MHz, DMSO-d_6) spectrum for compound **51**.

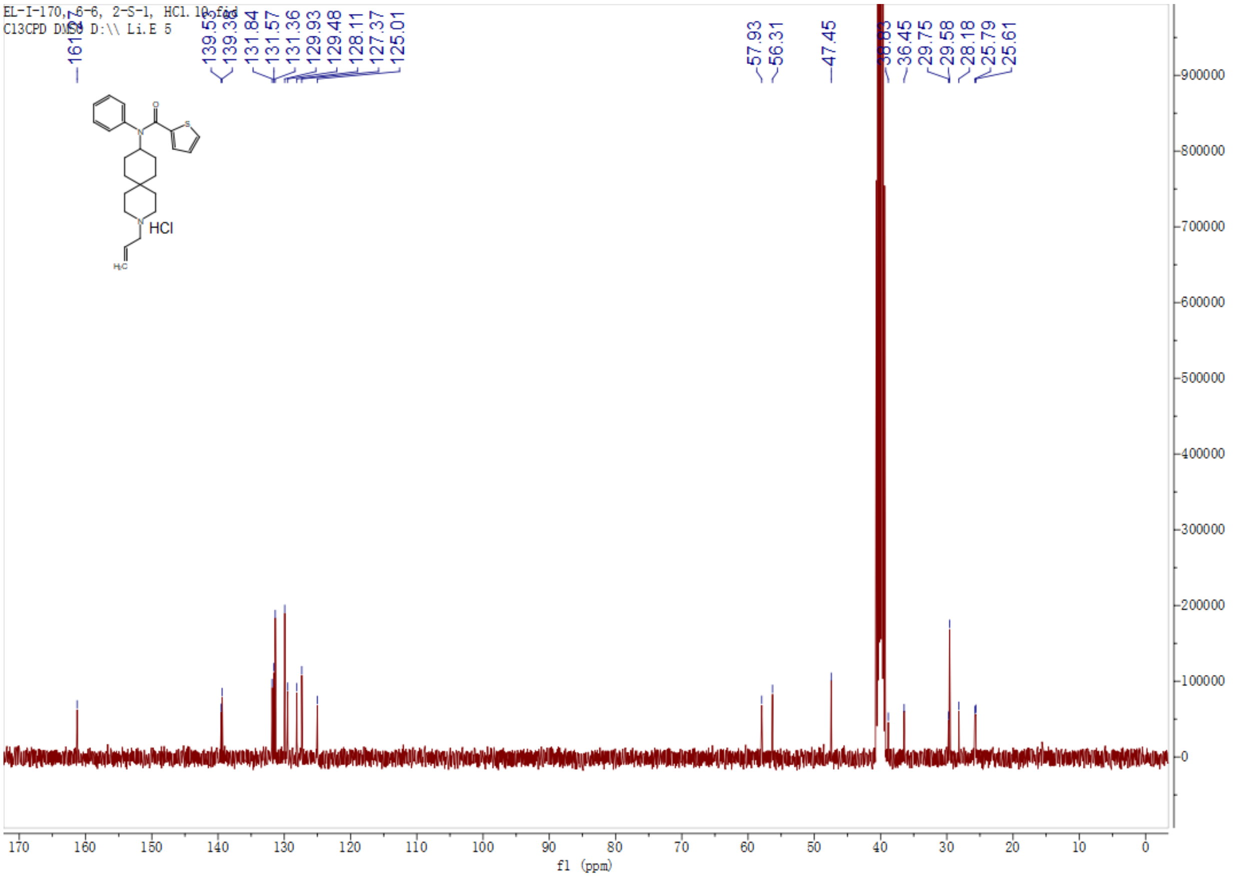

^{13}C NMR (100 MHz, DMSO-d_6) spectrum for compound **51**.

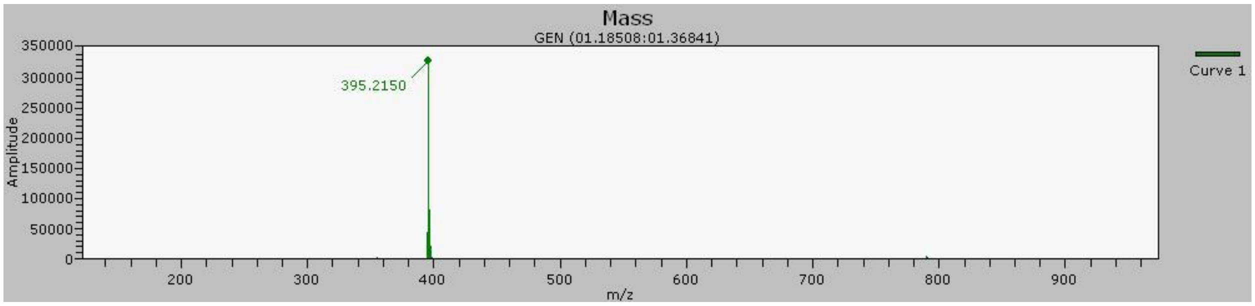

HRMS spectrum for compound **51**.

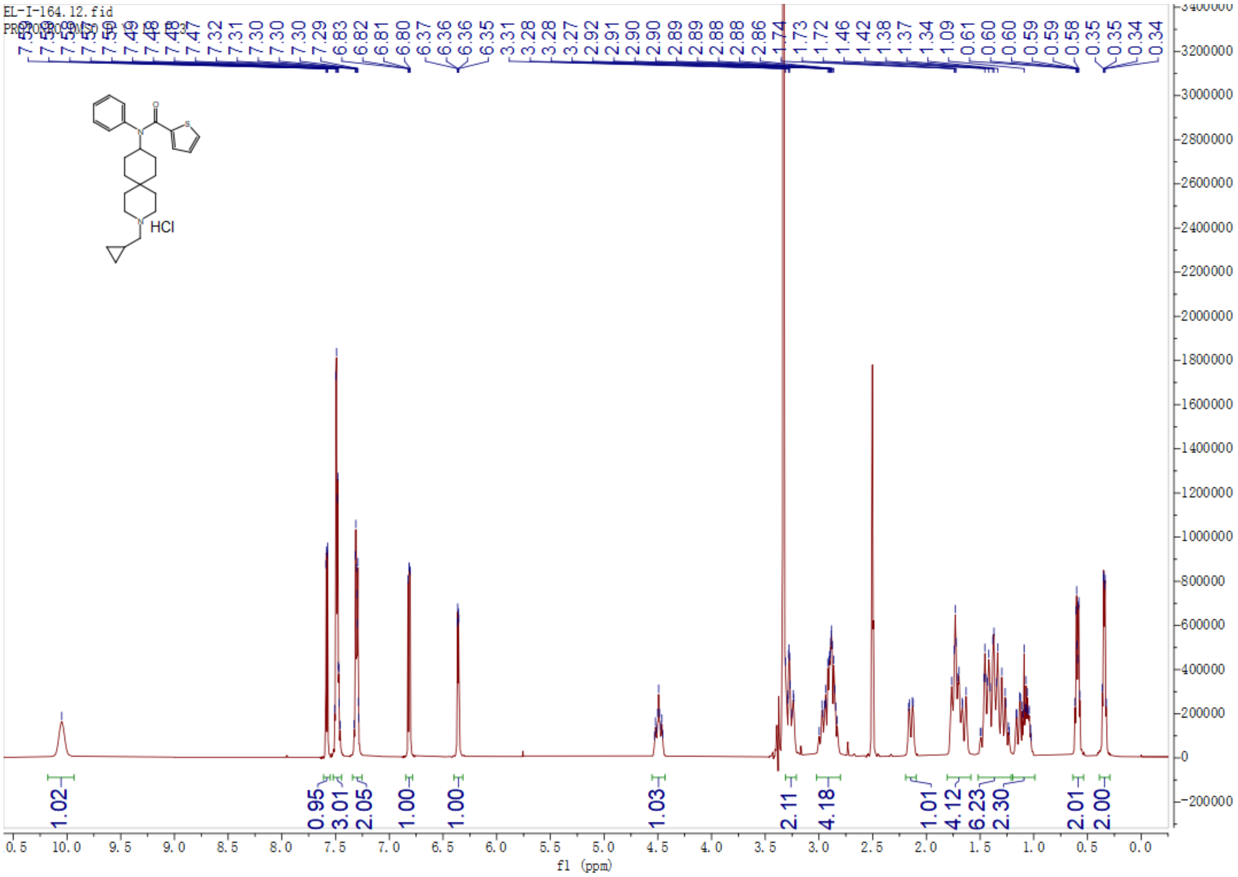

^{1}H NMR (400 MHz, DMSO-d_6) spectrum for compound **52**.

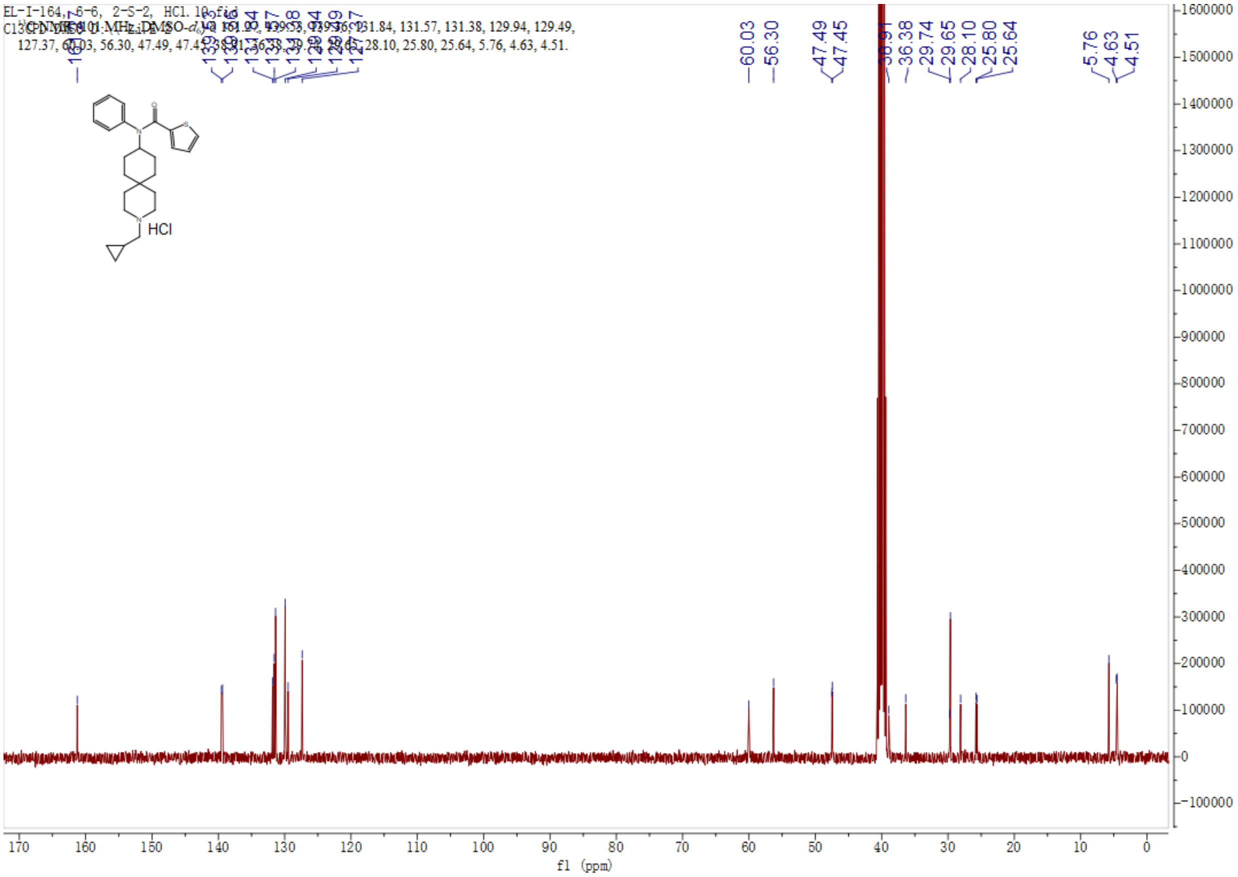

^{13}C NMR (100 MHz, DMSO-d_6) spectrum for compound **52**.

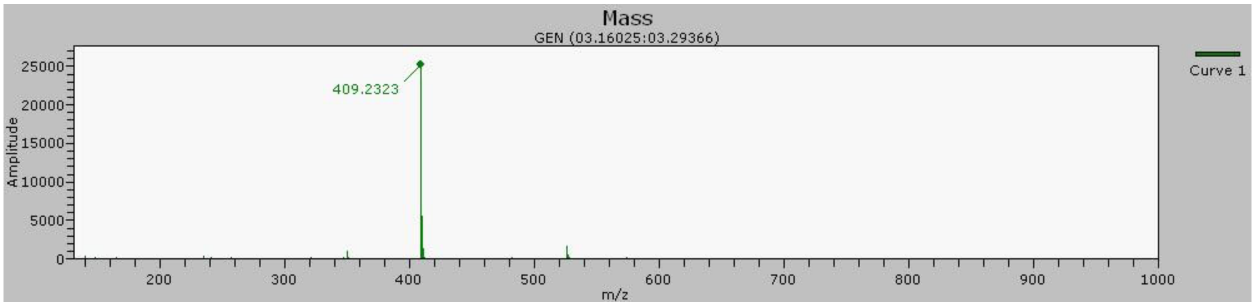

HRMS spectrum for compound **52**.

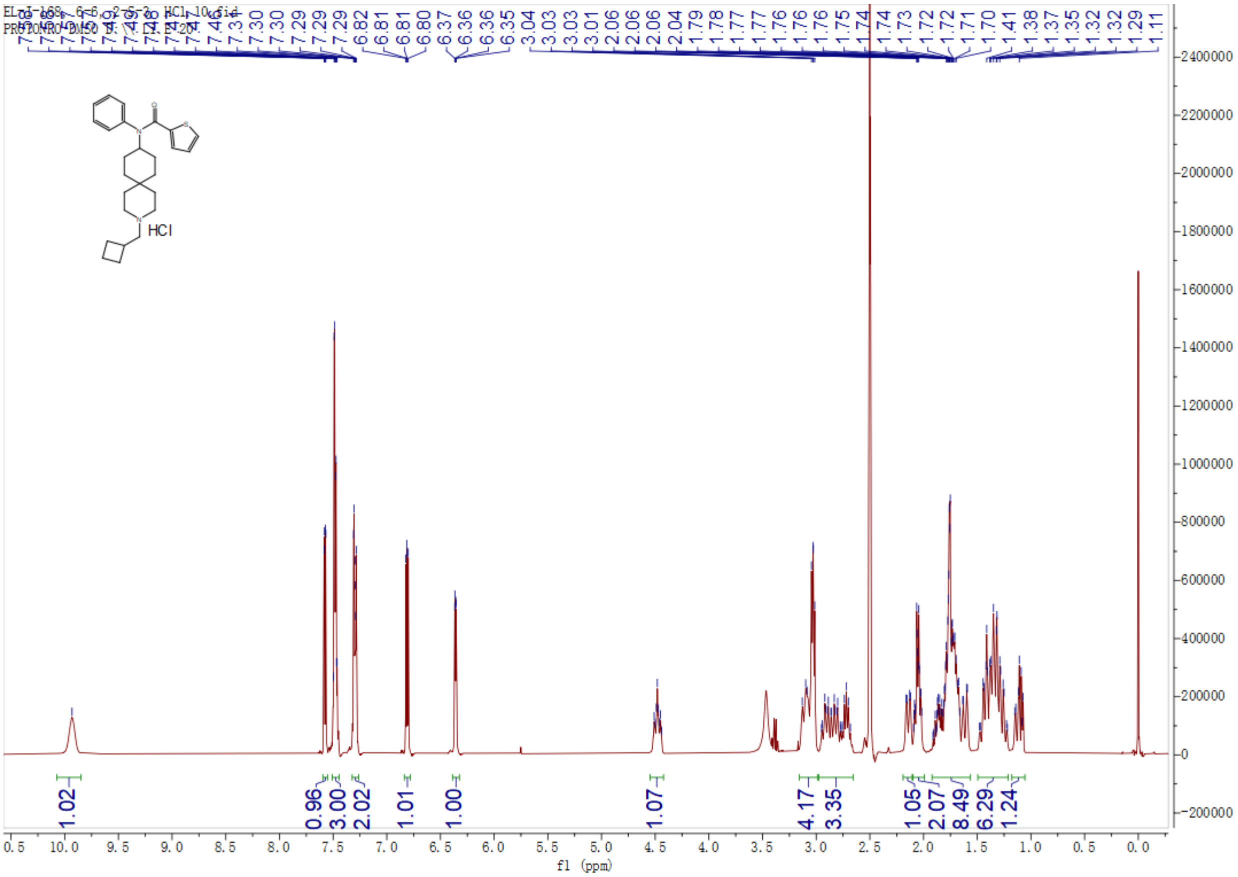

^{1}H NMR (400 MHz, DMSO-d_6) spectrum for compound **53**.

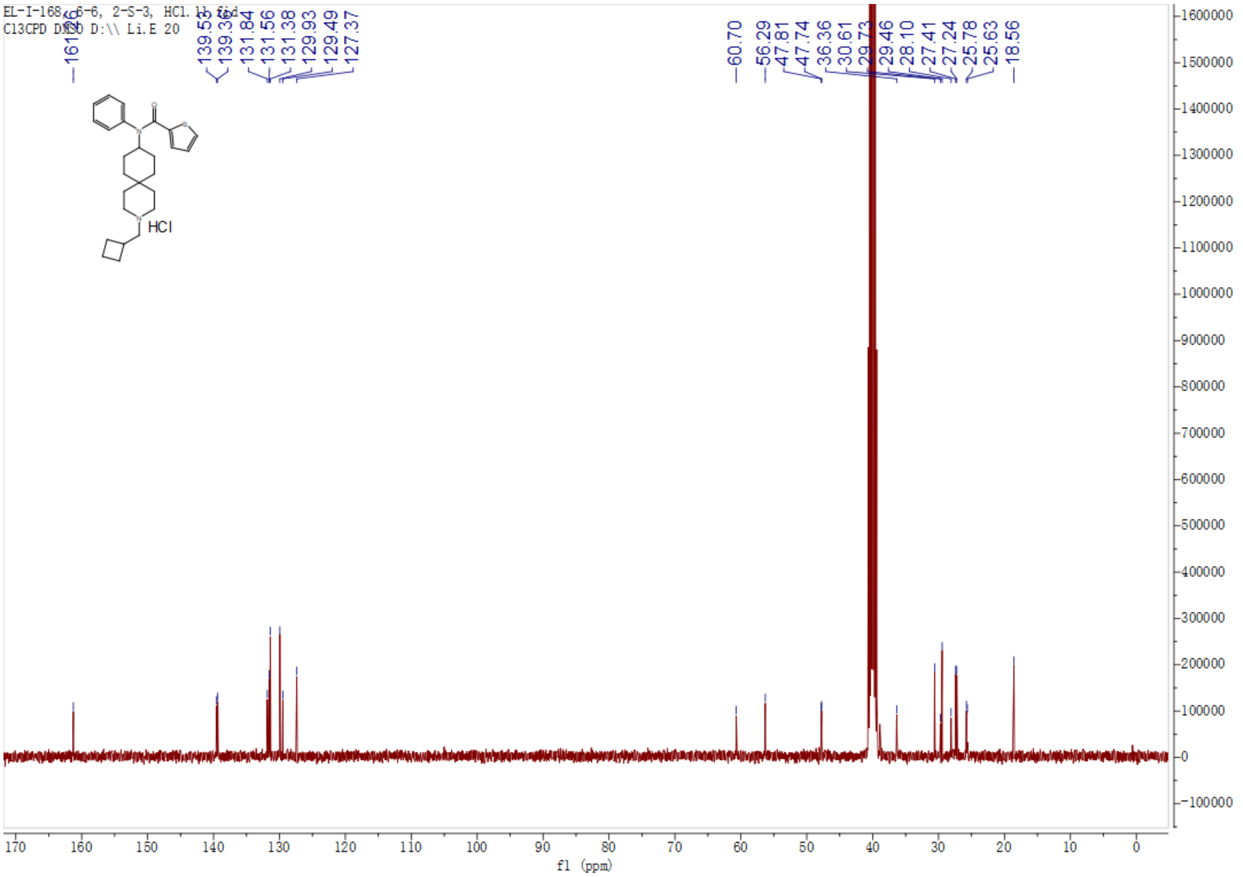

^{13}C NMR (100 MHz, DMSO-d_6) spectrum for compound **53**.

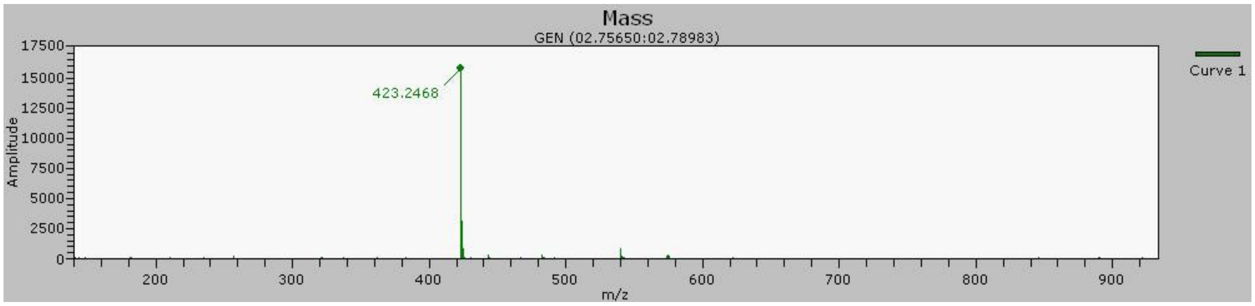

HRMS spectrum for compound **53**.

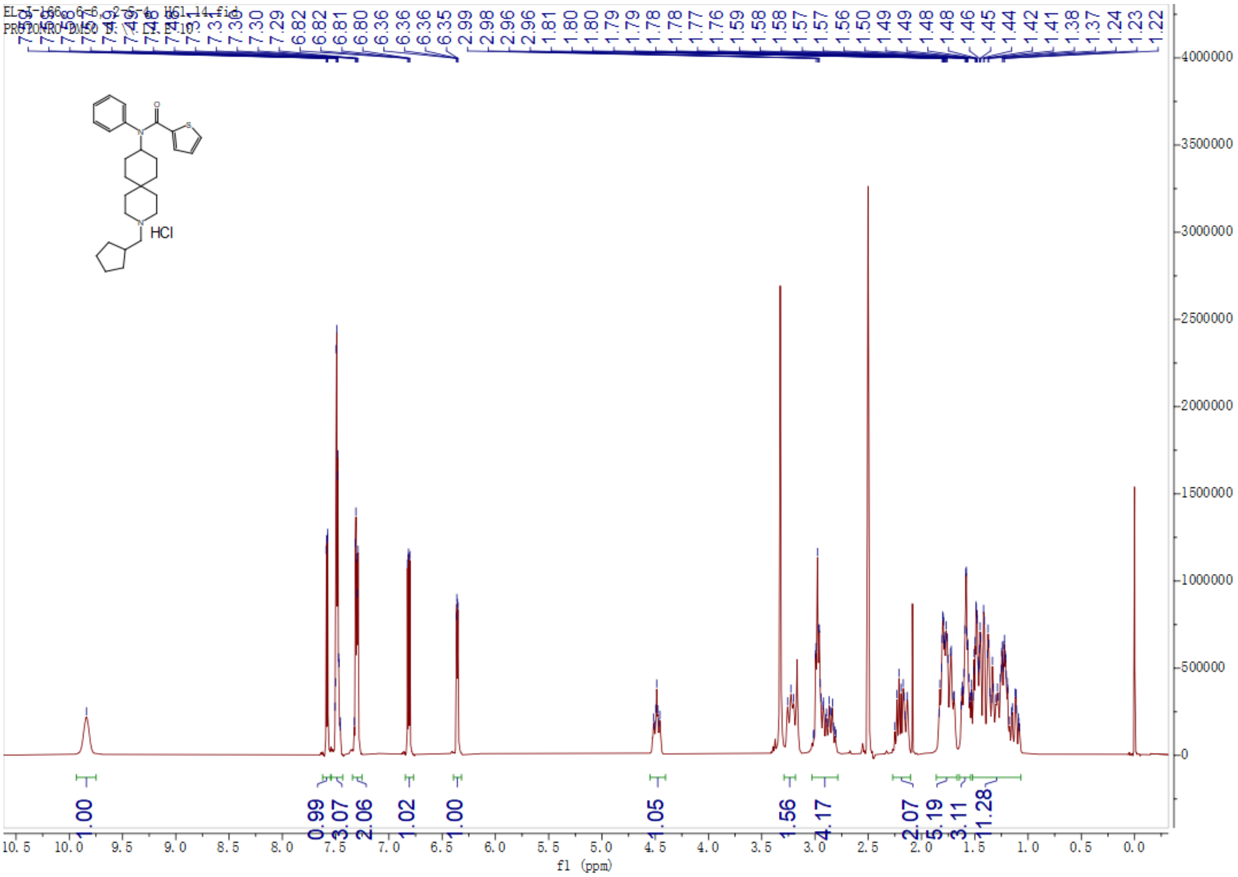

^{1}H NMR (400 MHz, DMSO-d_6) spectrum for compound **54**.

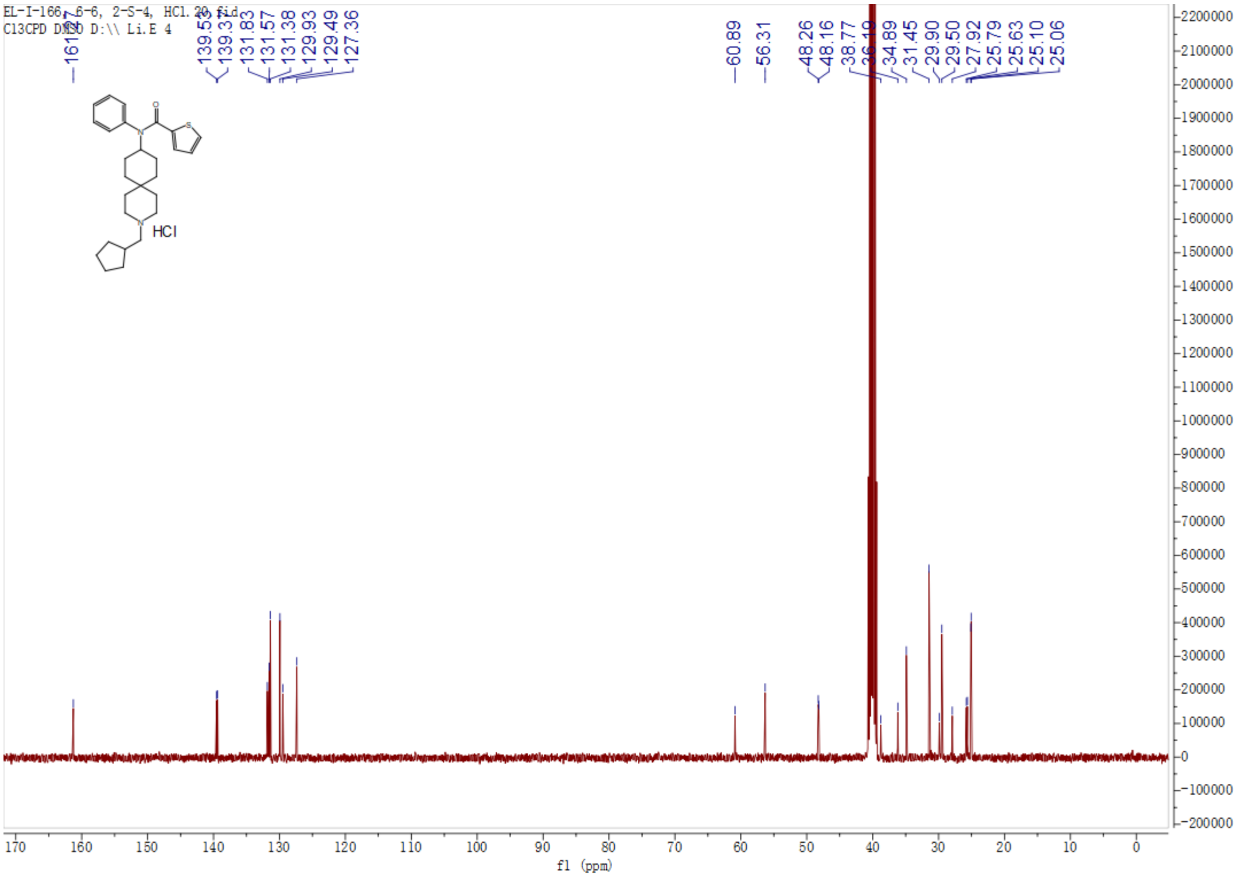

^{13}C NMR (100 MHz, DMSO-d_6) spectrum for compound **54**.

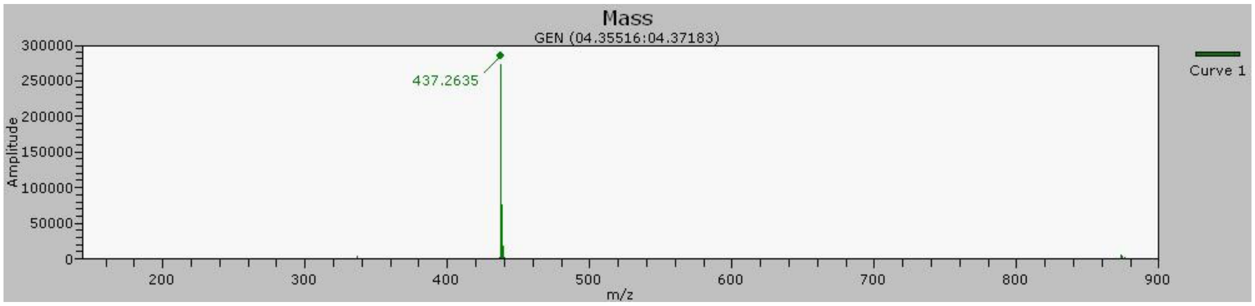

HRMS spectrum for compound **54**.

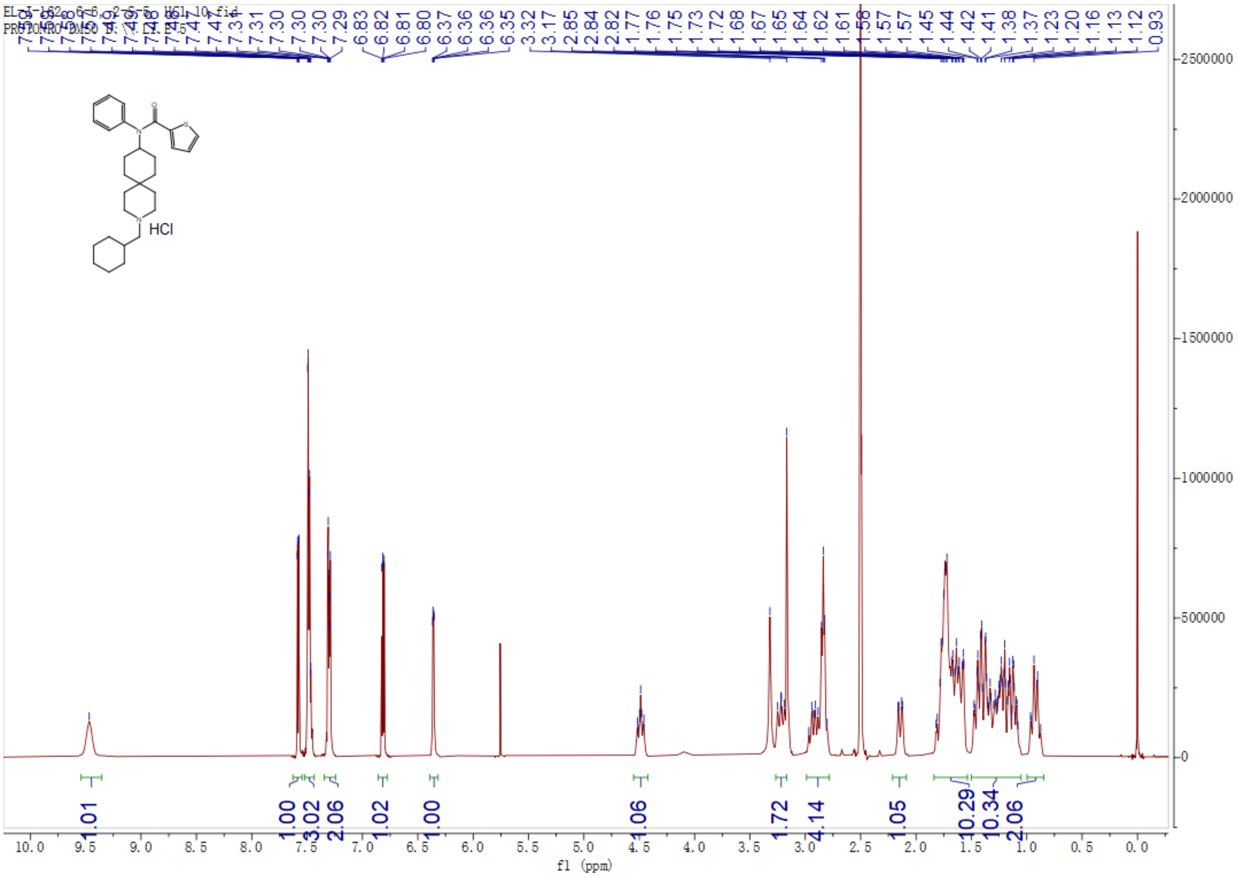

^{1}H NMR (400 MHz, DMSO-d_6) spectrum for compound **55**.

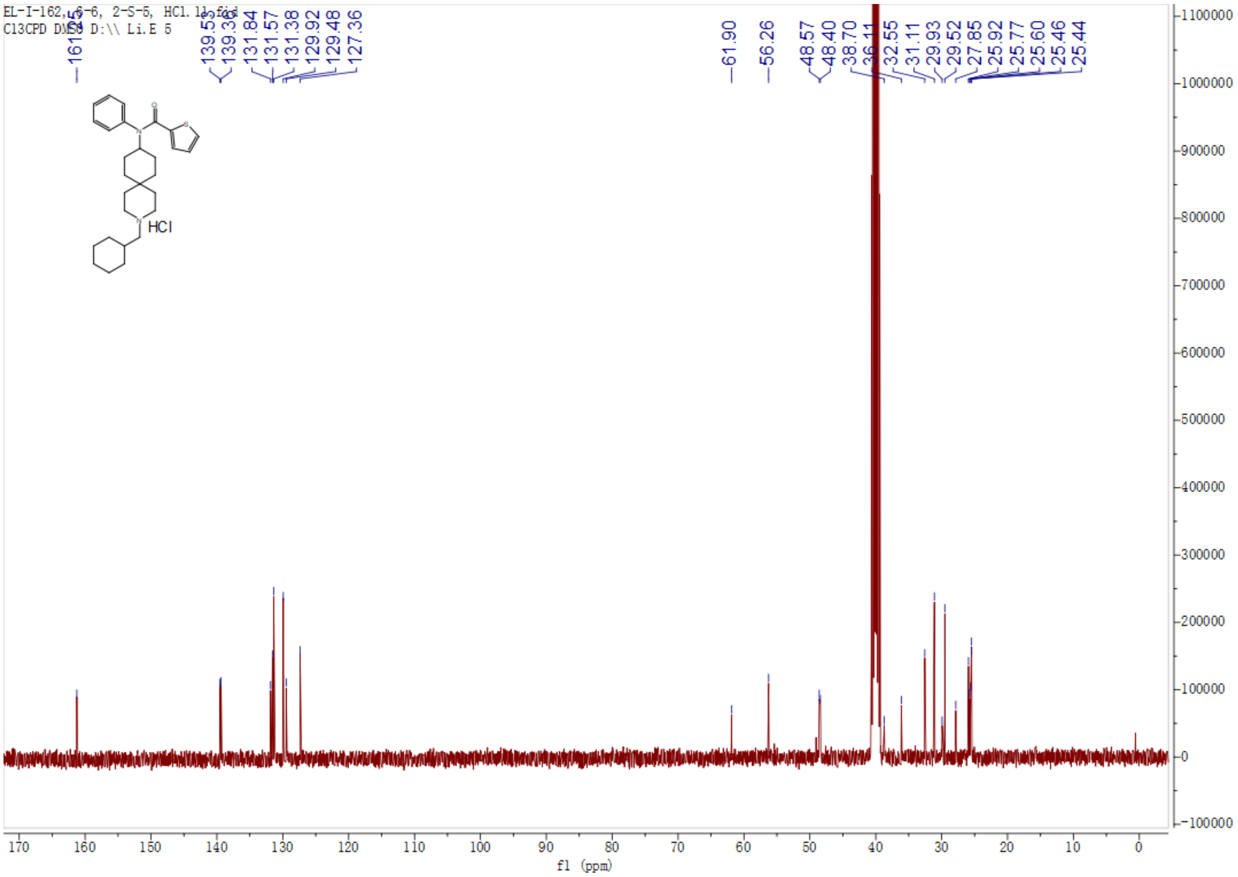

^{13}C NMR (100 MHz, DMSO-d_6) spectrum for compound **55**.

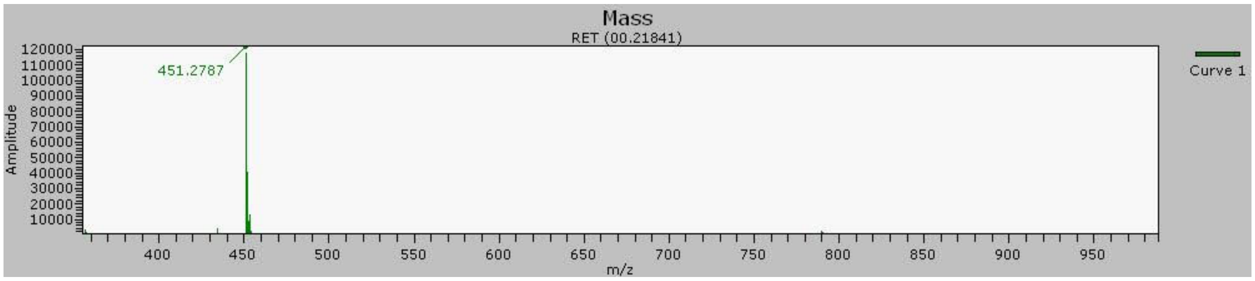

HRMS spectrum for compound **55**.

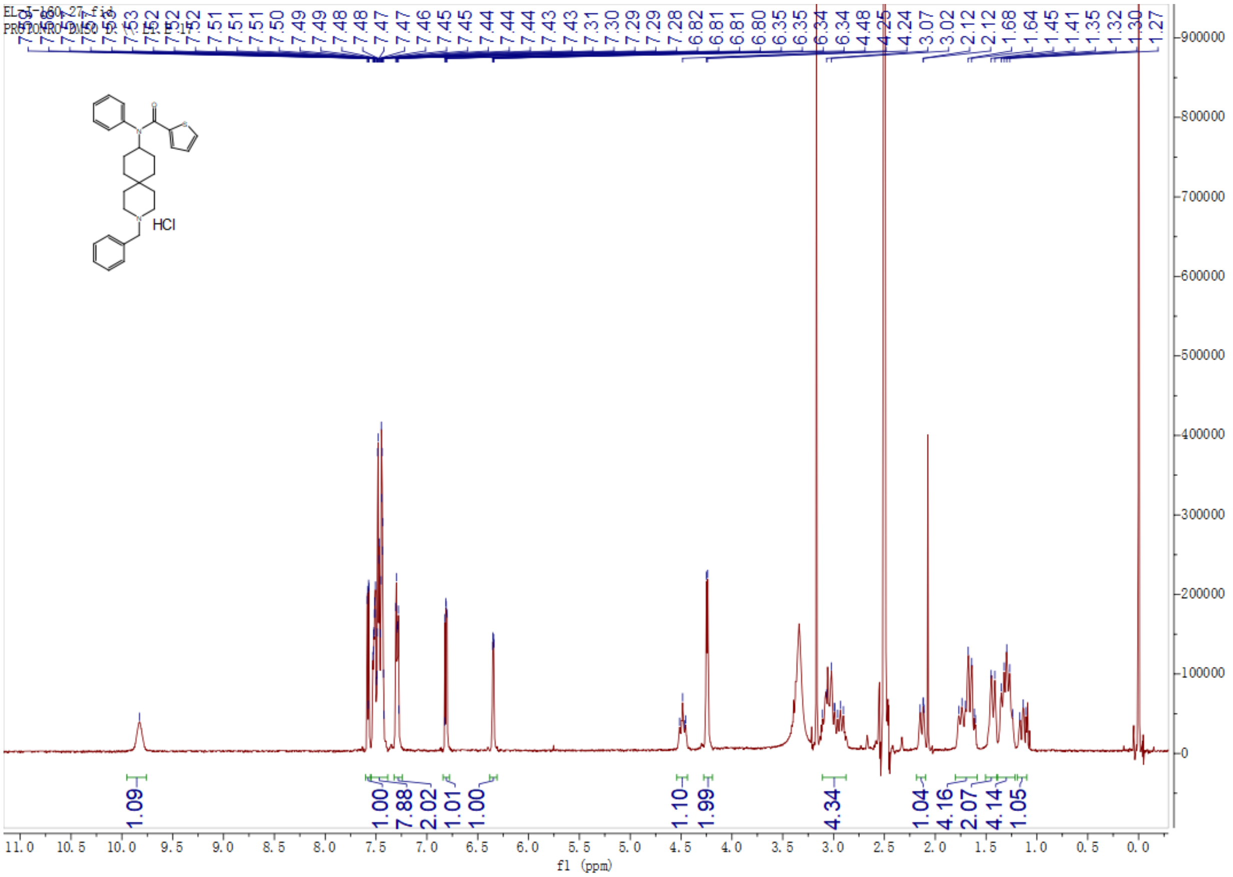

^{1}H NMR (400 MHz, DMSO-d_6) spectrum for compound **56**.

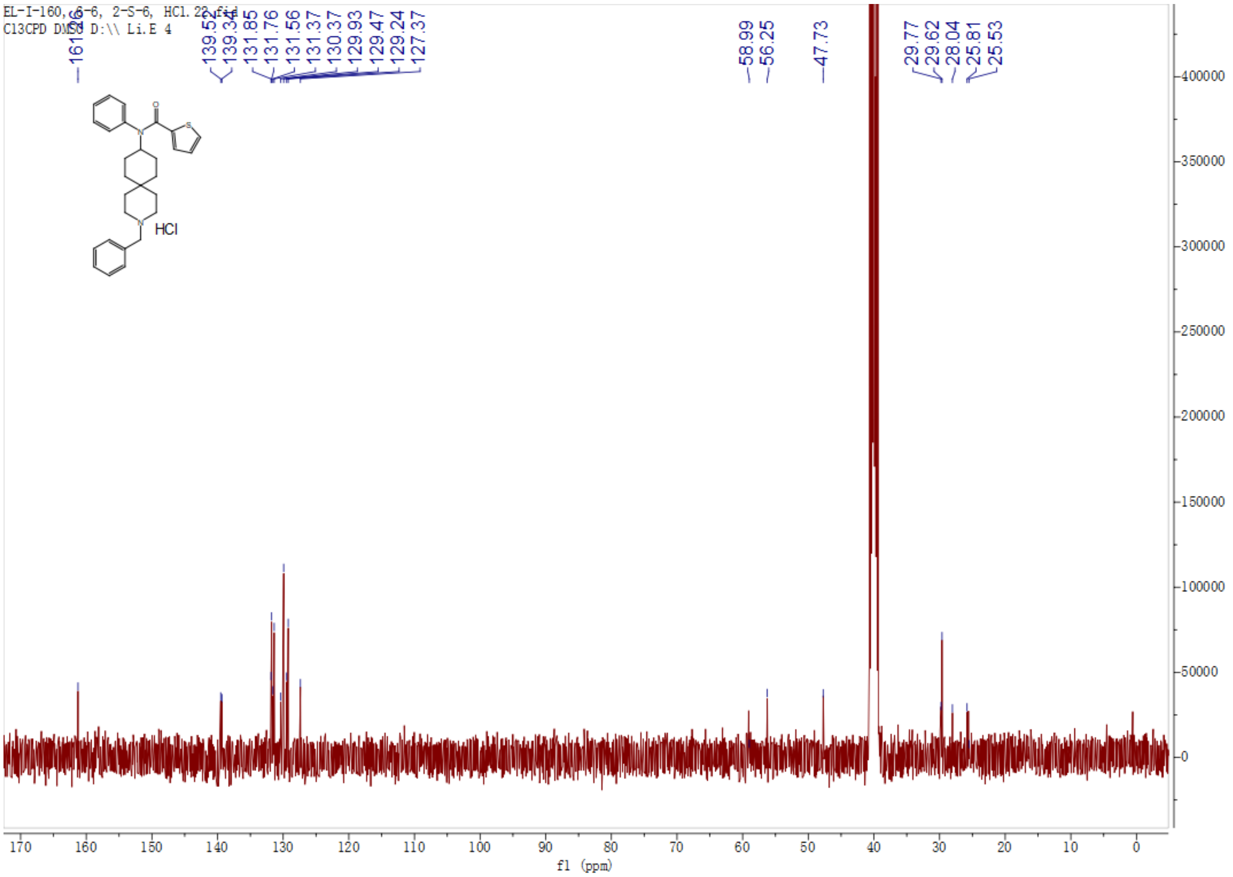

^{13}C NMR (100 MHz, DMSO-d_6) spectrum for compound **56**.

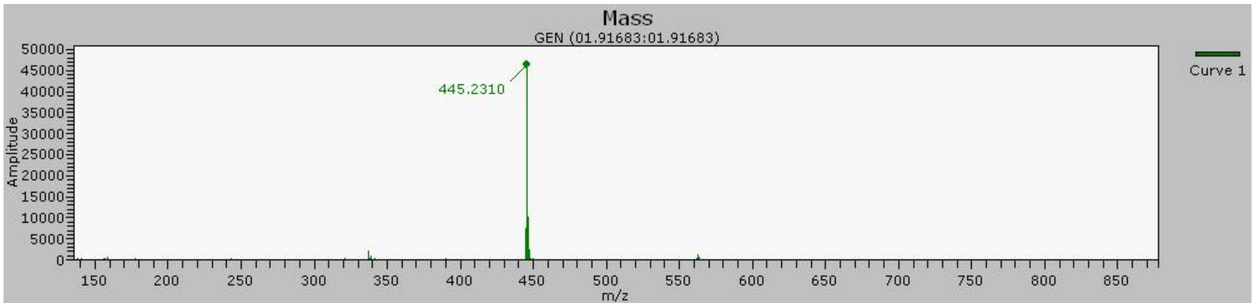

HRMS spectrum for compound **56**.

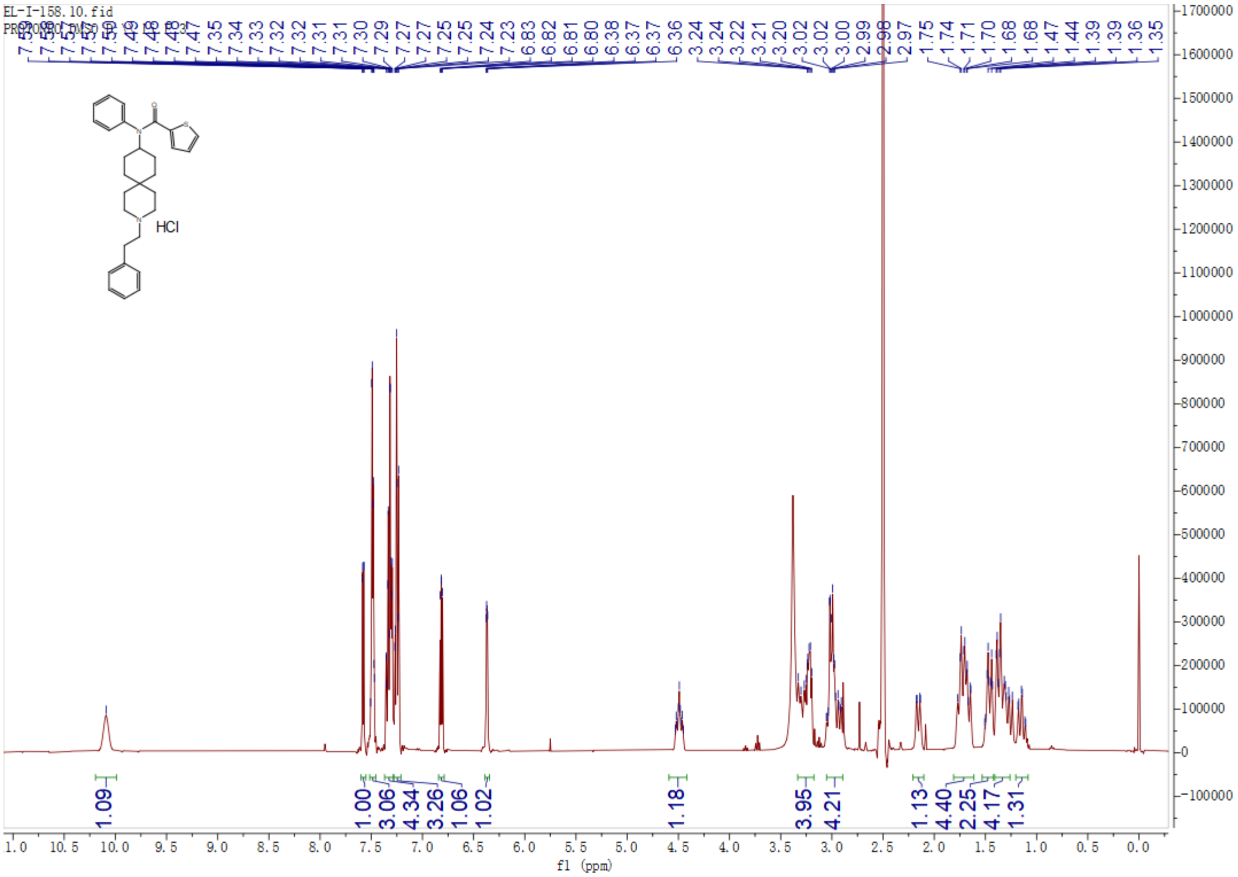

^{1}H NMR (400 MHz, DMSO-d_6) spectrum for compound **57**.

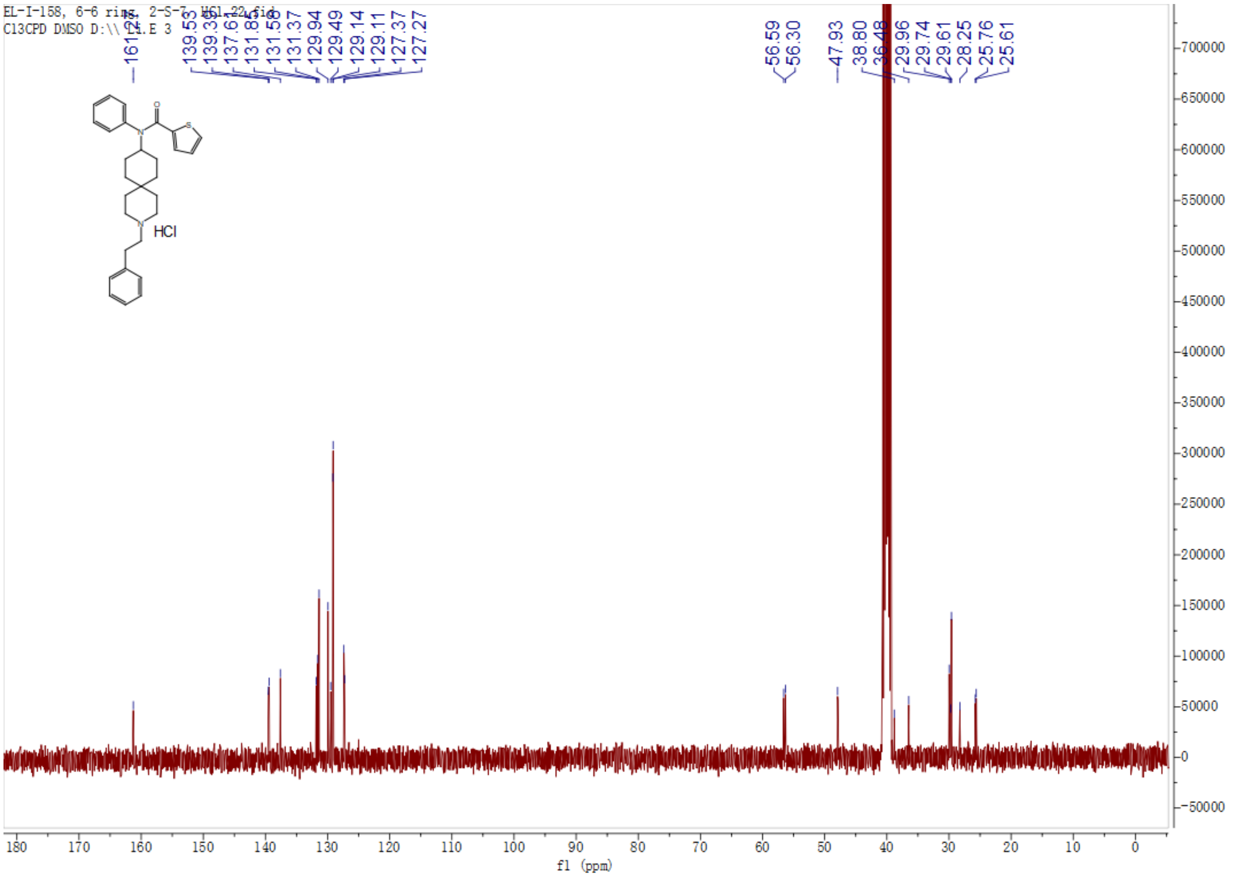

^{13}C NMR (100 MHz, DMSO-d_6) spectrum for compound **57**.

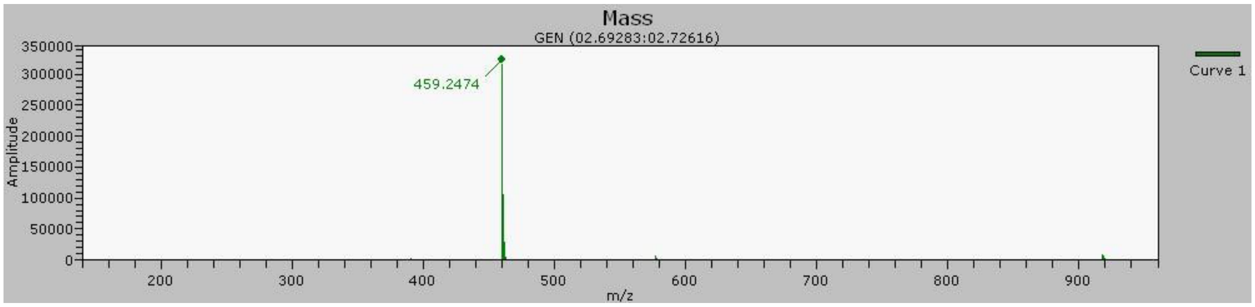

HRMS spectrum for compound **57**.

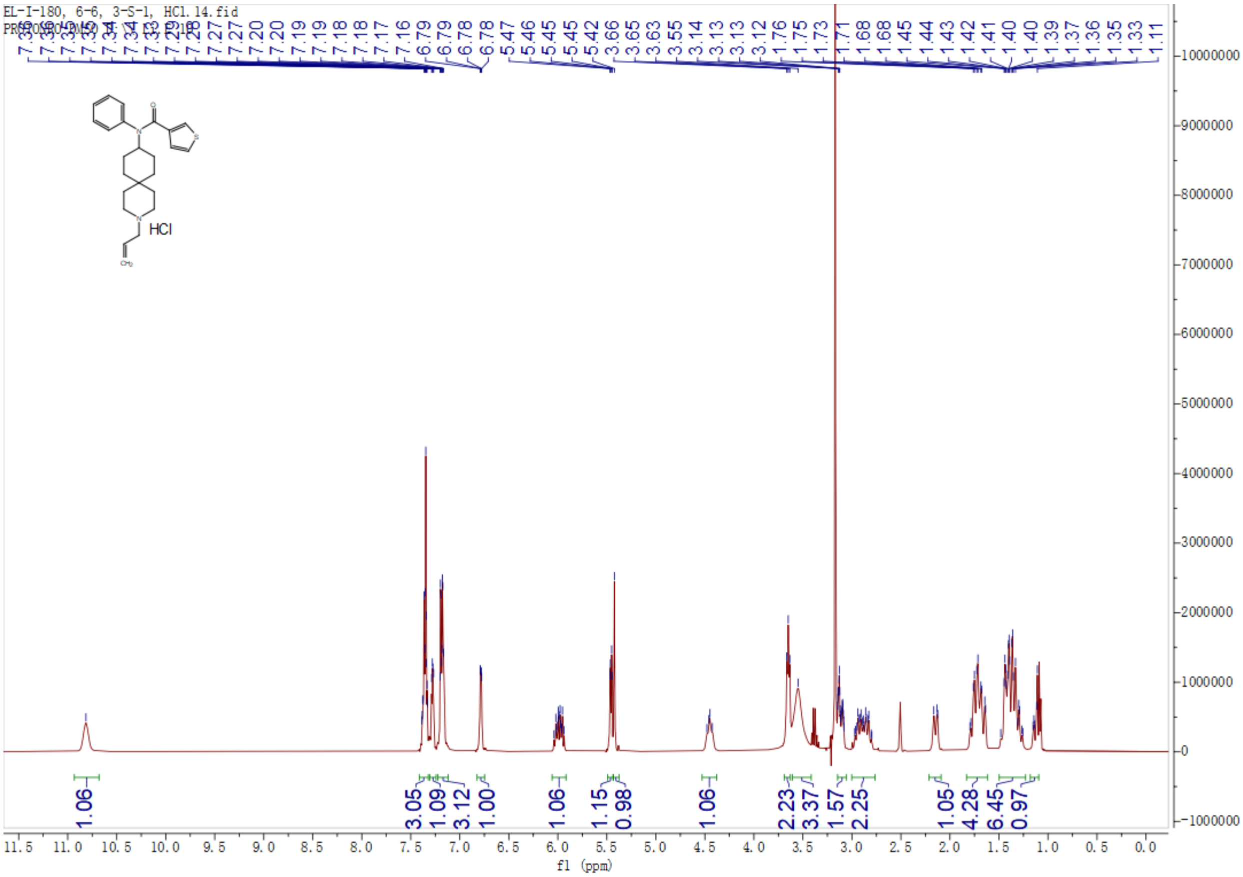

^{1}H NMR (400 MHz, DMSO-d_6) spectrum for compound **58**.

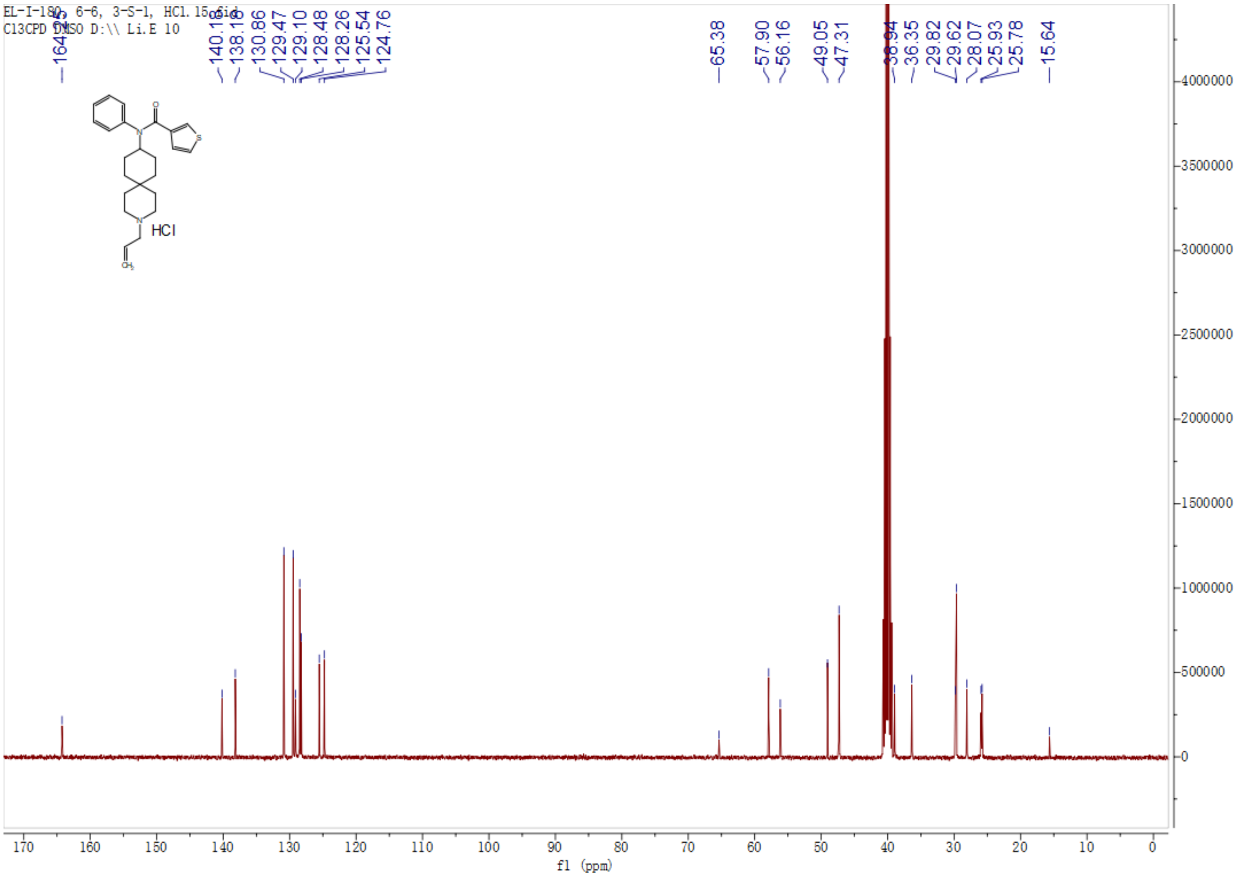

^{13}C NMR (100 MHz, DMSO-d_6) spectrum for compound **58**.

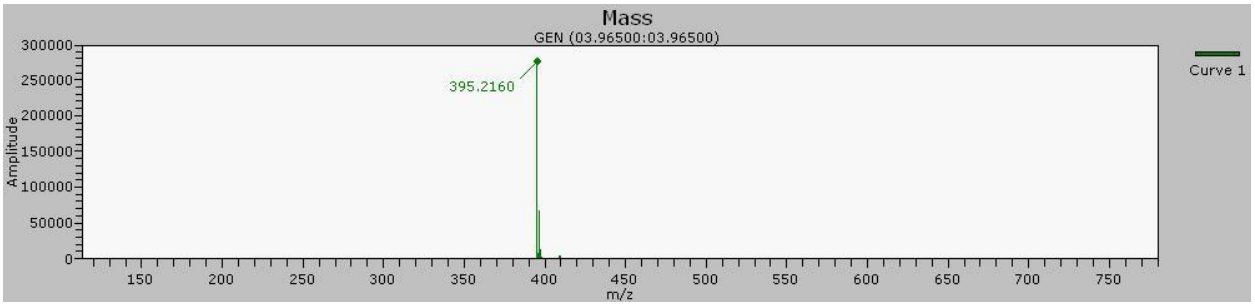

HRMS spectrum for compound **58**.

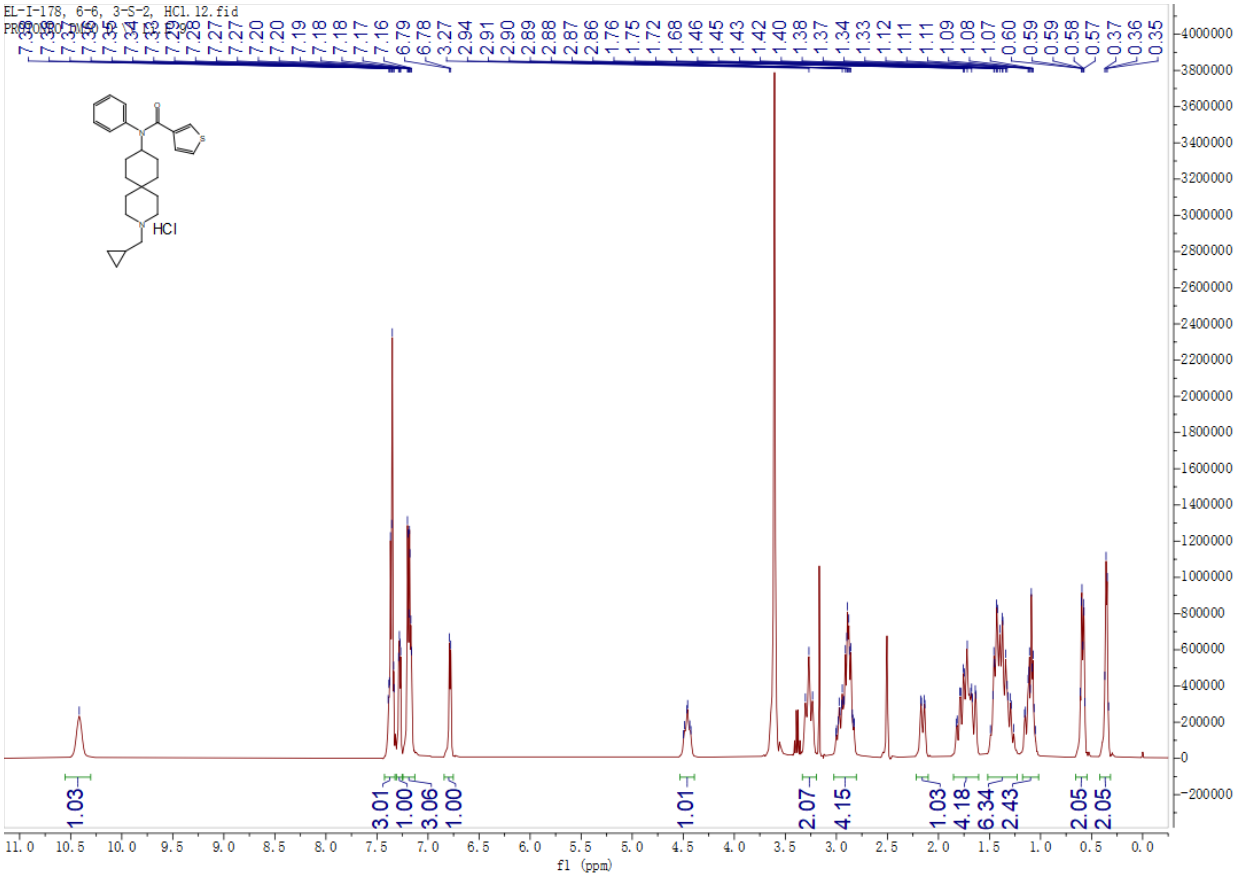

^{1}H NMR (400 MHz, DMSO-d_6) spectrum for compound **59**.

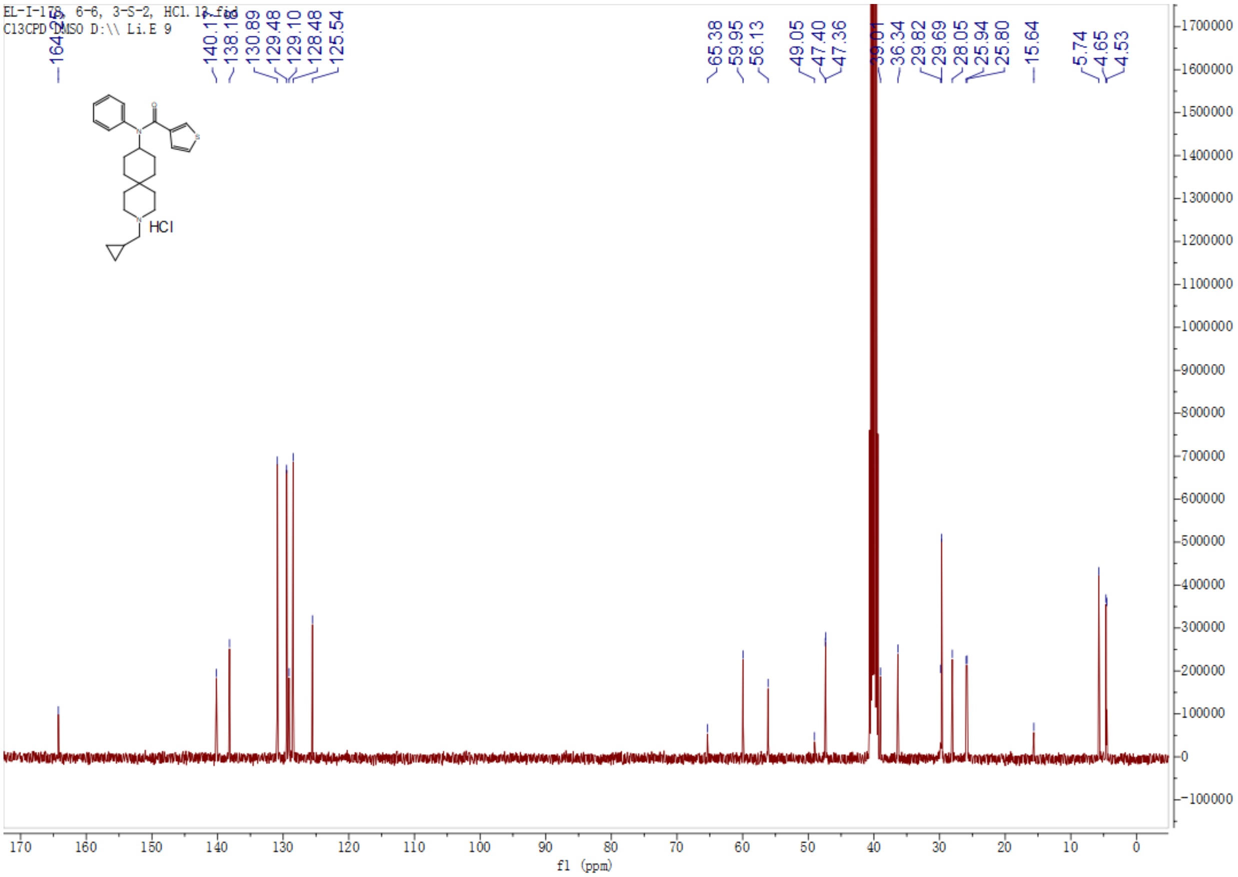

^{13}C NMR (100 MHz, DMSO-d_6) spectrum for compound **59**.

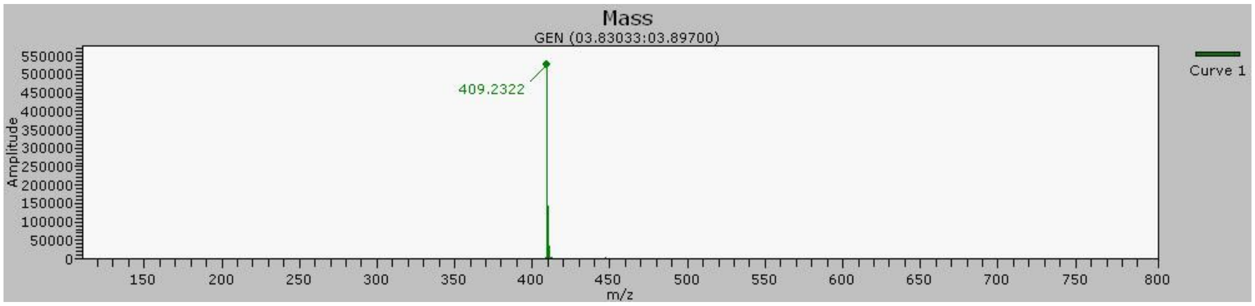

HRMS spectrum for compound **59**.

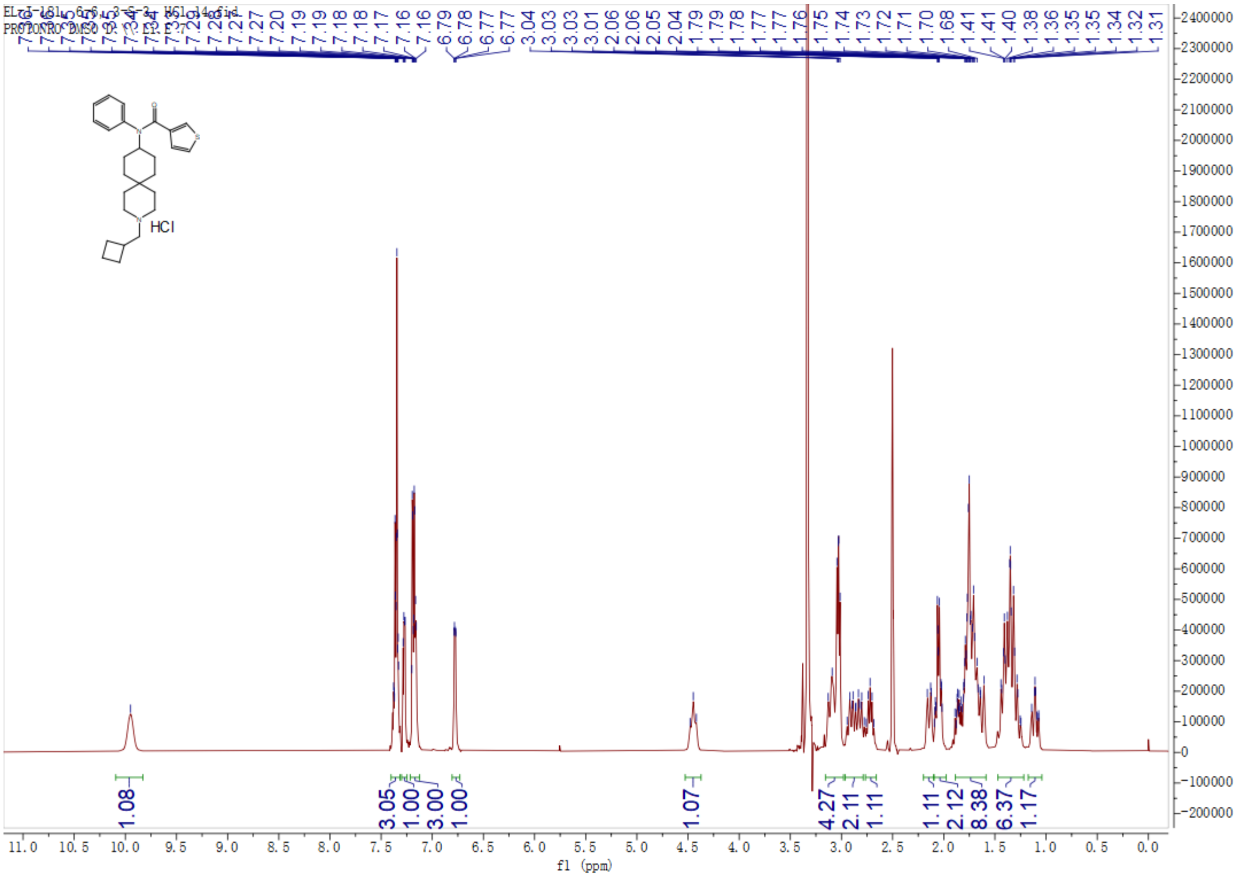

^{1}H NMR (400 MHz, DMSO-d_6) spectrum for compound **60**.

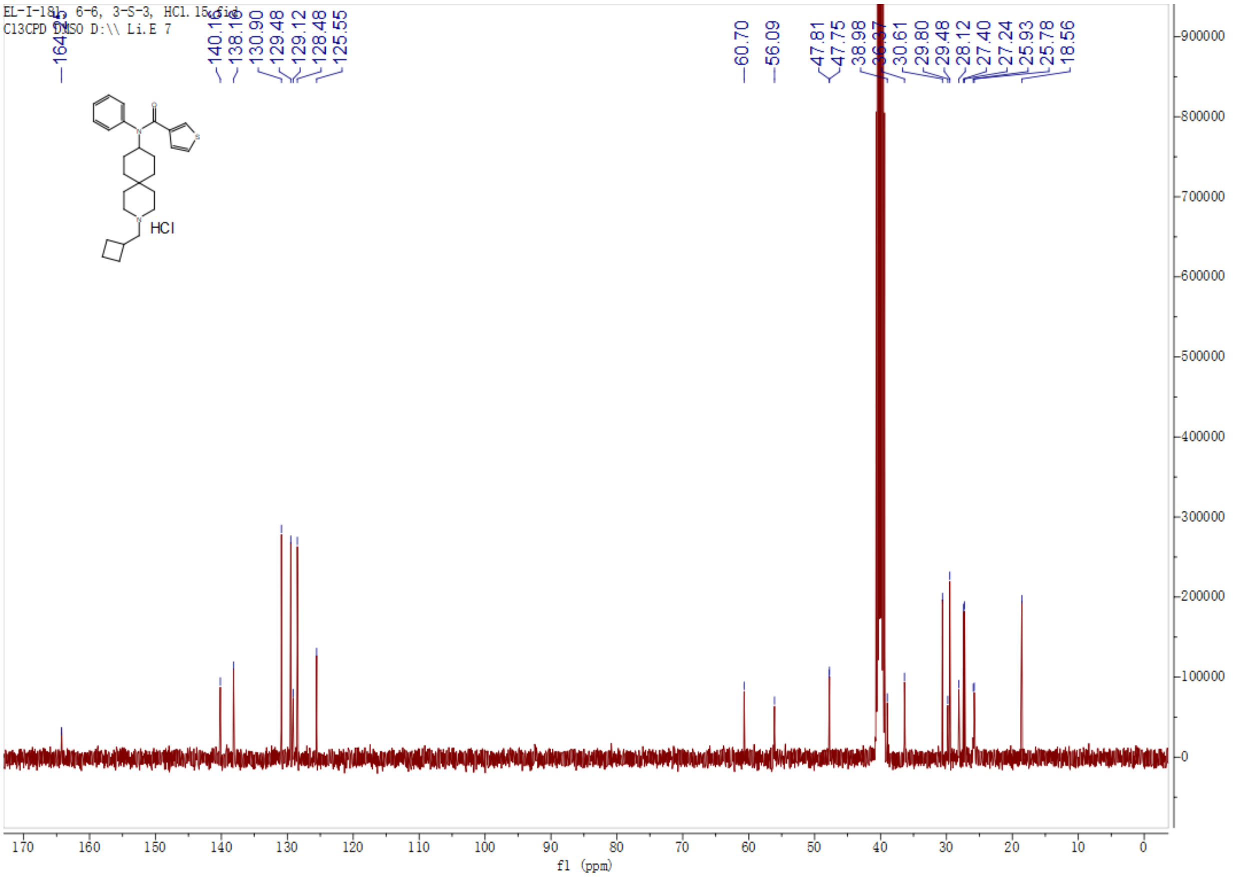

^{13}C NMR (100 MHz, DMSO-d_6) spectrum for compound **60**.

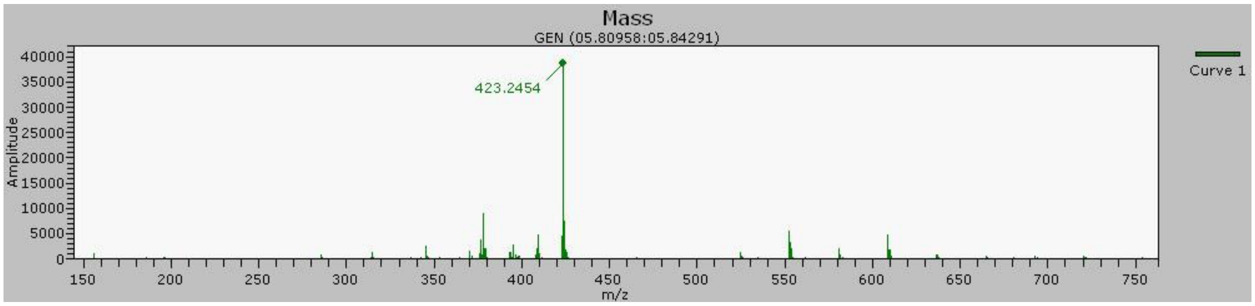

HRMS spectrum for compound **60**.

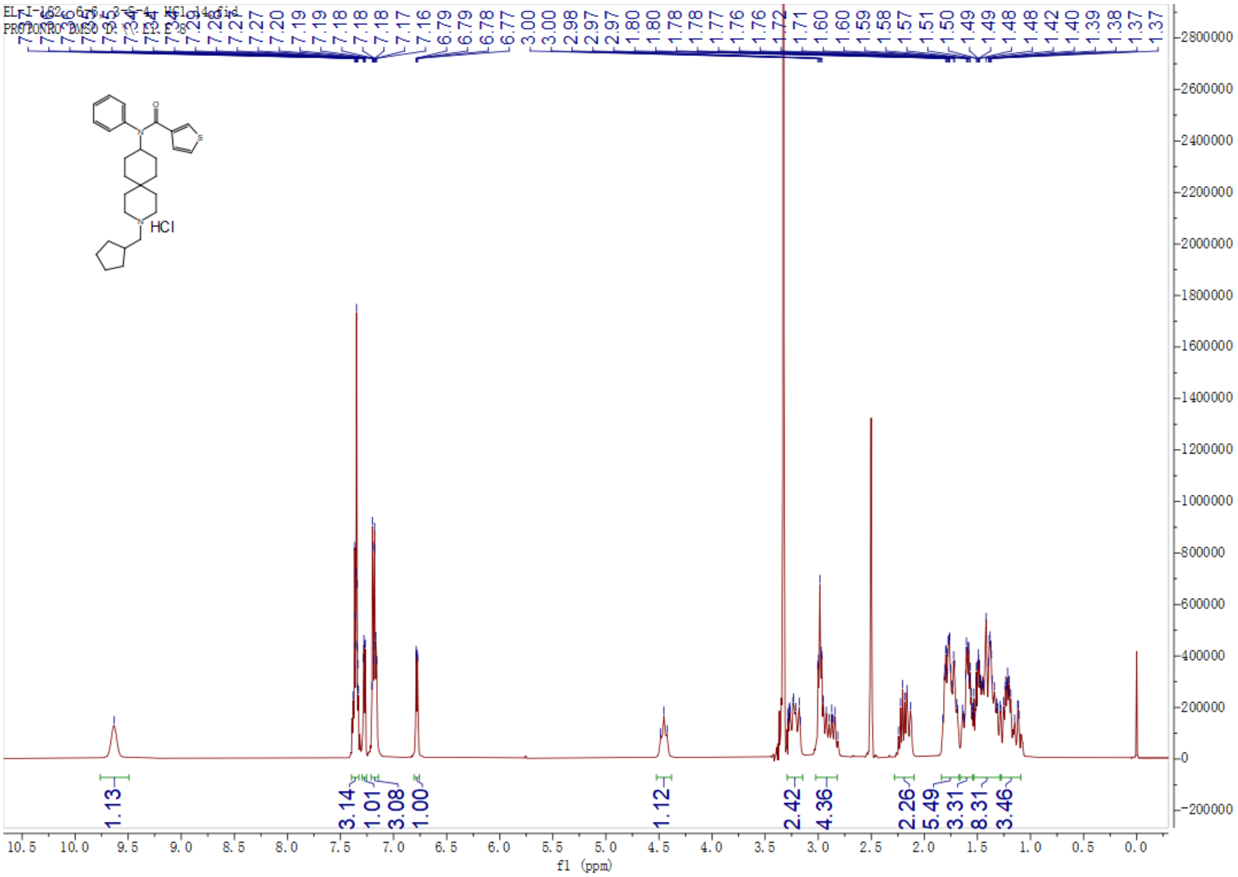

^{1}H NMR (400 MHz, DMSO-d_6) spectrum for compound **61**.

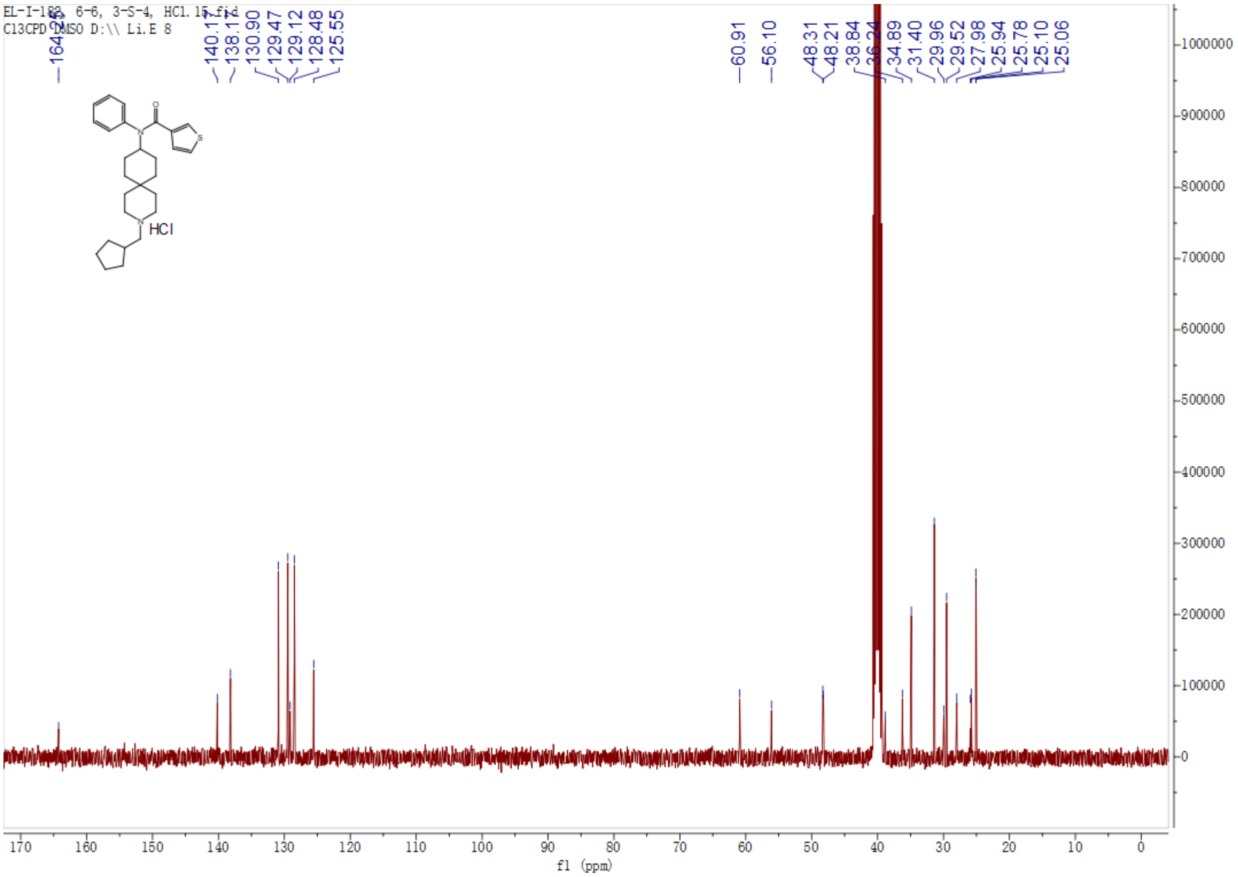

^{13}C NMR (100 MHz, DMSO-d_6) spectrum for compound **61**.

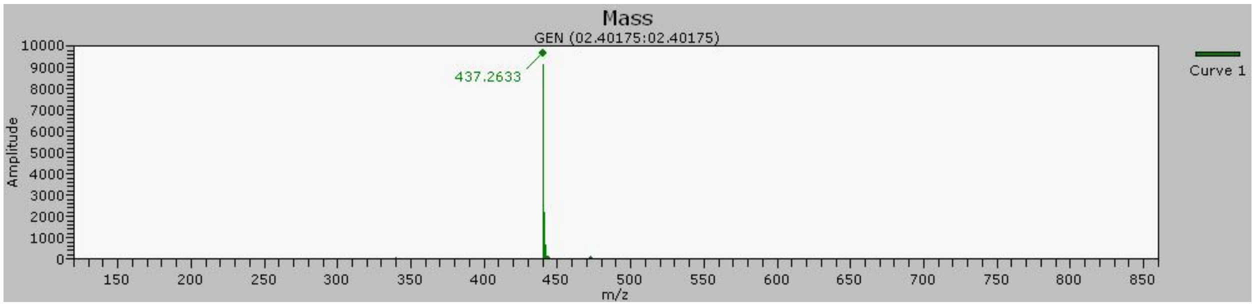

HRMS spectrum for compound **61**.

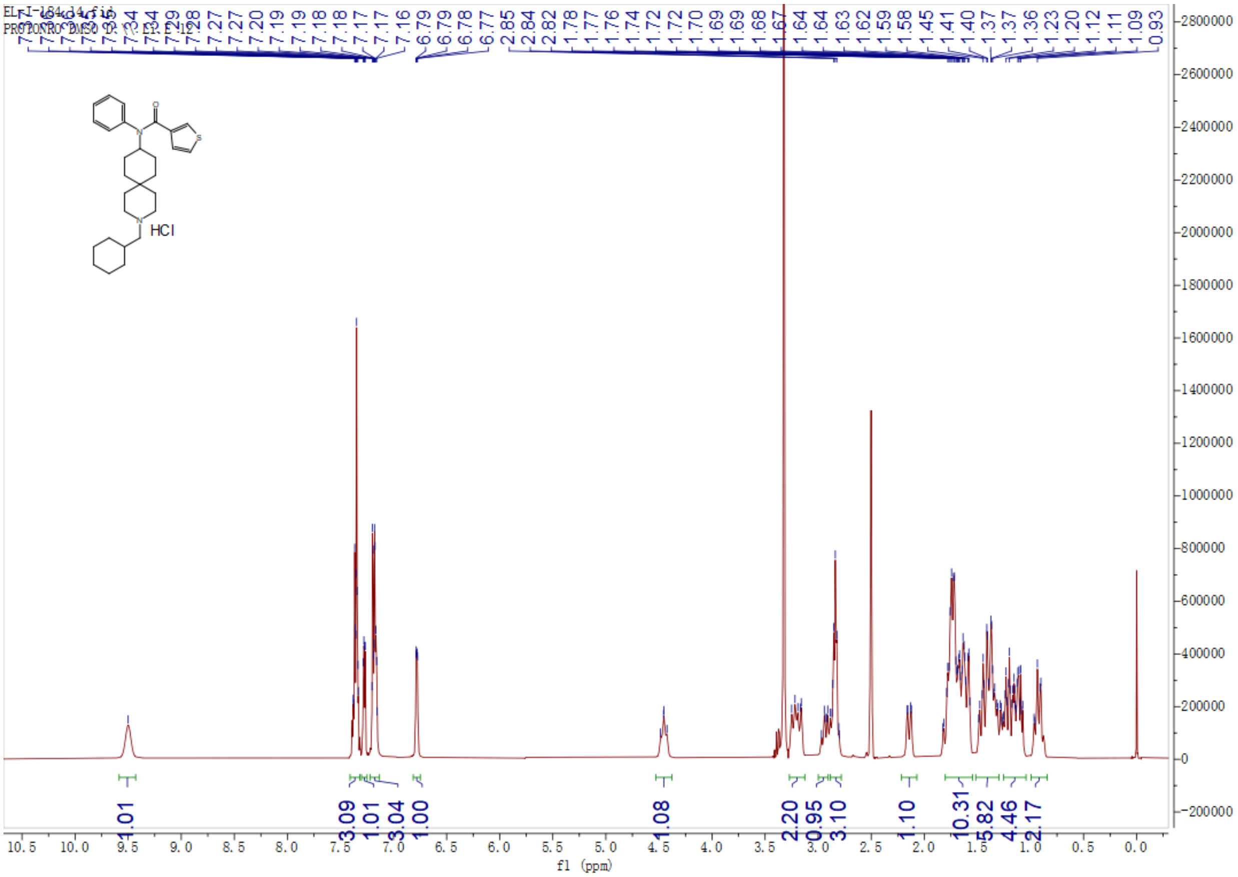

^{1}H NMR (400 MHz, DMSO-d_6) spectrum for compound **62**.

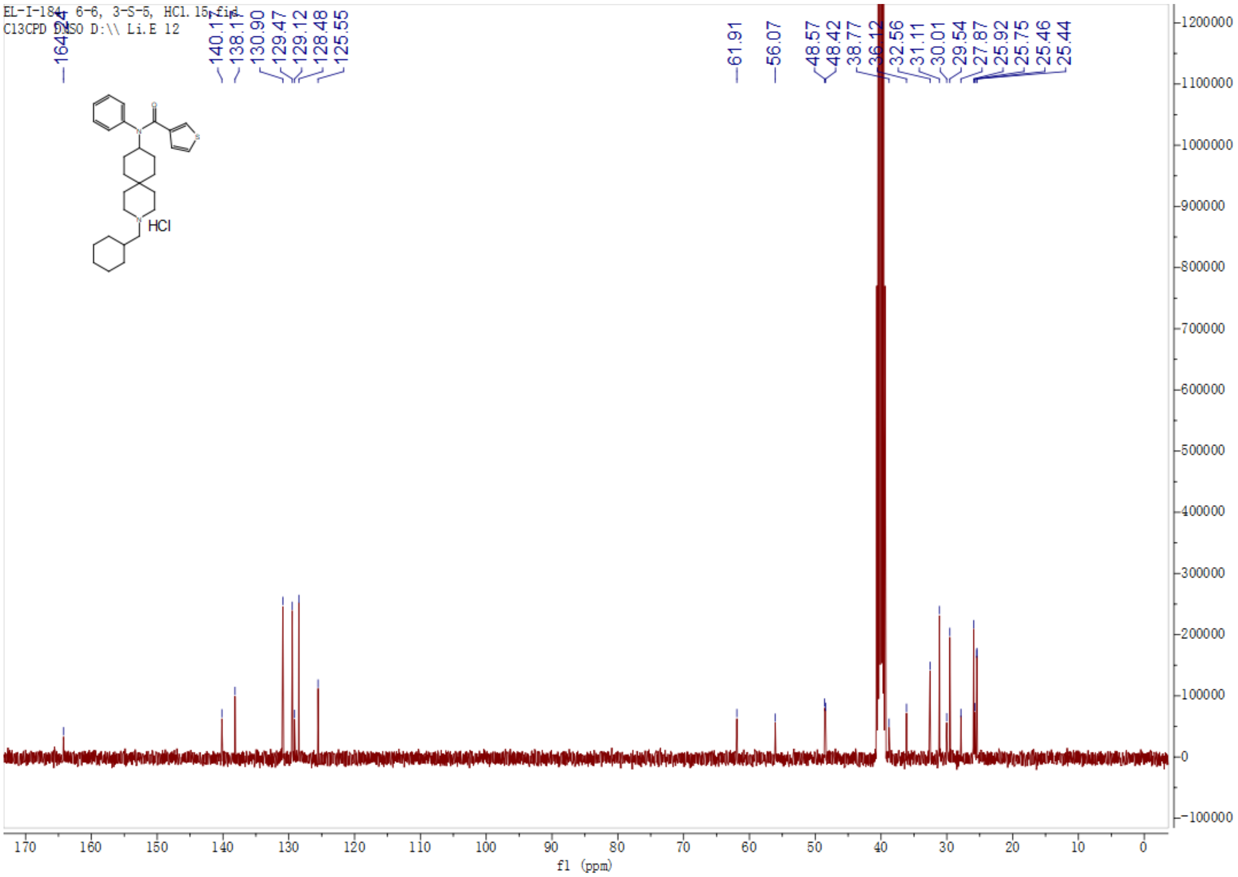

^{13}C NMR (100 MHz, DMSO-d_6) spectrum for compound **62**.

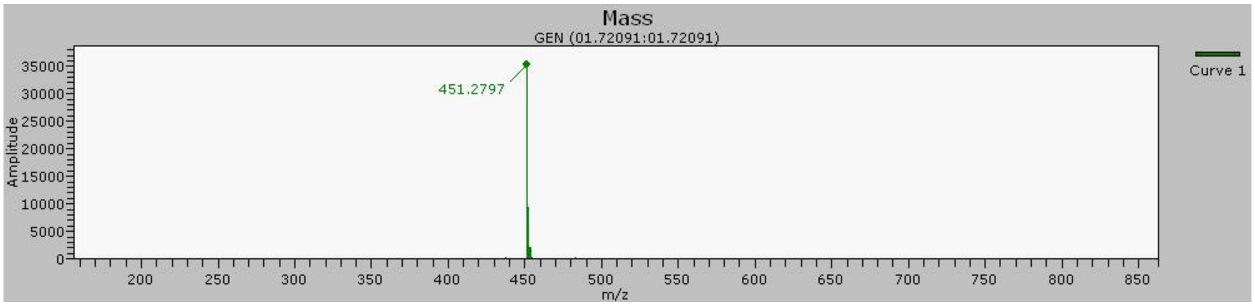

HRMS spectrum for compound **62**.

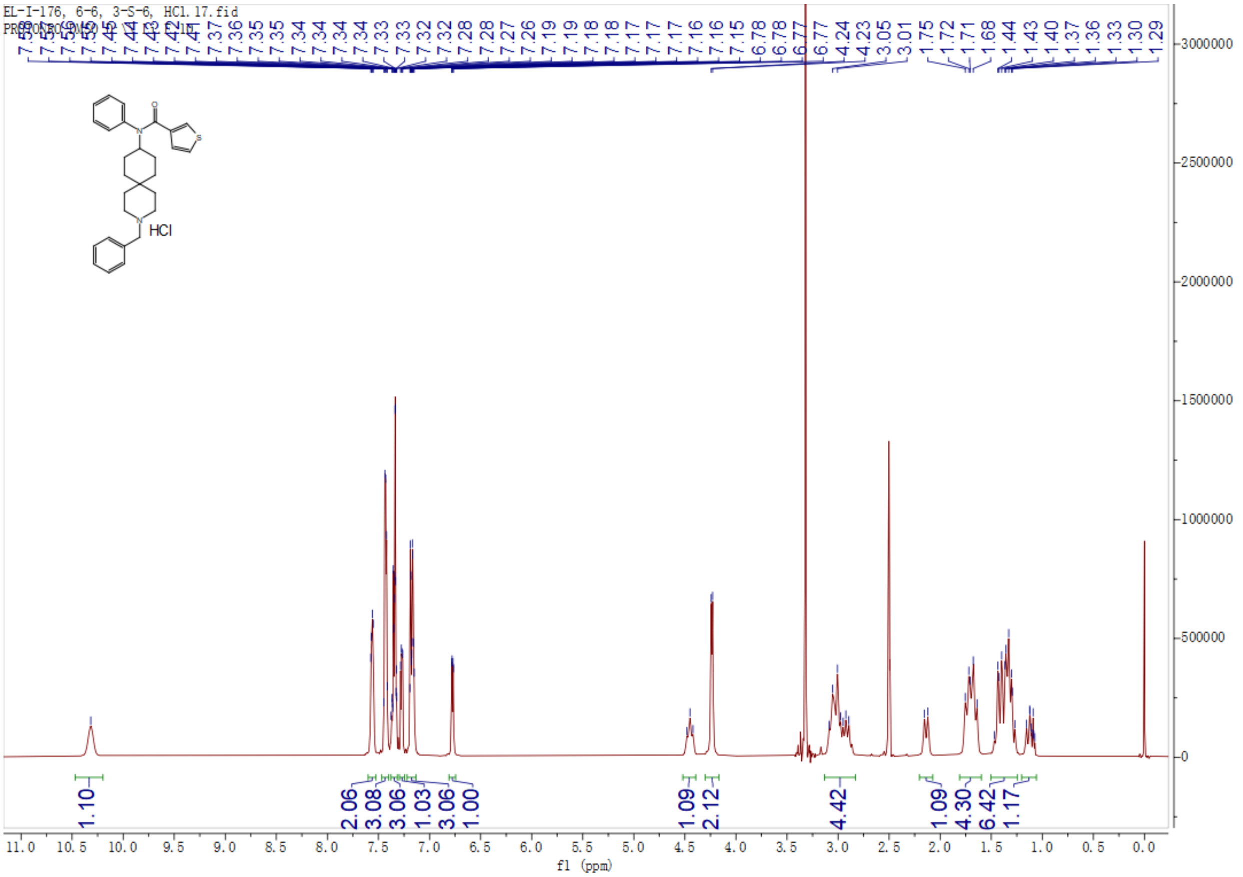

^{1}H NMR (400 MHz, DMSO-d_6) spectrum for compound **63**.

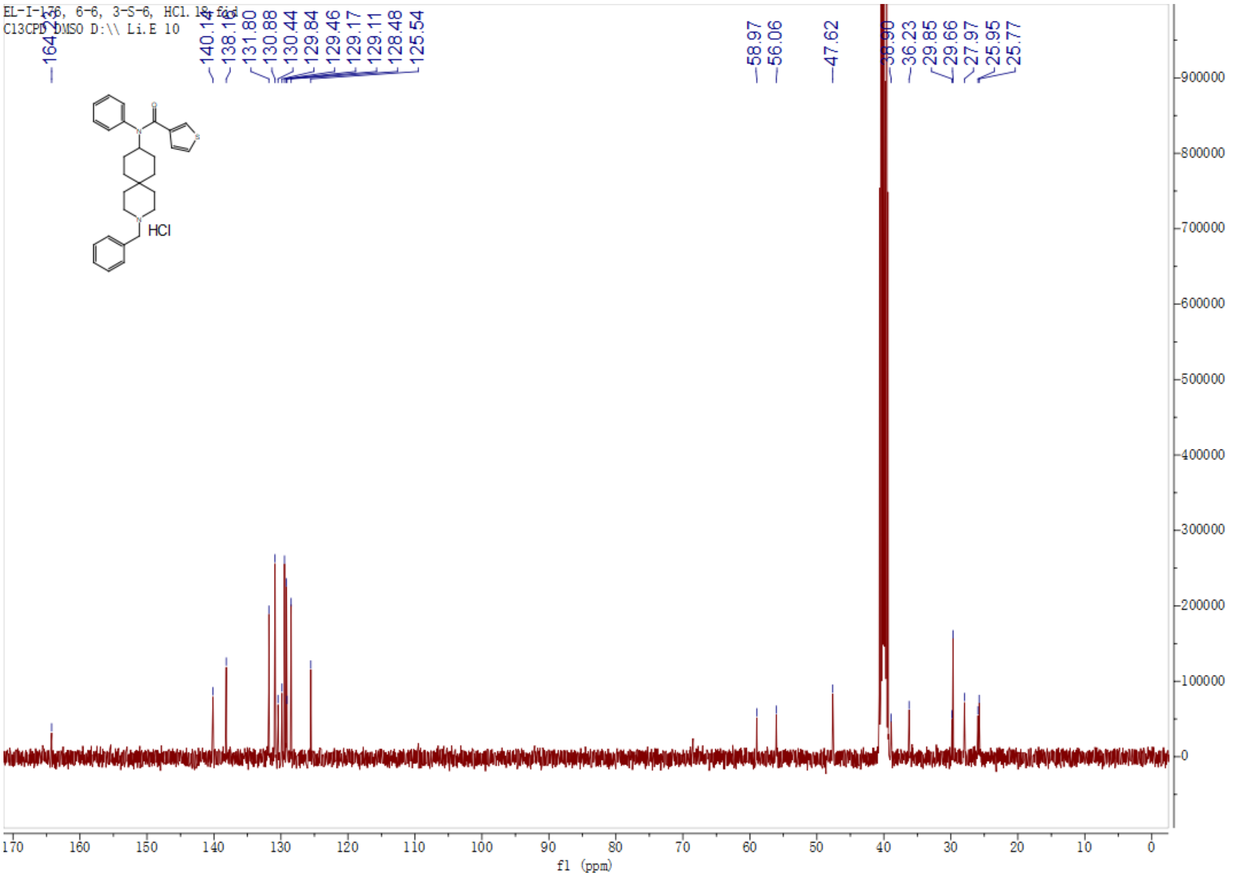

^{13}C NMR (100 MHz, DMSO-d_6) spectrum for compound **63**.

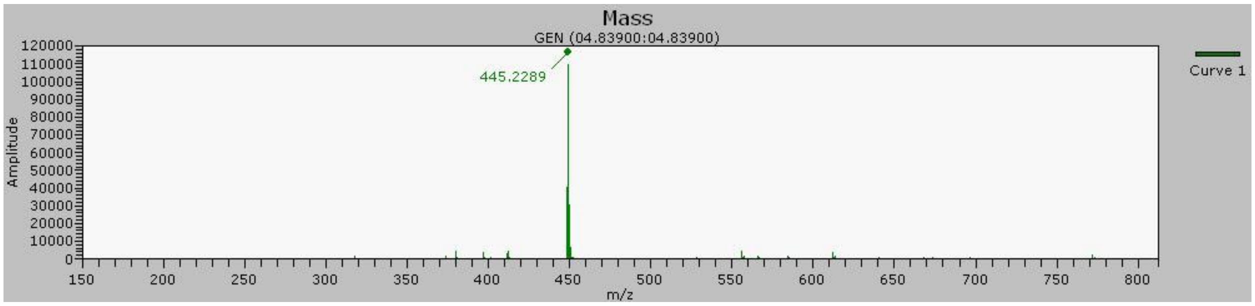

HRMS spectrum for compound **63**.

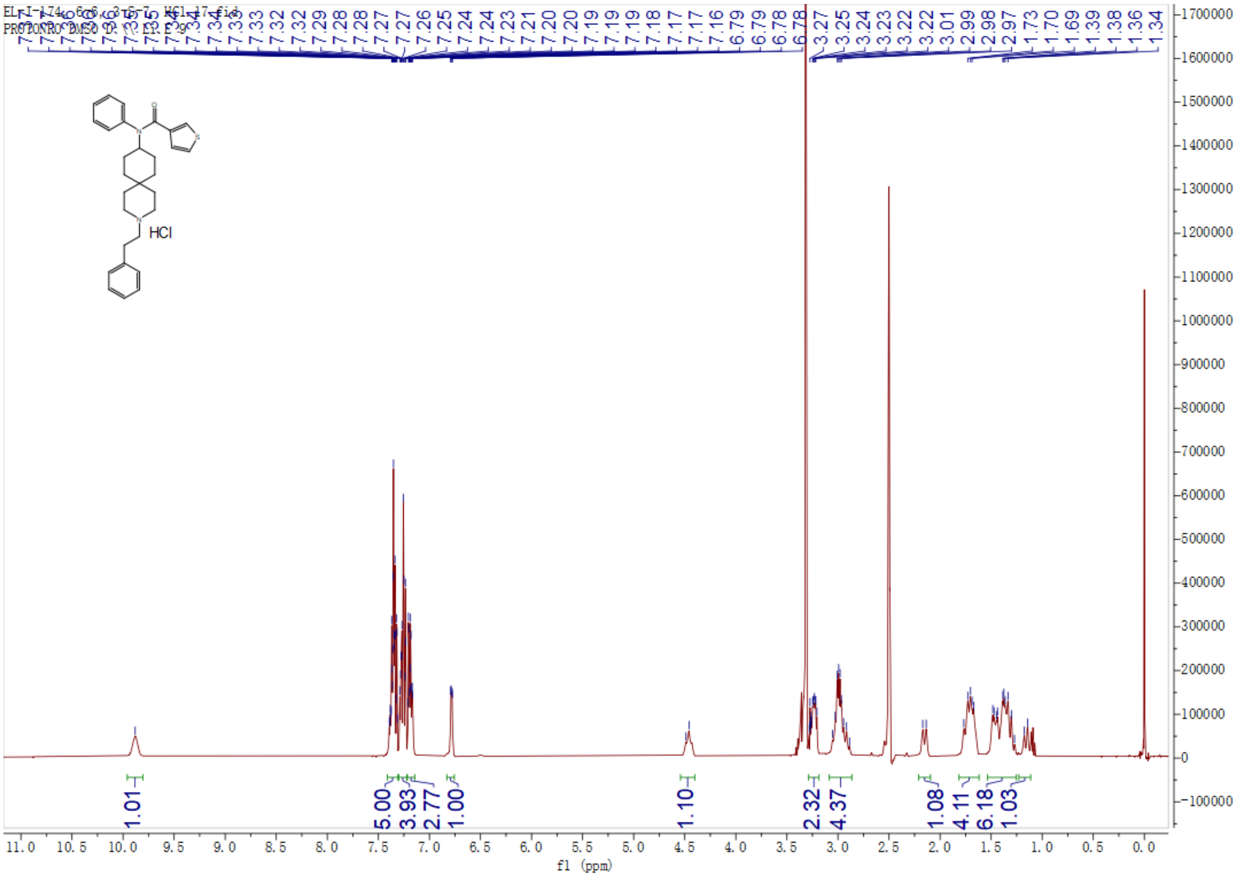

^{1}H NMR (400 MHz, DMSO-d_6) spectrum for compound **64**.

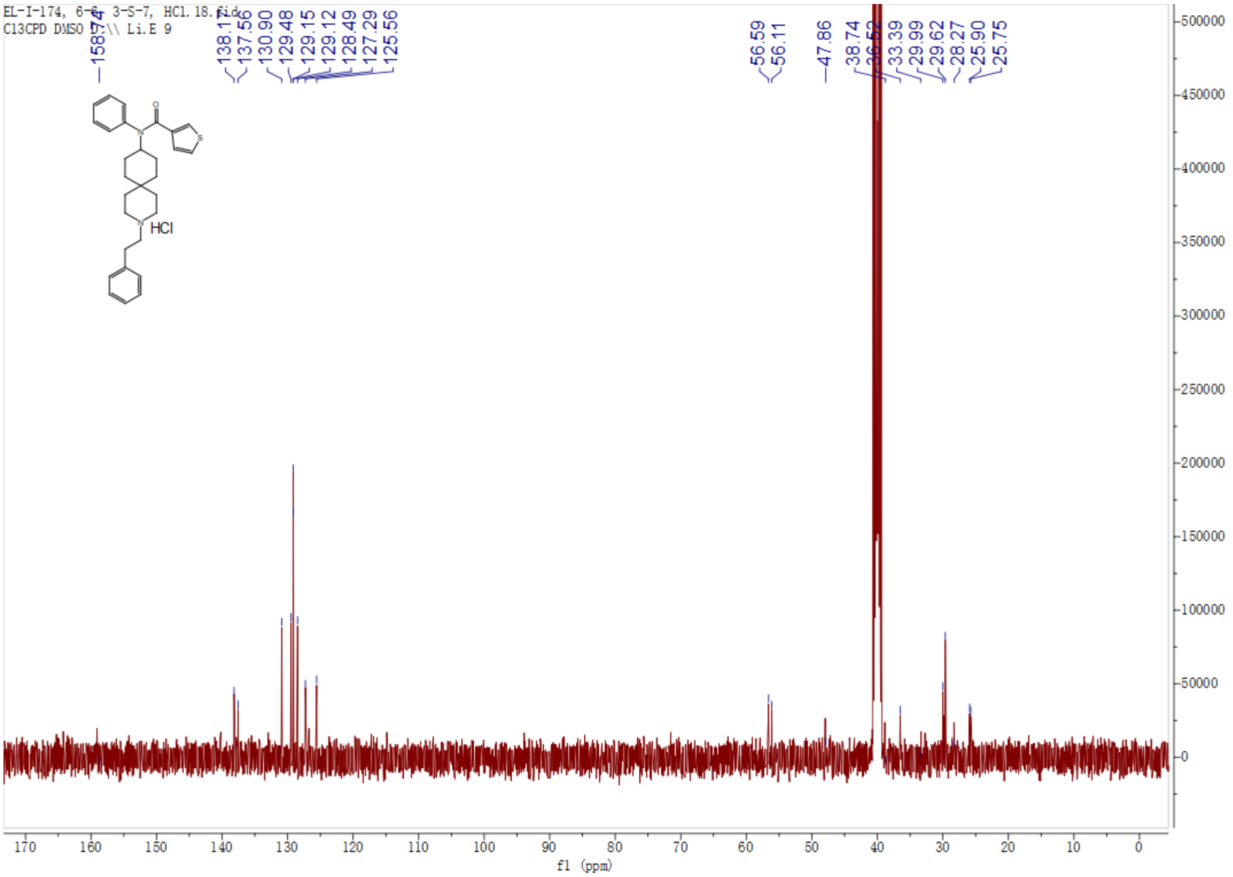

^{13}C NMR (100 MHz, DMSO-d_6) spectrum for compound **64**.

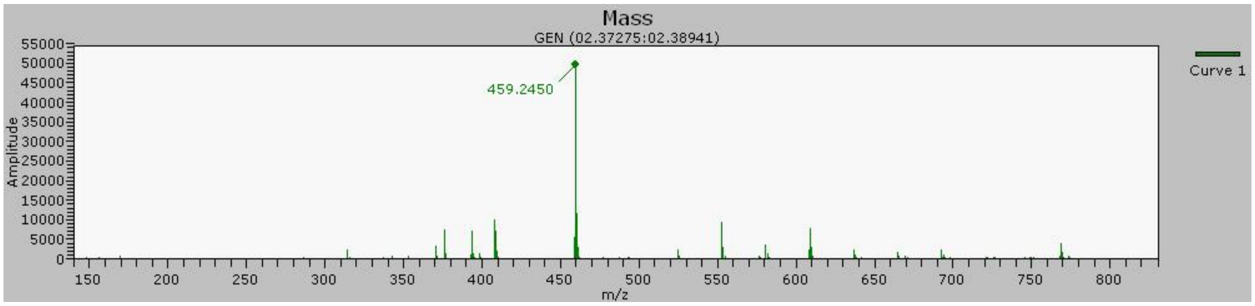

HRMS spectrum for compound **64**.

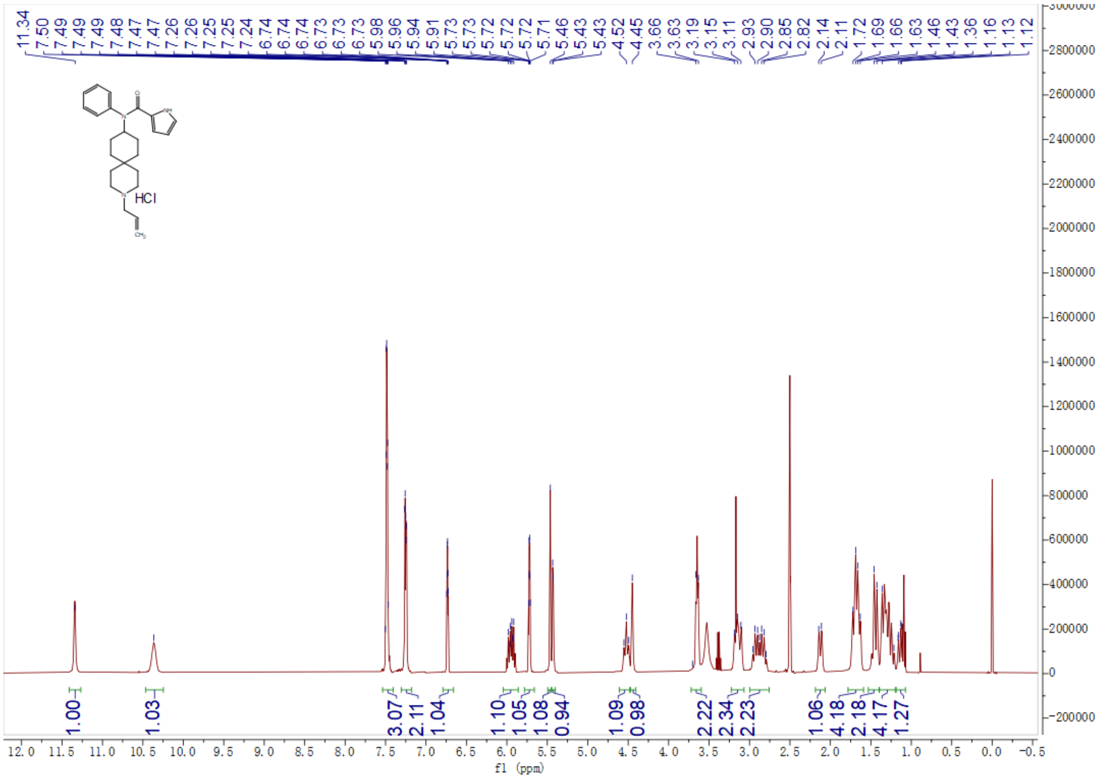

^{1}H NMR (400 MHz, DMSO-d_6) spectrum for compound **65**.

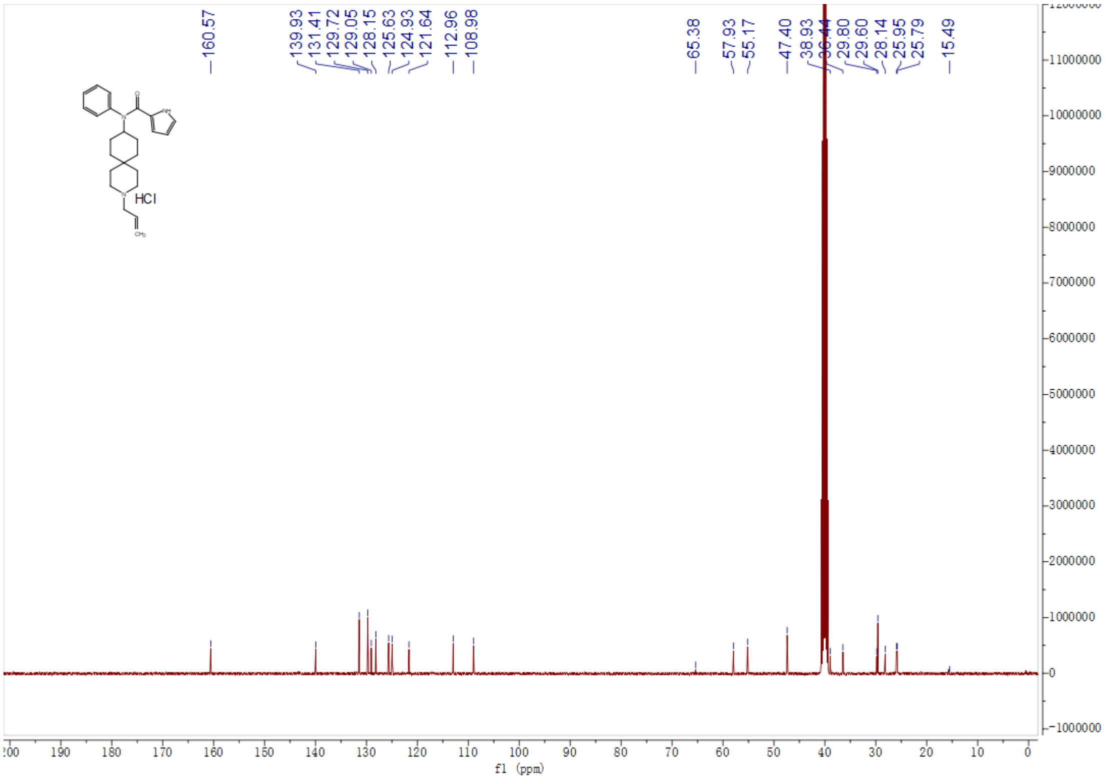

^{13}C NMR (100 MHz, DMSO-d_6) spectrum for compound **65**.

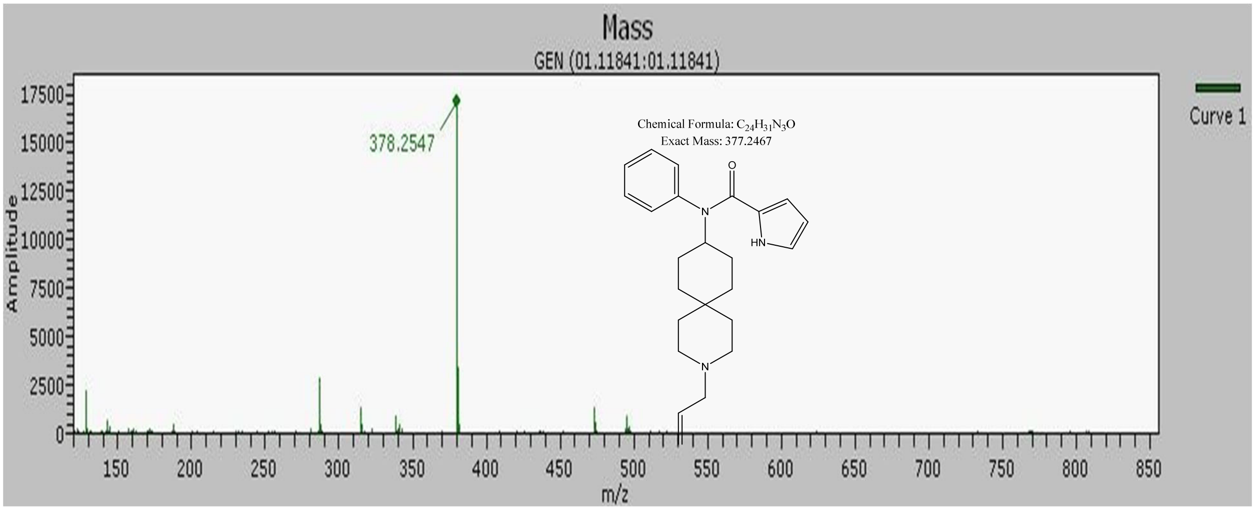

HRMS spectrum for compound **65**.

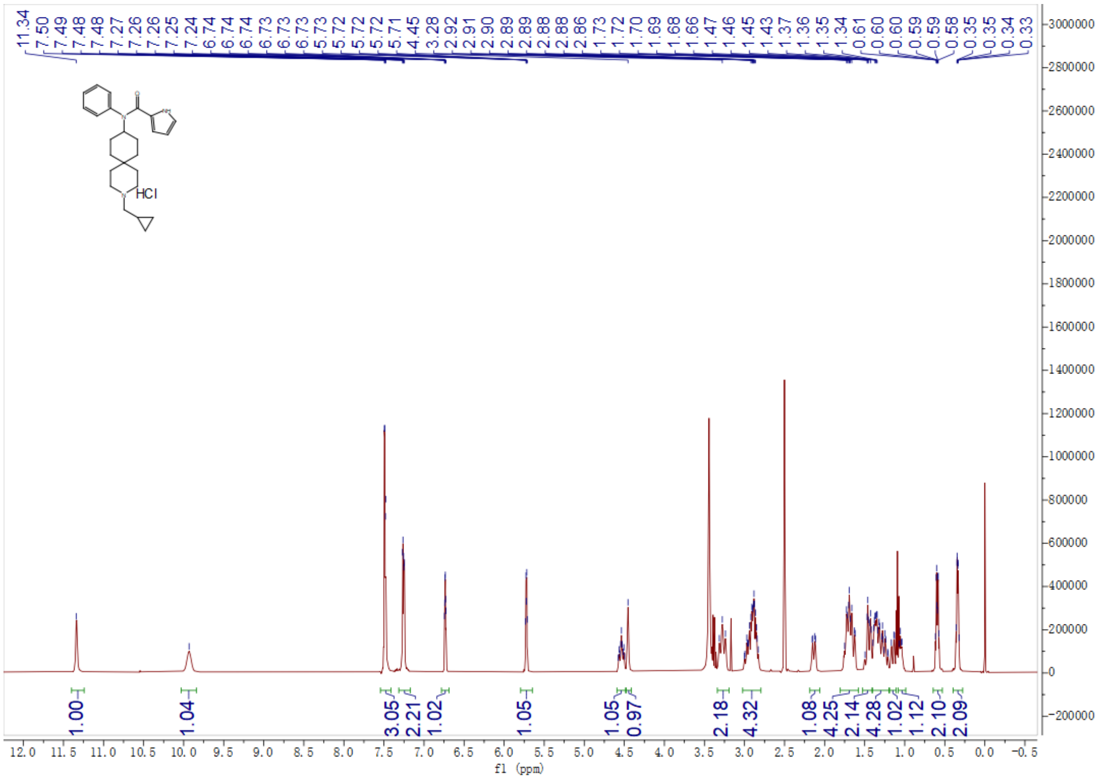

^{1}H NMR (400 MHz, DMSO-*d*6) spectrum for compound **66**.

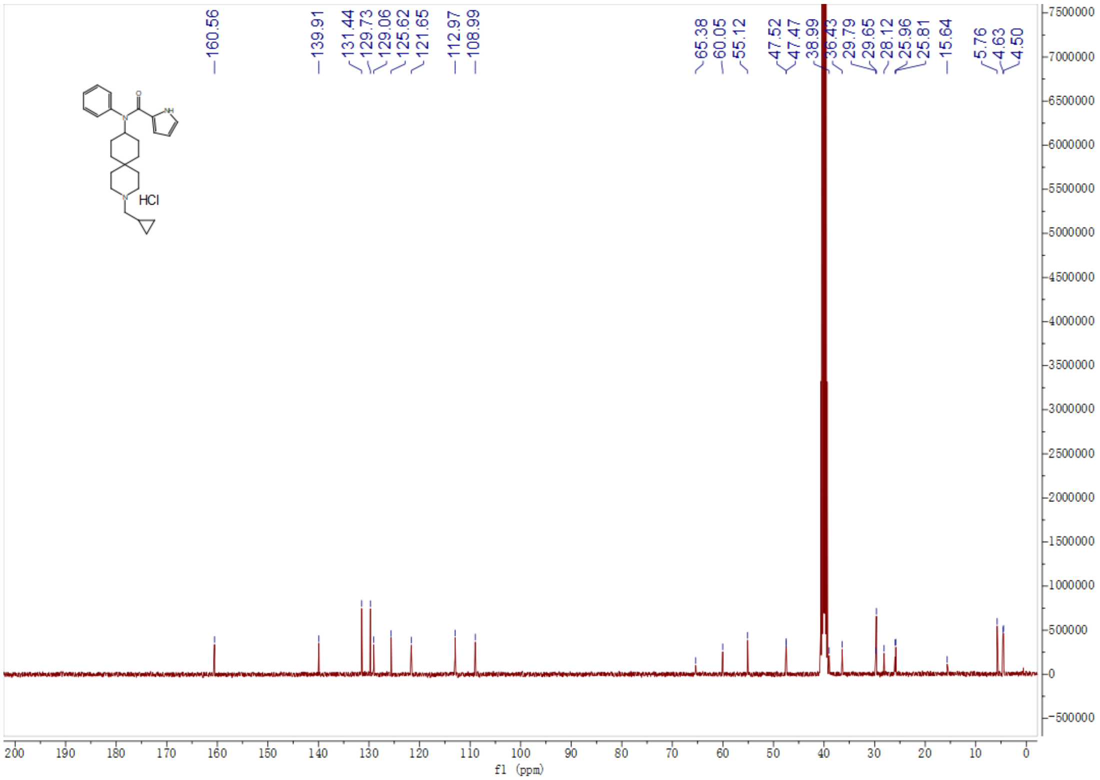

^{13}C NMR (100 MHz, DMSO-d_6) spectrum for compound **66**.

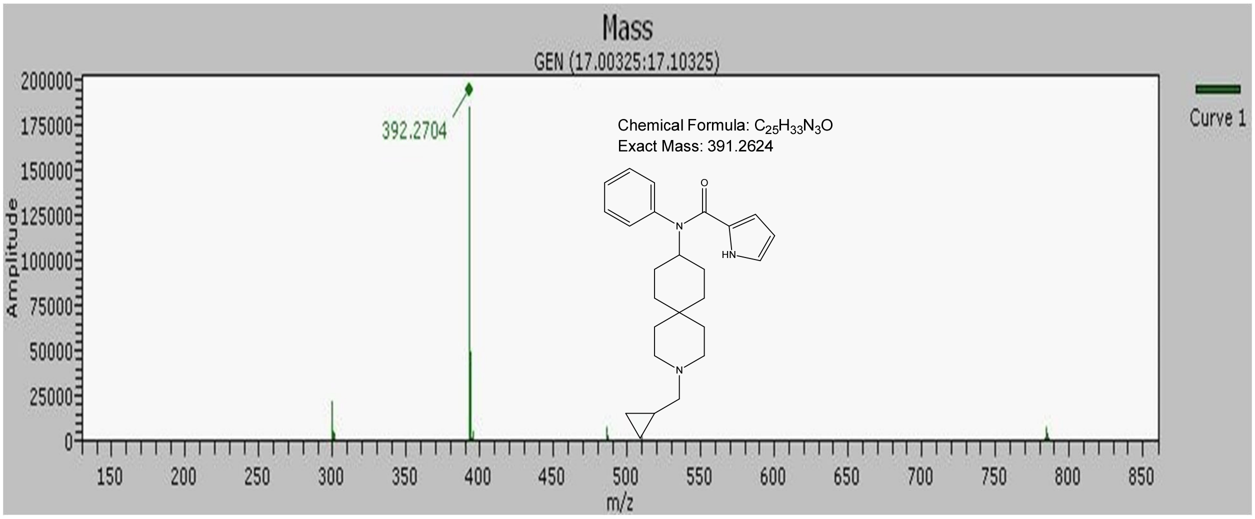

HRMS spectrum for compound **66**.

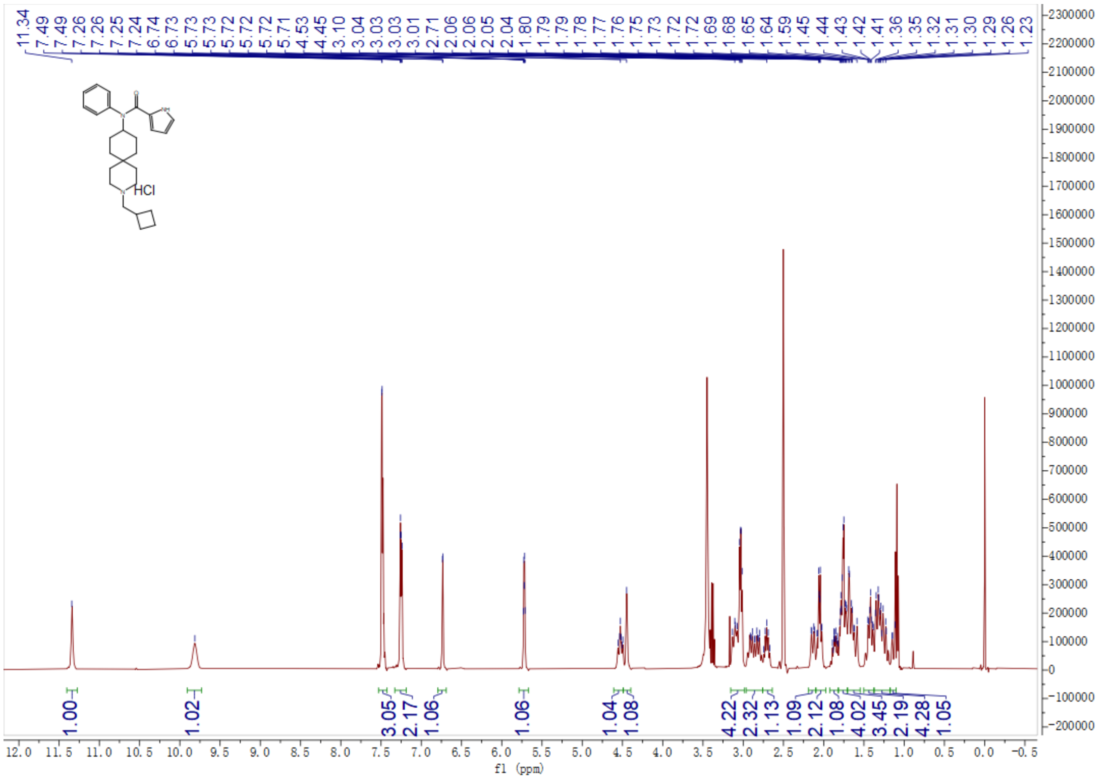

^{1}H NMR (400 MHz, DMSO-d_6) spectrum for compound **67**.

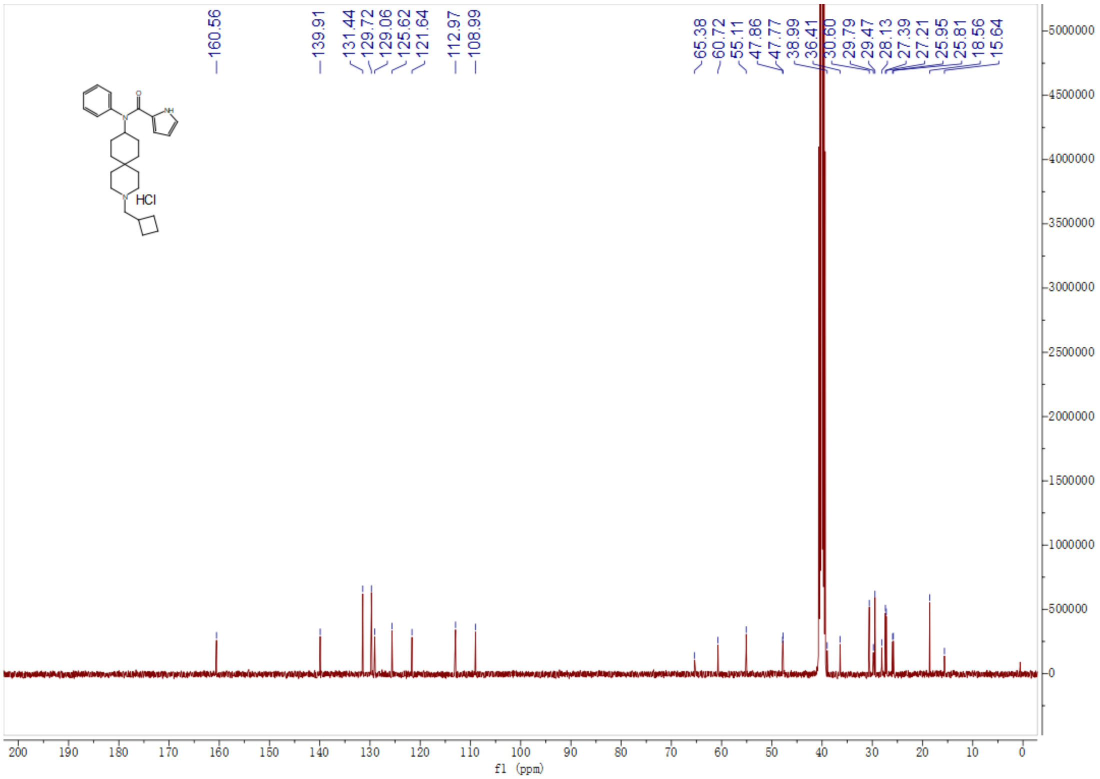

^{13}C NMR (100 MHz, DMSO-d_6) spectrum for compound **67**.

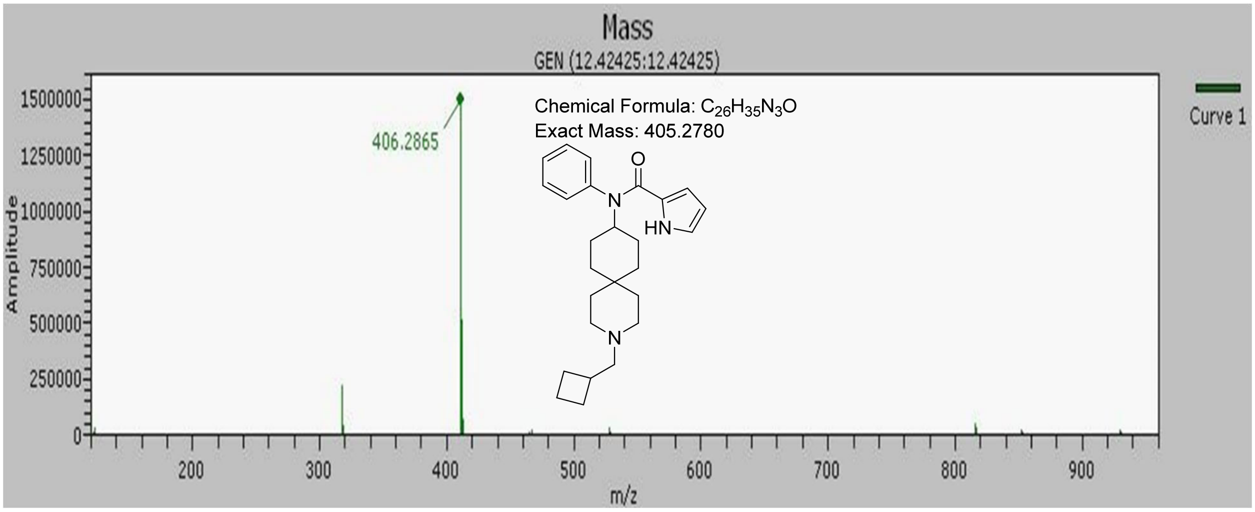

HRMS spectrum for compound **67**.

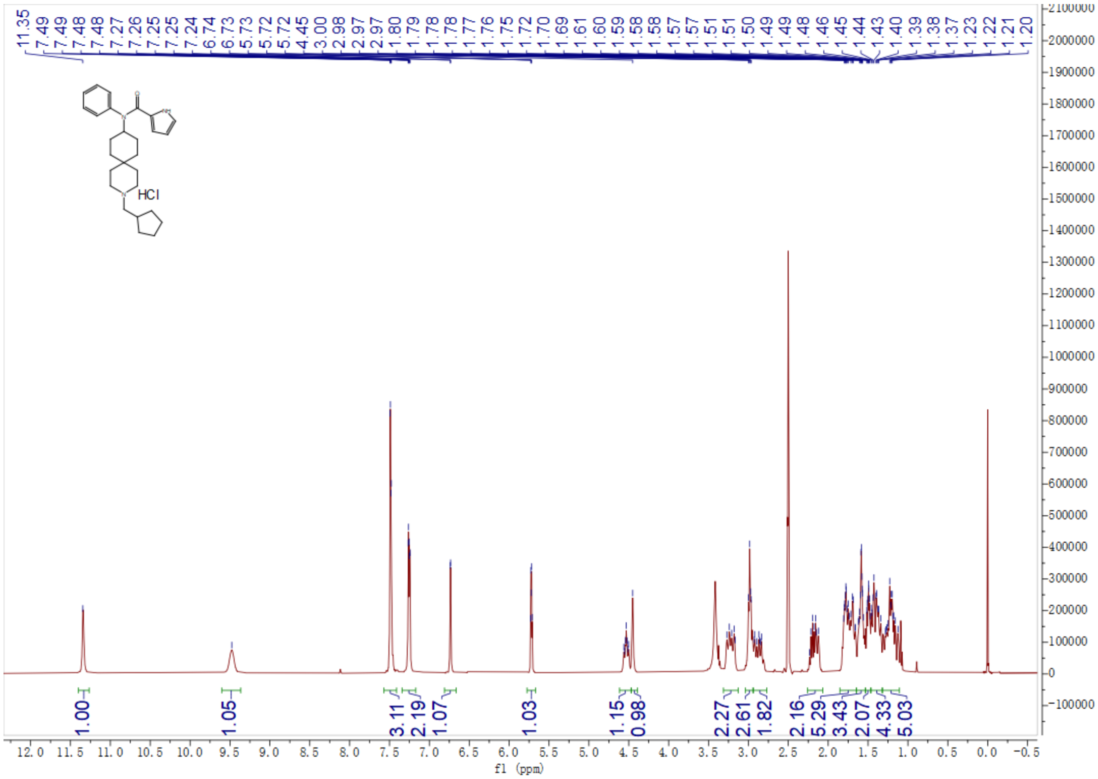

^{1}H NMR (400 MHz, DMSO-d_6) spectrum for compound **68**.

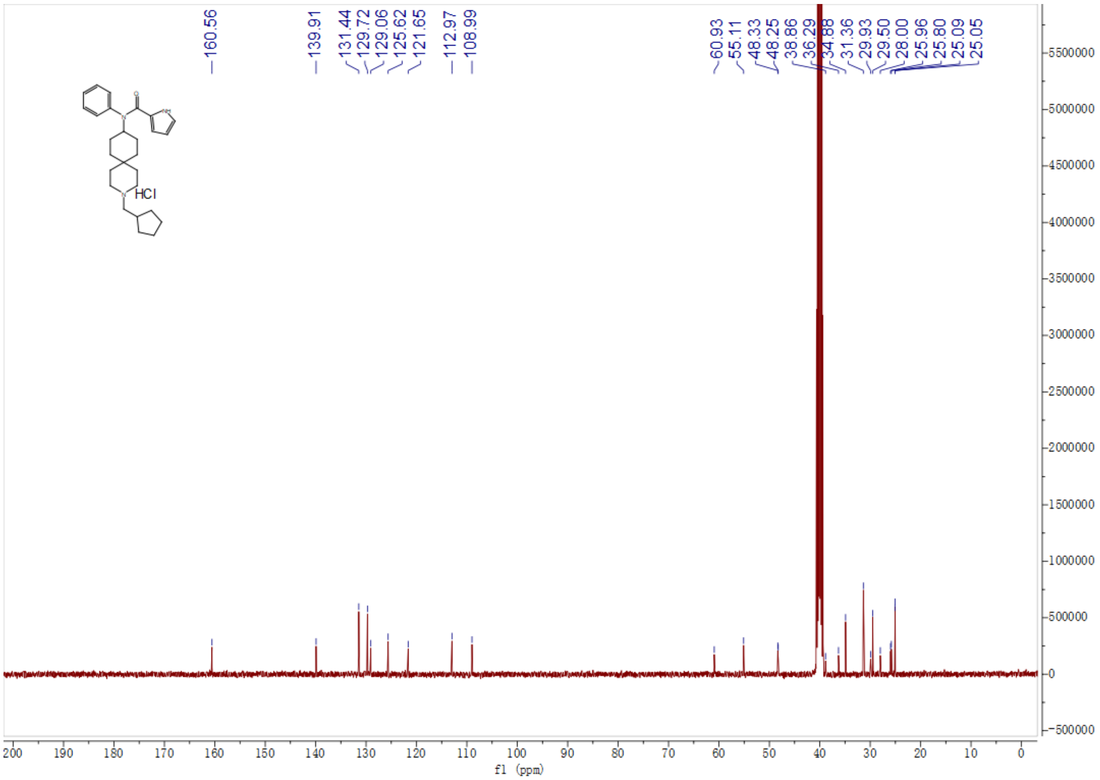

^{13}C NMR (100 MHz, DMSO-d_6) spectrum for compound **68**.

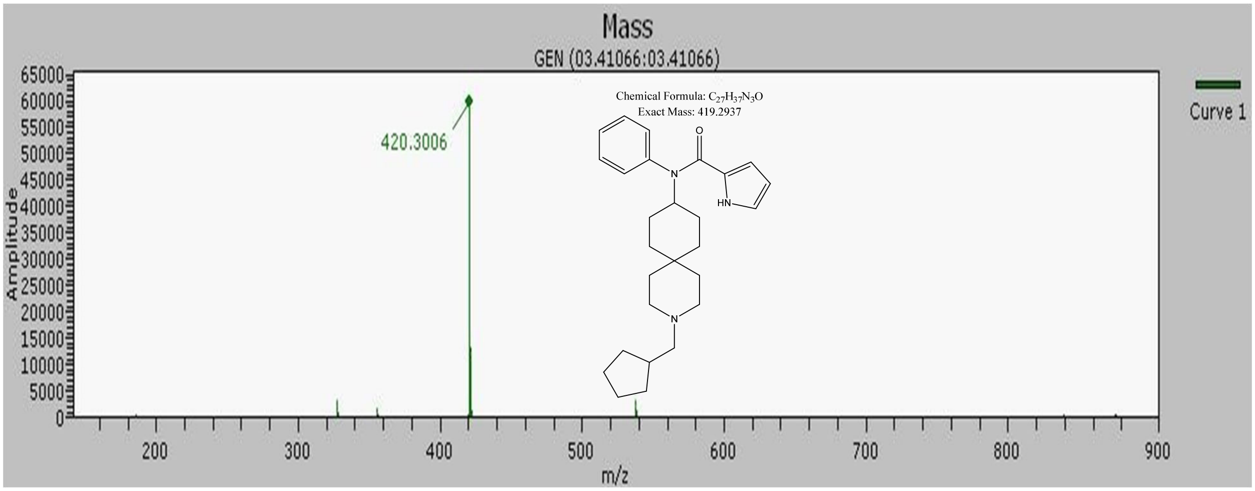

HRMS spectrum for compound **68**.

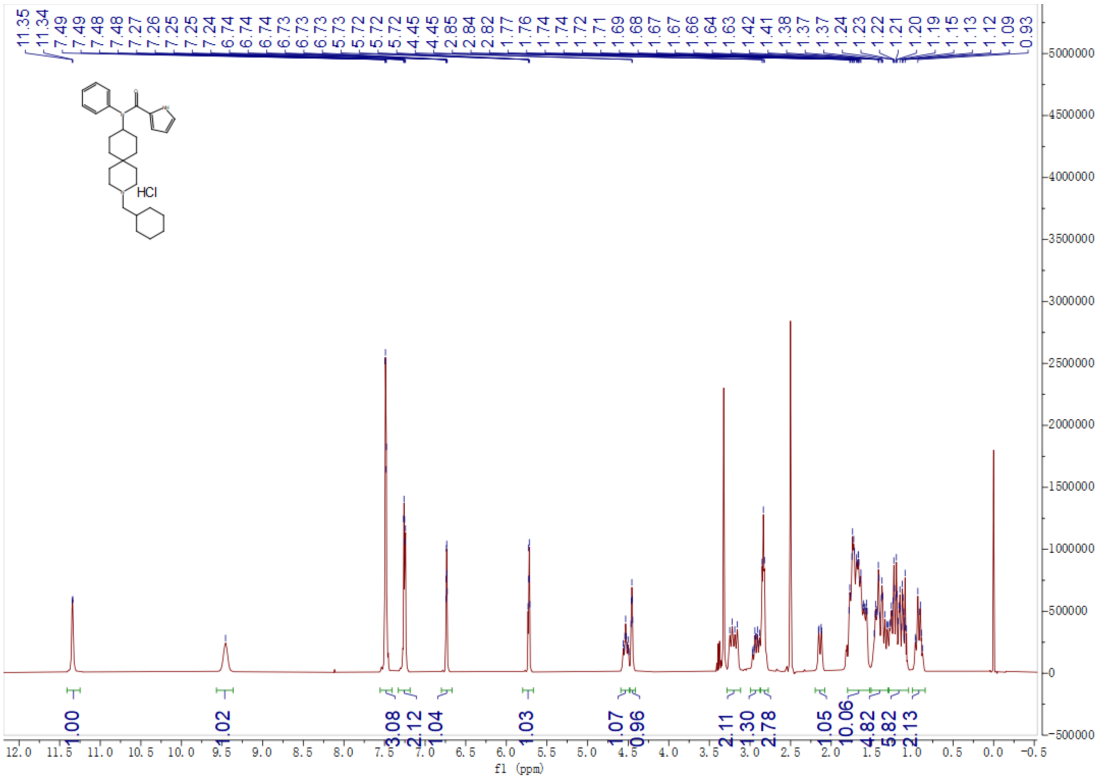

^{1}H NMR (400 MHz, DMSO-d_6) spectrum for compound **69**.

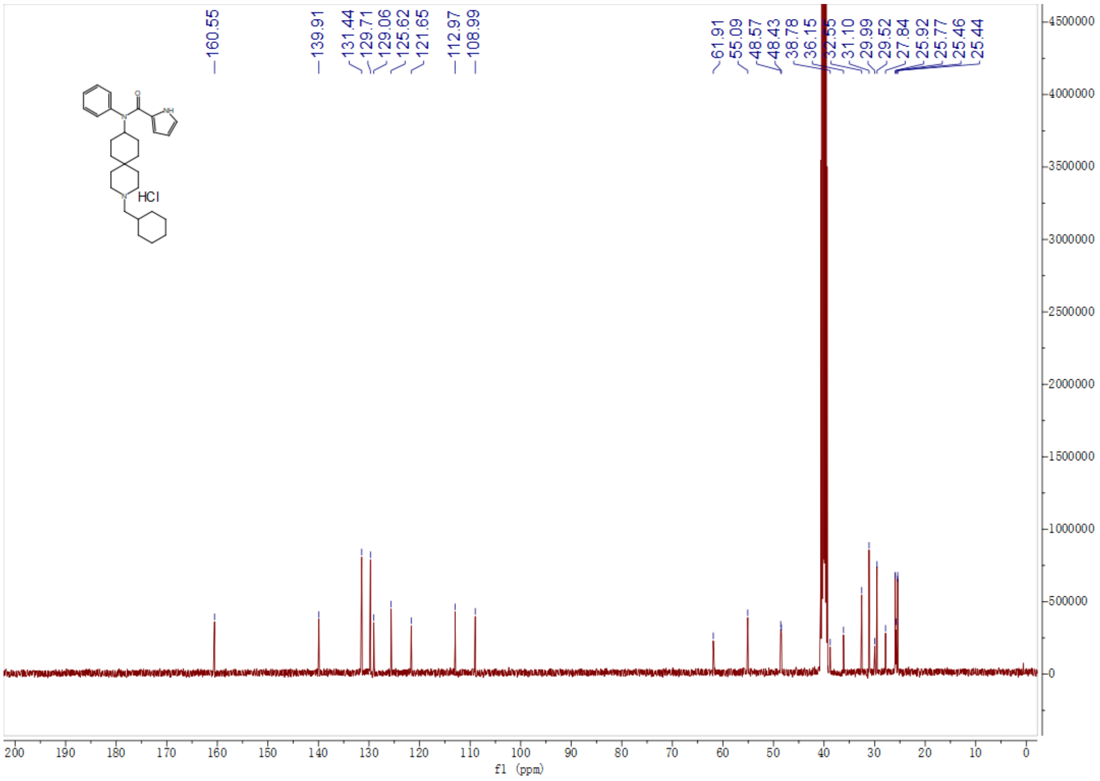

^{13}C NMR (100 MHz, DMSO-d_6) spectrum for compound **69**.

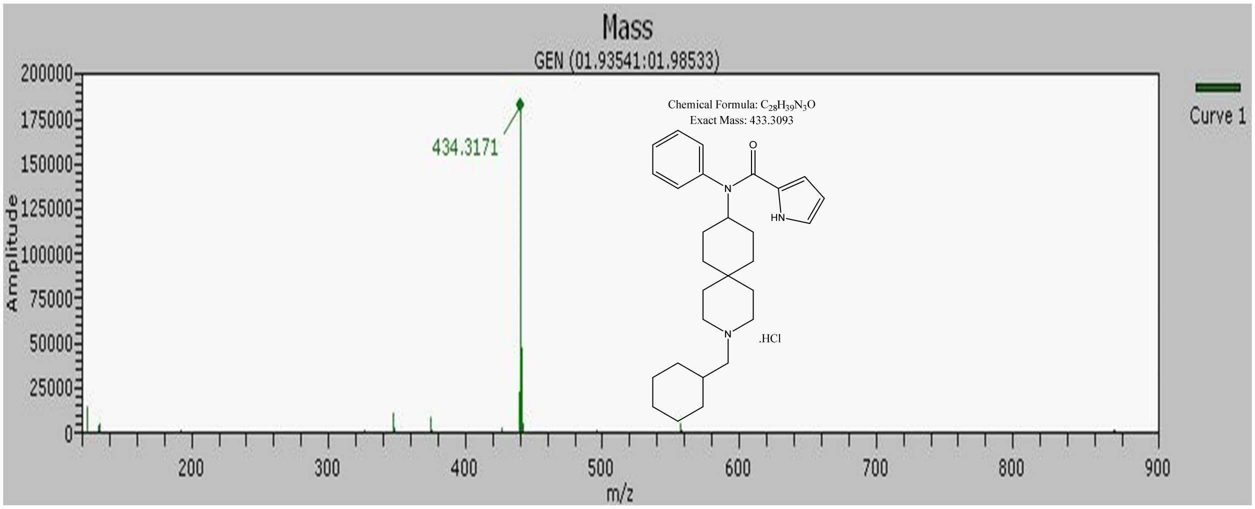

HRMS spectrum for compound **69**.

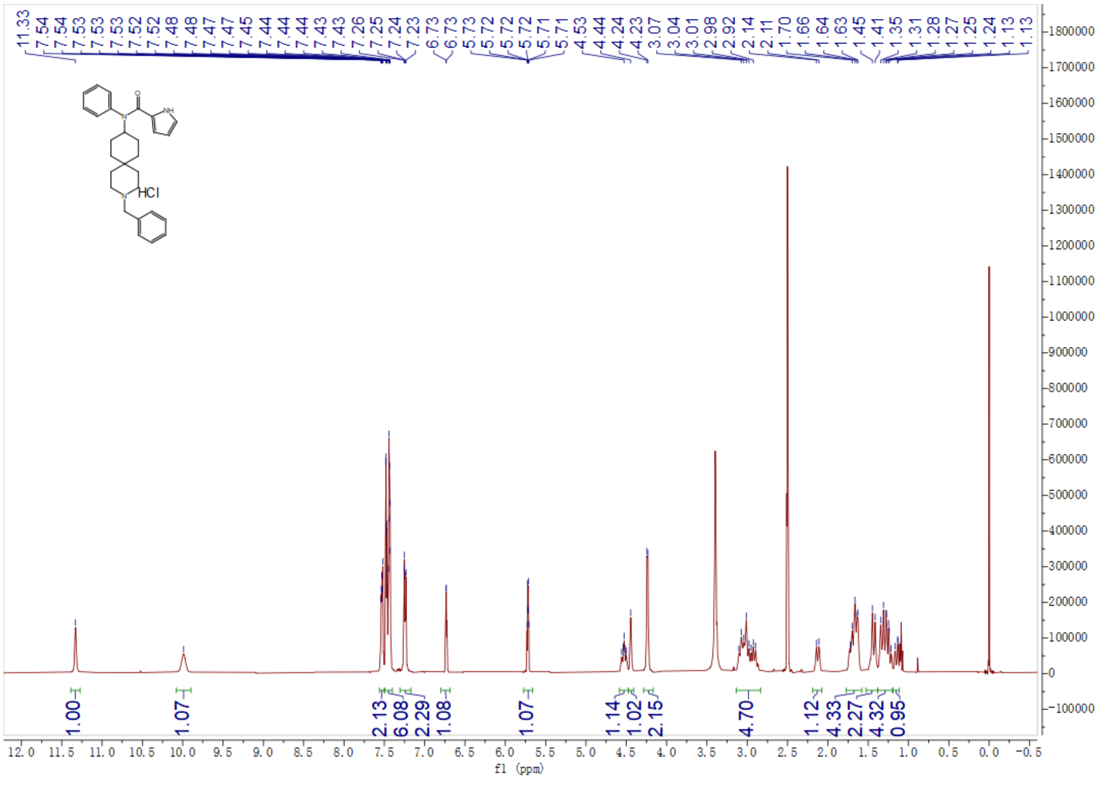

^{1}H NMR (400 MHz, DMSO-d_6) spectrum for compound **70**.

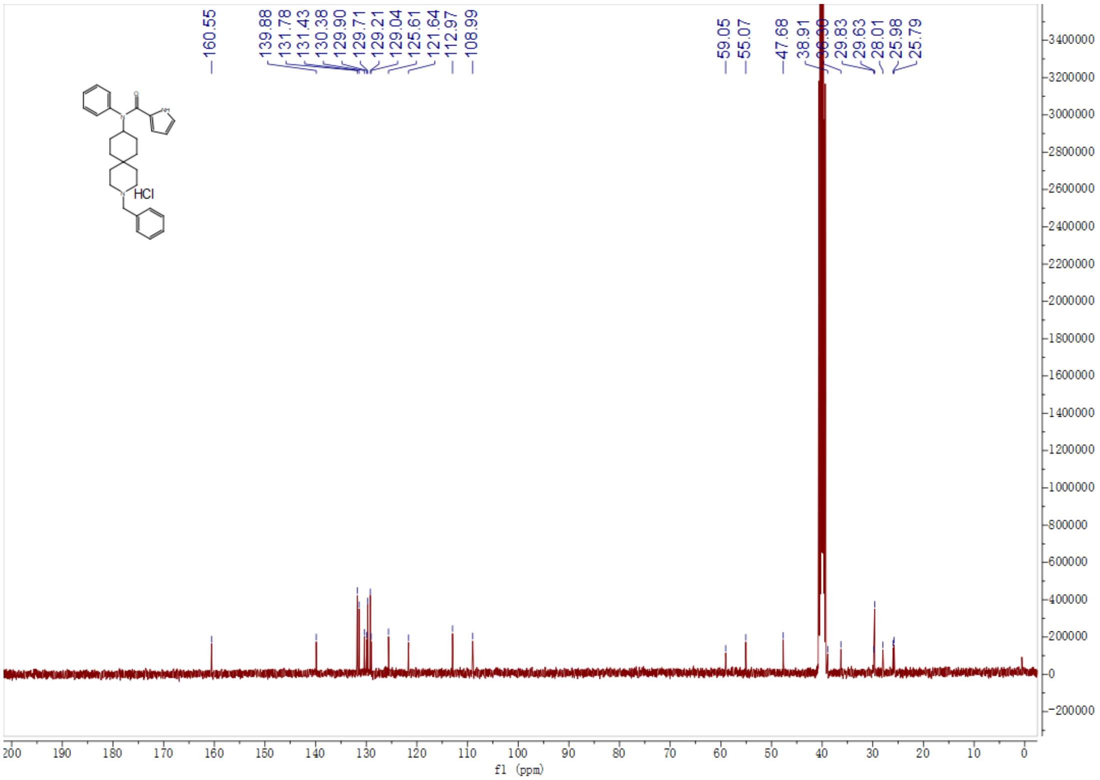

^{13}C NMR (100 MHz, DMSO-d_6) spectrum for compound **70**.

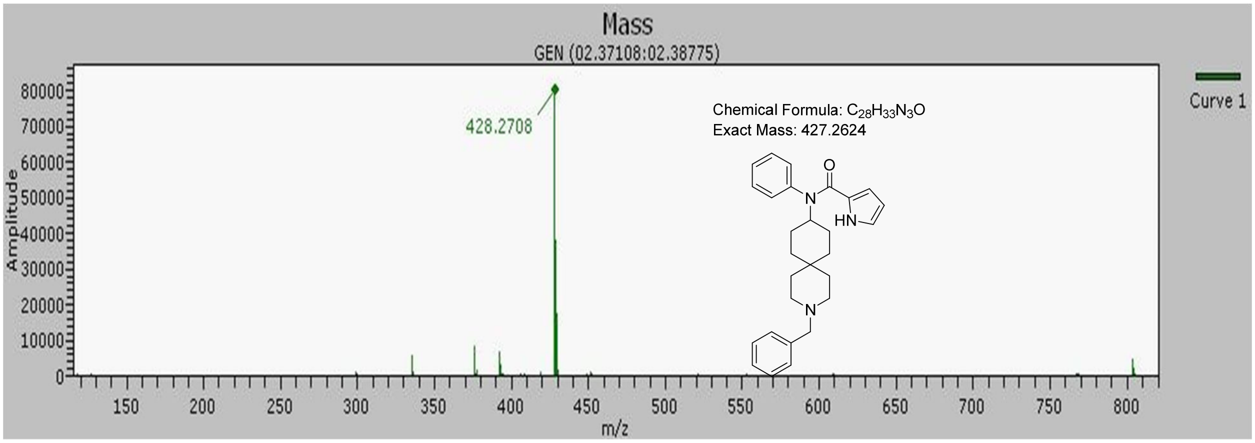

HRMS spectrum for compound **70**.

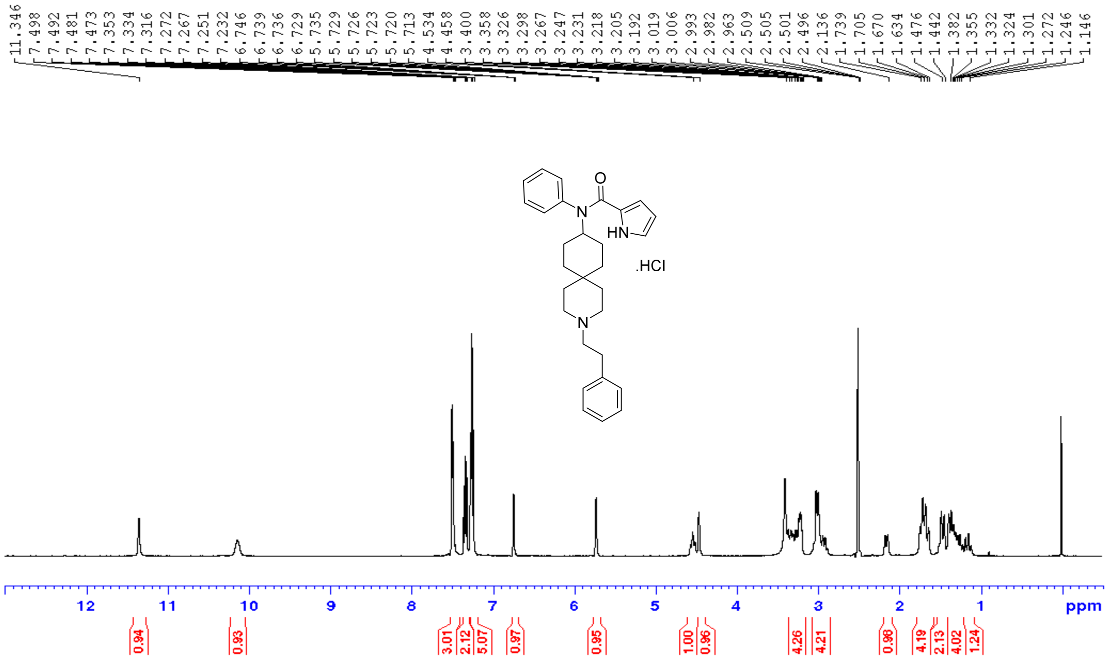

^{1}H NMR (400 MHz, DMSO-d_6) spectrum for compound **71**.

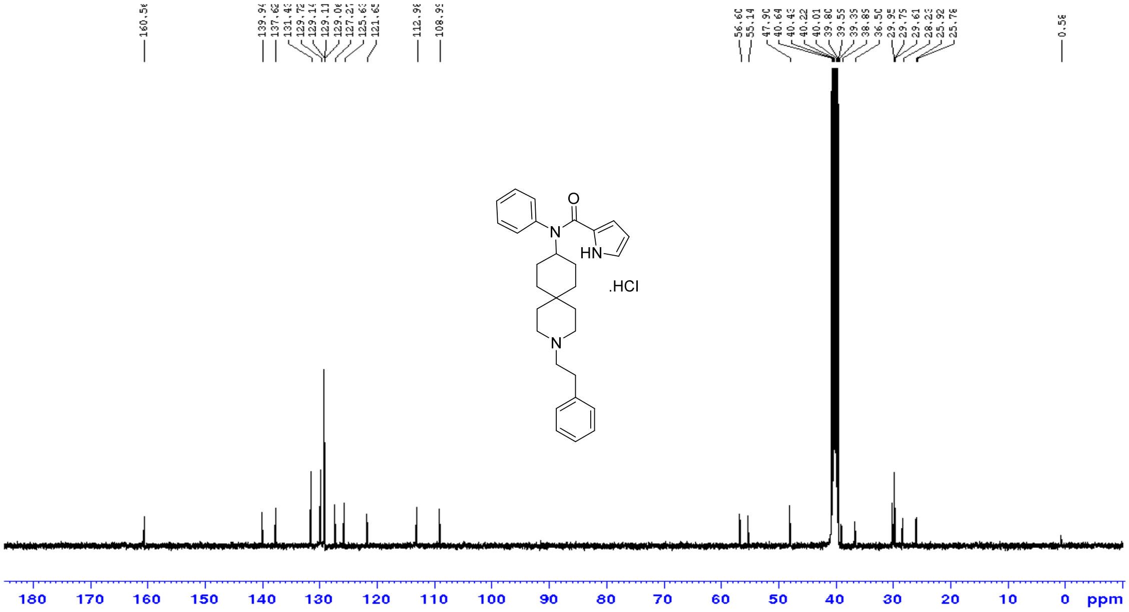

^{13}C NMR (100 MHz, DMSO-d_6) spectrum for compound **71**.

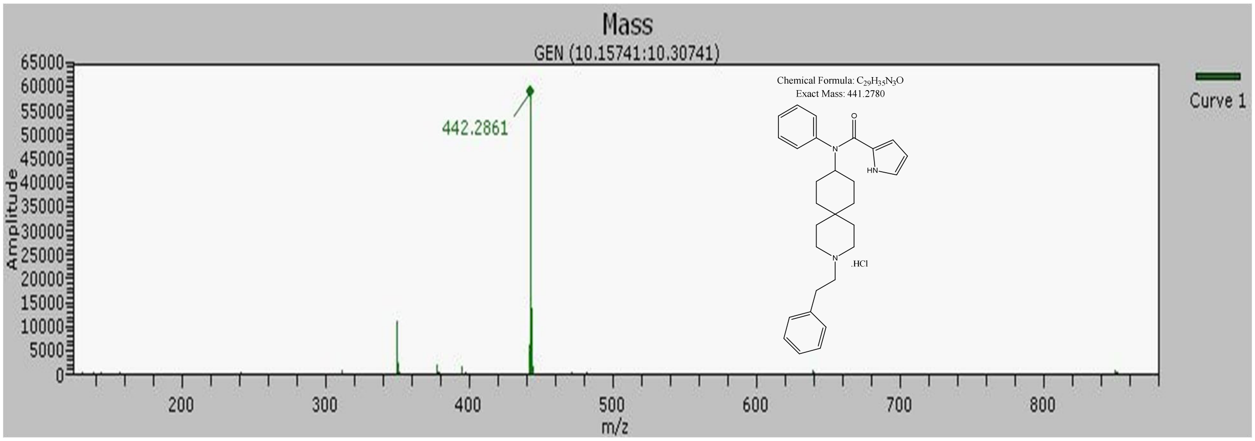

HRMS spectrum for compound **71**.

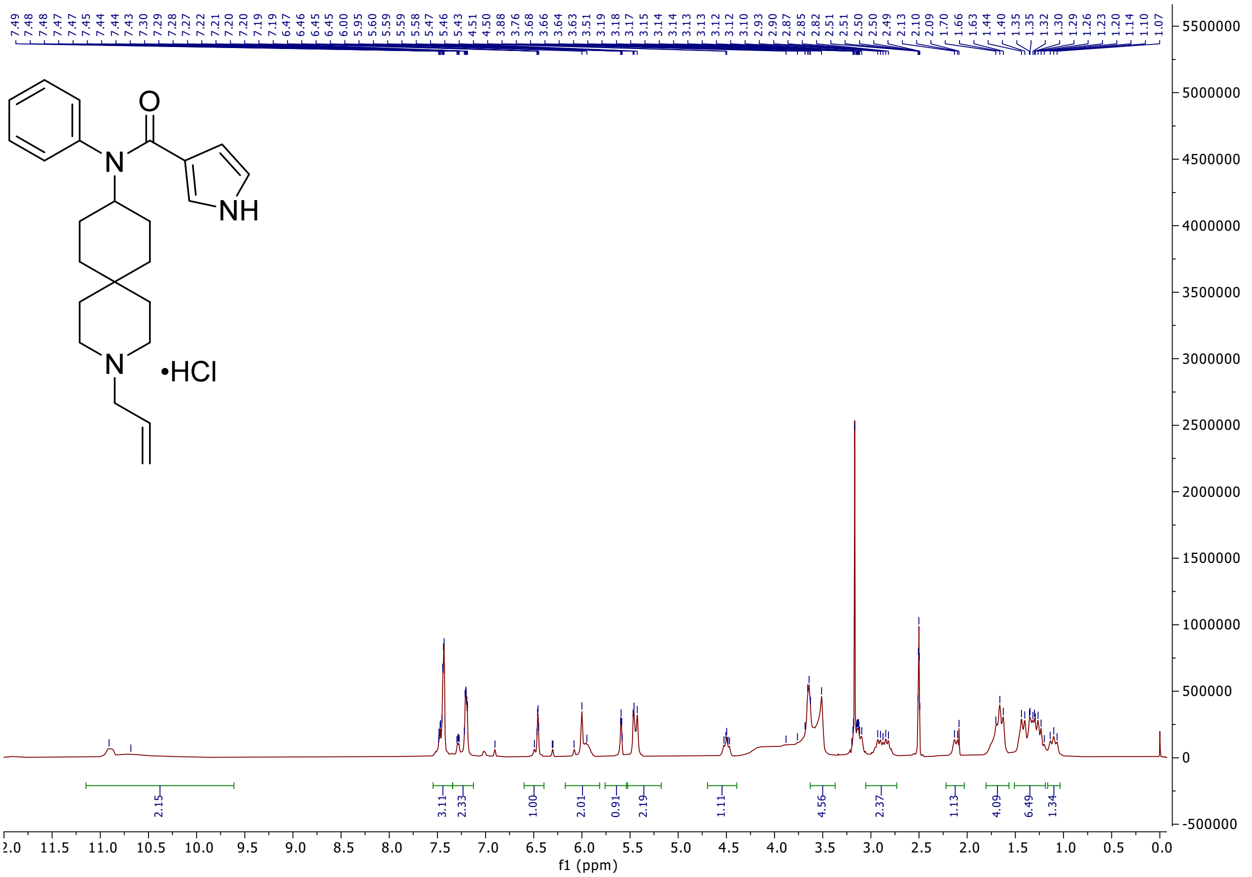

^{1}H NMR (400 MHz, DMSO-d_6) spectrum for compound **72**.

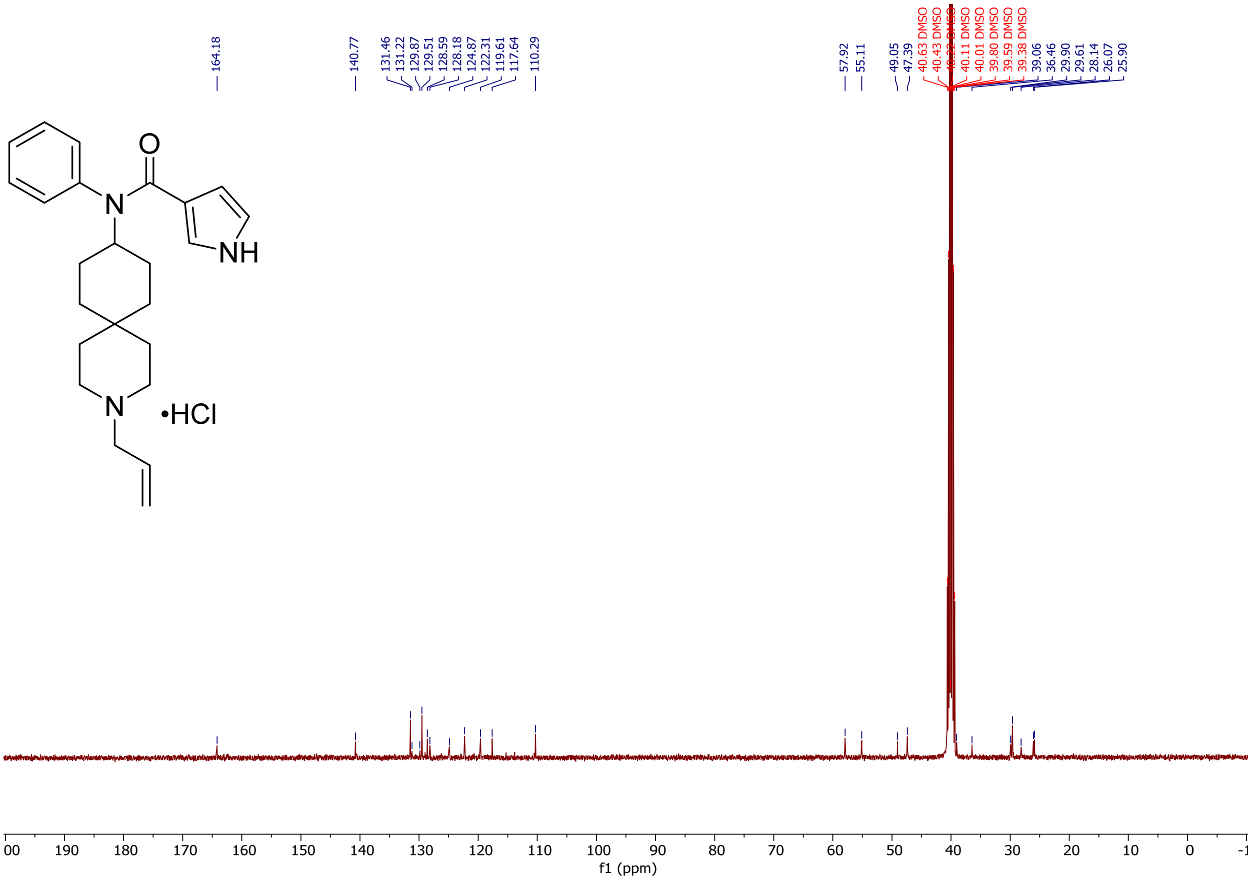

^{13}C NMR (100 MHz, DMSO-d_6) spectrum for compound **72**.

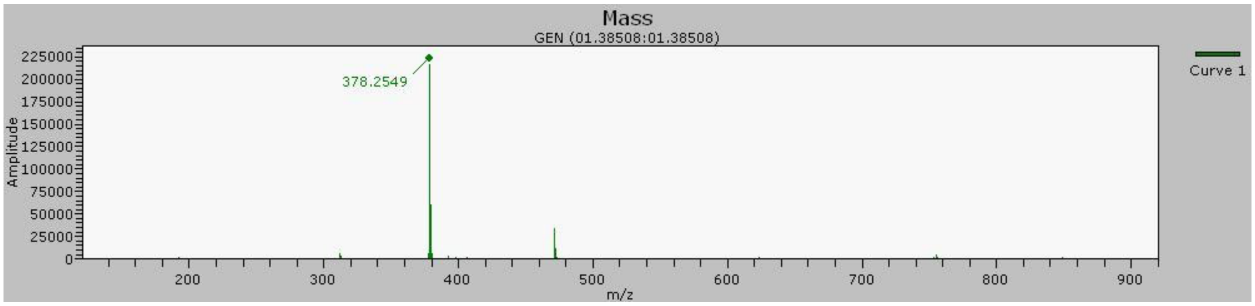

HRMS spectrum for compound **72**.

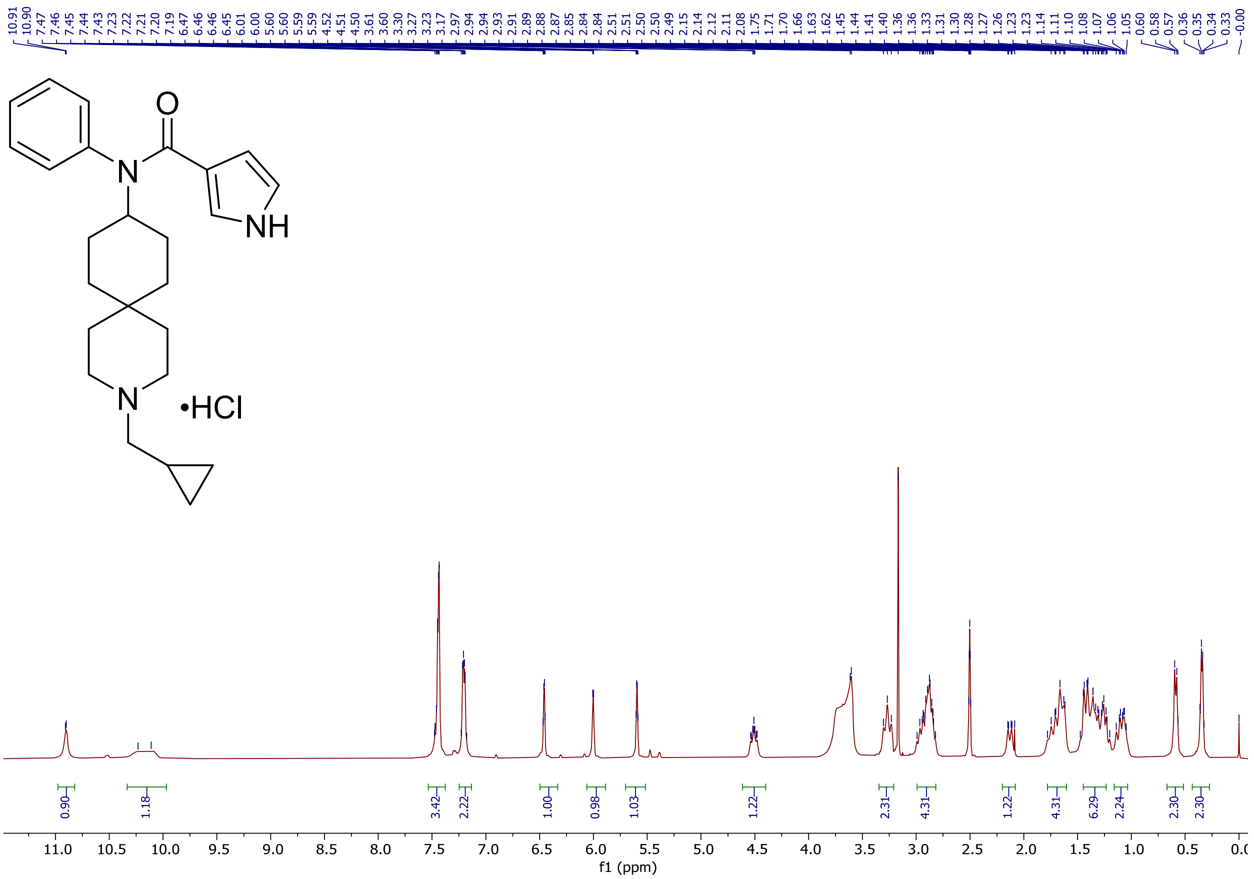

^{1}H NMR (400 MHz, DMSO-d_6) spectrum for compound **73**.

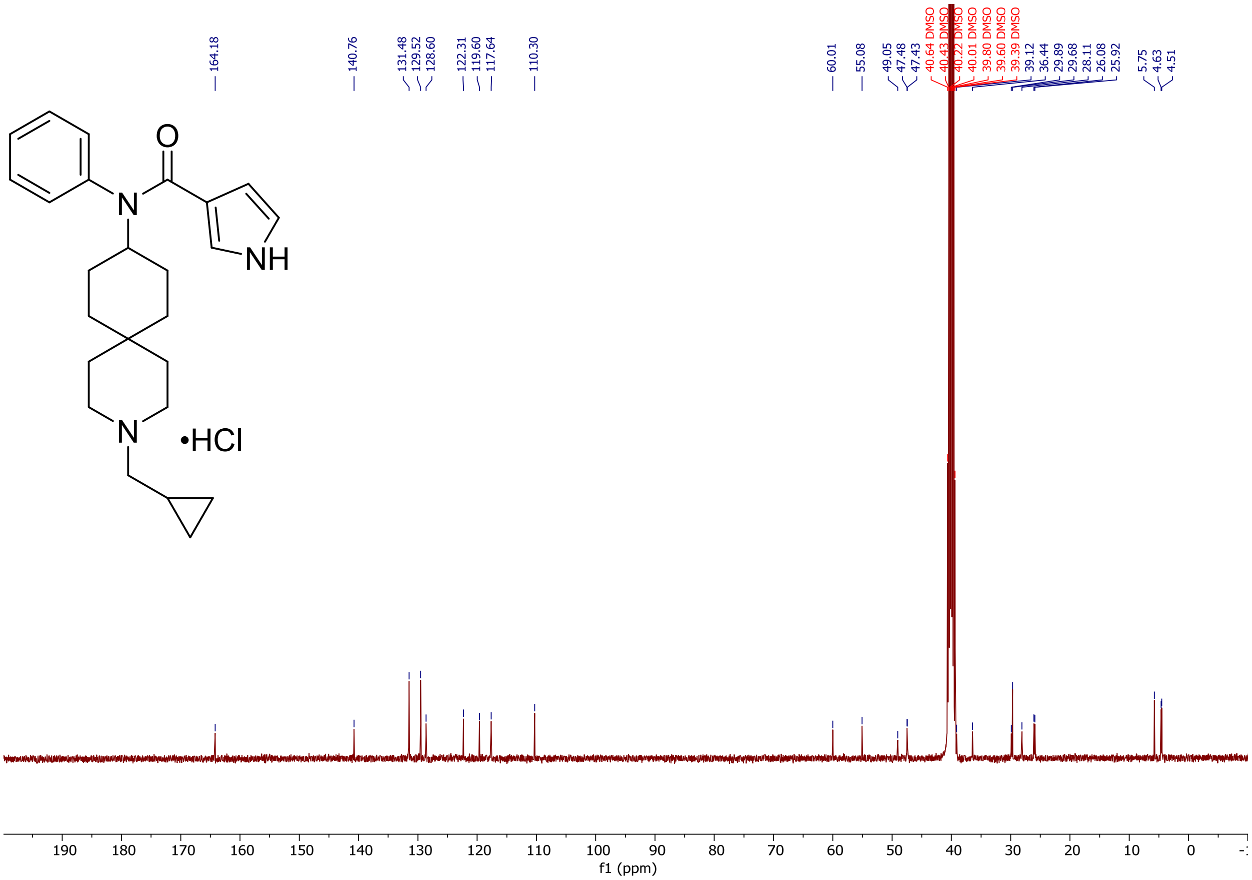

^{13}C NMR (100 MHz, DMSO-d_6) spectrum for compound **73**.

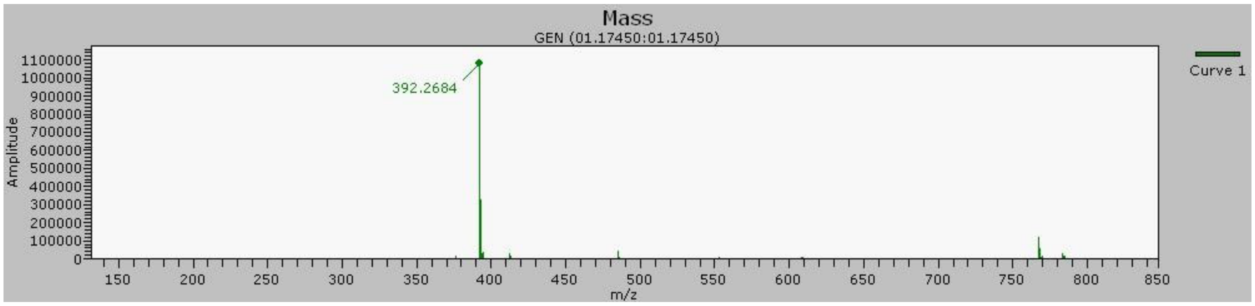

HRMS spectrum for compound **73**.

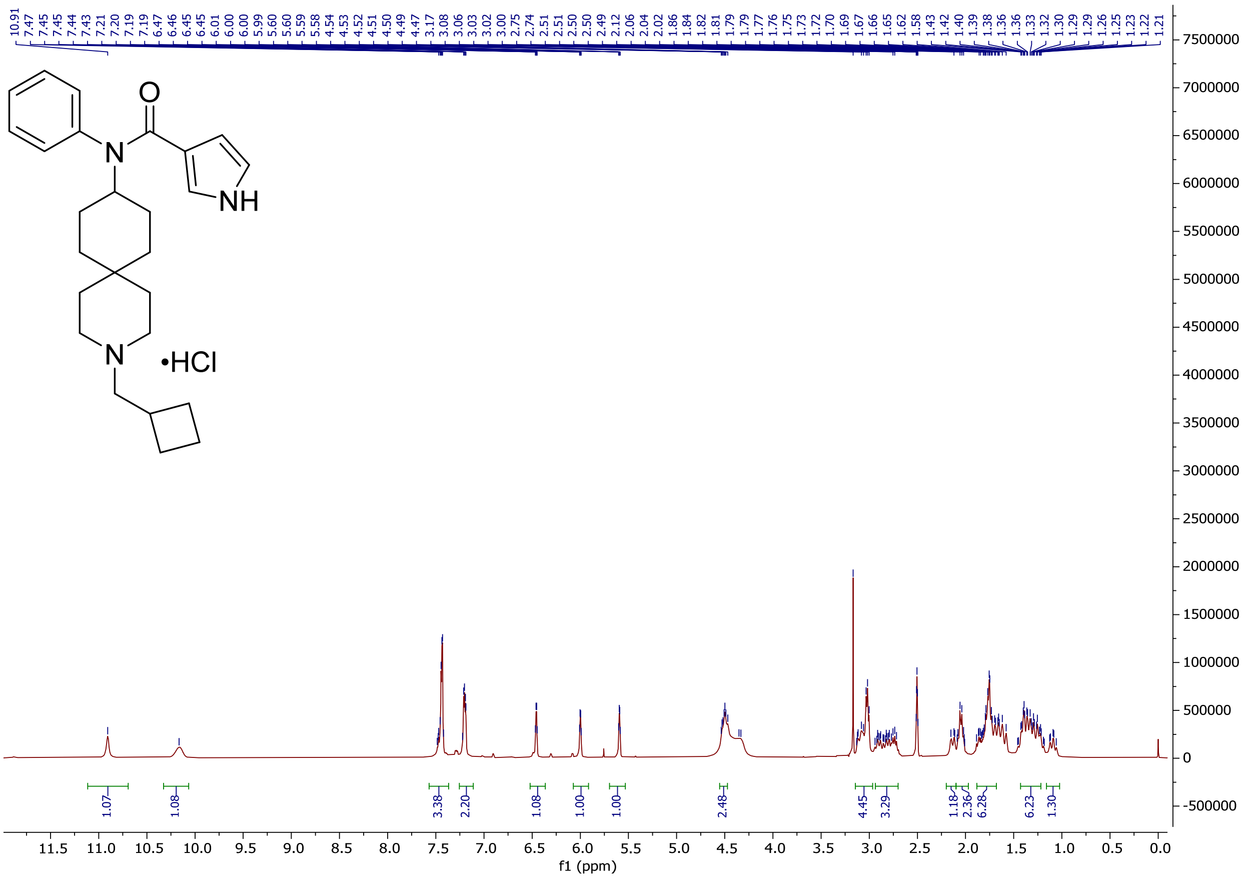

^{1}H NMR (400 MHz, DMSO-d_6) spectrum for compound **74**.

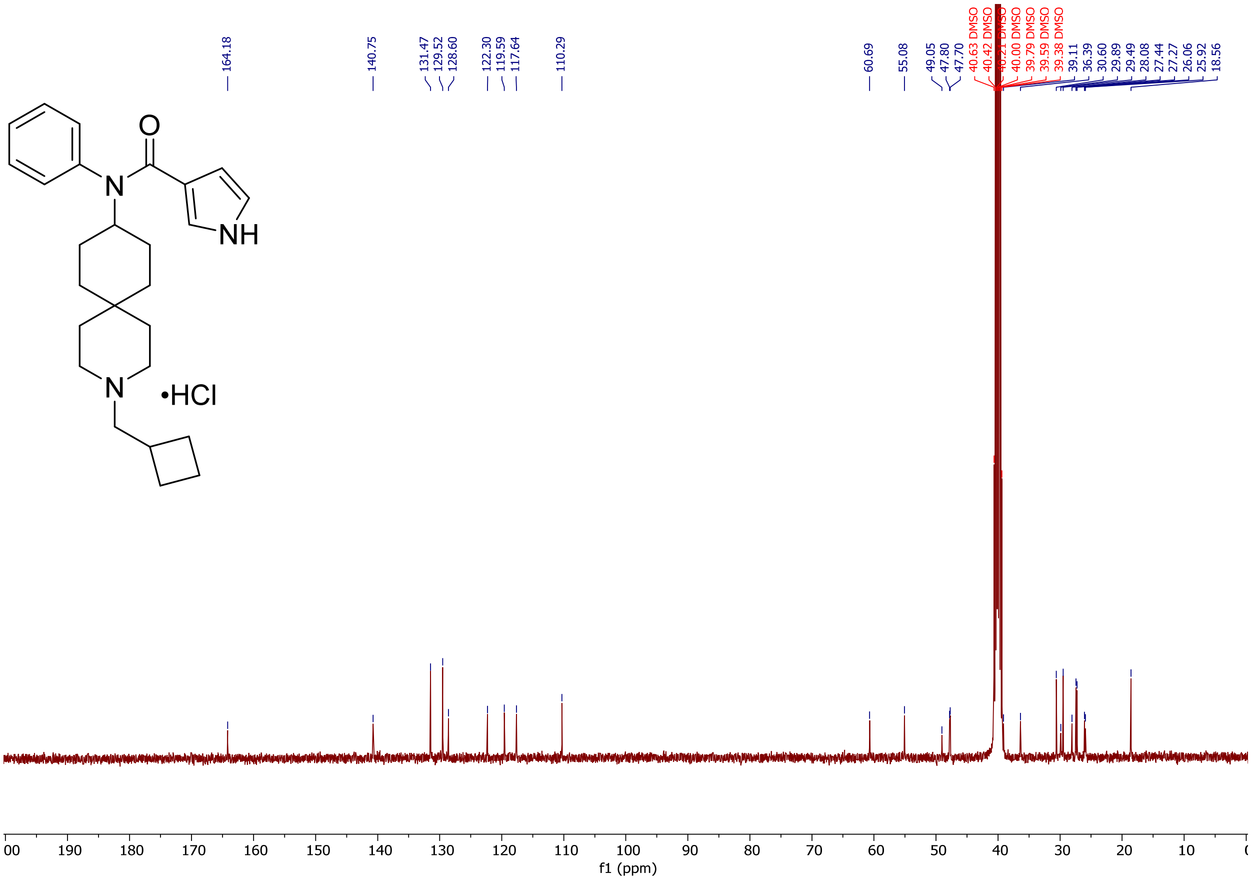

^{13}C NMR (100 MHz, DMSO-d_6) spectrum for compound **74**.

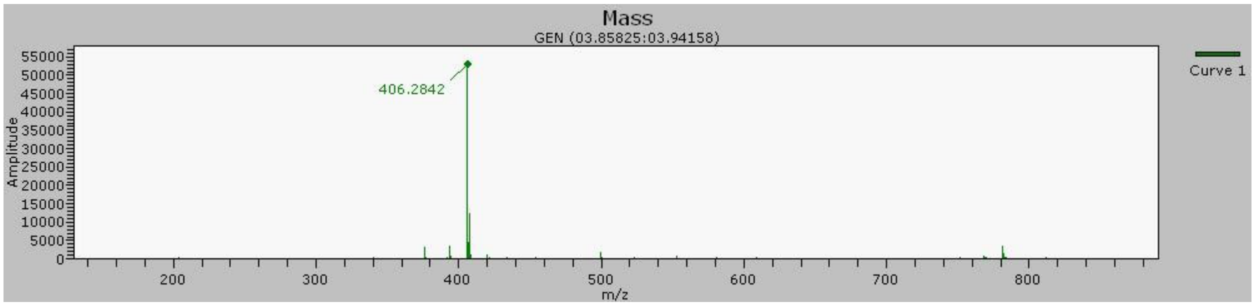

HRMS spectrum for compound **74**.

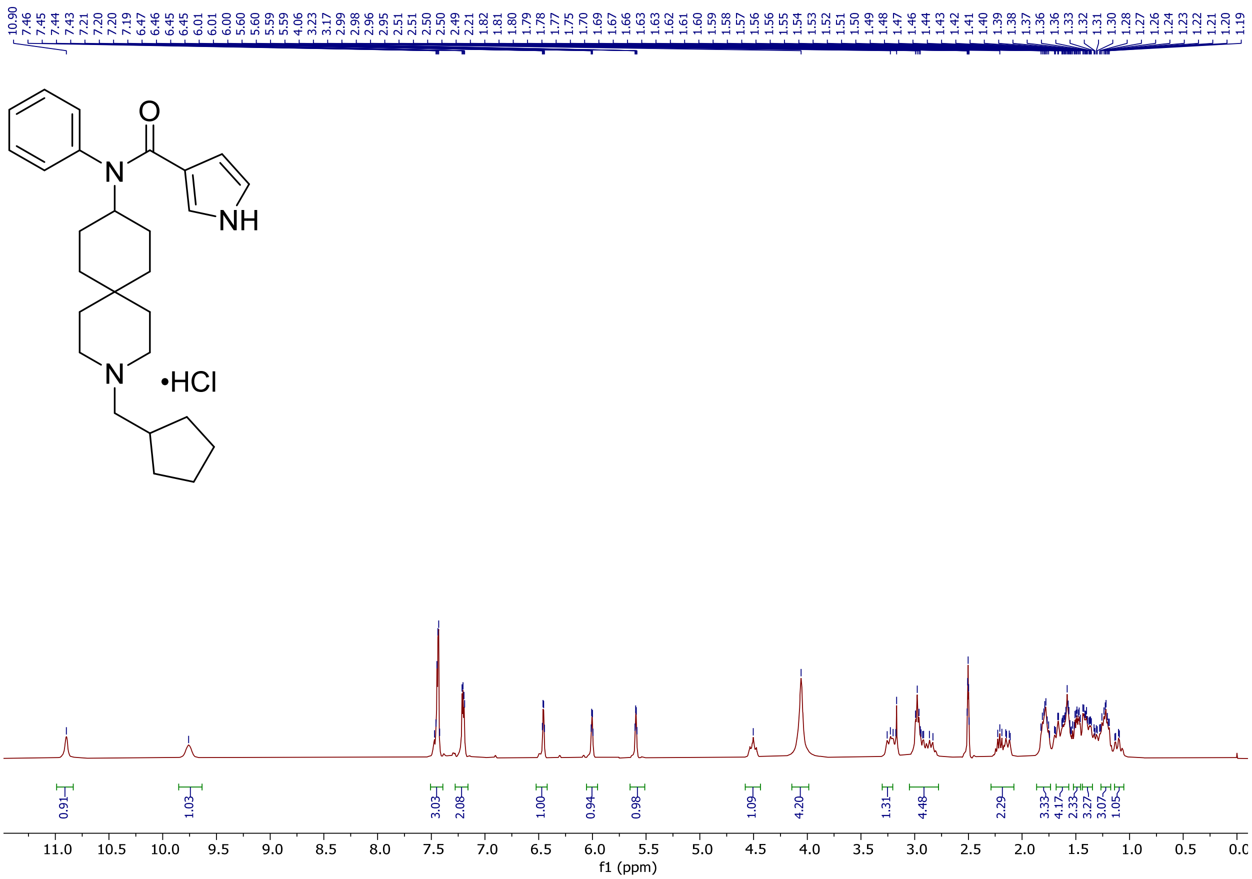

^{1}H NMR (400 MHz, DMSO-d_6) spectrum for compound **75**.

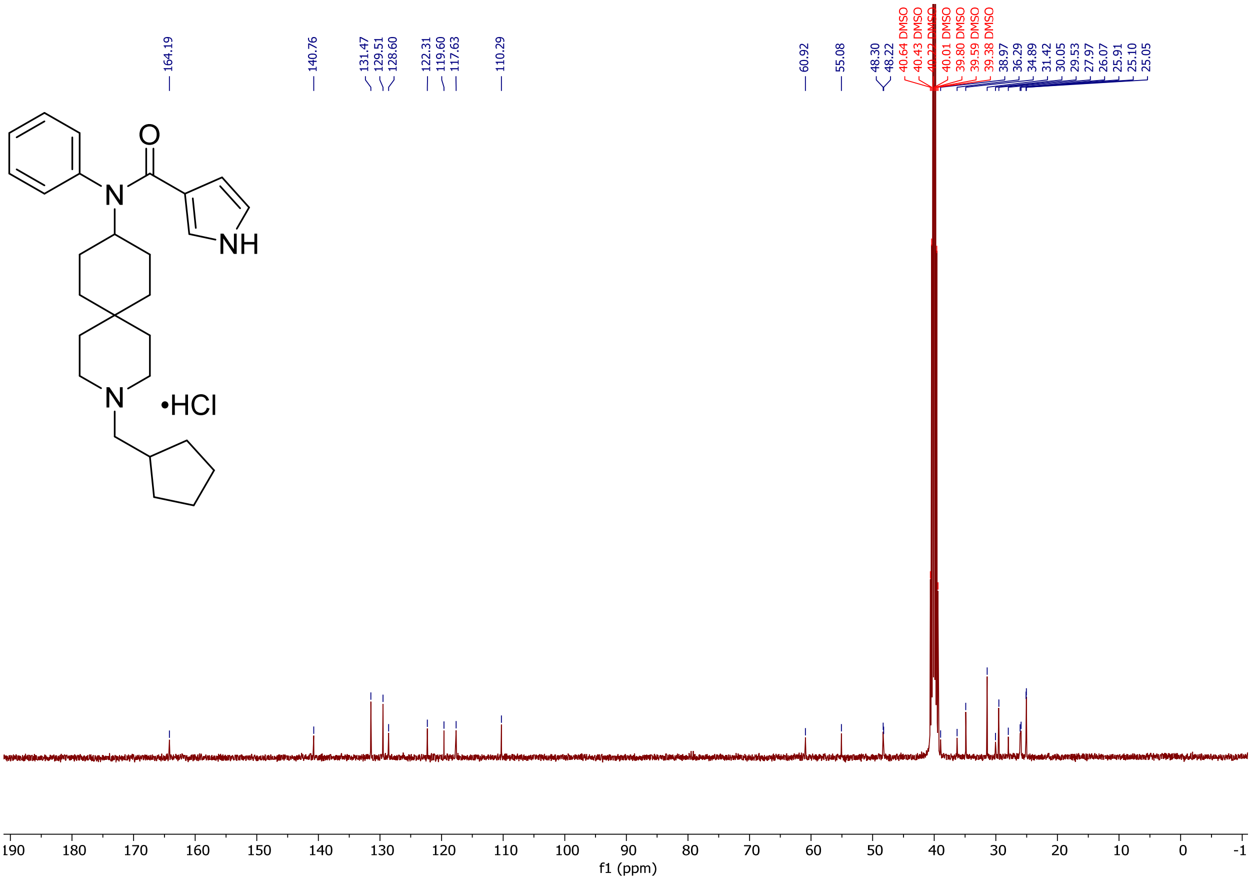

^{13}C NMR (100 MHz, DMSO-d_6) spectrum for compound **75**.

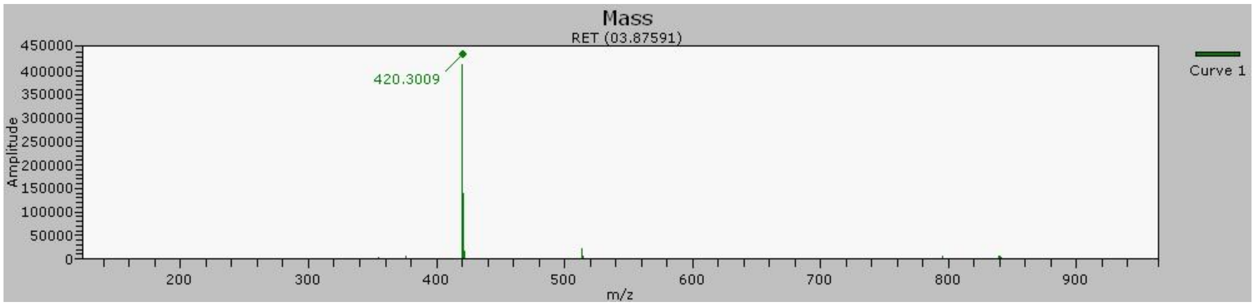

HRMS spectrum for compound **75**.

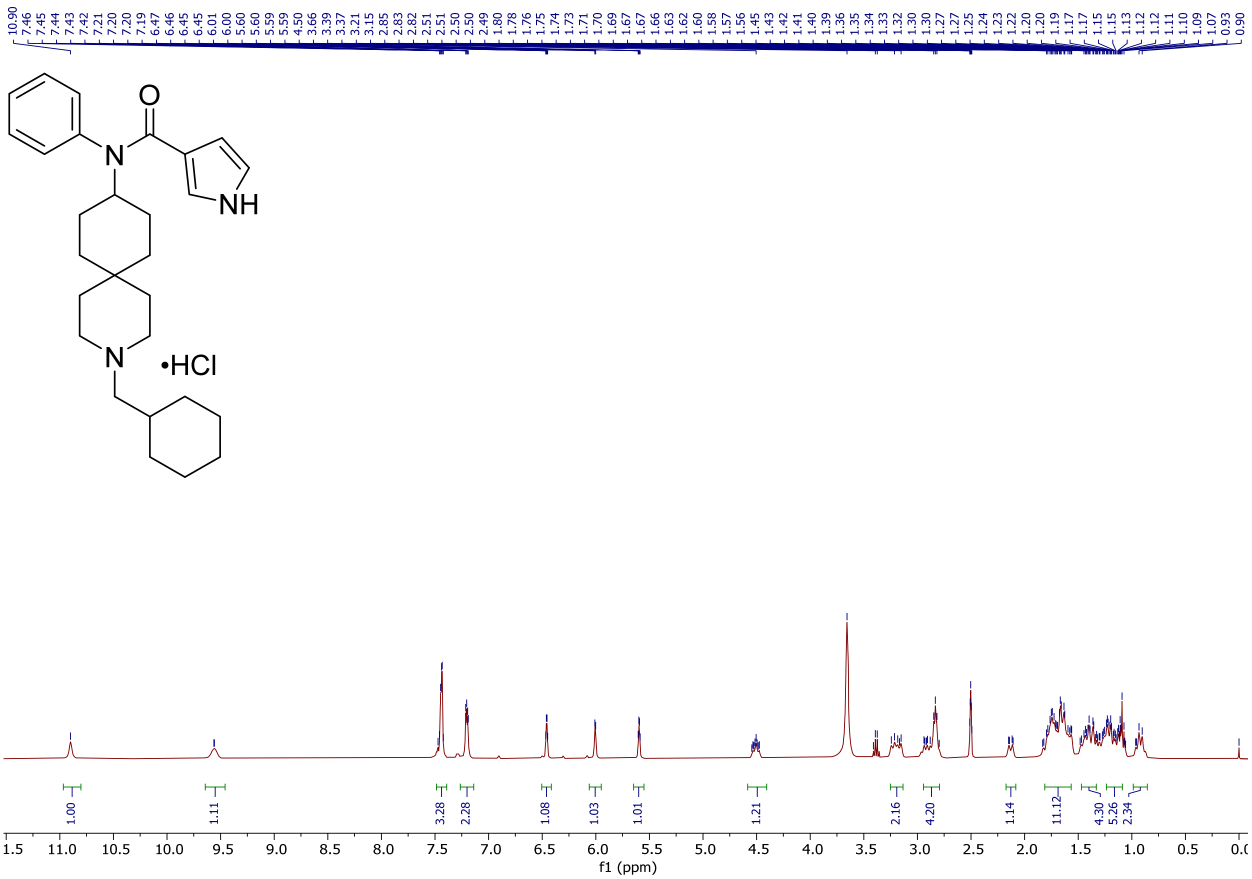

^{1}H NMR (400 MHz, DMSO-d_6) spectrum for compound **76**.

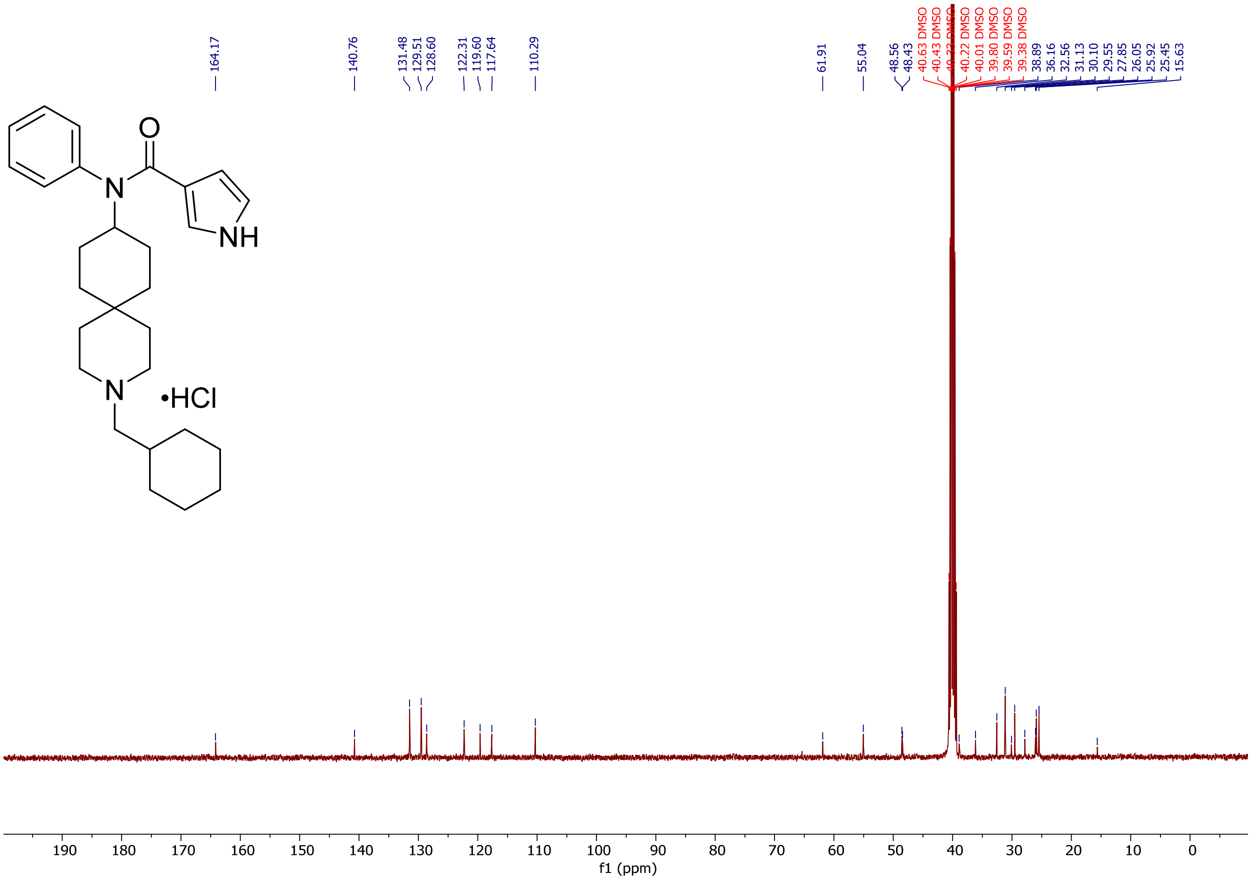

^{13}C NMR (100 MHz, DMSO-d_6) spectrum for compound **76**.

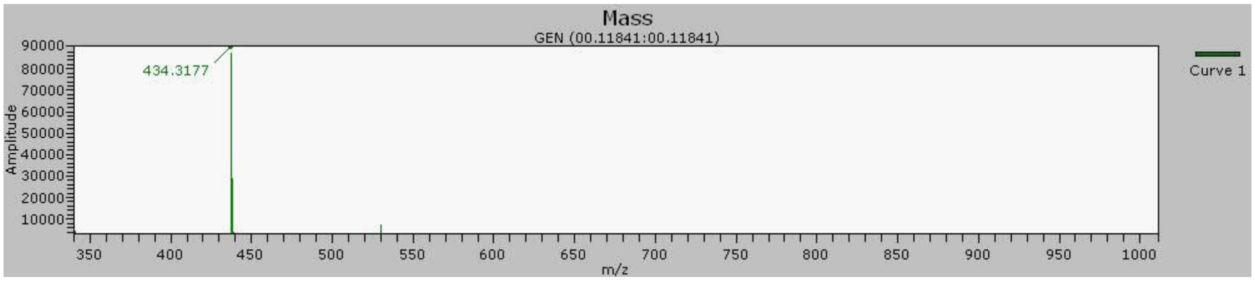

HRMS spectrum for compound **76**.

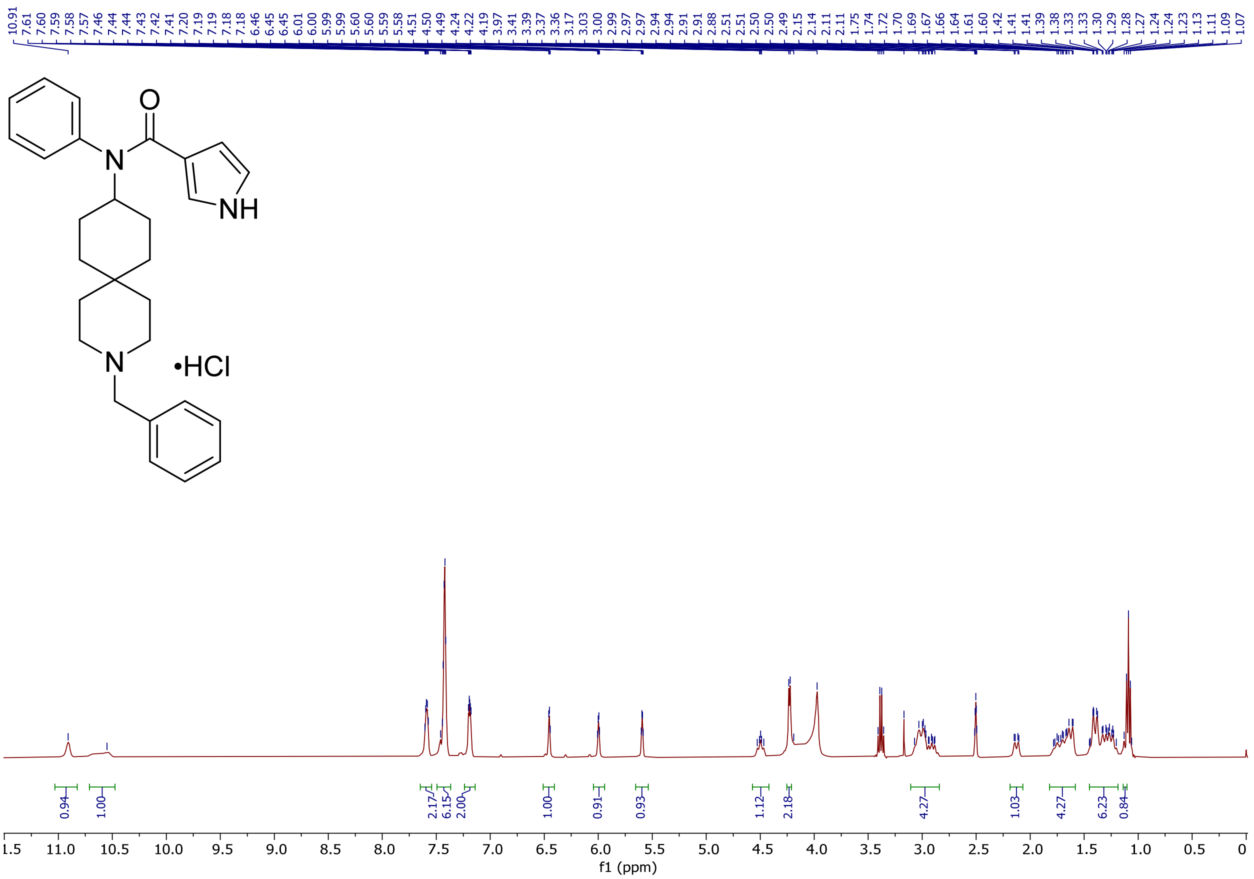

^{1}H NMR (400 MHz, DMSO-d_6) spectrum for compound **77**.

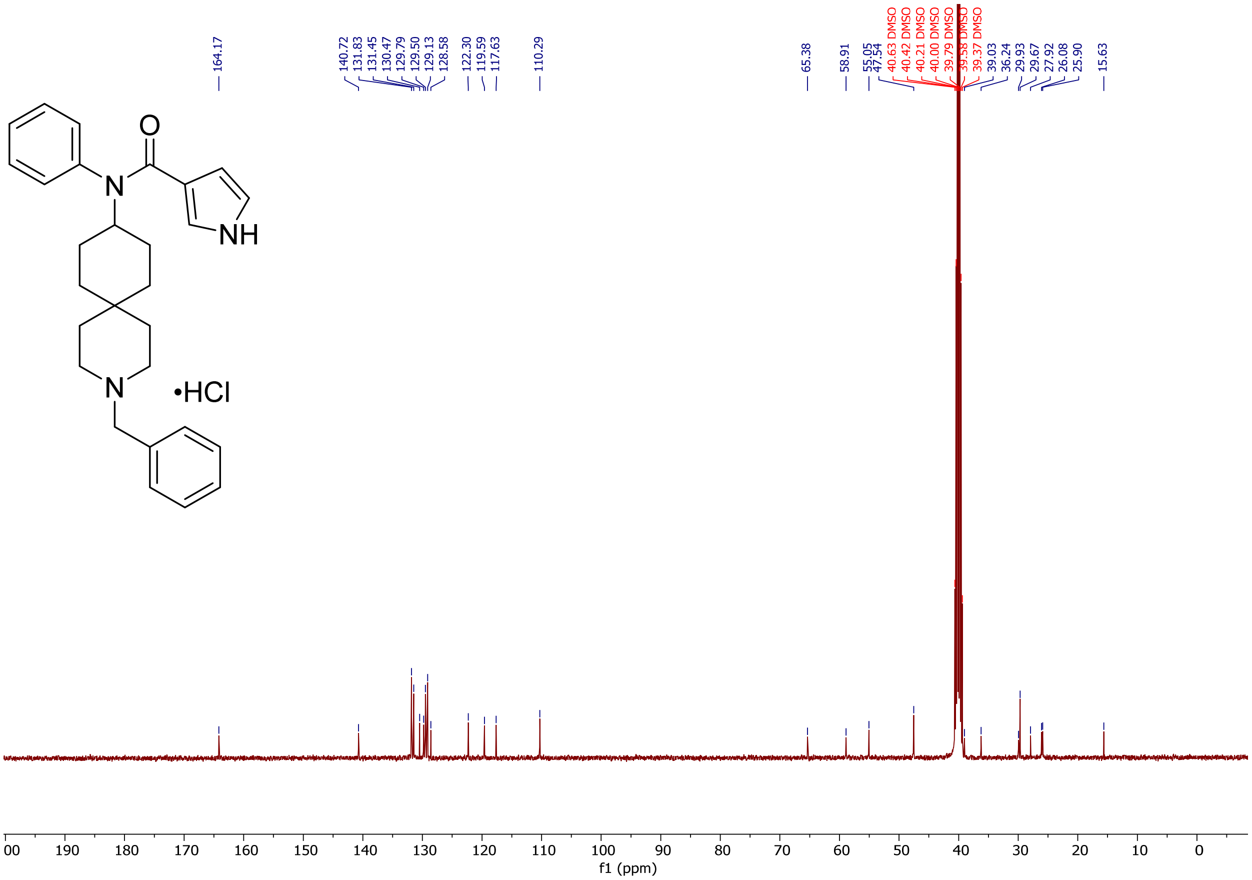

^{13}C NMR (100 MHz, DMSO-d_6) spectrum for compound **77**.

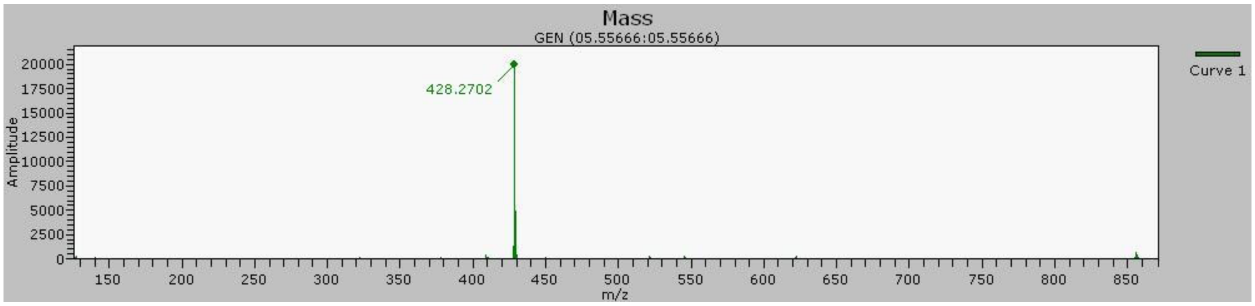

HRMS spectrum for compound **77**.

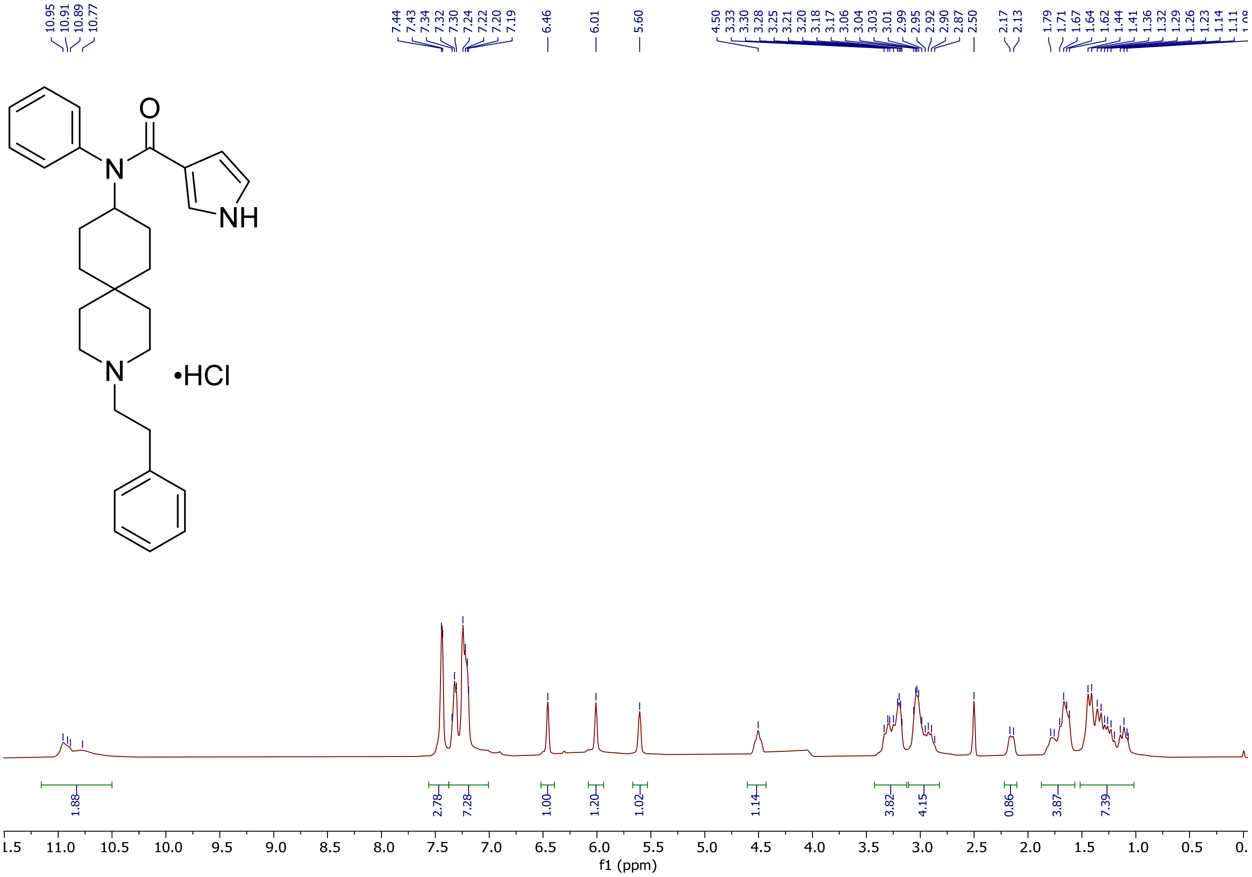

^{1}H NMR (400 MHz, DMSO-d_6) spectrum for compound **78**.

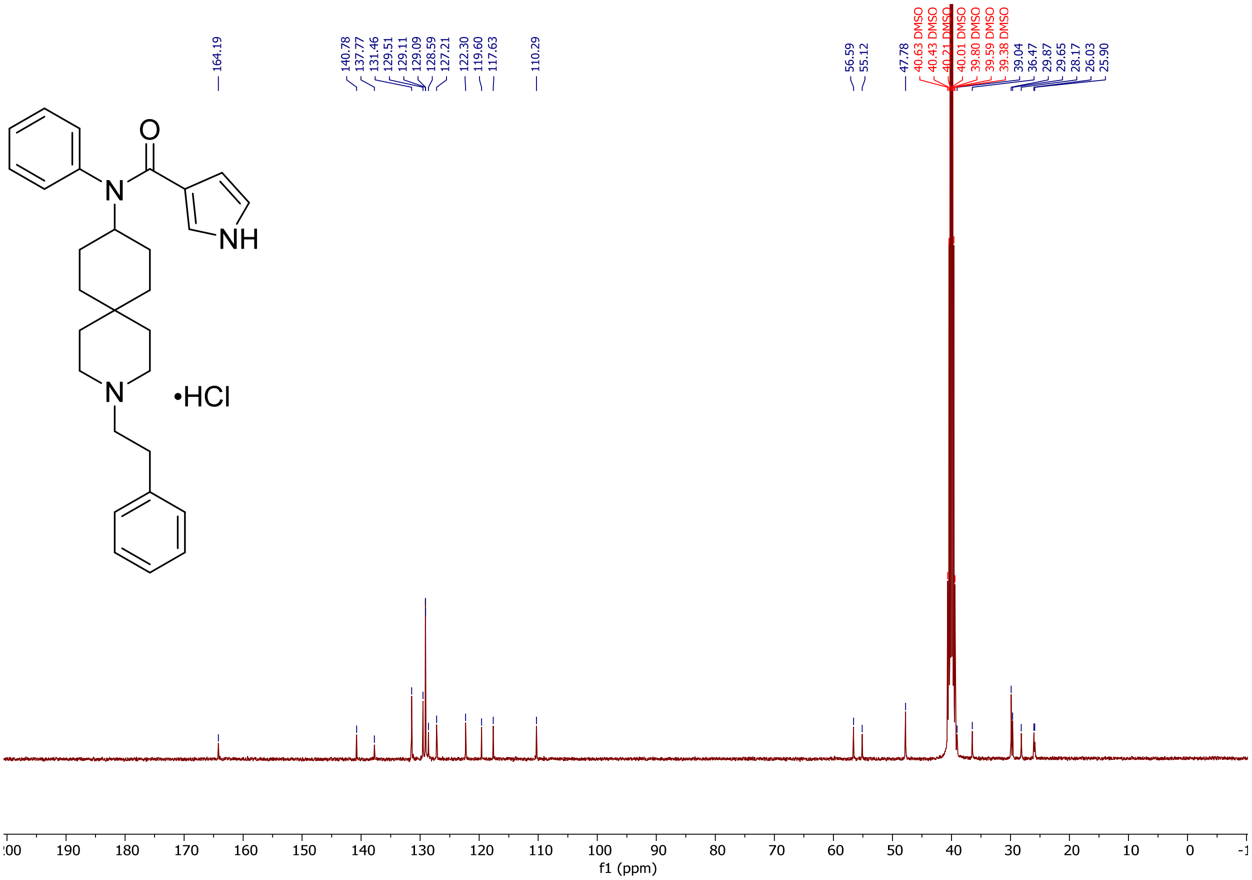

^{13}C NMR (100 MHz, DMSO-d_6) spectrum for compound **78**.

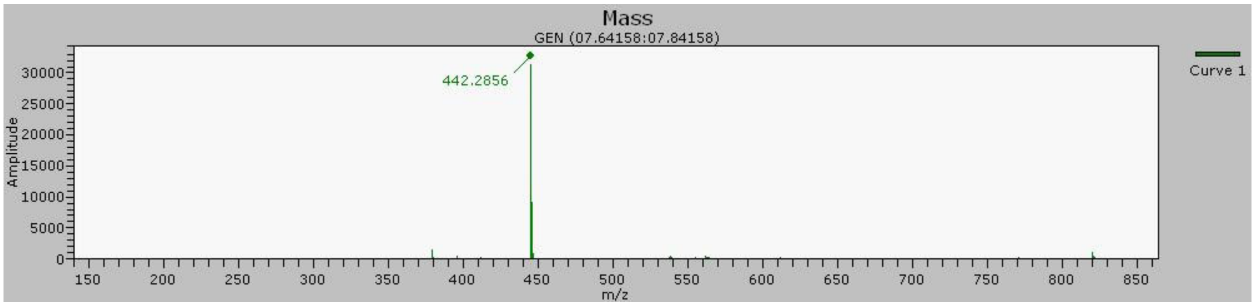

HRMS spectrum for compound **78**.

5. HPLC data and spectrum.

4.1 HPLC data and spectrum for compounds **1-36**.

HPLC System: Waters Arc HPLC
Column: XBridge™ C_{18} 3.5 µm (4.6 x 50 mm)
Sample Concentration: 0.75 mg/mL
Injection Solvent: Acetonitrile
Injection Volume: 5 µL
Single Wavelength: 210 nm
Run time: 10 min

Table S1. HPLC data for compounds **1-36**.

Compound	Retention Time (min)	Purity (%)	Mobile Phase	Flow Rate
1	3.235	100.00	80:20 (TFAH_2O:ACN)	0.8mL/min
2	4.102	100.00	80:20 (TFAH_2O:ACN)	0.8mL/min
3	3.705	100.00	80:20 (TFAH_2O:ACN)	1.5mL/min
4	2.427	100.00	70:30 (TFAH_2O:ACN)	0.8mL/min
5	3.532	98.79	70:30 (TFAH_2O:ACN)	0.8mL/min
6	2.213	100.00	70:30 (TFAH_2O:ACN)	0.8mL/min
7	2.292	96.21	80:20 (TFAH_2O:ACN)	1.5mL/min
8	2.907	99.88	80:20 (TFAH_2O:ACN)	1.5mL/min
9	2.117	100.00	70:30 (TFAH2O:ACN)	0.8mL/min
10	2.845	100.00	70:30 (TFAH_2O:ACN)	0.8mL/min
11	4.202	100.00	70:30 (TFAH_2O:ACN)	0.8mL/min
12	2.640	100.00	70:30 (TFAH_2O:ACN)	0.8mL/min
13	3.913	98.65	80:20 (TFAH_2O:ACN)	1.5mL/min
14	2.122	100.00	70:30 (TFAH_2O:ACN)	0.8mL/min
15	2.945	100.00	70:30 (TFAH_2O:ACN)	0.8mL/min

16	4.068	97.10	70:30 (TFAH2O:ACN)	0.8mL/min
17	2.025	100.00	60:40 (TFAH2O:ACN)	0.8mL/min
18	3.773	99.85	70:30 (TFAH2O:ACN)	0.8mL/min
19	3.275	99.36	80:20 (TFAH2O:ACN)	1.5mL/min
20	4.152	99.25	80:20 (TFAH2O:ACN)	1.5mL/min
21	2.615	100.00	70:30 (TFAH2O:ACN)	0.8mL/min
22	3.585	96.26	70:30 (TFAH2O:ACN)	0.8mL/min
23	1.885	100.00	60:40 (TFAH2O:ACN)	0.8mL/min
24	3.357	100.00	70:30 (TFAH2O:ACN)	0.8mL/min
25	1.510	98.41	70:30 (TFAH2O:ACN)	0.8mL/min
26	1.737	99.16	70:30 (TFAH2O:ACN)	0.8mL/min
27	2.345	98.59	70:30 (TFAH2O:ACN)	0.8mL/min
28	3.132	100.00	70:30 (TFAH2O:ACN)	0.8mL/min
29	4.743	97.35	70:30 (TFAH2O:ACN)	0.8mL/min
30	2.830	98.71	70:30 (TFAH2O:ACN)	0.8mL/min
31	2.980	98.85	80:20 (TFAH2O:ACN)	0.8mL/min
32	1.322	99.12	80:20 (TFAH2O:ACN)	1.5mL/min
33*	1.343	96.85	70:30 (TFAH2O:CAN)	0.8mL/min
34	1.730	97.14	70:30 (TFAH2O:ACN)	0.8mL/min
35*	2.458	96.99	70:30 (TFAH2O:ACN)	0.8mL/min
36	1.723	97.27	70:30 (TFAH2O:ACN)	0.8mL/min

*Tested as free base.

HPLC chromatograms of compounds **1-36**.

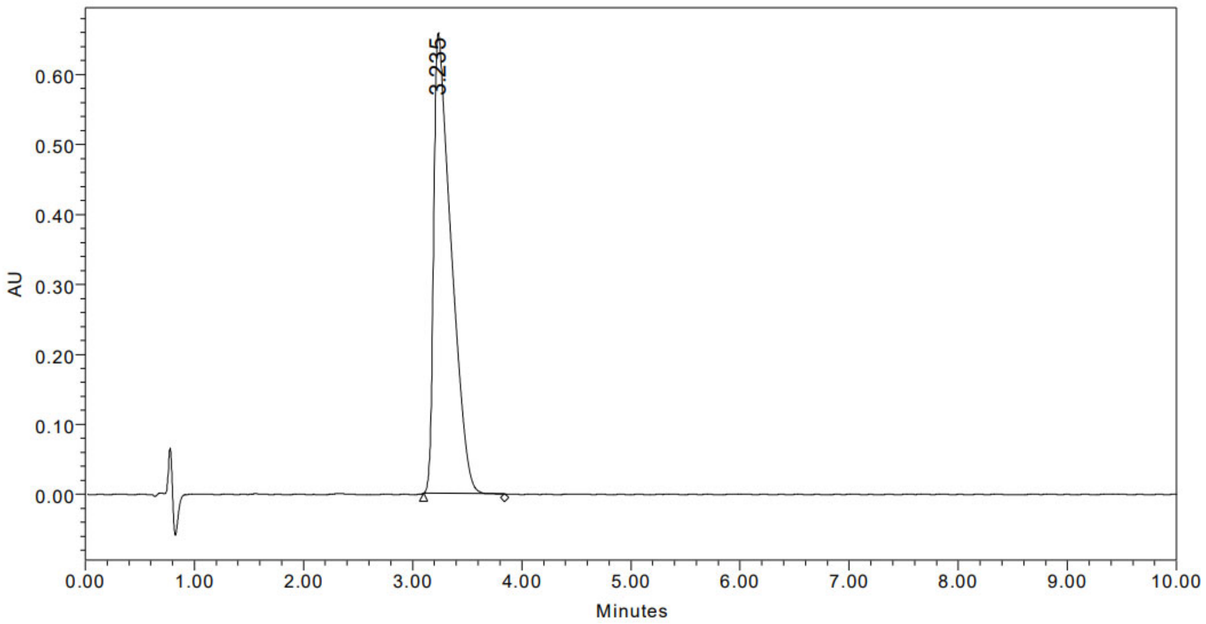

(1)

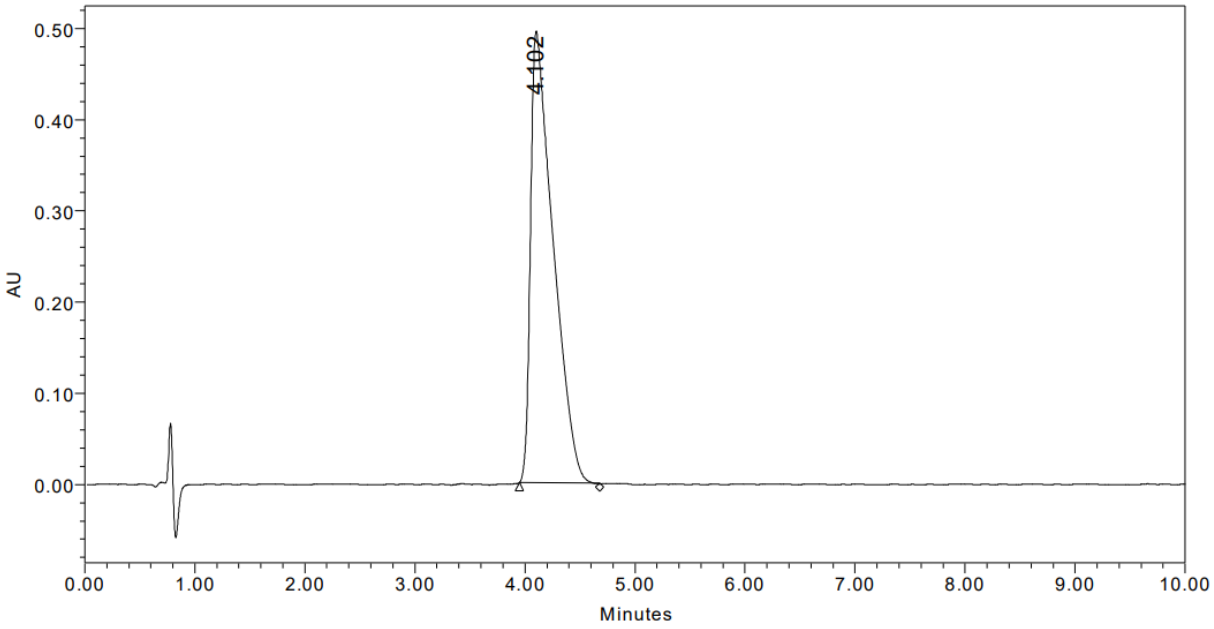

(2)

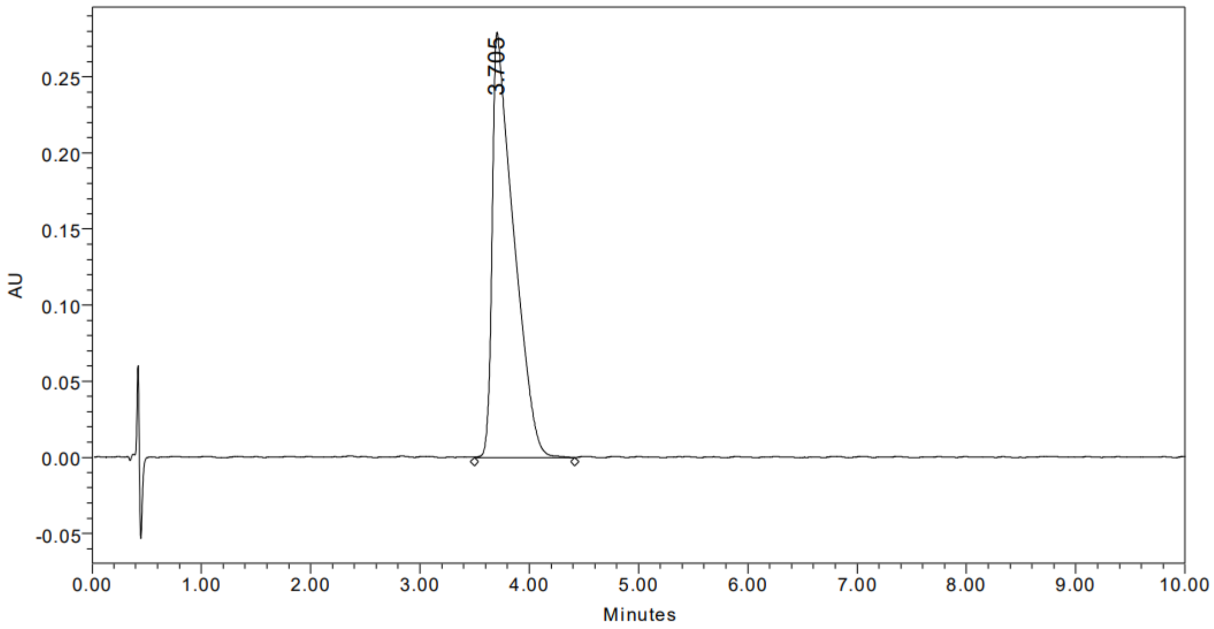

3.705
0.25
0.20
0.15
0.10
0.05
0.00
-0.05
AU
0.00
1.00
2.00
3.00
4.00
5.00
6.00
7.00
8.00
9.00
10.00
Minutes

(3)

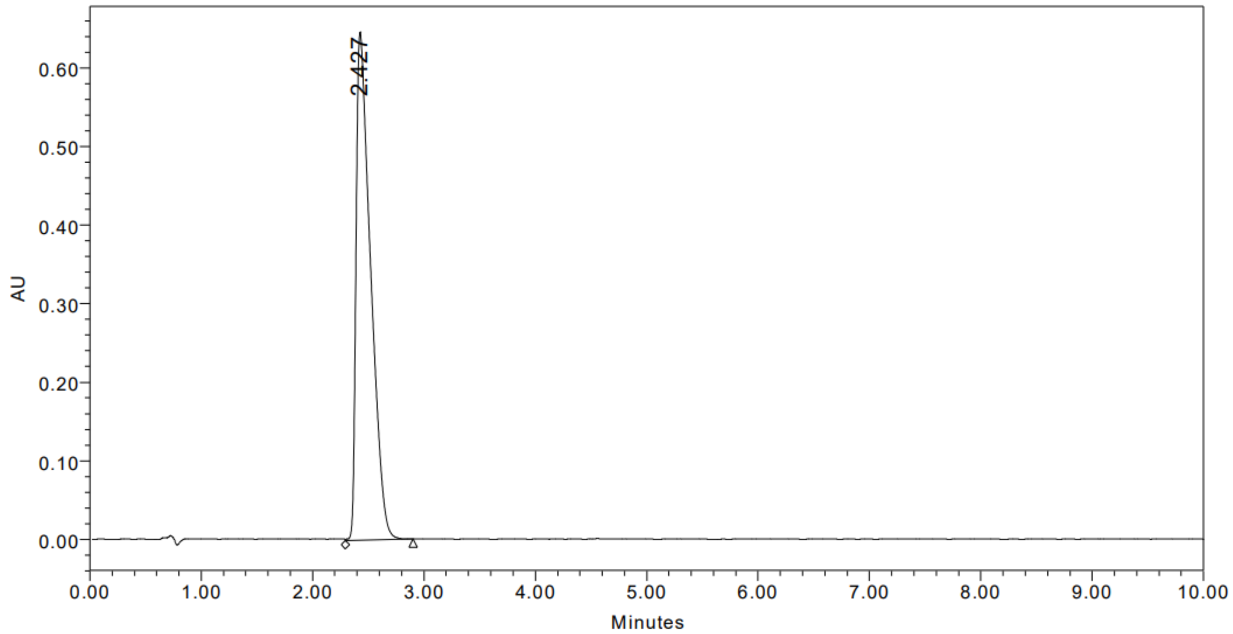

2.427
0.60
0.50
0.40
0.30
0.20
0.10
0.00
AU
0.00
1.00
2.00
3.00
4.00
5.00
6.00
7.00
8.00
9.00
10.00
Minutes

(4)

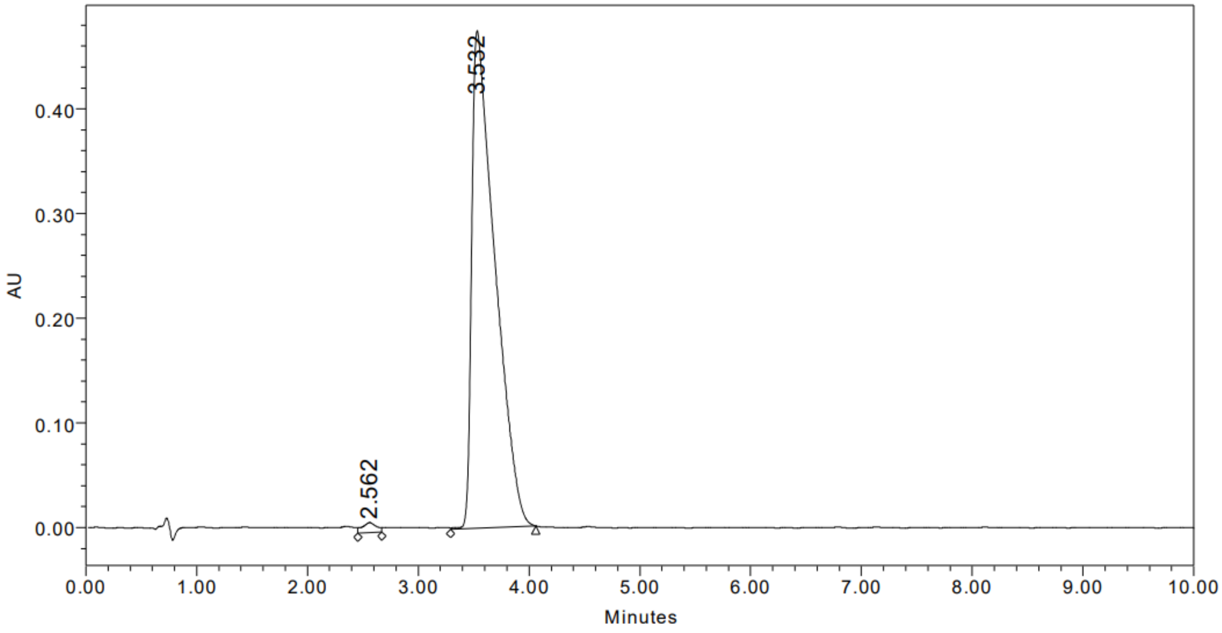

(5)

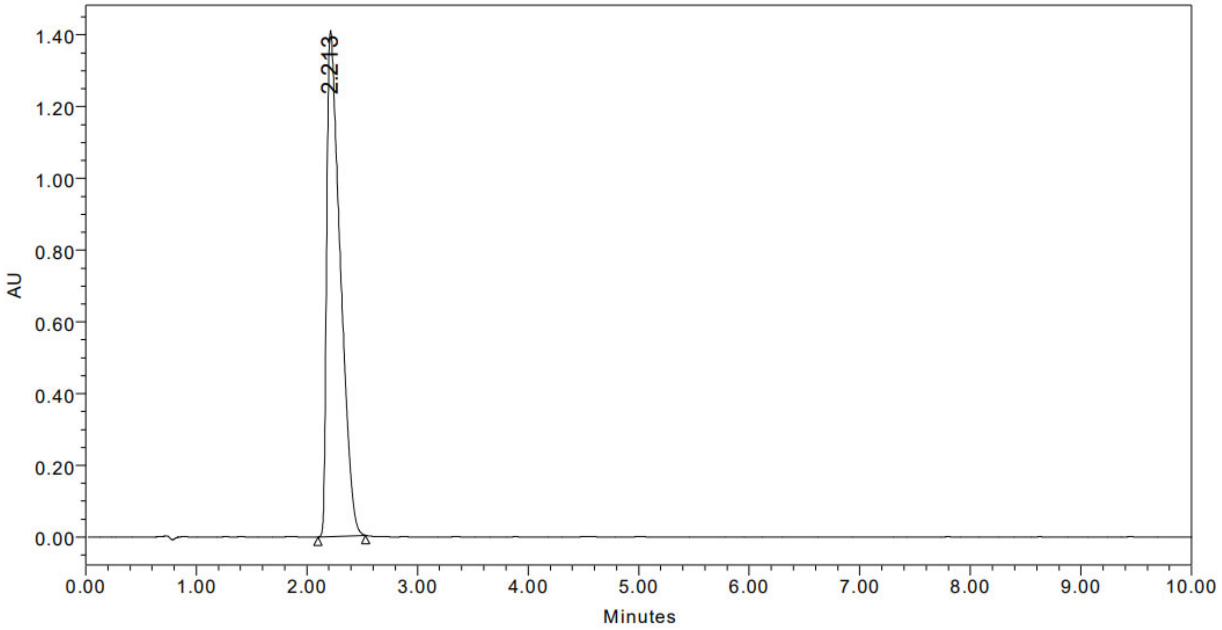

(6)

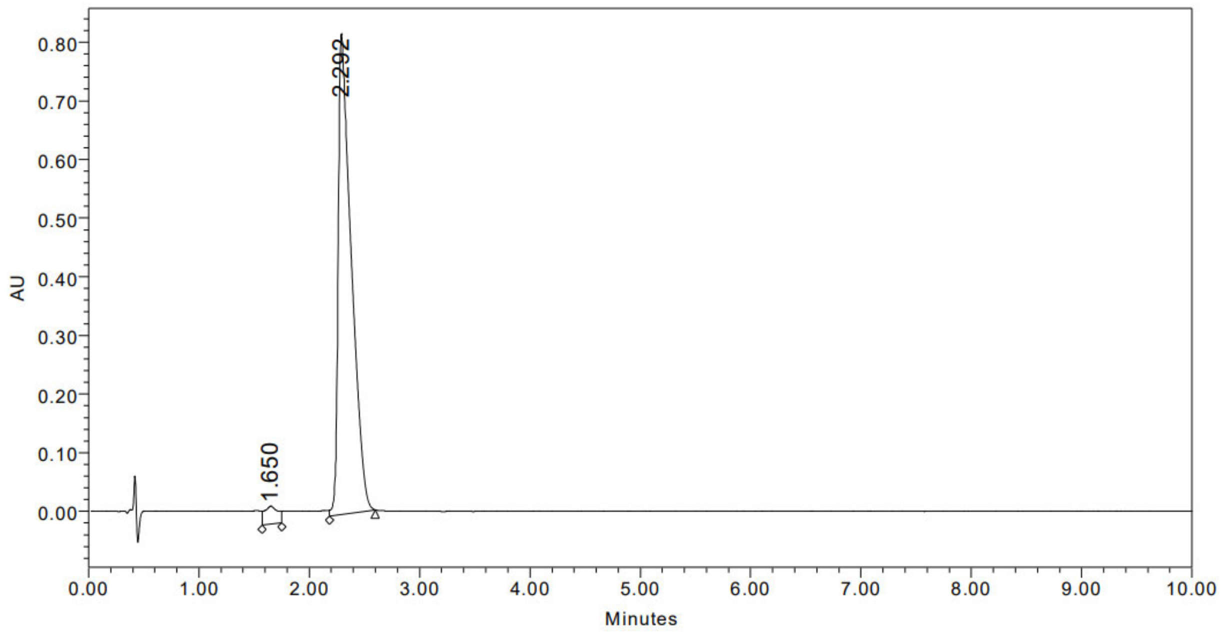

2.292
1.650
0.80
0.70
0.60
0.50
0.40
0.30
0.20
0.10
0.00
AU
0.00 1.00 2.00 3.00 4.00 5.00 6.00 7.00 8.00 9.00 10.00
Minutes

(7)

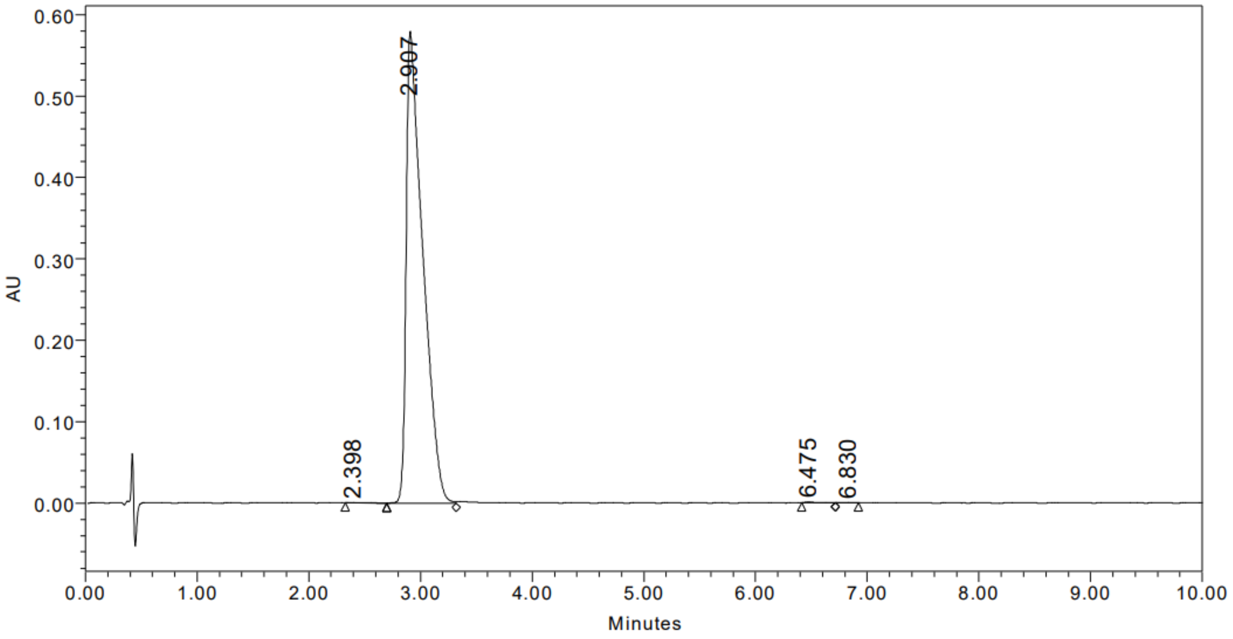

2.907
2.398
6.475
6.830
0.60
0.50
0.40
0.30
0.20
0.10
0.00
AU
0.00 1.00 2.00 3.00 4.00 5.00 6.00 7.00 8.00 9.00 10.00
Minutes

(8)

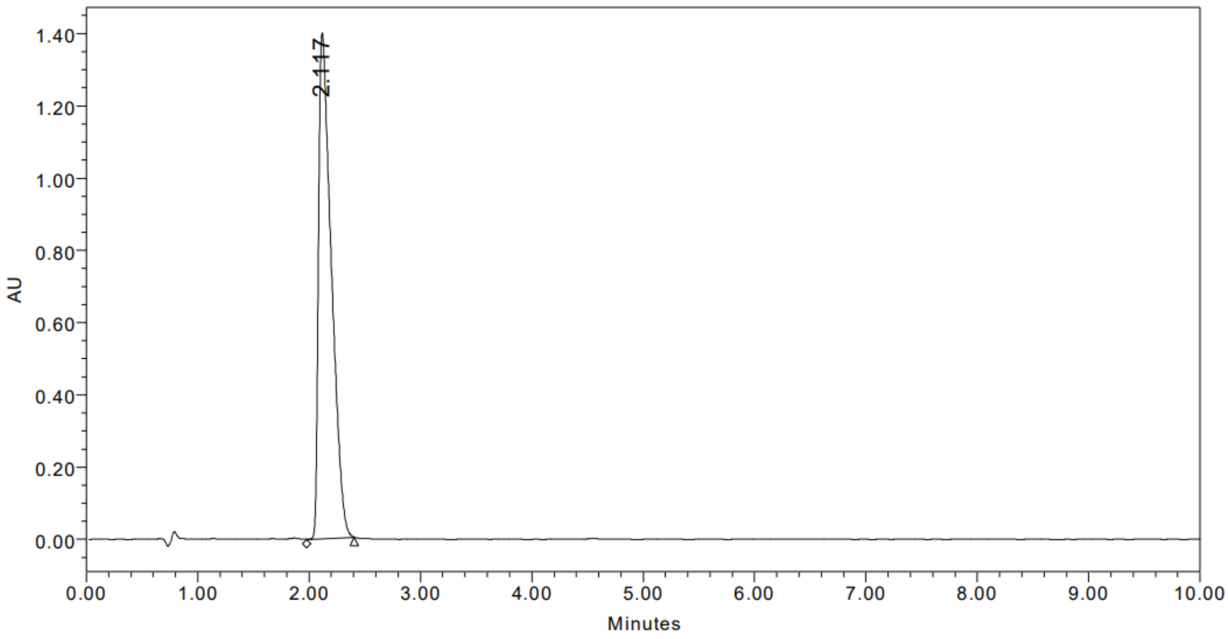

2.117
AU
1.40
1.20
1.00
0.80
0.60
0.40
0.20
0.00
0.00
1.00
2.00
3.00
4.00
5.00
6.00
7.00
8.00
9.00
10.00
Minutes

(9)

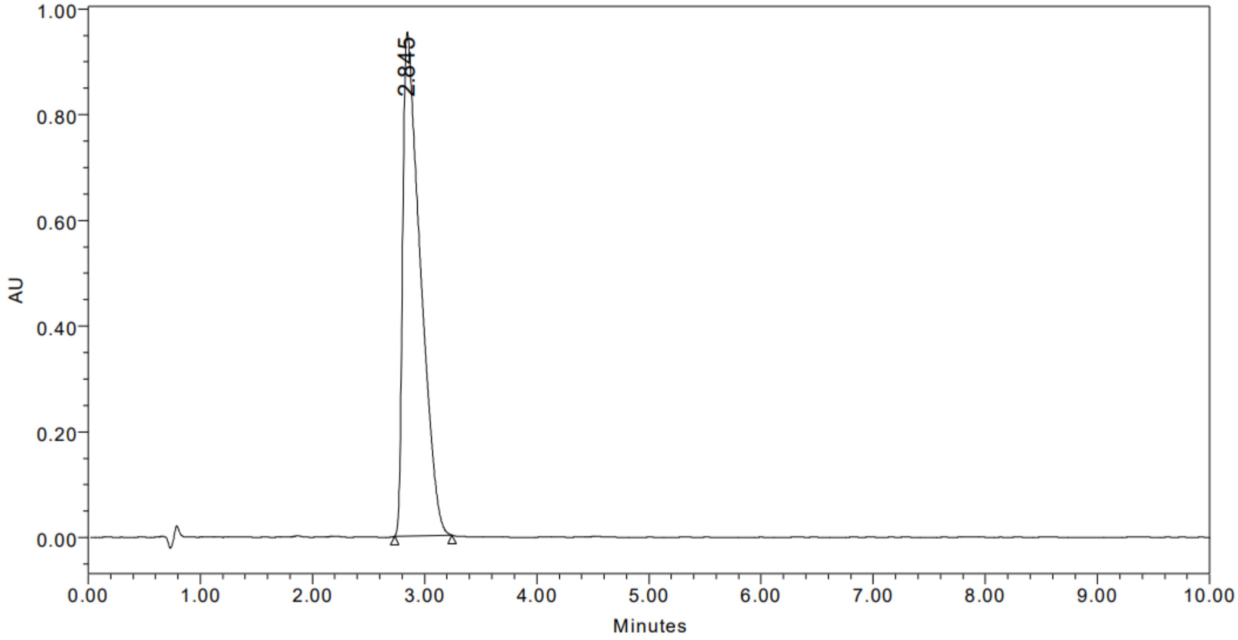

2.845
AU
1.00
0.80
0.60
0.40
0.20
0.00
0.00
1.00
2.00
3.00
4.00
5.00
6.00
7.00
8.00
9.00
10.00
Minutes

(10)

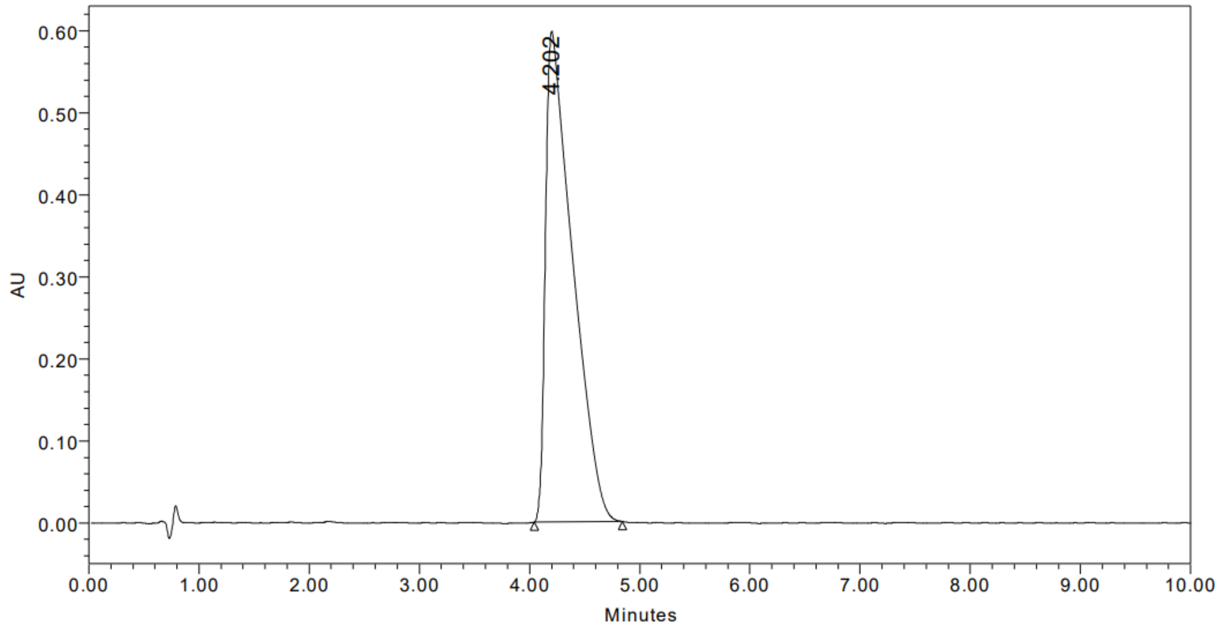
4.202
AU
0.60
0.50
0.40
0.30
0.20
0.10
0.00
0.00
1.00
2.00
3.00
4.00
5.00
6.00
7.00
8.00
9.00
10.00
Minutes

(11)

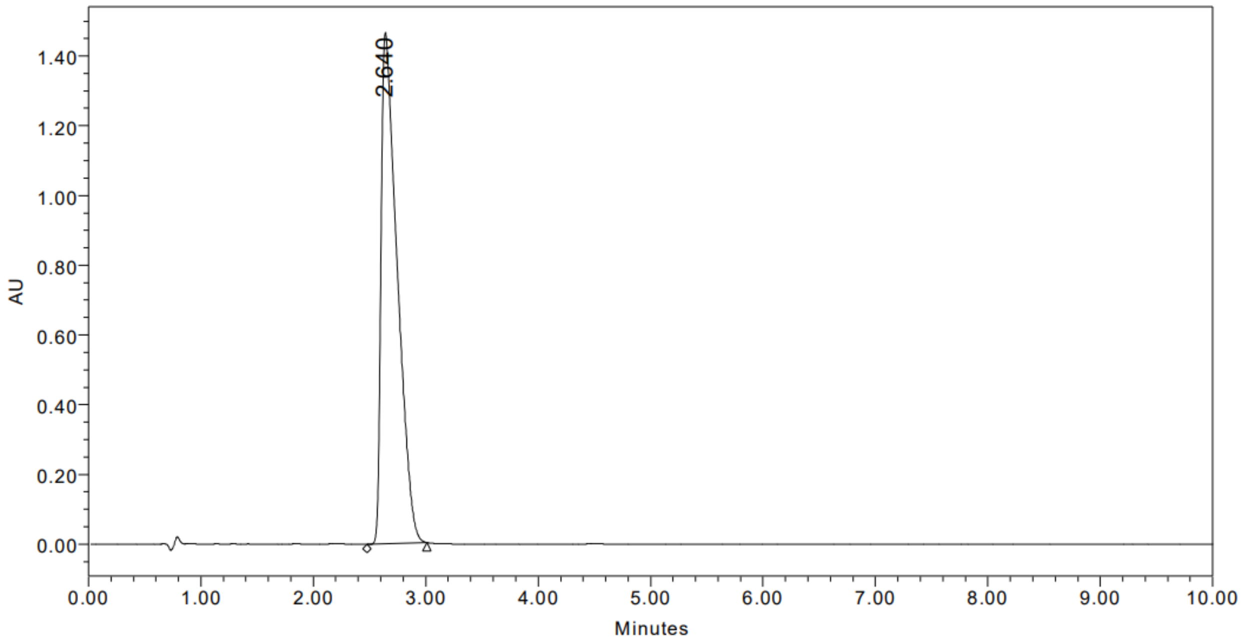
2.640
AU
1.40
1.20
1.00
0.80
0.60
0.40
0.20
0.00
0.00
1.00
2.00
3.00
4.00
5.00
6.00
7.00
8.00
9.00
10.00
Minutes

(12)

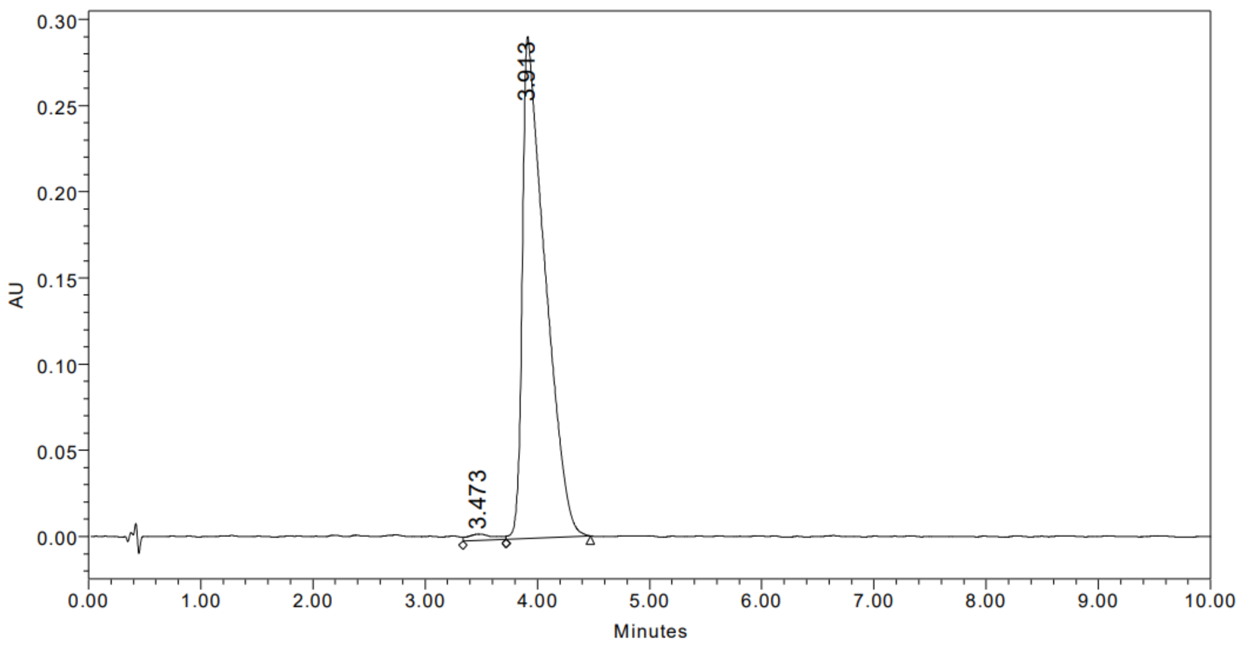

0.30
0.25
0.20
0.15
0.10
0.05
0.00
AU
3.913
3.473
0.00 1.00 2.00 3.00 4.00 5.00 6.00 7.00 8.00 9.00 10.00
Minutes

(13)

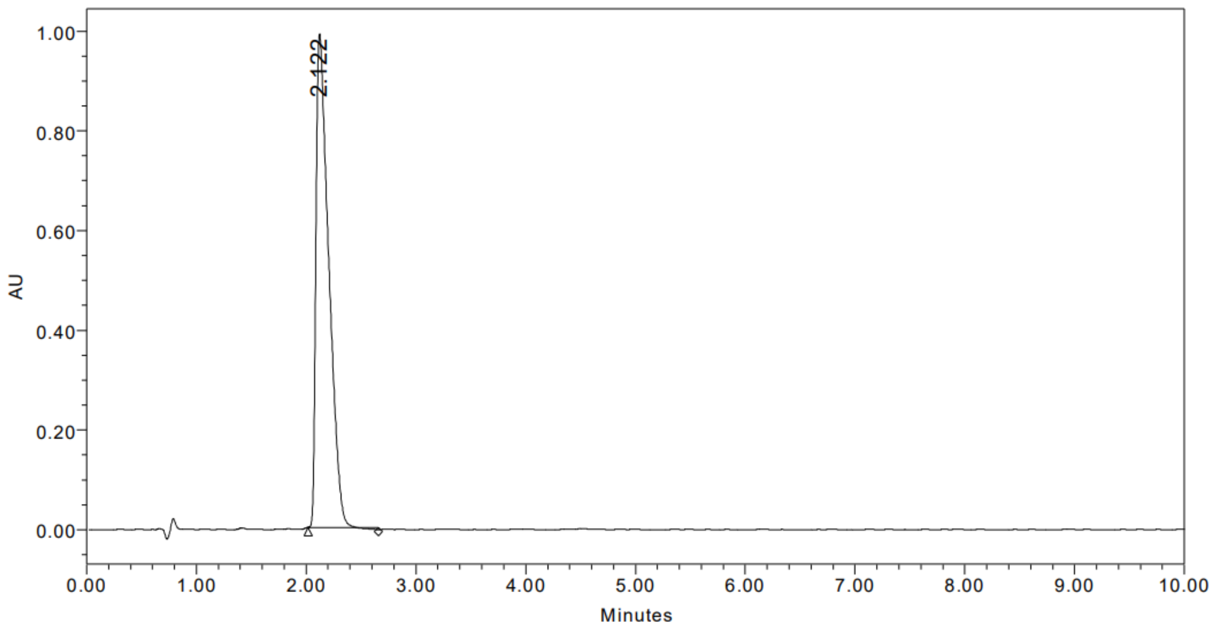

1.00
0.80
0.60
0.40
0.20
0.00
AU
2.122
0.00 1.00 2.00 3.00 4.00 5.00 6.00 7.00 8.00 9.00 10.00
Minutes

(14)

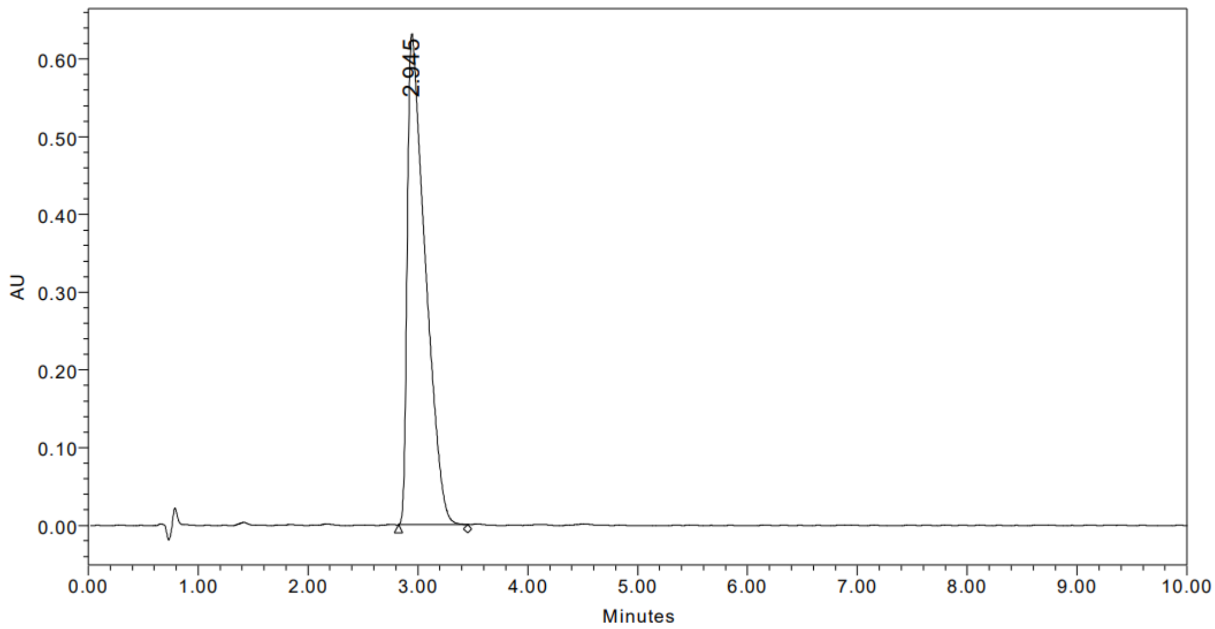
2.945
0.60
0.50
0.40
0.30
0.20
0.10
0.00
AU
0.00
1.00
2.00
3.00
4.00
5.00
6.00
7.00
8.00
9.00
10.00
Minutes

(15)

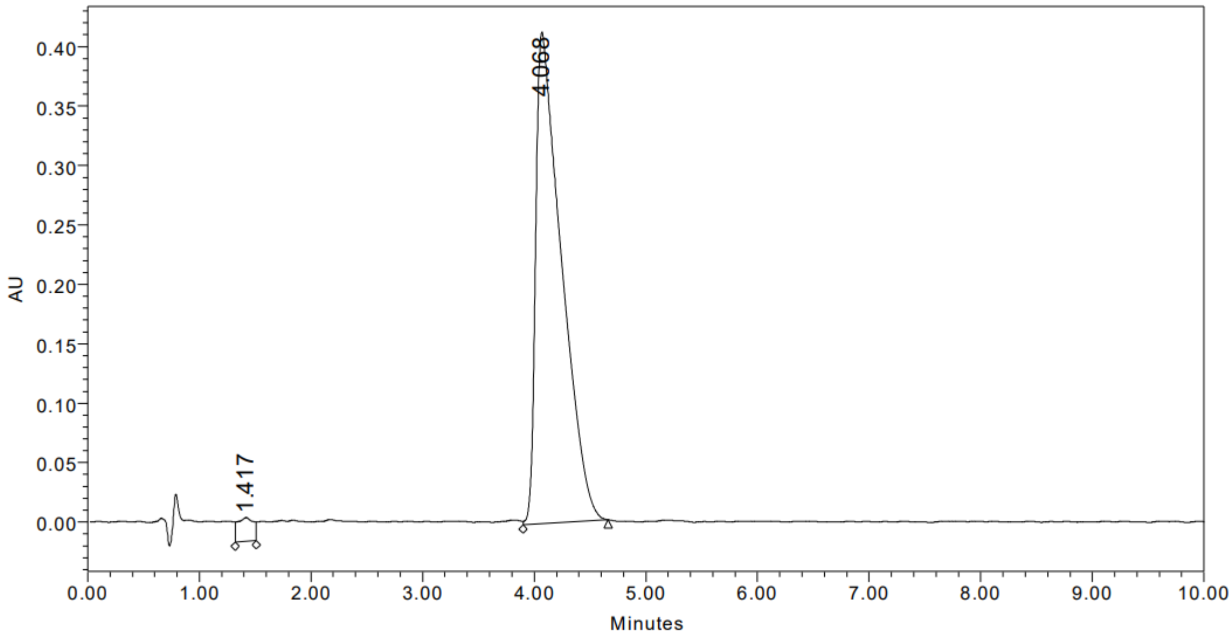
4.068
1.417
0.40
0.35
0.30
0.25
0.20
0.15
0.10
0.05
0.00
AU
0.00
1.00
2.00
3.00
4.00
5.00
6.00
7.00
8.00
9.00
10.00
Minutes

(16)

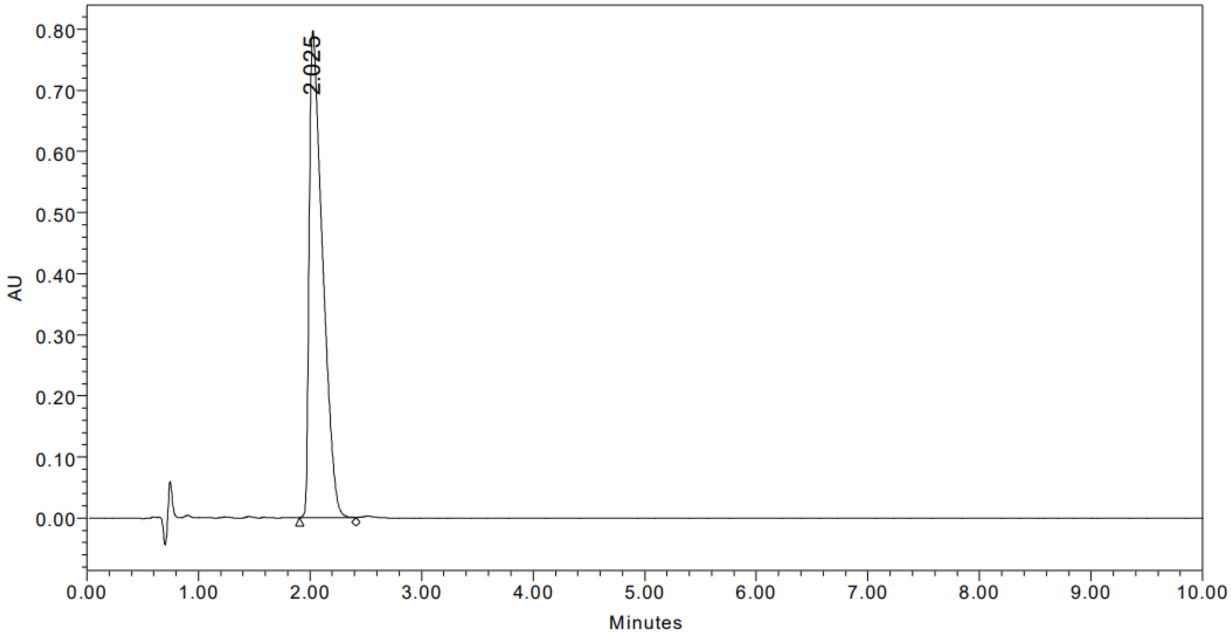

0.80
0.70
0.60
0.50
0.40
0.30
0.20
0.10
0.00
AU
2.025
0.00
1.00
2.00
3.00
4.00
5.00
6.00
7.00
8.00
9.00
10.00
Minutes

(17)

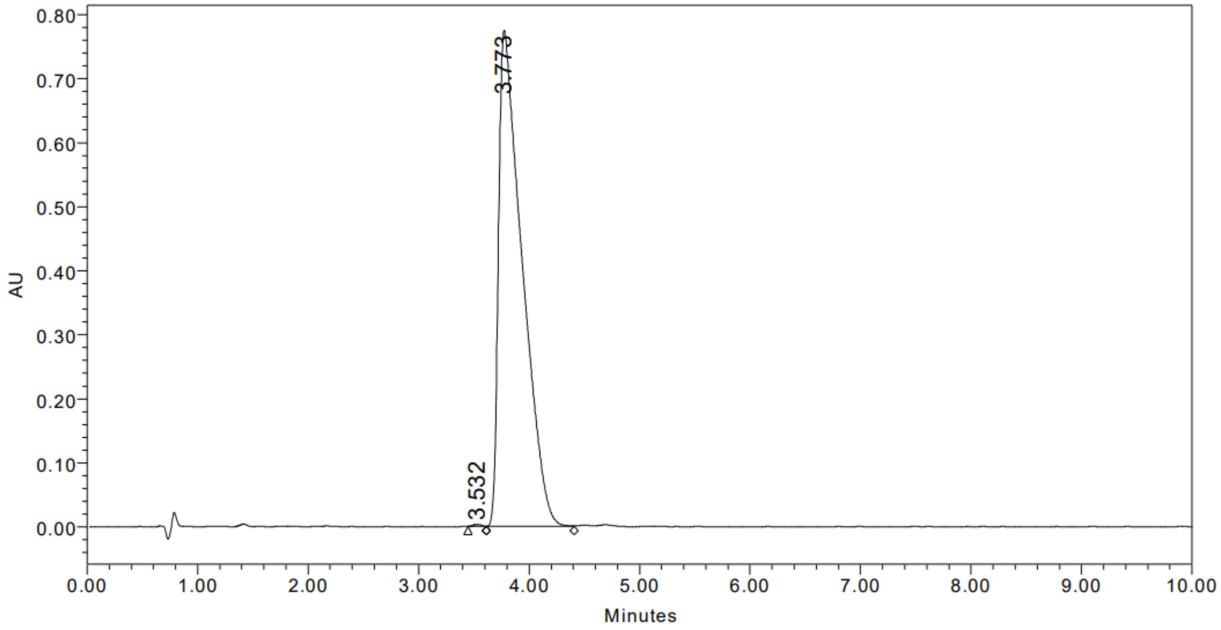

0.80
0.70
0.60
0.50
0.40
0.30
0.20
0.10
0.00
AU
3.773
3.532
0.00
1.00
2.00
3.00
4.00
5.00
6.00
7.00
8.00
9.00
10.00
Minutes

(18)

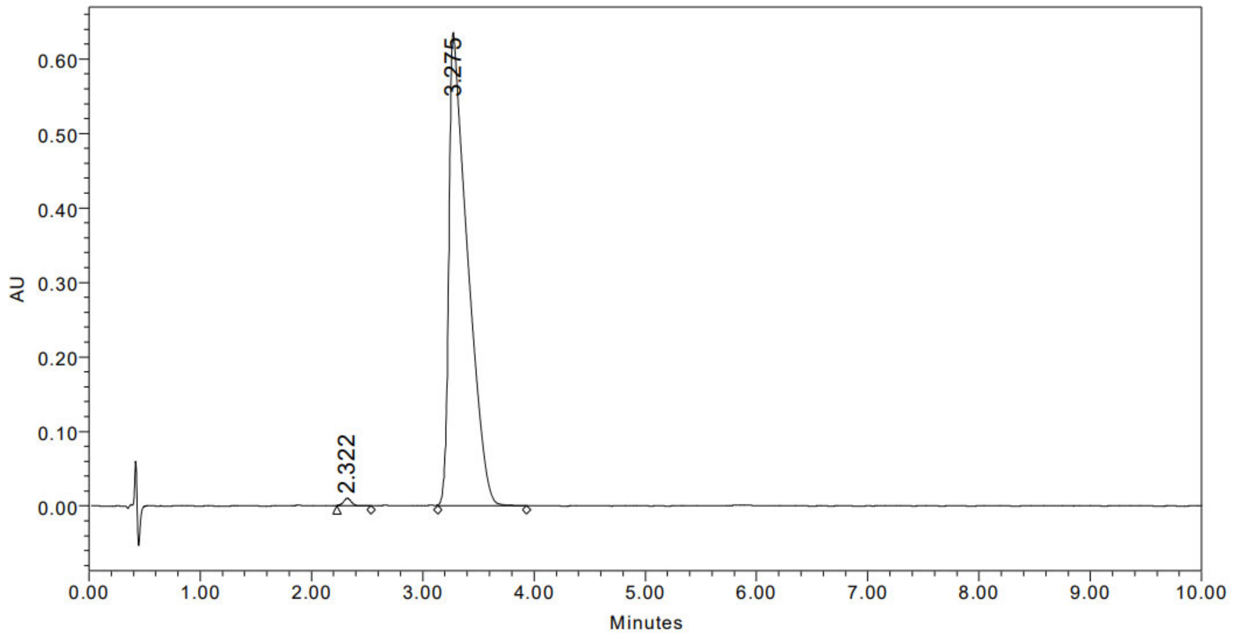

0.60
0.50
0.40
0.30
0.20
0.10
0.00
AU
3.275
2.322
0.00 1.00 2.00 3.00 4.00 5.00 6.00 7.00 8.00 9.00 10.00
Minutes

(19)

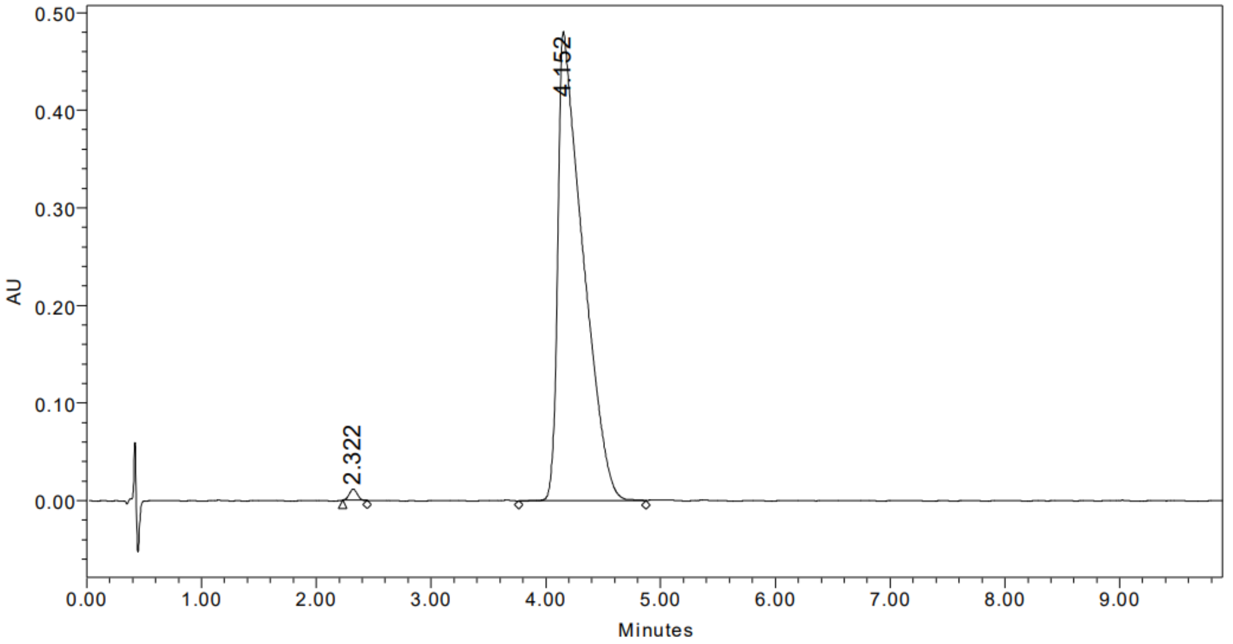

0.50
0.40
0.30
0.20
0.10
0.00
AU
4.152
2.322
0.00 1.00 2.00 3.00 4.00 5.00 6.00 7.00 8.00 9.00
Minutes

(20)

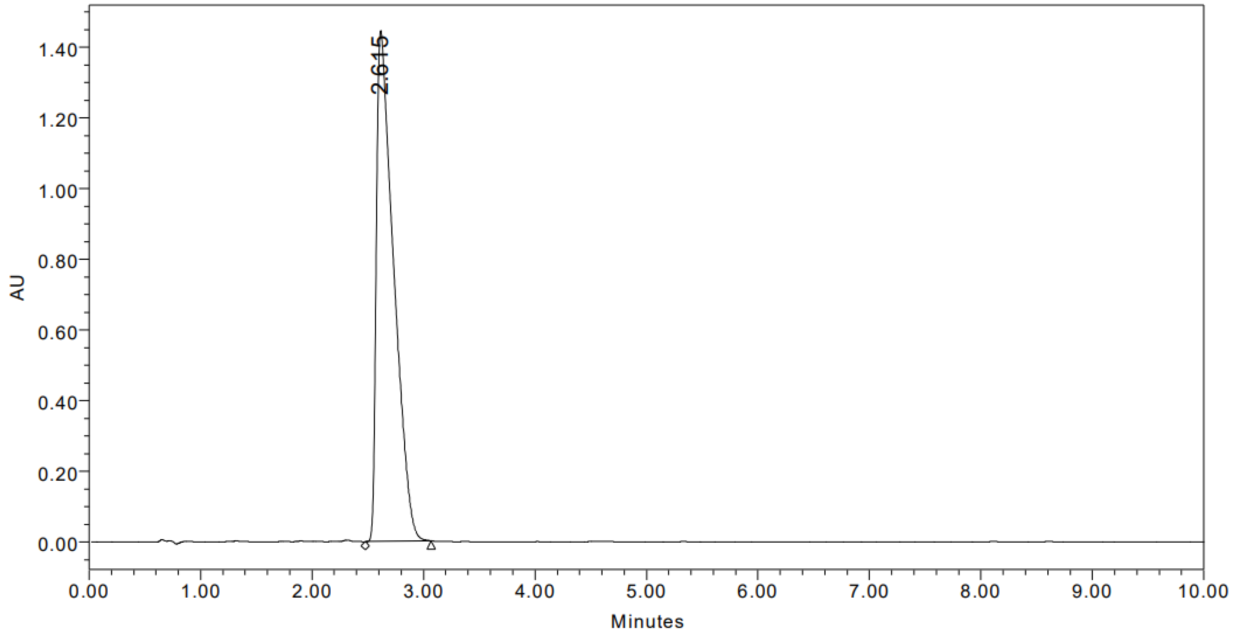
2.615
AU
1.40
1.20
1.00
0.80
0.60
0.40
0.20
0.00
0.00
1.00
2.00
3.00
4.00
5.00
6.00
7.00
8.00
9.00
10.00
Minutes

(21)

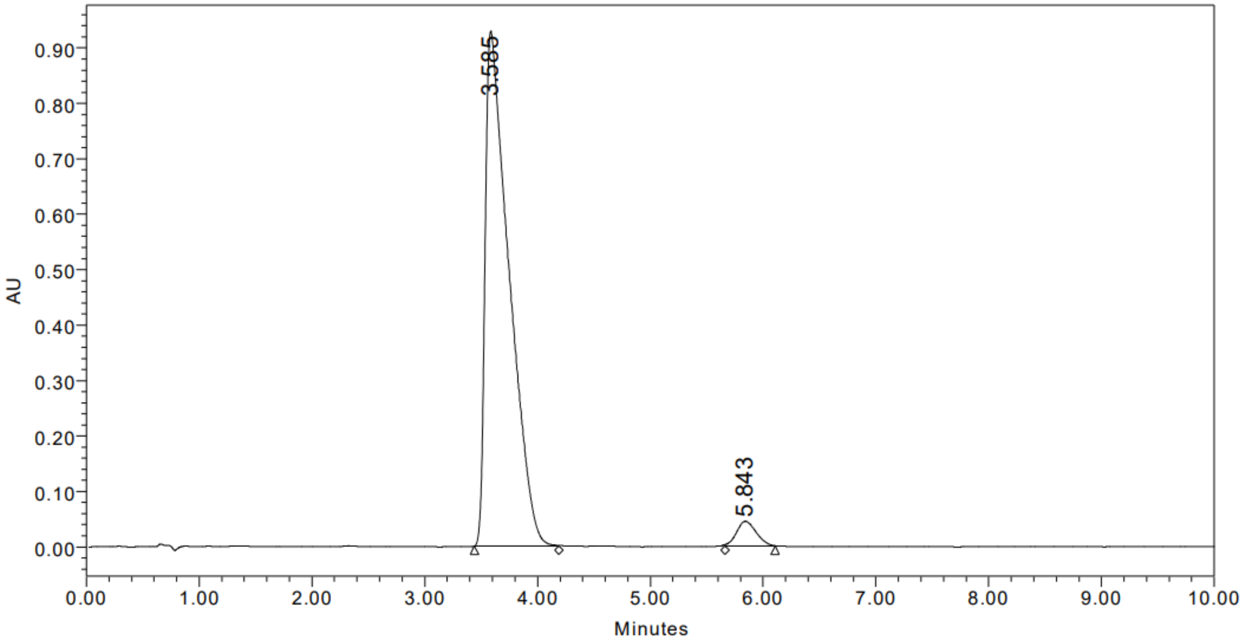
3.585
5.843
AU
0.90
0.80
0.70
0.60
0.50
0.40
0.30
0.20
0.10
0.00
0.00
1.00
2.00
3.00
4.00
5.00
6.00
7.00
8.00
9.00
10.00
Minutes

(22)

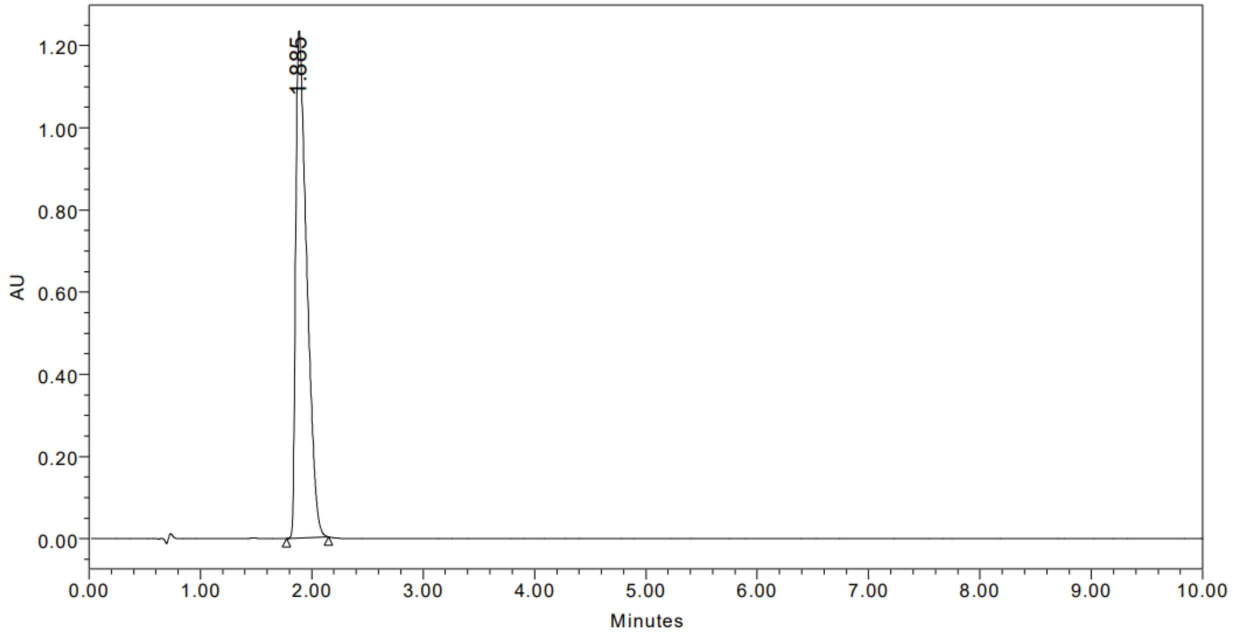
1.885
1.20
1.00
0.80
0.60
0.40
0.20
0.00
AU
0.00
1.00
2.00
3.00
4.00
5.00
6.00
7.00
8.00
9.00
10.00
Minutes

(23)

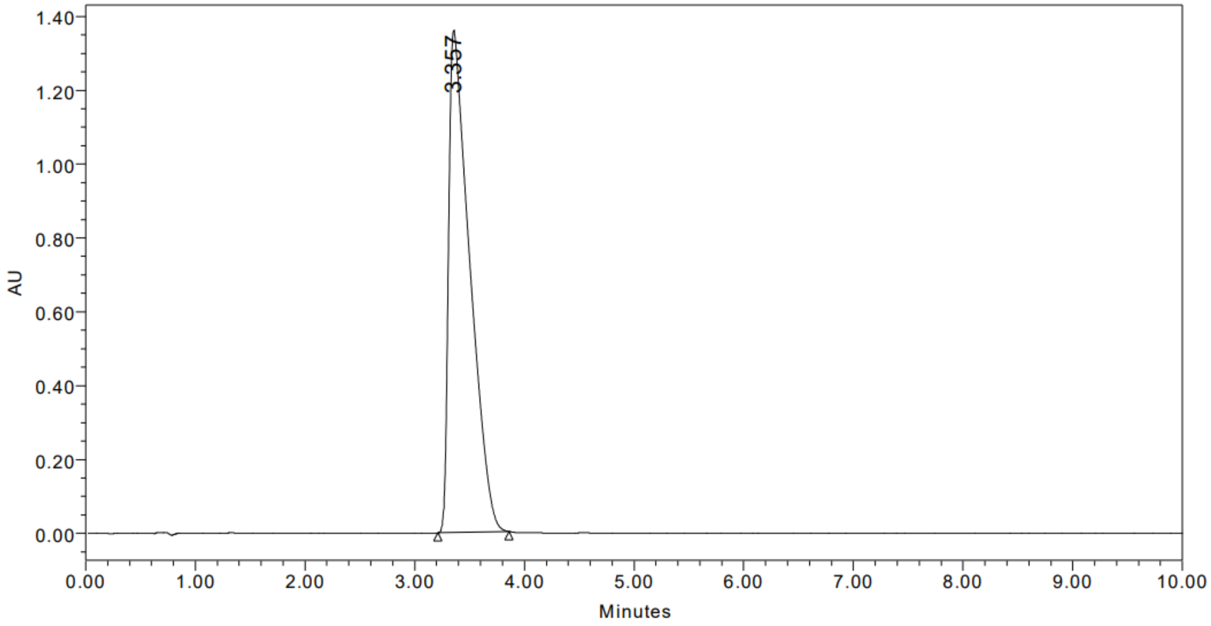
3.357
1.40
1.20
1.00
0.80
0.60
0.40
0.20
0.00
AU
0.00
1.00
2.00
3.00
4.00
5.00
6.00
7.00
8.00
9.00
10.00
Minutes

(24)

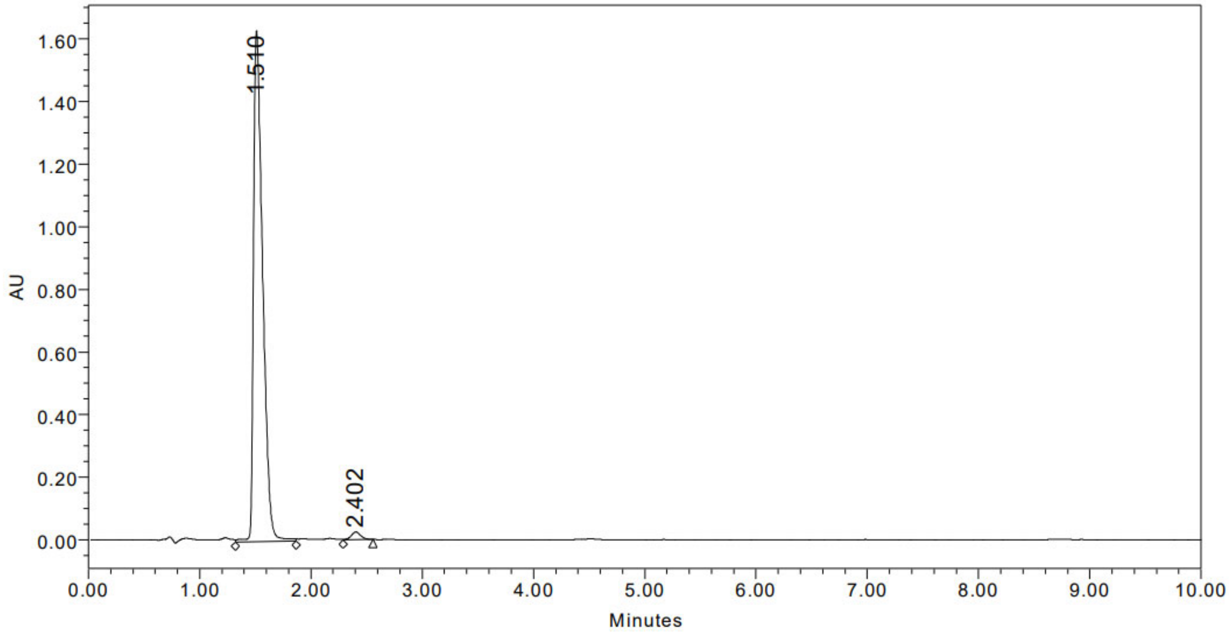
1.510
2.402
AU
Minutes
0.00
0.20
0.40
0.60
0.80
1.00
1.20
1.40
1.60
1.00
2.00
3.00
4.00
5.00
6.00
7.00
8.00
9.00
10.00

(25)

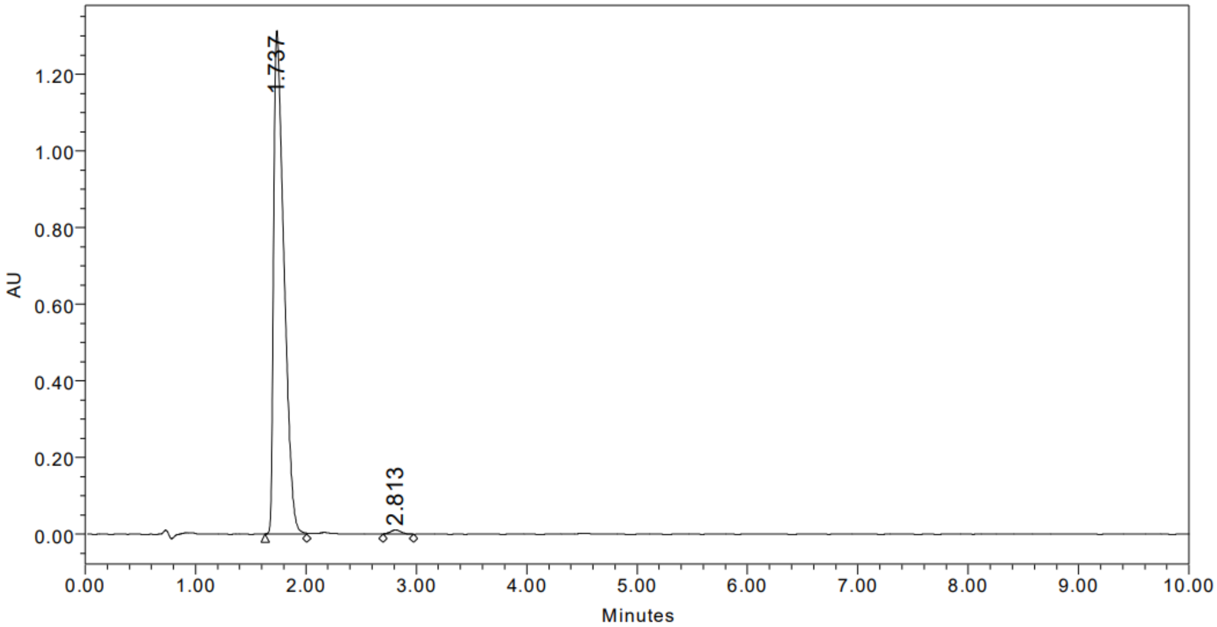
1.737
2.813
AU
Minutes
0.00
0.20
0.40
0.60
0.80
1.00
1.20
1.00
2.00
3.00
4.00
5.00
6.00
7.00
8.00
9.00
10.00

(26)

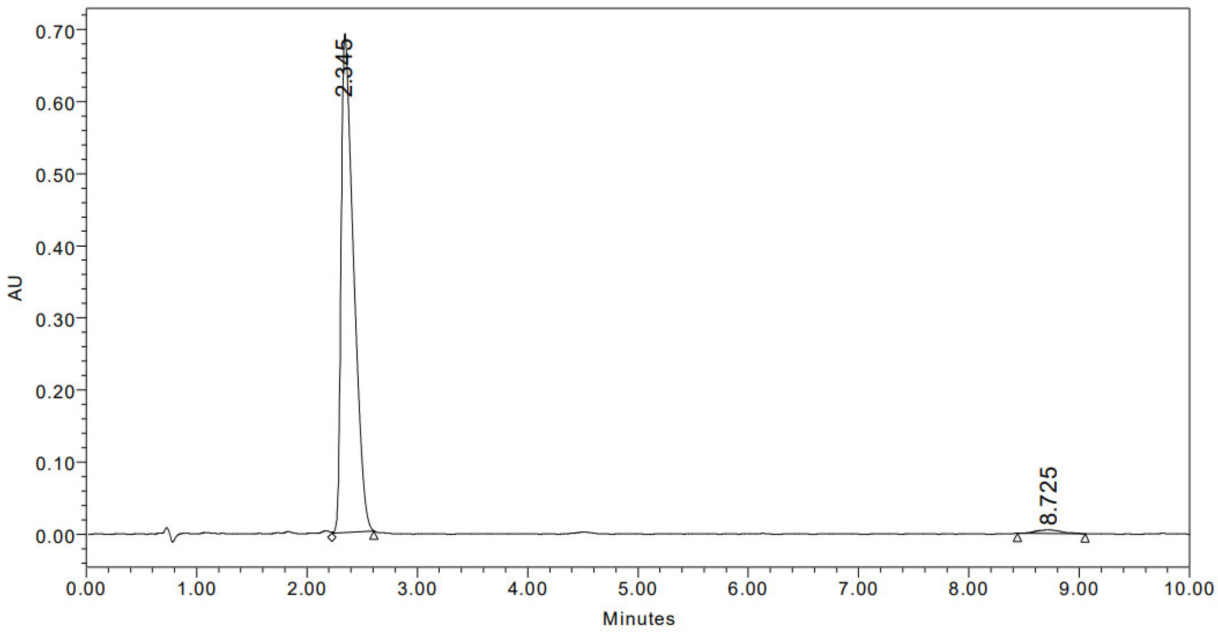
2.345
8.725
AU
0.70
0.60
0.50
0.40
0.30
0.20
0.10
0.00
0.00
1.00
2.00
3.00
4.00
5.00
6.00
7.00
8.00
9.00
10.00
Minutes

(27)

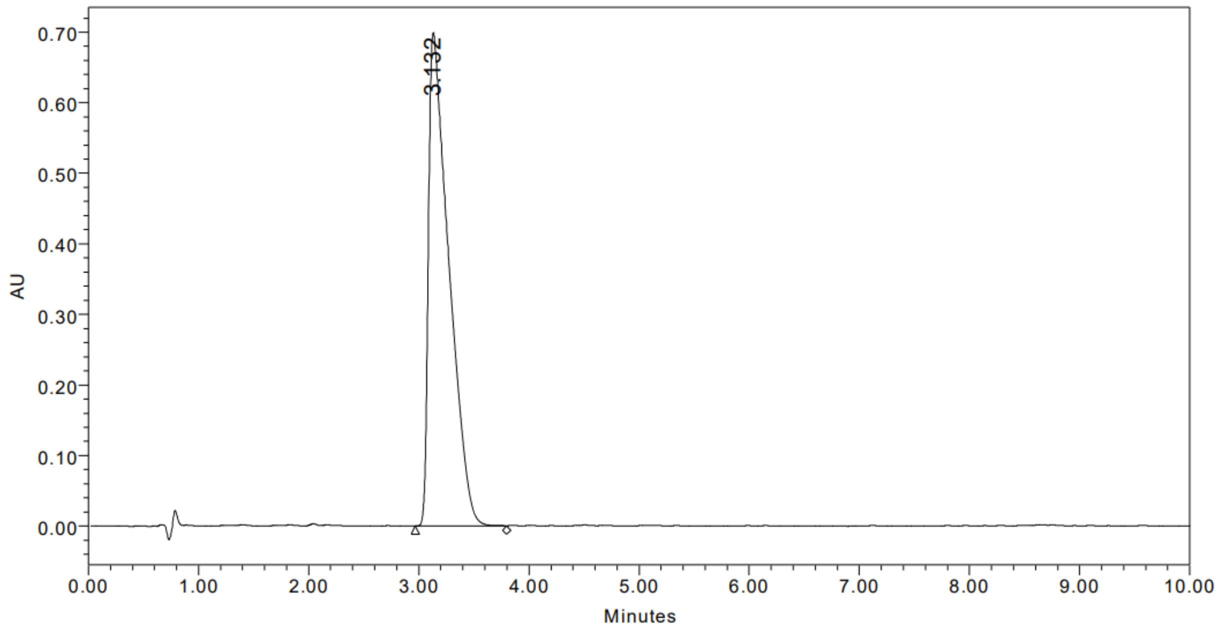
3.132
AU
0.70
0.60
0.50
0.40
0.30
0.20
0.10
0.00
0.00
1.00
2.00
3.00
4.00
5.00
6.00
7.00
8.00
9.00
10.00
Minutes

(28)

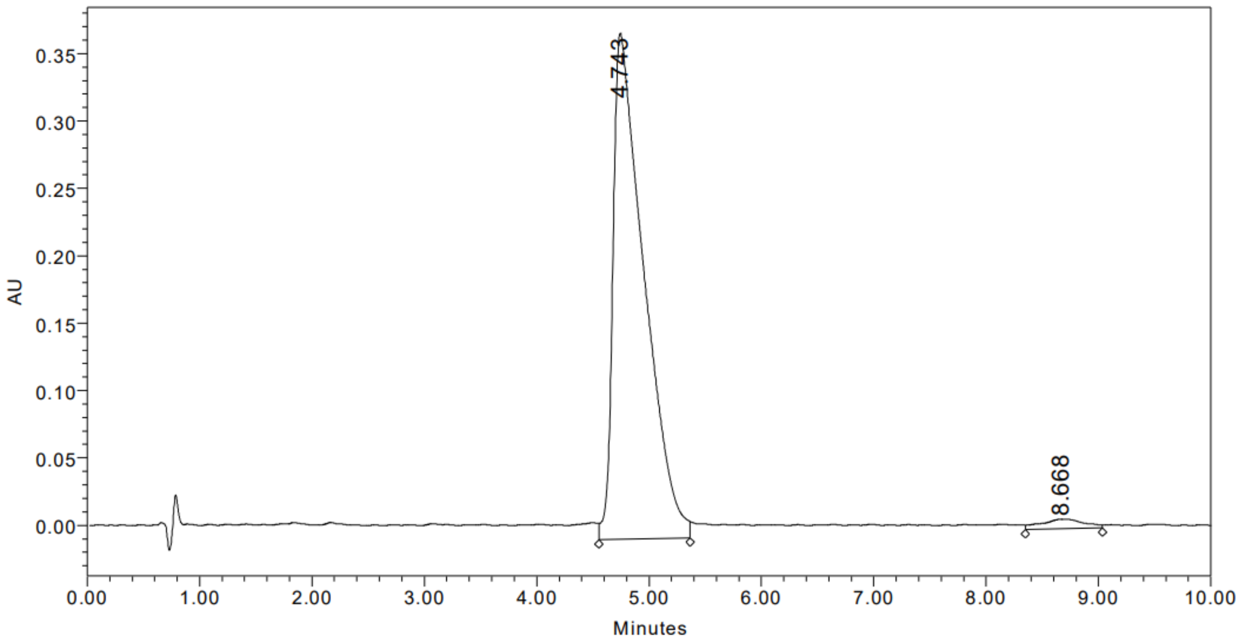

AU
0.35
0.30
0.25
0.20
0.15
0.10
0.05
0.00
4.743
8.668
0.00 1.00 2.00 3.00 4.00 5.00 6.00 7.00 8.00 9.00 10.00
Minutes

(29)

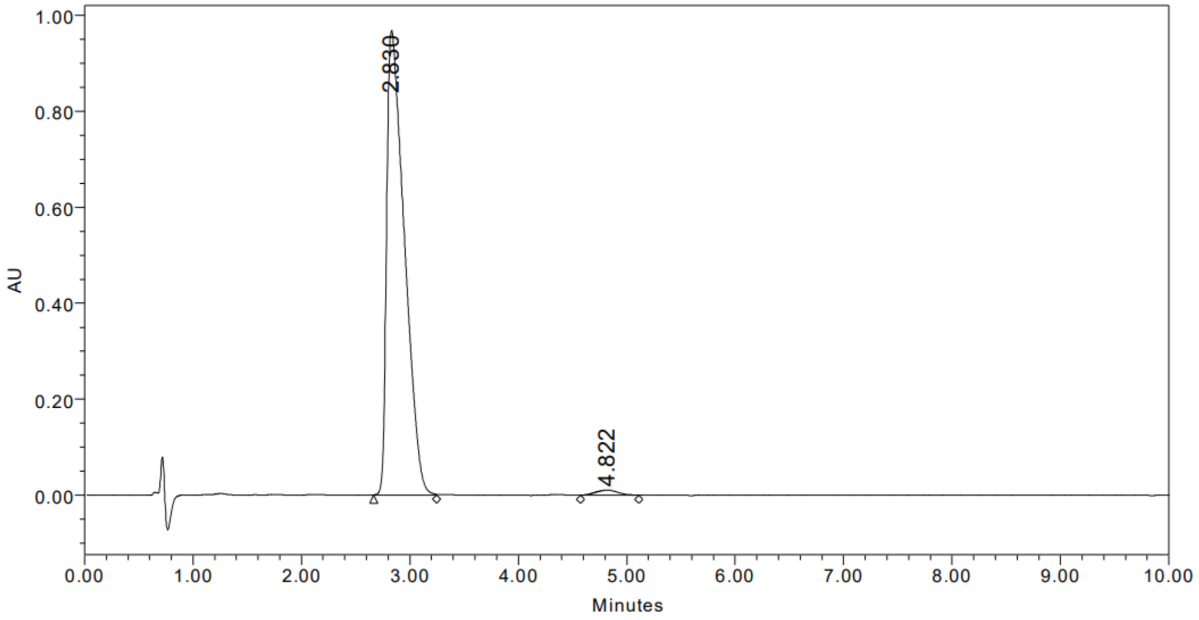

AU
1.00
0.80
0.60
0.40
0.20
0.00
2.830
4.822
0.00 1.00 2.00 3.00 4.00 5.00 6.00 7.00 8.00 9.00 10.00
Minutes

(30)

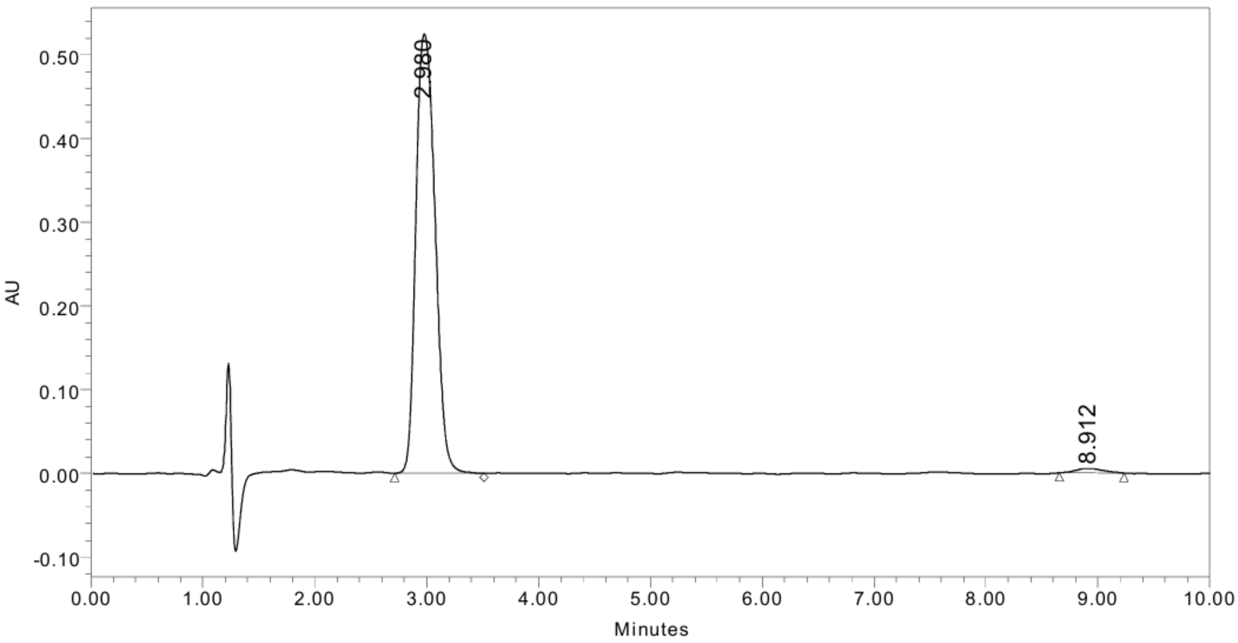

2.980
8.912
AU
0.50
0.40
0.30
0.20
0.10
0.00
-0.10
0.00 1.00 2.00 3.00 4.00 5.00 6.00 7.00 8.00 9.00 10.00
Minutes

(31)

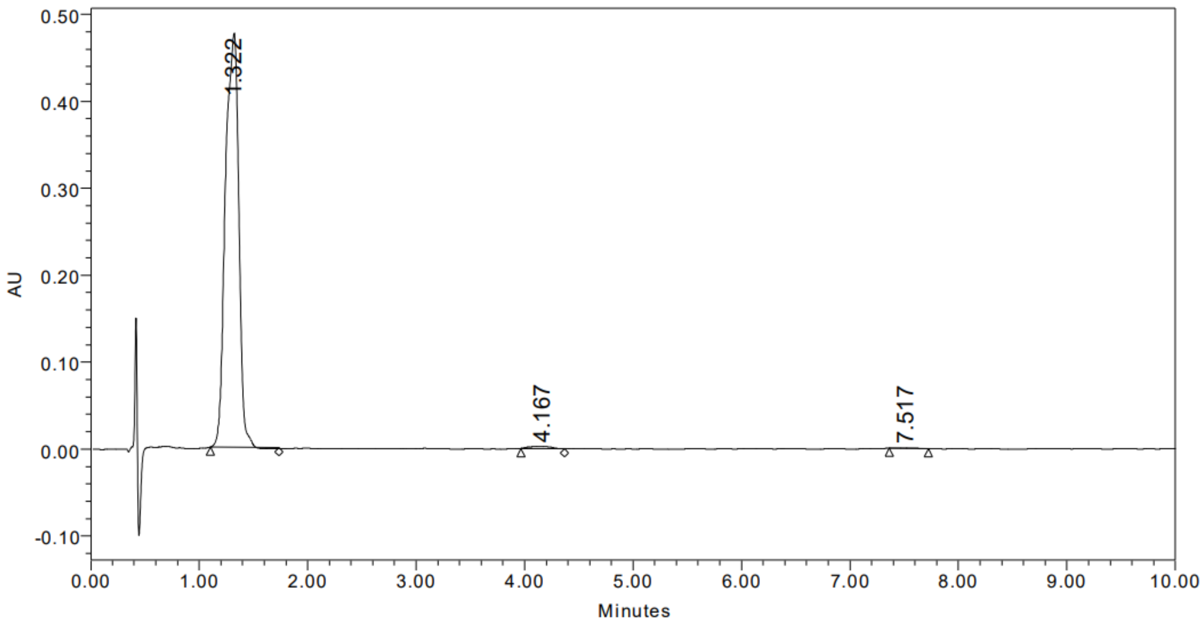

1.322
4.167
7.517
AU
0.50
0.40
0.30
0.20
0.10
0.00
-0.10
0.00 1.00 2.00 3.00 4.00 5.00 6.00 7.00 8.00 9.00 10.00
Minutes

(32)

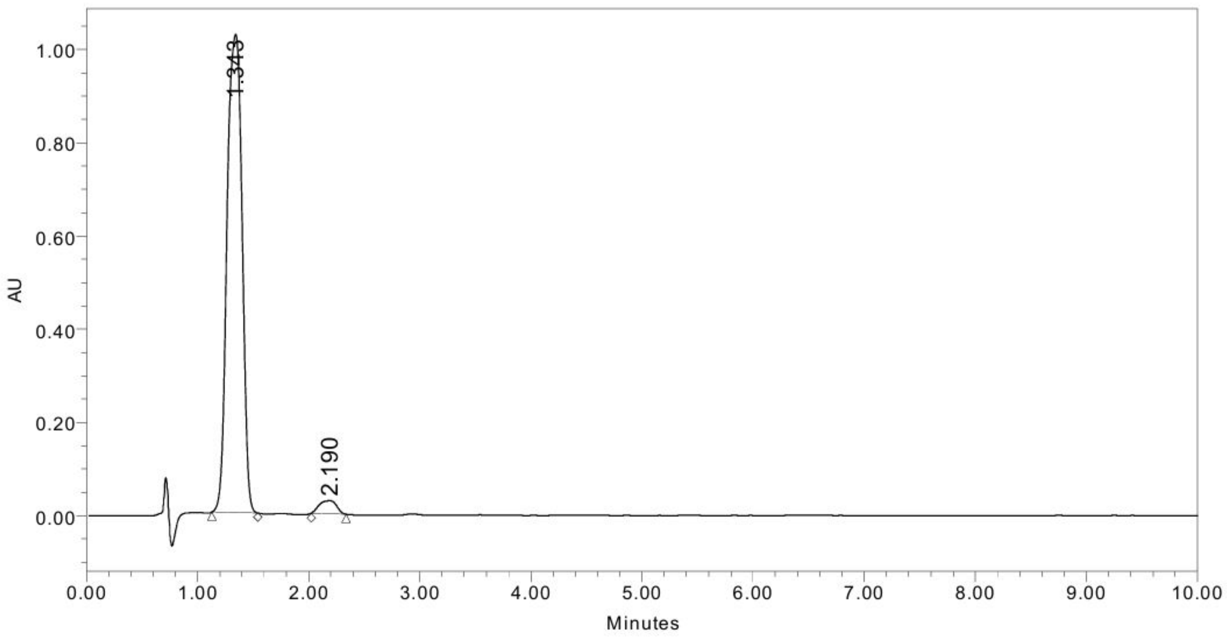
1.349
2.190
1.00
0.80
0.60
0.40
0.20
0.00
AU
0.00
1.00
2.00
3.00
4.00
5.00
6.00
7.00
8.00
9.00
10.00
Minutes

(33)

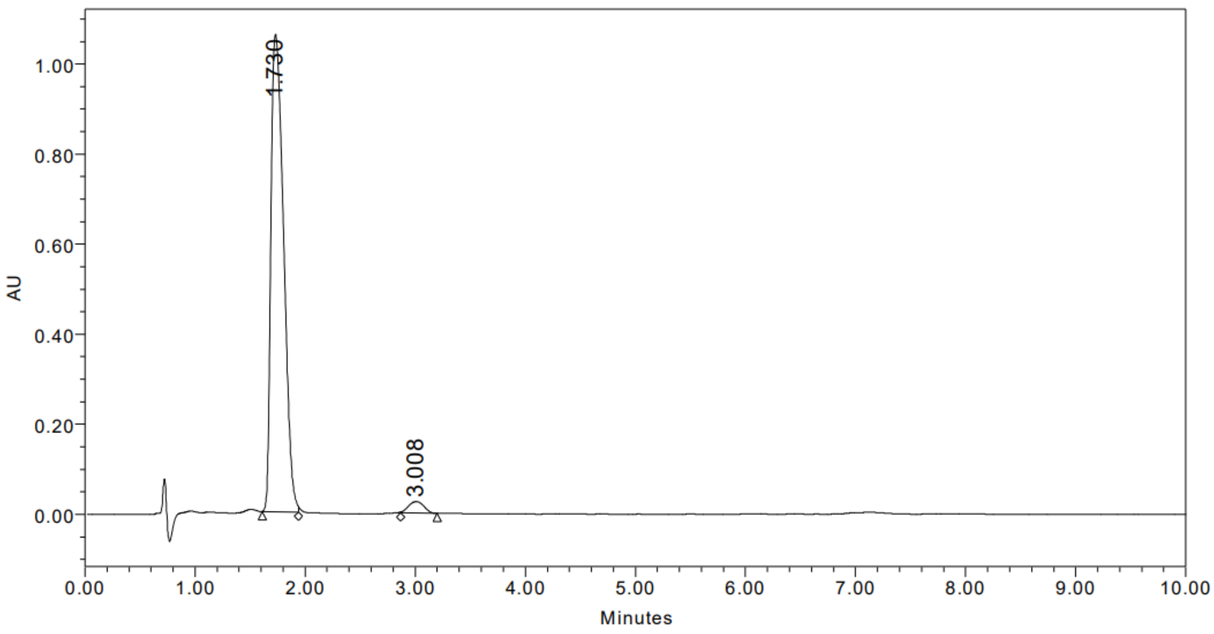
1.730
3.008
1.00
0.80
0.60
0.40
0.20
0.00
AU
0.00
1.00
2.00
3.00
4.00
5.00
6.00
7.00
8.00
9.00
10.00
Minutes

(34)

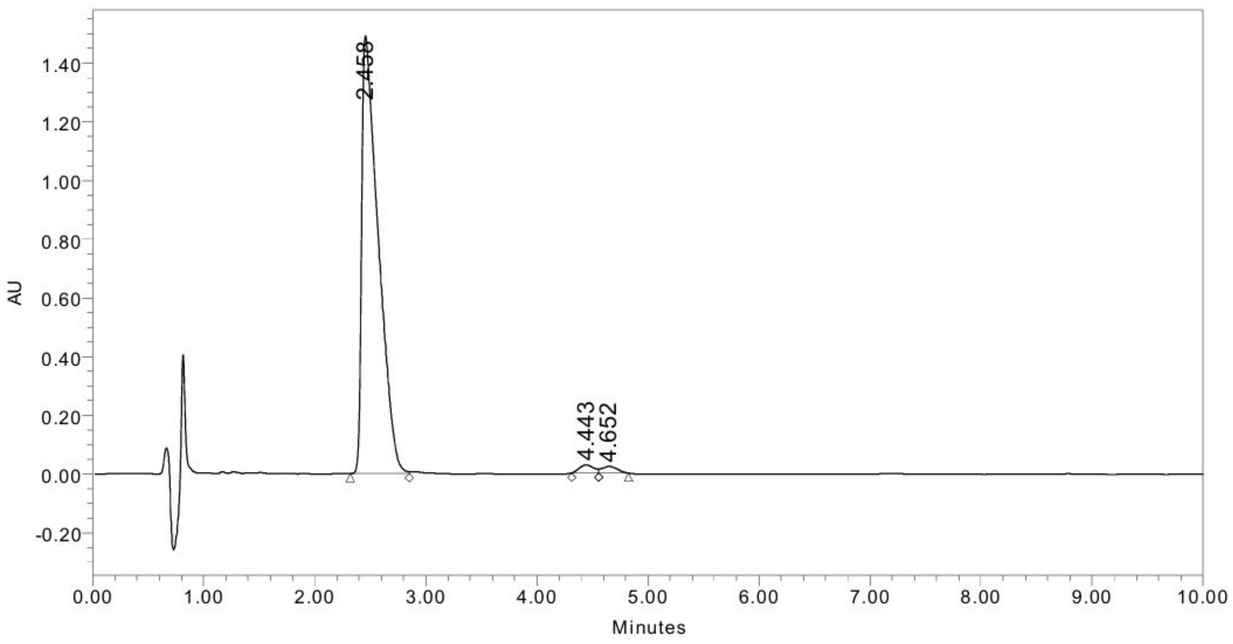

(35)

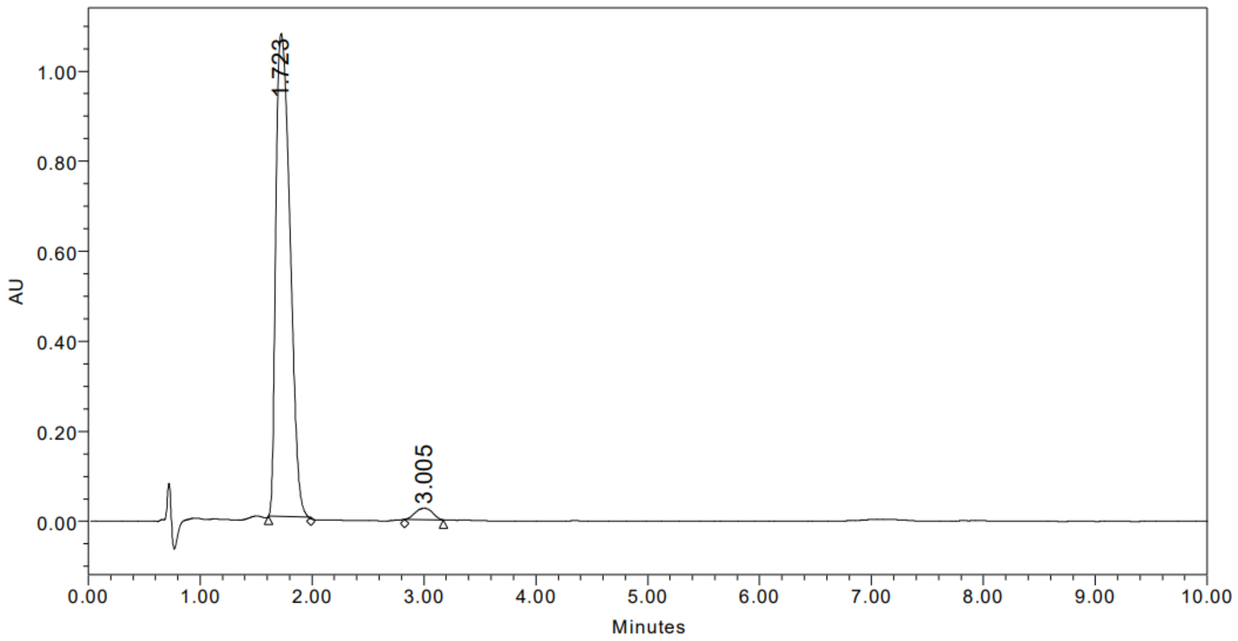

(36)

4.2 HPLC data and spectrum of compounds **37-78**.

HPLC System: Waters Arc HPLC
Column: XBridge™ C_{18} 3.5 µm (4.6 x 50 mm)
Sample Concentration: 0.25 mg/mL
Injection Solvent: Acetonitrile
Injection Volume: 5 µL
Isocratic Mobile Phase:
30% Mobile Phase A – 0.1% Trifluoroacetic acid in water
70% Mobile Phase B – Acetonitrile
Flow Rate: 0.2 mL/min
Single Wavelength: 210 nm
Run time: 10 min

Table S2. HPLC data for compounds **37-78**.

Compound	Retention Time (min)	Purity (%)
37	2.680	100.00
38	2.723	99.71
39	2.805	99.16
40	2.872	100.00
41	2.987	99.62
42	2.803	99.29
43	2.893	99.76
44	2.722	97.41
45	2.775	99.62
46	2.852	100.00
47	2.935	100.00
48	3.047	100.00
49	2.867	99.82
50	2.938	100.00
51	2.832	99.55
52	2.890	100.00
53	2.978	99.21
54	3.105	100.00
55	3.215	100.00
56	2.997	100.00
57	3.082	100.00
58	2.780	99.78
59	2.833	99.55
60	2.928	99.91
61	3.020	100.00
62	3.147	100.00
63	2.948	99.11
64	3.030	99.09
65[a]	2.715	98.42

66[a]	2.763	97.22
67[a]	2.853	98.22
68[a]	2.935	99.10
69[a]	3.057	97.04
70[a]	2.865	97.61
71[a]	2.935	98.34
72[a]	2.575	95.14
73[a]	2.593	99.50
74[a]	2.647	99.40
75[a]	2.700	97.85
76[a]	2.778	99.16
77[a]	2.648	98.00
78[a]	2.702	97.21
[a] 1 drop of H_2O added to injection solvent to ensure homogenous solution.		

HPLC chromatograms of compounds **37-78**.

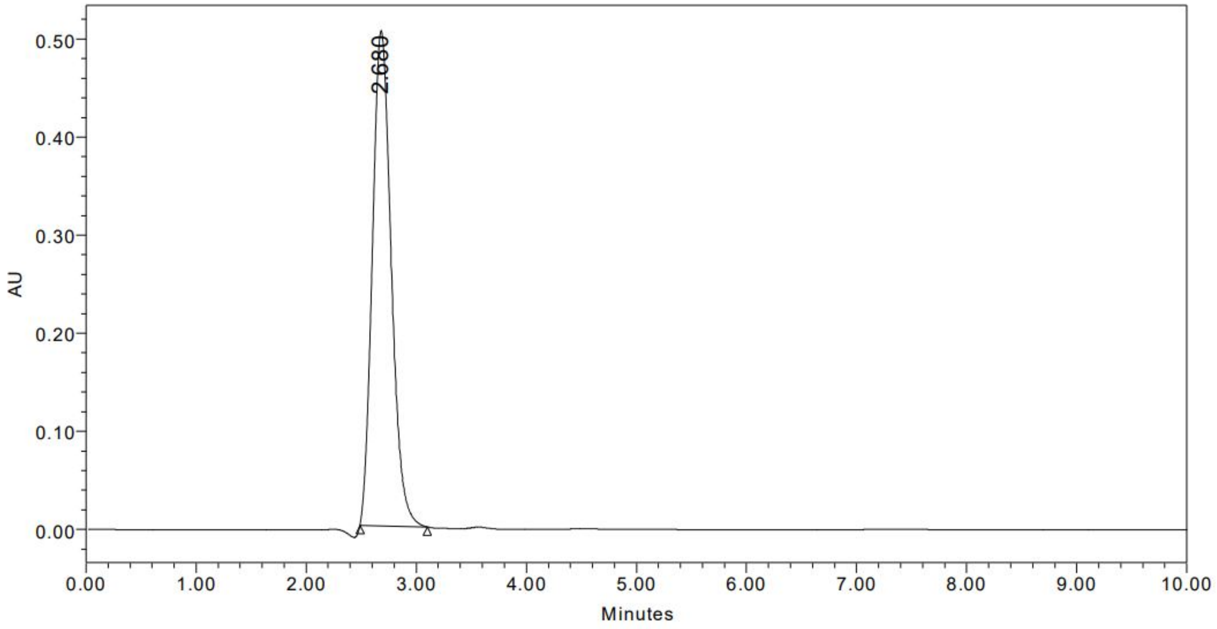

(37)

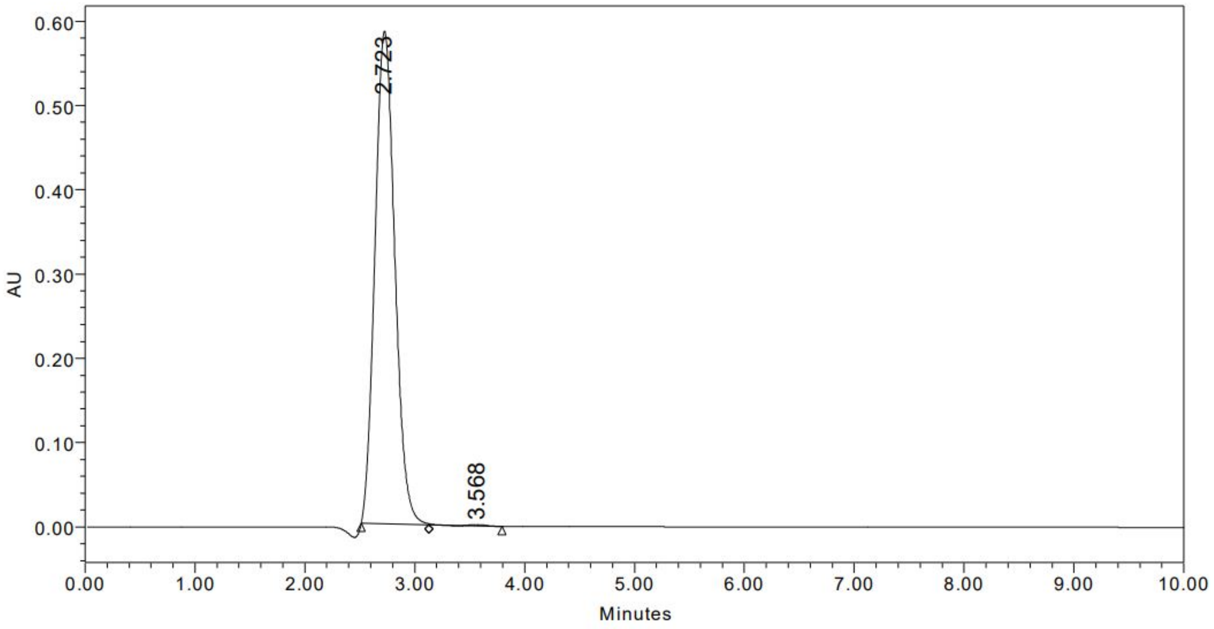
2.723
3.568
AU
0.60
0.50
0.40
0.30
0.20
0.10
0.00
0.00 1.00 2.00 3.00 4.00 5.00 6.00 7.00 8.00 9.00 10.00
Minutes

(38)

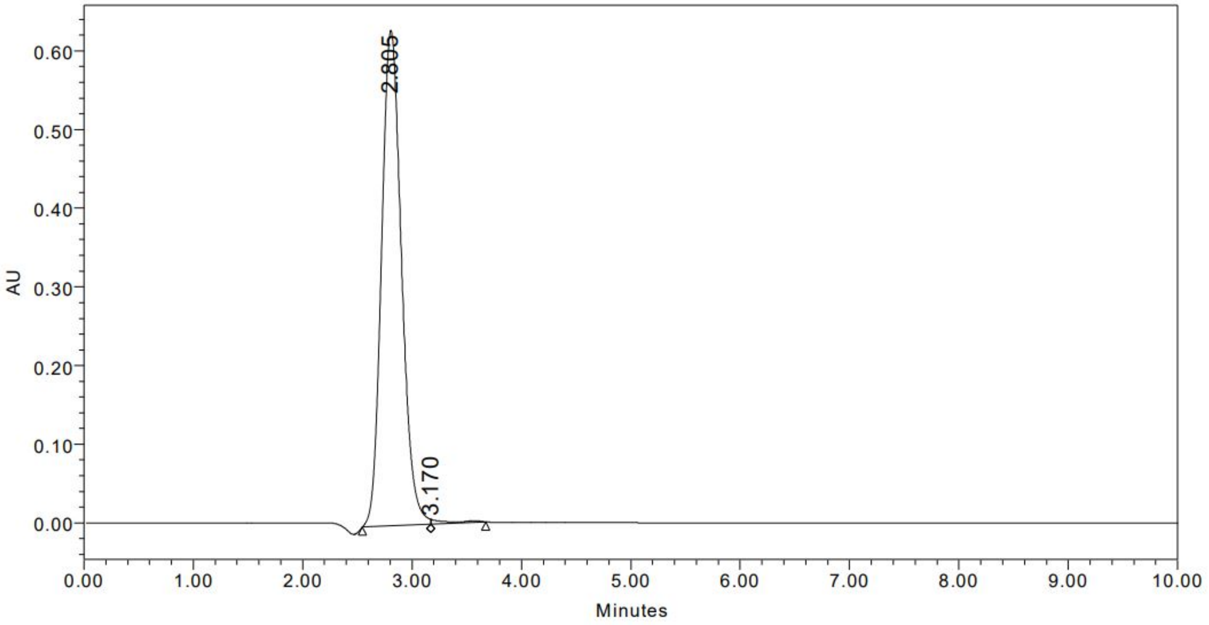
2.805
3.170
AU
0.60
0.50
0.40
0.30
0.20
0.10
0.00
0.00 1.00 2.00 3.00 4.00 5.00 6.00 7.00 8.00 9.00 10.00
Minutes

(39)

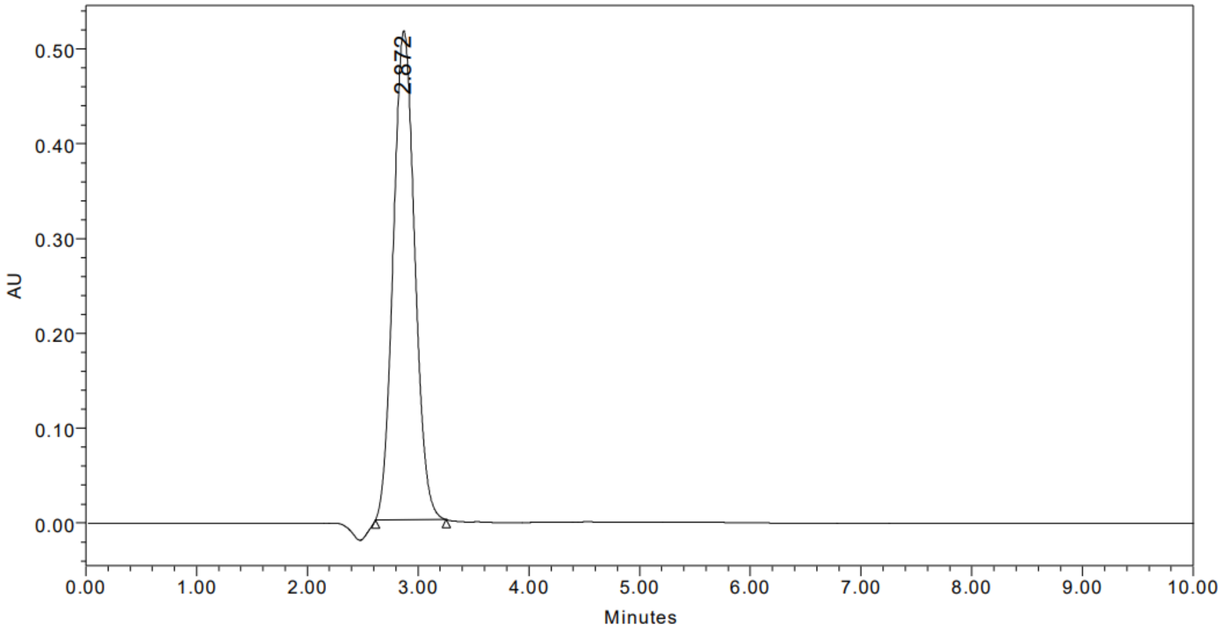
2.872
AU
0.50
0.40
0.30
0.20
0.10
0.00
0.00
1.00
2.00
3.00
4.00
5.00
6.00
7.00
8.00
9.00
10.00
Minutes

(40)

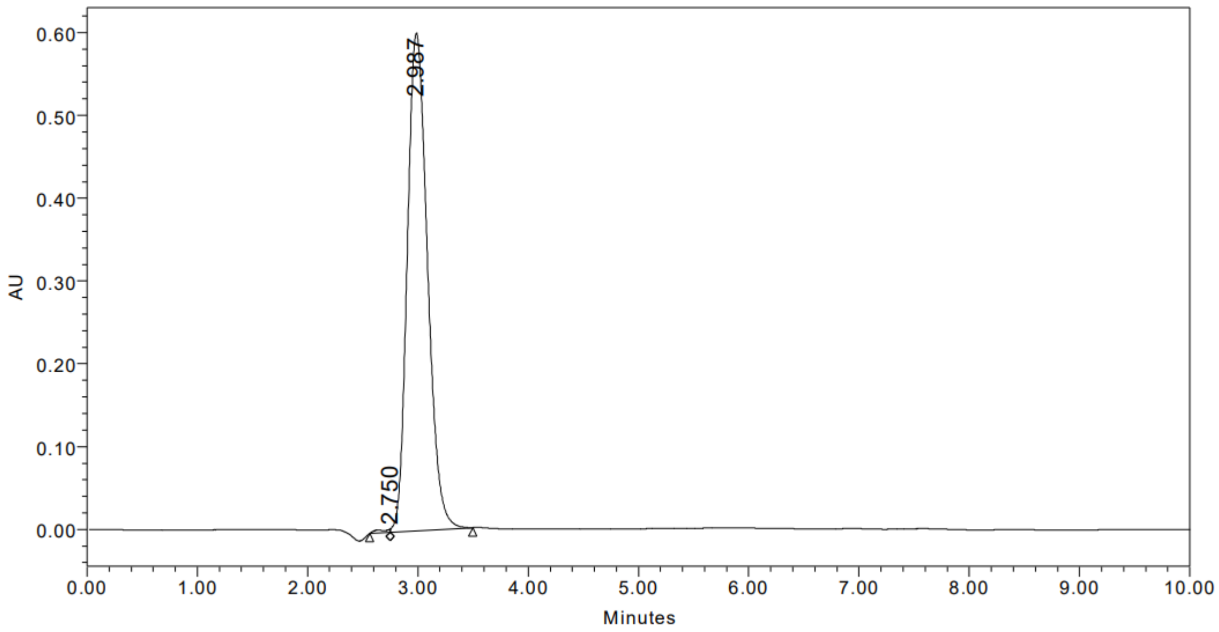
2.987
2.750
AU
0.60
0.50
0.40
0.30
0.20
0.10
0.00
0.00
1.00
2.00
3.00
4.00
5.00
6.00
7.00
8.00
9.00
10.00
Minutes

(41)

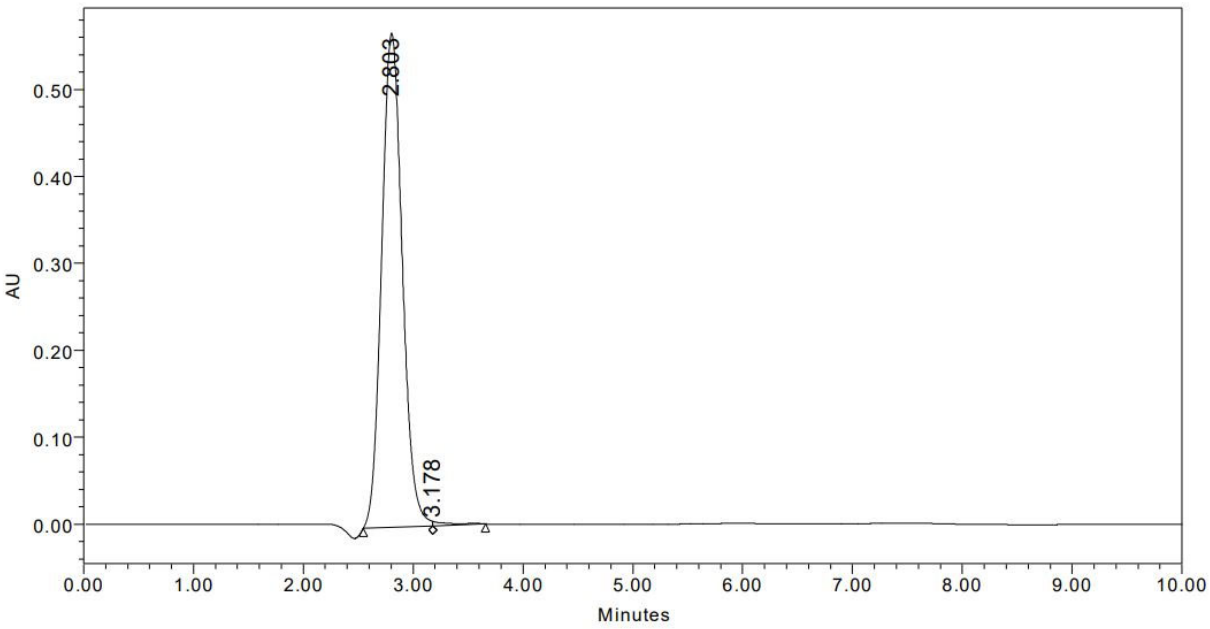

2.803
3.178
0.50
0.40
0.30
0.20
0.10
0.00
AU
0.00
1.00
2.00
3.00
4.00
5.00
6.00
7.00
8.00
9.00
10.00
Minutes

(42)

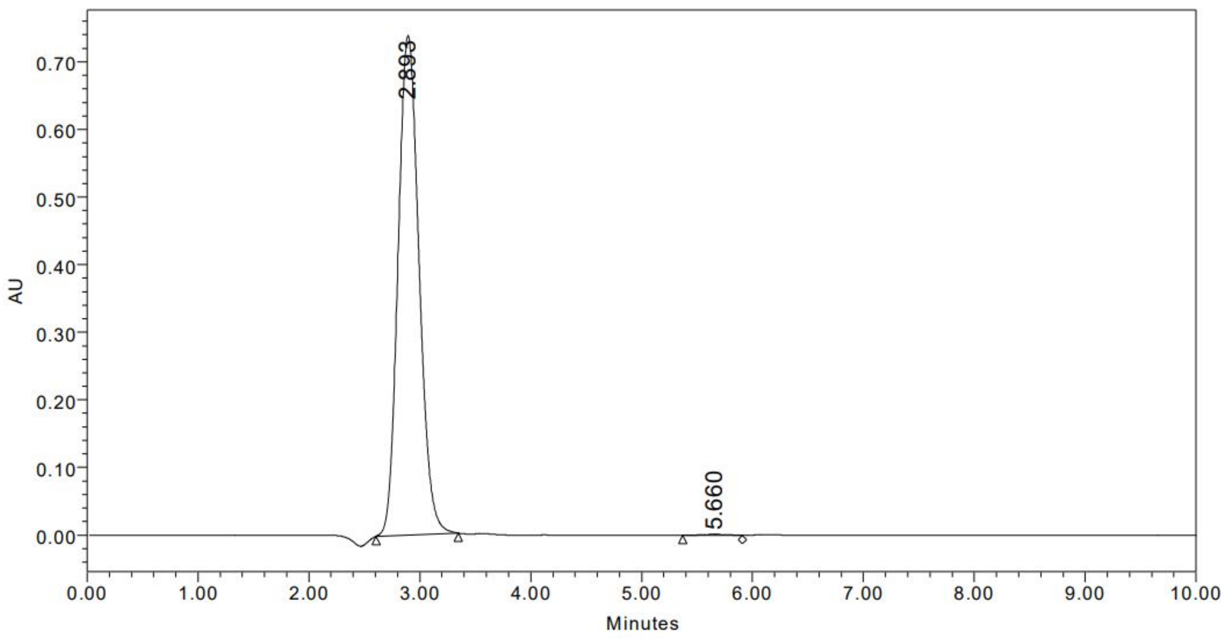

2.893
5.660
0.70
0.60
0.50
0.40
0.30
0.20
0.10
0.00
AU
0.00
1.00
2.00
3.00
4.00
5.00
6.00
7.00
8.00
9.00
10.00
Minutes

(43)

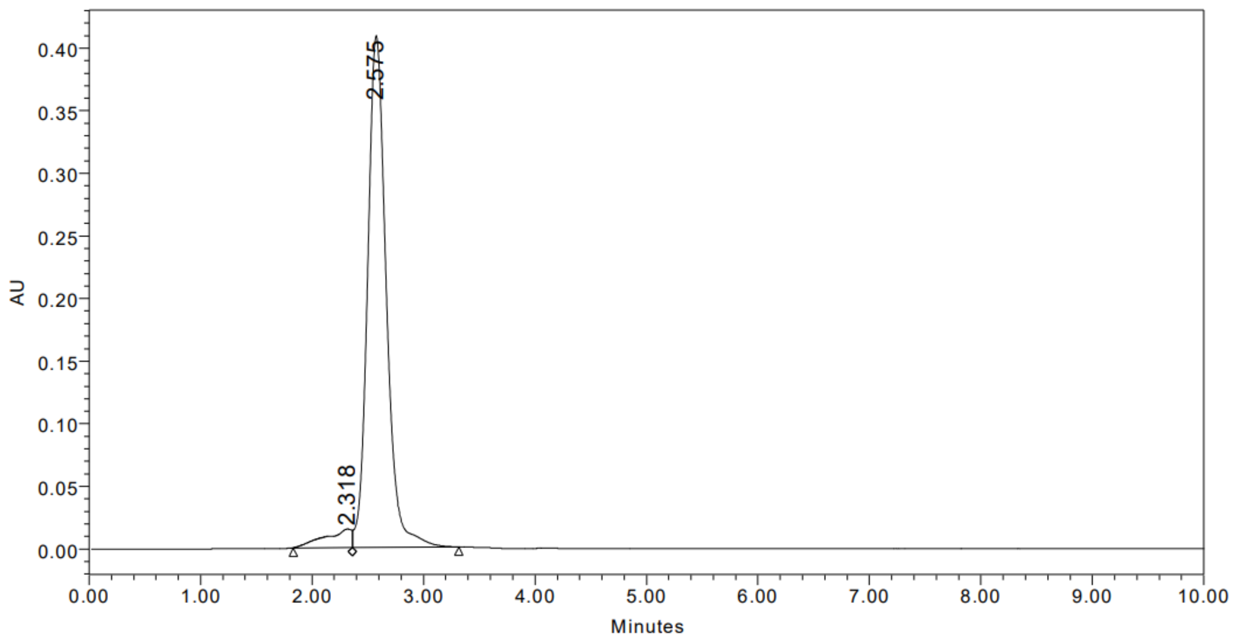

0.40
0.35
0.30
0.25
0.20
0.15
0.10
0.05
0.00
AU
2.575
2.318
0.00
1.00
2.00
3.00
4.00
5.00
6.00
7.00
8.00
9.00
10.00
Minutes

(44)

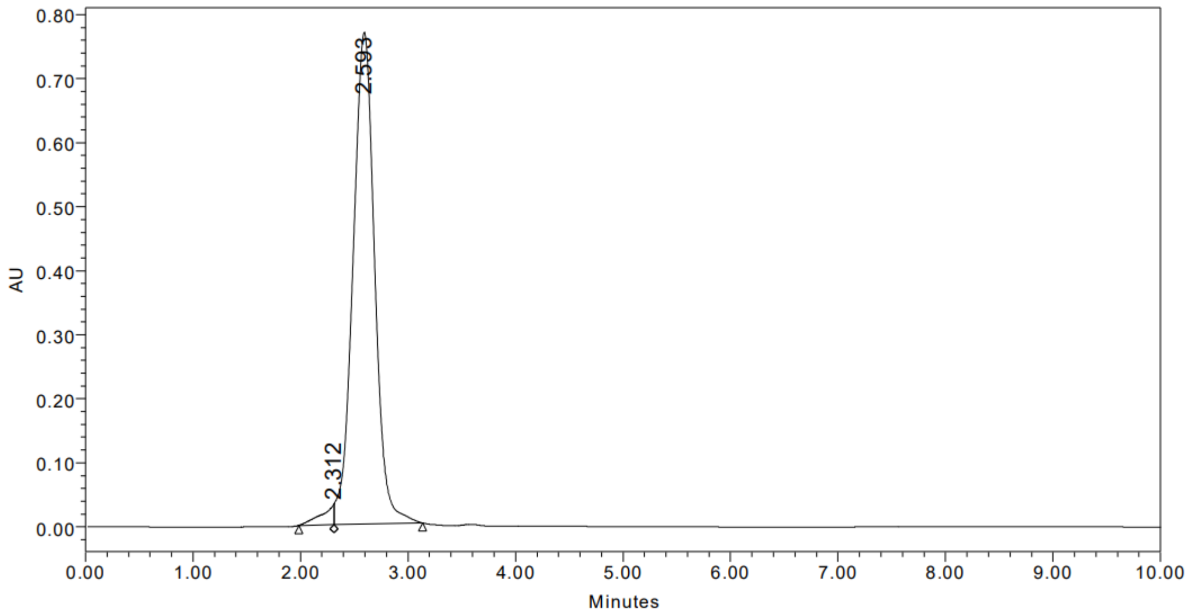

0.80
0.70
0.60
0.50
0.40
0.30
0.20
0.10
0.00
AU
2.593
2.312
0.00
1.00
2.00
3.00
4.00
5.00
6.00
7.00
8.00
9.00
10.00
Minutes

(45)

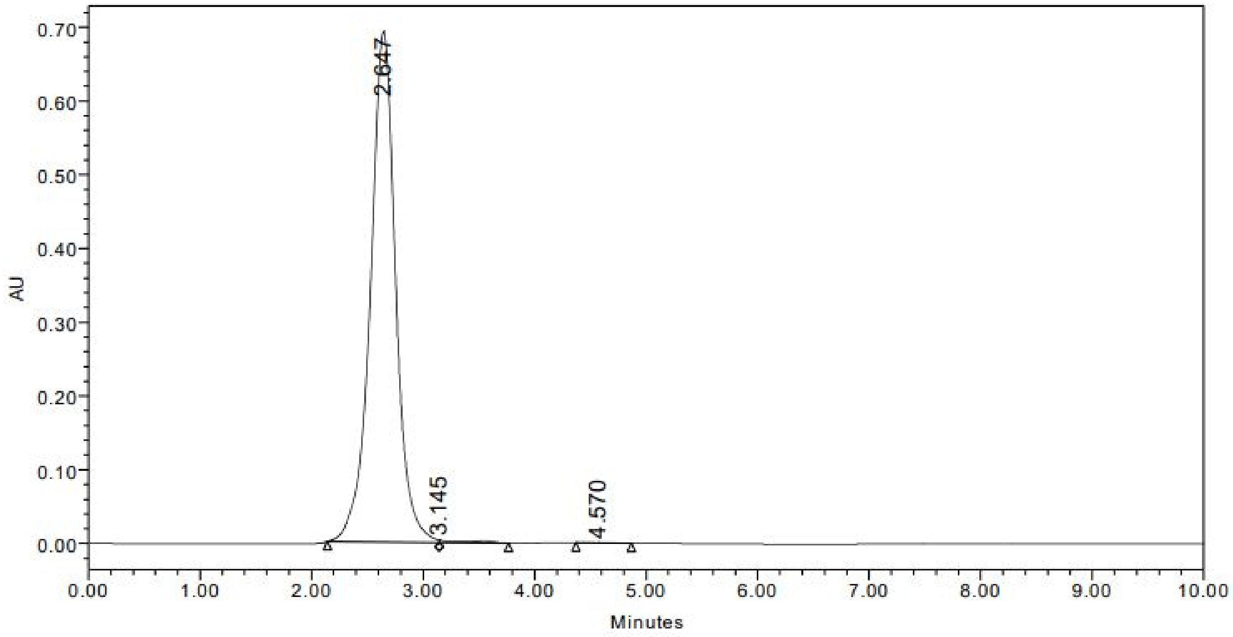

0.70
0.60
0.50
0.40
0.30
0.20
0.10
0.00
AU
2.647
3.145
4.570
0.00
1.00
2.00
3.00
4.00
5.00
6.00
7.00
8.00
9.00
10.00
Minutes

(46)

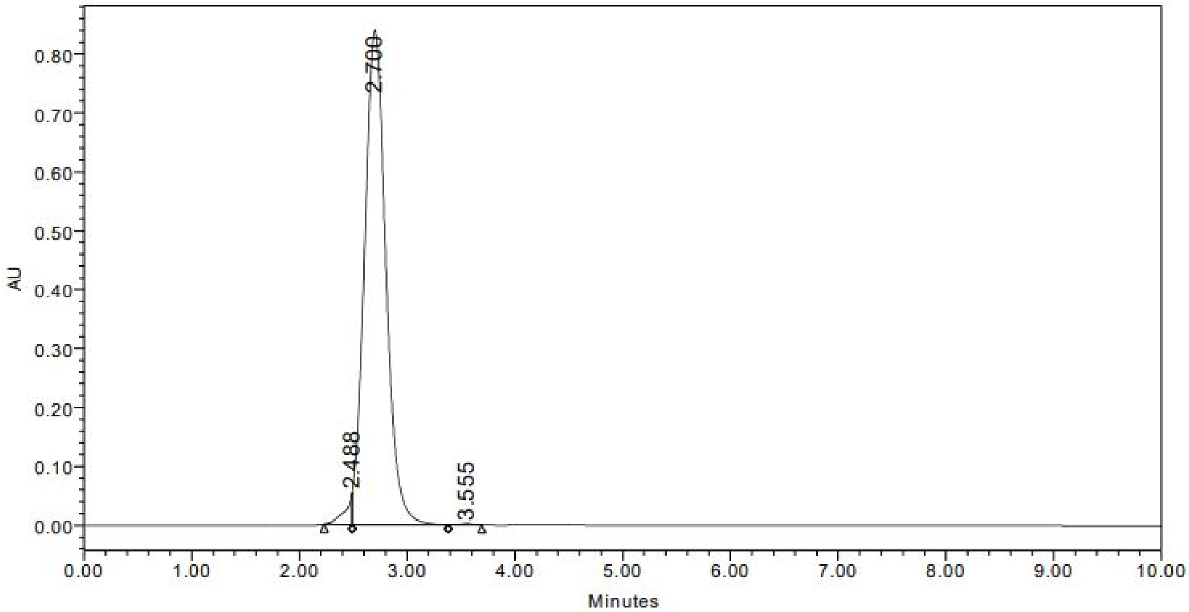

0.80
0.70
0.60
0.50
0.40
0.30
0.20
0.10
0.00
AU
2.700
2.488
3.555
0.00
1.00
2.00
3.00
4.00
5.00
6.00
7.00
8.00
9.00
10.00
Minutes

(47)

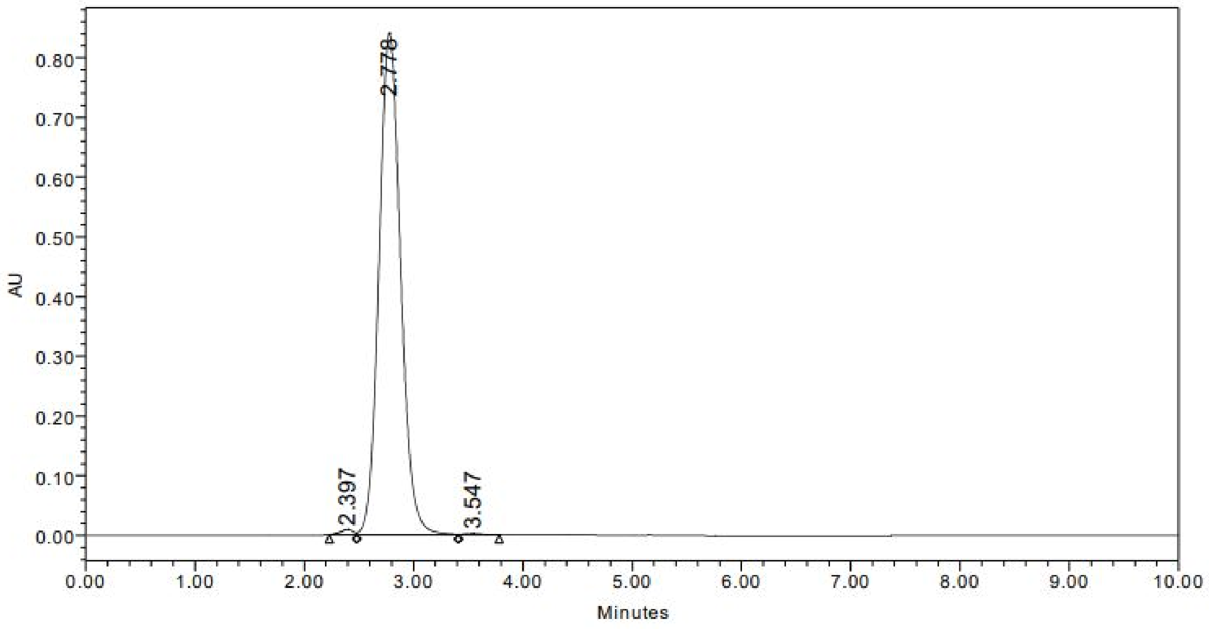
0.80
0.70
0.60
0.50
0.40
0.30
0.20
0.10
0.00
AU
2.778
2.397
3.547
0.00
1.00
2.00
3.00
4.00
5.00
6.00
7.00
8.00
9.00
10.00
Minutes

(48)

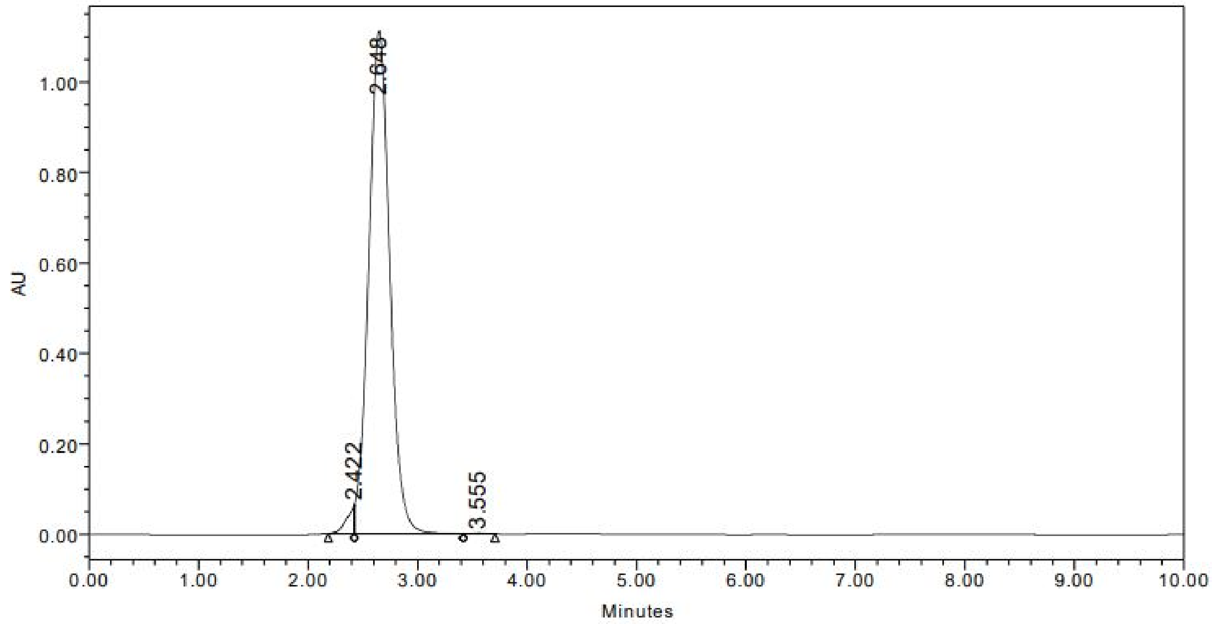
1.00
0.80
0.60
0.40
0.20
0.00
AU
2.648
2.422
3.555
0.00
1.00
2.00
3.00
4.00
5.00
6.00
7.00
8.00
9.00
10.00
Minutes

(49)

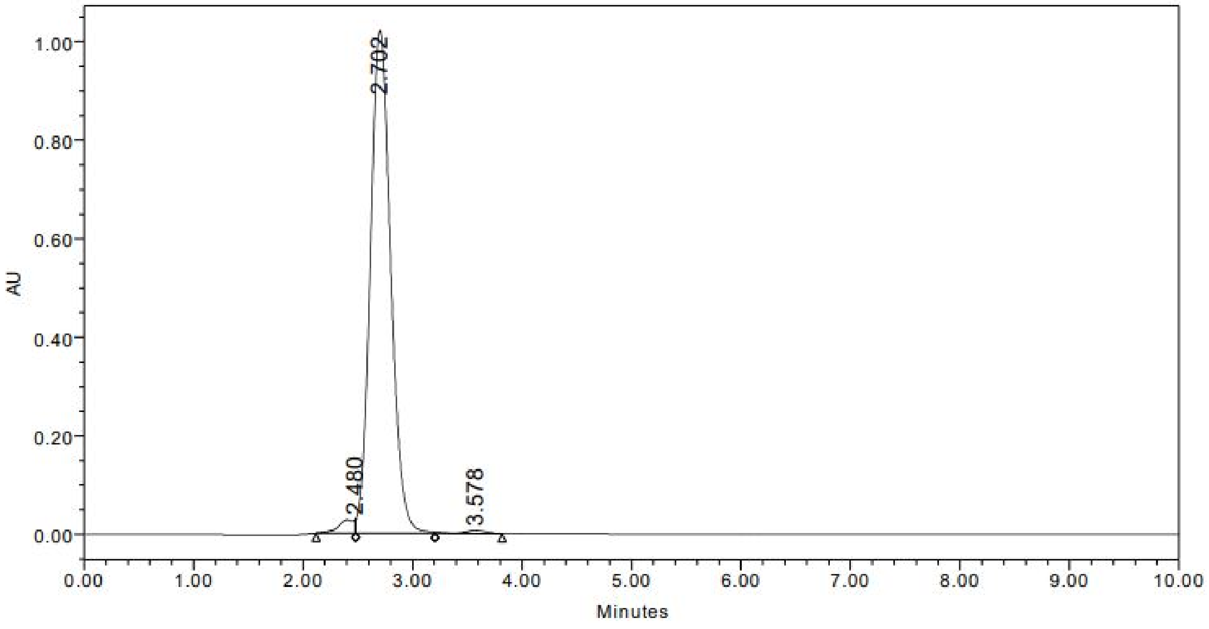

(50)

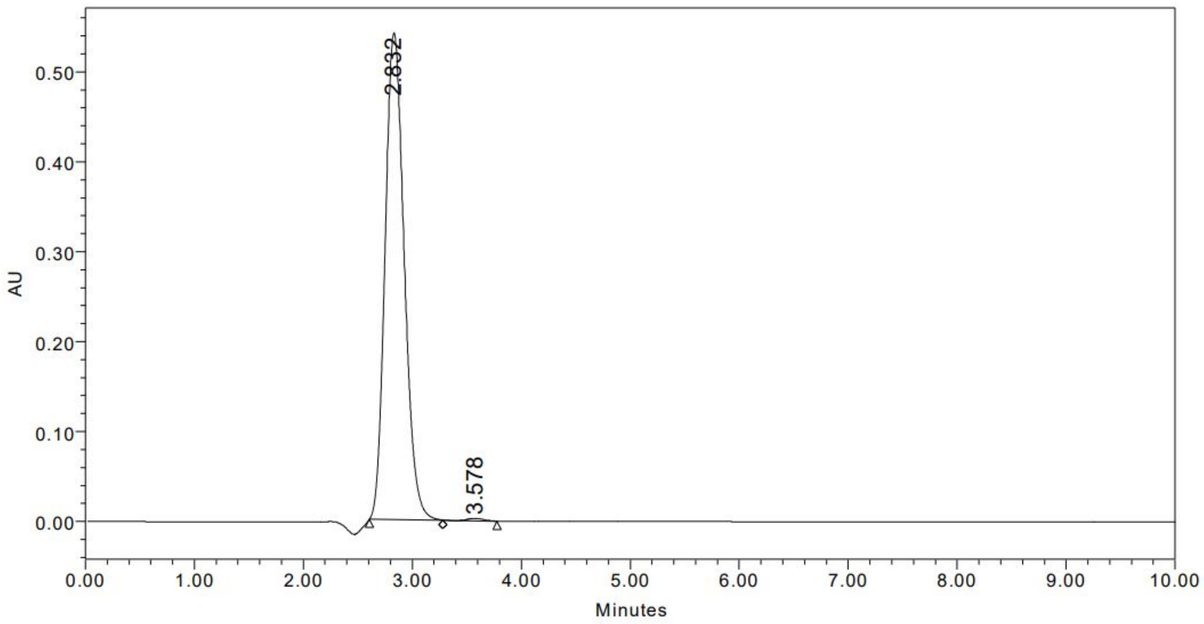

(51)

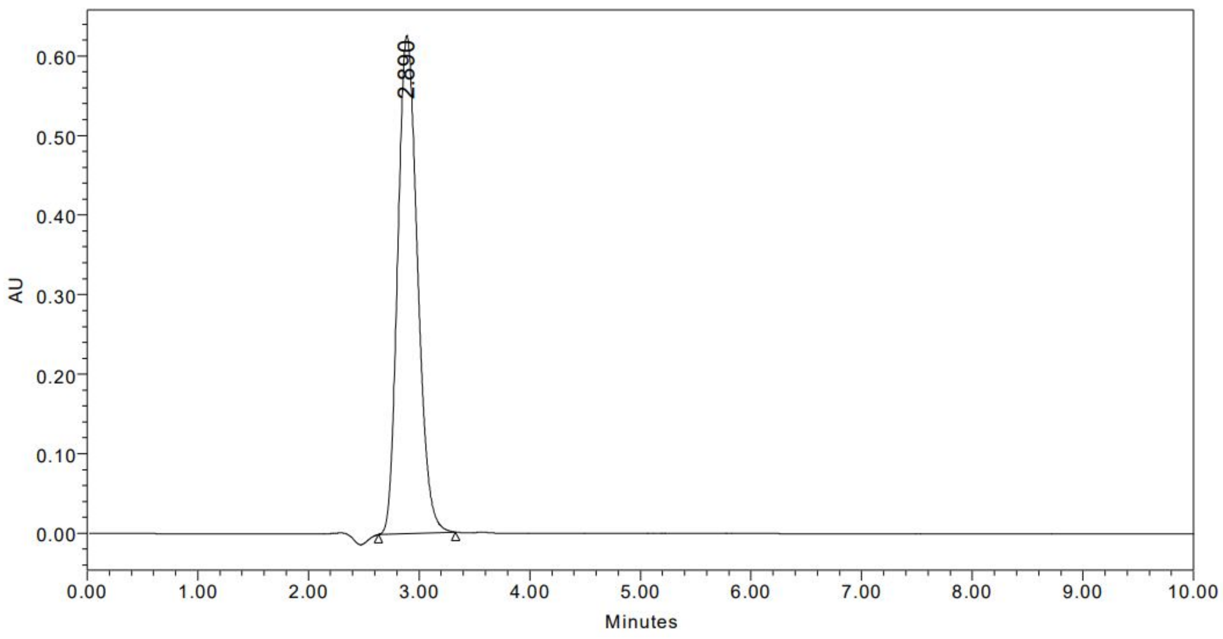
2.890
0.60
0.50
0.40
0.30
0.20
0.10
0.00
AU
0.00
1.00
2.00
3.00
4.00
5.00
6.00
7.00
8.00
9.00
10.00
Minutes

(52)

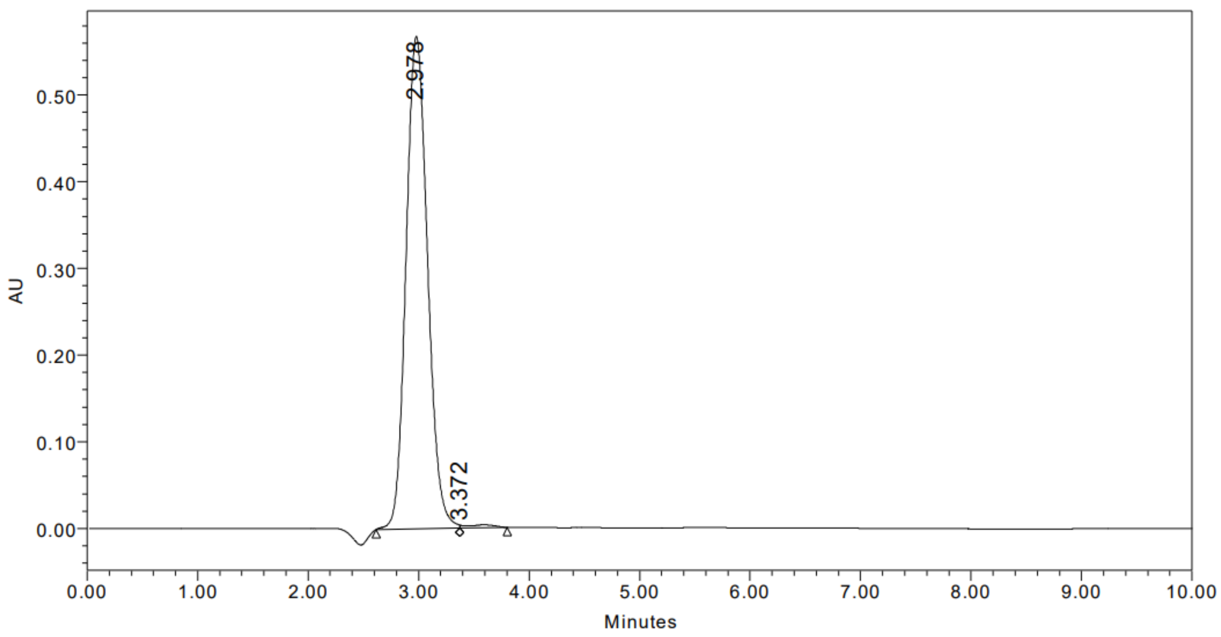
2.978
3.372
0.50
0.40
0.30
0.20
0.10
0.00
AU
0.00
1.00
2.00
3.00
4.00
5.00
6.00
7.00
8.00
9.00
10.00
Minutes

(53)

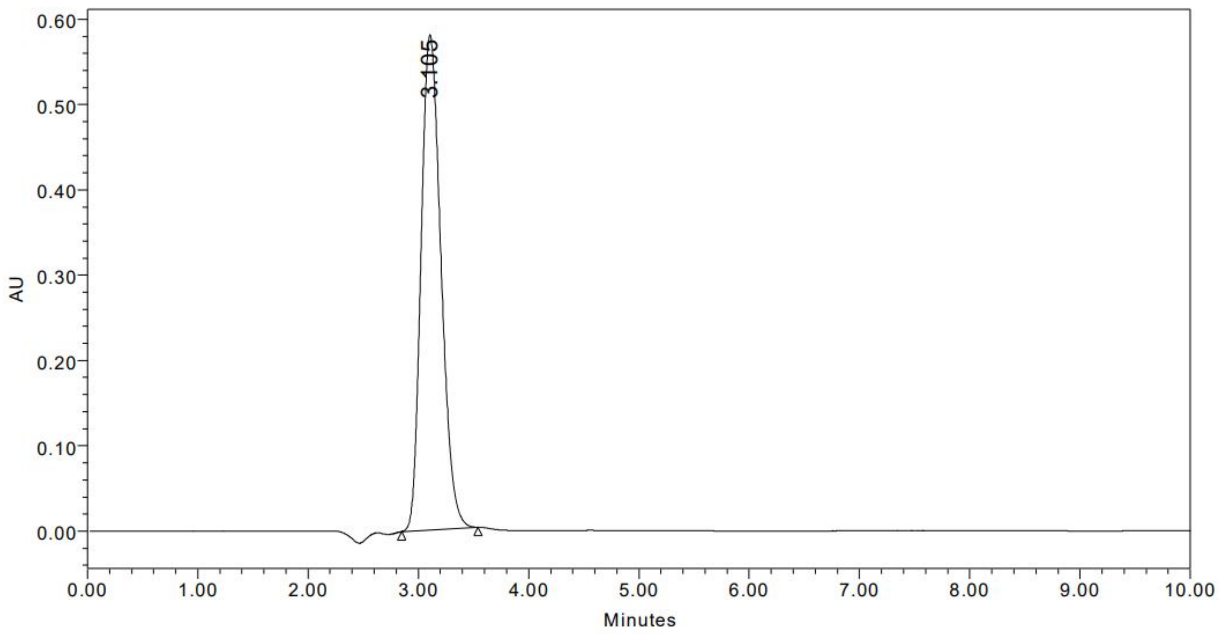
3.105
AU
0.60
0.50
0.40
0.30
0.20
0.10
0.00
0.00
1.00
2.00
3.00
4.00
5.00
6.00
7.00
8.00
9.00
10.00
Minutes

(54)

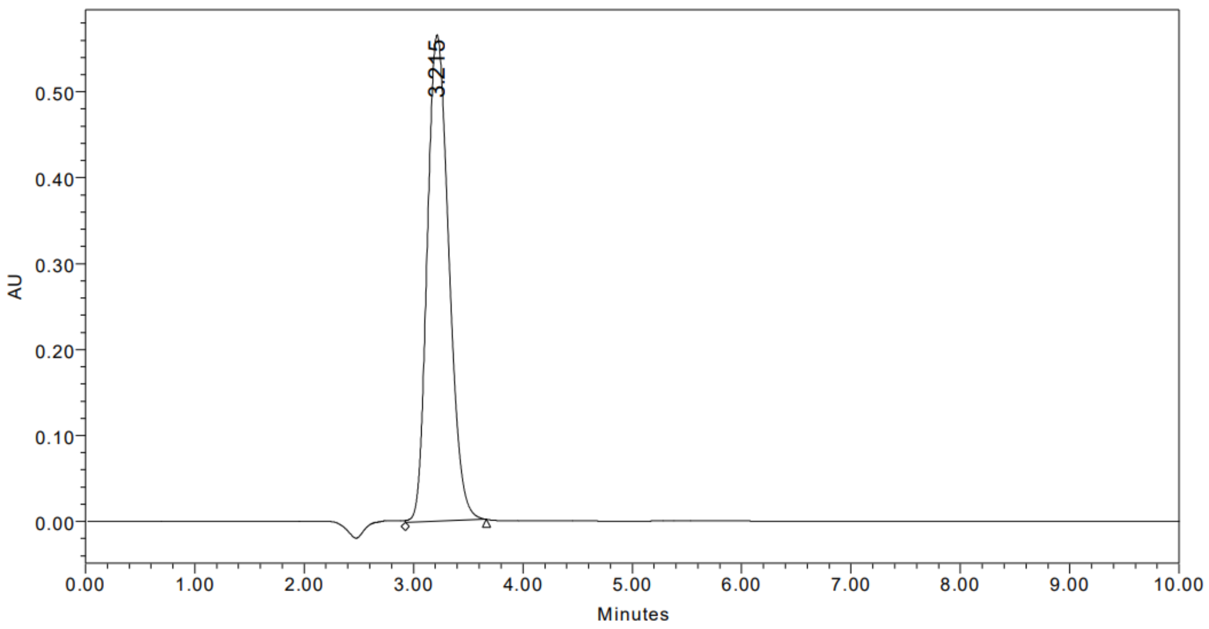
3.215
AU
0.50
0.40
0.30
0.20
0.10
0.00
0.00
1.00
2.00
3.00
4.00
5.00
6.00
7.00
8.00
9.00
10.00
Minutes

(55)

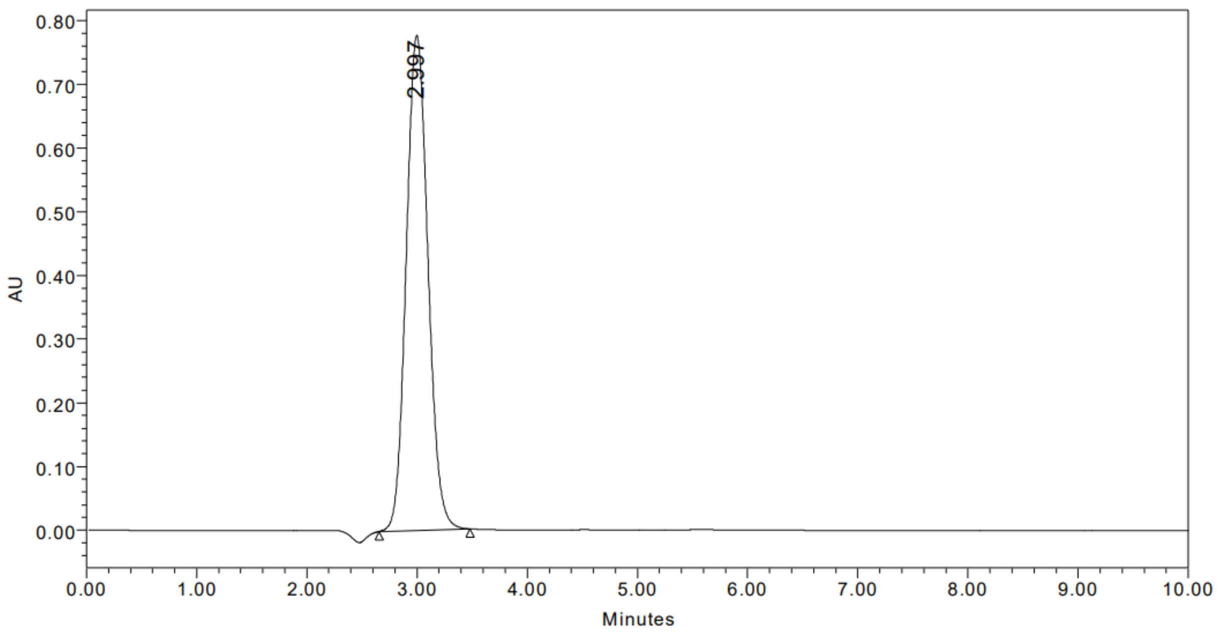

2.997
AU
0.80
0.70
0.60
0.50
0.40
0.30
0.20
0.10
0.00
0.00
1.00
2.00
3.00
4.00
5.00
6.00
7.00
8.00
9.00
10.00
Minutes

(56)

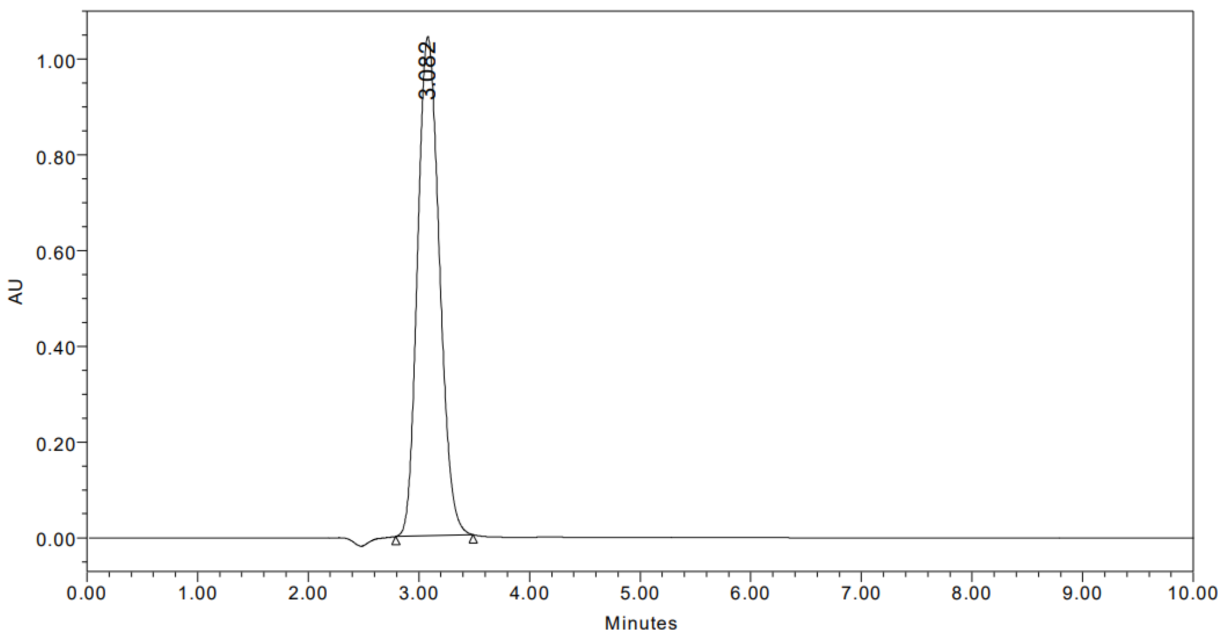

3.082
AU
1.00
0.80
0.60
0.40
0.20
0.00
0.00
1.00
2.00
3.00
4.00
5.00
6.00
7.00
8.00
9.00
10.00
Minutes

(57)

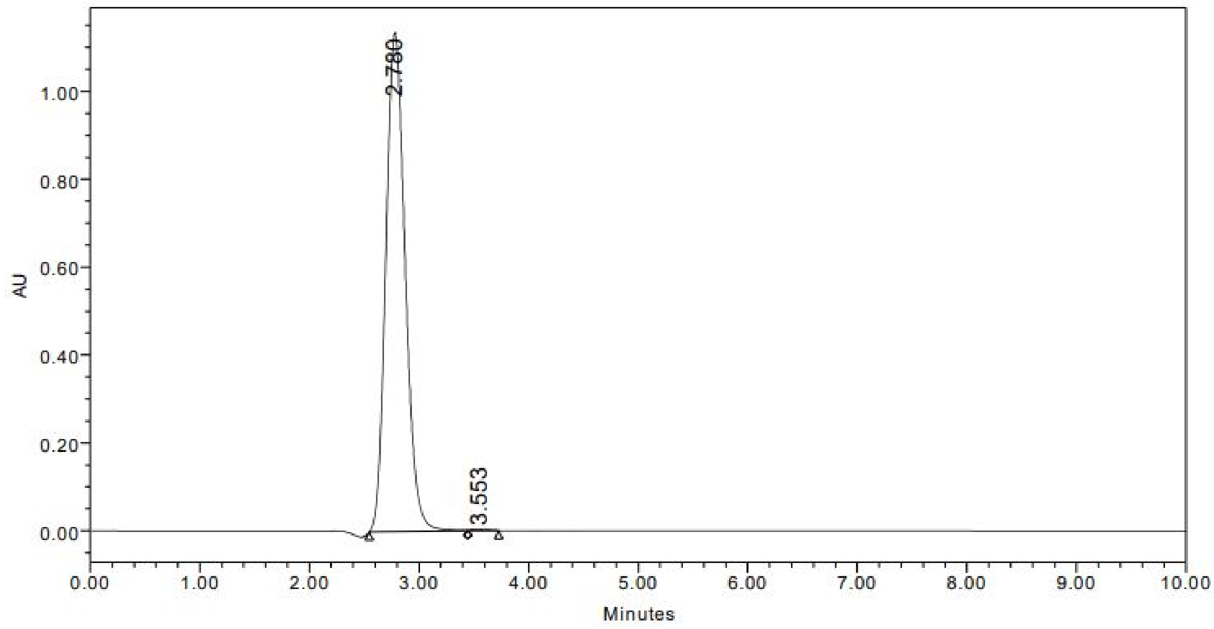

2.780
3.553
1.00
0.80
0.60
0.40
0.20
0.00
AU
0.00
1.00
2.00
3.00
4.00
5.00
6.00
7.00
8.00
9.00
10.00
Minutes

(58)

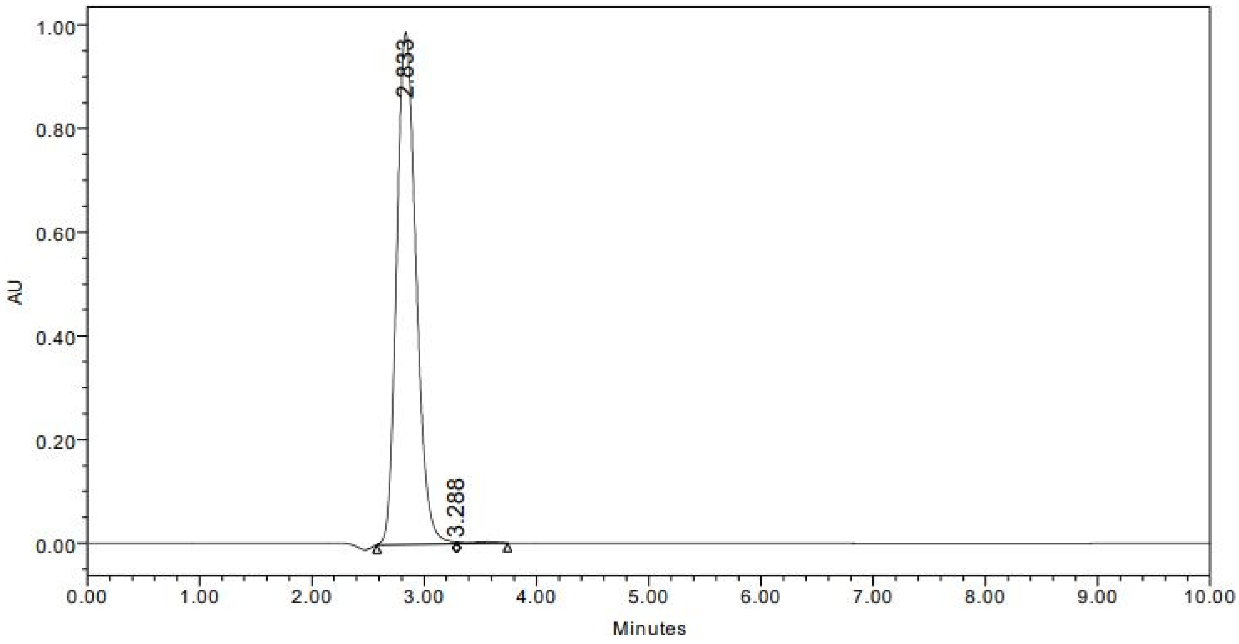

2.833
3.288
1.00
0.80
0.60
0.40
0.20
0.00
AU
0.00
1.00
2.00
3.00
4.00
5.00
6.00
7.00
8.00
9.00
10.00
Minutes

(59)

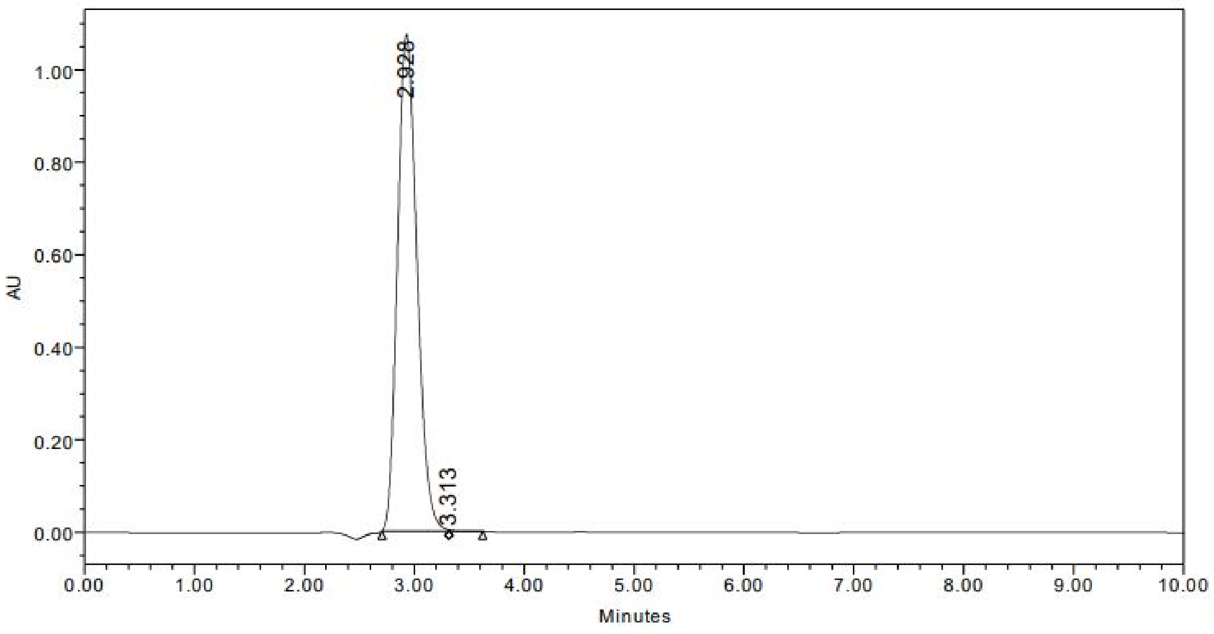

(60)

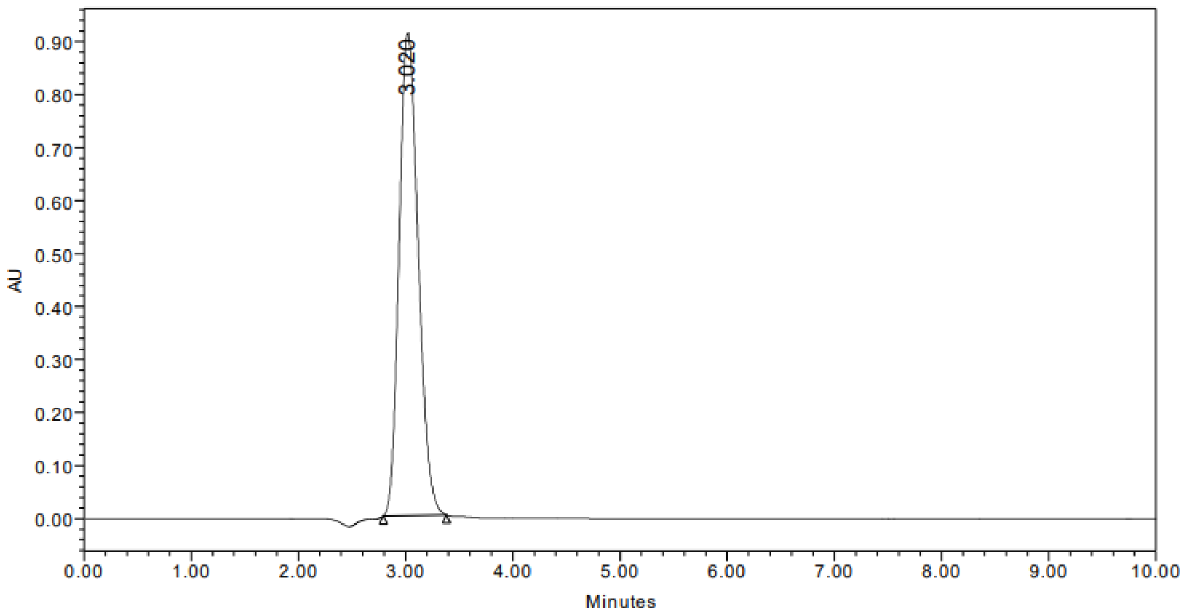

(61)

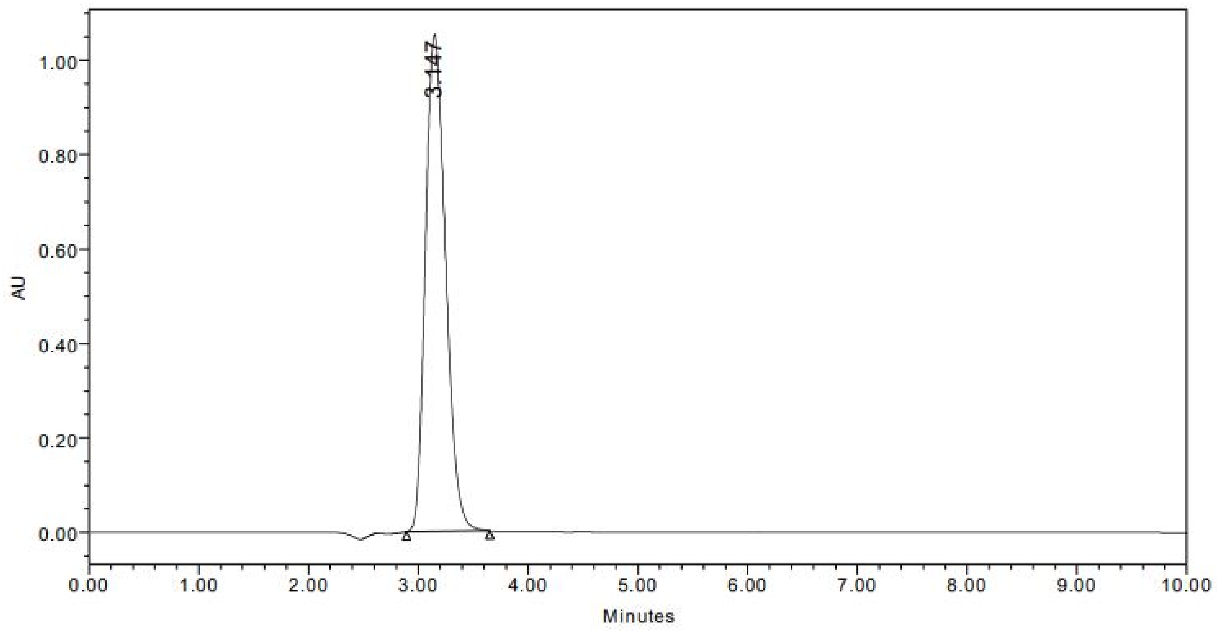
3.147
AU
1.00
0.80
0.60
0.40
0.20
0.00
0.00 1.00 2.00 3.00 4.00 5.00 6.00 7.00 8.00 9.00 10.00
Minutes

(62)

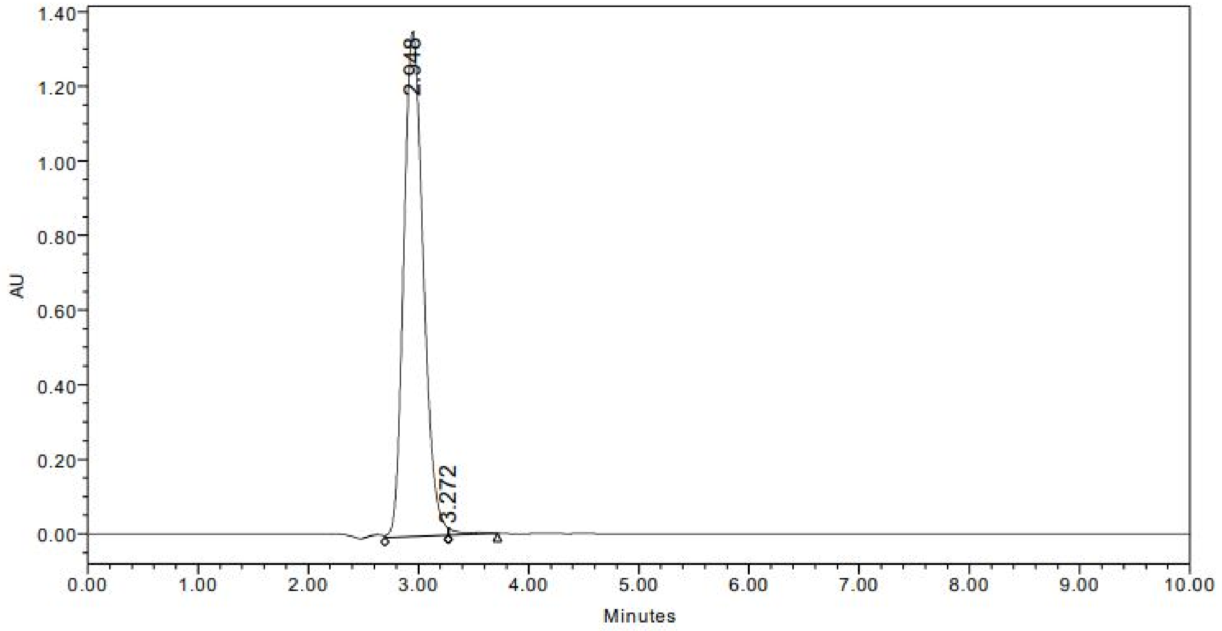
2.948
3.272
AU
1.40
1.20
1.00
0.80
0.60
0.40
0.20
0.00
0.00 1.00 2.00 3.00 4.00 5.00 6.00 7.00 8.00 9.00 10.00
Minutes

(63)

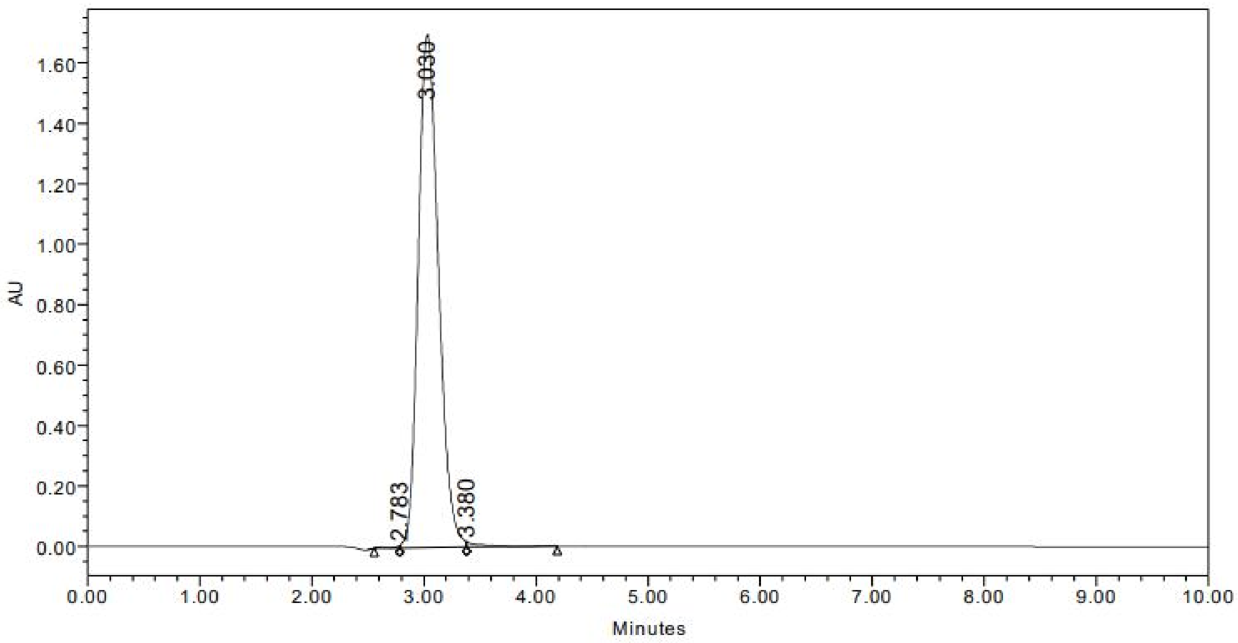

(64)

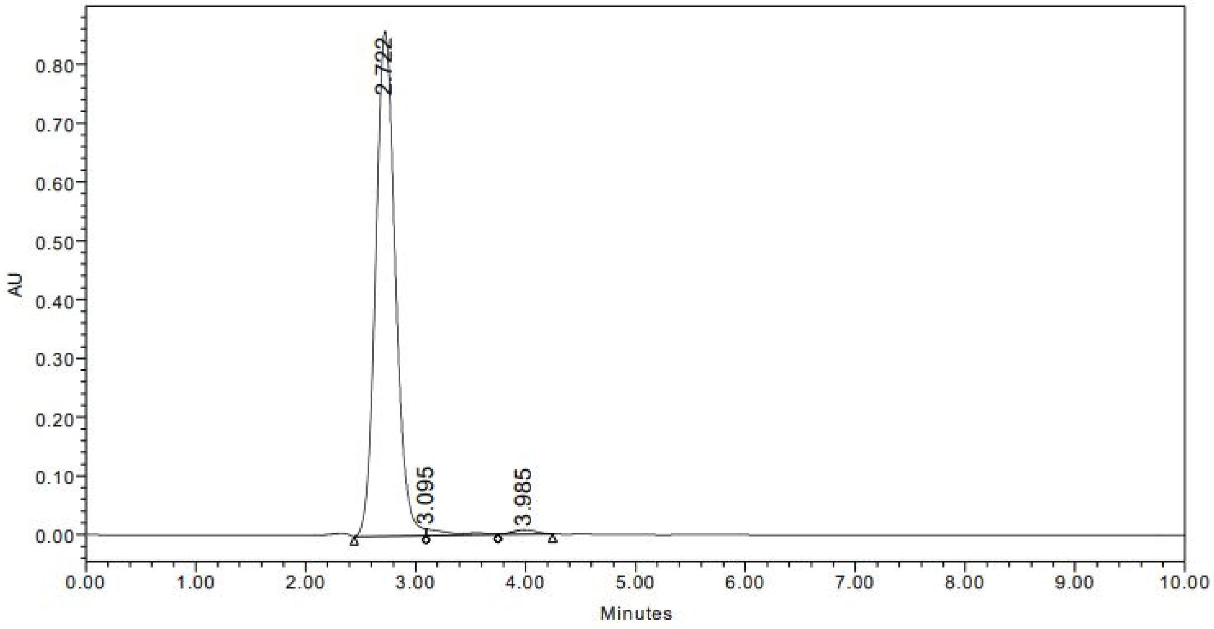

(65)

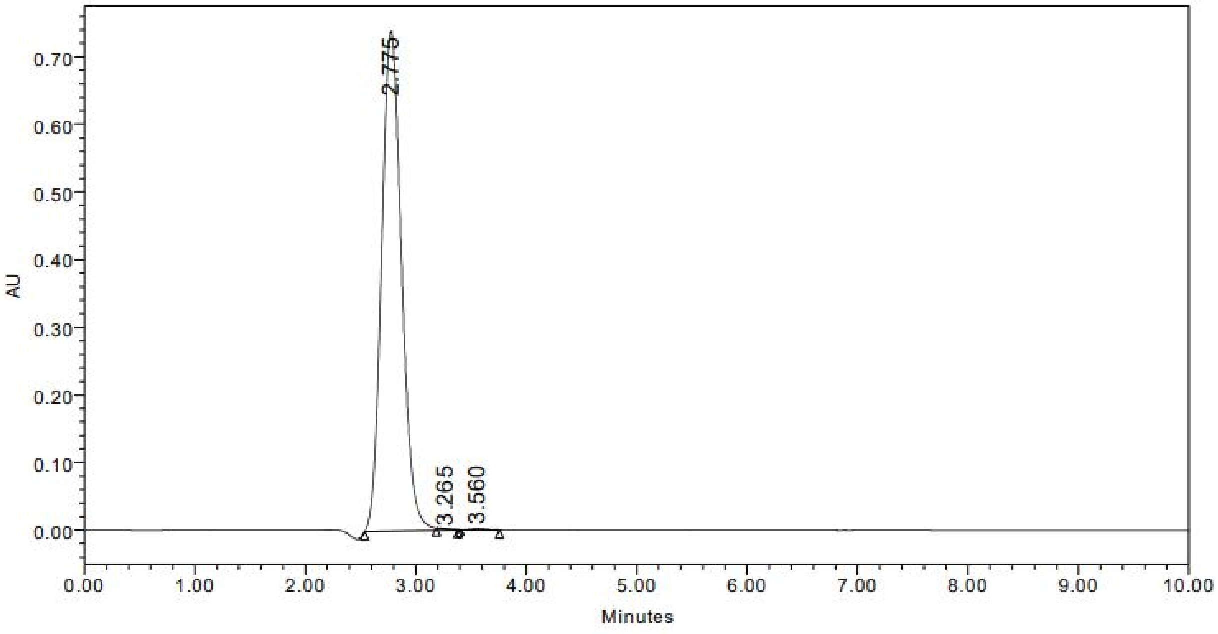
2.775
3.265
3.560
AU
0.70
0.60
0.50
0.40
0.30
0.20
0.10
0.00
0.00 1.00 2.00 3.00 4.00 5.00 6.00 7.00 8.00 9.00 10.00
Minutes

(66)

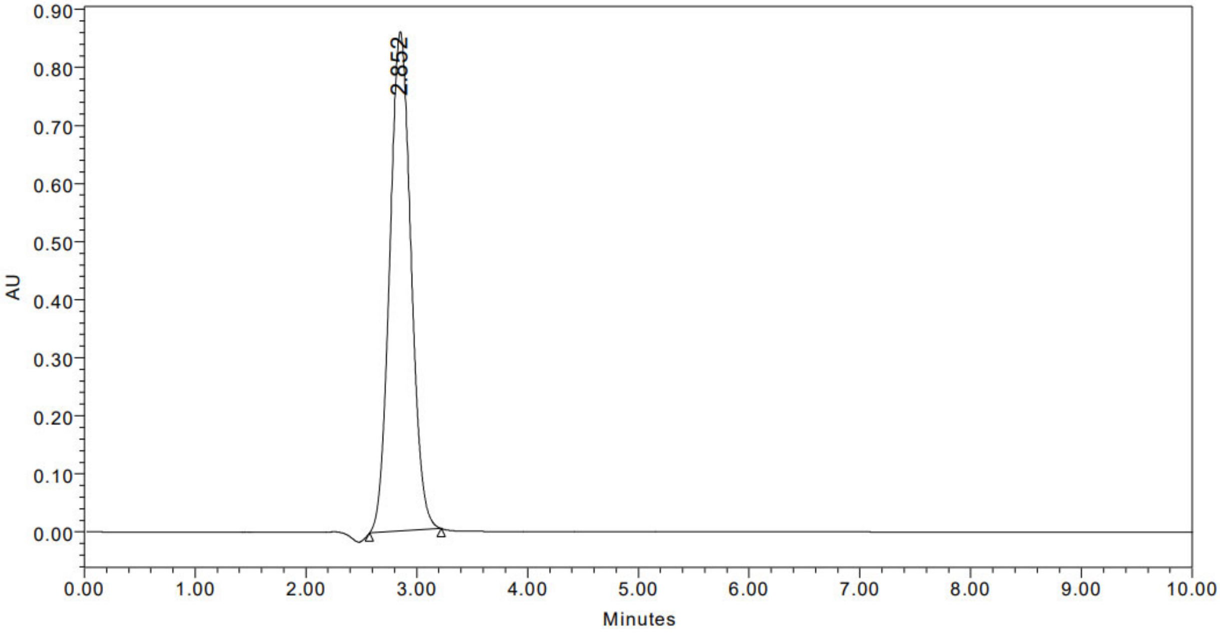
2.852
AU
0.90
0.80
0.70
0.60
0.50
0.40
0.30
0.20
0.10
0.00
0.00 1.00 2.00 3.00 4.00 5.00 6.00 7.00 8.00 9.00 10.00
Minutes

(67)

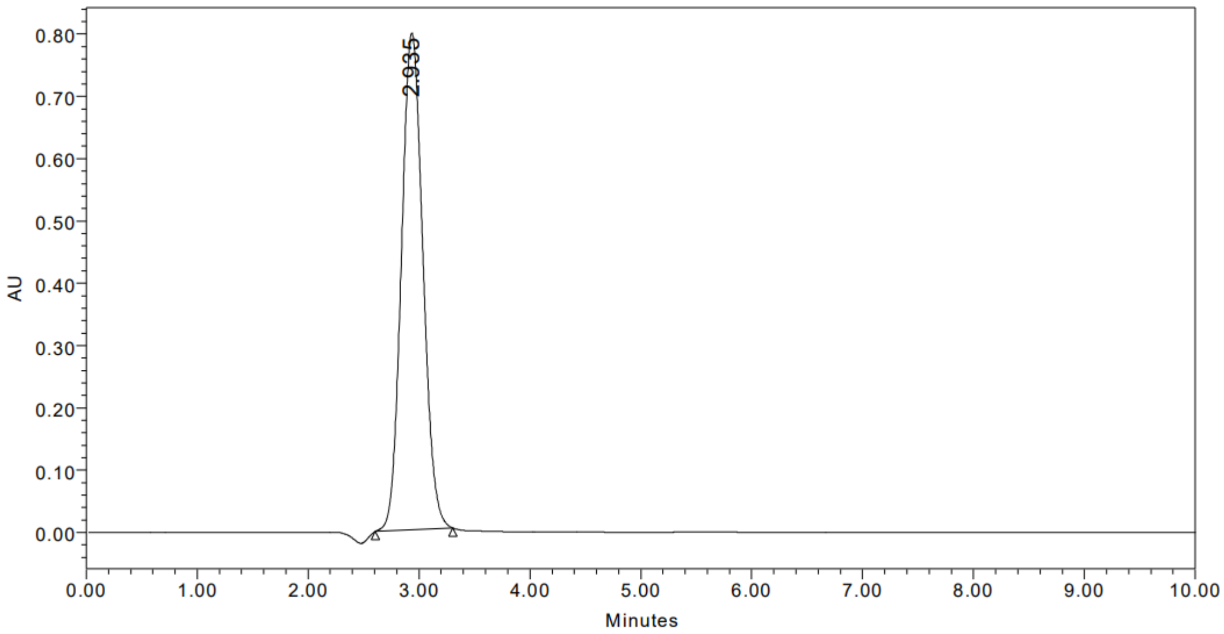

2.935
0.80
0.70
0.60
0.50
0.40
0.30
0.20
0.10
0.00
AU
0.00
1.00
2.00
3.00
4.00
5.00
6.00
7.00
8.00
9.00
10.00
Minutes

(68)

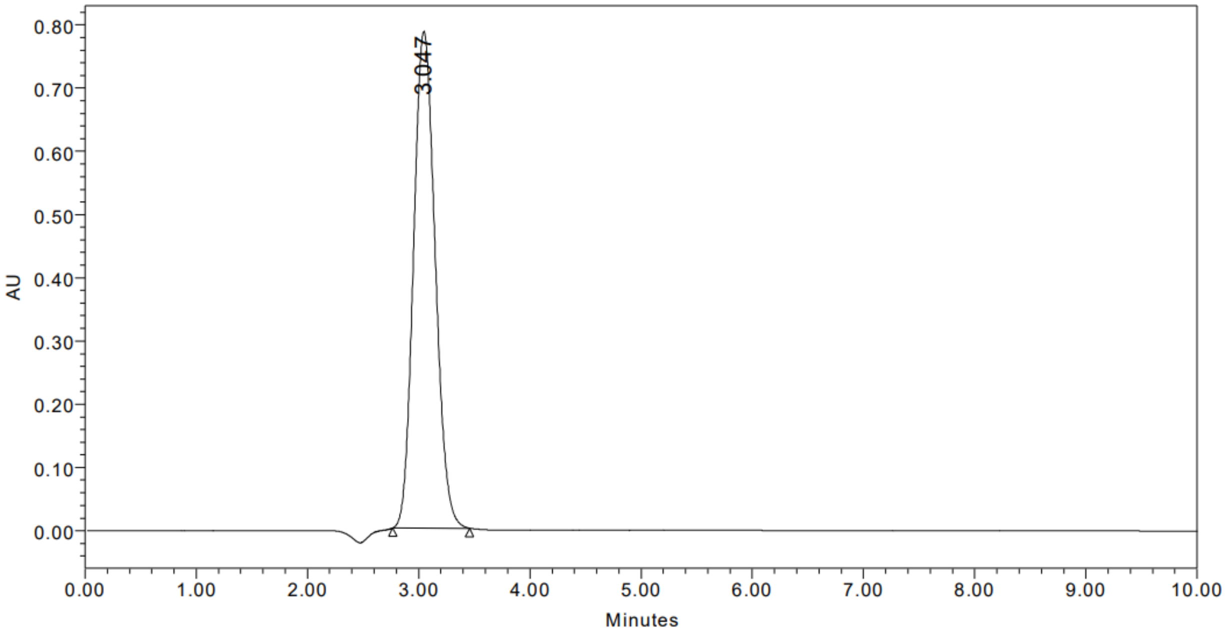

3.047
0.80
0.70
0.60
0.50
0.40
0.30
0.20
0.10
0.00
AU
0.00
1.00
2.00
3.00
4.00
5.00
6.00
7.00
8.00
9.00
10.00
Minutes

(69)

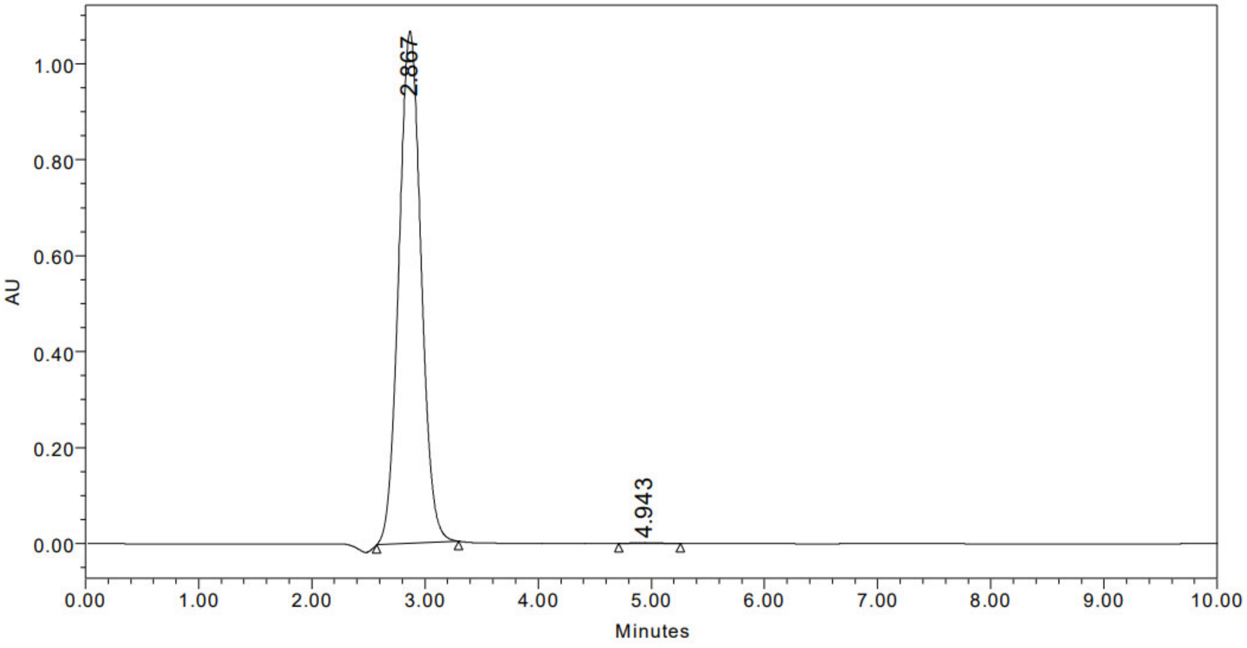

2.867
4.943
1.00
0.80
0.60
0.40
0.20
0.00
AU
0.00 1.00 2.00 3.00 4.00 5.00 6.00 7.00 8.00 9.00 10.00
Minutes

(70)

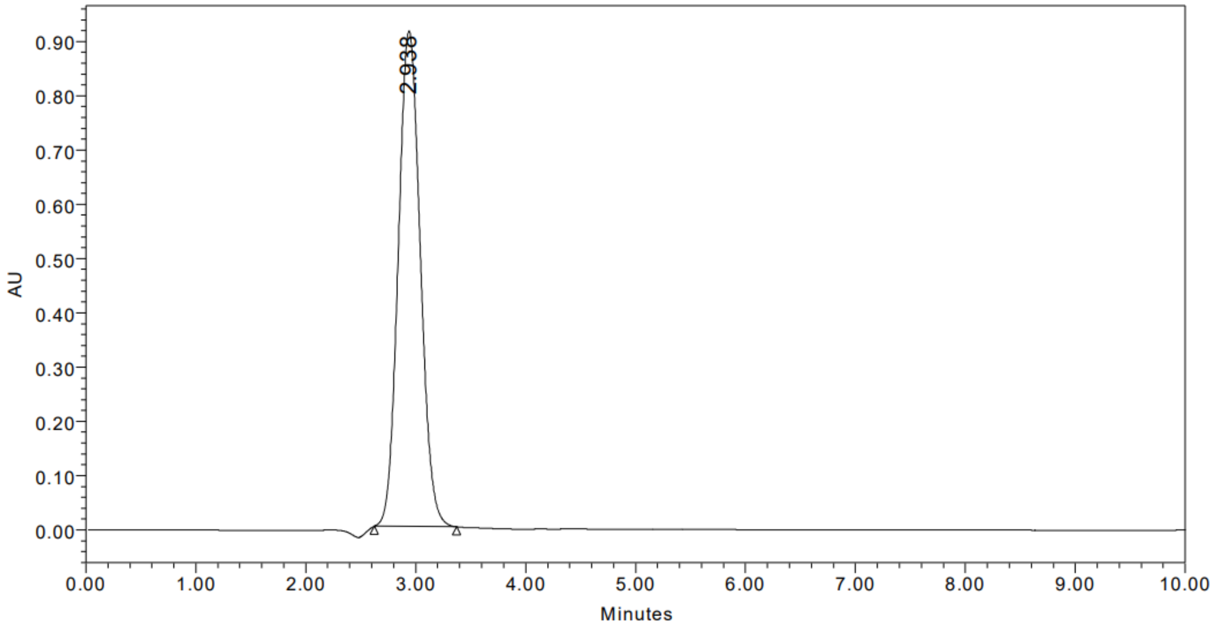

2.938
0.90
0.80
0.70
0.60
0.50
0.40
0.30
0.20
0.10
0.00
AU
0.00 1.00 2.00 3.00 4.00 5.00 6.00 7.00 8.00 9.00 10.00
Minutes

(71)

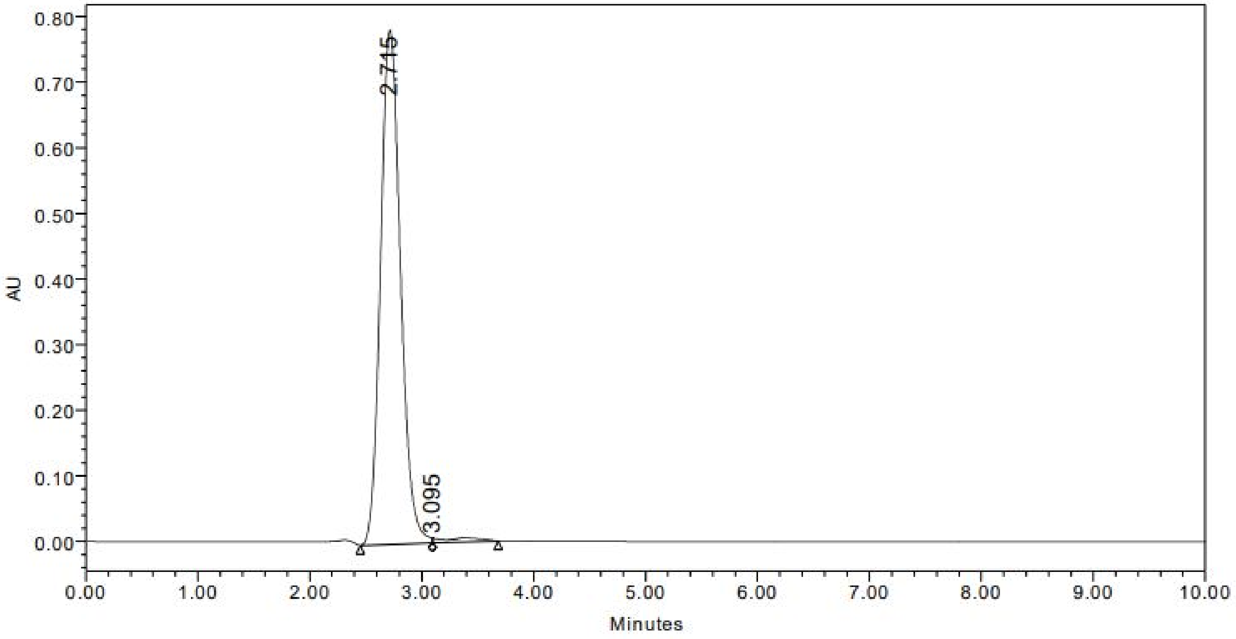

0.80
0.70
0.60
0.50
0.40
0.30
0.20
0.10
0.00
AU
2.715
3.095
0.00
1.00
2.00
3.00
4.00
5.00
6.00
7.00
8.00
9.00
10.00
Minutes

(72)

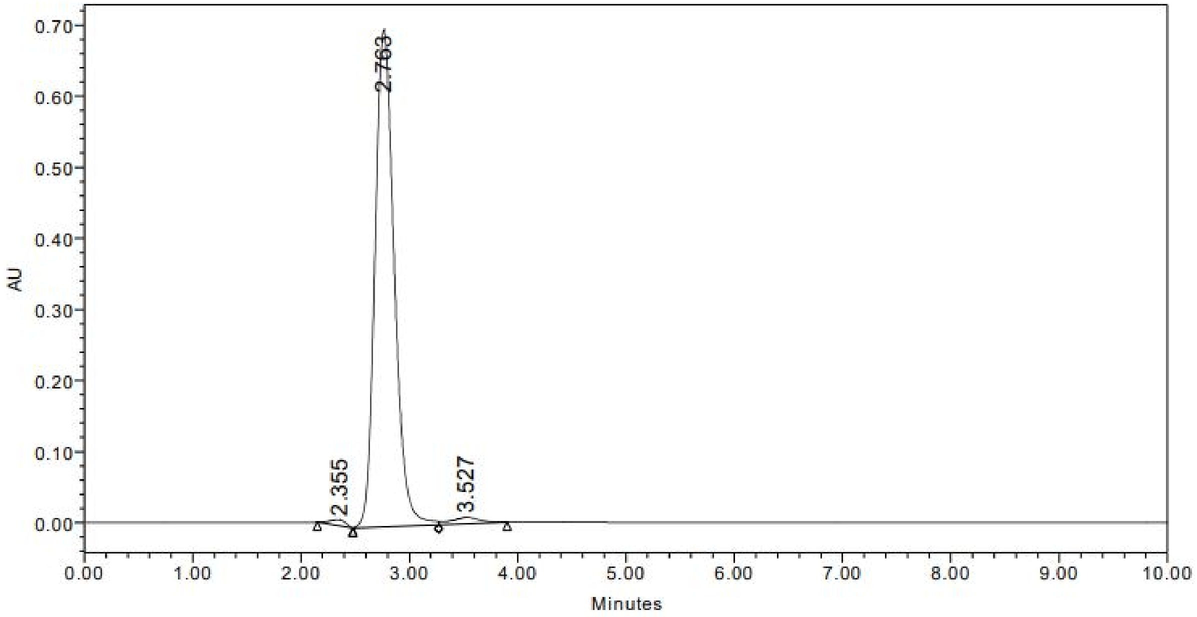

0.70
0.60
0.50
0.40
0.30
0.20
0.10
0.00
AU
2.355
2.763
3.527
0.00
1.00
2.00
3.00
4.00
5.00
6.00
7.00
8.00
9.00
10.00
Minutes

(73)

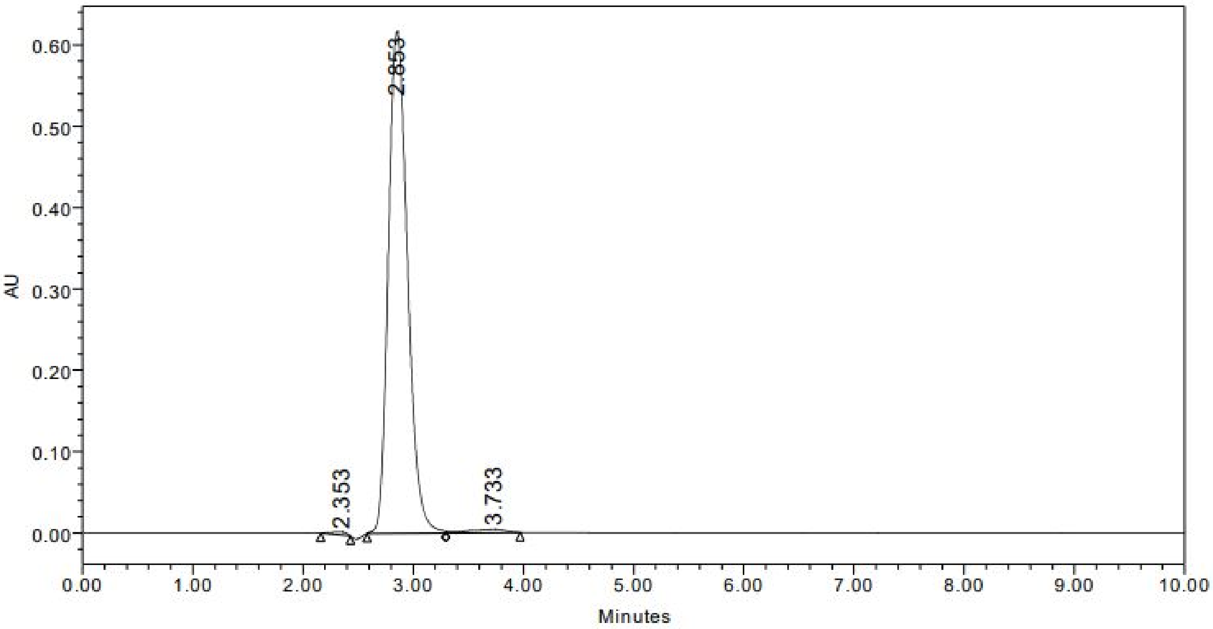

0.60
0.50
0.40
0.30
0.20
0.10
0.00
AU
2.853
2.353
3.733
0.00
1.00
2.00
3.00
4.00
5.00
6.00
7.00
8.00
9.00
10.00
Minutes

(74)

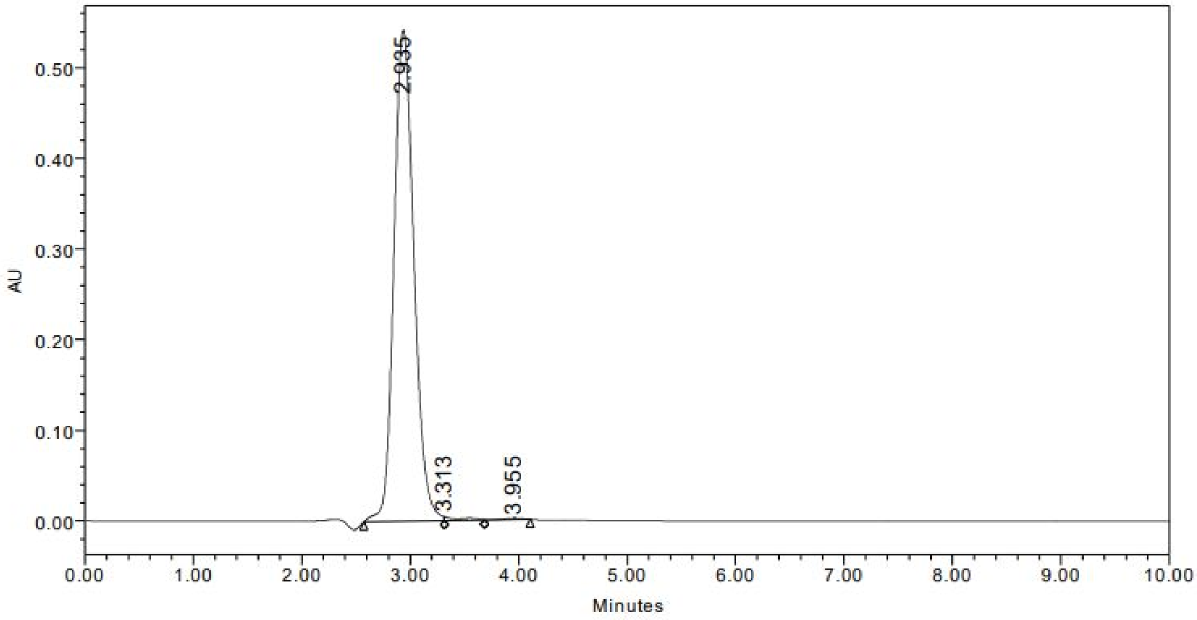

0.50
0.40
0.30
0.20
0.10
0.00
AU
2.935
3.313
3.955
0.00
1.00
2.00
3.00
4.00
5.00
6.00
7.00
8.00
9.00
10.00
Minutes

(75)

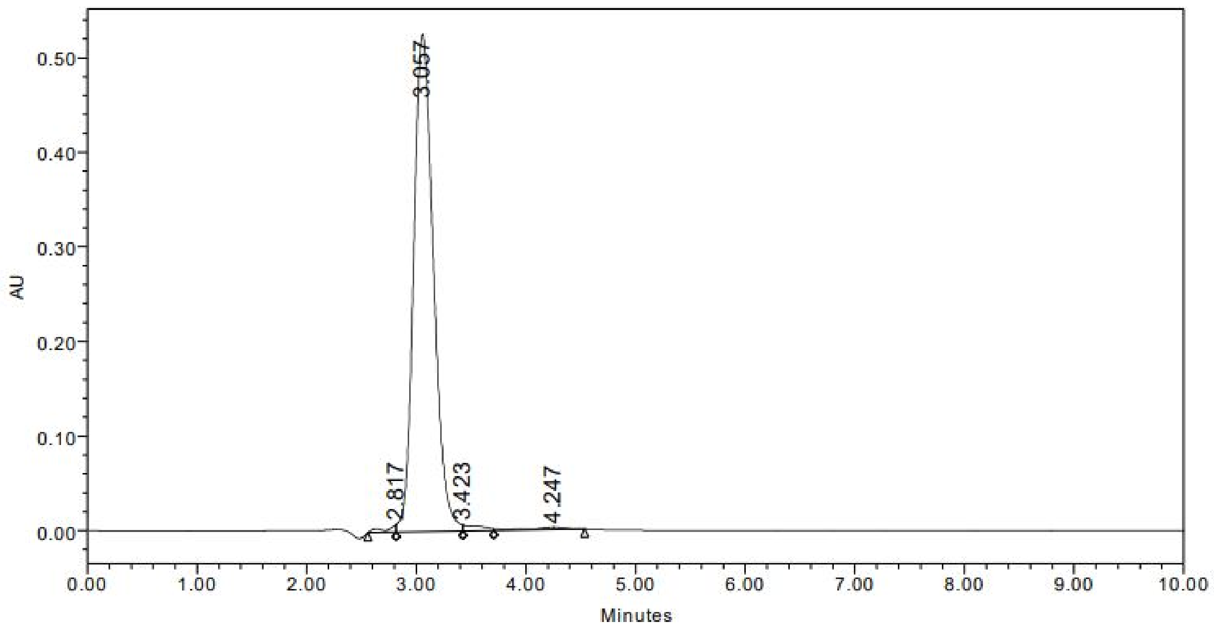

0.50
0.40
0.30
0.20
0.10
0.00
AU
3.057
2.817
3.423
4.247
0.00
1.00
2.00
3.00
4.00
5.00
6.00
7.00
8.00
9.00
10.00
Minutes

(76)

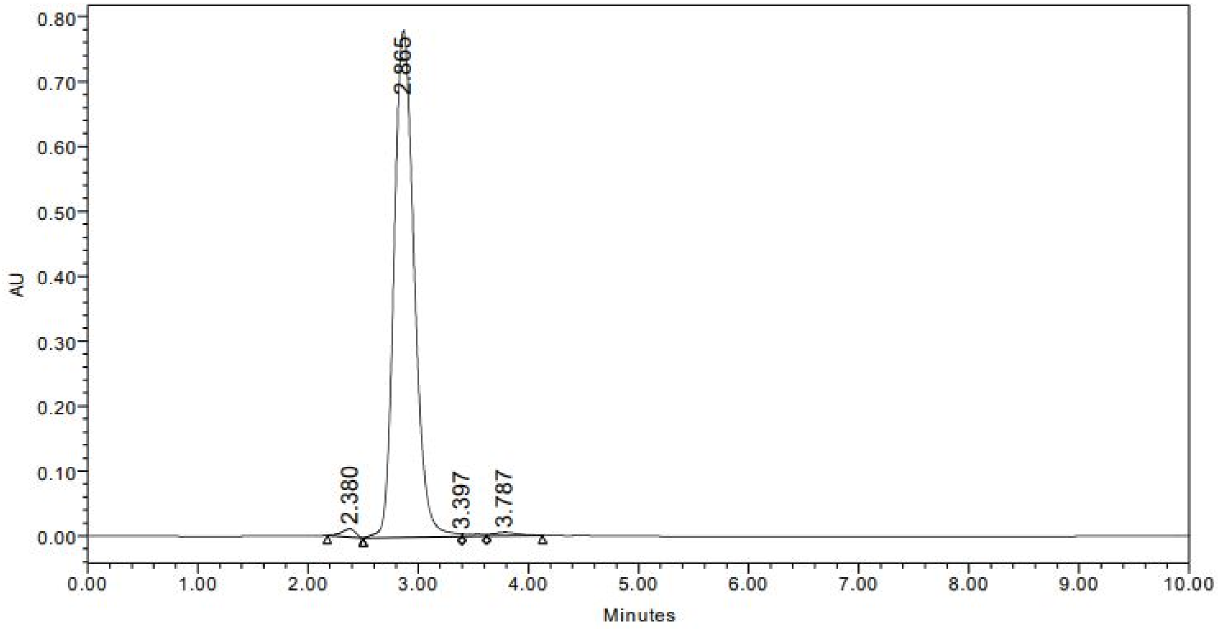

0.80
0.70
0.60
0.50
0.40
0.30
0.20
0.10
0.00
AU
2.865
2.380
3.397
3.787
0.00
1.00
2.00
3.00
4.00
5.00
6.00
7.00
8.00
9.00
10.00
Minutes

(77)

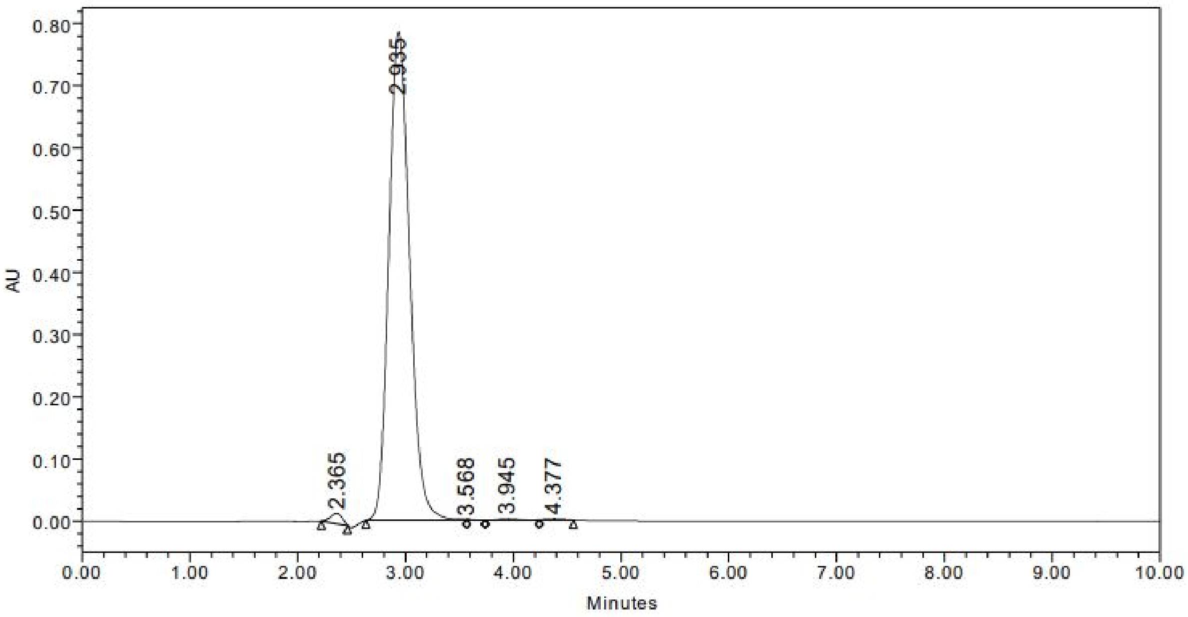
0.80
0.70
0.60
0.50
0.40
0.30
0.20
0.10
0.00
AU
2.935
2.365
3.568
3.945
4.377
0.00
1.00
2.00
3.00
4.00
5.00
6.00
7.00
8.00
9.00
10.00
Minutes

(78)
